# Tracking multidrug resistant tuberculosis: a 30-year analysis of global, regional, and national trends

**DOI:** 10.3389/fpubh.2024.1408316

**Published:** 2024-09-10

**Authors:** Hui-Wen Song, Jian-Hua Tian, Hui-Ping Song, Si-Jie Guo, Ye-Hong Lin, Jin-Shui Pan

**Affiliations:** ^1^Department of Infectious Diseases, Sanming First Hospital Affiliated to Fujian Medical University, Sanming, Fujian, China; ^2^The Graduate School of Fujian Medical University, Fuzhou, Fujian, China; ^3^Department of Hepatology, First Affiliated Hospital of Fujian Medical University, Fuzhou, Fujian, China; ^4^Hepatology Research Institute, Fujian Medical University, Fuzhou, Fujian, China; ^5^Fujian Clinical Research Center for Liver and Intestinal Diseases, Fuzhou, Fujian, China

**Keywords:** disability-adjusted life years, incidence, global burden of disease, multidrug-resistant tuberculosis, socio-demographic index

## Abstract

**Objectives:**

To provide valuable insights for targeted interventions and resource allocation, our analysis delved into the multifaceted burden, trends, risks, and projections of multi drug resistant tuberculosis (MDR-TB).

**Methods:**

This research employed data from the Global Burden of Disease (GBD) 2019 dataset, which used a comparative risk assessment to quantify the disease burden resulting from risk factors. Initially, this database was utilized to extract details concerning the disability-adjusted life years (DALYs), mortality, incidence, and the number of individuals afflicted by MDR-TB. Subsequently, regression analyses were conducted using the Joinpoint program to figure average annual percent change (AAPC) to ascertain the trend. Thirdly, the age-period-cohort model (APCM) was adopted to analyze evolutions in incidence and mortality. Finally, utilizing the Nordpred model within R software, we projected the incidence and mortality of MDR-TB from 2020 to 2030.

**Results:**

MDR-TB remained a pressing global health concern in regions with lower socio-demographic indexes (SDI), where the AAPC in DALYs topped 7% from 1990 to 2019. In 2019, the cumulative DALYs attributed to MDR-TB tallied up to 4.2 million, with India, the Russian Federation, and China bearing the brunt. Notably, the incidence rates have shown a steadfast presence over the past decade, and a troubling forecast predicts an uptick in these areas from 2020 to 2030. Additionally, the risk of contracting MDR-TB grew with advancing age, manifesting most acutely among men aged 40+ in lower SDI regions. Strikingly, alcohol consumption had been identified as a significant contributor, surpassing the impacts of smoking and high fasting plasma glucose, leading to 0.7 million DALYs in 2019.

**Conclusions:**

A robust strategy is needed to end tuberculosis (TB) by 2030, especially in lower SDI areas.

## 1 Introduction

Before the COVID-19 pandemic, tuberculosis had been the primary cause of death from a single infectious agent since 2007, outpacing HIV/AIDS in terms of mortality rates (1). From 2010 to 2020, the incidence of tuberculosis saw an annual decrease of ~2%. However, a rebound occurred from 2020 to 2022 due to the COVID-19 crisis. Of significant

concern is the rising global burden of MDR-TB, which arises when Mycobacterium tuberculosis develops resistance to drugs such as isoniazid and rifampicin. Disturbingly, the incidence of MDR-TB has been rising by more than 20% each year (2). The estimated number of new cases of MDR-TB or rifampicin-resistance TB (MDR/RR-TB) has shown a relatively stable trend from 2020 to 2022, with an estimated 410,000 cases in 2022 (3). Yet, the 2020 study cohort for MDR/RR-TB revealed a mere 63% global treatment success rate for MDR/RR-TB (3). MDR-TB is a major public health threat that not only harms patients' health but also imposes a financial burden on them and their families. According to a report, 81.58% of patients and their families incurred costs ranging from $650 to 8,266 (4). To achieve the goal of MDR-TB elimination by 2030, it is imperative to gain a profound understanding of the disease's global impact and the extent of its transmission.

MDR-TB represents a pressing global concern, impacting numerous countries across the globe. In India, approximately 6.7% of new TB cases are diagnosed with MDR-TB (5). The figures are significantly higher in Russia, where 53.4% of isolated strains exhibit drug resistance, with 67.9% of those being MDR (6). A recent study in China has revealed that 14% of isolated strains fall into the MDR category (7). There is a notable association between the incidence of MDR-TB and age, with young adults, particularly those aged between 26 and 45, being at a higher risk for MDR-TB (8). However, this correlation may differ across regions and specific demographic groups. Previous research has identified alcohol use as an independent risk factor for TB (9). As a distinct form of TB, MDR-TB's relationship with alcohol consumption has been less extensively investigated. Predictive studies on the burden of MDR-TB are of paramount importance for comprehending and managing the disease's dissemination. Despite this, only a limited number of national forecasting studies have been conducted.

By leveraging data from the GBD study, we have embarked on an extensive global investigation into the disease burden, prevalence trends, risk factors, and predictive modeling of MDR-TB, aiming to bridge the existing gaps in research. This endeavor is vital for pinpointing priority areas and specific populations in need of preventative and therapeutic interventions. It aids in optimizing resource allocation and offers evidence-based guidance to policymakers for the development of precision-targeted strategies.

## 2 Methods

### 2.1 Data sources

The MDR-TB data were sourced from the GBD 2019 database (http://ghdx.healthdata.org/gbd-2019), a comprehensive, multinational research platform providing epidemiological insights into 204 territories or countries, encompassing all WHO member states. These countries or regions are grouped into 21 GBD regions based on geographic location, including Sub-Saharan Africa, Oceania, Asia, the Middle East, Europe, the Caribbean, Latin America, Australasia, High Income North America, and High Income Asia-Pacific (10).

The GBD 2019, covering 369 disorders and 87 associated risk factors (11), is an authoritative resource for comprehending global health challenges. The GBD employs a robust set of statistical models to estimate disease burden. Data sources include published literature, official national health department websites, WHO, demographic health surveys, and national and regional disease surveillance systems. To address variations in data quality, comparability, accuracy, and completeness, statistical methods like misclassification correction, rubbish code reassignment, and data noise reduction were applied to enhance data uniformity and comparability. Specifically, the analysis for tuberculosis included 3,613 distinct datasets.

### 2.2 Related terms

GBD 2019 defined the following terms:

**Multidrug-resistant tuberculosis without extensive drug resistance:** the absence of extensive drug resistance, among individuals unaffected by HIV, represents a particular clinical scenario where the disease does not respond to the two most potent first-line antibiotics for treating tuberculosis. However, this form of TB does not exhibit resistance to any fluoroquinolone or any of the second-line injectable drugs.

**Alcohol use:** any alcohol consumption.

**High fasting plasma glucose:** a serum fasting plasma glucose level exceeding 4.8–5.4 mmol/L.

**Smoking:** currently engages in daily or periodic use of smoked tobacco products.

**Disability-adjusted life years (DALYs):** the total of years lost to premature death and years lived with disability.

### 2.3 Socio-demographic index

The SDI, ranging from 0 to 1, is a composite average reflecting the per capita income, educational attainment, and birth rates across all regions included in the GBD study. The SDI quintiles, as depicted in Data 1, are categorized into five tiers: low, low-middle, middle, high-middle, and high, indicating progressively better socio-demographic conditions from the lowest to the highest tier.

### 2.4 Assessing the burden of disease attributable to risk factors

The study conducted on GBD 2019 utilized the Comparative Risk Assessment framework to measure the disease burden caused by specific risk factors (11). To achieve this, the study followed several methodological steps. Initially, the study determined the relative risks (RR) as a measure of exposure to the risk factors. Subsequently, the Bayesian meta-regression model DisMod-MR 2.1 and the spatio-temporal Gaussian process regression model (ST-GPR) were utilized to approximate the mean exposure to each risk factor across all relevant data sources. Then, the Theoretical Minimum Risk Exposure Level (TMREL) was determined as the lowest exposure level to a risk factor observed in cohort studies and experimental trials. Moreover, the Population Attributable Fraction (PAF) was computed using age, sex, location, and time as variables, with the calculation method documented in earlier scholarly works (10). Finally, based on the data from these calculations, the estimates of disease caused by each risk factor were determined.

In this study, we extracted data on incidence, mortality, and DALYs related to MDR-TB from GBD 2019. We conducted a thorough analysis by organizing the data according to gender, age, etiology, geographical region, and SDI level. Additionally, we utilized Joinpoint software, the Age-Period-Cohort model, and the Nordpred tool (with specific methodologies detailed subsequently) to evaluate temporal trends in age-standardized DALYs and mortality rates.

### 2.5 Analysis and forecast of incidence and mortality trends: an age-period-cohort modeling perspective

As previously described in the literature (12), the age-period-cohort model (APCM) was used to analyze and project trends in incidence and mortality for MDR-TB. The input dataset comprised a granular distribution of 20 age brackets, ranging from birth to 95+ years, in increments of 5 years, and covered six distinct time frames, from 1990–1994 to 2015–2019. In addition, the dataset integrated 25 distinct birth cohorts, extending from 1890–1899 to 2010–2019 (Supplementary Table S1). In this study, control groups for ages 40, the 2000–2004 period, and the 1960 cohort. Based on the APCM, longitudinal age-specific rates were generated, which were adjusted for period bias using the reference cohort to reflect the natural history of age-related changes (i.e., the age effect). Simultaneously, the model yielded relative risks (RR) of incidence and mortality for each period (represented by different cohorts), capturing the period-specific influences on disease. The RR is defined as the ratio of the age-specific rate in each period (cohort) to the reference period (cohort). Furthermore, rooted in the APCM, we also employed the Nordpred tool in R to project MDR-TB incidence and mortality from 2020 to 2030.

### 2.6 Evolving patterns of MDR-TB: deciphering trends with joinpoint regression analysis

To delineate the trajectory of MDR-TB burden, comparisons of annual percentage change (APC) values for age-standardized DALY rate (ASDR) and age-standardized mortality rate (ASMR) across varying periods were conducted. In this study, regression analyses were performed utilizing the Joinpoint tool, provided by the National Cancer Institute of the United States of America (13). The APC and average annual percentage change (AAPC) were employed as primary indicators to gauge the progression of MDR-TB. Additionally, AAPC values were scrutinized across gender, etiologies, SDI, and GBD regions from 1990 to 2019. An APC or AAPC value above zero signifies an upward trend, whereas a value below zero indicates a declining trend.

### 2.7 Statistical analysis

Statistical analysis was performed using R 4.3.2, Joinpoint Regression Program (version 4.9.0.0), and GraphPad Prism 9. The Wald χ^2^ test was used for parameter estimation. Test level α = 0.05 (two-sided), *P* < 0.05 (two-tailed) was defined as significant.

## 3 Results

### 3.1 MDR-TB remains a substantial burden in lower SDI areas

From 1990 to 2019, the rate of DALYs attributable to MDR-TB has seen a startling escalation. The Joinpoint analysis exhibited an AAPC of 4.57 [95% confidence interval (CI): 3.89–5.26], signifying a substantial rise from 13.9 [95% uncertainty interval (UI): 4.9–32.5] per 100,000 individuals in 1990 to 52.4 (22.6–97.6) in 2019 (Table 1). The collective DALYs tallied for MDR-TB reached a staggering 4.2 million (1.8–7.8 million) by 2019 (Table 1). It is noteworthy that males were disproportionately affected compared to females. When regarding the SDI, the data revealed an inverse correlation between SDI levels and the number of MDR-TB DALYs. Over the past three decades, there has been a marked and persistent increase in DALYs from MDR-TB in low and low-middle SDI regions, with an AAPC exceeding 7% (Table 1).

**Table 1:** Global and regional trends in DALYs and age-standardized DALY rates of MDR-TB in 1990 and 2019.

**Characteristics**	**1990**		**2019**		**AAPC (%, 95% CI)**	***P*-value**
	**Number of DALYs (95% UI)**	**ASDR (per 10,000, 95% UI)**	**Number of DALYs (95% UI)**	**ASDR (per 100,000, 95% UI)**		
Global	687,571 (241,221–1,592,146)	13.9 (4.9–32.5)	4,191,700 (1,797,321–7,829,596)	52.4 (22.6–97.6)	4.57 (3.89–5.26)	<0.001
**Sex**						
Male	413,265 (145,011–975,770)	17.5 (6.1–41.3)	2,680,762 (1,147,346–4,952,996)	67.2 (28.8–123.3)	4.65 (3.9–5.41)	<0.001
Female	274,306 (93,825–635,302)	10.7 (3.7–24.9)	1,510,938 (643,129–2,834,784)	38.2 (16.4–70.9)	4.38 (3.73–5.04)	<0.001
**Etiology**						
Alcohol use	110,939 (35,805–261,621)	2.5 (0.8–5.9)	748,120 (317,753–1,500,193)	9.0 (3.8–18.1)	4.43 (3.55–5.30)	<0.001
High fasting plasma glucose	32,745 (10,056–83,558)	0.8 (0.2–2.0)	369,041 (124,376–791,533)	4.4 (1.5–9.4)	5.92 (4.57–7.29)	<0.001
Smoking	125,157 (40,841–308,706)	2.9 (0.9–7.2)	583,895 (243,824–1,098,920)	7.0 (2.9–13.1)	2.95 (2.16–3.74)	<0.001
**Socio-demographic index (SDI)**						
High	17,031 (7,368–32,645)	1.8 (0.8–3.5)	11,971 (5,402–23,300)	0.8 (0.4–1.7)	1.32 (0.25–2.39)	0.015
High-middle	117,268 (31,993–327,699)	10.5 (2.9–29.1)	320,078 (168,883–554,057)	18.2 (9.6–31.9)	−7.69 (−8.16 to −7.22)	<0.001
Middle	322,070 (94,820–823,734)	22.7 (6.8–57.9)	676,027 (266,514–1,281,464)	26.7 (10.6–50.5)	0.29 (−0.77 to 1.36)	0.597
Low-middle	147,916 (41,290–420,912)	15.7 (4.4–44.2)	1,885,643 (611,735–4,182,591)	117.8 (37.8–262.7)	7.61 (7.04–8.18)	<0.001
Low	83,144 (28,692–190,324)	18.9 (6.6–44.4)	1,296,925 (571,961–2,286,886)	159.4 (69.9–280.2)	7.14 (6.38–7.90)	<0.001
**Regions**						
Andean Latin America	12,908 (2,868–38,850)	36.6 (8.2–108.3)	18,577 (7,149–38,533)	30.0 (11.6–62.8)	−1.09 (−1.89 to −0.27)	0.009
Australasia	40 (9–112)	0.2 (0.0–0.5)	126 (44–278)	0.3 (0.1–0.6)	1.47 (0.63–2.31)	0.001
Caribbean	1,506 (339–4,731)	4.3 (1.0–13.3)	1,109 (250–3,505)	2.3 (0.5–7.4)	−1.83 (−3.26 to −0.38)	0.013
Central Asia	1,381 (344–3,846)	2.1 (0.5–6.0)	77,250 (44,235–116,514)	80.5 (46.1–121.6)	12.96 (10.99–14.96)	<0.001
Central Europe	2,573 (911–6,006)	1.9 (0.7–4.6)	2,673 (1,039–5,682)	1.8 (0.7–3.8)	−0.39 (−1.80 to 1.13)	0.613
Central Latin America	1,531 (440–4,088)	1.2 (0.3–3.1)	11,657 (4,115–27,134)	4.7 (1.7–10.9)	4.73 (3.82–5.64)	<0.001
Central Sub-Saharan Africa	24,606 (4,223–92,181)	45.7 (8.5–178.9)	167,193 (36,270–534,733)	177.9 (38.7–571.7)	4.78 (3.88–5.68)	<0.001
East Asia	424,758 (91,032–1,155,907)	39.5 (8.5–107.5)	101,473 (23,483–280,059)	5.5 (1.3–15.0)	−7.12 (−7.78 to −6.45)	<0.001
Eastern Europe	15,554 (4,767–43,009)	6.1 (1.9–17)	167,030 (98,686–247,486)	65.4 (38.8–96.6)	8.29 (7.19–9.41)	<0.001
Eastern Sub-Saharan Africa	18,015 (5,620–49,633)	13.1 (4.1–36.0)	573,822 (248,057–1,089,249)	204.2 (88.9–390.1)	9.92 (9.24–10.59)	<0.001
High-income Asia Pacific	4,659 (1,180–14,255)	2.4 (0.6–7.6)	2,378 (475–7,085)	0.6 (0.1–1.9)	−2.82 (−3.16 to −2.48)	<0.001
High-income North America	4,152 (1,697–8,256)	1.3 (0.5–2.5)	717 (252–1,705)	0.1 (0.0–0.3)	−5.51 (−6.44 to −4.58)	<0.001
North Africa and Middle East	4,807 (1,641–11,626)	1.7 (0.6–4.1)	39,285 (15,342–86,071)	7.1 (2.8–15.5)	5.14 (4.12–6.17)	<0.001
Oceania	74 (14–300)	1.5 (0.3–5.7)	5,582 (1,371–13,989)	49.6 (12.2–125.2)	13.01 (11.9–14.13)	<0.001
South Asia	70,749 (11,913–250,566)	8.2 (1.3–29.2)	2,365,403 (599,234–5,484,039)	144.3 (36.2–335.0)	10.43 (9.63–11.24)	<0.001
Southeast Asia	49,347 (12,574–146,934)	13.7 (3.7–39.6)	191,192 (81,734–394,630)	29.5 (12.8–60.6)	2.42 (0.94–3.92)	0.001
Southern Latin America	725 (144–2,281)	1.5 (0.3–4.7)	972 (227–3,046)	1.3 (0.3–4.1)	−0.76 (−1.76 to 0.24)	0.136
Southern Sub-Saharan Africa	12,073 (2,570–38,973)	26.2 (5.5–83.8)	116,813 (41,859–242,996)	157.7 (56.4–329.4)	5.68 (4.24–7.14)	<0.001
Tropical Latin America	529 (65–2,377)	0.4 (0.0–1.8)	12,175 (2,302–33,242)	5.0 (1.0–13.7)	9.24 (8.47–10.02)	<0.001
Western Europe	3,437 (1,314–7,802)	0.7 (0.3–1.5)	2,751 (1,311–5,169)	0.4 (0.2–0.7)	−2.23 (−3.04 to −1.41)	<0.001
Western Sub-Saharan Africa	34,148 (12,048–78,112)	21.3 (7.5–47.3)	333,523 (119,994–704,207)	102.1 (36.7–213.2)	5.49 (4.58–6.41)	<0.001

AAPC, average annual percent change; ASDR, age-standardized DALY rates; CI, confidence interval; DALYs, Disability-Adjusted Life Years; MDR-TB, Multidrug-resistant tuberculosis without extensive drug resistance; UI, uncertainty interval.

Regionally, the greatest burden of MDR-TB fell on South-East Asia and Sub-Saharan Africa in 2019. South Asia alone accounted for 2.4 million DALYs (range: 0.6–5.5 million), constituting 56.4% of the global total. The following regions also had significant burdens: Eastern Sub-Saharan Africa at 13.7%, Western Sub-Saharan Africa at 8.0%, Southeast Asia at 4.6%, Central Sub-Saharan Africa and Eastern Europe at 4.0%, Southern Sub-Saharan Africa at 2.8%, and East Asia at 2.4%. Significantly, the top three GBD regions for ASR of DALYs were Eastern Sub-Saharan Africa at 204.2 (88.9–390.1) per 100 000 population, Central Sub-Saharan Africa at 177.9 (38.7–571.7), and Southern Sub-Saharan Africa at 157.7 (56.4–329.4) (Table 1). Within the ranks of the top three GBD regions grappling with a high MDR-TB burden, the ASDRs have continued to escalate. The AAPC was 10.43 (9.63–11.24) in South Asia, 9.92 (9.24–10.59) in Eastern Sub-Saharan Africa, and 5.49 (4.58–6.41) in Western Sub-Saharan Africa (Table 1).

**Figure 1:**
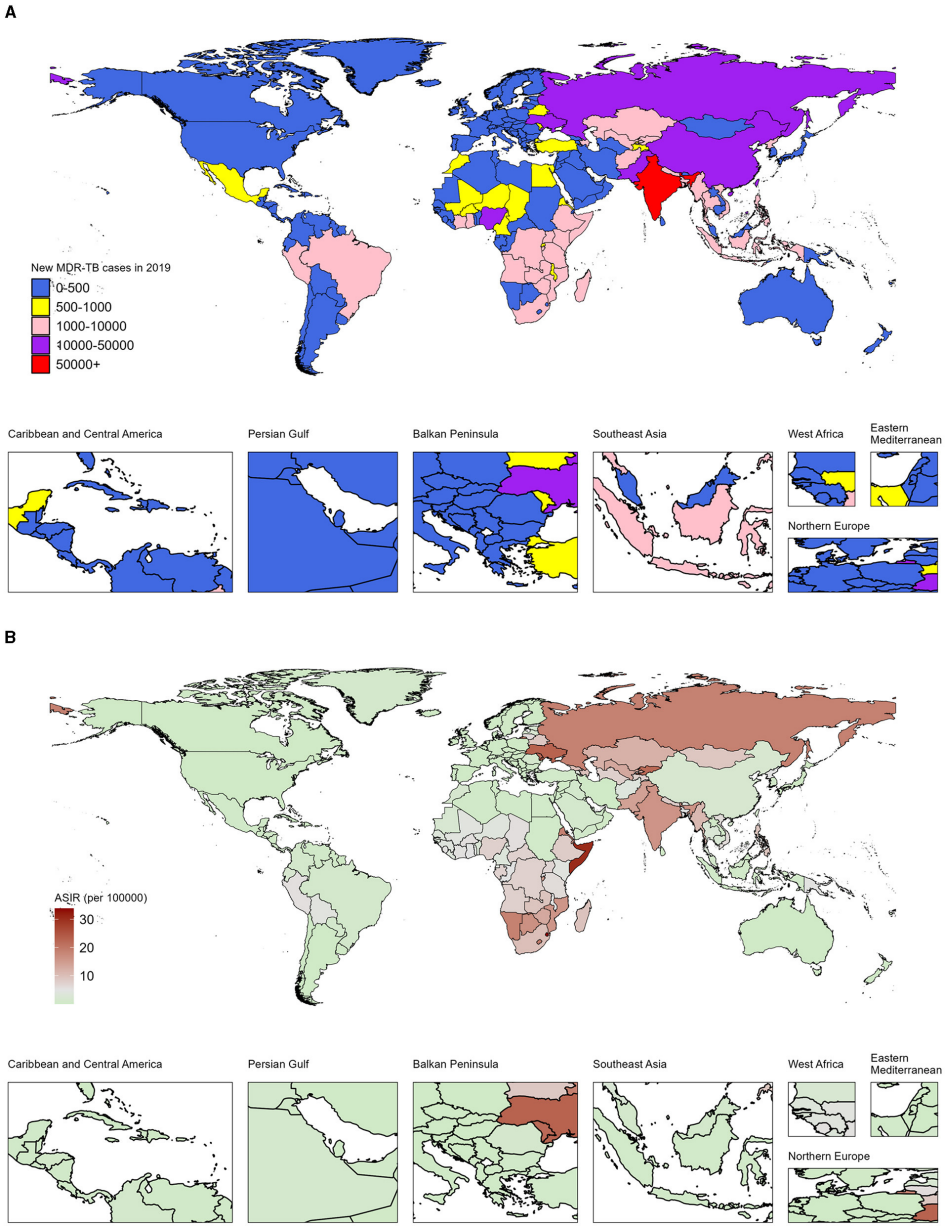
Number and age-standardized rates of incidence from MDR-TB among HIV-negative individuals in 204 countries and territories, 2019. **(A)** Number of new cases of MDR-TB across all ages. **(B)** Age-standardized incidence rates of MDR-TB.

Specifically, in 2019, India (South Asia), Russia (Eastern Europe), and China (Eastern Asia) led in the number of DALYs across 204 countries and territories. India topped the list with 220,000 cases, ahead of Russia with 31,000 and China with 30,000 cases (Figure 1A; Data 1, p. 15–19). Additionally, the top 10 countries by case count include South Asian nations: Pakistan (28,000 cases), Bangladesh (8,000 cases); Western sub-Saharan Africa's Nigeria (12,000 cases); Eastern European: Ukraine (12,000 cases); and South-East Asian: Philippines (9,000 cases), Viet Nam (6,000 cases), along with Eastern sub-Saharan Africa's Ethiopia (7,000 cases). In sub-Saharan Africa, Eswatini, Somalia, and Comoros exhibited the highest ASDRs of 33.9 (8.1–83.2), 30.4 (8.7–87.5), and 26.6 (3.4–87.0) per 100,000 population in the same period, respectively (Figure 1B; Data 1, p. 20–23). Additionally, the analysis of DALYs associated with MDR-TB exhibits a comparable pattern, as illustrated in Supplementary Figure S1 (Data 1, p. 87–95).

### 3.2 Alcohol consumption emerges as the leading cause, particularly among middle-aged men

Alcohol was the primary cause of DALYs from MDR-TB, surpassing smoking and high fasting plasma glucose levels. In 2019, alcohol-related DALYs from MDR-TB reached a staggering 0.7 million, with an AAPC of 4.43 (3.55–5.30) percent (Table 1).

**Figure 2:**
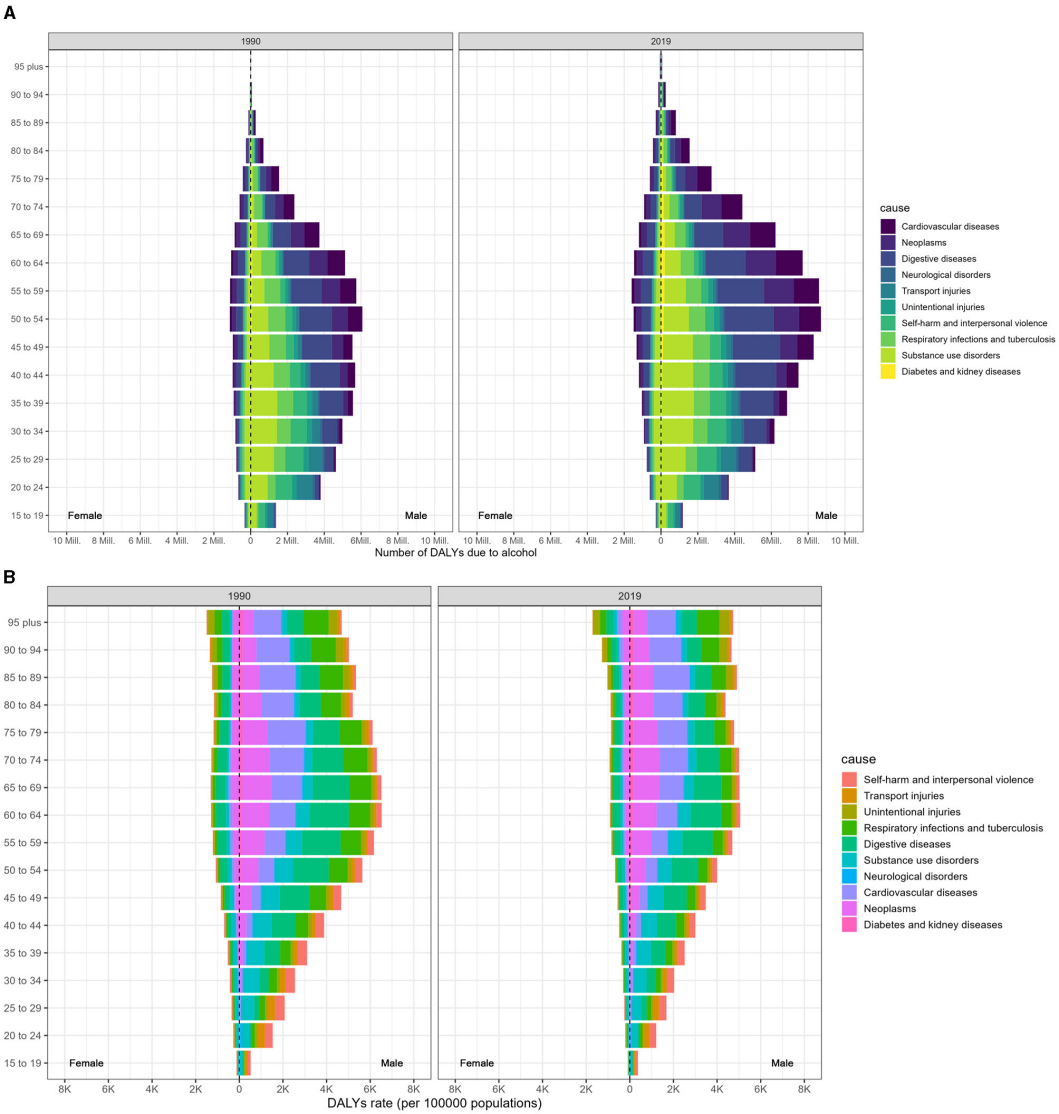
Age-specific distribution of DALYs resulting from alcohol consumption at the GBD level 2 causes in 1990 and 2019, categorized by sex. **(A)** Age-specific distribution of DALYs. **(B)** Age-specific DALYs rate.

Compared to females, males were predominantly affected by alcohol consumption. Additionally, the worldwide burden of DALYs caused by alcohol consumption was considerably higher for men in 2019 than in 1990, as shown in Figure 2. In 2019, respiratory infections and tuberculosis linked to alcohol use were the leading causes of DALYs among men aged 45–49, 50–54, and 40–44, with respective tallies of 926 (650–1,163), 890 (629–1,137), and 888 (619–1,126) thousand (Figure 2A; Data 1, p. 24–34). These figures were eight to ten times higher than those for women in the same age brackets. Likewise, the ASDR associated with alcohol consumption was also markedly higher in men than in women and increased with age (Figure 2B; Data 1, p. 35–45). Furthermore, Supplementary Figure S2 illustrates the corresponding mortality data (Data 1, p. 96–115).

**Figure 3:**
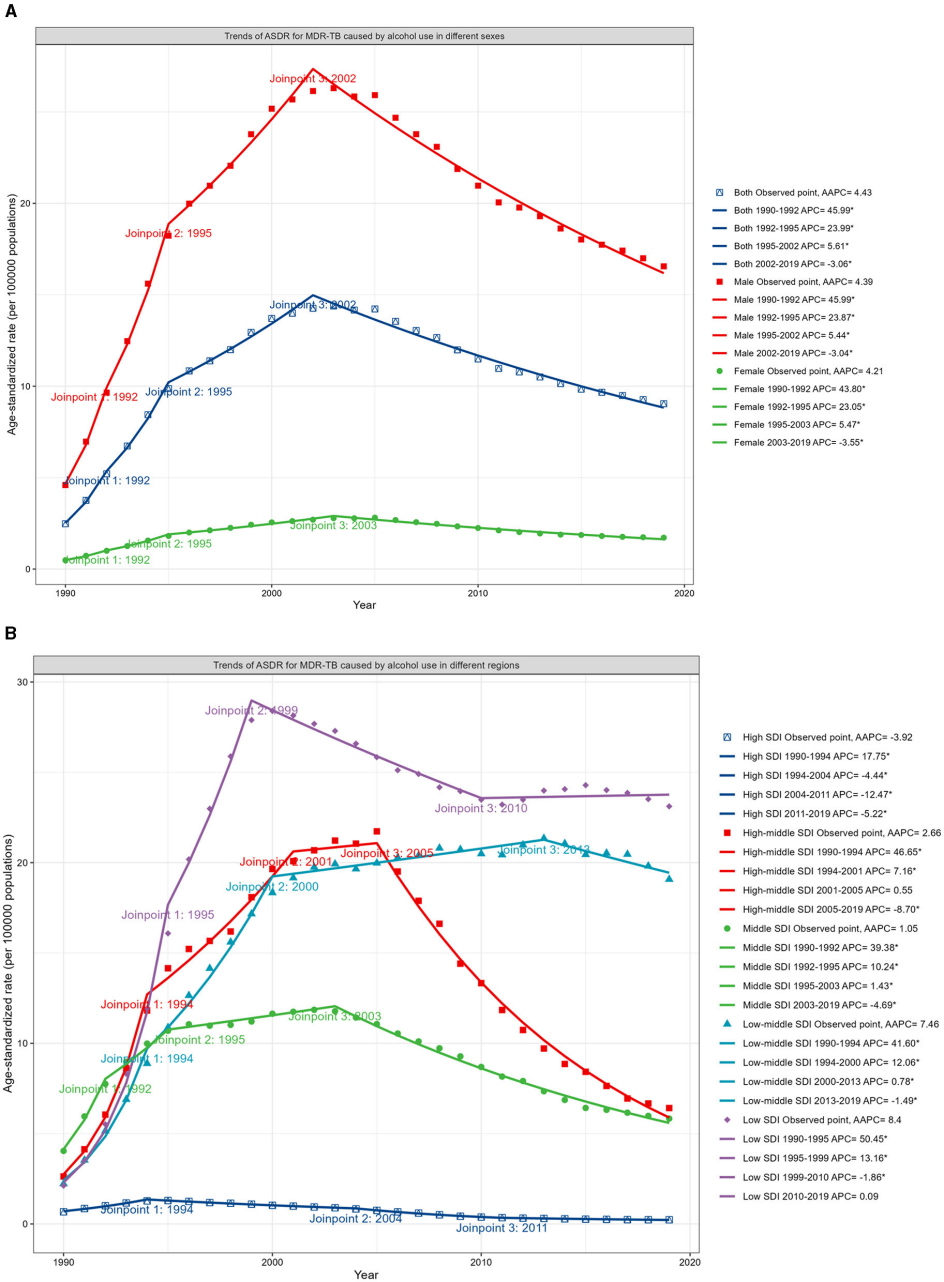
Trends in ASDR for MDR-TB in HIV-negative populations ascribable to alcohol consumption, differentiated by sex and SDI, 1990–2019. **(A)** Trends in ASDR, by sex. **(B)** Trends in ASDR, by SDI.

Analysis via Joinpoint regression revealed a mounting trend in the ASDR for MDR-TB until 2002, which was then followed by a decline from 2002 to 2019, with an APC of −3.06 (Figure 3A; Data 1, p.46–47). The turning point for this downward trend occurred earliest in the high SDI regions in 1994, while in the low SDI regions, it appeared until 1999. Nevertheless, the decline in DALYs for regions with low SDI levels plateaued after 2010 (Figure 3B; Data 1, p. 48–51). Moreover, Supplementary Figure S3 presents the associated mortality data (Data 1, p. 116–121).

### 3.3 Age, period, and cohort effects on MDR-TB

**Figure 4:**
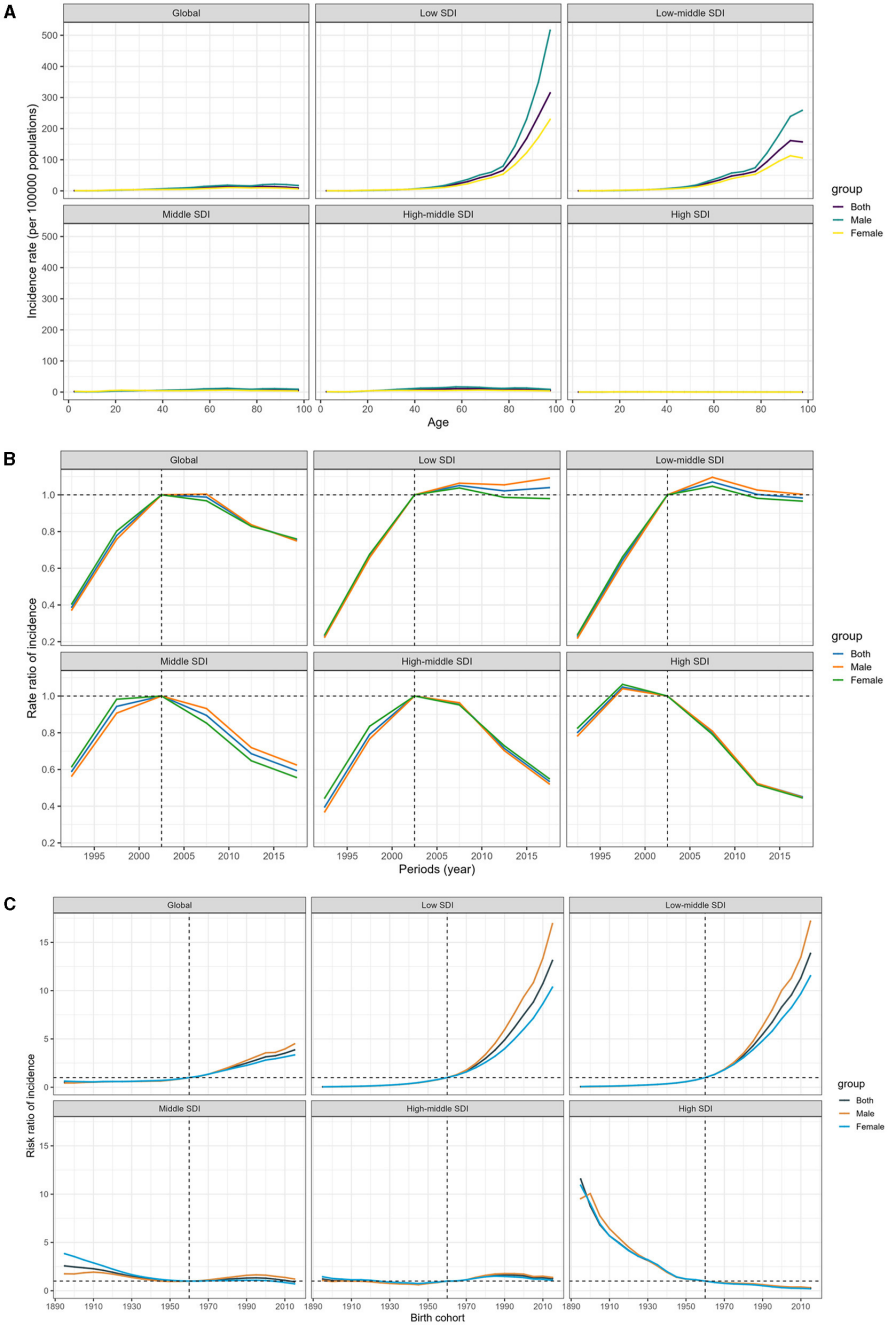
Age-period-cohort effect on the relative risk of MDR-TB among HIV-free populations, stratified by SDI. **(A)** Age effect. **(B)** Period effect. **(C)** Cohort effect.

Figure 4 shows the age-period-cohort effects from the APCM analysis, categorized by SDI quintile. Age curves present age effects, period effects display the relative risk of incidence by period, and cohort effects show the relative risk of incidence by cohort. Globally, there has been a consistently lower incidence of MDR-TB across all age groups. Nonetheless, it is concerning that the risk of developing MDR-TB increases with age, especially in men aged 40 and above in regions with lower SDI, in stark contrast to regions with higher SDI, where the risk is diminishing (Figure 4A; Data 1, p. 52–59).

Between 1990 and 2000, there was a troubling increase in the risk of MDR-TB incidence. However, the tide began to turn after 2000, and over the past decade, we have seen a sustained reduction in the incidence risk. The decline in high-SDI countries started earlier, and this downward trend was mirrored in the middle, high-middle, and high-SDI zones. In contrast, low-middle and low-SDI regions have shown little to no improvement in the incidence rates over the last decade, suggesting a stagnation in efforts to combat the disease (Figure 4B; Data 1, p. 60–61).

Worldwide, individuals born after 1960 have been increasingly susceptible to MDR-TB. The impact of declining birth cohorts has been more pronounced in higher-SDI countries. These countries have witnessed a progressive improvement in the incidence of MDR-TB for those born after the 1960s, while the risk in low and low-middle SDI countries has conversely mounted, with a particular increase observed in men. For individuals born in 2015, the risk ranged from 13.85 (95% CI 12.54–15.30) in low-middle SDI countries to a significantly lower 0.26 (0.18–0.38) in high SDI locations, in comparison to those born in 1960 (Figure 4C; Data 1, p. 62–70). In addition, Supplementary Figure S4 displays the correlative mortality data (Data 1, p. 122–141).

### 3.4 By 2030, an anticipated surge in low-middle and low SDI regions

**Figure 5:**
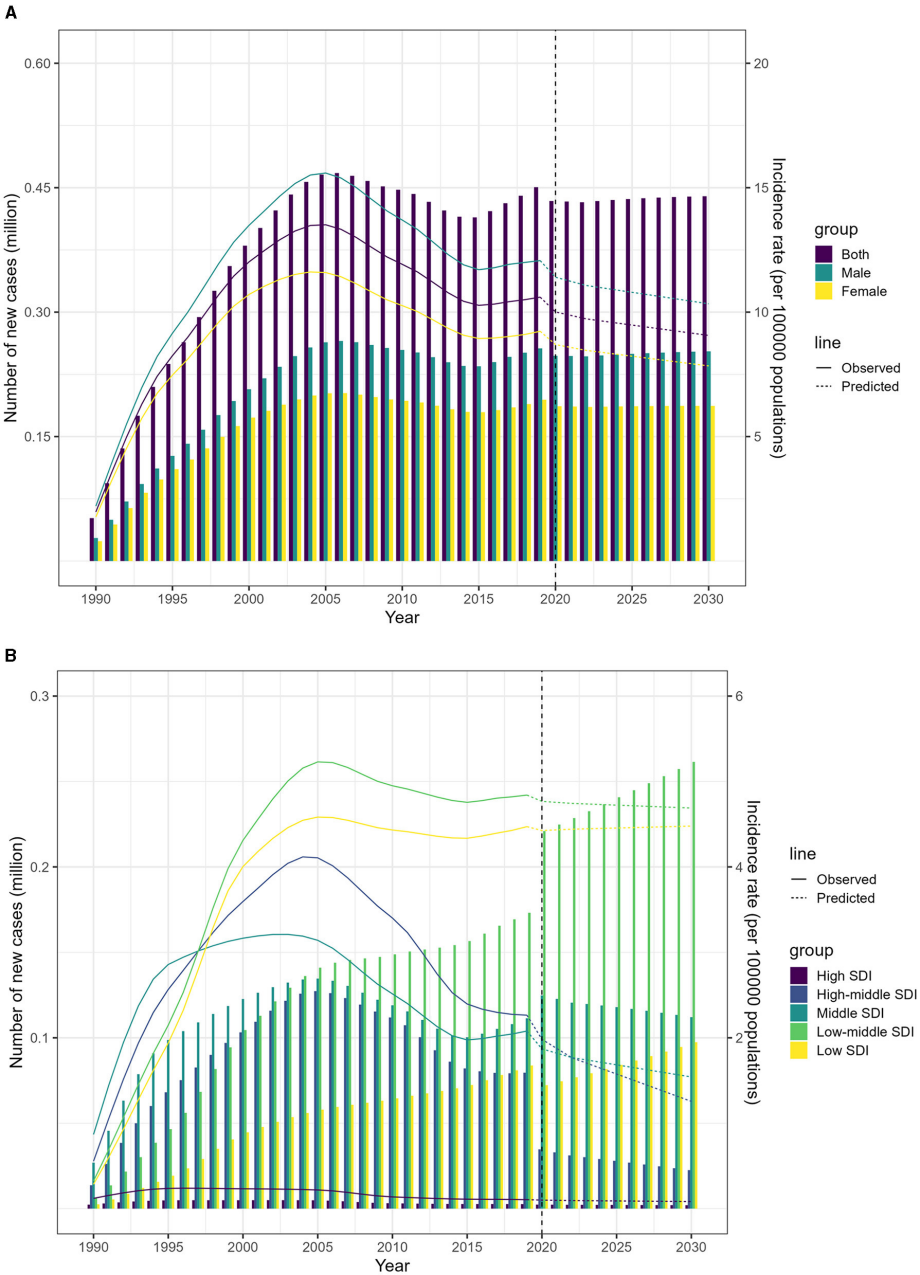
Projected incidence of MDR-TB from 2020 to 2030 using the Nordpred model. **(A)** Projected incidence rates and numbers, by sex. **(B)** Projected incidence rates and numbers, by SDI.

According to the Nordpred model, the number of people affected by MDR-TB is expected to decrease slightly between 2020 and 2030. The age-standardized incidence rate (ASIR) is projected to drop from 5.6 in 2019 to 4.8 per 100,000 population by 2030 (Figure 5A; Data 1, p. 71–85). However, the forecasting for 2030 indicates that the low-middle and low SDI regions may see a significant rise in new cases, with an estimated 261,473 and 97,419 individuals affected, respectively. This translates to an ASIR of 10.0 and 9.5 per 100,000 individuals (Figure 5B; Data 1, p. 71–85). Additionally, Supplementary Figure S5 illustrates the related death data (Data 1, p. 142–156).

## 4 Discussion

This research provides a comprehensive analysis of the global trajectory and projection of MDR-TB in HIV-negative individuals. Our main findings are delineated as follows: (a) MDR-TB persists as a critical global health issue, particularly impacting areas with lower SDI, such as South-East Asia and Sub-Saharan Africa. (b) Alcohol consumption plays a pivotal role in exacerbating the MDR-TB burden. (c) The risk of developing MDR-TB increases with age, especially in men aged 40 and above in lower SDI areas. (d) An anticipated rise in the incidence of MDR-TB is projected for low-middle and low-SDI regions by 2030.

By computing ASDRs and AAPCs, we determined that the global burden of MDR-TB in 2019 was substantially greater than in 1990. Fortunately, our analysis using the APCM revealed a consecutive annual decrease in the incidence rate from 2010 to 2019. This finding harmonizes with the WHO's Global TB Report, which indicated a slowing decline between 2015 and 2019 (1). Nonetheless, the incidence of MDR-TB has failed to diminish in regions characterized by low and medium-low SDI levels over the past decade. Notably, all South Asian countries, except Bhutan, are high-burden MDR-TB countries, based on the number of new cases in 2019. MDR-TB is heavily concentrated in these areas, showing an inverse relationship between SDI levels and the MDR-TB burden. At the country level, India, Russia, and China top the list of countries with the highest number of MDR-TB cases globally in 2019. This corresponds with the WHO's 2020 Global TB Report (1), which underscores the urgency to strengthen TB control efforts in India. TB is more prevalent among individuals with lower socio-economic status, lesser education, unemployment, substandard housing, and inadequate water supply (14). Unless effective control strategies are promptly implemented in high-burden regions post-COVID-19 epidemic—which has witnessed a resurgence (15, 16), —the aspiration to eliminate TB by 2030 may well be jeopardized (17).

Alcohol consumption has emerged as a predominant factor contributing to the scourge of MDR-TB, outstripping both smoking and high fasting plasma glucose, particularly among middle-aged men. Previous research has documented that alcoholism is a critical risk factor for the onset of drug-resistant TB (18), and is correlated with specific medical comorbidities as well as exacerbated illness severity (19). Excessive alcohol consumption wreaks havoc on the immune response of alveolar macrophages, undermining their vital roles in phagocytosis, cytokine and chemokine secretion, and neutrophil chemoattraction (20). Chronic alcohol ingestion also intensifies oxidative stress within the alveolar space (21, 22), potentially fostering the growth of Mycobacterium tuberculosis. Furthermore, among those without HIV, the MDR-TB burden in middle-aged men due to alcohol use was a staggering 8–10 times greater than that in their female counterparts. Men are more prone to binge drinking than women (23). Excessive alcohol intake poses a substantial health risk for males, and the severity of the hazard escalates with the volume of alcohol consumed. Other comorbidity risks are also more prevalent among men, further compounding the risk of illness or mortality in conjunction with alcohol misuse. Thus, it is imperative to prioritize screening among high-risk populations, with particular attention to those who indulge in alcohol consumption and middle-aged men.

The risk of developing MDR-TB increases with advancing age, particularly among middle-aged and older males in regions with lower SDIs. Consistent with our findings, the APCM has also demonstrated an aging effect on the risk of tuberculosis in South Africa (24). A comprehensive meta-analysis has similarly highlighted an elevated risk of MDR-TB among individuals aged 40 and above (25). However, a systematic review reached a contrasting conclusion, observing an increased pooled risk of MDR-TB among patients younger than 45 years from Iran and its neighboring countries (18). In parallel, two retrospective studies conducted in South India and China have found a negative correlation between older age (over 60 years) and the occurrence of MDR-TB (26, 27). As individuals age, the likelihood of underlying diseases that may compromise their immune system also rises. This vulnerability makes them more prone to contracting tuberculosis after exposure. Middle-aged and older men, who frequently have habits such as smoking, alcoholism, and diabetes, are particularly vulnerable to the disease. To date, there remains no consensus on the relationship between age and the risk of MDR-TB. Larger, prospective cohort studies are essential to elucidate any potential links between these factors and to inform targeted prevention and treatment strategies.

Our study has revealed that in 2019, India was responsible for nearly half of the global MDR-TB cases, with a disease burden that far outstrips any other nations. Furthermore, according to the Nordpred model, we can anticipate an increase in the incidence of MDR-TB over the next decade in low-middle and low SDI regions. The predominant factors contributing to this trend are the substantial number of cases in India, which significantly influences the overall trend, and the generally low efficacy of MDR-TB treatments, resulting in a rising caseload. This projected rise could pose a substantial challenge to the achievement of the United Nations Sustainable Development Goals, which targets the elimination of tuberculosis by 2030 (with an 80% reduction in the TB incidence rate compared with levels in 2015). The anticipated escalating incidence of MDR-TB threatens to undermine these global health objectives, necessitating urgent and targeted interventions to improve detection, treatment, and prevention strategies in affected regions. In harmony with our findings, a study utilizing a dynamic Markov model has projected that without a significant increase in treatment detection and coverage rates, MDR-TB cases in China could double to 58 per 100,000 individuals by 2050 (28). Preserving Effective TB Treatment Study projections indicate that by 2040, the proportion of MDR-TB cases is expected to rise in four high-burden countries: India (12.4%), the Philippines (8.9%), Russia (32.5%), and South Africa (5.7%) (29). The increase in MDR-TB cases is attributable to inadequate diagnosis and treatment, delays in effective management, and the proliferation of multi-drug resistance. The COVID-19 pandemic has redirected resources away from TB prevention and control efforts. To meet the goal of eradicating TB by 2030, countries with a high burden of MDR-TB, particularly India, must embrace a comprehensive, multifaceted strategy for TB management (30).

## 5 Limitations

It is crucial to recognize the inherent limitations of our study, which should be taken into account when interpreting the findings. A notable constraint is the reliance on secondary sources for the MDR-TB data utilized in our analysis. Using such data means that we are subject to the limitations of the original data collection methods, potentially leading to observation bias, information bias, and wide uncertainty intervals (UIs) for some cross-sectional data estimates. These broad UIs can introduce variability into the data analysis process and may compromise the precision of subsequent decision-making based on our results.

Additionally, the reliability of data from low- and middle-income countries is a concern, given the incomplete nature of MDR-TB surveillance systems in these regions. This incompleteness could lead to underreporting or misclassification, underestimations, or overestimations of the true burden of MDR-TB, thereby affecting the accuracy of the age-period-cohort effects we have identified. Similarly, the Nordpred model's predictions may be compromised by the quality of the input data, potentially skewing our understanding of the temporal trends in MDR-TB.

Lastly, the GBD 2019 analysis does not differentiate the risk factors associated with alcohol consumption by dose. This limitation raises an important question about the relationship between alcohol intake and the risk of MDR-TB. The absence of dose differentiation means that we cannot conclusively determine whether there is a linear relationship between increasing levels of alcohol consumption and the risk of developing MDR-TB. This oversight could have significant implications for public health interventions aimed at reducing MDR-TB incidence through alcohol-related risk reduction strategies.

These limitations underscore the need for caution in interpreting our findings. The potential inaccuracies in the data and the lack of granularity in risk factor analysis may affect the generalizability of our conclusions. Consequently, future research should aim to address these gaps by incorporating primary data collection, improving surveillance systems in resource-limited settings, and conducting more nuanced analyses of risk factors, including the dose-response relationship between alcohol consumption and MDR-TB risk.

## 6 Conclusion

Our investigation provides a profound global overview of MDR-TB, scrutinizing its disease impact, incidence patterns, risk factors, and predictive dynamics. MDR-TB poses a critical global health threat, disproportionately affecting regions with lower SDI. Projections indicate a rise in MDR-TB incidence in these regions between 2020 and 2030. The risk of MDR-TB increases with age, particularly among males over 40 in low SDI areas. To fulfill the vision of TB elimination by 2030, particularly for lower SDI regions, such as India, it is imperative to embrace a comprehensive, multifaceted TB management strategy. Prioritizing screening among high-risk populations, with a specific focus on those with alcohol habits and middle-aged men, is an imperative measure.

## Data Availability

The original contributions presented in the study are included in the article/Supplementary material, further inquiries can be directed to the corresponding author.
