# Supplementary material for: Tracking multidrug resistant tuberculosis: a 30-year analysis of global, regional, and national trends
Source: Front Public Health. 2024 Sep 10;12:1408316. doi: 10.3389/fpubh.2024.1408316 (PMC11421170; doi:10.3389/fpubh.2024.1408316)
Supplement: Supplementary file 1 [file Data_Sheet_1.pdf]

MDR-TB projected to rise in low and low-middle income regions by 2030: a 30-year data-driven review

Hui-Wen Song, Jian-Hua Tian, Hui-Ping Song, Si-Jie Guo, Ye-Hong Lin, Jin-Shui Pan

Contents of Data 1••**1**

List of abbreviations••**4**

Socio-Demographic Index values for all estimated GBD 2019 locations, 1990-2019•••**5**

SDI Reference Quintiles, Related to Socio-demographic Index•••••••••**12**

GBD Causes Mapped to ICD-9 and ICD-10 Codes, Related to ICD codes for the diseases attributable to alcohol use•••••••••••••••••••••••••••**13**

Figure 1 Number and age-standardized rates of incidence from MDR-TB among HIV-negative individuals in 204 countries and territories, 2019•••**15**

Figure 1A Number of new cases of MDR-TB across all ages•••••••••15

Figure 1B Age-standardized incidence rates of MDR-TB••••••••••••••20

Figure 2 Age-specific distribution of DALYs resulting from alcohol consumption at the GBD level 2 causes in 1990 and 2019••••••••••••••**24**

Figure 2A Age-specific distribution of DALYs•••••••••••••••••••••••••••24

Figure 2B Age-specific DALYs rate•••••••••••••••••••••••••••••••••••••35

Figure 3 Trends in ASDR for MDR-TB in HIV-negative populations

ascribable to alcohol consumption, differentiated by sex and SDI, 1990-2019······46

Figure 3A Trends in ASDR, by sex······46

Figure 3B Trends in ASDR, by SDI······48

Figure 4 Age-period-cohort effect on the relative risk of MDR-TB among HIV-free populations, stratified by SDI······52

Figure 4A Age effect······52

Figure 4B Period effect······60

Figure 4C Cohort effect······62

Figure 5 Projected incidence of MDR-TB from 2020 to 2030 using the Nordpred model, by sex and SDI······71

Table S1 The lexis diagram of incidence data for the age-period-cohort model······86

Figure S1 Number and age-standardized rates of DALYs from MDR-TB among HIV-negative individuals in 204 countries and territories, 2019······87

S.Figure 1A Number of DALYs from MDR-TB across all ages······87

S.Figure 1B Age-standardized DALYs rate of MDR-TB······92

Figure S2 Age-specific distribution of deaths due to alcohol use at GBD level 2 causes in 1990 and 2019······96

S.Figure 2A Age-specific distribution of deaths······96

S.Figure 2B Age-specific mortality rate······106

Figure S3 Trends in ASMR for MDR-TB among HIV-negative individuals resulting from alcohol use, classified by sex and SDI, 1990-2019······116

S.Figure 3A Trends in ASMR, by sex······116

S.Figure 3B Trends in ASMR, by SDI······118

Figure S4 Age-period-cohort impact on the risk of mortality from MDR-TB in HIV-negative individuals, stratified by SDI······**122**

S.Figure 4A Age-related variation······122

S.Figure 4B Period influence······131

S.Figure 4C Cohort effect······134

Figure S5 Projected mortality outcomes from MDR-TB: 2020-2030 forecast based on the Nordpred model, segmented by sex and SDI······**142**

List of abbreviations

Abbreviation	Full phrase
AAPC	average annual percent change
APC	annual percent change
APCM	age-period-cohort model
ASDR	age-standardized DALY rate
ASIR	age-standardized incidence rate
ASMR	age-standardized mortality rate
CI	Confidence interval
DALYs	disability-adjusted life years
GBD	global burden of disease
MDR	multi-drug resistant
MDR-TB	multi-drug resistant tuberculosis
RR-TB	rifampicin-resistance tuberculosis
RR	relative risks
SDI	socio-demographic index
TB	tuberculosis
UI	Uncertainty intervals

Socio- Demographic Index values for all estimated GBD 2019 locations, 1990-2019

Location	1990	1991	1992	1993	1994	1995	1996	1997	1998	1999	2000	2001	2002	2003	2004	2005	2006	2007	2008	2009	2010	2011	2012	2013	2014	2015	2016	2017	2018	2019
Global	0.511	0.516	0.521	0.525	0.529	0.534	0.538	0.542	0.547	0.551	0.556	0.561	0.566	0.571	0.576	0.581	0.586	0.591	0.596	0.601	0.607	0.612	0.616	0.621	0.626	0.631	0.635	0.641	0.647	0.651
Central Europe, eastern Europe, and central Asia	0.648	0.654	0.662	0.666	0.669	0.672	0.675	0.678	0.681	0.684	0.687	0.690	0.694	0.698	0.704	0.710	0.714	0.719	0.724	0.728	0.732	0.735	0.738	0.742	0.745	0.748	0.751	0.754	0.758	0.760
Central Asia	0.551	0.555	0.557	0.558	0.559	0.559	0.560	0.560	0.561	0.563	0.566	0.569	0.574	0.579	0.585	0.591	0.598	0.605	0.611	0.617	0.622	0.627	0.632	0.637	0.642	0.647	0.651	0.655	0.659	0.663
Armenia	0.536	0.541	0.541	0.542	0.544	0.546	0.550	0.554	0.559	0.564	0.570	0.577	0.586	0.596	0.606	0.616	0.626	0.637	0.647	0.652	0.658	0.662	0.666	0.670	0.673	0.676	0.679	0.682	0.686	0.689
Azerbaijan	0.576	0.578	0.579	0.578	0.576	0.573	0.569	0.565	0.561	0.559	0.559	0.561	0.564	0.569	0.575	0.583	0.594	0.607	0.619	0.628	0.637	0.645	0.652	0.658	0.664	0.669	0.673	0.677	0.680	0.683
Georgia	0.654	0.658	0.657	0.654	0.650	0.644	0.638	0.633	0.628	0.625	0.624	0.626	0.630	0.636	0.641	0.646	0.651	0.656	0.660	0.663	0.665	0.668	0.672	0.676	0.680	0.684	0.688	0.693	0.697	0.702
Kazakhstan	0.602	0.606	0.611	0.615	0.619	0.622	0.625	0.628	0.630	0.632	0.635	0.639	0.644	0.649	0.655	0.661	0.667	0.674	0.679	0.683	0.688	0.692	0.696	0.700	0.704	0.708	0.712	0.716	0.720	0.723
Kyrgyzstan	0.532	0.537	0.541	0.543	0.542	0.541	0.539	0.537	0.536	0.534	0.534	0.535	0.537	0.540	0.544	0.546	0.549	0.552	0.555	0.558	0.560	0.563	0.565	0.569	0.574	0.578	0.583	0.588	0.592	0.596
Mongolia	0.465	0.470	0.475	0.480	0.484	0.490	0.495	0.501	0.506	0.512	0.517	0.523	0.528	0.534	0.539	0.545	0.550	0.555	0.560	0.563	0.566	0.570	0.575	0.579	0.584	0.588	0.592	0.597	0.601	0.606
Tajikistan	0.468	0.473	0.474	0.474	0.472	0.468	0.462	0.457	0.451	0.445	0.441	0.440	0.443	0.448	0.456	0.463	0.470	0.477	0.483	0.489	0.495	0.500	0.505	0.511	0.516	0.521	0.526	0.531	0.535	0.539
Turkmenistan	0.548	0.551	0.554	0.557	0.557	0.558	0.558	0.557	0.556	0.557	0.561	0.565	0.570	0.576	0.582	0.588	0.595	0.601	0.606	0.611	0.616	0.622	0.628	0.635	0.642	0.648	0.654	0.660	0.666	0.670
Uzbekistan	0.490	0.492	0.494	0.496	0.498	0.501	0.505	0.510	0.515	0.520	0.525	0.531	0.536	0.541	0.546	0.551	0.556	0.561	0.567	0.572	0.578	0.584	0.590	0.597	0.603	0.609	0.616	0.622	0.627	0.631
Central Europe	0.641	0.647	0.652	0.658	0.665	0.672	0.678	0.683	0.689	0.695	0.702	0.709	0.715	0.720	0.726	0.731	0.736	0.740	0.745	0.750	0.756	0.760	0.764	0.768	0.771	0.775	0.778	0.781	0.785	0.788
Albania	0.540	0.537	0.534	0.533	0.535	0.538	0.544	0.549	0.555	0.561	0.569	0.577	0.585	0.593	0.601	0.608	0.615	0.621	0.627	0.631	0.636	0.640	0.645	0.651	0.658	0.664	0.669	0.674	0.678	0.681
Bosnia and Herzegovina	0.533	0.534	0.532	0.529	0.527	0.528	0.540	0.558	0.576	0.591	0.604	0.616	0.626	0.636	0.644	0.651	0.658	0.665	0.671	0.677	0.682	0.686	0.691	0.695	0.698	0.702	0.706	0.710	0.714	0.718
Bulgaria	0.631	0.641	0.648	0.656	0.666	0.671	0.676	0.681	0.677	0.675	0.680	0.688	0.693	0.697	0.701	0.706	0.710	0.715	0.718	0.724	0.733	0.737	0.740	0.743	0.746	0.750	0.752	0.755	0.760	0.764
Croatia	0.680	0.688	0.692	0.692	0.692	0.691	0.691	0.697	0.703	0.707	0.713	0.719	0.725	0.730	0.734	0.739	0.745	0.748	0.753	0.758	0.763	0.767	0.770	0.774	0.777	0.781	0.784	0.788	0.791	0.794
Czech Republic	0.688	0.696	0.705	0.718	0.736	0.748	0.755	0.760	0.765	0.771	0.776	0.782	0.786	0.790	0.794	0.798	0.801	0.804	0.807	0.810	0.813	0.816	0.818	0.819	0.820	0.820	0.820	0.822	0.825	0.828
Hungary	0.659	0.663	0.671	0.678	0.685	0.693	0.700	0.707	0.713	0.718	0.724	0.730	0.735	0.741	0.746	0.751	0.756	0.760	0.763	0.768	0.772	0.773	0.774	0.774	0.775	0.778	0.781	0.784	0.788	0.791
Montenegro	0.701	0.701	0.699	0.695	0.690	0.687	0.686	0.687	0.690	0.692	0.696	0.701	0.706	0.712	0.717	0.723	0.729	0.736	0.743	0.749	0.754	0.759	0.764	0.768	0.773	0.777	0.780	0.784	0.788	0.791
North Macedonia	0.618	0.620	0.623	0.625	0.627	0.631	0.635	0.640	0.646	0.651	0.656	0.662	0.668	0.674	0.679	0.684	0.689	0.694	0.700	0.704	0.709	0.713	0.717	0.722	0.726	0.730	0.734	0.738	0.741	0.744
Poland	0.632	0.637	0.644	0.653	0.661	0.670	0.677	0.685	0.693	0.701	0.709	0.717	0.724	0.730	0.735	0.740	0.743	0.747	0.752	0.757	0.763	0.770	0.775	0.780	0.784	0.788	0.791	0.795	0.798	0.802
Romania	0.625	0.632	0.635	0.638	0.643	0.649	0.653	0.655	0.659	0.664	0.669	0.677	0.682	0.686	0.693	0.698	0.702	0.707	0.711	0.718	0.726	0.729	0.734	0.740	0.741	0.744	0.747	0.752	0.756	0.760
Serbia	0.626	0.635	0.639	0.639	0.640	0.640	0.644	0.647	0.651	0.657	0.661	0.665	0.670	0.676	0.685	0.694	0.702	0.709	0.716	0.723	0.729	0.735	0.739	0.744	0.748	0.753	0.756	0.760	0.763	0.767
Slovakia	0.656	0.662	0.668	0.679	0.693	0.702	0.709	0.716	0.724	0.731	0.739	0.746	0.752	0.756	0.760	0.766	0.772	0.777	0.781	0.784	0.789	0.794	0.798	0.801	0.803	0.803	0.804	0.805	0.808	0.812
Slovenia	0.726	0.731	0.736	0.741	0.746	0.751	0.756	0.762	0.768	0.774	0.780	0.787	0.793	0.797	0.802	0.807	0.811	0.814	0.818	0.820	0.822	0.824	0.825	0.827	0.829	0.831	0.833	0.835	0.838	0.840
Eastern Europe	0.680	0.687	0.697	0.702	0.702	0.705	0.707	0.708	0.709	0.711	0.711	0.713	0.716	0.720	0.727	0.734	0.740	0.745	0.751	0.757	0.762	0.765	0.768	0.772	0.777	0.781	0.785	0.788	0.791	0.793
Belarus	0.591	0.595	0.600	0.606	0.611	0.614	0.618	0.620	0.621	0.624	0.629	0.635	0.642	0.650	0.658	0.665	0.671	0.678	0.687	0.695	0.703	0.709	0.713	0.719	0.725	0.730	0.734	0.738	0.742	0.745
Estonia	0.665	0.676	0.687	0.696	0.700	0.705	0.711	0.717	0.721	0.726	0.733	0.741	0.748	0.753	0.759	0.765	0.771	0.777	0.785	0.792	0.798	0.804	0.809	0.813	0.817	0.821	0.825	0.829	0.833	0.835
Latvia	0.675	0.682	0.691	0.700	0.708	0.713	0.716	0.719	0.721	0.723	0.727	0.733	0.739	0.745	0.753	0.760	0.766	0.774	0.784	0.793	0.797	0.798	0.801	0.803	0.804	0.805	0.809	0.813	0.817	0.820
Lithuania	0.670	0.672	0.682	0.691	0.694	0.696	0.700	0.705	0.709	0.714	0.723	0.730	0.736	0.743	0.752	0.760	0.765	0.771	0.782	0.792	0.797	0.801	0.808	0.813	0.817	0.822	0.829	0.835	0.839	0.843
Moldova	0.585	0.589	0.591	0.594	0.594	0.595	0.594	0.593	0.591	0.587	0.585	0.585	0.588	0.593	0.600	0.607	0.615	0.622	0.630	0.637	0.644	0.651	0.658	0.665	0.672	0.677	0.683	0.688	0.693	0.696

Socio- Demographic Index values for all estimated GBD 2019 locations, 1990-2019

Location	1990	1991	1992	1993	1994	1995	1996	1997	1998	1999	2000	2001	2002	2003	2004	2005	2006	2007	2008	2009	2010	2011	2012	2013	2014	2015	2016	2017	2018	2019
Russia	0.695	0.703	0.716	0.720	0.719	0.722	0.724	0.725	0.726	0.728	0.728	0.728	0.730	0.734	0.741	0.749	0.754	0.759	0.764	0.770	0.775	0.777	0.779	0.784	0.788	0.793	0.797	0.801	0.803	0.805
Ukraine	0.653	0.657	0.661	0.665	0.666	0.667	0.667	0.667	0.666	0.665	0.664	0.665	0.668	0.672	0.679	0.685	0.692	0.699	0.706	0.710	0.713	0.718	0.721	0.725	0.727	0.729	0.730	0.732	0.734	0.736
High income	0.755	0.760	0.765	0.769	0.773	0.777	0.780	0.783	0.786	0.788	0.791	0.795	0.799	0.801	0.804	0.806	0.807	0.809	0.812	0.816	0.820	0.823	0.826	0.829	0.832	0.835	0.839	0.842	0.845	0.847
Australasia	0.742	0.746	0.749	0.753	0.757	0.761	0.765	0.769	0.773	0.777	0.781	0.785	0.789	0.793	0.797	0.799	0.799	0.800	0.803	0.807	0.810	0.812	0.816	0.821	0.825	0.828	0.832	0.835	0.837	0.840
Australia	0.738	0.741	0.745	0.749	0.753	0.757	0.761	0.766	0.770	0.774	0.778	0.782	0.787	0.791	0.795	0.797	0.798	0.799	0.802	0.806	0.809	0.812	0.815	0.820	0.824	0.828	0.832	0.834	0.837	0.839
New Zealand	0.757	0.762	0.765	0.769	0.772	0.774	0.778	0.782	0.785	0.787	0.790	0.794	0.796	0.798	0.802	0.803	0.800	0.800	0.803	0.807	0.809	0.812	0.816	0.821	0.825	0.828	0.832	0.835	0.838	0.840
High- income Asia Pacific	0.767	0.773	0.779	0.785	0.790	0.796	0.801	0.805	0.809	0.813	0.816	0.819	0.823	0.826	0.830	0.833	0.836	0.839	0.842	0.844	0.847	0.850	0.853	0.856	0.859	0.862	0.865	0.868	0.871	0.873
Brunei	0.676	0.682	0.688	0.694	0.700	0.706	0.712	0.717	0.723	0.729	0.735	0.741	0.747	0.753	0.758	0.764	0.769	0.774	0.779	0.784	0.789	0.793	0.797	0.801	0.806	0.809	0.813	0.817	0.820	0.823
Japan	0.791	0.796	0.801	0.805	0.809	0.813	0.817	0.820	0.822	0.824	0.826	0.828	0.830	0.833	0.836	0.838	0.840	0.842	0.844	0.846	0.848	0.850	0.853	0.855	0.857	0.860	0.862	0.865	0.867	0.870
South Korea																														
Singapore	0.688	0.697	0.705	0.714	0.723	0.731	0.740	0.749	0.756	0.762	0.769	0.776	0.783	0.789	0.794	0.801	0.808	0.814	0.822	0.828	0.835	0.839	0.843	0.847	0.850	0.852	0.855	0.858	0.860	0.861
High- income North America	0.771	0.773	0.777	0.780	0.784	0.787	0.790	0.792	0.794	0.797	0.800	0.805	0.809	0.811	0.814	0.815	0.814	0.817	0.822	0.828	0.834	0.837	0.841	0.844	0.847	0.850	0.854	0.857	0.859	0.860
Canada	0.790	0.792	0.795	0.797	0.800	0.804	0.809	0.812	0.815	0.819	0.824	0.828	0.832	0.835	0.838	0.840	0.842	0.843	0.845	0.848	0.851	0.853	0.856	0.859	0.861	0.864	0.867	0.869	0.871	0.873
Greenland	0.655	0.652	0.651	0.651	0.653	0.653	0.653	0.655	0.661	0.664	0.667	0.671	0.678	0.684	0.689	0.696	0.704	0.710	0.715	0.721	0.728	0.734	0.737	0.740	0.743	0.747	0.751	0.756	0.759	0.761
USA	0.768	0.771	0.775	0.778	0.782	0.785	0.788	0.789	0.791	0.794	0.797	0.802	0.806	0.809	0.811	0.812	0.811	0.814	0.819	0.826	0.832	0.835	0.839	0.842	0.845	0.849	0.853	0.856	0.858	0.859
Southern Latin America	0.584	0.589	0.597	0.602	0.608	0.614	0.620	0.625	0.630	0.634	0.640	0.644	0.648	0.651	0.653	0.658	0.663	0.664	0.667	0.671	0.676	0.681	0.686	0.689	0.692	0.701	0.710	0.716	0.719	0.721
Argentina	0.581	0.585	0.593	0.599	0.605	0.611	0.617	0.622	0.625	0.628	0.634	0.637	0.640	0.641	0.642	0.649	0.653	0.655	0.657	0.661	0.665	0.670	0.674	0.677	0.679	0.687	0.696	0.702	0.706	0.708
Chile	0.592	0.600	0.606	0.611	0.617	0.624	0.630	0.637	0.644	0.651	0.657	0.663	0.671	0.678	0.683	0.686	0.689	0.692	0.695	0.700	0.706	0.712	0.719	0.724	0.728	0.738	0.747	0.753	0.756	0.759
Uruguay	0.581	0.584	0.588	0.591	0.594	0.597	0.600	0.606	0.612	0.618	0.622	0.626	0.628	0.631	0.633	0.636	0.639	0.642	0.645	0.649	0.653	0.658	0.663	0.668	0.673	0.678	0.684	0.688	0.693	0.697
Western Europe	0.750	0.756	0.762	0.767	0.772	0.775	0.779	0.782	0.784	0.787	0.790	0.794	0.797	0.800	0.802	0.805	0.807	0.810	0.812	0.815	0.817	0.821	0.824	0.827	0.830	0.832	0.835	0.838	0.841	0.843
Andorra	0.834	0.838	0.840	0.841	0.841	0.841	0.843	0.845	0.847	0.849	0.851	0.854	0.855	0.859	0.862	0.865	0.867	0.869	0.872	0.874	0.876	0.879	0.881	0.883	0.885	0.887	0.889	0.891	0.892	0.894
Austria	0.753	0.754	0.757	0.761	0.768	0.773	0.778	0.783	0.787	0.791	0.795	0.799	0.803	0.805	0.808	0.811	0.815	0.818	0.821	0.824	0.826	0.830	0.833	0.835	0.838	0.839	0.841	0.844	0.847	0.849
Belgium	0.746	0.750	0.756	0.762	0.767	0.771	0.775	0.779	0.782	0.784	0.787	0.792	0.796	0.799	0.802	0.805	0.808	0.810	0.813	0.816	0.820	0.824	0.829	0.834	0.837	0.841	0.843	0.846	0.849	0.851
Cyprus	0.662	0.670	0.680	0.691	0.702	0.713	0.723	0.732	0.741	0.750	0.758	0.767	0.774	0.780	0.786	0.791	0.797	0.804	0.810	0.816	0.820	0.824	0.827	0.829	0.831	0.832	0.834	0.836	0.838	0.841
Denmark	0.806	0.809	0.813	0.816	0.820	0.824	0.828	0.833	0.836	0.840	0.844	0.848	0.852	0.855	0.858	0.860	0.862	0.864	0.865	0.867	0.870	0.873	0.875	0.878	0.880	0.882	0.884	0.886	0.888	0.890
Finland	0.757	0.759	0.762	0.765	0.769	0.773	0.777	0.782	0.785	0.788	0.792	0.797	0.802	0.805	0.808	0.812	0.815	0.818	0.821	0.824	0.828	0.831	0.834	0.837	0.840	0.844	0.848	0.851	0.853	0.856
France	0.738	0.743	0.750	0.755	0.759	0.763	0.767	0.770	0.773	0.775	0.777	0.781	0.785	0.787	0.790	0.793	0.796	0.799	0.801	0.803	0.806	0.809	0.812	0.815	0.819	0.822	0.826	0.829	0.832	0.834
Germany	0.819	0.830	0.834	0.838	0.841	0.843	0.844	0.844	0.844	0.844	0.847	0.853	0.856	0.858	0.861	0.863	0.866	0.869	0.873	0.875	0.878	0.881	0.883	0.886	0.888	0.890	0.892	0.894	0.896	0.898
Greece	0.682	0.688	0.695	0.702	0.707	0.713	0.717	0.723	0.729	0.735	0.740	0.743	0.748	0.753	0.758	0.761	0.765	0.768	0.771	0.775	0.779	0.782	0.785	0.786	0.786	0.787	0.788	0.790	0.792	0.794
Iceland	0.764	0.770	0.774	0.778	0.782	0.785	0.788	0.790	0.794	0.799	0.806	0.813	0.818	0.822	0.824	0.827	0.830	0.834	0.838	0.842	0.846	0.847	0.847	0.848	0.850	0.854	0.858	0.863	0.866	0.869
Ireland	0.730	0.735	0.741	0.747	0.753	0.758	0.763	0.768	0.774	0.780	0.786	0.793	0.799	0.806	0.812	0.816	0.819	0.821	0.824	0.827	0.831	0.835	0.839	0.842	0.845	0.850	0.854	0.859	0.864	0.867
Israel	0.717	0.721	0.726	0.730	0.734	0.738	0.742	0.745	0.749	0.752	0.756	0.760	0.762	0.765	0.769	0.773	0.776	0.778	0.778	0.779	0.781	0.784	0.787	0.790	0.792	0.794	0.796	0.798	0.800	0.803
Italy	0.712	0.717	0.722	0.727	0.732	0.737	0.740	0.744	0.747	0.750	0.753	0.757	0.761	0.763	0.766	0.768	0.771	0.773	0.775	0.777	0.780	0.782	0.784	0.787	0.789	0.791	0.794	0.796	0.798	0.801
Luxembourg	0.815	0.818	0.820	0.823	0.828	0.833	0.836	0.839	0.842	0.844	0.847	0.850	0.853	0.855	0.857	0.858	0.862	0.866	0.869	0.871	0.872	0.874	0.877	0.880	0.883	0.886	0.889	0.892	0.894	0.895

Socio- Demographic Index values for all estimated GBD 2019 locations, 1990-2019																														
**Location**	**1990**	**1991**	**1992**	**1993**	**1994**	**1995**	**1996**	**1997**	**1998**	**1999**	**2000**	**2001**	**2002**	**2003**	**2004**	**2005**	**2006**	**2007**	**2008**	**2009**	**2010**	**2011**	**2012**	**2013**	**2014**	**2015**	**2016**	**2017**	**2018**	**2019**
Malta	0.666	0.670	0.675	0.682	0.690	0.695	0.696	0.700	0.708	0.715	0.722	0.729	0.733	0.737	0.741	0.745	0.749	0.753	0.757	0.761	0.764	0.768	0.772	0.775	0.779	0.784	0.788	0.793	0.797	0.801
Monaco	0.834	0.837	0.840	0.843	0.846	0.849	0.852	0.855	0.857	0.860	0.862	0.865	0.867	0.870	0.872	0.875	0.877	0.879	0.881	0.883	0.886	0.888	0.890	0.892	0.893	0.895	0.897	0.899	0.901	0.902
Netherlands	0.796	0.801	0.806	0.810	0.814	0.818	0.821	0.824	0.827	0.830	0.832	0.836	0.839	0.842	0.845	0.848	0.851	0.853	0.856	0.858	0.861	0.864	0.866	0.869	0.871	0.874	0.876	0.878	0.881	0.883
Norway	0.807	0.812	0.818	0.823	0.828	0.832	0.837	0.843	0.847	0.851	0.856	0.861	0.866	0.869	0.872	0.874	0.876	0.877	0.879	0.881	0.885	0.889	0.893	0.896	0.900	0.903	0.907	0.910	0.912	0.913
Portugal	0.607	0.615	0.622	0.629	0.636	0.641	0.647	0.651	0.656	0.661	0.666	0.671	0.676	0.681	0.686	0.690	0.694	0.698	0.701	0.705	0.709	0.714	0.718	0.722	0.726	0.729	0.732	0.736	0.739	0.743
San Marino	0.814	0.817	0.820	0.824	0.828	0.832	0.837	0.841	0.846	0.850	0.852	0.854	0.856	0.859	0.861	0.862	0.864	0.866	0.868	0.870	0.872	0.874	0.876	0.877	0.879	0.881	0.882	0.882	0.883	0.884
Spain	0.647	0.655	0.662	0.669	0.675	0.681	0.686	0.691	0.696	0.700	0.705	0.709	0.713	0.717	0.721	0.725	0.728	0.730	0.734	0.739	0.743	0.746	0.749	0.752	0.754	0.756	0.759	0.761	0.764	0.767
Sweden	0.769	0.775	0.782	0.788	0.794	0.801	0.806	0.811	0.815	0.819	0.823	0.826	0.829	0.832	0.835	0.838	0.840	0.842	0.844	0.846	0.849	0.852	0.855	0.858	0.860	0.863	0.865	0.868	0.870	0.872
Switzerland	0.868	0.869	0.873	0.876	0.878	0.880	0.881	0.883	0.884	0.886	0.889	0.893	0.894	0.896	0.898	0.900	0.902	0.905	0.908	0.909	0.912	0.914	0.917	0.919	0.921	0.922	0.924	0.926	0.928	0.929
UK	0.745	0.749	0.755	0.761	0.766	0.769	0.771	0.774	0.778	0.784	0.789	0.793	0.797	0.799	0.802	0.804	0.806	0.808	0.811	0.813	0.816	0.820	0.825	0.830	0.834	0.837	0.839	0.842	0.845	0.847
Latin America and Caribbean	0.491	0.496	0.501	0.506	0.511	0.516	0.521	0.526	0.531	0.536	0.541	0.546	0.550	0.554	0.558	0.563	0.568	0.573	0.578	0.583	0.589	0.594	0.600	0.605	0.611	0.616	0.620	0.625	0.629	0.633
Andean Latin America	0.489	0.492	0.496	0.500	0.504	0.509	0.514	0.519	0.524	0.529	0.534	0.538	0.543	0.548	0.553	0.558	0.564	0.569	0.575	0.580	0.585	0.591	0.596	0.602	0.608	0.613	0.618	0.624	0.628	0.632
Bolivia	0.412	0.417	0.422	0.428	0.434	0.441	0.448	0.455	0.462	0.469	0.474	0.480	0.485	0.491	0.496	0.501	0.506	0.510	0.514	0.518	0.521	0.525	0.528	0.533	0.538	0.544	0.550	0.556	0.562	0.566
Ecuador	0.503	0.506	0.510	0.514	0.517	0.521	0.524	0.528	0.532	0.535	0.539	0.544	0.549	0.554	0.559	0.565	0.570	0.575	0.581	0.586	0.591	0.597	0.603	0.609	0.615	0.621	0.626	0.632	0.636	0.640
Peru	0.501	0.505	0.508	0.511	0.516	0.521	0.526	0.532	0.537	0.543	0.548	0.552	0.557	0.562	0.566	0.571	0.577	0.583	0.589	0.595	0.601	0.608	0.614	0.620	0.625	0.630	0.635	0.640	0.645	0.648
Caribbean	0.517	0.522	0.525	0.528	0.531	0.533	0.536	0.539	0.542	0.547	0.552	0.557	0.563	0.569	0.573	0.579	0.585	0.588	0.591	0.594	0.598	0.601	0.605	0.609	0.612	0.616	0.621	0.625	0.628	0.631
Antigua and Barbuda	0.579	0.586	0.592	0.598	0.604	0.610	0.616	0.623	0.631	0.638	0.645	0.652	0.659	0.665	0.672	0.679	0.686	0.694	0.700	0.705	0.709	0.713	0.716	0.719	0.723	0.727	0.731	0.735	0.739	0.743
The Bahamas	0.692	0.684	0.676	0.679	0.686	0.692	0.697	0.702	0.710	0.721	0.731	0.735	0.737	0.739	0.742	0.749	0.755	0.759	0.762	0.767	0.774	0.779	0.780	0.782	0.784	0.786	0.789	0.791	0.794	0.796
Barbados	0.649	0.653	0.658	0.663	0.667	0.670	0.672	0.674	0.676	0.677	0.680	0.683	0.687	0.691	0.695	0.699	0.703	0.707	0.711	0.714	0.718	0.721	0.725	0.728	0.730	0.733	0.735	0.737	0.740	0.742
Belize	0.428	0.437	0.447	0.457	0.466	0.475	0.482	0.488	0.494	0.499	0.505	0.510	0.515	0.521	0.528	0.534	0.541	0.547	0.553	0.559	0.564	0.569	0.574	0.579	0.583	0.588	0.592	0.596	0.600	0.603
Bermuda	0.685	0.689	0.693	0.697	0.701	0.704	0.707	0.710	0.714	0.719	0.724	0.731	0.737	0.743	0.749	0.755	0.760	0.766	0.773	0.779	0.785	0.790	0.795	0.799	0.802	0.805	0.807	0.809	0.811	0.813
Cuba	0.578	0.586	0.589	0.588	0.586	0.583	0.583	0.582	0.579	0.581	0.586	0.588	0.595	0.602	0.605	0.611	0.618	0.618	0.616	0.618	0.620	0.624	0.631	0.636	0.640	0.645	0.653	0.660	0.665	0.668
Dominica	0.579	0.585	0.591	0.597	0.604	0.610	0.617	0.623	0.630	0.637	0.643	0.649	0.655	0.660	0.666	0.672	0.677	0.683	0.689	0.695	0.700	0.705	0.709	0.713	0.717	0.721	0.724	0.727	0.728	0.729
Dominican Republic	0.425	0.427	0.430	0.434	0.437	0.442	0.446	0.452	0.458	0.465	0.471	0.478	0.485	0.492	0.499	0.508	0.516	0.524	0.532	0.538	0.544	0.549	0.554	0.559	0.563	0.569	0.574	0.580	0.587	0.592
Grenada	0.463	0.474	0.485	0.495	0.505	0.515	0.524	0.533	0.543	0.553	0.562	0.570	0.577	0.585	0.592	0.600	0.606	0.613	0.619	0.624	0.628	0.633	0.637	0.641	0.645	0.650	0.654	0.659	0.664	0.669
Guyana	0.452	0.456	0.461	0.467	0.474	0.481	0.488	0.496	0.503	0.510	0.516	0.522	0.527	0.532	0.536	0.540	0.544	0.549	0.554	0.559	0.565	0.571	0.577	0.583	0.590	0.596	0.602	0.608	0.614	0.618
Haiti	0.307	0.311	0.314	0.317	0.320	0.323	0.328	0.333	0.338	0.344	0.350	0.356	0.361	0.366	0.371	0.375	0.379	0.383	0.387	0.392	0.395	0.399	0.403	0.407	0.412	0.416	0.420	0.424	0.428	0.432
Jamaica	0.542	0.547	0.553	0.560	0.566	0.573	0.580	0.586	0.592	0.598	0.603	0.609	0.614	0.620	0.625	0.630	0.635	0.640	0.645	0.649	0.653	0.657	0.661	0.664	0.668	0.671	0.675	0.678	0.681	0.684
Puerto Rico	0.670	0.676	0.679	0.684	0.689	0.693	0.699	0.705	0.711	0.714	0.720	0.731	0.738	0.742	0.744	0.748	0.752	0.756	0.760	0.764	0.769	0.774	0.779	0.785	0.793	0.802	0.808	0.811	0.813	0.814
Saint Kitts and Nevis	0.583	0.592	0.599	0.607	0.614	0.620	0.626	0.633	0.638	0.644	0.650	0.657	0.663	0.668	0.673	0.679	0.685	0.690	0.696	0.701	0.706	0.710	0.714	0.719	0.724	0.728	0.733	0.738	0.742	0.746
Saint Lucia	0.483	0.493	0.504	0.514	0.524	0.534	0.543	0.551	0.560	0.568	0.575	0.581	0.587	0.593	0.600	0.606	0.612	0.617	0.623	0.629	0.634	0.639	0.643	0.648	0.652	0.656	0.659	0.663	0.667	0.670
Saint Vincent and the Grenadines	0.462	0.471	0.479	0.487	0.495	0.502	0.509	0.515	0.522	0.528	0.534	0.539	0.545	0.551	0.557	0.562	0.568	0.574	0.580	0.585	0.589	0.593	0.598	0.602	0.606	0.610	0.615	0.619	0.623	0.627
Suriname	0.498	0.503	0.507	0.511	0.513	0.515	0.518	0.523	0.528	0.533	0.538	0.545	0.552	0.560	0.568	0.574	0.579	0.584	0.588	0.593	0.598	0.602	0.607	0.611	0.616	0.620	0.625	0.629	0.633	0.636
Trinidad and Tobago	0.618	0.622	0.626	0.630	0.634	0.639	0.644	0.650	0.656	0.662	0.669	0.675	0.682	0.689	0.696	0.704	0.711	0.718	0.724	0.728	0.732	0.736	0.739	0.742	0.745	0.748	0.751	0.753	0.755	0.757

Socio- Demographic Index values for all estimated GBD 2019 locations, 1990-2019

Location	1990	1991	1992	1993	1994	1995	1996	1997	1998	1999	2000	2001	2002	2003	2004	2005	2006	2007	2008	2009	2010	2011	2012	2013	2014	2015	2016	2017	2018	2019
Virgin Islands	0.667	0.680	0.689	0.697	0.704	0.710	0.715	0.720	0.724	0.728	0.731	0.734	0.742	0.749	0.756	0.762	0.768	0.773	0.778	0.782	0.785	0.788	0.790	0.791	0.792	0.794	0.795	0.796	0.798	0.799
Central Latin America	0.485	0.490	0.495	0.501	0.507	0.512	0.517	0.523	0.528	0.533	0.538	0.543	0.547	0.551	0.554	0.559	0.564	0.569	0.575	0.579	0.584	0.589	0.594	0.599	0.604	0.609	0.614	0.618	0.623	0.626
Colombia	0.478	0.480	0.482	0.486	0.492	0.498	0.503	0.509	0.514	0.518	0.522	0.526	0.529	0.533	0.536	0.540	0.546	0.552	0.559	0.567	0.574	0.582	0.589	0.596	0.603	0.609	0.616	0.622	0.628	0.633
Costa Rica	0.532	0.538	0.544	0.550	0.555	0.560	0.565	0.571	0.577	0.584	0.592	0.598	0.603	0.607	0.611	0.615	0.619	0.623	0.627	0.632	0.637	0.642	0.647	0.652	0.657	0.662	0.667	0.672	0.676	0.680
El Salvador	0.390	0.393	0.397	0.402	0.408	0.415	0.423	0.431	0.441	0.450	0.460	0.469	0.478	0.486	0.493	0.499	0.505	0.511	0.517	0.521	0.526	0.531	0.536	0.542	0.547	0.552	0.558	0.563	0.568	0.573
Guatemala	0.315	0.320	0.328	0.336	0.345	0.352	0.360	0.369	0.378	0.387	0.395	0.403	0.412	0.421	0.430	0.439	0.446	0.453	0.459	0.465	0.472	0.478	0.485	0.491	0.498	0.504	0.510	0.516	0.522	0.526
Honduras	0.330	0.335	0.340	0.345	0.351	0.357	0.363	0.370	0.377	0.384	0.391	0.397	0.404	0.411	0.417	0.424	0.430	0.437	0.443	0.448	0.454	0.459	0.464	0.469	0.473	0.478	0.482	0.487	0.492	0.496
Mexico	0.507	0.514	0.520	0.526	0.532	0.537	0.542	0.547	0.553	0.558	0.563	0.569	0.574	0.578	0.583	0.588	0.592	0.597	0.601	0.605	0.608	0.613	0.617	0.621	0.626	0.631	0.636	0.640	0.645	0.649
Nicaragua	0.338	0.345	0.353	0.360	0.368	0.376	0.385	0.393	0.402	0.411	0.419	0.426	0.433	0.438	0.444	0.449	0.453	0.458	0.462	0.466	0.470	0.474	0.479	0.484	0.489	0.495	0.500	0.506	0.512	0.517
Panama	0.544	0.549	0.555	0.559	0.562	0.565	0.568	0.573	0.579	0.586	0.592	0.597	0.602	0.605	0.607	0.610	0.613	0.616	0.620	0.623	0.627	0.630	0.635	0.642	0.650	0.658	0.666	0.674	0.680	0.686
Venezuela	0.509	0.514	0.522	0.530	0.537	0.542	0.546	0.551	0.554	0.557	0.559	0.560	0.558	0.552	0.548	0.550	0.557	0.566	0.575	0.582	0.586	0.591	0.596	0.600	0.604	0.607	0.608	0.608	0.608	0.607
Tropical Latin America	0.487	0.492	0.497	0.503	0.508	0.513	0.519	0.524	0.529	0.533	0.538	0.543	0.547	0.552	0.556	0.561	0.566	0.572	0.578	0.584	0.590	0.597	0.604	0.610	0.617	0.622	0.627	0.632	0.636	0.640
Brazil	0.487	0.492	0.498	0.503	0.508	0.513	0.519	0.524	0.529	0.533	0.538	0.543	0.547	0.551	0.556	0.561	0.566	0.572	0.577	0.583	0.590	0.597	0.603	0.610	0.616	0.622	0.627	0.632	0.636	0.640
Paraguay	0.465	0.471	0.477	0.484	0.491	0.498	0.505	0.512	0.519	0.524	0.529	0.534	0.539	0.544	0.549	0.554	0.559	0.564	0.570	0.575	0.582	0.588	0.594	0.601	0.608	0.615	0.621	0.627	0.633	0.638
North Africa and Middle East	0.414	0.423	0.432	0.441	0.450	0.460	0.469	0.478	0.487	0.497	0.506	0.515	0.524	0.532	0.541	0.550	0.560	0.569	0.578	0.587	0.595	0.603	0.611	0.619	0.626	0.633	0.640	0.647	0.654	0.660
North Africa and Middle East	0.414	0.423	0.432	0.441	0.450	0.460	0.469	0.478	0.487	0.497	0.506	0.515	0.524	0.532	0.541	0.550	0.560	0.569	0.578	0.587	0.595	0.603	0.611	0.619	0.626	0.633	0.640	0.647	0.654	0.660
Afghanistan	0.187	0.191	0.195	0.196	0.194	0.194	0.193	0.192	0.190	0.189	0.188	0.188	0.194	0.202	0.209	0.216	0.224	0.234	0.243	0.253	0.264	0.274	0.285	0.295	0.304	0.313	0.321	0.329	0.337	0.343
Algeria	0.436	0.446	0.456	0.465	0.474	0.483	0.492	0.500	0.509	0.518	0.526	0.534	0.542	0.550	0.558	0.566	0.573	0.580	0.587	0.593	0.599	0.605	0.611	0.617	0.623	0.628	0.634	0.640	0.646	0.652
Bahrain	0.553	0.560	0.566	0.573	0.580	0.587	0.595	0.602	0.610	0.618	0.626	0.634	0.643	0.653	0.662	0.672	0.680	0.688	0.697	0.705	0.711	0.715	0.720	0.726	0.731	0.735	0.739	0.743	0.747	0.751
Egypt	0.403	0.415	0.426	0.437	0.448	0.459	0.469	0.478	0.487	0.496	0.504	0.512	0.520	0.527	0.535	0.542	0.549	0.557	0.565	0.573	0.582	0.591	0.600	0.609	0.617	0.626	0.635	0.643	0.651	0.658
Iran	0.404	0.419	0.433	0.446	0.457	0.468	0.477	0.486	0.496	0.506	0.517	0.529	0.541	0.552	0.562	0.571	0.581	0.593	0.603	0.613	0.622	0.630	0.635	0.640	0.645	0.649	0.654	0.659	0.665	0.670
Iraq	0.392	0.397	0.402	0.407	0.412	0.417	0.422	0.429	0.439	0.451	0.462	0.475	0.485	0.493	0.504	0.514	0.525	0.535	0.547	0.558	0.570	0.583	0.597	0.610	0.622	0.632	0.644	0.654	0.663	0.671
Jordan	0.520	0.529	0.537	0.546	0.554	0.562	0.570	0.577	0.585	0.592	0.600	0.607	0.614	0.621	0.630	0.639	0.648	0.656	0.664	0.673	0.681	0.688	0.695	0.702	0.707	0.713	0.718	0.723	0.727	0.731
Kuwait	0.655	0.659	0.662	0.667	0.673	0.680	0.689	0.699	0.709	0.717	0.724	0.729	0.735	0.742	0.750	0.760	0.769	0.777	0.785	0.793	0.801	0.808	0.815	0.822	0.828	0.834	0.839	0.844	0.848	0.851
Lebanon	0.462	0.470	0.477	0.485	0.493	0.502	0.511	0.520	0.530	0.540	0.548	0.557	0.565	0.574	0.582	0.591	0.600	0.609	0.618	0.628	0.639	0.649	0.660	0.670	0.677	0.685	0.691	0.698	0.704	0.708
Libya	0.405	0.422	0.438	0.455	0.472	0.489	0.506	0.522	0.538	0.552	0.566	0.580	0.593	0.606	0.619	0.632	0.645	0.658	0.670	0.681	0.691	0.695	0.703	0.707	0.707	0.707	0.705	0.705	0.707	0.709
Morocco	0.347	0.354	0.361	0.367	0.374	0.380	0.386	0.392	0.398	0.403	0.409	0.414	0.420	0.426	0.432	0.439	0.445	0.452	0.460	0.467	0.475	0.483	0.491	0.499	0.508	0.516	0.524	0.533	0.541	0.548
Oman	0.441	0.455	0.469	0.485	0.500	0.514	0.528	0.543	0.558	0.573	0.588	0.601	0.615	0.628	0.641	0.653	0.664	0.678	0.692	0.704	0.715	0.726	0.737	0.747	0.754	0.760	0.767	0.773	0.778	0.783
Palestine	0.314	0.320	0.329	0.337	0.347	0.356	0.365	0.375	0.386	0.397	0.407	0.415	0.422	0.430	0.439	0.449	0.458	0.467	0.476	0.486	0.497	0.509	0.521	0.533	0.543	0.553	0.564	0.573	0.582	0.588
Qatar	0.585	0.598	0.610	0.621	0.632	0.643	0.654	0.666	0.676	0.685	0.694	0.703	0.711	0.719	0.727	0.735	0.743	0.750	0.757	0.764	0.772	0.779	0.786	0.793	0.799	0.806	0.812	0.818	0.825	0.830
Saudi Arabia	0.480	0.491	0.504	0.516	0.529	0.541	0.554	0.566	0.578	0.590	0.602	0.614	0.625	0.637	0.650	0.664	0.677	0.690	0.703	0.715	0.726	0.738	0.750	0.760	0.769	0.778	0.786	0.793	0.800	0.805
Sudan	0.227	0.234	0.241	0.248	0.256	0.263	0.271	0.280	0.289	0.298	0.308	0.318	0.327	0.337	0.348	0.358	0.370	0.381	0.393	0.404	0.416	0.428	0.440	0.451	0.462	0.474	0.485	0.497	0.507	0.515
Syria	0.367	0.376	0.387	0.398	0.409	0.421	0.432	0.443	0.454	0.465	0.476	0.486	0.497	0.508	0.521	0.535	0.548	0.560	0.572	0.583	0.594	0.601	0.605	0.606	0.607	0.608	0.610	0.613	0.616	0.619
Tunisia	0.434	0.444	0.455	0.466	0.476	0.487	0.498	0.508	0.518	0.528	0.538	0.548	0.556	0.565	0.574	0.582	0.591	0.599	0.607	0.614	0.622	0.628	0.634	0.640	0.646	0.651	0.657	0.662	0.667	0.672

Socio- Demographic Index values for all estimated GBD 2019 locations, 1990-2019																														
Location	1990	1991	1992	1993	1994	1995	1996	1997	1998	1999	2000	2001	2002	2003	2004	2005	2006	2007	2008	2009	2010	2011	2012	2013	2014	2015	2016	2017	2018	2019
Turkey	0.473	0.483	0.493	0.504	0.513	0.523	0.534	0.545	0.556	0.566	0.577	0.587	0.597	0.607	0.619	0.630	0.641	0.652	0.663	0.671	0.680	0.689	0.698	0.707	0.715	0.723	0.729	0.736	0.743	0.748
United Arab Emirates	0.621	0.637	0.653	0.669	0.685	0.700	0.714	0.727	0.740	0.751	0.762	0.773	0.783	0.792	0.801	0.810	0.819	0.829	0.838	0.846	0.853	0.859	0.863	0.868	0.870	0.872	0.874	0.876	0.879	0.880
Yemen	0.176	0.183	0.191	0.198	0.207	0.215	0.224	0.234	0.243	0.253	0.263	0.273	0.283	0.293	0.303	0.314	0.325	0.335	0.346	0.356	0.366	0.375	0.384	0.393	0.402	0.407	0.410	0.412	0.413	0.412
South Asia	0.313	0.319	0.325	0.331	0.337	0.344	0.351	0.357	0.364	0.371	0.378	0.385	0.391	0.398	0.405	0.413	0.421	0.430	0.438	0.447	0.456	0.465	0.475	0.485	0.495	0.505	0.515	0.525	0.535	0.543
South Asia	0.313	0.319	0.325	0.331	0.337	0.344	0.351	0.357	0.364	0.371	0.378	0.385	0.391	0.398	0.405	0.413	0.421	0.430	0.438	0.447	0.456	0.465	0.475	0.485	0.495	0.505	0.515	0.525	0.535	0.543
Bangladesh	0.267	0.275	0.283	0.290	0.297	0.304	0.311	0.317	0.324	0.330	0.336	0.343	0.349	0.355	0.362	0.369	0.376	0.384	0.392	0.400	0.408	0.416	0.425	0.433	0.441	0.449	0.457	0.466	0.475	0.483
Bhutan	0.228	0.232	0.237	0.243	0.251	0.258	0.266	0.275	0.282	0.290	0.298	0.306	0.314	0.322	0.330	0.338	0.347	0.356	0.365	0.375	0.384	0.394	0.403	0.411	0.419	0.426	0.434	0.442	0.449	0.455
India	0.327	0.333	0.339	0.345	0.351	0.358	0.364	0.371	0.378	0.386	0.393	0.400	0.407	0.414	0.421	0.429	0.437	0.446	0.455	0.463	0.473	0.483	0.493	0.504	0.515	0.526	0.537	0.547	0.558	0.566
Nepal	0.198	0.203	0.208	0.215	0.221	0.228	0.236	0.244	0.251	0.259	0.267	0.276	0.284	0.291	0.299	0.307	0.315	0.322	0.330	0.339	0.347	0.356	0.365	0.373	0.382	0.391	0.399	0.408	0.416	0.422
Pakistan	0.247	0.253	0.259	0.265	0.271	0.277	0.283	0.289	0.295	0.301	0.307	0.313	0.320	0.326	0.333	0.340	0.347	0.355	0.363	0.371	0.379	0.387	0.394	0.402	0.410	0.418	0.426	0.434	0.442	0.449
Southeast Asia, east Asia, and Oceania	0.452	0.460	0.468	0.476	0.485	0.494	0.503	0.511	0.519	0.526	0.533	0.541	0.548	0.556	0.563	0.571	0.580	0.589	0.597	0.605	0.614	0.622	0.629	0.636	0.643	0.647	0.651	0.659	0.667	0.673
East Asia	0.447	0.456	0.464	0.473	0.483	0.492	0.502	0.511	0.520	0.528	0.537	0.545	0.554	0.562	0.571	0.580	0.590	0.600	0.609	0.618	0.628	0.637	0.644	0.652	0.660	0.662	0.665	0.675	0.684	0.691
China	0.433	0.441	0.450	0.459	0.469	0.479	0.489	0.499	0.508	0.516	0.525	0.534	0.543	0.552	0.561	0.571	0.581	0.591	0.601	0.611	0.621	0.631	0.638	0.646	0.654	0.657	0.659	0.669	0.679	0.686
North Korea	0.431	0.436	0.439	0.442	0.445	0.447	0.450	0.451	0.452	0.455	0.458	0.463	0.467	0.473	0.478	0.485	0.490	0.496	0.502	0.507	0.513	0.518	0.523	0.528	0.534	0.538	0.543	0.548	0.553	0.558
Taiwan (province of China)	0.667	0.678	0.685	0.694	0.702	0.711	0.719	0.731	0.743	0.747	0.754	0.763	0.772	0.779	0.787	0.795	0.802	0.810	0.817	0.824	0.830	0.833	0.838	0.843	0.848	0.852	0.856	0.860	0.865	0.868
Oceania	0.383	0.385	0.388	0.391	0.394	0.397	0.400	0.403	0.405	0.408	0.410	0.412	0.413	0.414	0.416	0.417	0.419	0.421	0.422	0.424	0.426	0.428	0.431	0.433	0.436	0.440	0.443	0.446	0.449	0.452
American Samoa	0.606	0.609	0.613	0.616	0.619	0.623	0.627	0.630	0.634	0.637	0.641	0.645	0.649	0.652	0.656	0.660	0.663	0.667	0.671	0.674	0.678	0.682	0.686	0.690	0.694	0.698	0.702	0.706	0.709	0.712
Cook Islands	0.625	0.631	0.637	0.643	0.649	0.654	0.659	0.663	0.667	0.671	0.676	0.682	0.687	0.692	0.697	0.701	0.706	0.711	0.715	0.719	0.722	0.726	0.730	0.734	0.739	0.744	0.750	0.755	0.760	0.764
Fiji	0.527	0.532	0.538	0.544	0.550	0.555	0.561	0.567	0.572	0.577	0.582	0.587	0.592	0.596	0.601	0.605	0.609	0.612	0.616	0.619	0.622	0.626	0.630	0.635	0.639	0.644	0.649	0.654	0.659	0.664
Guam	0.693	0.688	0.684	0.684	0.688	0.695	0.704	0.715	0.728	0.741	0.753	0.762	0.767	0.768	0.770	0.771	0.773	0.775	0.778	0.782	0.785	0.789	0.792	0.795	0.797	0.799	0.803	0.807	0.810	0.813
Kiribati	0.425	0.427	0.429	0.432	0.435	0.438	0.441	0.444	0.447	0.451	0.455	0.459	0.463	0.468	0.472	0.476	0.480	0.484	0.487	0.490	0.492	0.495	0.497	0.501	0.504	0.509	0.514	0.518	0.523	0.527
Marshall Islands	0.398	0.404	0.410	0.416	0.423	0.430	0.435	0.439	0.442	0.446	0.450	0.454	0.458	0.463	0.467	0.472	0.477	0.482	0.487	0.493	0.498	0.504	0.509	0.515	0.520	0.525	0.531	0.536	0.541	0.544
Federated States of Micronesia	0.447	0.453	0.459	0.465	0.471	0.478	0.483	0.488	0.492	0.497	0.502	0.507	0.511	0.516	0.521	0.525	0.530	0.534	0.538	0.542	0.546	0.550	0.554	0.558	0.561	0.565	0.569	0.573	0.577	0.580
Nauru	0.499	0.501	0.503	0.504	0.504	0.505	0.505	0.504	0.504	0.503	0.503	0.503	0.503	0.503	0.504	0.506	0.509	0.510	0.515	0.521	0.529	0.538	0.547	0.559	0.573	0.585	0.595	0.605	0.613	0.618
Niue	0.566	0.571	0.576	0.581	0.586	0.591	0.595	0.600	0.605	0.609	0.614	0.618	0.622	0.626	0.631	0.638	0.645	0.651	0.657	0.663	0.669	0.675	0.681	0.685	0.690	0.695	0.699	0.703	0.707	0.711
Northern Mariana Islands	0.692	0.698	0.704	0.709	0.714	0.718	0.722	0.725	0.727	0.731	0.738	0.744	0.748	0.750	0.751	0.752	0.752	0.753	0.753	0.752	0.751	0.750	0.750	0.750	0.750	0.751	0.756	0.761	0.767	0.771
Palau	0.621	0.629	0.636	0.642	0.647	0.652	0.658	0.663	0.668	0.672	0.676	0.680	0.683	0.686	0.690	0.693	0.697	0.700	0.703	0.705	0.707	0.710	0.714	0.717	0.720	0.725	0.729	0.732	0.735	0.738
Papua New Guinea	0.292	0.295	0.299	0.304	0.309	0.313	0.317	0.320	0.323	0.326	0.329	0.331	0.333	0.335	0.337	0.339	0.342	0.345	0.348	0.352	0.356	0.360	0.363	0.367	0.372	0.377	0.382	0.386	0.391	0.394
Samoa	0.531	0.535	0.539	0.543	0.546	0.550	0.555	0.559	0.563	0.567	0.571	0.576	0.581	0.586	0.590	0.595	0.599	0.603	0.607	0.610	0.612	0.615	0.618	0.620	0.623	0.626	0.629	0.633	0.637	0.641
Solomon Islands	0.279	0.283	0.288	0.294	0.300	0.306	0.312	0.317	0.321	0.326	0.328	0.330	0.331	0.332	0.333	0.336	0.339	0.343	0.348	0.353	0.358	0.364	0.371	0.377	0.382	0.387	0.393	0.398	0.403	0.407
Tokelau	0.427	0.432	0.438	0.444	0.450	0.456	0.463	0.470	0.477	0.484	0.491	0.498	0.504	0.511	0.519	0.526	0.534	0.542	0.550	0.557	0.565	0.573	0.580	0.588	0.595	0.602	0.608	0.615	0.621	0.626
Tonga	0.510	0.517	0.522	0.527	0.533	0.538	0.543	0.547	0.551	0.555	0.559	0.563	0.568	0.572	0.576	0.580	0.583	0.587	0.590	0.594	0.598	0.602	0.606	0.610	0.614	0.618	0.622	0.627	0.632	0.636
Tuvalu	0.426	0.434	0.442	0.450	0.458	0.465	0.470	0.476	0.483	0.490	0.496	0.502	0.509	0.514	0.519	0.523	0.527	0.531	0.537	0.541	0.545	0.549	0.553	0.558	0.562	0.567	0.573	0.579	0.584	0.589
Vanuatu	0.361	0.365	0.369	0.373	0.377	0.381	0.386	0.390	0.395	0.399	0.403	0.407	0.410	0.413	0.417	0.420	0.425	0.430	0.435	0.440	0.446	0.451	0.455	0.460	0.464	0.468	0.473	0.477	0.481	0.485

Socio- Demographic Index values for all estimated GBD 2019 locations, 1990-2019																														
Location	1990	1991	1992	1993	1994	1995	1996	1997	1998	1999	2000	2001	2002	2003	2004	2005	2006	2007	2008	2009	2010	2011	2012	2013	2014	2015	2016	2017	2018	2019
Southeast Asia	0.455	0.463	0.471	0.479	0.488	0.496	0.504	0.512	0.517	0.523	0.528	0.533	0.538	0.543	0.548	0.554	0.559	0.566	0.572	0.578	0.585	0.592	0.599	0.606	0.612	0.619	0.626	0.632	0.639	0.644
Cambodia	0.266	0.272	0.277	0.282	0.286	0.291	0.296	0.301	0.307	0.313	0.321	0.328	0.337	0.345	0.354	0.363	0.373	0.382	0.391	0.399	0.406	0.413	0.421	0.428	0.435	0.442	0.449	0.456	0.463	0.469
Indonesia	0.452	0.462	0.472	0.481	0.490	0.499	0.508	0.516	0.522	0.527	0.533	0.537	0.542	0.547	0.552	0.558	0.564	0.571	0.578	0.585	0.593	0.601	0.609	0.617	0.625	0.633	0.640	0.647	0.654	0.660
Laos	0.268	0.274	0.279	0.285	0.290	0.296	0.302	0.309	0.315	0.322	0.329	0.336	0.344	0.351	0.359	0.367	0.376	0.385	0.394	0.403	0.413	0.422	0.431	0.441	0.450	0.458	0.467	0.475	0.483	0.490
Malaysia	0.542	0.548	0.554	0.562	0.572	0.581	0.590	0.600	0.611	0.622	0.630	0.638	0.646	0.652	0.659	0.665	0.671	0.677	0.681	0.687	0.693	0.698	0.704	0.710	0.716	0.722	0.726	0.728	0.732	0.737
Maldives	0.303	0.314	0.324	0.336	0.347	0.359	0.370	0.382	0.394	0.406	0.417	0.427	0.437	0.447	0.456	0.464	0.473	0.481	0.490	0.497	0.504	0.511	0.518	0.525	0.532	0.538	0.544	0.551	0.557	0.562
Mauritius	0.527	0.532	0.535	0.543	0.556	0.565	0.570	0.576	0.583	0.588	0.593	0.598	0.603	0.608	0.614	0.621	0.627	0.633	0.640	0.646	0.652	0.658	0.665	0.673	0.680	0.686	0.690	0.695	0.700	0.705
Myanmar	0.284	0.287	0.290	0.295	0.300	0.306	0.313	0.320	0.327	0.335	0.344	0.353	0.363	0.373	0.384	0.395	0.406	0.417	0.427	0.437	0.446	0.455	0.464	0.473	0.482	0.490	0.498	0.506	0.514	0.521
Philippines	0.497	0.501	0.505	0.509	0.513	0.518	0.522	0.526	0.529	0.532	0.534	0.537	0.540	0.542	0.545	0.547	0.550	0.554	0.558	0.562	0.567	0.572	0.577	0.583	0.589	0.596	0.603	0.610	0.617	0.623
Seychelles	0.567	0.576	0.584	0.592	0.600	0.607	0.614	0.621	0.629	0.636	0.642	0.647	0.652	0.656	0.659	0.662	0.666	0.670	0.673	0.676	0.679	0.683	0.687	0.691	0.696	0.702	0.707	0.713	0.719	0.724
Sri Lanka	0.504	0.511	0.518	0.525	0.532	0.539	0.547	0.554	0.561	0.567	0.573	0.578	0.582	0.587	0.592	0.597	0.602	0.609	0.615	0.621	0.628	0.636	0.644	0.651	0.658	0.666	0.672	0.678	0.684	0.690
Thailand	0.508	0.518	0.527	0.536	0.544	0.553	0.562	0.569	0.574	0.578	0.583	0.589	0.594	0.599	0.605	0.610	0.616	0.623	0.629	0.633	0.638	0.643	0.649	0.655	0.660	0.666	0.671	0.676	0.682	0.687
Timor- Leste	0.274	0.280	0.286	0.293	0.300	0.308	0.317	0.326	0.334	0.339	0.345	0.352	0.358	0.364	0.374	0.388	0.406	0.421	0.436	0.448	0.458	0.469	0.480	0.488	0.493	0.498	0.503	0.508	0.511	0.514
Vietnam	0.390	0.397	0.404	0.412	0.420	0.429	0.438	0.447	0.455	0.463	0.471	0.478	0.486	0.493	0.501	0.509	0.517	0.525	0.533	0.541	0.549	0.558	0.566	0.573	0.581	0.589	0.596	0.604	0.611	0.617
Sub- Saharan Africa	0.291	0.295	0.299	0.302	0.306	0.309	0.314	0.318	0.322	0.327	0.331	0.336	0.341	0.346	0.352	0.359	0.365	0.372	0.380	0.386	0.394	0.401	0.409	0.416	0.423	0.431	0.438	0.445	0.452	0.456
Central sub- Saharan Africa	0.269	0.273	0.276	0.279	0.281	0.283	0.286	0.289	0.292	0.295	0.298	0.302	0.308	0.313	0.320	0.328	0.336	0.346	0.357	0.367	0.378	0.389	0.400	0.412	0.423	0.434	0.445	0.454	0.463	0.470
Angola	0.238	0.243	0.247	0.250	0.253	0.257	0.262	0.267	0.273	0.278	0.283	0.289	0.296	0.303	0.311	0.319	0.330	0.341	0.353	0.364	0.376	0.387	0.398	0.410	0.421	0.432	0.443	0.454	0.463	0.470
Central African Republic	0.186	0.190	0.193	0.196	0.199	0.202	0.205	0.208	0.211	0.215	0.218	0.221	0.225	0.227	0.230	0.233	0.236	0.240	0.244	0.248	0.253	0.258	0.263	0.263	0.263	0.264	0.266	0.268	0.271	0.274
Congo (Brazzaville)	0.364	0.373	0.381	0.389	0.396	0.403	0.410	0.416	0.421	0.426	0.431	0.437	0.442	0.447	0.452	0.458	0.464	0.469	0.475	0.482	0.491	0.500	0.509	0.519	0.528	0.538	0.547	0.556	0.563	0.568
DR Congo	0.260	0.262	0.263	0.263	0.262	0.261	0.259	0.256	0.253	0.250	0.246	0.242	0.239	0.238	0.239	0.242	0.244	0.247	0.251	0.257	0.266	0.277	0.289	0.305	0.321	0.336	0.350	0.362	0.374	0.382
Equatorial Guinea	0.208	0.214	0.222	0.230	0.240	0.252	0.268	0.295	0.318	0.341	0.364	0.391	0.415	0.438	0.460	0.482	0.502	0.522	0.543	0.561	0.578	0.594	0.611	0.626	0.640	0.652	0.663	0.673	0.681	0.685
Gabon	0.388	0.399	0.409	0.420	0.431	0.442	0.453	0.463	0.474	0.483	0.493	0.502	0.510	0.519	0.528	0.537	0.546	0.554	0.563	0.570	0.579	0.587	0.596	0.605	0.614	0.623	0.632	0.641	0.649	0.656
Eastern sub- Saharan Africa	0.235	0.239	0.242	0.245	0.249	0.252	0.257	0.261	0.265	0.270	0.275	0.280	0.285	0.290	0.295	0.301	0.307	0.314	0.321	0.328	0.336	0.343	0.351	0.359	0.367	0.375	0.383	0.391	0.399	0.405
Burundi	0.198	0.201	0.204	0.207	0.209	0.210	0.210	0.211	0.212	0.213	0.213	0.214	0.216	0.218	0.220	0.223	0.226	0.230	0.234	0.238	0.243	0.248	0.254	0.260	0.266	0.270	0.274	0.278	0.282	0.284
Comoros	0.274	0.281	0.288	0.296	0.302	0.309	0.315	0.322	0.328	0.334	0.340	0.346	0.353	0.359	0.365	0.371	0.378	0.384	0.390	0.396	0.401	0.407	0.413	0.419	0.426	0.432	0.438	0.444	0.450	0.455
Djibouti	0.275	0.277	0.280	0.285	0.289	0.293	0.297	0.302	0.306	0.311	0.317	0.322	0.327	0.333	0.339	0.345	0.353	0.360	0.368	0.376	0.384	0.392	0.400	0.408	0.416	0.425	0.434	0.443	0.452	0.459
Eritrea	0.198	0.203	0.210	0.218	0.228	0.238	0.248	0.258	0.267	0.275	0.283	0.289	0.296	0.302	0.307	0.312	0.317	0.322	0.326	0.330	0.334	0.340	0.346	0.353	0.360	0.367	0.375	0.382	0.390	0.396
Ethiopia	0.144	0.146	0.147	0.149	0.151	0.153	0.157	0.161	0.164	0.167	0.171	0.176	0.180	0.183	0.189	0.195	0.203	0.213	0.223	0.233	0.244	0.256	0.268	0.279	0.291	0.302	0.313	0.324	0.334	0.343
Kenya	0.333	0.341	0.348	0.354	0.360	0.366	0.372	0.378	0.383	0.388	0.392	0.397	0.401	0.404	0.409	0.413	0.418	0.424	0.429	0.435	0.441	0.448	0.455	0.463	0.470	0.478	0.486	0.494	0.502	0.508
Madagascar	0.265	0.268	0.270	0.273	0.275	0.277	0.280	0.283	0.286	0.290	0.294	0.299	0.302	0.307	0.311	0.316	0.320	0.324	0.328	0.332	0.336	0.342	0.348	0.355	0.361	0.369	0.376	0.383	0.391	0.396
Malawi	0.213	0.215	0.215	0.217	0.218	0.220	0.225	0.230	0.235	0.240	0.245	0.249	0.254	0.260	0.267	0.274	0.281	0.290	0.299	0.308	0.317	0.326	0.335	0.342	0.350	0.358	0.365	0.372	0.379	0.384
Mozambique	0.120	0.122	0.123	0.126	0.129	0.131	0.137	0.144	0.152	0.159	0.165	0.172	0.179	0.186	0.194	0.201	0.208	0.215	0.223	0.230	0.237	0.244	0.252	0.260	0.268	0.277	0.285	0.294	0.301	0.307
Rwanda	0.257	0.259	0.262	0.265	0.261	0.261	0.262	0.265	0.269	0.272	0.277	0.283	0.291	0.298	0.307	0.315	0.325	0.333	0.342	0.351	0.359	0.368	0.376	0.384	0.391	0.399	0.407	0.415	0.422	0.429
Somalia	0.051	0.051	0.052	0.053	0.054	0.055	0.056	0.057	0.057	0.058	0.059	0.060	0.061	0.062	0.063	0.064	0.065	0.066	0.067	0.068	0.069	0.070	0.072	0.073	0.074	0.076	0.077	0.079	0.080	0.081

Socio- Demographic Index values for all estimated GBD 2019 locations, 1990-2019																														
**Location**	**1990**	**1991**	**1992**	**1993**	**1994**	**1995**	**1996**	**1997**	**1998**	**1999**	**2000**	**2001**	**2002**	**2003**	**2004**	**2005**	**2006**	**2007**	**2008**	**2009**	**2010**	**2011**	**2012**	**2013**	**2014**	**2015**	**2016**	**2017**	**2018**	**2019**
South Sudan	0.248	0.251	0.253	0.256	0.259	0.261	0.264	0.267	0.271	0.274	0.277	0.281	0.284	0.288	0.292	0.296	0.301	0.305	0.310	0.315	0.320	0.325	0.330	0.334	0.339	0.342	0.345	0.351	0.358	0.363
Uganda	0.167	0.169	0.172	0.176	0.181	0.187	0.194	0.201	0.208	0.216	0.225	0.234	0.243	0.253	0.262	0.272	0.282	0.293	0.303	0.314	0.325	0.335	0.345	0.355	0.364	0.373	0.382	0.391	0.399	0.404
Tanzania	0.260	0.265	0.269	0.273	0.276	0.280	0.284	0.288	0.292	0.296	0.301	0.305	0.310	0.315	0.320	0.326	0.331	0.337	0.342	0.348	0.354	0.361	0.368	0.375	0.383	0.391	0.399	0.408	0.416	0.423
Zambia	0.299	0.304	0.307	0.311	0.314	0.316	0.319	0.323	0.326	0.330	0.334	0.340	0.345	0.352	0.359	0.367	0.376	0.386	0.396	0.407	0.418	0.429	0.440	0.451	0.462	0.472	0.481	0.491	0.499	0.505
Southern sub- Saharan Africa	0.513	0.517	0.522	0.526	0.530	0.535	0.539	0.544	0.548	0.553	0.557	0.562	0.567	0.571	0.576	0.581	0.586	0.591	0.596	0.601	0.605	0.610	0.614	0.619	0.623	0.628	0.632	0.636	0.639	0.642
Botswana	0.431	0.441	0.451	0.459	0.467	0.475	0.483	0.491	0.498	0.506	0.514	0.521	0.528	0.535	0.541	0.548	0.555	0.562	0.569	0.575	0.581	0.587	0.593	0.600	0.606	0.612	0.618	0.624	0.630	0.634
eSwatini	0.392	0.401	0.410	0.419	0.427	0.435	0.443	0.450	0.456	0.462	0.468	0.474	0.479	0.484	0.490	0.495	0.501	0.507	0.513	0.519	0.526	0.532	0.538	0.545	0.551	0.557	0.563	0.569	0.574	0.577
Lesotho	0.321	0.327	0.333	0.340	0.346	0.353	0.360	0.367	0.373	0.380	0.387	0.393	0.399	0.405	0.411	0.417	0.423	0.429	0.435	0.441	0.448	0.455	0.462	0.469	0.476	0.483	0.489	0.496	0.502	0.507
Namibia	0.454	0.459	0.465	0.470	0.475	0.480	0.486	0.491	0.495	0.500	0.505	0.509	0.514	0.518	0.523	0.529	0.534	0.540	0.546	0.552	0.558	0.564	0.571	0.577	0.584	0.591	0.597	0.603	0.608	0.612
South Africa	0.552	0.556	0.560	0.564	0.567	0.572	0.576	0.580	0.584	0.588	0.593	0.597	0.602	0.607	0.611	0.616	0.622	0.627	0.632	0.637	0.642	0.647	0.651	0.656	0.660	0.664	0.668	0.672	0.676	0.678
Zimbabwe	0.394	0.403	0.409	0.415	0.421	0.426	0.432	0.437	0.441	0.444	0.446	0.448	0.448	0.447	0.444	0.441	0.437	0.433	0.428	0.425	0.425	0.427	0.432	0.438	0.445	0.452	0.459	0.465	0.471	0.476
Western sub- Saharan Africa	0.268	0.272	0.276	0.280	0.284	0.288	0.292	0.297	0.301	0.306	0.311	0.316	0.322	0.328	0.335	0.343	0.351	0.359	0.367	0.375	0.383	0.391	0.399	0.407	0.415	0.422	0.430	0.437	0.443	0.448
Benin	0.209	0.213	0.218	0.222	0.227	0.231	0.236	0.241	0.245	0.250	0.254	0.259	0.263	0.267	0.271	0.275	0.279	0.283	0.288	0.292	0.297	0.301	0.306	0.312	0.318	0.324	0.331	0.338	0.346	0.352
Burkina Faso	0.125	0.128	0.131	0.134	0.137	0.140	0.144	0.147	0.152	0.156	0.161	0.166	0.170	0.175	0.180	0.186	0.191	0.196	0.200	0.205	0.210	0.215	0.220	0.226	0.231	0.236	0.241	0.247	0.252	0.257
Cape Verde	0.292	0.298	0.305	0.311	0.318	0.326	0.333	0.342	0.350	0.360	0.370	0.379	0.388	0.397	0.407	0.415	0.424	0.434	0.443	0.452	0.461	0.469	0.477	0.484	0.491	0.498	0.505	0.512	0.519	0.525
Cameroon	0.313	0.320	0.325	0.330	0.334	0.338	0.342	0.346	0.349	0.353	0.357	0.360	0.364	0.368	0.373	0.378	0.384	0.390	0.397	0.404	0.412	0.420	0.428	0.436	0.445	0.455	0.464	0.474	0.483	0.490
Chad	0.108	0.112	0.115	0.118	0.120	0.123	0.125	0.128	0.130	0.132	0.135	0.137	0.140	0.144	0.149	0.156	0.162	0.167	0.173	0.179	0.185	0.191	0.197	0.203	0.210	0.216	0.223	0.228	0.234	0.238
Côte d' Ivoire	0.256	0.260	0.265	0.270	0.275	0.279	0.285	0.290	0.295	0.301	0.305	0.310	0.314	0.318	0.322	0.325	0.329	0.333	0.337	0.341	0.346	0.350	0.355	0.362	0.369	0.376	0.384	0.393	0.401	0.408
The Gambia	0.218	0.223	0.227	0.232	0.237	0.241	0.246	0.251	0.256	0.262	0.268	0.274	0.280	0.286	0.293	0.300	0.306	0.313	0.319	0.327	0.334	0.341	0.348	0.356	0.363	0.370	0.378	0.385	0.393	0.399
Ghana	0.355	0.362	0.368	0.374	0.380	0.385	0.391	0.396	0.401	0.406	0.411	0.415	0.420	0.426	0.431	0.437	0.444	0.450	0.458	0.466	0.474	0.484	0.494	0.504	0.514	0.523	0.531	0.541	0.549	0.557
Guinea	0.175	0.178	0.182	0.186	0.190	0.194	0.199	0.204	0.209	0.214	0.219	0.224	0.229	0.235	0.239	0.244	0.249	0.253	0.258	0.263	0.267	0.272	0.278	0.284	0.290	0.296	0.303	0.310	0.318	0.325
Guinea- Bissau	0.200	0.205	0.209	0.214	0.219	0.224	0.230	0.236	0.239	0.244	0.249	0.253	0.257	0.261	0.266	0.271	0.275	0.280	0.286	0.291	0.297	0.304	0.310	0.316	0.322	0.328	0.335	0.342	0.349	0.355
Liberia	0.221	0.222	0.219	0.214	0.206	0.196	0.183	0.176	0.175	0.184	0.203	0.220	0.238	0.245	0.252	0.258	0.265	0.272	0.279	0.287	0.296	0.305	0.314	0.325	0.335	0.344	0.351	0.358	0.365	0.370
Mali	0.126	0.129	0.132	0.136	0.139	0.143	0.147	0.151	0.155	0.159	0.163	0.168	0.173	0.178	0.183	0.188	0.193	0.198	0.203	0.209	0.214	0.220	0.225	0.230	0.235	0.241	0.247	0.253	0.259	0.263
Mauritania	0.308	0.314	0.319	0.326	0.332	0.338	0.344	0.349	0.355	0.360	0.365	0.369	0.374	0.379	0.384	0.390	0.398	0.406	0.413	0.420	0.427	0.435	0.443	0.450	0.459	0.467	0.474	0.482	0.490	0.496
Niger	0.073	0.075	0.076	0.078	0.079	0.081	0.082	0.084	0.085	0.087	0.089	0.091	0.093	0.096	0.098	0.101	0.104	0.108	0.111	0.115	0.119	0.123	0.128	0.133	0.138	0.143	0.148	0.153	0.158	0.162
Nigeria	0.305	0.308	0.312	0.315	0.319	0.324	0.329	0.334	0.339	0.344	0.350	0.356	0.363	0.371	0.381	0.392	0.402	0.412	0.422	0.432	0.442	0.451	0.460	0.469	0.478	0.487	0.495	0.503	0.510	0.515
São Tomé and PrÍncipe	0.299	0.302	0.306	0.309	0.313	0.317	0.322	0.327	0.332	0.338	0.344	0.351	0.358	0.365	0.373	0.381	0.390	0.398	0.407	0.416	0.424	0.433	0.443	0.452	0.461	0.470	0.478	0.487	0.495	0.502
Senegal	0.227	0.233	0.239	0.245	0.251	0.257	0.262	0.267	0.272	0.277	0.282	0.286	0.290	0.295	0.299	0.304	0.308	0.313	0.318	0.324	0.330	0.336	0.342	0.348	0.354	0.361	0.368	0.375	0.382	0.389
Sierra Leone	0.207	0.209	0.210	0.212	0.215	0.218	0.218	0.218	0.219	0.218	0.219	0.221	0.224	0.229	0.234	0.239	0.245	0.252	0.260	0.267	0.275	0.283	0.292	0.304	0.314	0.321	0.328	0.335	0.342	0.347
Togo	0.266	0.272	0.278	0.281	0.286	0.291	0.296	0.302	0.306	0.310	0.313	0.317	0.320	0.323	0.327	0.330	0.334	0.338	0.342	0.347	0.352	0.358	0.364	0.371	0.379	0.386	0.394	0.402	0.411	0.417

SDI Reference Quintiles

SDI_quintile	lower_bound	upper_bound
Low SDI	0.0000	0.4547
Low-middle SDI	0.4547	0.6077
Middle SDI	0.6077	0.6895
High-middle SDI	0.6895	0.8051
High SDI	0.8051	1.0000

GBD Causes Mapped to ICD-9 and ICD-10 Codes

List of International Classification of Diseases (ICD) codes mapped to the Global Burden of Disease cause list				
**Cause**	**ICD10**	**ICD10 Used in Hospital/ Claims Analyses**	**ICD9**	**ICD9 Used in Hospital/ Claims Analyses**
Respiratory infections and tuberculosis	A10-A14, A15 -A18.89, A19-A19.9, A48.1, A70, B90-B90.9, B96.0-B96.1, B97.21, B97.4-B97.6, H65 -H70.93, J00-J06.9, J09-J18.2, J18.8-J18.9, J19.6-J22.9, J36-J36.0, J85.1, J91.0, K67.3, K93.0, M49.0, N74.0-N74.1, P23-P23.9, P37.0, U04 -U04.9, U84.3, Z03.0, Z11.1, Z20.1, Z23.2, Z25.1	A10-A19.9, A48.1, A70, B90-B90.9, B96.0-B97.6, H65-H71.93, J00-J36.0, J85.1-J91.0, K67.3, K93.0, M49.0, N74.0-N74.1, P23-P23.9, P37.0, U04-U84.3	010-019.9, 079.82, 137-137.9, 320.4, 381 -383.9, 460-469, 470.0, 475-475.9, 480 -484, 484.1-490.9, 510-511.9, 513.0-513.9, 730.4-730.6, 770.0, V01.1, V01.82, V03.2, V04.7, V04.81, V12.01, V12.61, V74.1	010-019.9, 079.82, 137-137.9, 320.4, 381.0-382.3, 385.3-385.82, 460-484, 484.1-490.9, 510-513.9, 730.4-730.6, 770.0, V12.61
Tuberculosis	A10-A14, A15 -A18.89, A19-A19.9, B90-B90.9, K67.3, K93.0, M49.0, N74.0-N74.1, P37.0, U84.3, Z03.0, Z11.1, Z20.1, Z23.2	A10-A19.9, B90-B90.9, K67.3, K93.0, M49.0, N74.0-N74.1, P37.0, U84.3	010-019.9, 137-137.9, 320.4, 730.4-730.6, V01.1, V03.2, V12.01, V74.1	010-019.9, 137-137.9, 320.4, 730.4-730.6
Multidrug-resistant tuberculosis without extensive drug resistance	U84.3			
Drug-susceptible tuberculosis	A10-A14, A15 -A18.89, A19-A19.9, B90-B90.9, K67.3, K93.0, M49.0, N74.0-N74.1, P37.0		010-019.9, 137-137.9, 320.4, 730.4-730.6	
Digestive diseases	I84-I85.9, I98.2, K20-K23.8, K25-K31.9, K35-K38.9, K40-K42.9, K44-K46.9, K50-K51.319, K51.5-K52, K52.2-K52.9, K55-K62, K62.4-K62.6, K62.8-K63.4, K63.8-K67, K67.8-K68.1, K68.12-K68.9, K70-K75, K75.2, K75.4-K76.2, K76.4-K77.8, K80-K80.81, K81-K83.9, K85-K87.1, K90-K90.9, K92-K92.9, K93.8, R11-R19.8, R85-R85.9, Z13.81-Z13.818, Z43.1-Z43.4, Z52.6, Z83.7-Z83.79, Z87.1-Z87.19, Z94.4	I84-I85.9, I98.2-I98.2, K20-K42.9, K44-K51.319, K51.5 -K51.919, K52.2-K62, K62.4-K62.6, K62.8-K63.4, K63.8 -K67, K67.8-K68.1, K68.12-K90.9, K92-K92.9, K93.8, R12-R18.9, R85-R85.9, Z52.6, Z94.4	455-455.9, 456.0-456.21, 530-530.85, 530.89-536.3, 536.8-538, 540-543.9, 550-551.1, 551.3-552.1, 552.3-553.1, 553.3-558.9, 560-560.39, 560.8-562.13, 564-564.1, 564.5-569, 569.1-569.5, 569.81-572, 572.2-579.2, 579.4-579.9, 784-784.99, 787-787.99, 789.9, 792.1-792.4, V12.7-V12.79, V18.5-V18.59, V42.7, V45.72, V45.75, V47.3, V58.75, V59.6	455-456.21, 530-530.85, 530.89-536.3, 536.8-538, 540-551.1, 551.3-552.1, 552.3-553.1, 553.3-564.1, 564.5-569, 569.1-569.49, 569.81-579.2, 579.4-579.9, 787.1-792.4, V42.7, V59.6
Neoplasms	C00-C07, C08-C19.0, C20, C21-C21.8, C22-C22.4, C22.7-C23, C24-C26.1, C26.8-C26.9, C30-C30.1, C31-C33, C34-C34.92, C37-C37.0, C38-C39.9, C40-C41.4, C41.8-C41.9, C43-C45.2, C45.7, C45.9, C47-C4A, C50-C50.629, C50.8-C52, C53-C54.3, C54.8-C56.2, C56.9-C58.0, C60-C64.2, C64.9-C69.92, C70-C70.1, C70.9-C73, C74-C75.5, C75.8-C79.9, C80-C81.49, C81.7-C81.79, C81.9-C85.29, C85.7-C86.6, C88-C90.32, C91-C93.7, C93.9-C95.2, C95.7-C97.9, D00-D24.9, D26.0-D39.9, D4-D49.9, E34.0, K51.4-K51.419, K62.0-K62.3, K63.5, N60-N60.99, N84.0-N84.1, N87-N87.9, Z03.1, Z08-Z09.9, Z12-Z12.9, Z80-Z80.9, Z85-Z85.9, Z86.0-Z86.03	C44.01-C75.92, D00 -D24.9, D26.0-D39.9, D4-D49.9, E34.0, K51.4-K51.419, K62.0-K62.3, K63.5, N60-N60.99, N84.0-N84.1, N87-N87.9	140-175.9, 177-217.8, 219-237.6, 237.70-237.72, 237.9-239.9, 569.0, 610-610.9, 622.1-622.2, 622.7, V07.39, V10-V11, V13.22-V13.24, V16-V16.9, V42.4, V42.81-V42.82, V59.2-V59.3, V72.32, V76-V76.9	173.01-217.8, 219-237.6, 237.70-237.72, 237.9-239.9, 569.0, 610-610.9, 622.1-622.2, 622.7
Neurological disorders	F00-F02.0, F02.2-F02.3, F02.8-F03.91, F06.2, G10-G10.0, G11-G13.8, G20-G21, G21.2-G24, G24.1-G25.0, G25.2-G25.3, G25.5, G25.8-G26.0, G30-G31.1, G31.8-G32.89, G35-G35.0, G36-G37.9, G40-G41.9, G43-G44.89, G50-G54.1, G54.5-G62, G62.2-G65.2, G70-G71.19, G71.3-G72, G72.1-G73.7, G80-G83.9, G89-G93.6, G93.8-G95.29, G95.8-G96, G96.1, G96.12-G96.9, G98-G99.8, M33-M33.99, M60-M60.19, M60.8-M60.9, M79.7, R25-R27.9, R29-R29.91, R41-R42.0, R56-R56.9, R90-R90.89, Z03.3, Z13.85, Z13.858, Z82.0, Z86.6-Z86.69	F00-F06.2, G12-G20.9, G30-G31.1, G31.8-G44.41	290-290.9, 294.0-294.9, 307.8-307.89, 315-315.9, 330-331.8, 331.82-333.91, 333.93-346.93, 348-348.9, 350-353.0, 353.5-357.5, 357.7-359.23, 359.29-359.9, 710.3-710.4, 725-725.9, 728-728.85, 728.87-728.9, 775.2, 780.3-780.59, 780.7-780.72, 780.96, 781-	290-290.9, 294.0-294.9, 307.81, 331-331.7, 331.82-332.0, 335-346.93

			781.99, 793.0, 799.3-799.7, V17.2, V58.72	
Self-harm and interpersonal violence	T74.2-U03, X60-X64.9, X66-Y08.9, Y35-Y38.9, Y87.0-Y87.2, Y89.0-Y89.1	T74.2-U03, X60-X64.9, X66-Y08.9, Y35-Y38.9, Y87.0-Y87.2, Y89.0-Y89.1	E950-E979.9, E990-E999.1	E95.0-E99.91
Substance use disorders	E24.4, F10-F19.99, G31.2, G62.1, P96.1, R78.0-R78.9, X45-X45.9, X65-X65.9, Y15-Y15.9, Z81.1-Z81.4	F10.2-F15.99, G31.2, X65-X65.9, Y15-Y15.9	291-292.9, 303-305.93, 790.3, E850.0-E850.29, E860-E860.19, V11.3, V15.8-V15.83, V15.85-V15.86, nan	291.0-291.9, 303.0-305.73, E85.00-E85.029
Transport injuries	V00-V86.99, V87.2-V87.3, V88.2-V88.3, V90-V98.8	V00-V98.8	E800-E800.3, E801-E801.3, E802-E802.3, E803-E803.3, E804-E804.3, E805-E805.3, E806-E806.3, E807-E807.3, E810.0-E810.7, E811.0-E811.7, E812.0-E812.7, E813.0-E813.7, E814.0-E814.7, E815.0-E815.7, E816.0-E816.7, E817.0-E817.7, E818.0-E818.7, E819.0-E819.7, E820.0-E820.7, E821.0-E821.7, E822.0-E822.7, E823.0-E823.7, E824.0-E824.7, E825.0-E825.7, E826.0-E826.4, E827.0-E827.4, E828.0-E828.4, E829.0-E829.4, E830-E838.9, E840-E849.9, E929.1, V03, V07.8-V07.9, V13, V13.8, V13.9, V15.2, V15.3, V15.9, V19, V42, V42.8, V42.9-V43, V47-V47.1	E80.0-E84.99, E92.91
Unintentional injuries	D69.5-D69.59, D70.1-D70.2, D78-D78.89, D89.81-D89.813, E03.2, E06.4, E09-E09.9, E16.0, E23.1, E24.2, E27.3, E36-E36.8, E66.1, E86.02-E87.99, E89-E89.9, G21.0-G21.19, G24.0-G24.09, G25.1, G25.4, G25.6-G25.79, G62.0, G72.0, G93.7, G96.0, G96.11, G97-G97.9, H02.81-H02.819, H05.33-H05.339, H05.42-H05.53, H44.6-H44.799, H59-H59.89, H91.0-H91.09, H95-H95.9, I95.2-I95.81, I97-I97.9, J70-J70.5, J95-J95.9, K08.5-K08.59, K43-K43.9, K52.0, K62.7, K68.11, K91-K91.9, K94-K95.89, L23.3, L27.0-L27.1, L55-L55.9, L56.0-L56.1, L58-L58.9, L64.0, L76-L76.82, M10.2-M10.29, M60.2-M60.28, M87.1-M87.19, M96-M96.9, N14-N14.4, N30.4-N30.41, N46.021, N46.121, N52.2-N52.39, N65-N65.1, N99-N99.9, P93-P93.8, P96.2, P96.5, R50.2-R50.83, W00-W46.2, W49-W62.9, W64-W70.9, W73-W81.9, W83-W94.9, W97.9, W99-X06.9, X08-X44.9, X46-X58.9, Y10-Y14.9, Y16-Y19.9, Y40-Y84.9, Y88-Y88.3, Z21.0, Z42-Z43.0, Z43.8-Z43.9, Z48-Z48.9, Z51-Z51.9, Z88-Z88.9, Z92-Z94.0, Z94.6, Z94.8-Z94.9, Z96-Z96.49, Z96.6-Z97.2, Z97.8-Z99.12, Z99.3-Z99.9	D69.5-D69.59, D70.1-D70.2, D78-D78.89, D89.81-D89.813, E03.2, E06.4, E09-E09.9, E16.0, E23.1, E24.2, E27.3, E36-E36.8, E66.1, E89-E89.9, G21.0-G25.79, G62.0-G97.9, H02.81-H02.819, H05.33-H05.339, H05.42-H05.53, H44.6-H44.799, H59-H59.89, H91.0-H91.09, H95-H95.9, I95.2-I97.9, J70-J70.5, J95-J95.9, K08.5-K08.59, K43-K43.9, K52.0, K62.7, K68.11, K91-K91.9, K94-K95.89, L23.3, L27.0-L27.1, L55-L55.9, L56.0-L56.1, L58-L58.9, L64.0, L76-L76.82, M10.2-M10.29, M60.2-M60.28, M87.1-M87.19, M96-M96.9, N14-N14.4, N30.4-N30.41, N46.021-N46.121, N52.2-N52.39, N65-N65.1, N99-N99.9, P93-P93.8, P96.2-P96.5, R50.2-R50.83, W00-X58.9, Y40-Y84.9, Y88-Y88.3, Z21.0, Z42-Z51.9, Z88-Z94.0, Z94.6-Z99.9	244.0-244.1, 244.3, 251.3, 253.7, 279.5-279.53, 331.81, 333.92, 349-349.9, 357.6, 359.24, 360.5-360.69, 374.86, 376.6, 379.6-379.63, 440.3-440.32, 457.0, 458.2-458.29, 518.6-518.7, 519.0-519.1, 525.6-525.79, 526.62-526.63, 530.86-530.87, 536.4-536.49, 539-539.9, 551.2-551.29, 552.2-552.29, 553.2-553.29, 564.2-564.4, 569.6-569.8, 579.3, 595.82, 596.81-596.83, 598.2, 612-612.1, 709.4, 770.1-770.18, 779.4-779.5, 780.62-780.66, 995.89, E850.3-E858.99, E862-E869.99, E870-E876.9, E878-E879.9, E880-E886.99, E888-E928.89, E929.2-E929.5, E930-E949.9, V44-V45, V45.2-V45.4, V45.7, V45.77, V45.79-V45.8, V45.87-V45.89	244.0-244.1, 244.3, 251.3, 253.7, 279.5-279.53, 331.81, 333.92, 349-349.9, 357.6-359.24, 360.5-360.69, 374.86, 376.6, 379.6-379.63, 440.3-440.32, 457.0, 458.2-458.29, 518.6-519.1, 525.6-525.79, 526.62-526.63, 530.86-530.87, 536.4-536.49, 539-539.9, 551.2-551.29, 552.2-552.29, 553.2-553.29, 564.2-564.4, 569.6-569.8, 579.3, 595.82, 596.81-596.83, 598.2, 612-612.1, 709.4, 770.1-770.18, 779.4-780.66, 995.89, E85.6-E92.889, E92.93-E94.99, V44-V45.89

Figure 1A Number of new cases of MDR-TB among HIV-negative individuals in 204 countries and territories, all ages, 2019

measure	location	sex	age	cause	metric	year	value	95% UI (lower)	95% UI (upper)
Incidence	India	Both	All ages	MDR-TB	Number	2019	219863	41522	546672
Incidence	Russian Federation	Both	All ages	MDR-TB	Number	2019	30973	16222	49253
Incidence	China	Both	All ages	MDR-TB	Number	2019	29613	5485	94640
Incidence	Pakistan	Both	All ages	MDR-TB	Number	2019	27894	8880	67661
Incidence	Nigeria	Both	All ages	MDR-TB	Number	2019	11963	2710	33148
Incidence	Ukraine	Both	All ages	MDR-TB	Number	2019	11684	6245	18446
Incidence	Philippines	Both	All ages	MDR-TB	Number	2019	9159	2582	21465
Incidence	Bangladesh	Both	All ages	MDR-TB	Number	2019	7780	1870	19146
Incidence	Ethiopia	Both	All ages	MDR-TB	Number	2019	6762	1301	22695
Incidence	Viet Nam	Both	All ages	MDR-TB	Number	2019	6063	1778	14589
Incidence	South Africa	Both	All ages	MDR-TB	Number	2019	5851	2051	13702
Incidence	Myanmar	Both	All ages	MDR-TB	Number	2019	5756	2005	12002
Incidence	Democratic Republic of the Congo	Both	All ages	MDR-TB	Number	2019	5333	668	19685
Incidence	Somalia	Both	All ages	MDR-TB	Number	2019	4251	1163	11829
Incidence	Uzbekistan	Both	All ages	MDR-TB	Number	2019	4071	1523	7706
Incidence	Mozambique	Both	All ages	MDR-TB	Number	2019	3607	1165	7890
Incidence	United Republic of Tanzania	Both	All ages	MDR-TB	Number	2019	2785	502	9276
Incidence	Uganda	Both	All ages	MDR-TB	Number	2019	2498	526	6500
Incidence	Thailand	Both	All ages	MDR-TB	Number	2019	2390	659	5891
Incidence	Kazakhstan	Both	All ages	MDR-TB	Number	2019	2248	1195	3629
Incidence	Nepal	Both	All ages	MDR-TB	Number	2019	2179	529	5774
Incidence	Zimbabwe	Both	All ages	MDR-TB	Number	2019	2075	301	7024
Incidence	Brazil	Both	All ages	MDR-TB	Number	2019	1982	371	5481
Incidence	Madagascar	Both	All ages	MDR-TB	Number	2019	1942	291	5848
Incidence	Peru	Both	All ages	MDR-TB	Number	2019	1937	973	3577
Incidence	Angola	Both	All ages	MDR-TB	Number	2019	1807	237	6618
Incidence	Kenya	Both	All ages	MDR-TB	Number	2019	1770	570	4253
Incidence	Zambia	Both	All ages	MDR-TB	Number	2019	1523	238	5268
Incidence	Democratic People's Republic of Korea	Both	All ages	MDR-TB	Number	2019	1400	450	3194
Incidence	Kyrgyzstan	Both	All ages	MDR-TB	Number	2019	1370	487	2539
Incidence	Ghana	Both	All ages	MDR-TB	Number	2019	1346	206	3934
Incidence	Burundi	Both	All ages	MDR-TB	Number	2019	1299	180	4383
Incidence	Indonesia	Both	All ages	MDR-TB	Number	2019	1269	151	4817
Incidence	Côte d'Ivoire	Both	All ages	MDR-TB	Number	2019	1138	177	3896
Incidence	Azerbaijan	Both	All ages	MDR-TB	Number	2019	1096	343	2105
Incidence	Afghanistan	Both	All ages	MDR-TB	Number	2019	1042	181	3419
Incidence	Belarus	Both	All ages	MDR-TB	Number	2019	988	661	1366
Incidence	Burkina Faso	Both	All ages	MDR-TB	Number	2019	975	143	3216
Incidence	Eritrea	Both	All ages	MDR-TB	Number	2019	970	141	3210
Incidence	Republic of Moldova	Both	All ages	MDR-TB	Number	2019	950	644	1323
Incidence	Cameroon	Both	All ages	MDR-TB	Number	2019	800	114	2472
Incidence	Malawi	Both	All ages	MDR-TB	Number	2019	794	152	2295
Incidence	South Sudan	Both	All ages	MDR-TB	Number	2019	716	115	2236
Incidence	Mali	Both	All ages	MDR-TB	Number	2019	688	86	2050
Incidence	Tajikistan	Both	All ages	MDR-TB	Number	2019	685	314	1228
Incidence	Chad	Both	All ages	MDR-TB	Number	2019	678	94	2058
Incidence	Rwanda	Both	All ages	MDR-TB	Number	2019	664	259	1395

Incidence	Niger	Both	All ages	MDR-TB	Number	2019	612	87	1945
Incidence	Mexico	Both	All ages	MDR-TB	Number	2019	610	119	1819
Incidence	Morocco	Both	All ages	MDR-TB	Number	2019	570	164	1350
Incidence	Egypt	Both	All ages	MDR-TB	Number	2019	568	128	1476
Incidence	Turkey	Both	All ages	MDR-TB	Number	2019	567	253	1115
Incidence	Namibia	Both	All ages	MDR-TB	Number	2019	470	189	916
Incidence	Bolivia (Plurinational State of)	Both	All ages	MDR-TB	Number	2019	466	69	1514
Incidence	Eswatini	Both	All ages	MDR-TB	Number	2019	438	104	1081
Incidence	Lesotho	Both	All ages	MDR-TB	Number	2019	435	148	948
Incidence	Republic of Korea	Both	All ages	MDR-TB	Number	2019	425	62	1637
Incidence	Saudi Arabia	Both	All ages	MDR-TB	Number	2019	423	79	1262
Incidence	Botswana	Both	All ages	MDR-TB	Number	2019	422	79	1324
Incidence	Senegal	Both	All ages	MDR-TB	Number	2019	413	125	929
Incidence	Colombia	Both	All ages	MDR-TB	Number	2019	406	69	1097
Incidence	Papua New Guinea	Both	All ages	MDR-TB	Number	2019	399	146	916
Incidence	Georgia	Both	All ages	MDR-TB	Number	2019	393	226	627
Incidence	Turkmenistan	Both	All ages	MDR-TB	Number	2019	391	143	772
Incidence	Guinea	Both	All ages	MDR-TB	Number	2019	375	51	1208
Incidence	Iraq	Both	All ages	MDR-TB	Number	2019	364	107	847
Incidence	Sierra Leone	Both	All ages	MDR-TB	Number	2019	341	48	1104
Incidence	Cambodia	Both	All ages	MDR-TB	Number	2019	338	50	1120
Incidence	Central African Republic	Both	All ages	MDR-TB	Number	2019	329	52	1085
Incidence	Mongolia	Both	All ages	MDR-TB	Number	2019	313	61	871
Incidence	Ecuador	Both	All ages	MDR-TB	Number	2019	308	57	931
Incidence	Romania	Both	All ages	MDR-TB	Number	2019	291	118	588
Incidence	Togo	Both	All ages	MDR-TB	Number	2019	276	41	793
Incidence	Benin	Both	All ages	MDR-TB	Number	2019	260	48	727
Incidence	Malaysia	Both	All ages	MDR-TB	Number	2019	250	60	684
Incidence	Taiwan (Province of China)	Both	All ages	MDR-TB	Number	2019	246	29	908
Incidence	Sudan	Both	All ages	MDR-TB	Number	2019	216	24	878
Incidence	Congo	Both	All ages	MDR-TB	Number	2019	213	25	765
Incidence	Lithuania	Both	All ages	MDR-TB	Number	2019	193	112	295
Incidence	Germany	Both	All ages	MDR-TB	Number	2019	189	75	389
Incidence	Iran (Islamic Republic of)	Both	All ages	MDR-TB	Number	2019	182	52	442
Incidence	Yemen	Both	All ages	MDR-TB	Number	2019	180	38	657
Incidence	Comoros	Both	All ages	MDR-TB	Number	2019	170	21	557
Incidence	Djibouti	Both	All ages	MDR-TB	Number	2019	159	68	322
Incidence	Algeria	Both	All ages	MDR-TB	Number	2019	140	13	663
Incidence	Venezuela (Bolivarian Republic of)	Both	All ages	MDR-TB	Number	2019	127	15	429
Incidence	Japan	Both	All ages	MDR-TB	Number	2019	126	14	533
Incidence	Italy	Both	All ages	MDR-TB	Number	2019	123	49	250
Incidence	Liberia	Both	All ages	MDR-TB	Number	2019	120	14	489
Incidence	Gabon	Both	All ages	MDR-TB	Number	2019	117	15	428
Incidence	Armenia	Both	All ages	MDR-TB	Number	2019	116	25	272
Incidence	United States of America	Both	All ages	MDR-TB	Number	2019	112	44	243
Incidence	Gambia	Both	All ages	MDR-TB	Number	2019	88	11	297
Incidence	Argentina	Both	All ages	MDR-TB	Number	2019	87	12	318
Incidence	France	Both	All ages	MDR-TB	Number	2019	83	30	181
Incidence	United Kingdom	Both	All ages	MDR-TB	Number	2019	76	28	166
Incidence	Paraguay	Both	All ages	MDR-TB	Number	2019	70	8	229
Incidence	Guatemala	Both	All ages	MDR-TB	Number	2019	65	10	204

Incidence	Honduras	Both	All ages	MDR-TB	Number	2019	63	10	202
Incidence	Mauritania	Both	All ages	MDR-TB	Number	2019	62	7	196
Incidence	Poland	Both	All ages	MDR-TB	Number	2019	50	18	115
Incidence	Latvia	Both	All ages	MDR-TB	Number	2019	47	23	85
Incidence	Equatorial Guinea	Both	All ages	MDR-TB	Number	2019	46	6	146
Incidence	United Arab Emirates	Both	All ages	MDR-TB	Number	2019	44	5	169
Incidence	Australia	Both	All ages	MDR-TB	Number	2019	43	18	91
Incidence	Guinea-Bissau	Both	All ages	MDR-TB	Number	2019	40	6	135
Incidence	Spain	Both	All ages	MDR-TB	Number	2019	39	5	141
Incidence	Syrian Arab Republic	Both	All ages	MDR-TB	Number	2019	35	5	146
Incidence	Haiti	Both	All ages	MDR-TB	Number	2019	35	4	134
Incidence	Lao People's Democratic Republic	Both	All ages	MDR-TB	Number	2019	35	4	131
Incidence	Sri Lanka	Both	All ages	MDR-TB	Number	2019	34	3	132
Incidence	Estonia	Both	All ages	MDR-TB	Number	2019	34	18	57
Incidence	Panama	Both	All ages	MDR-TB	Number	2019	31	4	119
Incidence	Bhutan	Both	All ages	MDR-TB	Number	2019	29	4	96
Incidence	Bulgaria	Both	All ages	MDR-TB	Number	2019	28	8	68
Incidence	Sweden	Both	All ages	MDR-TB	Number	2019	27	10	59
Incidence	Nicaragua	Both	All ages	MDR-TB	Number	2019	25	4	72
Incidence	Dominican Republic	Both	All ages	MDR-TB	Number	2019	24	2	116
Incidence	Singapore	Both	All ages	MDR-TB	Number	2019	24	10	47
Incidence	Chile	Both	All ages	MDR-TB	Number	2019	22	7	52
Incidence	Tunisia	Both	All ages	MDR-TB	Number	2019	22	7	54
Incidence	Canada	Both	All ages	MDR-TB	Number	2019	22	8	50
Incidence	Switzerland	Both	All ages	MDR-TB	Number	2019	20	8	42
Incidence	Portugal	Both	All ages	MDR-TB	Number	2019	20	5	53
Incidence	Libya	Both	All ages	MDR-TB	Number	2019	20	2	72
Incidence	Jordan	Both	All ages	MDR-TB	Number	2019	19	3	67
Incidence	Cabo Verde	Both	All ages	MDR-TB	Number	2019	17	2	51
Incidence	Austria	Both	All ages	MDR-TB	Number	2019	17	6	35
Incidence	Israel	Both	All ages	MDR-TB	Number	2019	16	5	38
Incidence	Cuba	Both	All ages	MDR-TB	Number	2019	16	3	44
Incidence	Netherlands	Both	All ages	MDR-TB	Number	2019	15	6	33
Incidence	Belgium	Both	All ages	MDR-TB	Number	2019	15	5	36
Incidence	El Salvador	Both	All ages	MDR-TB	Number	2019	14	2	53
Incidence	Greece	Both	All ages	MDR-TB	Number	2019	14	2	42
Incidence	Kuwait	Both	All ages	MDR-TB	Number	2019	13	4	34
Incidence	Lebanon	Both	All ages	MDR-TB	Number	2019	12	1	49
Incidence	Oman	Both	All ages	MDR-TB	Number	2019	11	2	32
Incidence	Czechia	Both	All ages	MDR-TB	Number	2019	11	4	24
Incidence	Hungary	Both	All ages	MDR-TB	Number	2019	9	2	30
Incidence	Finland	Both	All ages	MDR-TB	Number	2019	9	3	22
Incidence	New Zealand	Both	All ages	MDR-TB	Number	2019	8	2	25
Incidence	Serbia	Both	All ages	MDR-TB	Number	2019	8	2	20
Incidence	Norway	Both	All ages	MDR-TB	Number	2019	8	2	20
Incidence	Costa Rica	Both	All ages	MDR-TB	Number	2019	8	1	26
Incidence	Qatar	Both	All ages	MDR-TB	Number	2019	7	1	23
Incidence	Bahrain	Both	All ages	MDR-TB	Number	2019	7	1	21
Incidence	Timor-Leste	Both	All ages	MDR-TB	Number	2019	7	1	26
Incidence	Palestine	Both	All ages	MDR-TB	Number	2019	6	1	23
Incidence	Denmark	Both	All ages	MDR-TB	Number	2019	5	1	13

Incidence	Sao Tome and Principe	Both	All ages	MDR-TB	Number	2019	5	1	14
Incidence	Ireland	Both	All ages	MDR-TB	Number	2019	4	1	11
Incidence	North Macedonia	Both	All ages	MDR-TB	Number	2019	4	1	9
Incidence	Bosnia and Herzegovina	Both	All ages	MDR-TB	Number	2019	3	1	7
Incidence	Guyana	Both	All ages	MDR-TB	Number	2019	3	0	13
Incidence	Slovakia	Both	All ages	MDR-TB	Number	2019	2	0	7
Incidence	Uruguay	Both	All ages	MDR-TB	Number	2019	2	0	7
Incidence	Jamaica	Both	All ages	MDR-TB	Number	2019	2	0	6
Incidence	Puerto Rico	Both	All ages	MDR-TB	Number	2019	2	0	5
Incidence	Croatia	Both	All ages	MDR-TB	Number	2019	1	0	4
Incidence	Luxembourg	Both	All ages	MDR-TB	Number	2019	1	0	3
Incidence	Trinidad and Tobago	Both	All ages	MDR-TB	Number	2019	1	0	4
Incidence	Mauritius	Both	All ages	MDR-TB	Number	2019	1	0	2
Incidence	Cyprus	Both	All ages	MDR-TB	Number	2019	1	0	2
Incidence	Solomon Islands	Both	All ages	MDR-TB	Number	2019	1	0	3
Incidence	Bahamas	Both	All ages	MDR-TB	Number	2019	1	0	3
Incidence	Albania	Both	All ages	MDR-TB	Number	2019	1	0	2
Incidence	Kiribati	Both	All ages	MDR-TB	Number	2019	1	0	3
Incidence	Brunei Darussalam	Both	All ages	MDR-TB	Number	2019	1	0	2
Incidence	Marshall Islands	Both	All ages	MDR-TB	Number	2019	1	0	2
Incidence	Maldives	Both	All ages	MDR-TB	Number	2019	1	0	3
Incidence	Greenland	Both	All ages	MDR-TB	Number	2019	1	0	2
Incidence	Fiji	Both	All ages	MDR-TB	Number	2019	1	0	2
Incidence	Belize	Both	All ages	MDR-TB	Number	2019	0	0	2
Incidence	Montenegro	Both	All ages	MDR-TB	Number	2019	0	0	1
Incidence	Vanuatu	Both	All ages	MDR-TB	Number	2019	0	0	1
Incidence	Malta	Both	All ages	MDR-TB	Number	2019	0	0	1
Incidence	Suriname	Both	All ages	MDR-TB	Number	2019	0	0	1
Incidence	Guam	Both	All ages	MDR-TB	Number	2019	0	0	1
Incidence	Northern Mariana Islands	Both	All ages	MDR-TB	Number	2019	0	0	1
Incidence	Micronesia (Federated States of)	Both	All ages	MDR-TB	Number	2019	0	0	1
Incidence	Iceland	Both	All ages	MDR-TB	Number	2019	0	0	1
Incidence	Slovenia	Both	All ages	MDR-TB	Number	2019	0	0	0
Incidence	Antigua and Barbuda	Both	All ages	MDR-TB	Number	2019	0	0	1
Incidence	Dominica	Both	All ages	MDR-TB	Number	2019	0	0	1
Incidence	Saint Vincent and the Grenadines	Both	All ages	MDR-TB	Number	2019	0	0	0
Incidence	Saint Lucia	Both	All ages	MDR-TB	Number	2019	0	0	0
Incidence	Grenada	Both	All ages	MDR-TB	Number	2019	0	0	0
Incidence	Tonga	Both	All ages	MDR-TB	Number	2019	0	0	0
Incidence	Saint Kitts and Nevis	Both	All ages	MDR-TB	Number	2019	0	0	0
Incidence	Seychelles	Both	All ages	MDR-TB	Number	2019	0	0	0
Incidence	Barbados	Both	All ages	MDR-TB	Number	2019	0	0	0
Incidence	Samoa	Both	All ages	MDR-TB	Number	2019	0	0	0
Incidence	Bermuda	Both	All ages	MDR-TB	Number	2019	0	0	0
Incidence	Monaco	Both	All ages	MDR-TB	Number	2019	0	0	0
Incidence	United States Virgin Islands	Both	All ages	MDR-TB	Number	2019	0	0	0
Incidence	Palau	Both	All ages	MDR-TB	Number	2019	0	0	0
Incidence	American Samoa	Both	All ages	MDR-TB	Number	2019	0	0	0
Incidence	Tuvalu	Both	All ages	MDR-TB	Number	2019	0	0	0
Incidence	San Marino	Both	All ages	MDR-TB	Number	2019	0	0	0
Incidence	Nauru	Both	All ages	MDR-TB	Number	2019	0	0	0

Incidence	Cook Islands	Both	All ages	MDR-TB	Number	2019	0	0	0
Incidence	Andorra	Both	All ages	MDR-TB	Number	2019	0	0	0
Incidence	Niue	Both	All ages	MDR-TB	Number	2019	0	0	0
Incidence	Tokelau	Both	All ages	MDR-TB	Number	2019	0	0	0

Figure 1B Age-standardised incidence rate of MDR-TB among HIV-negative individuals in 204 countries and territories, 2019

measure	location	sex	age	cause	metric	year	value (per 100,000)	95% UI (lower)	95%UI (upper)
Incidence	Eswatini	Both	Age-standardized	MDR-TB	Rate	2019	33.9	8.1	83.2
Incidence	Somalia	Both	Age-standardized	MDR-TB	Rate	2019	30.4	8.7	87.5
Incidence	Comoros	Both	Age-standardized	MDR-TB	Rate	2019	26.6	3.4	87
Incidence	Ukraine	Both	Age-standardized	MDR-TB	Rate	2019	23	12.3	36.2
Incidence	Kyrgyzstan	Both	Age-standardized	MDR-TB	Rate	2019	22.1	7.8	41
Incidence	Republic of Moldova	Both	Age-standardized	MDR-TB	Rate	2019	22	14.9	30.6
Incidence	Lesotho	Both	Age-standardized	MDR-TB	Rate	2019	19.1	6.6	41.9
Incidence	Russian Federation	Both	Age-standardized	MDR-TB	Rate	2019	18.7	9.9	29.8
Incidence	Namibia	Both	Age-standardized	MDR-TB	Rate	2019	18.7	7.6	36.2
Incidence	Eritrea	Both	Age-standardized	MDR-TB	Rate	2019	18.7	2.8	62
Incidence	Botswana	Both	Age-standardized	MDR-TB	Rate	2019	16.5	3.1	50.1
Incidence	India	Both	Age-standardized	MDR-TB	Rate	2019	16.1	3	40.3
Incidence	Mozambique	Both	Age-standardized	MDR-TB	Rate	2019	15.7	5.1	33.7
Incidence	Djibouti	Both	Age-standardized	MDR-TB	Rate	2019	15.5	6.6	31.5
Incidence	Burundi	Both	Age-standardized	MDR-TB	Rate	2019	14.7	2.1	50.5
Incidence	Pakistan	Both	Age-standardized	MDR-TB	Rate	2019	14.1	4.5	33.7
Incidence	Uzbekistan	Both	Age-standardized	MDR-TB	Rate	2019	13.2	4.9	24.8
Incidence	Zimbabwe	Both	Age-standardized	MDR-TB	Rate	2019	13	1.9	43.4
Incidence	Kazakhstan	Both	Age-standardized	MDR-TB	Rate	2019	11.9	6.4	19.2
Incidence	South Sudan	Both	Age-standardized	MDR-TB	Rate	2019	10.8	1.7	33.6
Incidence	Myanmar	Both	Age-standardized	MDR-TB	Rate	2019	10.8	3.8	22.5
Incidence	South Africa	Both	Age-standardized	MDR-TB	Rate	2019	10	3.5	23.2
Incidence	Georgia	Both	Age-standardized	MDR-TB	Rate	2019	10	5.7	15.7
Incidence	Azerbaijan	Both	Age-standardized	MDR-TB	Rate	2019	9.8	3.1	18.8
Incidence	Madagascar	Both	Age-standardized	MDR-TB	Rate	2019	9.4	1.4	28.2
Incidence	Zambia	Both	Age-standardized	MDR-TB	Rate	2019	9.3	1.5	32.3
Incidence	Philippines	Both	Age-standardized	MDR-TB	Rate	2019	9	2.5	21.3
Incidence	Mongolia	Both	Age-standardized	MDR-TB	Rate	2019	8.9	1.8	24.9
Incidence	Belarus	Both	Age-standardized	MDR-TB	Rate	2019	8.7	5.9	12.1
Incidence	Ethiopia	Both	Age-standardized	MDR-TB	Rate	2019	8.1	1.5	27.5
Incidence	Nepal	Both	Age-standardized	MDR-TB	Rate	2019	7.9	1.9	20.8
Incidence	Turkmenistan	Both	Age-standardized	MDR-TB	Rate	2019	7.7	2.8	15.3
Incidence	Nigeria	Both	Age-standardized	MDR-TB	Rate	2019	7.7	1.7	21
Incidence	Angola	Both	Age-standardized	MDR-TB	Rate	2019	7.6	1	27
Incidence	Tajikistan	Both	Age-standardized	MDR-TB	Rate	2019	7.6	3.5	13.7
Incidence	Democratic Republic of the Congo	Both	Age-standardized	MDR-TB	Rate	2019	7.5	1	27.4
Incidence	Central African Republic	Both	Age-standardized	MDR-TB	Rate	2019	7.4	1.2	24.3
Incidence	Gabon	Both	Age-standardized	MDR-TB	Rate	2019	7.3	1	26.6
Incidence	Uganda	Both	Age-standardized	MDR-TB	Rate	2019	6.9	1.4	17.8
Incidence	Rwanda	Both	Age-standardized	MDR-TB	Rate	2019	6.2	2.4	12.8
Incidence	United Republic of Tanzania	Both	Age-standardized	MDR-TB	Rate	2019	6.1	1.1	20.9
Incidence	Viet Nam	Both	Age-standardized	MDR-TB	Rate	2019	6	1.8	14.4
Incidence	Lithuania	Both	Age-standardized	MDR-TB	Rate	2019	5.9	3.4	9
Incidence	Chad	Both	Age-standardized	MDR-TB	Rate	2019	5.7	0.8	17.4
Incidence	Burkina Faso	Both	Age-standardized	MDR-TB	Rate	2019	5.6	0.8	18.7
Incidence	Peru	Both	Age-standardized	MDR-TB	Rate	2019	5.6	2.8	10.3
Incidence	Côte d'Ivoire	Both	Age-standardized	MDR-TB	Rate	2019	5.5	0.8	18.1
Incidence	Gambia	Both	Age-standardized	MDR-TB	Rate	2019	5.4	0.7	17.7
Incidence	Sierra Leone	Both	Age-standardized	MDR-TB	Rate	2019	5.3	0.7	16.9
Incidence	Ghana	Both	Age-standardized	MDR-TB	Rate	2019	5.1	0.8	14.7

Incidence	Bangladesh	Both	Age-standardized	MDR-TB	Rate	2019	5	1.2	12.4
Incidence	Malawi	Both	Age-standardized	MDR-TB	Rate	2019	5	1	15
Incidence	Democratic People's Republic of Korea	Both	Age-standardized	MDR-TB	Rate	2019	4.8	1.5	11
Incidence	Congo	Both	Age-standardized	MDR-TB	Rate	2019	4.7	0.6	16.6
Incidence	Papua New Guinea	Both	Age-standardized	MDR-TB	Rate	2019	4.7	1.7	10.6
Incidence	Togo	Both	Age-standardized	MDR-TB	Rate	2019	4.4	0.7	12.3
Incidence	Mali	Both	Age-standardized	MDR-TB	Rate	2019	4.3	0.5	13.2
Incidence	Kenya	Both	Age-standardized	MDR-TB	Rate	2019	4.2	1.4	9.9
Incidence	Bolivia (Plurinational State of)	Both	Age-standardized	MDR-TB	Rate	2019	4.1	0.6	13.3
Incidence	Equatorial Guinea	Both	Age-standardized	MDR-TB	Rate	2019	4	0.5	13
Incidence	Bhutan	Both	Age-standardized	MDR-TB	Rate	2019	4	0.6	13.2
Incidence	Guinea	Both	Age-standardized	MDR-TB	Rate	2019	3.9	0.5	12.7
Incidence	Niger	Both	Age-standardized	MDR-TB	Rate	2019	3.9	0.5	12.1
Incidence	Afghanistan	Both	Age-standardized	MDR-TB	Rate	2019	3.7	0.6	12.4
Incidence	Senegal	Both	Age-standardized	MDR-TB	Rate	2019	3.5	1.1	7.8
Incidence	Cameroon	Both	Age-standardized	MDR-TB	Rate	2019	3.5	0.5	10.7
Incidence	Armenia	Both	Age-standardized	MDR-TB	Rate	2019	3.5	0.7	8.2
Incidence	Liberia	Both	Age-standardized	MDR-TB	Rate	2019	3.3	0.4	13.1
Incidence	Cabo Verde	Both	Age-standardized	MDR-TB	Rate	2019	3.1	0.4	9.5
Incidence	Guinea-Bissau	Both	Age-standardized	MDR-TB	Rate	2019	2.8	0.4	9.1
Incidence	Sao Tome and Principe	Both	Age-standardized	MDR-TB	Rate	2019	2.8	0.4	8.6
Incidence	Benin	Both	Age-standardized	MDR-TB	Rate	2019	2.8	0.5	7.7
Incidence	Thailand	Both	Age-standardized	MDR-TB	Rate	2019	2.7	0.8	6.8
Incidence	Cambodia	Both	Age-standardized	MDR-TB	Rate	2019	2.2	0.3	7.4
Incidence	Estonia	Both	Age-standardized	MDR-TB	Rate	2019	2.2	1.1	3.8
Incidence	Latvia	Both	Age-standardized	MDR-TB	Rate	2019	2.2	1.1	3.9
Incidence	Mauritania	Both	Age-standardized	MDR-TB	Rate	2019	2	0.2	6.3
Incidence	China	Both	Age-standardized	MDR-TB	Rate	2019	1.8	0.3	5.6
Incidence	Ecuador	Both	Age-standardized	MDR-TB	Rate	2019	1.8	0.3	5.4
Incidence	Morocco	Both	Age-standardized	MDR-TB	Rate	2019	1.6	0.5	3.8
Incidence	Romania	Both	Age-standardized	MDR-TB	Rate	2019	1.3	0.5	2.6
Incidence	Marshall Islands	Both	Age-standardized	MDR-TB	Rate	2019	1.2	0.2	4
Incidence	Saudi Arabia	Both	Age-standardized	MDR-TB	Rate	2019	1.2	0.2	3.5
Incidence	Paraguay	Both	Age-standardized	MDR-TB	Rate	2019	1	0.1	3.3
Incidence	Iraq	Both	Age-standardized	MDR-TB	Rate	2019	1	0.3	2.3
Incidence	Brazil	Both	Age-standardized	MDR-TB	Rate	2019	0.9	0.2	2.4
Incidence	Greenland	Both	Age-standardized	MDR-TB	Rate	2019	0.8	0.1	2.9
Incidence	Taiwan (Province of China)	Both	Age-standardized	MDR-TB	Rate	2019	0.8	0.1	3
Incidence	Malaysia	Both	Age-standardized	MDR-TB	Rate	2019	0.8	0.2	2.2
Incidence	Colombia	Both	Age-standardized	MDR-TB	Rate	2019	0.8	0.1	2.1
Incidence	Honduras	Both	Age-standardized	MDR-TB	Rate	2019	0.8	0.1	2.5
Incidence	Panama	Both	Age-standardized	MDR-TB	Rate	2019	0.7	0.1	2.8
Incidence	Yemen	Both	Age-standardized	MDR-TB	Rate	2019	0.7	0.2	2.6
Incidence	Turkey	Both	Age-standardized	MDR-TB	Rate	2019	0.6	0.3	1.3
Incidence	Sudan	Both	Age-standardized	MDR-TB	Rate	2019	0.6	0.1	2.6
Incidence	Kiribati	Both	Age-standardized	MDR-TB	Rate	2019	0.6	0.1	2.5
Incidence	Egypt	Both	Age-standardized	MDR-TB	Rate	2019	0.6	0.1	1.6
Incidence	Timor-Leste	Both	Age-standardized	MDR-TB	Rate	2019	0.6	0.1	2.5
Incidence	Lao People's Democratic Republic	Both	Age-standardized	MDR-TB	Rate	2019	0.6	0.1	2.1
Incidence	Republic of Korea	Both	Age-standardized	MDR-TB	Rate	2019	0.6	0.1	2.3
Incidence	Indonesia	Both	Age-standardized	MDR-TB	Rate	2019	0.5	0.1	2
Incidence	Mexico	Both	Age-standardized	MDR-TB	Rate	2019	0.5	0.1	1.4
Incidence	Bahrain	Both	Age-standardized	MDR-TB	Rate	2019	0.5	0.1	1.4
Incidence	United Arab Emirates	Both	Age-standardized	MDR-TB	Rate	2019	0.4	0	1.7
Incidence	Venezuela (Bolivarian Republic of)	Both	Age-standardized	MDR-TB	Rate	2019	0.4	0.1	1.5

Incidence	Nicaragua	Both	Age-standardized	MDR-TB	Rate	2019	0.4	0.1	1.2
Incidence	Guatemala	Both	Age-standardized	MDR-TB	Rate	2019	0.4	0.1	1.3
Incidence	Northern Mariana Islands	Both	Age-standardized	MDR-TB	Rate	2019	0.4	0.1	1.4
Incidence	Algeria	Both	Age-standardized	MDR-TB	Rate	2019	0.3	0	1.6
Incidence	Bulgaria	Both	Age-standardized	MDR-TB	Rate	2019	0.3	0.1	0.8
Incidence	Singapore	Both	Age-standardized	MDR-TB	Rate	2019	0.3	0.1	0.7
Incidence	Guyana	Both	Age-standardized	MDR-TB	Rate	2019	0.3	0	1.7
Incidence	Haiti	Both	Age-standardized	MDR-TB	Rate	2019	0.3	0	1.1
Incidence	Libya	Both	Age-standardized	MDR-TB	Rate	2019	0.3	0	1.1
Incidence	Kuwait	Both	Age-standardized	MDR-TB	Rate	2019	0.3	0.1	0.7
Incidence	Oman	Both	Age-standardized	MDR-TB	Rate	2019	0.3	0.1	0.8
Incidence	Sweden	Both	Age-standardized	MDR-TB	Rate	2019	0.3	0.1	0.6
Incidence	Qatar	Both	Age-standardized	MDR-TB	Rate	2019	0.3	0.1	0.9
Incidence	Syrian Arab Republic	Both	Age-standardized	MDR-TB	Rate	2019	0.2	0	1
Incidence	Switzerland	Both	Age-standardized	MDR-TB	Rate	2019	0.2	0.1	0.5
Incidence	Germany	Both	Age-standardized	MDR-TB	Rate	2019	0.2	0.1	0.5
Incidence	Dominican Republic	Both	Age-standardized	MDR-TB	Rate	2019	0.2	0	1.1
Incidence	El Salvador	Both	Age-standardized	MDR-TB	Rate	2019	0.2	0	0.8
Incidence	Lebanon	Both	Age-standardized	MDR-TB	Rate	2019	0.2	0	0.9
Incidence	Italy	Both	Age-standardized	MDR-TB	Rate	2019	0.2	0.1	0.4
Incidence	Iran (Islamic Republic of)	Both	Age-standardized	MDR-TB	Rate	2019	0.2	0.1	0.5
Incidence	Micronesia (Federated States of)	Both	Age-standardized	MDR-TB	Rate	2019	0.2	0	0.9
Incidence	Jordan	Both	Age-standardized	MDR-TB	Rate	2019	0.2	0	0.7
Incidence	New Zealand	Both	Age-standardized	MDR-TB	Rate	2019	0.2	0	0.6
Incidence	Argentina	Both	Age-standardized	MDR-TB	Rate	2019	0.2	0	0.7
Incidence	Austria	Both	Age-standardized	MDR-TB	Rate	2019	0.2	0.1	0.4
Incidence	Bahamas	Both	Age-standardized	MDR-TB	Rate	2019	0.2	0	0.7
Incidence	Nauru	Both	Age-standardized	MDR-TB	Rate	2019	0.2	0	0.8
Incidence	Tunisia	Both	Age-standardized	MDR-TB	Rate	2019	0.2	0.1	0.4
Incidence	Israel	Both	Age-standardized	MDR-TB	Rate	2019	0.2	0.1	0.4
Incidence	Portugal	Both	Age-standardized	MDR-TB	Rate	2019	0.2	0	0.4
Incidence	Tuvalu	Both	Age-standardized	MDR-TB	Rate	2019	0.2	0	0.8
Incidence	Brunei Darussalam	Both	Age-standardized	MDR-TB	Rate	2019	0.2	0	0.5
Incidence	Australia	Both	Age-standardized	MDR-TB	Rate	2019	0.2	0.1	0.3
Incidence	Dominica	Both	Age-standardized	MDR-TB	Rate	2019	0.2	0	0.7
Incidence	Luxembourg	Both	Age-standardized	MDR-TB	Rate	2019	0.2	0	0.6
Incidence	Costa Rica	Both	Age-standardized	MDR-TB	Rate	2019	0.1	0	0.5
Incidence	Sri Lanka	Both	Age-standardized	MDR-TB	Rate	2019	0.1	0	0.5
Incidence	Finland	Both	Age-standardized	MDR-TB	Rate	2019	0.1	0	0.4
Incidence	Palestine	Both	Age-standardized	MDR-TB	Rate	2019	0.1	0	0.6
Incidence	North Macedonia	Both	Age-standardized	MDR-TB	Rate	2019	0.1	0	0.3
Incidence	Norway	Both	Age-standardized	MDR-TB	Rate	2019	0.1	0	0.4
Incidence	Guam	Both	Age-standardized	MDR-TB	Rate	2019	0.1	0	0.5
Incidence	Solomon Islands	Both	Age-standardized	MDR-TB	Rate	2019	0.1	0	0.5
Incidence	Antigua and Barbuda	Both	Age-standardized	MDR-TB	Rate	2019	0.1	0	0.6
Incidence	Maldives	Both	Age-standardized	MDR-TB	Rate	2019	0.1	0	0.5
Incidence	Belgium	Both	Age-standardized	MDR-TB	Rate	2019	0.1	0	0.3
Incidence	Belize	Both	Age-standardized	MDR-TB	Rate	2019	0.1	0	0.5
Incidence	France	Both	Age-standardized	MDR-TB	Rate	2019	0.1	0	0.3
Incidence	Tokelau	Both	Age-standardized	MDR-TB	Rate	2019	0.1	0	0.6
Incidence	Palau	Both	Age-standardized	MDR-TB	Rate	2019	0.1	0	0.5
Incidence	Vanuatu	Both	Age-standardized	MDR-TB	Rate	2019	0.1	0	0.5
Incidence	Monaco	Both	Age-standardized	MDR-TB	Rate	2019	0.1	0	0.4
Incidence	United Kingdom	Both	Age-standardized	MDR-TB	Rate	2019	0.1	0	0.3
Incidence	Cuba	Both	Age-standardized	MDR-TB	Rate	2019	0.1	0	0.3

Incidence	Greece	Both	Age-standardized	MDR-TB	Rate	2019	0.1	0	0.4
Incidence	Saint Kitts and Nevis	Both	Age-standardized	MDR-TB	Rate	2019	0.1	0	0.5
Incidence	Chile	Both	Age-standardized	MDR-TB	Rate	2019	0.1	0	0.3
Incidence	Niue	Both	Age-standardized	MDR-TB	Rate	2019	0.1	0	0.4
Incidence	Tonga	Both	Age-standardized	MDR-TB	Rate	2019	0.1	0	0.4
Incidence	Poland	Both	Age-standardized	MDR-TB	Rate	2019	0.1	0	0.2
Incidence	Saint Vincent and the Grenadines	Both	Age-standardized	MDR-TB	Rate	2019	0.1	0	0.3
Incidence	Netherlands	Both	Age-standardized	MDR-TB	Rate	2019	0.1	0	0.2
Incidence	Bermuda	Both	Age-standardized	MDR-TB	Rate	2019	0.1	0	0.5
Incidence	Grenada	Both	Age-standardized	MDR-TB	Rate	2019	0.1	0	0.4
Incidence	Denmark	Both	Age-standardized	MDR-TB	Rate	2019	0.1	0	0.2
Incidence	Spain	Both	Age-standardized	MDR-TB	Rate	2019	0.1	0	0.3
Incidence	Czechia	Both	Age-standardized	MDR-TB	Rate	2019	0.1	0	0.2
Incidence	Ireland	Both	Age-standardized	MDR-TB	Rate	2019	0.1	0	0.2
Incidence	Hungary	Both	Age-standardized	MDR-TB	Rate	2019	0.1	0	0.2
Incidence	Serbia	Both	Age-standardized	MDR-TB	Rate	2019	0.1	0	0.2
Incidence	Malta	Both	Age-standardized	MDR-TB	Rate	2019	0.1	0	0.3
Incidence	Seychelles	Both	Age-standardized	MDR-TB	Rate	2019	0.1	0	0.3
Incidence	Fiji	Both	Age-standardized	MDR-TB	Rate	2019	0.1	0	0.3
Incidence	Bosnia and Herzegovina	Both	Age-standardized	MDR-TB	Rate	2019	0.1	0	0.2
Incidence	Jamaica	Both	Age-standardized	MDR-TB	Rate	2019	0.1	0	0.2
Incidence	Uruguay	Both	Age-standardized	MDR-TB	Rate	2019	0.1	0	0.2
Incidence	Saint Lucia	Both	Age-standardized	MDR-TB	Rate	2019	0.1	0	0.2
Incidence	Japan	Both	Age-standardized	MDR-TB	Rate	2019	0.1	0	0.2
Incidence	Trinidad and Tobago	Both	Age-standardized	MDR-TB	Rate	2019	0.1	0	0.2
Incidence	Montenegro	Both	Age-standardized	MDR-TB	Rate	2019	0.1	0	0.2
Incidence	Canada	Both	Age-standardized	MDR-TB	Rate	2019	0.1	0	0.1
Incidence	Iceland	Both	Age-standardized	MDR-TB	Rate	2019	0.1	0	0.2
Incidence	Cyprus	Both	Age-standardized	MDR-TB	Rate	2019	0.1	0	0.2
Incidence	Mauritius	Both	Age-standardized	MDR-TB	Rate	2019	0.1	0	0.2
Incidence	Cook Islands	Both	Age-standardized	MDR-TB	Rate	2019	0	0	0.2
Incidence	San Marino	Both	Age-standardized	MDR-TB	Rate	2019	0	0	0.2
Incidence	Suriname	Both	Age-standardized	MDR-TB	Rate	2019	0	0	0.2
Incidence	American Samoa	Both	Age-standardized	MDR-TB	Rate	2019	0	0	0.2
Incidence	Puerto Rico	Both	Age-standardized	MDR-TB	Rate	2019	0	0	0.1
Incidence	Samoa	Both	Age-standardized	MDR-TB	Rate	2019	0	0	0.1
Incidence	United States of America	Both	Age-standardized	MDR-TB	Rate	2019	0	0	0.1
Incidence	Slovakia	Both	Age-standardized	MDR-TB	Rate	2019	0	0	0.1
Incidence	United States Virgin Islands	Both	Age-standardized	MDR-TB	Rate	2019	0	0	0.1
Incidence	Croatia	Both	Age-standardized	MDR-TB	Rate	2019	0	0	0.1
Incidence	Albania	Both	Age-standardized	MDR-TB	Rate	2019	0	0	0.1
Incidence	Barbados	Both	Age-standardized	MDR-TB	Rate	2019	0	0	0.1
Incidence	Andorra	Both	Age-standardized	MDR-TB	Rate	2019	0	0	0
Incidence	Slovenia	Both	Age-standardized	MDR-TB	Rate	2019	0	0	0

Figure 2A Age-specific distribution of DALYs from level 2 GBD causes associated with alcohol use in 1990 and 2019

measure	location	sex	age	cause	risk factor	metric	year	value	95% UI (lower)	95% UI (upper)
DALYs	Global	Female	15-19 years	Digestive diseases	Alcohol use	Number	1990	24843	17995	34104
DALYs	Global	Male	15-19 years	Digestive diseases	Alcohol use	Number	1990	72988	53324	96596
DALYs	Global	Female	15-19 years	Neoplasms	Alcohol use	Number	1990	2668	2234	3150
DALYs	Global	Male	15-19 years	Neoplasms	Alcohol use	Number	1990	9444	7279	12209
DALYs	Global	Female	15-19 years	Neurological disorders	Alcohol use	Number	1990	21367	12483	35154
DALYs	Global	Male	15-19 years	Neurological disorders	Alcohol use	Number	1990	54994	34358	85585
DALYs	Global	Female	15-19 years	Respiratory infections and tuberculosis	Alcohol use	Number	1990	35739	20176	53080
DALYs	Global	Male	15-19 years	Respiratory infections and tuberculosis	Alcohol use	Number	1990	99040	60783	139510
DALYs	Global	Female	15-19 years	Self-harm and interpersonal violence	Alcohol use	Number	1990	48769	27047	72609
DALYs	Global	Male	15-19 years	Self-harm and interpersonal violence	Alcohol use	Number	1990	345927	221046	478963
DALYs	Global	Female	15-19 years	Substance use disorders	Alcohol use	Number	1990	126135	79749	187792
DALYs	Global	Male	15-19 years	Substance use disorders	Alcohol use	Number	1990	319779	204193	473730
DALYs	Global	Female	15-19 years	Transport injuries	Alcohol use	Number	1990	44395	23008	69901
DALYs	Global	Male	15-19 years	Transport injuries	Alcohol use	Number	1990	317777	171072	487675
DALYs	Global	Female	15-19 years	Unintentional injuries	Alcohol use	Number	1990	21274	8901	39355
DALYs	Global	Male	15-19 years	Unintentional injuries	Alcohol use	Number	1990	128536	58160	228320
DALYs	Global	Female	20-24 years	Digestive diseases	Alcohol use	Number	1990	49692	37104	66497
DALYs	Global	Male	20-24 years	Digestive diseases	Alcohol use	Number	1990	237431	176034	310653
DALYs	Global	Female	20-24 years	Neoplasms	Alcohol use	Number	1990	8494	7295	9909
DALYs	Global	Male	20-24 years	Neoplasms	Alcohol use	Number	1990	36240	28675	44529
DALYs	Global	Female	20-24 years	Neurological disorders	Alcohol use	Number	1990	30039	18343	47758
DALYs	Global	Male	20-24 years	Neurological disorders	Alcohol use	Number	1990	102227	68352	147041
DALYs	Global	Female	20-24 years	Respiratory infections and tuberculosis	Alcohol use	Number	1990	100368	61536	144034
DALYs	Global	Male	20-24 years	Respiratory infections and tuberculosis	Alcohol use	Number	1990	419302	280839	561049
DALYs	Global	Female	20-24 years	Self-harm and interpersonal violence	Alcohol use	Number	1990	95541	55172	141235
DALYs	Global	Male	20-24 years	Self-harm and interpersonal violence	Alcohol use	Number	1990	902260	600682	1232609
DALYs	Global	Female	20-24 years	Substance use disorders	Alcohol use	Number	1990	282168	173555	450379
DALYs	Global	Male	20-24 years	Substance use disorders	Alcohol use	Number	1990	915729	593403	1370792
DALYs	Global	Female	20-24 years	Transport injuries	Alcohol use	Number	1990	59761	31236	93418
DALYs	Global	Male	20-24 years	Transport injuries	Alcohol use	Number	1990	865112	473842	1259053
DALYs	Global	Female	20-24 years	Unintentional injuries	Alcohol use	Number	1990	32447	13881	59324
DALYs	Global	Male	20-24 years	Unintentional injuries	Alcohol use	Number	1990	260399	123098	454904
DALYs	Global	Female	25-29 years	Digestive diseases	Alcohol use	Number	1990	80568	59377	106139
DALYs	Global	Male	25-29 years	Digestive diseases	Alcohol use	Number	1990	476472	366548	593287
DALYs	Global	Female	25-29 years	Neoplasms	Alcohol use	Number	1990	19423	16910	21953
DALYs	Global	Male	25-29 years	Neoplasms	Alcohol use	Number	1990	57851	46033	70812
DALYs	Global	Female	25-29 years	Neurological disorders	Alcohol use	Number	1990	28731	17817	44367
DALYs	Global	Male	25-29 years	Neurological disorders	Alcohol use	Number	1990	101613	70607	142721
DALYs	Global	Female	25-29 years	Respiratory infections and tuberculosis	Alcohol use	Number	1990	120899	75895	169729
DALYs	Global	Male	25-29 years	Respiratory infections and tuberculosis	Alcohol use	Number	1990	627578	431757	832277
DALYs	Global	Female	25-29 years	Self-harm and interpersonal violence	Alcohol use	Number	1990	99667	58067	144876
DALYs	Global	Male	25-29 years	Self-harm and interpersonal violence	Alcohol use	Number	1990	983091	668261	1309117
DALYs	Global	Female	25-29 years	Substance use disorders	Alcohol use	Number	1990	326635	215092	473110
DALYs	Global	Male	25-29 years	Substance use disorders	Alcohol use	Number	1990	1247627	859854	1737646
DALYs	Global	Female	25-29 years	Transport injuries	Alcohol use	Number	1990	43638	22748	68822
DALYs	Global	Male	25-29 years	Transport injuries	Alcohol use	Number	1990	750399	423661	1088963
DALYs	Global	Female	25-29 years	Unintentional injuries	Alcohol use	Number	1990	35563	15902	65679

DALYs	Global	Male	25-29 years	Unintentional injuries	Alcohol use	Number	1990	277222	130189	479184
DALYs	Global	Female	30-34 years	Digestive diseases	Alcohol use	Number	1990	126882	97407	160048
DALYs	Global	Male	30-34 years	Digestive diseases	Alcohol use	Number	1990	824830	653205	999159
DALYs	Global	Female	30-34 years	Neoplasms	Alcohol use	Number	1990	47594	41006	53957
DALYs	Global	Male	30-34 years	Neoplasms	Alcohol use	Number	1990	116809	94655	144474
DALYs	Global	Female	30-34 years	Neurological disorders	Alcohol use	Number	1990	24003	14895	37312
DALYs	Global	Male	30-34 years	Neurological disorders	Alcohol use	Number	1990	89740	62537	125874
DALYs	Global	Female	30-34 years	Respiratory infections and tuberculosis	Alcohol use	Number	1990	114191	70474	163789
DALYs	Global	Male	30-34 years	Respiratory infections and tuberculosis	Alcohol use	Number	1990	747516	516145	965263
DALYs	Global	Female	30-34 years	Self-harm and interpersonal violence	Alcohol use	Number	1990	92900	52999	136888
DALYs	Global	Male	30-34 years	Self-harm and interpersonal violence	Alcohol use	Number	1990	876253	594975	1162842
DALYs	Global	Female	30-34 years	Substance use disorders	Alcohol use	Number	1990	324994	217110	478852
DALYs	Global	Male	30-34 years	Substance use disorders	Alcohol use	Number	1990	1410341	1043038	1940183
DALYs	Global	Female	30-34 years	Transport injuries	Alcohol use	Number	1990	23576	12342	37023
DALYs	Global	Male	30-34 years	Transport injuries	Alcohol use	Number	1990	455908	254981	685408
DALYs	Global	Female	30-34 years	Unintentional injuries	Alcohol use	Number	1990	37952	16563	70152
DALYs	Global	Male	30-34 years	Unintentional injuries	Alcohol use	Number	1990	276546	129042	468594
DALYs	Global	Female	35-39 years	Digestive diseases	Alcohol use	Number	1990	185895	140448	237909
DALYs	Global	Male	35-39 years	Digestive diseases	Alcohol use	Number	1990	1291456	1015422	1563468
DALYs	Global	Female	35-39 years	Neoplasms	Alcohol use	Number	1990	93266	80054	106822
DALYs	Global	Male	35-39 years	Neoplasms	Alcohol use	Number	1990	249921	201067	307548
DALYs	Global	Female	35-39 years	Neurological disorders	Alcohol use	Number	1990	20703	12495	31570
DALYs	Global	Male	35-39 years	Neurological disorders	Alcohol use	Number	1990	83816	58150	114698
DALYs	Global	Female	35-39 years	Respiratory infections and tuberculosis	Alcohol use	Number	1990	108392	66876	154595
DALYs	Global	Male	35-39 years	Respiratory infections and tuberculosis	Alcohol use	Number	1990	858657	592580	1110080
DALYs	Global	Female	35-39 years	Self-harm and interpersonal violence	Alcohol use	Number	1990	87008	48321	129227
DALYs	Global	Male	35-39 years	Self-harm and interpersonal violence	Alcohol use	Number	1990	759799	510681	1030280
DALYs	Global	Female	35-39 years	Substance use disorders	Alcohol use	Number	1990	316536	235319	428221
DALYs	Global	Male	35-39 years	Substance use disorders	Alcohol use	Number	1990	1422414	1118373	1836001
DALYs	Global	Female	35-39 years	Transport injuries	Alcohol use	Number	1990	19888	10422	31378
DALYs	Global	Male	35-39 years	Transport injuries	Alcohol use	Number	1990	304906	172236	463834
DALYs	Global	Female	35-39 years	Unintentional injuries	Alcohol use	Number	1990	39778	17195	74022
DALYs	Global	Male	35-39 years	Unintentional injuries	Alcohol use	Number	1990	282377	132453	482889
DALYs	Global	Female	40-44 years	Digestive diseases	Alcohol use	Number	1990	244217	185466	303741
DALYs	Global	Male	40-44 years	Digestive diseases	Alcohol use	Number	1990	1584977	1263513	1890755
DALYs	Global	Female	40-44 years	Neoplasms	Alcohol use	Number	1990	144596	124863	165307
DALYs	Global	Male	40-44 years	Neoplasms	Alcohol use	Number	1990	442416	369071	528384
DALYs	Global	Female	40-44 years	Neurological disorders	Alcohol use	Number	1990	17665	10460	26748
DALYs	Global	Male	40-44 years	Neurological disorders	Alcohol use	Number	1990	67657	47004	92277
DALYs	Global	Female	40-44 years	Respiratory infections and tuberculosis	Alcohol use	Number	1990	95946	60528	137957
DALYs	Global	Male	40-44 years	Respiratory infections and tuberculosis	Alcohol use	Number	1990	879134	613453	1126082
DALYs	Global	Female	40-44 years	Self-harm and interpersonal violence	Alcohol use	Number	1990	75231	41007	112459
DALYs	Global	Male	40-44 years	Self-harm and interpersonal violence	Alcohol use	Number	1990	581920	393292	777324
DALYs	Global	Female	40-44 years	Substance use disorders	Alcohol use	Number	1990	262834	200854	348922
DALYs	Global	Male	40-44 years	Substance use disorders	Alcohol use	Number	1990	1218936	974717	1544096
DALYs	Global	Female	40-44 years	Transport injuries	Alcohol use	Number	1990	28056	14590	44399
DALYs	Global	Male	40-44 years	Transport injuries	Alcohol use	Number	1990	221784	125953	333525
DALYs	Global	Female	40-44 years	Unintentional injuries	Alcohol use	Number	1990	39211	16981	71280
DALYs	Global	Male	40-44 years	Unintentional injuries	Alcohol use	Number	1990	250261	115971	425268
DALYs	Global	Female	45-49 years	Digestive diseases	Alcohol use	Number	1990	278266	209137	351748
DALYs	Global	Male	45-49 years	Digestive diseases	Alcohol use	Number	1990	1627226	1280173	1963980

DALYs	Global	Female	45-49 years	Neoplasms	Alcohol use	Number	1990	181190	155901	207475
DALYs	Global	Male	45-49 years	Neoplasms	Alcohol use	Number	1990	606308	519471	700812
DALYs	Global	Female	45-49 years	Neurological disorders	Alcohol use	Number	1990	13508	7947	20152
DALYs	Global	Male	45-49 years	Neurological disorders	Alcohol use	Number	1990	50813	35077	69164
DALYs	Global	Female	45-49 years	Respiratory infections and tuberculosis	Alcohol use	Number	1990	89440	54488	126355
DALYs	Global	Male	45-49 years	Respiratory infections and tuberculosis	Alcohol use	Number	1990	896066	615073	1174020
DALYs	Global	Female	45-49 years	Self-harm and interpersonal violence	Alcohol use	Number	1990	60321	33362	88568
DALYs	Global	Male	45-49 years	Self-harm and interpersonal violence	Alcohol use	Number	1990	425930	282357	575003
DALYs	Global	Female	45-49 years	Substance use disorders	Alcohol use	Number	1990	204807	165828	256491
DALYs	Global	Male	45-49 years	Substance use disorders	Alcohol use	Number	1990	971035	800201	1185163
DALYs	Global	Female	45-49 years	Transport injuries	Alcohol use	Number	1990	16687	8705	26467
DALYs	Global	Male	45-49 years	Transport injuries	Alcohol use	Number	1990	196138	111258	294694
DALYs	Global	Female	45-49 years	Unintentional injuries	Alcohol use	Number	1990	35152	15314	64676
DALYs	Global	Male	45-49 years	Unintentional injuries	Alcohol use	Number	1990	206369	93970	353202
DALYs	Global	Female	50-54 years	Digestive diseases	Alcohol use	Number	1990	345584	255624	439412
DALYs	Global	Male	50-54 years	Digestive diseases	Alcohol use	Number	1990	1763223	1396172	2109443
DALYs	Global	Female	50-54 years	Neoplasms	Alcohol use	Number	1990	231276	200579	264054
DALYs	Global	Male	50-54 years	Neoplasms	Alcohol use	Number	1990	877084	755416	1000568
DALYs	Global	Female	50-54 years	Neurological disorders	Alcohol use	Number	1990	11789	6925	17929
DALYs	Global	Male	50-54 years	Neurological disorders	Alcohol use	Number	1990	41757	28514	57264
DALYs	Global	Female	50-54 years	Respiratory infections and tuberculosis	Alcohol use	Number	1990	104575	63498	150188
DALYs	Global	Male	50-54 years	Respiratory infections and tuberculosis	Alcohol use	Number	1990	929156	646195	1193606
DALYs	Global	Female	50-54 years	Self-harm and interpersonal violence	Alcohol use	Number	1990	55357	29006	83953
DALYs	Global	Male	50-54 years	Self-harm and interpersonal violence	Alcohol use	Number	1990	373821	246081	499810
DALYs	Global	Female	50-54 years	Substance use disorders	Alcohol use	Number	1990	206257	168352	254048
DALYs	Global	Male	50-54 years	Substance use disorders	Alcohol use	Number	1990	884281	735712	1080965
DALYs	Global	Female	50-54 years	Transport injuries	Alcohol use	Number	1990	17620	9167	28442
DALYs	Global	Male	50-54 years	Transport injuries	Alcohol use	Number	1990	143792	81603	216823
DALYs	Global	Female	50-54 years	Unintentional injuries	Alcohol use	Number	1990	37758	16122	69869
DALYs	Global	Male	50-54 years	Unintentional injuries	Alcohol use	Number	1990	209555	96860	354573
DALYs	Global	Female	55-59 years	Digestive diseases	Alcohol use	Number	1990	383032	289897	491461
DALYs	Global	Male	55-59 years	Digestive diseases	Alcohol use	Number	1990	1652727	1307236	2000742
DALYs	Global	Female	55-59 years	Neoplasms	Alcohol use	Number	1990	250190	218513	285133
DALYs	Global	Male	55-59 years	Neoplasms	Alcohol use	Number	1990	1004247	877104	1145939
DALYs	Global	Female	55-59 years	Neurological disorders	Alcohol use	Number	1990	10064	5942	15399
DALYs	Global	Male	55-59 years	Neurological disorders	Alcohol use	Number	1990	34287	22678	48852
DALYs	Global	Female	55-59 years	Respiratory infections and tuberculosis	Alcohol use	Number	1990	101819	63292	145185
DALYs	Global	Male	55-59 years	Respiratory infections and tuberculosis	Alcohol use	Number	1990	860873	594171	1116964
DALYs	Global	Female	55-59 years	Self-harm and interpersonal violence	Alcohol use	Number	1990	42390	22393	65206
DALYs	Global	Male	55-59 years	Self-harm and interpersonal violence	Alcohol use	Number	1990	264321	169439	361154
DALYs	Global	Female	55-59 years	Substance use disorders	Alcohol use	Number	1990	152621	127367	185100
DALYs	Global	Male	55-59 years	Substance use disorders	Alcohol use	Number	1990	666755	556675	793949
DALYs	Global	Female	55-59 years	Transport injuries	Alcohol use	Number	1990	19368	10117	31447
DALYs	Global	Male	55-59 years	Transport injuries	Alcohol use	Number	1990	124716	70216	188835
DALYs	Global	Female	55-59 years	Unintentional injuries	Alcohol use	Number	1990	34788	15528	62840
DALYs	Global	Male	55-59 years	Unintentional injuries	Alcohol use	Number	1990	169629	78843	290982
DALYs	Global	Female	60-64 years	Digestive diseases	Alcohol use	Number	1990	383805	291160	494368
DALYs	Global	Male	60-64 years	Digestive diseases	Alcohol use	Number	1990	1410274	1117249	1703770
DALYs	Global	Female	60-64 years	Neoplasms	Alcohol use	Number	1990	258347	224301	293191
DALYs	Global	Male	60-64 years	Neoplasms	Alcohol use	Number	1990	985128	863668	1111983
DALYs	Global	Female	60-64 years	Neurological disorders	Alcohol use	Number	1990	9683	5622	15424

DALYs	Global	Male	60-64 years	Neurological disorders	Alcohol use	Number	1990	29827	19871	42888
DALYs	Global	Female	60-64 years	Respiratory infections and tuberculosis	Alcohol use	Number	1990	93150	58639	131651
DALYs	Global	Male	60-64 years	Respiratory infections and tuberculosis	Alcohol use	Number	1990	770454	535088	996019
DALYs	Global	Female	60-64 years	Self-harm and interpersonal violence	Alcohol use	Number	1990	34529	17934	53037
DALYs	Global	Male	60-64 years	Self-harm and interpersonal violence	Alcohol use	Number	1990	188606	120189	260036
DALYs	Global	Female	60-64 years	Substance use disorders	Alcohol use	Number	1990	117613	95916	148235
DALYs	Global	Male	60-64 years	Substance use disorders	Alcohol use	Number	1990	483090	395319	590673
DALYs	Global	Female	60-64 years	Transport injuries	Alcohol use	Number	1990	11143	5830	17873
DALYs	Global	Male	60-64 years	Transport injuries	Alcohol use	Number	1990	82624	46703	123930
DALYs	Global	Female	60-64 years	Unintentional injuries	Alcohol use	Number	1990	35374	15682	65610
DALYs	Global	Male	60-64 years	Unintentional injuries	Alcohol use	Number	1990	142413	65591	244560
DALYs	Global	Female	65-69 years	Digestive diseases	Alcohol use	Number	1990	317998	239073	401721
DALYs	Global	Male	65-69 years	Digestive diseases	Alcohol use	Number	1990	962575	769155	1156876
DALYs	Global	Female	65-69 years	Neoplasms	Alcohol use	Number	1990	233969	201693	267559
DALYs	Global	Male	65-69 years	Neoplasms	Alcohol use	Number	1990	760860	670570	863708
DALYs	Global	Female	65-69 years	Neurological disorders	Alcohol use	Number	1990	8453	4835	13526
DALYs	Global	Male	65-69 years	Neurological disorders	Alcohol use	Number	1990	21943	14447	31635
DALYs	Global	Female	65-69 years	Respiratory infections and tuberculosis	Alcohol use	Number	1990	74999	47669	106747
DALYs	Global	Male	65-69 years	Respiratory infections and tuberculosis	Alcohol use	Number	1990	575978	402279	737689
DALYs	Global	Female	65-69 years	Self-harm and interpersonal violence	Alcohol use	Number	1990	25151	12418	38433
DALYs	Global	Male	65-69 years	Self-harm and interpersonal violence	Alcohol use	Number	1990	113184	70207	159130
DALYs	Global	Female	65-69 years	Substance use disorders	Alcohol use	Number	1990	75888	62418	94224
DALYs	Global	Male	65-69 years	Substance use disorders	Alcohol use	Number	1990	258649	210345	316210
DALYs	Global	Female	65-69 years	Transport injuries	Alcohol use	Number	1990	7713	4005	12397
DALYs	Global	Male	65-69 years	Transport injuries	Alcohol use	Number	1990	54711	30842	82492
DALYs	Global	Female	65-69 years	Unintentional injuries	Alcohol use	Number	1990	31842	13934	57729
DALYs	Global	Male	65-69 years	Unintentional injuries	Alcohol use	Number	1990	95119	43332	164561
DALYs	Global	Female	70-74 years	Digestive diseases	Alcohol use	Number	1990	213130	162613	270834
DALYs	Global	Male	70-74 years	Digestive diseases	Alcohol use	Number	1990	528795	414260	644603
DALYs	Global	Female	70-74 years	Neoplasms	Alcohol use	Number	1990	166960	142737	191934
DALYs	Global	Male	70-74 years	Neoplasms	Alcohol use	Number	1990	472877	409309	541761
DALYs	Global	Female	70-74 years	Neurological disorders	Alcohol use	Number	1990	6110	3493	9682
DALYs	Global	Male	70-74 years	Neurological disorders	Alcohol use	Number	1990	13980	9104	20318
DALYs	Global	Female	70-74 years	Respiratory infections and tuberculosis	Alcohol use	Number	1990	68386	43887	97978
DALYs	Global	Male	70-74 years	Respiratory infections and tuberculosis	Alcohol use	Number	1990	419443	292092	550384
DALYs	Global	Female	70-74 years	Self-harm and interpersonal violence	Alcohol use	Number	1990	16104	7877	25135
DALYs	Global	Male	70-74 years	Self-harm and interpersonal violence	Alcohol use	Number	1990	65441	39675	93993
DALYs	Global	Female	70-74 years	Substance use disorders	Alcohol use	Number	1990	39218	30462	51641
DALYs	Global	Male	70-74 years	Substance use disorders	Alcohol use	Number	1990	127087	100531	162773
DALYs	Global	Female	70-74 years	Transport injuries	Alcohol use	Number	1990	13466	6965	21826
DALYs	Global	Male	70-74 years	Transport injuries	Alcohol use	Number	1990	37374	21067	56386
DALYs	Global	Female	70-74 years	Unintentional injuries	Alcohol use	Number	1990	26016	11447	48260
DALYs	Global	Male	70-74 years	Unintentional injuries	Alcohol use	Number	1990	60289	26985	104324
DALYs	Global	Female	75-79 years	Digestive diseases	Alcohol use	Number	1990	150199	114613	190068
DALYs	Global	Male	75-79 years	Digestive diseases	Alcohol use	Number	1990	308165	247704	369910
DALYs	Global	Female	75-79 years	Neoplasms	Alcohol use	Number	1990	128480	109976	146210
DALYs	Global	Male	75-79 years	Neoplasms	Alcohol use	Number	1990	287158	252374	324082
DALYs	Global	Female	75-79 years	Neurological disorders	Alcohol use	Number	1990	5041	2828	7976
DALYs	Global	Male	75-79 years	Neurological disorders	Alcohol use	Number	1990	10010	6520	14419
DALYs	Global	Female	75-79 years	Respiratory infections and tuberculosis	Alcohol use	Number	1990	48791	28668	71079
DALYs	Global	Male	75-79 years	Respiratory infections and tuberculosis	Alcohol use	Number	1990	249336	177502	322382

DALYs	Global	Female	75-79 years	Self-harm and interpersonal violence	Alcohol use	Number	1990	11398	5439	18077
DALYs	Global	Male	75-79 years	Self-harm and interpersonal violence	Alcohol use	Number	1990	42614	25108	61656
DALYs	Global	Female	75-79 years	Substance use disorders	Alcohol use	Number	1990	29710	23337	38473
DALYs	Global	Male	75-79 years	Substance use disorders	Alcohol use	Number	1990	73888	59176	92756
DALYs	Global	Female	75-79 years	Transport injuries	Alcohol use	Number	1990	10235	5283	16756
DALYs	Global	Male	75-79 years	Transport injuries	Alcohol use	Number	1990	36905	21059	55218
DALYs	Global	Female	75-79 years	Unintentional injuries	Alcohol use	Number	1990	28407	12370	51985
DALYs	Global	Male	75-79 years	Unintentional injuries	Alcohol use	Number	1990	47314	21805	81527
DALYs	Global	Female	80-84	Digestive diseases	Alcohol use	Number	1990	81606	60970	104454
DALYs	Global	Male	80-84	Digestive diseases	Alcohol use	Number	1990	128190	101176	156539
DALYs	Global	Female	80-84	Neoplasms	Alcohol use	Number	1990	71743	60306	83310
DALYs	Global	Male	80-84	Neoplasms	Alcohol use	Number	1990	118286	102192	134239
DALYs	Global	Female	80-84	Neurological disorders	Alcohol use	Number	1990	3185	1787	5070
DALYs	Global	Male	80-84	Neurological disorders	Alcohol use	Number	1990	4955	3113	7349
DALYs	Global	Female	80-84	Respiratory infections and tuberculosis	Alcohol use	Number	1990	34505	19179	50350
DALYs	Global	Male	80-84	Respiratory infections and tuberculosis	Alcohol use	Number	1990	121433	82871	159541
DALYs	Global	Female	80-84	Self-harm and interpersonal violence	Alcohol use	Number	1990	5600	2510	8935
DALYs	Global	Male	80-84	Self-harm and interpersonal violence	Alcohol use	Number	1990	18999	10441	28205
DALYs	Global	Female	80-84	Substance use disorders	Alcohol use	Number	1990	16014	11812	21557
DALYs	Global	Male	80-84	Substance use disorders	Alcohol use	Number	1990	28385	22166	36594
DALYs	Global	Female	80-84	Transport injuries	Alcohol use	Number	1990	11615	6053	18872
DALYs	Global	Male	80-84	Transport injuries	Alcohol use	Number	1990	25053	14629	35727
DALYs	Global	Female	80-84	Unintentional injuries	Alcohol use	Number	1990	24702	11131	45362
DALYs	Global	Male	80-84	Unintentional injuries	Alcohol use	Number	1990	27706	12952	48487
DALYs	Global	Female	85-89	Digestive diseases	Alcohol use	Number	1990	34791	25722	44779
DALYs	Global	Male	85-89	Digestive diseases	Alcohol use	Number	1990	43076	34218	52556
DALYs	Global	Female	85-89	Neoplasms	Alcohol use	Number	1990	34572	28191	40575
DALYs	Global	Male	85-89	Neoplasms	Alcohol use	Number	1990	40190	34253	45740
DALYs	Global	Female	85-89	Neurological disorders	Alcohol use	Number	1990	1664	947	2675
DALYs	Global	Male	85-89	Neurological disorders	Alcohol use	Number	1990	2040	1272	3013
DALYs	Global	Female	85-89	Respiratory infections and tuberculosis	Alcohol use	Number	1990	21879	10455	33655
DALYs	Global	Male	85-89	Respiratory infections and tuberculosis	Alcohol use	Number	1990	53744	34451	71216
DALYs	Global	Female	85-89	Self-harm and interpersonal violence	Alcohol use	Number	1990	2318	1069	3662
DALYs	Global	Male	85-89	Self-harm and interpersonal violence	Alcohol use	Number	1990	6979	3804	10218
DALYs	Global	Female	85-89	Substance use disorders	Alcohol use	Number	1990	8161	6051	10926
DALYs	Global	Male	85-89	Substance use disorders	Alcohol use	Number	1990	9501	7380	12140
DALYs	Global	Female	85-89	Transport injuries	Alcohol use	Number	1990	4883	2591	7964
DALYs	Global	Male	85-89	Transport injuries	Alcohol use	Number	1990	8597	5029	12390
DALYs	Global	Male	85-89	Unintentional injuries	Alcohol use	Number	1990	13950	6598	24061
DALYs	Global	Female	85-89	Unintentional injuries	Alcohol use	Number	1990	17851	8048	32230
DALYs	Global	Male	90-94	Digestive diseases	Alcohol use	Number	1990	9395	7236	11595
DALYs	Global	Female	90-94	Digestive diseases	Alcohol use	Number	1990	10477	7248	13939
DALYs	Global	Male	90-94	Neoplasms	Alcohol use	Number	1990	7878	6539	9128
DALYs	Global	Female	90-94	Neoplasms	Alcohol use	Number	1990	10570	8268	12832
DALYs	Global	Male	90-94	Neurological disorders	Alcohol use	Number	1990	538	326	815
DALYs	Global	Female	90-94	Neurological disorders	Alcohol use	Number	1990	585	344	940
DALYs	Global	Female	90-94	Respiratory infections and tuberculosis	Alcohol use	Number	1990	8497	3554	13642
DALYs	Global	Male	90-94	Respiratory infections and tuberculosis	Alcohol use	Number	1990	14267	8707	19573
DALYs	Global	Female	90-94	Self-harm and interpersonal violence	Alcohol use	Number	1990	528	259	838
DALYs	Global	Male	90-94	Self-harm and interpersonal violence	Alcohol use	Number	1990	1295	719	1872
DALYs	Global	Male	90-94	Substance use disorders	Alcohol use	Number	1990	2209	1636	2972

DALYs	Global	Female	90-94	Substance use disorders	Alcohol use	Number	1990	2855	1973	4045
DALYs	Global	Female	90-94	Transport injuries	Alcohol use	Number	1990	1377	728	2243
DALYs	Global	Male	90-94	Transport injuries	Alcohol use	Number	1990	1843	1092	2688
DALYs	Global	Male	90-94	Unintentional injuries	Alcohol use	Number	1990	4304	2028	7497
DALYs	Global	Female	90-94	Unintentional injuries	Alcohol use	Number	1990	7860	3581	14190
DALYs	Global	Male	95+ years	Digestive diseases	Alcohol use	Number	1990	1858	1322	2387
DALYs	Global	Female	95+ years	Digestive diseases	Alcohol use	Number	1990	2678	1686	3740
DALYs	Global	Male	95+ years	Neoplasms	Alcohol use	Number	1990	1237	987	1461
DALYs	Global	Female	95+ years	Neoplasms	Alcohol use	Number	1990	2429	1821	2959
DALYs	Global	Male	95+ years	Neurological disorders	Alcohol use	Number	1990	114	68	177
DALYs	Global	Female	95+ years	Neurological disorders	Alcohol use	Number	1990	156	87	254
DALYs	Global	Female	95+ years	Respiratory infections and tuberculosis	Alcohol use	Number	1990	2633	1022	4375
DALYs	Global	Male	95+ years	Respiratory infections and tuberculosis	Alcohol use	Number	1990	2990	1644	4338
DALYs	Global	Female	95+ years	Self-harm and interpersonal violence	Alcohol use	Number	1990	87	45	136
DALYs	Global	Male	95+ years	Self-harm and interpersonal violence	Alcohol use	Number	1990	186	113	259
DALYs	Global	Male	95+ years	Substance use disorders	Alcohol use	Number	1990	562	397	805
DALYs	Global	Female	95+ years	Substance use disorders	Alcohol use	Number	1990	922	611	1399
DALYs	Global	Female	95+ years	Transport injuries	Alcohol use	Number	1990	356	189	577
DALYs	Global	Male	95+ years	Transport injuries	Alcohol use	Number	1990	377	223	555
DALYs	Global	Male	95+ years	Unintentional injuries	Alcohol use	Number	1990	959	454	1673
DALYs	Global	Female	95+ years	Unintentional injuries	Alcohol use	Number	1990	2344	1076	4255
DALYs	Global	Male	15-19 years	Digestive diseases	Alcohol use	Number	2019	89998	60394	125663
DALYs	Global	Female	15-19 years	Digestive diseases	Alcohol use	Number	2019	26413	17290	38688
DALYs	Global	Male	15-19 years	Unintentional injuries	Alcohol use	Number	2019	79579	35516	140912
DALYs	Global	Female	15-19 years	Unintentional injuries	Alcohol use	Number	2019	14954	6203	28212
DALYs	Global	Male	15-19 years	Self-harm and interpersonal violence	Alcohol use	Number	2019	310142	198190	429313
DALYs	Global	Female	15-19 years	Self-harm and interpersonal violence	Alcohol use	Number	2019	42754	24081	63853
DALYs	Global	Male	15-19 years	Respiratory infections and tuberculosis	Alcohol use	Number	2019	77192	46391	109701
DALYs	Global	Female	15-19 years	Respiratory infections and tuberculosis	Alcohol use	Number	2019	26509	14577	39314
DALYs	Global	Male	15-19 years	Substance use disorders	Alcohol use	Number	2019	286559	177509	433072
DALYs	Global	Female	15-19 years	Substance use disorders	Alcohol use	Number	2019	113606	66859	180498
DALYs	Global	Male	15-19 years	Transport injuries	Alcohol use	Number	2019	263858	146261	410893
DALYs	Global	Female	15-19 years	Transport injuries	Alcohol use	Number	2019	32061	16276	51379
DALYs	Global	Male	15-19 years	Neoplasms	Alcohol use	Number	2019	7970	6440	9647
DALYs	Global	Female	15-19 years	Neoplasms	Alcohol use	Number	2019	2513	2070	2932
DALYs	Global	Male	15-19 years	Neurological disorders	Alcohol use	Number	2019	57480	35019	89436
DALYs	Global	Female	15-19 years	Neurological disorders	Alcohol use	Number	2019	21069	11942	34895
DALYs	Global	Male	20-24 years	Digestive diseases	Alcohol use	Number	2019	323638	232793	429458
DALYs	Global	Female	20-24 years	Digestive diseases	Alcohol use	Number	2019	58211	39868	81898
DALYs	Global	Male	20-24 years	Unintentional injuries	Alcohol use	Number	2019	193544	92278	331505
DALYs	Global	Female	20-24 years	Unintentional injuries	Alcohol use	Number	2019	25663	11090	46921
DALYs	Global	Male	20-24 years	Self-harm and interpersonal violence	Alcohol use	Number	2019	903379	607728	1214038
DALYs	Global	Female	20-24 years	Self-harm and interpersonal violence	Alcohol use	Number	2019	78284	47024	111334
DALYs	Global	Male	20-24 years	Respiratory infections and tuberculosis	Alcohol use	Number	2019	369263	241900	492099
DALYs	Global	Female	20-24 years	Respiratory infections and tuberculosis	Alcohol use	Number	2019	78976	47530	112978
DALYs	Global	Male	20-24 years	Substance use disorders	Alcohol use	Number	2019	849818	552090	1290762
DALYs	Global	Female	20-24 years	Substance use disorders	Alcohol use	Number	2019	272964	160047	446400
DALYs	Global	Male	20-24 years	Transport injuries	Alcohol use	Number	2019	823673	465396	1194167
DALYs	Global	Female	20-24 years	Transport injuries	Alcohol use	Number	2019	46548	23996	74753
DALYs	Global	Male	20-24 years	Neoplasms	Alcohol use	Number	2019	37259	31028	44619
DALYs	Global	Female	20-24 years	Neoplasms	Alcohol use	Number	2019	9037	7662	10579

DALYs	Global	Male	20-24 years	Neurological disorders	Alcohol use	Number	2019	119275	78327	173827
DALYs	Global	Female	20-24 years	Neurological disorders	Alcohol use	Number	2019	30634	17857	48893
DALYs	Global	Female	25-29 years	Neoplasms	Alcohol use	Number	2019	20865	17739	24079
DALYs	Global	Male	25-29 years	Digestive diseases	Alcohol use	Number	2019	707168	534330	889845
DALYs	Global	Female	25-29 years	Digestive diseases	Alcohol use	Number	2019	100419	72722	134826
DALYs	Global	Male	25-29 years	Unintentional injuries	Alcohol use	Number	2019	242376	113702	418621
DALYs	Global	Female	25-29 years	Unintentional injuries	Alcohol use	Number	2019	31412	13550	58403
DALYs	Global	Male	25-29 years	Self-harm and interpersonal violence	Alcohol use	Number	2019	1055133	723623	1388965
DALYs	Global	Female	25-29 years	Self-harm and interpersonal violence	Alcohol use	Number	2019	83577	49928	120125
DALYs	Global	Male	25-29 years	Respiratory infections and tuberculosis	Alcohol use	Number	2019	614742	417351	802025
DALYs	Global	Female	25-29 years	Respiratory infections and tuberculosis	Alcohol use	Number	2019	95791	59428	135724
DALYs	Global	Male	25-29 years	Substance use disorders	Alcohol use	Number	2019	1320566	902314	1862714
DALYs	Global	Female	25-29 years	Substance use disorders	Alcohol use	Number	2019	351777	222095	521945
DALYs	Global	Male	25-29 years	Transport injuries	Alcohol use	Number	2019	835536	480041	1211482
DALYs	Global	Female	25-29 years	Transport injuries	Alcohol use	Number	2019	35201	18117	56022
DALYs	Global	Male	25-29 years	Neoplasms	Alcohol use	Number	2019	76127	64213	89840
DALYs	Global	Male	25-29 years	Neurological disorders	Alcohol use	Number	2019	134299	91608	192158
DALYs	Global	Female	25-29 years	Neurological disorders	Alcohol use	Number	2019	30526	18022	47836
DALYs	Global	Male	30-34 years	Neoplasms	Alcohol use	Number	2019	173417	145942	203251
DALYs	Global	Female	30-34 years	Neoplasms	Alcohol use	Number	2019	50104	42543	57697
DALYs	Global	Male	30-34 years	Digestive diseases	Alcohol use	Number	2019	1215486	933353	1495319
DALYs	Global	Female	30-34 years	Digestive diseases	Alcohol use	Number	2019	176459	133093	224662
DALYs	Global	Male	30-34 years	Unintentional injuries	Alcohol use	Number	2019	277202	129724	472566
DALYs	Global	Female	30-34 years	Unintentional injuries	Alcohol use	Number	2019	37187	16186	68648
DALYs	Global	Male	30-34 years	Self-harm and interpersonal violence	Alcohol use	Number	2019	1011777	687339	1334927
DALYs	Global	Female	30-34 years	Self-harm and interpersonal violence	Alcohol use	Number	2019	82410	49527	119264
DALYs	Global	Male	30-34 years	Respiratory infections and tuberculosis	Alcohol use	Number	2019	782825	538020	1015947
DALYs	Global	Female	30-34 years	Respiratory infections and tuberculosis	Alcohol use	Number	2019	99059	61236	139715
DALYs	Global	Male	30-34 years	Substance use disorders	Alcohol use	Number	2019	1715692	1250135	2391497
DALYs	Global	Female	30-34 years	Substance use disorders	Alcohol use	Number	2019	391514	254553	592374
DALYs	Global	Male	30-34 years	Neurological disorders	Alcohol use	Number	2019	128148	86138	182220
DALYs	Global	Female	30-34 years	Neurological disorders	Alcohol use	Number	2019	27399	16350	42525
DALYs	Global	Male	30-34 years	Transport injuries	Alcohol use	Number	2019	560948	321382	863223
DALYs	Global	Female	30-34 years	Transport injuries	Alcohol use	Number	2019	21980	11280	35025
DALYs	Global	Male	35-39 years	Neoplasms	Alcohol use	Number	2019	314088	267188	366794
DALYs	Global	Female	35-39 years	Neoplasms	Alcohol use	Number	2019	92183	78818	107015
DALYs	Global	Male	35-39 years	Digestive diseases	Alcohol use	Number	2019	1789004	1376664	2202411
DALYs	Global	Female	35-39 years	Digestive diseases	Alcohol use	Number	2019	243686	179102	311415
DALYs	Global	Male	35-39 years	Unintentional injuries	Alcohol use	Number	2019	286986	134116	494562
DALYs	Global	Female	35-39 years	Unintentional injuries	Alcohol use	Number	2019	41015	18223	76989
DALYs	Global	Male	35-39 years	Self-harm and interpersonal violence	Alcohol use	Number	2019	871001	599459	1155751
DALYs	Global	Female	35-39 years	Self-harm and interpersonal violence	Alcohol use	Number	2019	76891	44539	110795
DALYs	Global	Male	35-39 years	Respiratory infections and tuberculosis	Alcohol use	Number	2019	876273	610595	1124514
DALYs	Global	Female	35-39 years	Respiratory infections and tuberculosis	Alcohol use	Number	2019	95447	58997	133123
DALYs	Global	Male	35-39 years	Substance use disorders	Alcohol use	Number	2019	1734632	1341957	2290985
DALYs	Global	Female	35-39 years	Substance use disorders	Alcohol use	Number	2019	382672	274439	532306
DALYs	Global	Male	35-39 years	Neurological disorders	Alcohol use	Number	2019	118441	81041	164575
DALYs	Global	Female	35-39 years	Neurological disorders	Alcohol use	Number	2019	24521	14526	37692
DALYs	Global	Male	35-39 years	Transport injuries	Alcohol use	Number	2019	380065	216296	591002
DALYs	Global	Female	35-39 years	Transport injuries	Alcohol use	Number	2019	18620	9680	29932
DALYs	Global	Male	40-44 years	Neoplasms	Alcohol use	Number	2019	566546	485210	650641

DALYs	Global	Female	40-44 years	Neoplasms	Alcohol use	Number	2019	152510	129168	176899
DALYs	Global	Male	40-44 years	Digestive diseases	Alcohol use	Number	2019	2228086	1723970	2728083
DALYs	Global	Female	40-44 years	Digestive diseases	Alcohol use	Number	2019	336414	248406	425671
DALYs	Global	Male	40-44 years	Unintentional injuries	Alcohol use	Number	2019	289610	135349	492933
DALYs	Global	Female	40-44 years	Unintentional injuries	Alcohol use	Number	2019	45378	20005	83915
DALYs	Global	Male	40-44 years	Self-harm and interpersonal violence	Alcohol use	Number	2019	701515	478794	938717
DALYs	Global	Female	40-44 years	Self-harm and interpersonal violence	Alcohol use	Number	2019	74572	44987	106728
DALYs	Global	Male	40-44 years	Respiratory infections and tuberculosis	Alcohol use	Number	2019	888247	618986	1126015
DALYs	Global	Female	40-44 years	Respiratory infections and tuberculosis	Alcohol use	Number	2019	96764	59275	141238
DALYs	Global	Male	40-44 years	Substance use disorders	Alcohol use	Number	2019	1680327	1316876	2177323
DALYs	Global	Female	40-44 years	Substance use disorders	Alcohol use	Number	2019	352603	254950	484952
DALYs	Global	Male	40-44 years	Neurological disorders	Alcohol use	Number	2019	104384	70456	144084
DALYs	Global	Female	40-44 years	Neurological disorders	Alcohol use	Number	2019	24230	14173	36299
DALYs	Global	Male	40-44 years	Transport injuries	Alcohol use	Number	2019	296348	168752	464042
DALYs	Global	Female	40-44 years	Transport injuries	Alcohol use	Number	2019	24566	12744	39174
DALYs	Global	Male	45-49 years	Neoplasms	Alcohol use	Number	2019	935389	799586	1079811
DALYs	Global	Female	45-49 years	Neoplasms	Alcohol use	Number	2019	222249	189473	255464
DALYs	Global	Male	45-49 years	Digestive diseases	Alcohol use	Number	2019	2559326	1994459	3133834
DALYs	Global	Female	45-49 years	Digestive diseases	Alcohol use	Number	2019	407241	304476	522046
DALYs	Global	Male	45-49 years	Unintentional injuries	Alcohol use	Number	2019	295895	135632	505463
DALYs	Global	Female	45-49 years	Unintentional injuries	Alcohol use	Number	2019	49000	21685	90103
DALYs	Global	Male	45-49 years	Self-harm and interpersonal violence	Alcohol use	Number	2019	591680	405979	794319
DALYs	Global	Female	45-49 years	Self-harm and interpersonal violence	Alcohol use	Number	2019	70815	41210	102733
DALYs	Global	Male	45-49 years	Respiratory infections and tuberculosis	Alcohol use	Number	2019	926109	648974	1162910
DALYs	Global	Female	45-49 years	Respiratory infections and tuberculosis	Alcohol use	Number	2019	94756	58270	134611
DALYs	Global	Male	45-49 years	Substance use disorders	Alcohol use	Number	2019	1621797	1311632	2025292
DALYs	Global	Female	45-49 years	Substance use disorders	Alcohol use	Number	2019	329657	257021	428274
DALYs	Global	Male	45-49 years	Neurological disorders	Alcohol use	Number	2019	92717	62242	127617
DALYs	Global	Female	45-49 years	Neurological disorders	Alcohol use	Number	2019	22037	12937	33815
DALYs	Global	Male	45-49 years	Transport injuries	Alcohol use	Number	2019	265661	151267	418864
DALYs	Global	Female	45-49 years	Transport injuries	Alcohol use	Number	2019	16742	8830	26866
DALYs	Global	Male	50-54 years	Neoplasms	Alcohol use	Number	2019	1379204	1173564	1574736
DALYs	Global	Female	50-54 years	Neoplasms	Alcohol use	Number	2019	301141	257575	347310
DALYs	Global	Male	50-54 years	Digestive diseases	Alcohol use	Number	2019	2677022	2044322	3282569
DALYs	Global	Female	50-54 years	Digestive diseases	Alcohol use	Number	2019	495206	360780	646497
DALYs	Global	Male	50-54 years	Unintentional injuries	Alcohol use	Number	2019	284890	128836	494992
DALYs	Global	Female	50-54 years	Unintentional injuries	Alcohol use	Number	2019	52381	23439	97475
DALYs	Global	Male	50-54 years	Self-harm and interpersonal violence	Alcohol use	Number	2019	476568	320193	642949
DALYs	Global	Female	50-54 years	Self-harm and interpersonal violence	Alcohol use	Number	2019	62585	35289	91447
DALYs	Global	Male	50-54 years	Respiratory infections and tuberculosis	Alcohol use	Number	2019	889639	628735	1137470
DALYs	Global	Female	50-54 years	Respiratory infections and tuberculosis	Alcohol use	Number	2019	110939	68434	159721
DALYs	Global	Male	50-54 years	Substance use disorders	Alcohol use	Number	2019	1354369	1079336	1704019
DALYs	Global	Female	50-54 years	Substance use disorders	Alcohol use	Number	2019	290677	222654	387699
DALYs	Global	Male	50-54 years	Neurological disorders	Alcohol use	Number	2019	77397	51428	109128
DALYs	Global	Female	50-54 years	Neurological disorders	Alcohol use	Number	2019	20690	12142	31584
DALYs	Global	Male	50-54 years	Transport injuries	Alcohol use	Number	2019	203225	115184	320209
DALYs	Global	Female	50-54 years	Transport injuries	Alcohol use	Number	2019	19134	10151	30781
DALYs	Global	Male	55-59 years	Neoplasms	Alcohol use	Number	2019	1627006	1411523	1859679
DALYs	Global	Female	55-59 years	Neoplasms	Alcohol use	Number	2019	343030	296284	391668
DALYs	Global	Male	55-59 years	Digestive diseases	Alcohol use	Number	2019	2540184	1954045	3110197
DALYs	Global	Female	55-59 years	Digestive diseases	Alcohol use	Number	2019	586331	427097	763745

DALYs	Global	Male	55-59 years	Unintentional injuries	Alcohol use	Number	2019	255991	117920	441877
DALYs	Global	Female	55-59 years	Unintentional injuries	Alcohol use	Number	2019	54187	24059	102392
DALYs	Global	Male	55-59 years	Self-harm and interpersonal violence	Alcohol use	Number	2019	375197	245708	507558
DALYs	Global	Female	55-59 years	Self-harm and interpersonal violence	Alcohol use	Number	2019	50211	28882	73202
DALYs	Global	Male	55-59 years	Respiratory infections and tuberculosis	Alcohol use	Number	2019	843448	586075	1067064
DALYs	Global	Female	55-59 years	Respiratory infections and tuberculosis	Alcohol use	Number	2019	107484	67175	150332
DALYs	Global	Male	55-59 years	Substance use disorders	Alcohol use	Number	2019	1153721	951907	1412077
DALYs	Global	Female	55-59 years	Substance use disorders	Alcohol use	Number	2019	250388	199136	317698
DALYs	Global	Male	55-59 years	Neurological disorders	Alcohol use	Number	2019	68209	45822	98135
DALYs	Global	Female	55-59 years	Neurological disorders	Alcohol use	Number	2019	19062	11208	30011
DALYs	Global	Male	55-59 years	Transport injuries	Alcohol use	Number	2019	171824	96590	268818
DALYs	Global	Female	55-59 years	Transport injuries	Alcohol use	Number	2019	22161	11700	36066
DALYs	Global	Male	60-64 years	Neoplasms	Alcohol use	Number	2019	1659846	1439971	1894608
DALYs	Global	Female	60-64 years	Neoplasms	Alcohol use	Number	2019	344541	297805	393610
DALYs	Global	Male	60-64 years	Digestive diseases	Alcohol use	Number	2019	2149200	1661236	2627099
DALYs	Global	Female	60-64 years	Digestive diseases	Alcohol use	Number	2019	565924	418307	737075
DALYs	Global	Male	60-64 years	Unintentional injuries	Alcohol use	Number	2019	223106	102319	388446
DALYs	Global	Female	60-64 years	Unintentional injuries	Alcohol use	Number	2019	53767	23577	96851
DALYs	Global	Male	60-64 years	Self-harm and interpersonal violence	Alcohol use	Number	2019	257998	167778	346569
DALYs	Global	Female	60-64 years	Self-harm and interpersonal violence	Alcohol use	Number	2019	36516	20175	54083
DALYs	Global	Male	60-64 years	Respiratory infections and tuberculosis	Alcohol use	Number	2019	712670	512468	909298
DALYs	Global	Female	60-64 years	Respiratory infections and tuberculosis	Alcohol use	Number	2019	95976	59941	135238
DALYs	Global	Male	60-64 years	Substance use disorders	Alcohol use	Number	2019	857070	692947	1068561
DALYs	Global	Female	60-64 years	Substance use disorders	Alcohol use	Number	2019	188873	146877	248552
DALYs	Global	Male	60-64 years	Neurological disorders	Alcohol use	Number	2019	59472	39068	87793
DALYs	Global	Female	60-64 years	Neurological disorders	Alcohol use	Number	2019	17573	10311	27944
DALYs	Global	Male	60-64 years	Transport injuries	Alcohol use	Number	2019	121405	68031	189731
DALYs	Global	Female	60-64 years	Transport injuries	Alcohol use	Number	2019	11363	5956	18605
DALYs	Global	Male	65-69 years	Neoplasms	Alcohol use	Number	2019	1471429	1276289	1685204
DALYs	Global	Female	65-69 years	Neoplasms	Alcohol use	Number	2019	314908	270208	366005
DALYs	Global	Male	65-69 years	Digestive diseases	Alcohol use	Number	2019	1553292	1204548	1895748
DALYs	Global	Female	65-69 years	Digestive diseases	Alcohol use	Number	2019	453378	330215	593301
DALYs	Global	Male	65-69 years	Unintentional injuries	Alcohol use	Number	2019	180624	82837	311818
DALYs	Global	Female	65-69 years	Unintentional injuries	Alcohol use	Number	2019	50003	22079	90065
DALYs	Global	Male	65-69 years	Self-harm and interpersonal violence	Alcohol use	Number	2019	173795	110306	241677
DALYs	Global	Female	65-69 years	Self-harm and interpersonal violence	Alcohol use	Number	2019	25708	14032	38896
DALYs	Global	Male	65-69 years	Respiratory infections and tuberculosis	Alcohol use	Number	2019	580026	416176	746337
DALYs	Global	Female	65-69 years	Respiratory infections and tuberculosis	Alcohol use	Number	2019	74830	45888	107329
DALYs	Global	Male	65-69 years	Substance use disorders	Alcohol use	Number	2019	554231	450328	687587
DALYs	Global	Female	65-69 years	Substance use disorders	Alcohol use	Number	2019	132825	104635	170721
DALYs	Global	Male	65-69 years	Neurological disorders	Alcohol use	Number	2019	49903	31903	74082
DALYs	Global	Female	65-69 years	Neurological disorders	Alcohol use	Number	2019	15473	8985	25458
DALYs	Global	Male	65-69 years	Transport injuries	Alcohol use	Number	2019	83230	46849	129690
DALYs	Global	Female	65-69 years	Transport injuries	Alcohol use	Number	2019	8149	4281	13230
DALYs	Global	Male	70-74 years	Neoplasms	Alcohol use	Number	2019	1073852	922827	1233983
DALYs	Global	Female	70-74 years	Neoplasms	Alcohol use	Number	2019	257344	218546	300230
DALYs	Global	Male	70-74 years	Digestive diseases	Alcohol use	Number	2019	948054	725468	1172576
DALYs	Global	Female	70-74 years	Digestive diseases	Alcohol use	Number	2019	324658	234475	423050
DALYs	Global	Male	70-74 years	Unintentional injuries	Alcohol use	Number	2019	139745	64929	244569
DALYs	Global	Female	70-74 years	Unintentional injuries	Alcohol use	Number	2019	46887	20982	87989
DALYs	Global	Male	70-74 years	Self-harm and interpersonal violence	Alcohol use	Number	2019	111421	68285	157371

DALYs	Global	Female	70-74 years	Self-harm and interpersonal violence	Alcohol use	Number	2019	18101	9390	26997
DALYs	Global	Male	70-74 years	Respiratory infections and tuberculosis	Alcohol use	Number	2019	468134	338469	598096
DALYs	Global	Female	70-74 years	Respiratory infections and tuberculosis	Alcohol use	Number	2019	68754	42471	98603
DALYs	Global	Male	70-74 years	Substance use disorders	Alcohol use	Number	2019	306455	242777	392758
DALYs	Global	Female	70-74 years	Substance use disorders	Alcohol use	Number	2019	76656	57684	104186
DALYs	Global	Male	70-74 years	Neurological disorders	Alcohol use	Number	2019	36765	23637	54980
DALYs	Global	Female	70-74 years	Neurological disorders	Alcohol use	Number	2019	12948	7487	20910
DALYs	Global	Male	70-74 years	Transport injuries	Alcohol use	Number	2019	55912	31499	87602
DALYs	Global	Female	70-74 years	Transport injuries	Alcohol use	Number	2019	16551	8525	27011
DALYs	Global	Male	75-79 years	Neoplasms	Alcohol use	Number	2019	628245	542523	722541
DALYs	Global	Female	75-79 years	Neoplasms	Alcohol use	Number	2019	173572	146022	203144
DALYs	Global	Male	75-79 years	Digestive diseases	Alcohol use	Number	2019	524718	408786	653048
DALYs	Global	Female	75-79 years	Digestive diseases	Alcohol use	Number	2019	207937	152960	271879
DALYs	Global	Male	75-79 years	Unintentional injuries	Alcohol use	Number	2019	100324	45164	173966
DALYs	Global	Female	75-79 years	Unintentional injuries	Alcohol use	Number	2019	41115	17662	78426
DALYs	Global	Male	75-79 years	Self-harm and interpersonal violence	Alcohol use	Number	2019	63450	37264	90932
DALYs	Global	Female	75-79 years	Self-harm and interpersonal violence	Alcohol use	Number	2019	10541	5447	16136
DALYs	Global	Male	75-79 years	Respiratory infections and tuberculosis	Alcohol use	Number	2019	293533	205936	385014
DALYs	Global	Female	75-79 years	Respiratory infections and tuberculosis	Alcohol use	Number	2019	47097	27136	71384
DALYs	Global	Male	75-79 years	Substance use disorders	Alcohol use	Number	2019	163793	130263	206556
DALYs	Global	Female	75-79 years	Substance use disorders	Alcohol use	Number	2019	52346	39693	69537
DALYs	Global	Male	75-79 years	Neurological disorders	Alcohol use	Number	2019	25187	16136	38027
DALYs	Global	Female	75-79 years	Neurological disorders	Alcohol use	Number	2019	9452	5411	15502
DALYs	Global	Male	75-79 years	Transport injuries	Alcohol use	Number	2019	57835	32763	90499
DALYs	Global	Female	75-79 years	Transport injuries	Alcohol use	Number	2019	12019	6240	19617
DALYs	Global	Male	80-84	Neoplasms	Alcohol use	Number	2019	326577	275321	375882
DALYs	Global	Female	80-84	Neoplasms	Alcohol use	Number	2019	118421	95556	139027
DALYs	Global	Male	80-84	Digestive diseases	Alcohol use	Number	2019	268770	203169	345179
DALYs	Global	Female	80-84	Digestive diseases	Alcohol use	Number	2019	135164	95982	181800
DALYs	Global	Male	80-84	Unintentional injuries	Alcohol use	Number	2019	72630	33696	130644
DALYs	Global	Female	80-84	Unintentional injuries	Alcohol use	Number	2019	40233	17368	75675
DALYs	Global	Male	80-84	Respiratory infections and tuberculosis	Alcohol use	Number	2019	186177	123913	247771
DALYs	Global	Female	80-84	Respiratory infections and tuberculosis	Alcohol use	Number	2019	38920	20330	59426
DALYs	Global	Male	80-84	Self-harm and interpersonal violence	Alcohol use	Number	2019	33002	17857	48553
DALYs	Global	Female	80-84	Self-harm and interpersonal violence	Alcohol use	Number	2019	6159	3000	9744
DALYs	Global	Male	80-84	Substance use disorders	Alcohol use	Number	2019	76844	59527	101111
DALYs	Global	Female	80-84	Substance use disorders	Alcohol use	Number	2019	33814	24373	46680
DALYs	Global	Male	80-84	Neurological disorders	Alcohol use	Number	2019	15707	9899	23663
DALYs	Global	Female	80-84	Neurological disorders	Alcohol use	Number	2019	7441	4275	12031
DALYs	Global	Male	80-84	Transport injuries	Alcohol use	Number	2019	41906	23914	65147
DALYs	Global	Female	80-84	Transport injuries	Alcohol use	Number	2019	14049	7254	23119
DALYs	Global	Male	85-89	Neoplasms	Alcohol use	Number	2019	147428	122411	171401
DALYs	Global	Female	85-89	Neoplasms	Alcohol use	Number	2019	72296	55742	86217
DALYs	Global	Male	85-89	Digestive diseases	Alcohol use	Number	2019	120303	92345	153263
DALYs	Global	Female	85-89	Digestive diseases	Alcohol use	Number	2019	70364	49752	93857
DALYs	Global	Male	85-89	Unintentional injuries	Alcohol use	Number	2019	48004	22252	85728
DALYs	Global	Female	85-89	Unintentional injuries	Alcohol use	Number	2019	36254	16111	67637
DALYs	Global	Male	85-89	Respiratory infections and tuberculosis	Alcohol use	Number	2019	110598	68708	153687
DALYs	Global	Female	85-89	Respiratory infections and tuberculosis	Alcohol use	Number	2019	30845	13652	50198
DALYs	Global	Male	85-89	Self-harm and interpersonal violence	Alcohol use	Number	2019	15671	8316	23285
DALYs	Global	Female	85-89	Self-harm and interpersonal violence	Alcohol use	Number	2019	3328	1603	5371

DALYs	Global	Male	85-89	Substance use disorders	Alcohol use	Number	2019	31753	24057	41507
DALYs	Global	Female	85-89	Substance use disorders	Alcohol use	Number	2019	21415	15440	29648
DALYs	Global	Male	85-89	Neurological disorders	Alcohol use	Number	2019	8307	5145	12583
DALYs	Global	Female	85-89	Neurological disorders	Alcohol use	Number	2019	5009	2891	8119
DALYs	Global	Male	85-89	Transport injuries	Alcohol use	Number	2019	17736	9992	27473
DALYs	Global	Female	85-89	Transport injuries	Alcohol use	Number	2019	7205	3740	11924
DALYs	Global	Male	90-94	Neoplasms	Alcohol use	Number	2019	38300	30487	45079
DALYs	Global	Female	90-94	Neoplasms	Alcohol use	Number	2019	34372	25117	42019
DALYs	Global	Male	90-94	Digestive diseases	Alcohol use	Number	2019	34822	25676	44737
DALYs	Global	Female	90-94	Digestive diseases	Alcohol use	Number	2019	30772	20541	42506
DALYs	Global	Male	90-94	Unintentional injuries	Alcohol use	Number	2019	20759	9736	36183
DALYs	Global	Female	90-94	Unintentional injuries	Alcohol use	Number	2019	24296	10770	44519
DALYs	Global	Male	90-94	Respiratory infections and tuberculosis	Alcohol use	Number	2019	44582	24803	64410
DALYs	Global	Female	90-94	Respiratory infections and tuberculosis	Alcohol use	Number	2019	20037	7411	34299
DALYs	Global	Male	90-94	Self-harm and interpersonal violence	Alcohol use	Number	2019	4048	2253	5953
DALYs	Global	Female	90-94	Self-harm and interpersonal violence	Alcohol use	Number	2019	1190	570	1891
DALYs	Global	Male	90-94	Substance use disorders	Alcohol use	Number	2019	10077	7303	13899
DALYs	Global	Female	90-94	Substance use disorders	Alcohol use	Number	2019	11109	7563	15728
DALYs	Global	Male	90-94	Neurological disorders	Alcohol use	Number	2019	2992	1832	4555
DALYs	Global	Female	90-94	Neurological disorders	Alcohol use	Number	2019	2553	1486	4166
DALYs	Global	Male	90-94	Transport injuries	Alcohol use	Number	2019	4934	2739	7651
DALYs	Global	Female	90-94	Transport injuries	Alcohol use	Number	2019	2912	1519	4774
DALYs	Global	Male	95+ years	Neoplasms	Alcohol use	Number	2019	7591	5758	9160
DALYs	Global	Female	95+ years	Neoplasms	Alcohol use	Number	2019	12019	8606	14850
DALYs	Global	Male	95+ years	Unintentional injuries	Alcohol use	Number	2019	6140	2941	10545
DALYs	Global	Female	95+ years	Unintentional injuries	Alcohol use	Number	2019	10830	4806	20044
DALYs	Global	Male	95+ years	Digestive diseases	Alcohol use	Number	2019	8629	5918	11479
DALYs	Global	Female	95+ years	Digestive diseases	Alcohol use	Number	2019	11895	7138	17545
DALYs	Global	Male	95+ years	Self-harm and interpersonal violence	Alcohol use	Number	2019	790	477	1126
DALYs	Global	Female	95+ years	Self-harm and interpersonal violence	Alcohol use	Number	2019	308	158	479
DALYs	Global	Male	95+ years	Respiratory infections and tuberculosis	Alcohol use	Number	2019	13221	6575	19907
DALYs	Global	Female	95+ years	Respiratory infections and tuberculosis	Alcohol use	Number	2019	10168	3438	17915
DALYs	Global	Male	95+ years	Substance use disorders	Alcohol use	Number	2019	2994	2068	4319
DALYs	Global	Female	95+ years	Substance use disorders	Alcohol use	Number	2019	4611	3038	7078
DALYs	Global	Male	95+ years	Neurological disorders	Alcohol use	Number	2019	749	449	1153
DALYs	Global	Female	95+ years	Neurological disorders	Alcohol use	Number	2019	900	516	1463
DALYs	Global	Male	95+ years	Transport injuries	Alcohol use	Number	2019	1095	605	1709
DALYs	Global	Female	95+ years	Transport injuries	Alcohol use	Number	2019	910	474	1487

Figure 2B Age-specific DALYs rate from level 2 GBD causes attributable to alcohol use in 1990 and 2019

measure	location	sex	age	cause	risk factor	metric	year	value (per 100000)	95% UI (lower)	95% UI (upper)
DALYs	Global	Male	15-19 years	Digestive diseases	Alcohol use	Rate	1990	27.6	36.6	20.2
DALYs	Global	Female	15-19 years	Digestive diseases	Alcohol use	Rate	1990	9.7	13.3	7.0
DALYs	Global	Male	15-19 years	Neoplasms	Alcohol use	Rate	1990	3.6	4.6	2.8
DALYs	Global	Female	15-19 years	Neoplasms	Alcohol use	Rate	1990	1.0	1.2	0.9
DALYs	Global	Male	15-19 years	Neurological disorders	Alcohol use	Rate	1990	20.8	32.4	13.0
DALYs	Global	Female	15-19 years	Neurological disorders	Alcohol use	Rate	1990	8.4	13.8	4.9
DALYs	Global	Male	15-19 years	Respiratory infections and tuberculosis	Alcohol use	Rate	1990	37.5	52.8	23.0
DALYs	Global	Female	15-19 years	Respiratory infections and tuberculosis	Alcohol use	Rate	1990	14.0	20.8	7.9
DALYs	Global	Male	15-19 years	Self-harm and interpersonal violence	Alcohol use	Rate	1990	131.0	181.4	83.7
DALYs	Global	Female	15-19 years	Self-harm and interpersonal violence	Alcohol use	Rate	1990	19.1	28.4	10.6
DALYs	Global	Male	15-19 years	Substance use disorders	Alcohol use	Rate	1990	121.1	179.4	77.3
DALYs	Global	Female	15-19 years	Substance use disorders	Alcohol use	Rate	1990	49.4	73.5	31.2
DALYs	Global	Male	15-19 years	Transport injuries	Alcohol use	Rate	1990	120.3	184.7	64.8
DALYs	Global	Female	15-19 years	Transport injuries	Alcohol use	Rate	1990	17.4	27.4	9.0
DALYs	Global	Male	15-19 years	Unintentional injuries	Alcohol use	Rate	1990	48.7	86.5	22.0
DALYs	Global	Female	15-19 years	Unintentional injuries	Alcohol use	Rate	1990	8.3	15.4	3.5
DALYs	Global	Male	20-24 years	Digestive diseases	Alcohol use	Rate	1990	95.6	125.1	70.9
DALYs	Global	Female	20-24 years	Digestive diseases	Alcohol use	Rate	1990	20.3	27.2	15.2
DALYs	Global	Male	20-24 years	Neoplasms	Alcohol use	Rate	1990	14.6	17.9	11.5
DALYs	Global	Female	20-24 years	Neoplasms	Alcohol use	Rate	1990	3.5	4.1	3.0
DALYs	Global	Male	20-24 years	Neurological disorders	Alcohol use	Rate	1990	41.2	59.2	27.5
DALYs	Global	Female	20-24 years	Neurological disorders	Alcohol use	Rate	1990	12.3	19.5	7.5
DALYs	Global	Male	20-24 years	Respiratory infections and tuberculosis	Alcohol use	Rate	1990	168.9	225.9	113.1
DALYs	Global	Female	20-24 years	Respiratory infections and tuberculosis	Alcohol use	Rate	1990	41.1	58.9	25.2
DALYs	Global	Male	20-24 years	Self-harm and interpersonal violence	Alcohol use	Rate	1990	363.4	496.4	241.9
DALYs	Global	Female	20-24 years	Self-harm and interpersonal violence	Alcohol use	Rate	1990	39.1	57.8	22.6
DALYs	Global	Male	20-24 years	Substance use disorders	Alcohol use	Rate	1990	368.8	552.1	239.0
DALYs	Global	Female	20-24 years	Substance use disorders	Alcohol use	Rate	1990	115.5	184.3	71.0
DALYs	Global	Male	20-24 years	Transport injuries	Alcohol use	Rate	1990	348.4	507.1	190.8
DALYs	Global	Female	20-24 years	Transport injuries	Alcohol use	Rate	1990	24.5	38.2	12.8
DALYs	Global	Male	20-24 years	Unintentional injuries	Alcohol use	Rate	1990	104.9	183.2	49.6
DALYs	Global	Female	20-24 years	Unintentional injuries	Alcohol use	Rate	1990	13.3	24.3	5.7
DALYs	Global	Male	25-29 years	Digestive diseases	Alcohol use	Rate	1990	213.9	266.4	164.6
DALYs	Global	Female	25-29 years	Digestive diseases	Alcohol use	Rate	1990	36.6	48.2	27.0
DALYs	Global	Male	25-29 years	Neoplasms	Alcohol use	Rate	1990	26.0	31.8	20.7
DALYs	Global	Female	25-29 years	Neoplasms	Alcohol use	Rate	1990	8.8	10.0	7.7
DALYs	Global	Male	25-29 years	Neurological disorders	Alcohol use	Rate	1990	45.6	64.1	31.7
DALYs	Global	Female	25-29 years	Neurological disorders	Alcohol use	Rate	1990	13.1	20.2	8.1
DALYs	Global	Male	25-29 years	Respiratory infections and tuberculosis	Alcohol use	Rate	1990	281.8	373.7	193.8
DALYs	Global	Female	25-29 years	Respiratory infections and tuberculosis	Alcohol use	Rate	1990	54.9	77.1	34.5
DALYs	Global	Male	25-29 years	Self-harm and interpersonal violence	Alcohol use	Rate	1990	441.4	587.7	300.0
DALYs	Global	Female	25-29 years	Self-harm and interpersonal violence	Alcohol use	Rate	1990	45.3	65.8	26.4
DALYs	Global	Male	25-29 years	Substance use disorders	Alcohol use	Rate	1990	560.1	780.1	386.0
DALYs	Global	Female	25-29 years	Substance use disorders	Alcohol use	Rate	1990	148.4	214.9	97.7
DALYs	Global	Male	25-29 years	Transport injuries	Alcohol use	Rate	1990	336.9	488.9	190.2
DALYs	Global	Female	25-29 years	Transport injuries	Alcohol use	Rate	1990	19.8	31.3	10.3

DALYs	Global	Male	25-29 years	Unintentional injuries	Alcohol use	Rate	1990	124.5	215.1	58.5
DALYs	Global	Female	25-29 years	Unintentional injuries	Alcohol use	Rate	1990	16.2	29.8	7.2
DALYs	Global	Male	30-34 years	Digestive diseases	Alcohol use	Rate	1990	422.0	511.2	334.2
DALYs	Global	Female	30-34 years	Digestive diseases	Alcohol use	Rate	1990	66.7	84.2	51.2
DALYs	Global	Male	30-34 years	Neoplasms	Alcohol use	Rate	1990	59.8	73.9	48.4
DALYs	Global	Female	30-34 years	Neoplasms	Alcohol use	Rate	1990	25.0	28.4	21.6
DALYs	Global	Male	30-34 years	Neurological disorders	Alcohol use	Rate	1990	45.9	64.4	32.0
DALYs	Global	Female	30-34 years	Neurological disorders	Alcohol use	Rate	1990	12.6	19.6	7.8
DALYs	Global	Male	30-34 years	Respiratory infections and tuberculosis	Alcohol use	Rate	1990	382.4	493.8	264.1
DALYs	Global	Female	30-34 years	Respiratory infections and tuberculosis	Alcohol use	Rate	1990	60.0	86.1	37.1
DALYs	Global	Male	30-34 years	Self-harm and interpersonal violence	Alcohol use	Rate	1990	448.3	594.9	304.4
DALYs	Global	Female	30-34 years	Self-harm and interpersonal violence	Alcohol use	Rate	1990	48.9	72.0	27.9
DALYs	Global	Male	30-34 years	Substance use disorders	Alcohol use	Rate	1990	721.5	992.6	533.6
DALYs	Global	Female	30-34 years	Substance use disorders	Alcohol use	Rate	1990	170.9	251.8	114.2
DALYs	Global	Male	30-34 years	Transport injuries	Alcohol use	Rate	1990	233.2	350.7	130.4
DALYs	Global	Female	30-34 years	Transport injuries	Alcohol use	Rate	1990	12.4	19.5	6.5
DALYs	Global	Male	30-34 years	Unintentional injuries	Alcohol use	Rate	1990	141.5	239.7	66.0
DALYs	Global	Female	30-34 years	Unintentional injuries	Alcohol use	Rate	1990	20.0	36.9	8.7
DALYs	Global	Male	35-39 years	Digestive diseases	Alcohol use	Rate	1990	721.5	873.4	567.3
DALYs	Global	Female	35-39 years	Digestive diseases	Alcohol use	Rate	1990	107.0	136.9	80.8
DALYs	Global	Male	35-39 years	Neoplasms	Alcohol use	Rate	1990	139.6	171.8	112.3
DALYs	Global	Female	35-39 years	Neoplasms	Alcohol use	Rate	1990	53.7	61.5	46.1
DALYs	Global	Male	35-39 years	Neurological disorders	Alcohol use	Rate	1990	46.8	64.1	32.5
DALYs	Global	Female	35-39 years	Neurological disorders	Alcohol use	Rate	1990	11.9	18.2	7.2
DALYs	Global	Male	35-39 years	Respiratory infections and tuberculosis	Alcohol use	Rate	1990	479.7	620.1	331.0
DALYs	Global	Female	35-39 years	Respiratory infections and tuberculosis	Alcohol use	Rate	1990	62.4	89.0	38.5
DALYs	Global	Male	35-39 years	Self-harm and interpersonal violence	Alcohol use	Rate	1990	424.5	575.6	285.3
DALYs	Global	Female	35-39 years	Self-harm and interpersonal violence	Alcohol use	Rate	1990	50.1	74.4	27.8
DALYs	Global	Male	35-39 years	Substance use disorders	Alcohol use	Rate	1990	794.6	1025.7	624.8
DALYs	Global	Female	35-39 years	Substance use disorders	Alcohol use	Rate	1990	182.2	246.5	135.4
DALYs	Global	Male	35-39 years	Transport injuries	Alcohol use	Rate	1990	170.3	259.1	96.2
DALYs	Global	Female	35-39 years	Transport injuries	Alcohol use	Rate	1990	11.4	18.1	6.0
DALYs	Global	Male	35-39 years	Unintentional injuries	Alcohol use	Rate	1990	157.7	269.8	74.0
DALYs	Global	Female	35-39 years	Unintentional injuries	Alcohol use	Rate	1990	22.9	42.6	9.9
DALYs	Global	Male	40-44 years	Digestive diseases	Alcohol use	Rate	1990	1084.2	1293.4	864.3
DALYs	Global	Female	40-44 years	Digestive diseases	Alcohol use	Rate	1990	174.3	216.8	132.4
DALYs	Global	Male	40-44 years	Neoplasms	Alcohol use	Rate	1990	302.6	361.5	252.5
DALYs	Global	Female	40-44 years	Neoplasms	Alcohol use	Rate	1990	103.2	118.0	89.1
DALYs	Global	Male	40-44 years	Neurological disorders	Alcohol use	Rate	1990	46.3	63.1	32.2
DALYs	Global	Female	40-44 years	Neurological disorders	Alcohol use	Rate	1990	12.6	19.1	7.5
DALYs	Global	Male	40-44 years	Respiratory infections and tuberculosis	Alcohol use	Rate	1990	601.4	770.3	419.6
DALYs	Global	Female	40-44 years	Respiratory infections and tuberculosis	Alcohol use	Rate	1990	68.5	98.5	43.2
DALYs	Global	Male	40-44 years	Self-harm and interpersonal violence	Alcohol use	Rate	1990	398.1	531.7	269.0
DALYs	Global	Female	40-44 years	Self-harm and interpersonal violence	Alcohol use	Rate	1990	53.7	80.3	29.3
DALYs	Global	Male	40-44 years	Substance use disorders	Alcohol use	Rate	1990	833.8	1056.3	666.8
DALYs	Global	Female	40-44 years	Substance use disorders	Alcohol use	Rate	1990	187.6	249.0	143.4
DALYs	Global	Male	40-44 years	Transport injuries	Alcohol use	Rate	1990	151.7	228.2	86.2
DALYs	Global	Female	40-44 years	Transport injuries	Alcohol use	Rate	1990	20.0	31.7	10.4
DALYs	Global	Male	40-44 years	Unintentional injuries	Alcohol use	Rate	1990	171.2	290.9	79.3
DALYs	Global	Female	40-44 years	Unintentional injuries	Alcohol use	Rate	1990	28.0	50.9	12.1
DALYs	Global	Male	45-49 years	Digestive diseases	Alcohol use	Rate	1990	1373.0	1657.1	1080.1

DALYs	Global	Female	45-49 years	Digestive diseases	Alcohol use	Rate	1990	244.3	308.9	183.6
DALYs	Global	Male	45-49 years	Neoplasms	Alcohol use	Rate	1990	511.6	591.3	438.3
DALYs	Global	Female	45-49 years	Neoplasms	Alcohol use	Rate	1990	159.1	182.2	136.9
DALYs	Global	Male	45-49 years	Neurological disorders	Alcohol use	Rate	1990	42.9	58.4	29.6
DALYs	Global	Female	45-49 years	Neurological disorders	Alcohol use	Rate	1990	11.9	17.7	7.0
DALYs	Global	Male	45-49 years	Respiratory infections and tuberculosis	Alcohol use	Rate	1990	756.0	990.6	519.0
DALYs	Global	Female	45-49 years	Respiratory infections and tuberculosis	Alcohol use	Rate	1990	78.5	110.9	47.8
DALYs	Global	Male	45-49 years	Self-harm and interpersonal violence	Alcohol use	Rate	1990	359.4	485.2	238.2
DALYs	Global	Female	45-49 years	Self-harm and interpersonal violence	Alcohol use	Rate	1990	53.0	77.8	29.3
DALYs	Global	Male	45-49 years	Substance use disorders	Alcohol use	Rate	1990	819.3	1000.0	675.2
DALYs	Global	Female	45-49 years	Substance use disorders	Alcohol use	Rate	1990	179.8	225.2	145.6
DALYs	Global	Male	45-49 years	Transport injuries	Alcohol use	Rate	1990	165.5	248.6	93.9
DALYs	Global	Female	45-49 years	Transport injuries	Alcohol use	Rate	1990	14.7	23.2	7.6
DALYs	Global	Male	45-49 years	Unintentional injuries	Alcohol use	Rate	1990	174.1	298.0	79.3
DALYs	Global	Female	45-49 years	Unintentional injuries	Alcohol use	Rate	1990	30.9	56.8	13.4
DALYs	Global	Male	50-54 years	Digestive diseases	Alcohol use	Rate	1990	1637.9	1959.5	1296.9
DALYs	Global	Female	50-54 years	Digestive diseases	Alcohol use	Rate	1990	329.3	418.7	243.6
DALYs	Global	Male	50-54 years	Neoplasms	Alcohol use	Rate	1990	814.7	929.4	701.7
DALYs	Global	Female	50-54 years	Neoplasms	Alcohol use	Rate	1990	220.4	251.6	191.1
DALYs	Global	Male	50-54 years	Neurological disorders	Alcohol use	Rate	1990	38.8	53.2	26.5
DALYs	Global	Female	50-54 years	Neurological disorders	Alcohol use	Rate	1990	11.2	17.1	6.6
DALYs	Global	Male	50-54 years	Respiratory infections and tuberculosis	Alcohol use	Rate	1990	863.1	1108.8	600.3
DALYs	Global	Female	50-54 years	Respiratory infections and tuberculosis	Alcohol use	Rate	1990	99.7	143.1	60.5
DALYs	Global	Male	50-54 years	Self-harm and interpersonal violence	Alcohol use	Rate	1990	347.2	464.3	228.6
DALYs	Global	Female	50-54 years	Self-harm and interpersonal violence	Alcohol use	Rate	1990	52.8	80.0	27.6
DALYs	Global	Male	50-54 years	Substance use disorders	Alcohol use	Rate	1990	821.4	1004.1	683.4
DALYs	Global	Female	50-54 years	Substance use disorders	Alcohol use	Rate	1990	196.6	242.1	160.4
DALYs	Global	Male	50-54 years	Transport injuries	Alcohol use	Rate	1990	133.6	201.4	75.8
DALYs	Global	Female	50-54 years	Transport injuries	Alcohol use	Rate	1990	16.8	27.1	8.7
DALYs	Global	Male	50-54 years	Unintentional injuries	Alcohol use	Rate	1990	194.7	329.4	90.0
DALYs	Global	Female	50-54 years	Unintentional injuries	Alcohol use	Rate	1990	36.0	66.6	15.4
DALYs	Global	Male	55-59 years	Digestive diseases	Alcohol use	Rate	1990	1776.3	2150.3	1405.0
DALYs	Global	Female	55-59 years	Digestive diseases	Alcohol use	Rate	1990	414.7	532.0	313.8
DALYs	Global	Male	55-59 years	Neoplasms	Alcohol use	Rate	1990	1079.3	1231.6	942.7
DALYs	Global	Female	55-59 years	Neoplasms	Alcohol use	Rate	1990	270.8	308.7	236.6
DALYs	Global	Male	55-59 years	Neurological disorders	Alcohol use	Rate	1990	36.9	52.5	24.4
DALYs	Global	Female	55-59 years	Neurological disorders	Alcohol use	Rate	1990	10.9	16.7	6.4
DALYs	Global	Male	55-59 years	Respiratory infections and tuberculosis	Alcohol use	Rate	1990	925.2	1200.5	638.6
DALYs	Global	Female	55-59 years	Respiratory infections and tuberculosis	Alcohol use	Rate	1990	110.2	157.2	68.5
DALYs	Global	Male	55-59 years	Self-harm and interpersonal violence	Alcohol use	Rate	1990	284.1	388.2	182.1
DALYs	Global	Female	55-59 years	Self-harm and interpersonal violence	Alcohol use	Rate	1990	45.9	70.6	24.2
DALYs	Global	Male	55-59 years	Substance use disorders	Alcohol use	Rate	1990	716.6	853.3	598.3
DALYs	Global	Female	55-59 years	Substance use disorders	Alcohol use	Rate	1990	165.2	200.4	137.9
DALYs	Global	Male	55-59 years	Transport injuries	Alcohol use	Rate	1990	134.0	203.0	75.5
DALYs	Global	Female	55-59 years	Transport injuries	Alcohol use	Rate	1990	21.0	34.0	11.0
DALYs	Global	Male	55-59 years	Unintentional injuries	Alcohol use	Rate	1990	182.3	312.7	84.7
DALYs	Global	Female	55-59 years	Unintentional injuries	Alcohol use	Rate	1990	37.7	68.0	16.8
DALYs	Global	Male	60-64 years	Digestive diseases	Alcohol use	Rate	1990	1794.6	2168.1	1421.8
DALYs	Global	Female	60-64 years	Digestive diseases	Alcohol use	Rate	1990	467.7	602.4	354.8
DALYs	Global	Male	60-64 years	Neoplasms	Alcohol use	Rate	1990	1253.6	1415.1	1099.1
DALYs	Global	Female	60-64 years	Neoplasms	Alcohol use	Rate	1990	314.8	357.3	273.3

DALYs	Global	Male	60-64 years	Neurological disorders	Alcohol use	Rate	1990	38.0	54.6	25.3
DALYs	Global	Female	60-64 years	Neurological disorders	Alcohol use	Rate	1990	11.8	18.8	6.9
DALYs	Global	Male	60-64 years	Respiratory infections and tuberculosis	Alcohol use	Rate	1990	980.4	1267.5	680.9
DALYs	Global	Female	60-64 years	Respiratory infections and tuberculosis	Alcohol use	Rate	1990	113.5	160.4	71.5
DALYs	Global	Male	60-64 years	Self-harm and interpersonal violence	Alcohol use	Rate	1990	240.0	330.9	152.9
DALYs	Global	Female	60-64 years	Self-harm and interpersonal violence	Alcohol use	Rate	1990	42.1	64.6	21.9
DALYs	Global	Male	60-64 years	Substance use disorders	Alcohol use	Rate	1990	614.8	751.7	503.1
DALYs	Global	Female	60-64 years	Substance use disorders	Alcohol use	Rate	1990	143.3	180.6	116.9
DALYs	Global	Male	60-64 years	Transport injuries	Alcohol use	Rate	1990	105.1	157.7	59.4
DALYs	Global	Female	60-64 years	Transport injuries	Alcohol use	Rate	1990	13.6	21.8	7.1
DALYs	Global	Male	60-64 years	Unintentional injuries	Alcohol use	Rate	1990	181.2	311.2	83.5
DALYs	Global	Female	60-64 years	Unintentional injuries	Alcohol use	Rate	1990	43.1	80.0	19.1
DALYs	Global	Male	65-69 years	Digestive diseases	Alcohol use	Rate	1990	1681.1	2020.5	1343.3
DALYs	Global	Female	65-69 years	Digestive diseases	Alcohol use	Rate	1990	480.1	606.5	361.0
DALYs	Global	Male	65-69 years	Neoplasms	Alcohol use	Rate	1990	1328.8	1508.5	1171.1
DALYs	Global	Female	65-69 years	Neoplasms	Alcohol use	Rate	1990	353.3	404.0	304.5
DALYs	Global	Male	65-69 years	Neurological disorders	Alcohol use	Rate	1990	38.3	55.3	25.2
DALYs	Global	Female	65-69 years	Neurological disorders	Alcohol use	Rate	1990	12.8	20.4	7.3
DALYs	Global	Male	65-69 years	Respiratory infections and tuberculosis	Alcohol use	Rate	1990	1005.9	1288.4	702.6
DALYs	Global	Female	65-69 years	Respiratory infections and tuberculosis	Alcohol use	Rate	1990	113.2	161.2	72.0
DALYs	Global	Male	65-69 years	Self-harm and interpersonal violence	Alcohol use	Rate	1990	197.7	277.9	122.6
DALYs	Global	Female	65-69 years	Self-harm and interpersonal violence	Alcohol use	Rate	1990	38.0	58.0	18.8
DALYs	Global	Male	65-69 years	Substance use disorders	Alcohol use	Rate	1990	451.7	552.3	367.4
DALYs	Global	Female	65-69 years	Substance use disorders	Alcohol use	Rate	1990	114.6	142.3	94.2
DALYs	Global	Male	65-69 years	Transport injuries	Alcohol use	Rate	1990	95.6	144.1	53.9
DALYs	Global	Female	65-69 years	Transport injuries	Alcohol use	Rate	1990	11.6	18.7	6.0
DALYs	Global	Male	65-69 years	Unintentional injuries	Alcohol use	Rate	1990	166.1	287.4	75.7
DALYs	Global	Female	65-69 years	Unintentional injuries	Alcohol use	Rate	1990	48.1	87.2	21.0
DALYs	Global	Male	70-74 years	Digestive diseases	Alcohol use	Rate	1990	1409.3	1718.0	1104.1
DALYs	Global	Female	70-74 years	Digestive diseases	Alcohol use	Rate	1990	453.5	576.3	346.0
DALYs	Global	Male	70-74 years	Neoplasms	Alcohol use	Rate	1990	1260.3	1443.9	1090.9
DALYs	Global	Female	70-74 years	Neoplasms	Alcohol use	Rate	1990	355.3	408.4	303.7
DALYs	Global	Male	70-74 years	Neurological disorders	Alcohol use	Rate	1990	37.3	54.2	24.3
DALYs	Global	Female	70-74 years	Neurological disorders	Alcohol use	Rate	1990	13.0	20.6	7.4
DALYs	Global	Male	70-74 years	Respiratory infections and tuberculosis	Alcohol use	Rate	1990	1117.9	1466.8	778.5
DALYs	Global	Female	70-74 years	Respiratory infections and tuberculosis	Alcohol use	Rate	1990	145.5	208.5	93.4
DALYs	Global	Male	70-74 years	Self-harm and interpersonal violence	Alcohol use	Rate	1990	174.4	250.5	105.7
DALYs	Global	Female	70-74 years	Self-harm and interpersonal violence	Alcohol use	Rate	1990	34.3	53.5	16.8
DALYs	Global	Male	70-74 years	Substance use disorders	Alcohol use	Rate	1990	338.7	433.8	267.9
DALYs	Global	Female	70-74 years	Substance use disorders	Alcohol use	Rate	1990	83.5	109.9	64.8
DALYs	Global	Male	70-74 years	Transport injuries	Alcohol use	Rate	1990	99.6	150.3	56.1
DALYs	Global	Female	70-74 years	Transport injuries	Alcohol use	Rate	1990	28.7	46.4	14.8
DALYs	Global	Male	70-74 years	Unintentional injuries	Alcohol use	Rate	1990	160.7	278.0	71.9
DALYs	Global	Female	70-74 years	Unintentional injuries	Alcohol use	Rate	1990	55.4	102.7	24.4
DALYs	Global	Male	75-79 years	Digestive diseases	Alcohol use	Rate	1990	1229.8	1476.2	988.5
DALYs	Global	Female	75-79 years	Digestive diseases	Alcohol use	Rate	1990	414.3	524.3	316.2
DALYs	Global	Male	75-79 years	Neoplasms	Alcohol use	Rate	1990	1146.0	1293.3	1007.2
DALYs	Global	Female	75-79 years	Neoplasms	Alcohol use	Rate	1990	354.4	403.3	303.4
DALYs	Global	Male	75-79 years	Neurological disorders	Alcohol use	Rate	1990	39.9	57.5	26.0
DALYs	Global	Female	75-79 years	Neurological disorders	Alcohol use	Rate	1990	13.9	22.0	7.8
DALYs	Global	Male	75-79 years	Respiratory infections and tuberculosis	Alcohol use	Rate	1990	995.0	1286.5	708.4

DALYs	Global	Female	75-79 years	Respiratory infections and tuberculosis	Alcohol use	Rate	1990	134.6	196.1	79.1
DALYs	Global	Male	75-79 years	Self-harm and interpersonal violence	Alcohol use	Rate	1990	170.1	246.1	100.2
DALYs	Global	Female	75-79 years	Self-harm and interpersonal violence	Alcohol use	Rate	1990	31.4	49.9	15.0
DALYs	Global	Male	75-79 years	Substance use disorders	Alcohol use	Rate	1990	294.9	370.2	236.2
DALYs	Global	Female	75-79 years	Substance use disorders	Alcohol use	Rate	1990	82.0	106.1	64.4
DALYs	Global	Male	75-79 years	Transport injuries	Alcohol use	Rate	1990	147.3	220.4	84.0
DALYs	Global	Female	75-79 years	Transport injuries	Alcohol use	Rate	1990	28.2	46.2	14.6
DALYs	Global	Male	75-79 years	Unintentional injuries	Alcohol use	Rate	1990	188.8	325.4	87.0
DALYs	Global	Female	75-79 years	Unintentional injuries	Alcohol use	Rate	1990	78.4	143.4	34.1
DALYs	Global	Male	80-84	Digestive diseases	Alcohol use	Rate	1990	972.5	1187.6	767.6
DALYs	Global	Female	80-84	Digestive diseases	Alcohol use	Rate	1990	370.3	474.0	276.7
DALYs	Global	Male	80-84	Neoplasms	Alcohol use	Rate	1990	897.4	1018.4	775.3
DALYs	Global	Female	80-84	Neoplasms	Alcohol use	Rate	1990	325.5	378.0	273.6
DALYs	Global	Male	80-84	Neurological disorders	Alcohol use	Rate	1990	37.6	55.7	23.6
DALYs	Global	Female	80-84	Neurological disorders	Alcohol use	Rate	1990	14.5	23.0	8.1
DALYs	Global	Male	80-84	Respiratory infections and tuberculosis	Alcohol use	Rate	1990	921.2	1210.3	628.7
DALYs	Global	Female	80-84	Respiratory infections and tuberculosis	Alcohol use	Rate	1990	156.6	228.5	87.0
DALYs	Global	Male	80-84	Self-harm and interpersonal violence	Alcohol use	Rate	1990	144.1	214.0	79.2
DALYs	Global	Female	80-84	Self-harm and interpersonal violence	Alcohol use	Rate	1990	25.4	40.5	11.4
DALYs	Global	Male	80-84	Substance use disorders	Alcohol use	Rate	1990	215.3	277.6	168.2
DALYs	Global	Female	80-84	Substance use disorders	Alcohol use	Rate	1990	72.7	97.8	53.6
DALYs	Global	Male	80-84	Transport injuries	Alcohol use	Rate	1990	190.1	271.0	111.0
DALYs	Global	Female	80-84	Transport injuries	Alcohol use	Rate	1990	52.7	85.6	27.5
DALYs	Global	Male	80-84	Unintentional injuries	Alcohol use	Rate	1990	210.2	367.8	98.3
DALYs	Global	Female	80-84	Unintentional injuries	Alcohol use	Rate	1990	112.1	205.8	50.5
DALYs	Global	Male	85-89	Digestive diseases	Alcohol use	Rate	1990	866.2	1056.8	688.1
DALYs	Global	Female	85-89	Digestive diseases	Alcohol use	Rate	1990	344.6	443.5	254.8
DALYs	Global	Male	85-89	Neoplasms	Alcohol use	Rate	1990	808.2	919.7	688.8
DALYs	Global	Female	85-89	Neoplasms	Alcohol use	Rate	1990	342.4	401.9	279.2
DALYs	Global	Male	85-89	Neurological disorders	Alcohol use	Rate	1990	41.0	60.6	25.6
DALYs	Global	Female	85-89	Neurological disorders	Alcohol use	Rate	1990	16.5	26.5	9.4
DALYs	Global	Male	85-89	Respiratory infections and tuberculosis	Alcohol use	Rate	1990	1080.7	1432.0	692.8
DALYs	Global	Female	85-89	Respiratory infections and tuberculosis	Alcohol use	Rate	1990	216.7	333.4	103.6
DALYs	Global	Male	85-89	Self-harm and interpersonal violence	Alcohol use	Rate	1990	140.3	205.5	76.5
DALYs	Global	Female	85-89	Self-harm and interpersonal violence	Alcohol use	Rate	1990	23.0	36.3	10.6
DALYs	Global	Male	85-89	Substance use disorders	Alcohol use	Rate	1990	191.0	244.1	148.4
DALYs	Global	Female	85-89	Substance use disorders	Alcohol use	Rate	1990	80.8	108.2	59.9
DALYs	Global	Male	85-89	Transport injuries	Alcohol use	Rate	1990	172.9	249.1	101.1
DALYs	Global	Female	85-89	Transport injuries	Alcohol use	Rate	1990	48.4	78.9	25.7
DALYs	Global	Male	85-89	Unintentional injuries	Alcohol use	Rate	1990	280.5	483.8	132.7
DALYs	Global	Female	85-89	Unintentional injuries	Alcohol use	Rate	1990	176.8	319.2	79.7
DALYs	Global	Male	90-94	Digestive diseases	Alcohol use	Rate	1990	756.0	933.1	582.3
DALYs	Global	Female	90-94	Digestive diseases	Alcohol use	Rate	1990	331.2	440.6	229.1
DALYs	Global	Male	90-94	Neoplasms	Alcohol use	Rate	1990	634.0	734.5	526.2
DALYs	Global	Female	90-94	Neoplasms	Alcohol use	Rate	1990	334.1	405.6	261.4
DALYs	Global	Male	90-94	Neurological disorders	Alcohol use	Rate	1990	43.3	65.6	26.2
DALYs	Global	Female	90-94	Neurological disorders	Alcohol use	Rate	1990	18.5	29.7	10.9
DALYs	Global	Male	90-94	Respiratory infections and tuberculosis	Alcohol use	Rate	1990	1148.1	1575.1	700.7
DALYs	Global	Female	90-94	Respiratory infections and tuberculosis	Alcohol use	Rate	1990	268.6	431.2	112.4
DALYs	Global	Male	90-94	Self-harm and interpersonal violence	Alcohol use	Rate	1990	104.2	150.7	57.9
DALYs	Global	Female	90-94	Self-harm and interpersonal violence	Alcohol use	Rate	1990	16.7	26.5	8.2

DALYs	Global	Male	90-94	Substance use disorders	Alcohol use	Rate	1990	177.8	239.1	131.7
DALYs	Global	Female	90-94	Substance use disorders	Alcohol use	Rate	1990	90.2	127.9	62.4
DALYs	Global	Male	90-94	Transport injuries	Alcohol use	Rate	1990	148.4	216.3	87.9
DALYs	Global	Female	90-94	Transport injuries	Alcohol use	Rate	1990	43.5	70.9	23.0
DALYs	Global	Male	90-94	Unintentional injuries	Alcohol use	Rate	1990	346.4	603.3	163.2
DALYs	Global	Female	90-94	Unintentional injuries	Alcohol use	Rate	1990	248.5	448.6	113.2
DALYs	Global	Male	95+ years	Digestive diseases	Alcohol use	Rate	1990	728.9	936.4	518.5
DALYs	Global	Female	95+ years	Digestive diseases	Alcohol use	Rate	1990	345.8	482.9	217.7
DALYs	Global	Male	95+ years	Neoplasms	Alcohol use	Rate	1990	485.4	573.2	387.2
DALYs	Global	Female	95+ years	Neoplasms	Alcohol use	Rate	1990	313.6	382.0	235.1
DALYs	Global	Male	95+ years	Neurological disorders	Alcohol use	Rate	1990	44.8	69.5	26.6
DALYs	Global	Female	95+ years	Neurological disorders	Alcohol use	Rate	1990	20.1	32.7	11.3
DALYs	Global	Male	95+ years	Respiratory infections and tuberculosis	Alcohol use	Rate	1990	1172.9	1701.7	644.9
DALYs	Global	Female	95+ years	Respiratory infections and tuberculosis	Alcohol use	Rate	1990	339.9	564.9	131.9
DALYs	Global	Male	95+ years	Self-harm and interpersonal violence	Alcohol use	Rate	1990	72.9	101.6	44.2
DALYs	Global	Female	95+ years	Self-harm and interpersonal violence	Alcohol use	Rate	1990	11.2	17.5	5.9
DALYs	Global	Male	95+ years	Substance use disorders	Alcohol use	Rate	1990	220.6	315.7	155.6
DALYs	Global	Female	95+ years	Substance use disorders	Alcohol use	Rate	1990	119.1	180.7	78.9
DALYs	Global	Male	95+ years	Transport injuries	Alcohol use	Rate	1990	148.0	217.9	87.6
DALYs	Global	Female	95+ years	Transport injuries	Alcohol use	Rate	1990	46.0	74.5	24.5
DALYs	Global	Male	95+ years	Unintentional injuries	Alcohol use	Rate	1990	376.3	656.4	178.0
DALYs	Global	Female	95+ years	Unintentional injuries	Alcohol use	Rate	1990	302.7	549.3	139.0
DALYs	Global	Male	15-19 years	Digestive diseases	Alcohol use	Rate	2019	28.3	39.5	19.0
DALYs	Global	Female	15-19 years	Digestive diseases	Alcohol use	Rate	2019	8.8	12.8	5.7
DALYs	Global	Male	15-19 years	Neoplasms	Alcohol use	Rate	2019	2.5	3.0	2.0
DALYs	Global	Female	15-19 years	Neoplasms	Alcohol use	Rate	2019	0.8	1.0	0.7
DALYs	Global	Male	15-19 years	Neurological disorders	Alcohol use	Rate	2019	18.1	28.1	11.0
DALYs	Global	Female	15-19 years	Neurological disorders	Alcohol use	Rate	2019	7.0	11.6	4.0
DALYs	Global	Male	15-19 years	Respiratory infections and tuberculosis	Alcohol use	Rate	2019	24.3	34.5	14.6
DALYs	Global	Female	15-19 years	Respiratory infections and tuberculosis	Alcohol use	Rate	2019	8.8	13.0	4.8
DALYs	Global	Male	15-19 years	Self-harm and interpersonal violence	Alcohol use	Rate	2019	97.6	135.1	62.4
DALYs	Global	Female	15-19 years	Self-harm and interpersonal violence	Alcohol use	Rate	2019	14.2	21.2	8.0
DALYs	Global	Male	15-19 years	Substance use disorders	Alcohol use	Rate	2019	90.2	136.3	55.9
DALYs	Global	Female	15-19 years	Substance use disorders	Alcohol use	Rate	2019	37.6	59.8	22.2
DALYs	Global	Male	15-19 years	Transport injuries	Alcohol use	Rate	2019	83.0	129.3	46.0
DALYs	Global	Female	15-19 years	Transport injuries	Alcohol use	Rate	2019	10.6	17.0	5.4
DALYs	Global	Male	15-19 years	Unintentional injuries	Alcohol use	Rate	2019	25.0	44.3	11.2
DALYs	Global	Female	15-19 years	Unintentional injuries	Alcohol use	Rate	2019	5.0	9.3	2.1
DALYs	Global	Male	20-24 years	Digestive diseases	Alcohol use	Rate	2019	106.3	141.1	76.5
DALYs	Global	Female	20-24 years	Digestive diseases	Alcohol use	Rate	2019	19.7	27.7	13.5
DALYs	Global	Male	20-24 years	Neoplasms	Alcohol use	Rate	2019	12.2	14.7	10.2
DALYs	Global	Female	20-24 years	Neoplasms	Alcohol use	Rate	2019	3.1	3.6	2.6
DALYs	Global	Male	20-24 years	Neurological disorders	Alcohol use	Rate	2019	39.2	57.1	25.7
DALYs	Global	Female	20-24 years	Neurological disorders	Alcohol use	Rate	2019	10.4	16.5	6.0
DALYs	Global	Male	20-24 years	Respiratory infections and tuberculosis	Alcohol use	Rate	2019	121.3	161.7	79.5
DALYs	Global	Female	20-24 years	Respiratory infections and tuberculosis	Alcohol use	Rate	2019	26.7	38.2	16.1
DALYs	Global	Male	20-24 years	Self-harm and interpersonal violence	Alcohol use	Rate	2019	296.8	398.9	199.7
DALYs	Global	Female	20-24 years	Self-harm and interpersonal violence	Alcohol use	Rate	2019	26.5	37.6	15.9
DALYs	Global	Male	20-24 years	Substance use disorders	Alcohol use	Rate	2019	279.2	424.1	181.4
DALYs	Global	Female	20-24 years	Substance use disorders	Alcohol use	Rate	2019	92.3	150.9	54.1
DALYs	Global	Male	20-24 years	Transport injuries	Alcohol use	Rate	2019	270.6	392.3	152.9

DALYs	Global	Female	20-24 years	Transport injuries	Alcohol use	Rate	2019	15.7	25.3	8.1
DALYs	Global	Male	20-24 years	Unintentional injuries	Alcohol use	Rate	2019	63.6	108.9	30.3
DALYs	Global	Female	20-24 years	Unintentional injuries	Alcohol use	Rate	2019	8.7	15.9	3.7
DALYs	Global	Male	25-29 years	Digestive diseases	Alcohol use	Rate	2019	232.0	292.0	175.3
DALYs	Global	Female	25-29 years	Digestive diseases	Alcohol use	Rate	2019	33.4	44.8	24.2
DALYs	Global	Male	25-29 years	Neoplasms	Alcohol use	Rate	2019	25.0	29.5	21.1
DALYs	Global	Female	25-29 years	Neoplasms	Alcohol use	Rate	2019	6.9	8.0	5.9
DALYs	Global	Male	25-29 years	Neurological disorders	Alcohol use	Rate	2019	44.1	63.0	30.1
DALYs	Global	Female	25-29 years	Neurological disorders	Alcohol use	Rate	2019	10.2	15.9	6.0
DALYs	Global	Male	25-29 years	Respiratory infections and tuberculosis	Alcohol use	Rate	2019	201.7	263.2	136.9
DALYs	Global	Female	25-29 years	Respiratory infections and tuberculosis	Alcohol use	Rate	2019	31.9	45.1	19.8
DALYs	Global	Male	25-29 years	Self-harm and interpersonal violence	Alcohol use	Rate	2019	346.2	455.7	237.4
DALYs	Global	Female	25-29 years	Self-harm and interpersonal violence	Alcohol use	Rate	2019	27.8	39.9	16.6
DALYs	Global	Male	25-29 years	Substance use disorders	Alcohol use	Rate	2019	433.3	611.2	296.1
DALYs	Global	Female	25-29 years	Substance use disorders	Alcohol use	Rate	2019	117.0	173.6	73.9
DALYs	Global	Male	25-29 years	Transport injuries	Alcohol use	Rate	2019	274.1	397.5	157.5
DALYs	Global	Female	25-29 years	Transport injuries	Alcohol use	Rate	2019	11.7	18.6	6.0
DALYs	Global	Male	25-29 years	Unintentional injuries	Alcohol use	Rate	2019	79.5	137.4	37.3
DALYs	Global	Female	25-29 years	Unintentional injuries	Alcohol use	Rate	2019	10.4	19.4	4.5
DALYs	Global	Male	30-34 years	Digestive diseases	Alcohol use	Rate	2019	400.9	493.2	307.9
DALYs	Global	Female	30-34 years	Digestive diseases	Alcohol use	Rate	2019	59.1	75.2	44.6
DALYs	Global	Male	30-34 years	Neoplasms	Alcohol use	Rate	2019	57.2	67.0	48.1
DALYs	Global	Female	30-34 years	Neoplasms	Alcohol use	Rate	2019	16.8	19.3	14.2
DALYs	Global	Male	30-34 years	Neurological disorders	Alcohol use	Rate	2019	42.3	60.1	28.4
DALYs	Global	Female	30-34 years	Neurological disorders	Alcohol use	Rate	2019	9.2	14.2	5.5
DALYs	Global	Male	30-34 years	Respiratory infections and tuberculosis	Alcohol use	Rate	2019	258.2	335.1	177.5
DALYs	Global	Female	30-34 years	Respiratory infections and tuberculosis	Alcohol use	Rate	2019	33.2	46.8	20.5
DALYs	Global	Male	30-34 years	Self-harm and interpersonal violence	Alcohol use	Rate	2019	333.7	440.3	226.7
DALYs	Global	Female	30-34 years	Self-harm and interpersonal violence	Alcohol use	Rate	2019	27.6	39.9	16.6
DALYs	Global	Male	30-34 years	Substance use disorders	Alcohol use	Rate	2019	565.9	788.8	412.3
DALYs	Global	Female	30-34 years	Substance use disorders	Alcohol use	Rate	2019	131.1	198.4	85.3
DALYs	Global	Male	30-34 years	Transport injuries	Alcohol use	Rate	2019	185.0	284.7	106.0
DALYs	Global	Female	30-34 years	Transport injuries	Alcohol use	Rate	2019	7.4	11.7	3.8
DALYs	Global	Male	30-34 years	Unintentional injuries	Alcohol use	Rate	2019	91.4	155.9	42.8
DALYs	Global	Female	30-34 years	Unintentional injuries	Alcohol use	Rate	2019	12.5	23.0	5.4
DALYs	Global	Male	35-39 years	Digestive diseases	Alcohol use	Rate	2019	656.4	808.1	505.1
DALYs	Global	Female	35-39 years	Digestive diseases	Alcohol use	Rate	2019	90.8	116.0	66.7
DALYs	Global	Male	35-39 years	Neoplasms	Alcohol use	Rate	2019	115.2	134.6	98.0
DALYs	Global	Female	35-39 years	Neoplasms	Alcohol use	Rate	2019	34.3	39.9	29.4
DALYs	Global	Male	35-39 years	Neurological disorders	Alcohol use	Rate	2019	43.5	60.4	29.7
DALYs	Global	Female	35-39 years	Neurological disorders	Alcohol use	Rate	2019	9.1	14.0	5.4
DALYs	Global	Male	35-39 years	Respiratory infections and tuberculosis	Alcohol use	Rate	2019	321.5	412.6	224.0
DALYs	Global	Female	35-39 years	Respiratory infections and tuberculosis	Alcohol use	Rate	2019	35.6	49.6	22.0
DALYs	Global	Male	35-39 years	Self-harm and interpersonal violence	Alcohol use	Rate	2019	319.6	424.1	220.0
DALYs	Global	Female	35-39 years	Self-harm and interpersonal violence	Alcohol use	Rate	2019	28.6	41.3	16.6
DALYs	Global	Male	35-39 years	Substance use disorders	Alcohol use	Rate	2019	636.5	840.6	492.4
DALYs	Global	Female	35-39 years	Substance use disorders	Alcohol use	Rate	2019	142.6	198.3	102.2
DALYs	Global	Male	35-39 years	Transport injuries	Alcohol use	Rate	2019	139.5	216.8	79.4
DALYs	Global	Female	35-39 years	Transport injuries	Alcohol use	Rate	2019	6.9	11.2	3.6
DALYs	Global	Male	35-39 years	Unintentional injuries	Alcohol use	Rate	2019	105.3	181.5	49.2
DALYs	Global	Female	35-39 years	Unintentional injuries	Alcohol use	Rate	2019	15.3	28.7	6.8

DALYs	Global	Male	40-44 years	Digestive diseases	Alcohol use	Rate	2019	895.7	1096.7	693.1
DALYs	Global	Female	40-44 years	Digestive diseases	Alcohol use	Rate	2019	137.5	174.0	101.5
DALYs	Global	Male	40-44 years	Neoplasms	Alcohol use	Rate	2019	227.8	261.6	195.1
DALYs	Global	Female	40-44 years	Neoplasms	Alcohol use	Rate	2019	62.3	72.3	52.8
DALYs	Global	Male	40-44 years	Neurological disorders	Alcohol use	Rate	2019	42.0	57.9	28.3
DALYs	Global	Female	40-44 years	Neurological disorders	Alcohol use	Rate	2019	9.9	14.8	5.8
DALYs	Global	Male	40-44 years	Respiratory infections and tuberculosis	Alcohol use	Rate	2019	357.1	452.7	248.8
DALYs	Global	Female	40-44 years	Respiratory infections and tuberculosis	Alcohol use	Rate	2019	39.5	57.7	24.2
DALYs	Global	Male	40-44 years	Self-harm and interpersonal violence	Alcohol use	Rate	2019	282.0	377.4	192.5
DALYs	Global	Female	40-44 years	Self-harm and interpersonal violence	Alcohol use	Rate	2019	30.5	43.6	18.4
DALYs	Global	Male	40-44 years	Substance use disorders	Alcohol use	Rate	2019	675.5	875.3	529.4
DALYs	Global	Female	40-44 years	Substance use disorders	Alcohol use	Rate	2019	144.1	198.2	104.2
DALYs	Global	Male	40-44 years	Transport injuries	Alcohol use	Rate	2019	119.1	186.6	67.8
DALYs	Global	Female	40-44 years	Transport injuries	Alcohol use	Rate	2019	10.0	16.0	5.2
DALYs	Global	Male	40-44 years	Unintentional injuries	Alcohol use	Rate	2019	116.4	198.2	54.4
DALYs	Global	Female	40-44 years	Unintentional injuries	Alcohol use	Rate	2019	18.5	34.3	8.2
DALYs	Global	Male	45-49 years	Digestive diseases	Alcohol use	Rate	2019	1073.7	1314.7	836.7
DALYs	Global	Female	45-49 years	Digestive diseases	Alcohol use	Rate	2019	173.0	221.7	129.3
DALYs	Global	Male	45-49 years	Neoplasms	Alcohol use	Rate	2019	392.4	453.0	335.4
DALYs	Global	Female	45-49 years	Neoplasms	Alcohol use	Rate	2019	94.4	108.5	80.5
DALYs	Global	Male	45-49 years	Neurological disorders	Alcohol use	Rate	2019	38.9	53.5	26.1
DALYs	Global	Female	45-49 years	Neurological disorders	Alcohol use	Rate	2019	9.4	14.4	5.5
DALYs	Global	Male	45-49 years	Respiratory infections and tuberculosis	Alcohol use	Rate	2019	388.5	487.9	272.3
DALYs	Global	Female	45-49 years	Respiratory infections and tuberculosis	Alcohol use	Rate	2019	40.2	57.2	24.8
DALYs	Global	Male	45-49 years	Self-harm and interpersonal violence	Alcohol use	Rate	2019	248.2	333.2	170.3
DALYs	Global	Female	45-49 years	Self-harm and interpersonal violence	Alcohol use	Rate	2019	30.1	43.6	17.5
DALYs	Global	Male	45-49 years	Substance use disorders	Alcohol use	Rate	2019	680.4	849.6	550.2
DALYs	Global	Female	45-49 years	Substance use disorders	Alcohol use	Rate	2019	140.0	181.9	109.2
DALYs	Global	Male	45-49 years	Transport injuries	Alcohol use	Rate	2019	111.4	175.7	63.5
DALYs	Global	Female	45-49 years	Transport injuries	Alcohol use	Rate	2019	7.1	11.4	3.8
DALYs	Global	Male	45-49 years	Unintentional injuries	Alcohol use	Rate	2019	124.1	212.0	56.9
DALYs	Global	Female	45-49 years	Unintentional injuries	Alcohol use	Rate	2019	20.8	38.3	9.2
DALYs	Global	Male	50-54 years	Digestive diseases	Alcohol use	Rate	2019	1230.3	1508.6	939.5
DALYs	Global	Female	50-54 years	Digestive diseases	Alcohol use	Rate	2019	225.9	294.9	164.6
DALYs	Global	Male	50-54 years	Neoplasms	Alcohol use	Rate	2019	633.9	723.7	539.4
DALYs	Global	Female	50-54 years	Neoplasms	Alcohol use	Rate	2019	137.4	158.4	117.5
DALYs	Global	Male	50-54 years	Neurological disorders	Alcohol use	Rate	2019	35.6	50.2	23.6
DALYs	Global	Female	50-54 years	Neurological disorders	Alcohol use	Rate	2019	9.4	14.4	5.5
DALYs	Global	Male	50-54 years	Respiratory infections and tuberculosis	Alcohol use	Rate	2019	408.9	522.8	289.0
DALYs	Global	Female	50-54 years	Respiratory infections and tuberculosis	Alcohol use	Rate	2019	50.6	72.9	31.2
DALYs	Global	Male	50-54 years	Self-harm and interpersonal violence	Alcohol use	Rate	2019	219.0	295.5	147.2
DALYs	Global	Female	50-54 years	Self-harm and interpersonal violence	Alcohol use	Rate	2019	28.5	41.7	16.1
DALYs	Global	Male	50-54 years	Substance use disorders	Alcohol use	Rate	2019	622.5	783.1	496.1
DALYs	Global	Female	50-54 years	Substance use disorders	Alcohol use	Rate	2019	132.6	176.8	101.6
DALYs	Global	Male	50-54 years	Transport injuries	Alcohol use	Rate	2019	93.4	147.2	52.9
DALYs	Global	Female	50-54 years	Transport injuries	Alcohol use	Rate	2019	8.7	14.0	4.6
DALYs	Global	Male	50-54 years	Unintentional injuries	Alcohol use	Rate	2019	130.9	227.5	59.2
DALYs	Global	Female	50-54 years	Unintentional injuries	Alcohol use	Rate	2019	23.9	44.5	10.7
DALYs	Global	Male	55-59 years	Digestive diseases	Alcohol use	Rate	2019	1389.9	1701.8	1069.2
DALYs	Global	Female	55-59 years	Digestive diseases	Alcohol use	Rate	2019	311.5	405.7	226.9
DALYs	Global	Male	55-59 years	Neoplasms	Alcohol use	Rate	2019	890.2	1017.5	772.3

DALYs	Global	Female	55-59 years	Neoplasms	Alcohol use	Rate	2019	182.2	208.1	157.4
DALYs	Global	Male	55-59 years	Neurological disorders	Alcohol use	Rate	2019	37.3	53.7	25.1
DALYs	Global	Female	55-59 years	Neurological disorders	Alcohol use	Rate	2019	10.1	15.9	6.0
DALYs	Global	Male	55-59 years	Respiratory infections and tuberculosis	Alcohol use	Rate	2019	461.5	583.9	320.7
DALYs	Global	Female	55-59 years	Respiratory infections and tuberculosis	Alcohol use	Rate	2019	57.1	79.9	35.7
DALYs	Global	Male	55-59 years	Self-harm and interpersonal violence	Alcohol use	Rate	2019	205.3	277.7	134.4
DALYs	Global	Female	55-59 years	Self-harm and interpersonal violence	Alcohol use	Rate	2019	26.7	38.9	15.3
DALYs	Global	Male	55-59 years	Substance use disorders	Alcohol use	Rate	2019	631.3	772.6	520.8
DALYs	Global	Female	55-59 years	Substance use disorders	Alcohol use	Rate	2019	133.0	168.8	105.8
DALYs	Global	Male	55-59 years	Transport injuries	Alcohol use	Rate	2019	94.0	147.1	52.9
DALYs	Global	Female	55-59 years	Transport injuries	Alcohol use	Rate	2019	11.8	19.2	6.2
DALYs	Global	Male	55-59 years	Unintentional injuries	Alcohol use	Rate	2019	140.1	241.8	64.5
DALYs	Global	Female	55-59 years	Unintentional injuries	Alcohol use	Rate	2019	28.8	54.4	12.8
DALYs	Global	Male	60-64 years	Digestive diseases	Alcohol use	Rate	2019	1412.5	1726.6	1091.8
DALYs	Global	Female	60-64 years	Digestive diseases	Alcohol use	Rate	2019	352.9	459.6	260.8
DALYs	Global	Male	60-64 years	Neoplasms	Alcohol use	Rate	2019	1090.9	1245.2	946.4
DALYs	Global	Female	60-64 years	Neoplasms	Alcohol use	Rate	2019	214.8	245.4	185.7
DALYs	Global	Male	60-64 years	Neurological disorders	Alcohol use	Rate	2019	39.1	57.7	25.7
DALYs	Global	Female	60-64 years	Neurological disorders	Alcohol use	Rate	2019	11.0	17.4	6.4
DALYs	Global	Male	60-64 years	Respiratory infections and tuberculosis	Alcohol use	Rate	2019	468.4	597.6	336.8
DALYs	Global	Female	60-64 years	Respiratory infections and tuberculosis	Alcohol use	Rate	2019	59.8	84.3	37.4
DALYs	Global	Male	60-64 years	Self-harm and interpersonal violence	Alcohol use	Rate	2019	169.6	227.8	110.3
DALYs	Global	Female	60-64 years	Self-harm and interpersonal violence	Alcohol use	Rate	2019	22.8	33.7	12.6
DALYs	Global	Male	60-64 years	Substance use disorders	Alcohol use	Rate	2019	563.3	702.3	455.4
DALYs	Global	Female	60-64 years	Substance use disorders	Alcohol use	Rate	2019	117.8	155.0	91.6
DALYs	Global	Male	60-64 years	Transport injuries	Alcohol use	Rate	2019	79.8	124.7	44.7
DALYs	Global	Female	60-64 years	Transport injuries	Alcohol use	Rate	2019	7.1	11.6	3.7
DALYs	Global	Male	60-64 years	Unintentional injuries	Alcohol use	Rate	2019	146.6	255.3	67.2
DALYs	Global	Female	60-64 years	Unintentional injuries	Alcohol use	Rate	2019	33.5	60.4	14.7
DALYs	Global	Male	65-69 years	Digestive diseases	Alcohol use	Rate	2019	1256.5	1533.6	974.4
DALYs	Global	Female	65-69 years	Digestive diseases	Alcohol use	Rate	2019	335.9	439.6	244.7
DALYs	Global	Male	65-69 years	Neoplasms	Alcohol use	Rate	2019	1190.3	1363.2	1032.5
DALYs	Global	Female	65-69 years	Neoplasms	Alcohol use	Rate	2019	233.3	271.2	200.2
DALYs	Global	Male	65-69 years	Neurological disorders	Alcohol use	Rate	2019	40.4	59.9	25.8
DALYs	Global	Female	65-69 years	Neurological disorders	Alcohol use	Rate	2019	11.5	18.9	6.7
DALYs	Global	Male	65-69 years	Respiratory infections and tuberculosis	Alcohol use	Rate	2019	469.2	603.8	336.7
DALYs	Global	Female	65-69 years	Respiratory infections and tuberculosis	Alcohol use	Rate	2019	55.4	79.5	34.0
DALYs	Global	Male	65-69 years	Self-harm and interpersonal violence	Alcohol use	Rate	2019	140.6	195.5	89.2
DALYs	Global	Female	65-69 years	Self-harm and interpersonal violence	Alcohol use	Rate	2019	19.0	28.8	10.4
DALYs	Global	Male	65-69 years	Substance use disorders	Alcohol use	Rate	2019	448.3	556.2	364.3
DALYs	Global	Female	65-69 years	Substance use disorders	Alcohol use	Rate	2019	98.4	126.5	77.5
DALYs	Global	Male	65-69 years	Transport injuries	Alcohol use	Rate	2019	67.3	104.9	37.9
DALYs	Global	Female	65-69 years	Transport injuries	Alcohol use	Rate	2019	6.0	9.8	3.2
DALYs	Global	Male	65-69 years	Unintentional injuries	Alcohol use	Rate	2019	146.1	252.2	67.0
DALYs	Global	Female	65-69 years	Unintentional injuries	Alcohol use	Rate	2019	37.0	66.7	16.4
DALYs	Global	Male	70-74 years	Digestive diseases	Alcohol use	Rate	2019	1076.1	1330.9	823.4
DALYs	Global	Female	70-74 years	Digestive diseases	Alcohol use	Rate	2019	328.0	427.4	236.9
DALYs	Global	Male	70-74 years	Neoplasms	Alcohol use	Rate	2019	1218.9	1400.6	1047.4
DALYs	Global	Female	70-74 years	Neoplasms	Alcohol use	Rate	2019	260.0	303.3	220.8
DALYs	Global	Male	70-74 years	Neurological disorders	Alcohol use	Rate	2019	41.7	62.4	26.8
DALYs	Global	Female	70-74 years	Neurological disorders	Alcohol use	Rate	2019	13.1	21.1	7.6

DALYs	Global	Male	70-74 years	Respiratory infections and tuberculosis	Alcohol use	Rate	2019	531.3	678.9	384.2
DALYs	Global	Female	70-74 years	Respiratory infections and tuberculosis	Alcohol use	Rate	2019	69.5	99.6	42.9
DALYs	Global	Male	70-74 years	Self-harm and interpersonal violence	Alcohol use	Rate	2019	126.5	178.6	77.5
DALYs	Global	Female	70-74 years	Self-harm and interpersonal violence	Alcohol use	Rate	2019	18.3	27.3	9.5
DALYs	Global	Male	70-74 years	Substance use disorders	Alcohol use	Rate	2019	347.8	445.8	275.6
DALYs	Global	Female	70-74 years	Substance use disorders	Alcohol use	Rate	2019	77.4	105.3	58.3
DALYs	Global	Male	70-74 years	Transport injuries	Alcohol use	Rate	2019	63.5	99.4	35.8
DALYs	Global	Female	70-74 years	Transport injuries	Alcohol use	Rate	2019	16.7	27.3	8.6
DALYs	Global	Male	70-74 years	Unintentional injuries	Alcohol use	Rate	2019	158.6	277.6	73.7
DALYs	Global	Female	70-74 years	Unintentional injuries	Alcohol use	Rate	2019	47.4	88.9	21.2
DALYs	Global	Male	75-79 years	Digestive diseases	Alcohol use	Rate	2019	917.3	1141.6	714.6
DALYs	Global	Female	75-79 years	Digestive diseases	Alcohol use	Rate	2019	297.7	389.2	219.0
DALYs	Global	Male	75-79 years	Neoplasms	Alcohol use	Rate	2019	1098.2	1263.1	948.4
DALYs	Global	Female	75-79 years	Neoplasms	Alcohol use	Rate	2019	248.5	290.8	209.1
DALYs	Global	Male	75-79 years	Neurological disorders	Alcohol use	Rate	2019	44.0	66.5	28.2
DALYs	Global	Female	75-79 years	Neurological disorders	Alcohol use	Rate	2019	13.5	22.2	7.7
DALYs	Global	Male	75-79 years	Respiratory infections and tuberculosis	Alcohol use	Rate	2019	513.1	673.0	360.0
DALYs	Global	Female	75-79 years	Respiratory infections and tuberculosis	Alcohol use	Rate	2019	67.4	102.2	38.8
DALYs	Global	Male	75-79 years	Self-harm and interpersonal violence	Alcohol use	Rate	2019	110.9	159.0	65.1
DALYs	Global	Female	75-79 years	Self-harm and interpersonal violence	Alcohol use	Rate	2019	15.1	23.1	7.8
DALYs	Global	Male	75-79 years	Substance use disorders	Alcohol use	Rate	2019	286.3	361.1	227.7
DALYs	Global	Female	75-79 years	Substance use disorders	Alcohol use	Rate	2019	74.9	99.6	56.8
DALYs	Global	Male	75-79 years	Transport injuries	Alcohol use	Rate	2019	101.1	158.2	57.3
DALYs	Global	Female	75-79 years	Transport injuries	Alcohol use	Rate	2019	17.2	28.1	8.9
DALYs	Global	Male	75-79 years	Unintentional injuries	Alcohol use	Rate	2019	175.4	304.1	79.0
DALYs	Global	Female	75-79 years	Unintentional injuries	Alcohol use	Rate	2019	58.9	112.3	25.3
DALYs	Global	Male	80-84	Digestive diseases	Alcohol use	Rate	2019	762.7	979.6	576.6
DALYs	Global	Female	80-84	Digestive diseases	Alcohol use	Rate	2019	274.8	369.6	195.1
DALYs	Global	Male	80-84	Neoplasms	Alcohol use	Rate	2019	926.8	1066.7	781.3
DALYs	Global	Female	80-84	Neoplasms	Alcohol use	Rate	2019	240.8	282.7	194.3
DALYs	Global	Male	80-84	Neurological disorders	Alcohol use	Rate	2019	44.6	67.2	28.1
DALYs	Global	Female	80-84	Neurological disorders	Alcohol use	Rate	2019	15.1	24.5	8.7
DALYs	Global	Male	80-84	Respiratory infections and tuberculosis	Alcohol use	Rate	2019	528.3	703.1	351.6
DALYs	Global	Female	80-84	Respiratory infections and tuberculosis	Alcohol use	Rate	2019	79.1	120.8	41.3
DALYs	Global	Male	80-84	Self-harm and interpersonal violence	Alcohol use	Rate	2019	93.7	137.8	50.7
DALYs	Global	Female	80-84	Self-harm and interpersonal violence	Alcohol use	Rate	2019	12.5	19.8	6.1
DALYs	Global	Male	80-84	Substance use disorders	Alcohol use	Rate	2019	218.1	286.9	168.9
DALYs	Global	Female	80-84	Substance use disorders	Alcohol use	Rate	2019	68.7	94.9	49.6
DALYs	Global	Male	80-84	Transport injuries	Alcohol use	Rate	2019	118.9	184.9	67.9
DALYs	Global	Female	80-84	Transport injuries	Alcohol use	Rate	2019	28.6	47.0	14.7
DALYs	Global	Male	80-84	Unintentional injuries	Alcohol use	Rate	2019	206.1	370.7	95.6
DALYs	Global	Female	80-84	Unintentional injuries	Alcohol use	Rate	2019	81.8	153.9	35.3
DALYs	Global	Male	85-89	Digestive diseases	Alcohol use	Rate	2019	738.8	941.2	567.1
DALYs	Global	Female	85-89	Digestive diseases	Alcohol use	Rate	2019	258.7	345.1	182.9
DALYs	Global	Male	85-89	Neoplasms	Alcohol use	Rate	2019	905.4	1052.6	751.8
DALYs	Global	Female	85-89	Neoplasms	Alcohol use	Rate	2019	265.8	317.0	205.0
DALYs	Global	Male	85-89	Neurological disorders	Alcohol use	Rate	2019	51.0	77.3	31.6
DALYs	Global	Female	85-89	Neurological disorders	Alcohol use	Rate	2019	18.4	29.9	10.6
DALYs	Global	Male	85-89	Respiratory infections and tuberculosis	Alcohol use	Rate	2019	679.2	943.8	422.0
DALYs	Global	Female	85-89	Respiratory infections and tuberculosis	Alcohol use	Rate	2019	113.4	184.6	50.2
DALYs	Global	Male	85-89	Self-harm and interpersonal violence	Alcohol use	Rate	2019	96.2	143.0	51.1

DALYs	Global	Female	85-89	Self-harm and interpersonal violence	Alcohol use	Rate	2019	12.2	19.7	5.9
DALYs	Global	Male	85-89	Substance use disorders	Alcohol use	Rate	2019	195.0	254.9	147.7
DALYs	Global	Female	85-89	Substance use disorders	Alcohol use	Rate	2019	78.7	109.0	56.8
DALYs	Global	Male	85-89	Transport injuries	Alcohol use	Rate	2019	108.9	168.7	61.4
DALYs	Global	Female	85-89	Transport injuries	Alcohol use	Rate	2019	26.5	43.8	13.8
DALYs	Global	Male	85-89	Unintentional injuries	Alcohol use	Rate	2019	294.8	526.5	136.7
DALYs	Global	Female	85-89	Unintentional injuries	Alcohol use	Rate	2019	133.3	248.7	59.2
DALYs	Global	Male	90-94	Digestive diseases	Alcohol use	Rate	2019	656.1	842.8	483.7
DALYs	Global	Female	90-94	Digestive diseases	Alcohol use	Rate	2019	266.4	368.0	177.8
DALYs	Global	Male	90-94	Neoplasms	Alcohol use	Rate	2019	721.6	849.3	574.4
DALYs	Global	Female	90-94	Neoplasms	Alcohol use	Rate	2019	297.6	363.8	217.5
DALYs	Global	Male	90-94	Neurological disorders	Alcohol use	Rate	2019	56.4	85.8	34.5
DALYs	Global	Female	90-94	Neurological disorders	Alcohol use	Rate	2019	22.1	36.1	12.9
DALYs	Global	Male	90-94	Respiratory infections and tuberculosis	Alcohol use	Rate	2019	839.9	1213.5	467.3
DALYs	Global	Female	90-94	Respiratory infections and tuberculosis	Alcohol use	Rate	2019	173.5	297.0	64.2
DALYs	Global	Male	90-94	Self-harm and interpersonal violence	Alcohol use	Rate	2019	76.3	112.2	42.5
DALYs	Global	Female	90-94	Self-harm and interpersonal violence	Alcohol use	Rate	2019	10.3	16.4	4.9
DALYs	Global	Male	90-94	Substance use disorders	Alcohol use	Rate	2019	189.9	261.9	137.6
DALYs	Global	Female	90-94	Substance use disorders	Alcohol use	Rate	2019	96.2	136.2	65.5
DALYs	Global	Male	90-94	Transport injuries	Alcohol use	Rate	2019	93.0	144.1	51.6
DALYs	Global	Female	90-94	Transport injuries	Alcohol use	Rate	2019	25.2	41.3	13.2
DALYs	Global	Male	90-94	Unintentional injuries	Alcohol use	Rate	2019	391.1	681.7	183.4
DALYs	Global	Female	90-94	Unintentional injuries	Alcohol use	Rate	2019	210.4	385.5	93.2
DALYs	Global	Male	95+ years	Digestive diseases	Alcohol use	Rate	2019	676.1	899.4	463.7
DALYs	Global	Female	95+ years	Digestive diseases	Alcohol use	Rate	2019	340.2	501.7	204.1
DALYs	Global	Male	95+ years	Neoplasms	Alcohol use	Rate	2019	594.7	717.7	451.1
DALYs	Global	Female	95+ years	Neoplasms	Alcohol use	Rate	2019	343.7	424.7	246.1
DALYs	Global	Male	95+ years	Neurological disorders	Alcohol use	Rate	2019	58.7	90.3	35.2
DALYs	Global	Female	95+ years	Neurological disorders	Alcohol use	Rate	2019	25.7	41.8	14.7
DALYs	Global	Male	95+ years	Respiratory infections and tuberculosis	Alcohol use	Rate	2019	1035.8	1559.7	515.1
DALYs	Global	Female	95+ years	Respiratory infections and tuberculosis	Alcohol use	Rate	2019	290.8	512.3	98.3
DALYs	Global	Male	95+ years	Self-harm and interpersonal violence	Alcohol use	Rate	2019	61.9	88.3	37.4
DALYs	Global	Female	95+ years	Self-harm and interpersonal violence	Alcohol use	Rate	2019	8.8	13.7	4.5
DALYs	Global	Male	95+ years	Substance use disorders	Alcohol use	Rate	2019	234.6	338.4	162.0
DALYs	Global	Female	95+ years	Substance use disorders	Alcohol use	Rate	2019	131.9	202.4	86.9
DALYs	Global	Male	95+ years	Transport injuries	Alcohol use	Rate	2019	85.8	133.9	47.4
DALYs	Global	Female	95+ years	Transport injuries	Alcohol use	Rate	2019	26.0	42.5	13.6
DALYs	Global	Male	95+ years	Unintentional injuries	Alcohol use	Rate	2019	481.0	826.2	230.4
DALYs	Global	Female	95+ years	Unintentional injuries	Alcohol use	Rate	2019	309.7	573.2	137.4

Figure 3A Trends in ASDR for MDR-TB among HIV-negative individuals caused by alcohol use, stratified by sex , 1990-2019

measure	location	sex	age	cause	risk factor	metric	year	value (per 100000)	95% UI (lower)	95% UI (upper)
DALYs	Global	Male	Age-standardized	MDR-TB	Alcohol use	Rate	1990	4.6	10.7	1.5
DALYs	Global	Female	Age-standardized	MDR-TB	Alcohol use	Rate	1990	0.5	1.1	0.2
DALYs	Global	Both	Age-standardized	MDR-TB	Alcohol use	Rate	1990	2.5	5.9	0.8
DALYs	Global	Male	Age-standardized	MDR-TB	Alcohol use	Rate	1991	7.0	15.4	2.4
DALYs	Global	Female	Age-standardized	MDR-TB	Alcohol use	Rate	1991	0.7	1.6	0.3
DALYs	Global	Both	Age-standardized	MDR-TB	Alcohol use	Rate	1991	3.8	8.2	1.3
DALYs	Global	Male	Age-standardized	MDR-TB	Alcohol use	Rate	1992	9.6	19.0	4.0
DALYs	Global	Female	Age-standardized	MDR-TB	Alcohol use	Rate	1992	1.0	2.0	0.4
DALYs	Global	Both	Age-standardized	MDR-TB	Alcohol use	Rate	1992	5.2	10.2	2.1
DALYs	Global	Male	Age-standardized	MDR-TB	Alcohol use	Rate	1993	12.5	23.4	5.7
DALYs	Global	Female	Age-standardized	MDR-TB	Alcohol use	Rate	1993	1.3	2.4	0.5
DALYs	Global	Both	Age-standardized	MDR-TB	Alcohol use	Rate	1993	6.7	12.6	3.0
DALYs	Global	Male	Age-standardized	MDR-TB	Alcohol use	Rate	1994	15.6	28.8	7.2
DALYs	Global	Female	Age-standardized	MDR-TB	Alcohol use	Rate	1994	1.6	3.0	0.6
DALYs	Global	Both	Age-standardized	MDR-TB	Alcohol use	Rate	1994	8.4	15.7	3.9
DALYs	Global	Male	Age-standardized	MDR-TB	Alcohol use	Rate	1995	18.2	33.5	8.7
DALYs	Global	Female	Age-standardized	MDR-TB	Alcohol use	Rate	1995	1.8	3.4	0.8
DALYs	Global	Both	Age-standardized	MDR-TB	Alcohol use	Rate	1995	9.9	18.3	4.7
DALYs	Global	Male	Age-standardized	MDR-TB	Alcohol use	Rate	1996	20.0	37.3	9.5
DALYs	Global	Female	Age-standardized	MDR-TB	Alcohol use	Rate	1996	2.0	3.7	0.9
DALYs	Global	Both	Age-standardized	MDR-TB	Alcohol use	Rate	1996	10.8	20.0	5.1
DALYs	Global	Male	Age-standardized	MDR-TB	Alcohol use	Rate	1997	21.0	38.5	10.1
DALYs	Global	Female	Age-standardized	MDR-TB	Alcohol use	Rate	1997	2.1	4.0	0.9
DALYs	Global	Both	Age-standardized	MDR-TB	Alcohol use	Rate	1997	11.4	20.8	5.5
DALYs	Global	Male	Age-standardized	MDR-TB	Alcohol use	Rate	1998	22.1	40.4	10.6
DALYs	Global	Female	Age-standardized	MDR-TB	Alcohol use	Rate	1998	2.3	4.2	1.0
DALYs	Global	Both	Age-standardized	MDR-TB	Alcohol use	Rate	1998	12.0	21.9	5.7
DALYs	Global	Male	Age-standardized	MDR-TB	Alcohol use	Rate	1999	23.8	42.8	11.4
DALYs	Global	Female	Age-standardized	MDR-TB	Alcohol use	Rate	1999	2.4	4.5	1.1
DALYs	Global	Both	Age-standardized	MDR-TB	Alcohol use	Rate	1999	12.9	23.0	6.2
DALYs	Global	Male	Age-standardized	MDR-TB	Alcohol use	Rate	2000	25.2	44.2	12.1
DALYs	Global	Female	Age-standardized	MDR-TB	Alcohol use	Rate	2000	2.6	4.7	1.2
DALYs	Global	Both	Age-standardized	MDR-TB	Alcohol use	Rate	2000	13.7	24.2	6.6
DALYs	Global	Male	Age-standardized	MDR-TB	Alcohol use	Rate	2001	25.7	44.5	12.7
DALYs	Global	Female	Age-standardized	MDR-TB	Alcohol use	Rate	2001	2.6	4.9	1.2
DALYs	Global	Both	Age-standardized	MDR-TB	Alcohol use	Rate	2001	14.0	24.3	6.9
DALYs	Global	Male	Age-standardized	MDR-TB	Alcohol use	Rate	2002	26.1	44.9	12.9
DALYs	Global	Female	Age-standardized	MDR-TB	Alcohol use	Rate	2002	2.7	5.0	1.3
DALYs	Global	Both	Age-standardized	MDR-TB	Alcohol use	Rate	2002	14.3	24.4	7.1
DALYs	Global	Male	Age-standardized	MDR-TB	Alcohol use	Rate	2003	26.3	44.6	13.3
DALYs	Global	Female	Age-standardized	MDR-TB	Alcohol use	Rate	2003	2.8	5.1	1.3
DALYs	Global	Both	Age-standardized	MDR-TB	Alcohol use	Rate	2003	14.4	24.6	7.2
DALYs	Global	Male	Age-standardized	MDR-TB	Alcohol use	Rate	2004	25.8	44.2	13.1
DALYs	Global	Female	Age-standardized	MDR-TB	Alcohol use	Rate	2004	2.8	5.0	1.3
DALYs	Global	Both	Age-standardized	MDR-TB	Alcohol use	Rate	2004	14.2	24.5	7.2
DALYs	Global	Male	Age-standardized	MDR-TB	Alcohol use	Rate	2005	25.9	44.4	13.2
DALYs	Global	Female	Age-standardized	MDR-TB	Alcohol use	Rate	2005	2.8	5.0	1.3
DALYs	Global	Both	Age-standardized	MDR-TB	Alcohol use	Rate	2005	14.2	24.4	7.2
DALYs	Global	Male	Age-standardized	MDR-TB	Alcohol use	Rate	2006	24.7	42.0	12.5

DALYs	Global	Female	Age-standardized	MDR-TB	Alcohol use	Rate	2006	2.7	4.8	1.3
DALYs	Global	Both	Age-standardized	MDR-TB	Alcohol use	Rate	2006	13.5	23.0	6.8
DALYs	Global	Male	Age-standardized	MDR-TB	Alcohol use	Rate	2007	23.8	40.8	11.7
DALYs	Global	Female	Age-standardized	MDR-TB	Alcohol use	Rate	2007	2.6	4.6	1.2
DALYs	Global	Both	Age-standardized	MDR-TB	Alcohol use	Rate	2007	13.0	22.5	6.5
DALYs	Global	Male	Age-standardized	MDR-TB	Alcohol use	Rate	2008	23.1	39.5	11.3
DALYs	Global	Female	Age-standardized	MDR-TB	Alcohol use	Rate	2008	2.5	4.4	1.2
DALYs	Global	Both	Age-standardized	MDR-TB	Alcohol use	Rate	2008	12.7	21.7	6.2
DALYs	Global	Male	Age-standardized	MDR-TB	Alcohol use	Rate	2009	21.9	37.7	10.7
DALYs	Global	Female	Age-standardized	MDR-TB	Alcohol use	Rate	2009	2.3	4.1	1.1
DALYs	Global	Both	Age-standardized	MDR-TB	Alcohol use	Rate	2009	12.0	20.7	5.9
DALYs	Global	Male	Age-standardized	MDR-TB	Alcohol use	Rate	2010	21.0	36.5	9.8
DALYs	Global	Female	Age-standardized	MDR-TB	Alcohol use	Rate	2010	2.2	4.0	1.0
DALYs	Global	Both	Age-standardized	MDR-TB	Alcohol use	Rate	2010	11.5	20.1	5.4
DALYs	Global	Male	Age-standardized	MDR-TB	Alcohol use	Rate	2011	20.1	36.1	9.2
DALYs	Global	Female	Age-standardized	MDR-TB	Alcohol use	Rate	2011	2.1	3.8	1.0
DALYs	Global	Both	Age-standardized	MDR-TB	Alcohol use	Rate	2011	11.0	19.7	5.1
DALYs	Global	Male	Age-standardized	MDR-TB	Alcohol use	Rate	2012	19.8	36.0	9.1
DALYs	Global	Female	Age-standardized	MDR-TB	Alcohol use	Rate	2012	2.0	3.7	0.9
DALYs	Global	Both	Age-standardized	MDR-TB	Alcohol use	Rate	2012	10.8	19.5	5.0
DALYs	Global	Male	Age-standardized	MDR-TB	Alcohol use	Rate	2013	19.3	36.4	8.7
DALYs	Global	Female	Age-standardized	MDR-TB	Alcohol use	Rate	2013	1.9	3.5	0.9
DALYs	Global	Both	Age-standardized	MDR-TB	Alcohol use	Rate	2013	10.5	19.4	4.8
DALYs	Global	Male	Age-standardized	MDR-TB	Alcohol use	Rate	2014	18.6	35.3	8.3
DALYs	Global	Female	Age-standardized	MDR-TB	Alcohol use	Rate	2014	1.9	3.5	0.9
DALYs	Global	Both	Age-standardized	MDR-TB	Alcohol use	Rate	2014	10.1	19.0	4.6
DALYs	Global	Male	Age-standardized	MDR-TB	Alcohol use	Rate	2015	18.0	33.7	7.9
DALYs	Global	Female	Age-standardized	MDR-TB	Alcohol use	Rate	2015	1.9	3.4	0.8
DALYs	Global	Both	Age-standardized	MDR-TB	Alcohol use	Rate	2015	9.8	18.4	4.4
DALYs	Global	Male	Age-standardized	MDR-TB	Alcohol use	Rate	2016	17.7	34.6	7.5
DALYs	Global	Female	Age-standardized	MDR-TB	Alcohol use	Rate	2016	1.8	3.3	0.8
DALYs	Global	Both	Age-standardized	MDR-TB	Alcohol use	Rate	2016	9.7	18.7	4.2
DALYs	Global	Male	Age-standardized	MDR-TB	Alcohol use	Rate	2017	17.4	34.2	7.2
DALYs	Global	Female	Age-standardized	MDR-TB	Alcohol use	Rate	2017	1.8	3.3	0.8
DALYs	Global	Both	Age-standardized	MDR-TB	Alcohol use	Rate	2017	9.5	18.5	4.0
DALYs	Global	Male	Age-standardized	MDR-TB	Alcohol use	Rate	2018	17.0	33.3	7.2
DALYs	Global	Female	Age-standardized	MDR-TB	Alcohol use	Rate	2018	1.7	3.3	0.8
DALYs	Global	Both	Age-standardized	MDR-TB	Alcohol use	Rate	2018	9.3	18.0	4.0
DALYs	Global	Male	Age-standardized	MDR-TB	Alcohol use	Rate	2019	16.6	33.2	6.9
DALYs	Global	Female	Age-standardized	MDR-TB	Alcohol use	Rate	2019	1.7	3.3	0.8
DALYs	Global	Both	Age-standardized	MDR-TB	Alcohol use	Rate	2019	9.0	18.1	3.8

DALYs: disability-sdjusted life years; ASDR: age-standardized DALYs rate

Figure 3B Trends in ASDR for MDR-TB among HIV-negative individuals caused by alcohol use, stratified by SDI, 1990-2019

measure	location	sex	age	cause	risk factor	metric	year	value (per 100000)	95% UI (lower)	95% UI (upper)
DALYs	High-middle SDI	Both	Age-standardized	MDR-TB	Alcohol use	Rate	1990	2.6	6.8	0.8
DALYs	Low SDI	Both	Age-standardized	MDR-TB	Alcohol use	Rate	1990	2.1	4.9	0.7
DALYs	Middle SDI	Both	Age-standardized	MDR-TB	Alcohol use	Rate	1990	4.0	10.9	1.1
DALYs	Low-middle SDI	Both	Age-standardized	MDR-TB	Alcohol use	Rate	1990	2.2	7.0	0.5
DALYs	High SDI	Both	Age-standardized	MDR-TB	Alcohol use	Rate	1990	0.7	1.4	0.3
DALYs	Low-middle SDI	Both	Age-standardized	MDR-TB	Alcohol use	Rate	1991	3.5	9.7	0.9
DALYs	Middle SDI	Both	Age-standardized	MDR-TB	Alcohol use	Rate	1991	6.0	14.2	1.8
DALYs	High-middle SDI	Both	Age-standardized	MDR-TB	Alcohol use	Rate	1991	4.1	9.7	1.4
DALYs	High SDI	Both	Age-standardized	MDR-TB	Alcohol use	Rate	1991	0.9	1.7	0.3
DALYs	Low SDI	Both	Age-standardized	MDR-TB	Alcohol use	Rate	1991	3.5	8.1	1.1
DALYs	High SDI	Both	Age-standardized	MDR-TB	Alcohol use	Rate	1992	1.0	1.9	0.4
DALYs	Low SDI	Both	Age-standardized	MDR-TB	Alcohol use	Rate	1992	5.5	12.8	1.9
DALYs	High-middle SDI	Both	Age-standardized	MDR-TB	Alcohol use	Rate	1992	6.1	12.8	2.4
DALYs	Low-middle SDI	Both	Age-standardized	MDR-TB	Alcohol use	Rate	1992	5.1	13.4	1.4
DALYs	Middle SDI	Both	Age-standardized	MDR-TB	Alcohol use	Rate	1992	7.8	16.5	2.8
DALYs	Low-middle SDI	Both	Age-standardized	MDR-TB	Alcohol use	Rate	1993	6.9	17.4	2.0
DALYs	Middle SDI	Both	Age-standardized	MDR-TB	Alcohol use	Rate	1993	9.0	17.9	3.7
DALYs	High SDI	Both	Age-standardized	MDR-TB	Alcohol use	Rate	1993	1.2	2.1	0.5
DALYs	Low SDI	Both	Age-standardized	MDR-TB	Alcohol use	Rate	1993	8.3	19.1	2.8
DALYs	High-middle SDI	Both	Age-standardized	MDR-TB	Alcohol use	Rate	1993	8.6	16.7	3.8
DALYs	High SDI	Both	Age-standardized	MDR-TB	Alcohol use	Rate	1994	1.3	2.3	0.6
DALYs	Low SDI	Both	Age-standardized	MDR-TB	Alcohol use	Rate	1994	11.9	27.5	4.1
DALYs	Low-middle SDI	Both	Age-standardized	MDR-TB	Alcohol use	Rate	1994	8.9	23.0	2.7
DALYs	High-middle SDI	Both	Age-standardized	MDR-TB	Alcohol use	Rate	1994	11.8	21.5	5.8
DALYs	Middle SDI	Both	Age-standardized	MDR-TB	Alcohol use	Rate	1994	10.0	18.4	4.4
DALYs	Middle SDI	Both	Age-standardized	MDR-TB	Alcohol use	Rate	1995	10.7	18.8	4.9
DALYs	Low-middle SDI	Both	Age-standardized	MDR-TB	Alcohol use	Rate	1995	10.9	29.1	3.5
DALYs	High-middle SDI	Both	Age-standardized	MDR-TB	Alcohol use	Rate	1995	14.2	24.5	7.1
DALYs	High SDI	Both	Age-standardized	MDR-TB	Alcohol use	Rate	1995	1.3	2.3	0.6
DALYs	Low SDI	Both	Age-standardized	MDR-TB	Alcohol use	Rate	1995	16.1	36.7	5.4
DALYs	High SDI	Both	Age-standardized	MDR-TB	Alcohol use	Rate	1996	1.2	2.2	0.6
DALYs	Low SDI	Both	Age-standardized	MDR-TB	Alcohol use	Rate	1996	20.2	46.2	6.8
DALYs	High-middle SDI	Both	Age-standardized	MDR-TB	Alcohol use	Rate	1996	15.2	25.5	7.8
DALYs	Middle SDI	Both	Age-standardized	MDR-TB	Alcohol use	Rate	1996	11.1	19.8	5.1
DALYs	Low-middle SDI	Both	Age-standardized	MDR-TB	Alcohol use	Rate	1996	12.6	32.3	4.1
DALYs	Low-middle SDI	Both	Age-standardized	MDR-TB	Alcohol use	Rate	1997	14.1	36.1	4.7
DALYs	Middle SDI	Both	Age-standardized	MDR-TB	Alcohol use	Rate	1997	11.0	19.7	5.0
DALYs	High SDI	Both	Age-standardized	MDR-TB	Alcohol use	Rate	1997	1.2	2.1	0.6
DALYs	Low SDI	Both	Age-standardized	MDR-TB	Alcohol use	Rate	1997	23.0	52.4	8.1
DALYs	High-middle SDI	Both	Age-standardized	MDR-TB	Alcohol use	Rate	1997	15.7	26.3	8.1
DALYs	High SDI	Both	Age-standardized	MDR-TB	Alcohol use	Rate	1998	1.1	2.0	0.5
DALYs	Low SDI	Both	Age-standardized	MDR-TB	Alcohol use	Rate	1998	25.9	56.2	9.5
DALYs	Middle SDI	Both	Age-standardized	MDR-TB	Alcohol use	Rate	1998	11.0	20.0	5.1

DALYs	High-middle SDI	Both	Age-standardized	MDR-TB	Alcohol use	Rate	1998	16.2	26.6	8.6
DALYs	Low-middle SDI	Both	Age-standardized	MDR-TB	Alcohol use	Rate	1998	15.6	39.0	5.5
DALYs	High-middle SDI	Both	Age-standardized	MDR-TB	Alcohol use	Rate	1999	18.1	29.5	9.5
DALYs	Low SDI	Both	Age-standardized	MDR-TB	Alcohol use	Rate	1999	27.9	60.3	10.5
DALYs	Low-middle SDI	Both	Age-standardized	MDR-TB	Alcohol use	Rate	1999	17.2	41.7	6.1
DALYs	Middle SDI	Both	Age-standardized	MDR-TB	Alcohol use	Rate	1999	11.2	20.4	5.1
DALYs	High SDI	Both	Age-standardized	MDR-TB	Alcohol use	Rate	1999	1.1	1.9	0.5
DALYs	High SDI	Both	Age-standardized	MDR-TB	Alcohol use	Rate	2000	1.0	1.9	0.5
DALYs	Low SDI	Both	Age-standardized	MDR-TB	Alcohol use	Rate	2000	28.4	59.3	10.7
DALYs	Low-middle SDI	Both	Age-standardized	MDR-TB	Alcohol use	Rate	2000	18.3	41.5	6.8
DALYs	High-middle SDI	Both	Age-standardized	MDR-TB	Alcohol use	Rate	2000	19.7	31.1	10.3
DALYs	Middle SDI	Both	Age-standardized	MDR-TB	Alcohol use	Rate	2000	11.6	20.7	5.3
DALYs	Low SDI	Both	Age-standardized	MDR-TB	Alcohol use	Rate	2001	28.1	57.8	10.8
DALYs	High-middle SDI	Both	Age-standardized	MDR-TB	Alcohol use	Rate	2001	20.1	31.4	10.7
DALYs	Middle SDI	Both	Age-standardized	MDR-TB	Alcohol use	Rate	2001	11.7	20.6	5.3
DALYs	Low-middle SDI	Both	Age-standardized	MDR-TB	Alcohol use	Rate	2001	19.2	40.2	7.4
DALYs	High SDI	Both	Age-standardized	MDR-TB	Alcohol use	Rate	2001	1.0	1.8	0.5
DALYs	Low SDI	Both	Age-standardized	MDR-TB	Alcohol use	Rate	2002	27.7	56.1	10.4
DALYs	Middle SDI	Both	Age-standardized	MDR-TB	Alcohol use	Rate	2002	11.9	21.0	5.4
DALYs	High SDI	Both	Age-standardized	MDR-TB	Alcohol use	Rate	2002	0.9	1.7	0.5
DALYs	High-middle SDI	Both	Age-standardized	MDR-TB	Alcohol use	Rate	2002	20.7	32.9	11.0
DALYs	Low-middle SDI	Both	Age-standardized	MDR-TB	Alcohol use	Rate	2002	19.8	39.5	8.0
DALYs	High-middle SDI	Both	Age-standardized	MDR-TB	Alcohol use	Rate	2003	21.2	33.7	11.0
DALYs	Low SDI	Both	Age-standardized	MDR-TB	Alcohol use	Rate	2003	27.3	55.1	10.7
DALYs	Middle SDI	Both	Age-standardized	MDR-TB	Alcohol use	Rate	2003	11.8	20.7	5.3
DALYs	Low-middle SDI	Both	Age-standardized	MDR-TB	Alcohol use	Rate	2003	19.9	37.8	8.4
DALYs	High SDI	Both	Age-standardized	MDR-TB	Alcohol use	Rate	2003	0.9	1.6	0.5
DALYs	High SDI	Both	Age-standardized	MDR-TB	Alcohol use	Rate	2004	0.8	1.4	0.4
DALYs	Low SDI	Both	Age-standardized	MDR-TB	Alcohol use	Rate	2004	26.6	54.1	10.9
DALYs	Middle SDI	Both	Age-standardized	MDR-TB	Alcohol use	Rate	2004	11.4	20.5	5.2
DALYs	Low-middle SDI	Both	Age-standardized	MDR-TB	Alcohol use	Rate	2004	19.7	37.2	8.4
DALYs	High-middle SDI	Both	Age-standardized	MDR-TB	Alcohol use	Rate	2004	21.1	33.7	10.8
DALYs	Low SDI	Both	Age-standardized	MDR-TB	Alcohol use	Rate	2005	25.8	52.2	10.4
DALYs	High-middle SDI	Both	Age-standardized	MDR-TB	Alcohol use	Rate	2005	21.7	35.2	11.2
DALYs	Middle SDI	Both	Age-standardized	MDR-TB	Alcohol use	Rate	2005	11.1	19.9	4.9
DALYs	High SDI	Both	Age-standardized	MDR-TB	Alcohol use	Rate	2005	0.8	1.3	0.4
DALYs	Low-middle SDI	Both	Age-standardized	MDR-TB	Alcohol use	Rate	2005	20.0	36.3	8.8
DALYs	High SDI	Both	Age-standardized	MDR-TB	Alcohol use	Rate	2006	0.7	1.2	0.3
DALYs	Low SDI	Both	Age-standardized	MDR-TB	Alcohol use	Rate	2006	25.1	49.6	10.2
DALYs	Middle SDI	Both	Age-standardized	MDR-TB	Alcohol use	Rate	2006	10.5	18.5	4.6
DALYs	Low-middle SDI	Both	Age-standardized	MDR-TB	Alcohol use	Rate	2006	20.3	36.8	9.2
DALYs	High-middle SDI	Both	Age-standardized	MDR-TB	Alcohol use	Rate	2006	19.5	31.1	10.1
DALYs	Low SDI	Both	Age-standardized	MDR-TB	Alcohol use	Rate	2007	24.9	49.4	10.4
DALYs	High-middle SDI	Both	Age-standardized	MDR-TB	Alcohol use	Rate	2007	17.9	28.3	9.2
DALYs	Middle SDI	Both	Age-standardized	MDR-TB	Alcohol use	Rate	2007	10.1	17.9	4.6
DALYs	Low-middle SDI	Both	Age-standardized	MDR-TB	Alcohol use	Rate	2007	20.4	38.0	8.9
DALYs	High SDI	Both	Age-standardized	MDR-TB	Alcohol use	Rate	2007	0.6	1.1	0.3
DALYs	Middle SDI	Both	Age-standardized	MDR-TB	Alcohol use	Rate	2008	9.7	17.6	4.2

DALYs	Low-middle SDI	Both	Age-standardized	MDR-TB	Alcohol use	Rate	2008	20.8	40.1	8.8
DALYs	High SDI	Both	Age-standardized	MDR-TB	Alcohol use	Rate	2008	0.5	0.9	0.3
DALYs	Low SDI	Both	Age-standardized	MDR-TB	Alcohol use	Rate	2008	24.2	46.4	9.8
DALYs	High-middle SDI	Both	Age-standardized	MDR-TB	Alcohol use	Rate	2008	16.6	26.0	8.5
DALYs	Low-middle SDI	Both	Age-standardized	MDR-TB	Alcohol use	Rate	2009	20.7	40.7	8.3
DALYs	Low SDI	Both	Age-standardized	MDR-TB	Alcohol use	Rate	2009	24.0	45.8	9.3
DALYs	High SDI	Both	Age-standardized	MDR-TB	Alcohol use	Rate	2009	0.4	0.7	0.2
DALYs	Middle SDI	Both	Age-standardized	MDR-TB	Alcohol use	Rate	2009	9.3	16.6	4.1
DALYs	High-middle SDI	Both	Age-standardized	MDR-TB	Alcohol use	Rate	2009	14.4	22.7	7.5
DALYs	Middle SDI	Both	Age-standardized	MDR-TB	Alcohol use	Rate	2010	8.7	15.5	3.9
DALYs	Low-middle SDI	Both	Age-standardized	MDR-TB	Alcohol use	Rate	2010	20.5	42.5	7.8
DALYs	High SDI	Both	Age-standardized	MDR-TB	Alcohol use	Rate	2010	0.4	0.7	0.2
DALYs	Low SDI	Both	Age-standardized	MDR-TB	Alcohol use	Rate	2010	23.5	46.0	9.4
DALYs	High-middle SDI	Both	Age-standardized	MDR-TB	Alcohol use	Rate	2010	13.3	20.7	6.9
DALYs	High-middle SDI	Both	Age-standardized	MDR-TB	Alcohol use	Rate	2011	11.8	18.4	6.2
DALYs	Low SDI	Both	Age-standardized	MDR-TB	Alcohol use	Rate	2011	23.2	45.0	9.5
DALYs	Middle SDI	Both	Age-standardized	MDR-TB	Alcohol use	Rate	2011	8.2	14.3	3.6
DALYs	Low-middle SDI	Both	Age-standardized	MDR-TB	Alcohol use	Rate	2011	20.4	43.9	7.5
DALYs	High SDI	Both	Age-standardized	MDR-TB	Alcohol use	Rate	2011	0.3	0.6	0.2
DALYs	High-middle SDI	Both	Age-standardized	MDR-TB	Alcohol use	Rate	2012	10.7	17.0	5.7
DALYs	Low-middle SDI	Both	Age-standardized	MDR-TB	Alcohol use	Rate	2012	21.0	44.9	7.7
DALYs	Middle SDI	Both	Age-standardized	MDR-TB	Alcohol use	Rate	2012	7.9	14.1	3.5
DALYs	High SDI	Both	Age-standardized	MDR-TB	Alcohol use	Rate	2012	0.3	0.6	0.2
DALYs	Low SDI	Both	Age-standardized	MDR-TB	Alcohol use	Rate	2012	23.5	47.3	9.5
DALYs	Low SDI	Both	Age-standardized	MDR-TB	Alcohol use	Rate	2013	24.0	48.5	9.8
DALYs	Middle SDI	Both	Age-standardized	MDR-TB	Alcohol use	Rate	2013	7.3	13.6	3.2
DALYs	Low-middle SDI	Both	Age-standardized	MDR-TB	Alcohol use	Rate	2013	21.3	47.5	7.2
DALYs	High SDI	Both	Age-standardized	MDR-TB	Alcohol use	Rate	2013	0.3	0.6	0.2
DALYs	High-middle SDI	Both	Age-standardized	MDR-TB	Alcohol use	Rate	2013	9.7	15.4	5.2
DALYs	Middle SDI	Both	Age-standardized	MDR-TB	Alcohol use	Rate	2014	6.9	13.0	3.0
DALYs	Low-middle SDI	Both	Age-standardized	MDR-TB	Alcohol use	Rate	2014	21.1	48.9	6.8
DALYs	High SDI	Both	Age-standardized	MDR-TB	Alcohol use	Rate	2014	0.3	0.5	0.1
DALYs	High-middle SDI	Both	Age-standardized	MDR-TB	Alcohol use	Rate	2014	8.9	13.9	4.8
DALYs	Low SDI	Both	Age-standardized	MDR-TB	Alcohol use	Rate	2014	24.1	48.3	9.8
DALYs	Low SDI	Both	Age-standardized	MDR-TB	Alcohol use	Rate	2015	24.3	48.7	9.8
DALYs	High-middle SDI	Both	Age-standardized	MDR-TB	Alcohol use	Rate	2015	8.4	13.1	4.7
DALYs	Middle SDI	Both	Age-standardized	MDR-TB	Alcohol use	Rate	2015	6.4	11.8	2.8
DALYs	Low-middle SDI	Both	Age-standardized	MDR-TB	Alcohol use	Rate	2015	20.4	47.1	6.6
DALYs	High SDI	Both	Age-standardized	MDR-TB	Alcohol use	Rate	2015	0.3	0.5	0.1
DALYs	High-middle SDI	Both	Age-standardized	MDR-TB	Alcohol use	Rate	2016	7.6	12.1	4.3
DALYs	Middle SDI	Both	Age-standardized	MDR-TB	Alcohol use	Rate	2016	6.3	11.6	2.7
DALYs	Low-middle SDI	Both	Age-standardized	MDR-TB	Alcohol use	Rate	2016	20.5	48.4	6.5
DALYs	High SDI	Both	Age-standardized	MDR-TB	Alcohol use	Rate	2016	0.3	0.5	0.1
DALYs	Low SDI	Both	Age-standardized	MDR-TB	Alcohol use	Rate	2016	24.0	48.4	9.6
DALYs	High SDI	Both	Age-standardized	MDR-TB	Alcohol use	Rate	2017	0.2	0.5	0.1
DALYs	Low SDI	Both	Age-standardized	MDR-TB	Alcohol use	Rate	2017	23.9	47.2	9.5
DALYs	Middle SDI	Both	Age-standardized	MDR-TB	Alcohol use	Rate	2017	6.2	11.6	2.6
DALYs	Low-middle SDI	Both	Age-standardized	MDR-TB	Alcohol use	Rate	2017	20.5	49.3	6.1

DALYs	High-middle SDI	Both	Age-standardized	MDR-TB	Alcohol use	Rate	2017	6.9	11.1	3.8
DALYs	Low-middle SDI	Both	Age-standardized	MDR-TB	Alcohol use	Rate	2018	19.8	46.9	5.8
DALYs	High SDI	Both	Age-standardized	MDR-TB	Alcohol use	Rate	2018	0.2	0.5	0.1
DALYs	Middle SDI	Both	Age-standardized	MDR-TB	Alcohol use	Rate	2018	6.0	11.2	2.5
DALYs	Low SDI	Both	Age-standardized	MDR-TB	Alcohol use	Rate	2018	23.5	46.7	9.5
DALYs	High-middle SDI	Both	Age-standardized	MDR-TB	Alcohol use	Rate	2018	6.7	10.7	3.7
DALYs	High SDI	Both	Age-standardized	MDR-TB	Alcohol use	Rate	2019	0.2	0.4	0.1
DALYs	Low SDI	Both	Age-standardized	MDR-TB	Alcohol use	Rate	2019	23.1	45.9	9.3
DALYs	Middle SDI	Both	Age-standardized	MDR-TB	Alcohol use	Rate	2019	5.8	11.1	2.4
DALYs	High-middle SDI	Both	Age-standardized	MDR-TB	Alcohol use	Rate	2019	6.4	10.4	3.6
DALYs	Low-middle SDI	Both	Age-standardized	MDR-TB	Alcohol use	Rate	2019	19.1	45.0	5.6

DALYs: disability-sdjusted life years; ASDR: age-standardized DALYs rate

Figure 4A Age effect on the relative risk of MDR-TB among HIV-free individuals, grouped by SDI

label	Age (years)	location	sex	cause	measure	Rate	95%CI (lower)	95%CI (upper)
0 to 4	2.5	Global	Male	MDR-TB	Incidence	0.5	0.4	0.5
5 to 9	7.5	Global	Male	MDR-TB	Incidence	0.3	0.3	0.4
10 to 14	12.5	Global	Male	MDR-TB	Incidence	0.5	0.5	0.6
15 to 19	17.5	Global	Male	MDR-TB	Incidence	1.7	1.5	1.8
20 to 24	22.5	Global	Male	MDR-TB	Incidence	2.6	2.5	2.8
25 to 29	27.5	Global	Male	MDR-TB	Incidence	3.5	3.3	3.7
30 to 34	32.5	Global	Male	MDR-TB	Incidence	4.8	4.5	5.1
35 to 39	37.5	Global	Male	MDR-TB	Incidence	5.9	5.6	6.2
40 to 44	42.5	Global	Male	MDR-TB	Incidence	7.7	7.3	8.0
45 to 49	47.5	Global	Male	MDR-TB	Incidence	8.9	8.5	9.3
50 to 54	52.5	Global	Male	MDR-TB	Incidence	10.9	10.3	11.4
55 to 59	57.5	Global	Male	MDR-TB	Incidence	14.4	13.6	15.1
60 to 64	62.5	Global	Male	MDR-TB	Incidence	16.3	15.3	17.4
65 to 69	67.5	Global	Male	MDR-TB	Incidence	18.4	17.1	19.7
70 to 74	72.5	Global	Male	MDR-TB	Incidence	16.8	15.4	18.3
75 to 79	77.5	Global	Male	MDR-TB	Incidence	16.0	14.5	17.7
80 to 84	82.5	Global	Male	MDR-TB	Incidence	19.8	17.6	22.4
85 to 89	87.5	Global	Male	MDR-TB	Incidence	21.3	18.1	25.2
90 to 94	92.5	Global	Male	MDR-TB	Incidence	20.2	15.1	26.9
95 plus	97.5	Global	Male	MDR-TB	Incidence	17.2	9.3	31.9
0 to 4	2.5	High SDI	Male	MDR-TB	Incidence	0.2	0.2	0.2
5 to 9	7.5	High SDI	Male	MDR-TB	Incidence	0.1	0.1	0.2
10 to 14	12.5	High SDI	Male	MDR-TB	Incidence	0.2	0.1	0.2
15 to 19	17.5	High SDI	Male	MDR-TB	Incidence	0.5	0.4	0.5
20 to 24	22.5	High SDI	Male	MDR-TB	Incidence	0.7	0.6	0.8
25 to 29	27.5	High SDI	Male	MDR-TB	Incidence	0.6	0.6	0.7
30 to 34	32.5	High SDI	Male	MDR-TB	Incidence	0.7	0.6	0.7
35 to 39	37.5	High SDI	Male	MDR-TB	Incidence	0.6	0.5	0.6
40 to 44	42.5	High SDI	Male	MDR-TB	Incidence	0.6	0.5	0.6
45 to 49	47.5	High SDI	Male	MDR-TB	Incidence	0.5	0.4	0.5
50 to 54	52.5	High SDI	Male	MDR-TB	Incidence	0.4	0.4	0.5
55 to 59	57.5	High SDI	Male	MDR-TB	Incidence	0.4	0.4	0.5
60 to 64	62.5	High SDI	Male	MDR-TB	Incidence	0.4	0.4	0.4
65 to 69	67.5	High SDI	Male	MDR-TB	Incidence	0.4	0.4	0.5
70 to 74	72.5	High SDI	Male	MDR-TB	Incidence	0.4	0.3	0.4
75 to 79	77.5	High SDI	Male	MDR-TB	Incidence	0.3	0.3	0.3
80 to 84	82.5	High SDI	Male	MDR-TB	Incidence	0.3	0.3	0.4
85 to 89	87.5	High SDI	Male	MDR-TB	Incidence	0.3	0.2	0.3
90 to 94	92.5	High SDI	Male	MDR-TB	Incidence	0.2	0.2	0.3
95 plus	97.5	High SDI	Male	MDR-TB	Incidence	0.2	0.1	0.3
0 to 4	2.5	High-middle SDI	Male	MDR-TB	Incidence	0.9	0.8	1.1
5 to 9	7.5	High-middle SDI	Male	MDR-TB	Incidence	0.6	0.5	0.7
10 to 14	12.5	High-middle SDI	Male	MDR-TB	Incidence	0.8	0.7	0.9
15 to 19	17.5	High-middle SDI	Male	MDR-TB	Incidence	2.7	2.4	3.0
20 to 24	22.5	High-middle SDI	Male	MDR-TB	Incidence	4.7	4.3	5.1

25 to 29	27.5	High-middle SDI	Male	MDR-TB	Incidence	6.3	5.8	6.8
30 to 34	32.5	High-middle SDI	Male	MDR-TB	Incidence	8.8	8.2	9.4
35 to 39	37.5	High-middle SDI	Male	MDR-TB	Incidence	10.6	10.0	11.2
40 to 44	42.5	High-middle SDI	Male	MDR-TB	Incidence	12.8	12.2	13.5
45 to 49	47.5	High-middle SDI	Male	MDR-TB	Incidence	13.3	12.7	14.1
50 to 54	52.5	High-middle SDI	Male	MDR-TB	Incidence	14.4	13.6	15.2
55 to 59	57.5	High-middle SDI	Male	MDR-TB	Incidence	16.9	15.8	18.0
60 to 64	62.5	High-middle SDI	Male	MDR-TB	Incidence	16.3	15.1	17.7
65 to 69	67.5	High-middle SDI	Male	MDR-TB	Incidence	15.4	14.0	16.9
70 to 74	72.5	High-middle SDI	Male	MDR-TB	Incidence	13.1	11.6	14.7
75 to 79	77.5	High-middle SDI	Male	MDR-TB	Incidence	12.0	10.4	13.7
80 to 84	82.5	High-middle SDI	Male	MDR-TB	Incidence	13.4	11.3	15.8
85 to 89	87.5	High-middle SDI	Male	MDR-TB	Incidence	13.1	10.4	16.6
90 to 94	92.5	High-middle SDI	Male	MDR-TB	Incidence	11.0	7.1	17.0
95 plus	97.5	High-middle SDI	Male	MDR-TB	Incidence	8.8	3.2	24.3
0 to 4	2.5	Low SDI	Male	MDR-TB	Incidence	0.2	0.2	0.2
5 to 9	7.5	Low SDI	Male	MDR-TB	Incidence	0.1	0.1	0.2
10 to 14	12.5	Low SDI	Male	MDR-TB	Incidence	0.2	0.2	0.3
15 to 19	17.5	Low SDI	Male	MDR-TB	Incidence	0.9	0.8	1.0
20 to 24	22.5	Low SDI	Male	MDR-TB	Incidence	1.6	1.5	1.7
25 to 29	27.5	Low SDI	Male	MDR-TB	Incidence	2.4	2.2	2.5
30 to 34	32.5	Low SDI	Male	MDR-TB	Incidence	3.6	3.3	3.8
35 to 39	37.5	Low SDI	Male	MDR-TB	Incidence	5.0	4.7	5.3
40 to 44	42.5	Low SDI	Male	MDR-TB	Incidence	7.9	7.6	8.3
45 to 49	47.5	Low SDI	Male	MDR-TB	Incidence	11.7	11.2	12.3
50 to 54	52.5	Low SDI	Male	MDR-TB	Incidence	17.3	16.5	18.1
55 to 59	57.5	Low SDI	Male	MDR-TB	Incidence	26.8	25.5	28.2
60 to 64	62.5	Low SDI	Male	MDR-TB	Incidence	36.7	34.5	39.2
65 to 69	67.5	Low SDI	Male	MDR-TB	Incidence	50.5	47.0	54.4
70 to 74	72.5	Low SDI	Male	MDR-TB	Incidence	59.9	54.9	65.3
75 to 79	77.5	Low SDI	Male	MDR-TB	Incidence	79.5	71.5	88.3
80 to 84	82.5	Low SDI	Male	MDR-TB	Incidence	143.3	126.5	162.4
85 to 89	87.5	Low SDI	Male	MDR-TB	Incidence	230.4	195.0	272.1
90 to 94	92.5	Low SDI	Male	MDR-TB	Incidence	349.2	267.2	456.6
95 plus	97.5	Low SDI	Male	MDR-TB	Incidence	516.0	293.0	908.9
0 to 4	2.5	Low-middle SDI	Male	MDR-TB	Incidence	0.2	0.1	0.2
5 to 9	7.5	Low-middle SDI	Male	MDR-TB	Incidence	0.1	0.1	0.1
10 to 14	12.5	Low-middle SDI	Male	MDR-TB	Incidence	0.2	0.2	0.3
15 to 19	17.5	Low-middle SDI	Male	MDR-TB	Incidence	0.9	0.9	1.0
20 to 24	22.5	Low-middle SDI	Male	MDR-TB	Incidence	1.6	1.5	1.7
25 to 29	27.5	Low-middle SDI	Male	MDR-TB	Incidence	2.6	2.4	2.7
30 to 34	32.5	Low-middle SDI	Male	MDR-TB	Incidence	4.1	3.9	4.3
35 to 39	37.5	Low-middle SDI	Male	MDR-TB	Incidence	5.8	5.5	6.0
40 to 44	42.5	Low-middle SDI	Male	MDR-TB	Incidence	8.9	8.6	9.2
45 to 49	47.5	Low-middle SDI	Male	MDR-TB	Incidence	12.5	12.1	13.0
50 to 54	52.5	Low-middle SDI	Male	MDR-TB	Incidence	18.8	18.2	19.5
55 to 59	57.5	Low-middle SDI	Male	MDR-TB	Incidence	31.0	29.9	32.2
60 to 64	62.5	Low-middle SDI	Male	MDR-TB	Incidence	43.0	41.1	45.1
65 to 69	67.5	Low-middle SDI	Male	MDR-TB	Incidence	57.2	54.2	60.3
70 to 74	72.5	Low-middle SDI	Male	MDR-TB	Incidence	61.9	58.1	66.0
75 to 79	77.5	Low-middle SDI	Male	MDR-TB	Incidence	74.3	68.8	80.2

80 to 84	82.5	Low-middle SDI	Male	MDR-TB	Incidence	121.2	110.6	132.8
85 to 89	87.5	Low-middle SDI	Male	MDR-TB	Incidence	178.4	157.9	201.5
90 to 94	92.5	Low-middle SDI	Male	MDR-TB	Incidence	239.3	196.0	292.3
95 plus	97.5	Low-middle SDI	Male	MDR-TB	Incidence	258.6	166.5	401.5
0 to 4	2.5	Middle SDI	Male	MDR-TB	Incidence	1.1	1.0	1.3
5 to 9	7.5	Middle SDI	Male	MDR-TB	Incidence	0.7	0.6	0.8
10 to 14	12.5	Middle SDI	Male	MDR-TB	Incidence	1.0	0.9	1.1
15 to 19	17.5	Middle SDI	Male	MDR-TB	Incidence	2.7	2.5	2.9
20 to 24	22.5	Middle SDI	Male	MDR-TB	Incidence	3.7	3.4	4.0
25 to 29	27.5	Middle SDI	Male	MDR-TB	Incidence	4.0	3.7	4.3
30 to 34	32.5	Middle SDI	Male	MDR-TB	Incidence	4.9	4.6	5.3
35 to 39	37.5	Middle SDI	Male	MDR-TB	Incidence	5.4	5.1	5.7
40 to 44	42.5	Middle SDI	Male	MDR-TB	Incidence	6.4	6.1	6.8
45 to 49	47.5	Middle SDI	Male	MDR-TB	Incidence	7.0	6.6	7.4
50 to 54	52.5	Middle SDI	Male	MDR-TB	Incidence	8.3	7.8	8.8
55 to 59	57.5	Middle SDI	Male	MDR-TB	Incidence	10.4	9.8	11.1
60 to 64	62.5	Middle SDI	Male	MDR-TB	Incidence	11.1	10.4	12.0
65 to 69	67.5	Middle SDI	Male	MDR-TB	Incidence	12.3	11.3	13.3
70 to 74	72.5	Middle SDI	Male	MDR-TB	Incidence	10.4	9.5	11.4
75 to 79	77.5	Middle SDI	Male	MDR-TB	Incidence	9.0	8.1	10.1
80 to 84	82.5	Middle SDI	Male	MDR-TB	Incidence	10.6	9.4	12.1
85 to 89	87.5	Middle SDI	Male	MDR-TB	Incidence	11.1	9.3	13.3
90 to 94	92.5	Middle SDI	Male	MDR-TB	Incidence	10.3	7.5	14.1
95 plus	97.5	Middle SDI	Male	MDR-TB	Incidence	9.3	4.9	17.5
0 to 4	2.5	Global	Female	MDR-TB	Incidence	0.8	0.8	0.9
5 to 9	7.5	Global	Female	MDR-TB	Incidence	0.6	0.6	0.7
10 to 14	12.5	Global	Female	MDR-TB	Incidence	1.0	1.0	1.1
15 to 19	17.5	Global	Female	MDR-TB	Incidence	2.5	2.4	2.6
20 to 24	22.5	Global	Female	MDR-TB	Incidence	3.4	3.2	3.5
25 to 29	27.5	Global	Female	MDR-TB	Incidence	3.6	3.5	3.8
30 to 34	32.5	Global	Female	MDR-TB	Incidence	4.1	3.9	4.2
35 to 39	37.5	Global	Female	MDR-TB	Incidence	4.0	3.9	4.2
40 to 44	42.5	Global	Female	MDR-TB	Incidence	4.6	4.4	4.7
45 to 49	47.5	Global	Female	MDR-TB	Incidence	4.9	4.7	5.1
50 to 54	52.5	Global	Female	MDR-TB	Incidence	5.6	5.4	5.8
55 to 59	57.5	Global	Female	MDR-TB	Incidence	7.2	6.9	7.6
60 to 64	62.5	Global	Female	MDR-TB	Incidence	8.6	8.2	9.0
65 to 69	67.5	Global	Female	MDR-TB	Incidence	10.4	9.8	11.0
70 to 74	72.5	Global	Female	MDR-TB	Incidence	10.3	9.6	11.0
75 to 79	77.5	Global	Female	MDR-TB	Incidence	9.3	8.6	10.0
80 to 84	82.5	Global	Female	MDR-TB	Incidence	9.8	8.9	10.6
85 to 89	87.5	Global	Female	MDR-TB	Incidence	9.3	8.3	10.4
90 to 94	92.5	Global	Female	MDR-TB	Incidence	8.1	6.8	9.7
95 plus	97.5	Global	Female	MDR-TB	Incidence	6.3	4.4	9.0
0 to 4	2.5	High SDI	Female	MDR-TB	Incidence	0.3	0.3	0.4
5 to 9	7.5	High SDI	Female	MDR-TB	Incidence	0.2	0.2	0.3
10 to 14	12.5	High SDI	Female	MDR-TB	Incidence	0.3	0.3	0.4
15 to 19	17.5	High SDI	Female	MDR-TB	Incidence	0.6	0.6	0.7
20 to 24	22.5	High SDI	Female	MDR-TB	Incidence	0.8	0.7	0.9
25 to 29	27.5	High SDI	Female	MDR-TB	Incidence	0.7	0.6	0.7
30 to 34	32.5	High SDI	Female	MDR-TB	Incidence	0.6	0.5	0.6

35 to 39	37.5	High SDI	Female	MDR-TB	Incidence	0.4	0.4	0.4
40 to 44	42.5	High SDI	Female	MDR-TB	Incidence	0.3	0.3	0.4
45 to 49	47.5	High SDI	Female	MDR-TB	Incidence	0.2	0.2	0.3
50 to 54	52.5	High SDI	Female	MDR-TB	Incidence	0.2	0.2	0.2
55 to 59	57.5	High SDI	Female	MDR-TB	Incidence	0.2	0.2	0.2
60 to 64	62.5	High SDI	Female	MDR-TB	Incidence	0.2	0.1	0.2
65 to 69	67.5	High SDI	Female	MDR-TB	Incidence	0.2	0.2	0.3
70 to 74	72.5	High SDI	Female	MDR-TB	Incidence	0.2	0.2	0.2
75 to 79	77.5	High SDI	Female	MDR-TB	Incidence	0.1	0.1	0.2
80 to 84	82.5	High SDI	Female	MDR-TB	Incidence	0.1	0.1	0.2
85 to 89	87.5	High SDI	Female	MDR-TB	Incidence	0.1	0.1	0.1
90 to 94	92.5	High SDI	Female	MDR-TB	Incidence	0.1	0.1	0.1
95 plus	97.5	High SDI	Female	MDR-TB	Incidence	0.1	0.0	0.1
0 to 4	2.5	High-middle SDI	Female	MDR-TB	Incidence	1.3	1.2	1.5
5 to 9	7.5	High-middle SDI	Female	MDR-TB	Incidence	1.0	0.9	1.1
10 to 14	12.5	High-middle SDI	Female	MDR-TB	Incidence	1.3	1.2	1.5
15 to 19	17.5	High-middle SDI	Female	MDR-TB	Incidence	3.2	2.9	3.5
20 to 24	22.5	High-middle SDI	Female	MDR-TB	Incidence	4.6	4.2	4.9
25 to 29	27.5	High-middle SDI	Female	MDR-TB	Incidence	4.8	4.5	5.2
30 to 34	32.5	High-middle SDI	Female	MDR-TB	Incidence	5.3	5.0	5.6
35 to 39	37.5	High-middle SDI	Female	MDR-TB	Incidence	5.1	4.8	5.4
40 to 44	42.5	High-middle SDI	Female	MDR-TB	Incidence	5.2	4.9	5.5
45 to 49	47.5	High-middle SDI	Female	MDR-TB	Incidence	4.9	4.7	5.2
50 to 54	52.5	High-middle SDI	Female	MDR-TB	Incidence	4.9	4.6	5.3
55 to 59	57.5	High-middle SDI	Female	MDR-TB	Incidence	5.5	5.1	5.9
60 to 64	62.5	High-middle SDI	Female	MDR-TB	Incidence	5.7	5.3	6.2
65 to 69	67.5	High-middle SDI	Female	MDR-TB	Incidence	6.3	5.7	6.9
70 to 74	72.5	High-middle SDI	Female	MDR-TB	Incidence	5.8	5.2	6.4
75 to 79	77.5	High-middle SDI	Female	MDR-TB	Incidence	5.1	4.5	5.7
80 to 84	82.5	High-middle SDI	Female	MDR-TB	Incidence	5.1	4.4	5.9
85 to 89	87.5	High-middle SDI	Female	MDR-TB	Incidence	4.9	4.1	5.8
90 to 94	92.5	High-middle SDI	Female	MDR-TB	Incidence	4.4	3.3	5.7
95 plus	97.5	High-middle SDI	Female	MDR-TB	Incidence	3.6	2.0	6.3
0 to 4	2.5	Low SDI	Female	MDR-TB	Incidence	0.5	0.5	0.5
5 to 9	7.5	Low SDI	Female	MDR-TB	Incidence	0.3	0.3	0.4
10 to 14	12.5	Low SDI	Female	MDR-TB	Incidence	0.6	0.5	0.6
15 to 19	17.5	Low SDI	Female	MDR-TB	Incidence	1.6	1.5	1.7
20 to 24	22.5	Low SDI	Female	MDR-TB	Incidence	2.4	2.2	2.5
25 to 29	27.5	Low SDI	Female	MDR-TB	Incidence	3.1	2.9	3.3
30 to 34	32.5	Low SDI	Female	MDR-TB	Incidence	4.0	3.8	4.3
35 to 39	37.5	Low SDI	Female	MDR-TB	Incidence	4.6	4.4	4.8
40 to 44	42.5	Low SDI	Female	MDR-TB	Incidence	6.3	6.0	6.6
45 to 49	47.5	Low SDI	Female	MDR-TB	Incidence	8.1	7.7	8.5
50 to 54	52.5	Low SDI	Female	MDR-TB	Incidence	10.8	10.3	11.4
55 to 59	57.5	Low SDI	Female	MDR-TB	Incidence	16.3	15.4	17.2
60 to 64	62.5	Low SDI	Female	MDR-TB	Incidence	22.9	21.4	24.4
65 to 69	67.5	Low SDI	Female	MDR-TB	Incidence	33.4	31.0	36.0
70 to 74	72.5	Low SDI	Female	MDR-TB	Incidence	42.6	39.1	46.4
75 to 79	77.5	Low SDI	Female	MDR-TB	Incidence	53.4	48.2	59.3
80 to 84	82.5	Low SDI	Female	MDR-TB	Incidence	84.0	74.2	95.1
85 to 89	87.5	Low SDI	Female	MDR-TB	Incidence	122.4	103.9	144.3

90 to 94	92.5	Low SDI	Female	MDR-TB	Incidence	171.5	133.5	220.2
95 plus	97.5	Low SDI	Female	MDR-TB	Incidence	229.6	144.1	366.1
0 to 4	2.5	Low-middle SDI	Female	MDR-TB	Incidence	0.3	0.3	0.4
5 to 9	7.5	Low-middle SDI	Female	MDR-TB	Incidence	0.3	0.3	0.3
10 to 14	12.5	Low-middle SDI	Female	MDR-TB	Incidence	0.6	0.6	0.6
15 to 19	17.5	Low-middle SDI	Female	MDR-TB	Incidence	1.7	1.6	1.7
20 to 24	22.5	Low-middle SDI	Female	MDR-TB	Incidence	2.5	2.4	2.6
25 to 29	27.5	Low-middle SDI	Female	MDR-TB	Incidence	3.1	3.0	3.2
30 to 34	32.5	Low-middle SDI	Female	MDR-TB	Incidence	4.1	3.9	4.2
35 to 39	37.5	Low-middle SDI	Female	MDR-TB	Incidence	4.8	4.7	5.0
40 to 44	42.5	Low-middle SDI	Female	MDR-TB	Incidence	6.6	6.4	6.8
45 to 49	47.5	Low-middle SDI	Female	MDR-TB	Incidence	8.6	8.4	8.9
50 to 54	52.5	Low-middle SDI	Female	MDR-TB	Incidence	12.3	11.9	12.7
55 to 59	57.5	Low-middle SDI	Female	MDR-TB	Incidence	19.6	18.9	20.3
60 to 64	62.5	Low-middle SDI	Female	MDR-TB	Incidence	27.9	26.7	29.1
65 to 69	67.5	Low-middle SDI	Female	MDR-TB	Incidence	39.4	37.5	41.3
70 to 74	72.5	Low-middle SDI	Female	MDR-TB	Incidence	46.7	44.2	49.4
75 to 79	77.5	Low-middle SDI	Female	MDR-TB	Incidence	52.9	49.5	56.5
80 to 84	82.5	Low-middle SDI	Female	MDR-TB	Incidence	72.8	67.2	78.9
85 to 89	87.5	Low-middle SDI	Female	MDR-TB	Incidence	94.9	85.2	105.7
90 to 94	92.5	Low-middle SDI	Female	MDR-TB	Incidence	112.8	94.8	134.1
95 plus	97.5	Low-middle SDI	Female	MDR-TB	Incidence	105.8	73.6	152.0
0 to 4	2.5	Middle SDI	Female	MDR-TB	Incidence	2.4	2.3	2.6
5 to 9	7.5	Middle SDI	Female	MDR-TB	Incidence	1.6	1.5	1.7
10 to 14	12.5	Middle SDI	Female	MDR-TB	Incidence	2.2	2.1	2.4
15 to 19	17.5	Middle SDI	Female	MDR-TB	Incidence	4.8	4.6	5.1
20 to 24	22.5	Middle SDI	Female	MDR-TB	Incidence	5.9	5.6	6.2
25 to 29	27.5	Middle SDI	Female	MDR-TB	Incidence	5.4	5.1	5.7
30 to 34	32.5	Middle SDI	Female	MDR-TB	Incidence	5.3	5.0	5.5
35 to 39	37.5	Middle SDI	Female	MDR-TB	Incidence	4.4	4.3	4.6
40 to 44	42.5	Middle SDI	Female	MDR-TB	Incidence	4.4	4.2	4.6
45 to 49	47.5	Middle SDI	Female	MDR-TB	Incidence	4.3	4.1	4.5
50 to 54	52.5	Middle SDI	Female	MDR-TB	Incidence	4.5	4.3	4.7
55 to 59	57.5	Middle SDI	Female	MDR-TB	Incidence	5.3	5.1	5.6
60 to 64	62.5	Middle SDI	Female	MDR-TB	Incidence	5.6	5.3	5.9
65 to 69	67.5	Middle SDI	Female	MDR-TB	Incidence	6.2	5.8	6.6
70 to 74	72.5	Middle SDI	Female	MDR-TB	Incidence	5.6	5.3	6.0
75 to 79	77.5	Middle SDI	Female	MDR-TB	Incidence	4.7	4.3	5.0
80 to 84	82.5	Middle SDI	Female	MDR-TB	Incidence	4.6	4.2	5.1
85 to 89	87.5	Middle SDI	Female	MDR-TB	Incidence	4.4	3.9	5.0
90 to 94	92.5	Middle SDI	Female	MDR-TB	Incidence	4.0	3.3	4.9
95 plus	97.5	Middle SDI	Female	MDR-TB	Incidence	3.6	2.4	5.2
0 to 4	2.5	Global	Both	MDR-TB	Incidence	0.6	0.6	0.7
5 to 9	7.5	Global	Both	MDR-TB	Incidence	0.5	0.4	0.5
10 to 14	12.5	Global	Both	MDR-TB	Incidence	0.7	0.7	0.8
15 to 19	17.5	Global	Both	MDR-TB	Incidence	2.0	1.9	2.2
20 to 24	22.5	Global	Both	MDR-TB	Incidence	3.0	2.8	3.1
25 to 29	27.5	Global	Both	MDR-TB	Incidence	3.6	3.4	3.7
30 to 34	32.5	Global	Both	MDR-TB	Incidence	4.5	4.3	4.7
35 to 39	37.5	Global	Both	MDR-TB	Incidence	5.0	4.8	5.2
40 to 44	42.5	Global	Both	MDR-TB	Incidence	6.1	5.9	6.4

45 to 49	47.5	Global	Both	MDR-TB	Incidence	6.9	6.6	7.2
50 to 54	52.5	Global	Both	MDR-TB	Incidence	8.2	7.9	8.6
55 to 59	57.5	Global	Both	MDR-TB	Incidence	10.7	10.3	11.2
60 to 64	62.5	Global	Both	MDR-TB	Incidence	12.3	11.7	13.0
65 to 69	67.5	Global	Both	MDR-TB	Incidence	14.2	13.3	15.1
70 to 74	72.5	Global	Both	MDR-TB	Incidence	13.3	12.4	14.3
75 to 79	77.5	Global	Both	MDR-TB	Incidence	12.3	11.3	13.4
80 to 84	82.5	Global	Both	MDR-TB	Incidence	13.9	12.6	15.4
85 to 89	87.5	Global	Both	MDR-TB	Incidence	13.7	12.0	15.7
90 to 94	92.5	Global	Both	MDR-TB	Incidence	11.9	9.5	14.9
95 plus	97.5	Global	Both	MDR-TB	Incidence	9.4	5.9	14.9
0 to 4	2.5	High SDI	Both	MDR-TB	Incidence	0.3	0.2	0.3
5 to 9	7.5	High SDI	Both	MDR-TB	Incidence	0.2	0.1	0.2
10 to 14	12.5	High SDI	Both	MDR-TB	Incidence	0.2	0.2	0.3
15 to 19	17.5	High SDI	Both	MDR-TB	Incidence	0.6	0.5	0.6
20 to 24	22.5	High SDI	Both	MDR-TB	Incidence	0.8	0.7	0.8
25 to 29	27.5	High SDI	Both	MDR-TB	Incidence	0.6	0.6	0.7
30 to 34	32.5	High SDI	Both	MDR-TB	Incidence	0.6	0.6	0.7
35 to 39	37.5	High SDI	Both	MDR-TB	Incidence	0.5	0.5	0.5
40 to 44	42.5	High SDI	Both	MDR-TB	Incidence	0.4	0.4	0.5
45 to 49	47.5	High SDI	Both	MDR-TB	Incidence	0.4	0.3	0.4
50 to 54	52.5	High SDI	Both	MDR-TB	Incidence	0.3	0.3	0.3
55 to 59	57.5	High SDI	Both	MDR-TB	Incidence	0.3	0.3	0.3
60 to 64	62.5	High SDI	Both	MDR-TB	Incidence	0.3	0.3	0.3
65 to 69	67.5	High SDI	Both	MDR-TB	Incidence	0.3	0.3	0.4
70 to 74	72.5	High SDI	Both	MDR-TB	Incidence	0.3	0.2	0.3
75 to 79	77.5	High SDI	Both	MDR-TB	Incidence	0.2	0.2	0.2
80 to 84	82.5	High SDI	Both	MDR-TB	Incidence	0.2	0.2	0.2
85 to 89	87.5	High SDI	Both	MDR-TB	Incidence	0.2	0.2	0.2
90 to 94	92.5	High SDI	Both	MDR-TB	Incidence	0.1	0.1	0.2
95 plus	97.5	High SDI	Both	MDR-TB	Incidence	0.1	0.1	0.1
0 to 4	2.5	High-middle SDI	Both	MDR-TB	Incidence	1.1	0.9	1.3
5 to 9	7.5	High-middle SDI	Both	MDR-TB	Incidence	0.8	0.7	0.9
10 to 14	12.5	High-middle SDI	Both	MDR-TB	Incidence	1.0	0.9	1.2
15 to 19	17.5	High-middle SDI	Both	MDR-TB	Incidence	2.9	2.6	3.2
20 to 24	22.5	High-middle SDI	Both	MDR-TB	Incidence	4.6	4.2	5.0
25 to 29	27.5	High-middle SDI	Both	MDR-TB	Incidence	5.6	5.2	6.0
30 to 34	32.5	High-middle SDI	Both	MDR-TB	Incidence	7.1	6.7	7.6
35 to 39	37.5	High-middle SDI	Both	MDR-TB	Incidence	7.9	7.5	8.4
40 to 44	42.5	High-middle SDI	Both	MDR-TB	Incidence	9.1	8.6	9.6
45 to 49	47.5	High-middle SDI	Both	MDR-TB	Incidence	9.2	8.7	9.7
50 to 54	52.5	High-middle SDI	Both	MDR-TB	Incidence	9.6	9.0	10.2
55 to 59	57.5	High-middle SDI	Both	MDR-TB	Incidence	11.0	10.3	11.8
60 to 64	62.5	High-middle SDI	Both	MDR-TB	Incidence	10.8	9.9	11.7
65 to 69	67.5	High-middle SDI	Both	MDR-TB	Incidence	10.5	9.6	11.6
70 to 74	72.5	High-middle SDI	Both	MDR-TB	Incidence	9.1	8.2	10.2
75 to 79	77.5	High-middle SDI	Both	MDR-TB	Incidence	8.1	7.1	9.2
80 to 84	82.5	High-middle SDI	Both	MDR-TB	Incidence	8.4	7.2	9.8
85 to 89	87.5	High-middle SDI	Both	MDR-TB	Incidence	7.8	6.3	9.7
90 to 94	92.5	High-middle SDI	Both	MDR-TB	Incidence	6.5	4.5	9.3
95 plus	97.5	High-middle SDI	Both	MDR-TB	Incidence	5.1	2.3	11.2

0 to 4	2.5	Low SDI	Both	MDR-TB	Incidence	0.3	0.3	0.4
5 to 9	7.5	Low SDI	Both	MDR-TB	Incidence	0.2	0.2	0.2
10 to 14	12.5	Low SDI	Both	MDR-TB	Incidence	0.4	0.4	0.4
15 to 19	17.5	Low SDI	Both	MDR-TB	Incidence	1.2	1.1	1.3
20 to 24	22.5	Low SDI	Both	MDR-TB	Incidence	2.0	1.8	2.1
25 to 29	27.5	Low SDI	Both	MDR-TB	Incidence	2.7	2.6	2.9
30 to 34	32.5	Low SDI	Both	MDR-TB	Incidence	3.8	3.6	4.0
35 to 39	37.5	Low SDI	Both	MDR-TB	Incidence	4.8	4.6	5.0
40 to 44	42.5	Low SDI	Both	MDR-TB	Incidence	7.1	6.8	7.4
45 to 49	47.5	Low SDI	Both	MDR-TB	Incidence	9.9	9.5	10.4
50 to 54	52.5	Low SDI	Both	MDR-TB	Incidence	14.1	13.5	14.8
55 to 59	57.5	Low SDI	Both	MDR-TB	Incidence	21.5	20.5	22.6
60 to 64	62.5	Low SDI	Both	MDR-TB	Incidence	29.7	28.0	31.6
65 to 69	67.5	Low SDI	Both	MDR-TB	Incidence	41.9	39.1	44.8
70 to 74	72.5	Low SDI	Both	MDR-TB	Incidence	50.9	47.0	55.2
75 to 79	77.5	Low SDI	Both	MDR-TB	Incidence	65.6	59.6	72.3
80 to 84	82.5	Low SDI	Both	MDR-TB	Incidence	110.6	98.5	124.2
85 to 89	87.5	Low SDI	Both	MDR-TB	Incidence	168.3	144.3	196.3
90 to 94	92.5	Low SDI	Both	MDR-TB	Incidence	240.4	188.8	306.2
95 plus	97.5	Low SDI	Both	MDR-TB	Incidence	314.9	194.0	511.0
0 to 4	2.5	Low-middle SDI	Both	MDR-TB	Incidence	0.2	0.2	0.2
5 to 9	7.5	Low-middle SDI	Both	MDR-TB	Incidence	0.2	0.2	0.2
10 to 14	12.5	Low-middle SDI	Both	MDR-TB	Incidence	0.4	0.4	0.4
15 to 19	17.5	Low-middle SDI	Both	MDR-TB	Incidence	1.3	1.2	1.3
20 to 24	22.5	Low-middle SDI	Both	MDR-TB	Incidence	2.0	1.9	2.1
25 to 29	27.5	Low-middle SDI	Both	MDR-TB	Incidence	2.8	2.7	2.9
30 to 34	32.5	Low-middle SDI	Both	MDR-TB	Incidence	4.1	3.9	4.2
35 to 39	37.5	Low-middle SDI	Both	MDR-TB	Incidence	5.3	5.2	5.5
40 to 44	42.5	Low-middle SDI	Both	MDR-TB	Incidence	7.8	7.5	8.0
45 to 49	47.5	Low-middle SDI	Both	MDR-TB	Incidence	10.6	10.3	10.9
50 to 54	52.5	Low-middle SDI	Both	MDR-TB	Incidence	15.6	15.1	16.1
55 to 59	57.5	Low-middle SDI	Both	MDR-TB	Incidence	25.2	24.4	26.0
60 to 64	62.5	Low-middle SDI	Both	MDR-TB	Incidence	35.2	33.8	36.6
65 to 69	67.5	Low-middle SDI	Both	MDR-TB	Incidence	47.8	45.7	49.9
70 to 74	72.5	Low-middle SDI	Both	MDR-TB	Incidence	53.6	50.9	56.5
75 to 79	77.5	Low-middle SDI	Both	MDR-TB	Incidence	62.3	58.6	66.4
80 to 84	82.5	Low-middle SDI	Both	MDR-TB	Incidence	93.7	86.9	101.0
85 to 89	87.5	Low-middle SDI	Both	MDR-TB	Incidence	129.3	116.9	142.9
90 to 94	92.5	Low-middle SDI	Both	MDR-TB	Incidence	161.4	137.1	190.1
95 plus	97.5	Low-middle SDI	Both	MDR-TB	Incidence	157.3	110.5	223.8
0 to 4	2.5	Middle SDI	Both	MDR-TB	Incidence	1.7	1.6	1.8
5 to 9	7.5	Middle SDI	Both	MDR-TB	Incidence	1.1	1.0	1.2
10 to 14	12.5	Middle SDI	Both	MDR-TB	Incidence	1.5	1.4	1.7
15 to 19	17.5	Middle SDI	Both	MDR-TB	Incidence	3.6	3.4	3.9
20 to 24	22.5	Middle SDI	Both	MDR-TB	Incidence	4.7	4.4	5.0
25 to 29	27.5	Middle SDI	Both	MDR-TB	Incidence	4.6	4.4	4.9
30 to 34	32.5	Middle SDI	Both	MDR-TB	Incidence	5.1	4.9	5.4
35 to 39	37.5	Middle SDI	Both	MDR-TB	Incidence	4.9	4.7	5.2
40 to 44	42.5	Middle SDI	Both	MDR-TB	Incidence	5.4	5.2	5.7
45 to 49	47.5	Middle SDI	Both	MDR-TB	Incidence	5.7	5.4	5.9
50 to 54	52.5	Middle SDI	Both	MDR-TB	Incidence	6.4	6.1	6.7

55 to 59	57.5	Middle SDI	Both	MDR-TB	Incidence	7.8	7.4	8.2
60 to 64	62.5	Middle SDI	Both	MDR-TB	Incidence	8.3	7.8	8.8
65 to 69	67.5	Middle SDI	Both	MDR-TB	Incidence	9.1	8.5	9.7
70 to 74	72.5	Middle SDI	Both	MDR-TB	Incidence	7.9	7.3	8.5
75 to 79	77.5	Middle SDI	Both	MDR-TB	Incidence	6.7	6.1	7.3
80 to 84	82.5	Middle SDI	Both	MDR-TB	Incidence	7.2	6.5	8.0
85 to 89	87.5	Middle SDI	Both	MDR-TB	Incidence	7.0	6.1	8.0
90 to 94	92.5	Middle SDI	Both	MDR-TB	Incidence	6.3	5.0	7.9
95 plus	97.5	Middle SDI	Both	MDR-TB	Incidence	5.6	3.6	8.8

Figure 4B Period effect on the relative risk of MDR-TB among HIV-free individuals, grouped by SDI

label	period	location	sex	cause	measure	Rate Ratio	95%CI (lower)	95%CI (upper)
1990 to 1994	1992	Global	Male	MDR-TB	Incidence	0.4	0.4	0.4
1995 to 1999	1997	Global	Male	MDR-TB	Incidence	0.8	0.7	0.8
2000 to 2004	2002	Global	Male	MDR-TB	Incidence	1.0	1.0	1.0
2005 to 2009	2007	Global	Male	MDR-TB	Incidence	1.0	1.0	1.0
2010 to 2014	2012	Global	Male	MDR-TB	Incidence	0.8	0.8	0.9
2015 to 2019	2017	Global	Male	MDR-TB	Incidence	0.7	0.7	0.8
1990 to 1994	1992	High SDI	Male	MDR-TB	Incidence	0.8	0.7	0.8
1995 to 1999	1997	High SDI	Male	MDR-TB	Incidence	1.0	1.0	1.1
2000 to 2004	2002	High SDI	Male	MDR-TB	Incidence	1.0	1.0	1.0
2005 to 2009	2007	High SDI	Male	MDR-TB	Incidence	0.8	0.8	0.9
2010 to 2014	2012	High SDI	Male	MDR-TB	Incidence	0.5	0.5	0.6
2015 to 2019	2017	High SDI	Male	MDR-TB	Incidence	0.4	0.4	0.5
1990 to 1994	1992	High-middle SDI	Male	MDR-TB	Incidence	0.4	0.3	0.4
1995 to 1999	1997	High-middle SDI	Male	MDR-TB	Incidence	0.8	0.7	0.8
2000 to 2004	2002	High-middle SDI	Male	MDR-TB	Incidence	1.0	1.0	1.0
2005 to 2009	2007	High-middle SDI	Male	MDR-TB	Incidence	1.0	0.9	1.0
2010 to 2014	2012	High-middle SDI	Male	MDR-TB	Incidence	0.7	0.7	0.7
2015 to 2019	2017	High-middle SDI	Male	MDR-TB	Incidence	0.5	0.5	0.6
1990 to 1994	1992	Low SDI	Male	MDR-TB	Incidence	0.2	0.2	0.2
1995 to 1999	1997	Low SDI	Male	MDR-TB	Incidence	0.7	0.6	0.7
2000 to 2004	2002	Low SDI	Male	MDR-TB	Incidence	1.0	1.0	1.0
2005 to 2009	2007	Low SDI	Male	MDR-TB	Incidence	1.1	1.0	1.1
2010 to 2014	2012	Low SDI	Male	MDR-TB	Incidence	1.1	1.0	1.1
2015 to 2019	2017	Low SDI	Male	MDR-TB	Incidence	1.1	1.0	1.1
1990 to 1994	1992	Low-middle SDI	Male	MDR-TB	Incidence	0.2	0.2	0.2
1995 to 1999	1997	Low-middle SDI	Male	MDR-TB	Incidence	0.6	0.6	0.6
2000 to 2004	2002	Low-middle SDI	Male	MDR-TB	Incidence	1.0	1.0	1.0
2005 to 2009	2007	Low-middle SDI	Male	MDR-TB	Incidence	1.1	1.1	1.1
2010 to 2014	2012	Low-middle SDI	Male	MDR-TB	Incidence	1.0	1.0	1.1
2015 to 2019	2017	Low-middle SDI	Male	MDR-TB	Incidence	1.0	1.0	1.0
1990 to 1994	1992	Middle SDI	Male	MDR-TB	Incidence	0.6	0.5	0.6
1995 to 1999	1997	Middle SDI	Male	MDR-TB	Incidence	0.9	0.9	0.9
2000 to 2004	2002	Middle SDI	Male	MDR-TB	Incidence	1.0	1.0	1.0
2005 to 2009	2007	Middle SDI	Male	MDR-TB	Incidence	0.9	0.9	1.0
2010 to 2014	2012	Middle SDI	Male	MDR-TB	Incidence	0.7	0.7	0.8
2015 to 2019	2017	Middle SDI	Male	MDR-TB	Incidence	0.6	0.6	0.7
1990 to 1994	1992	Global	Female	MDR-TB	Incidence	0.4	0.4	0.4
1995 to 1999	1997	Global	Female	MDR-TB	Incidence	0.8	0.8	0.8
2000 to 2004	2002	Global	Female	MDR-TB	Incidence	1.0	1.0	1.0
2005 to 2009	2007	Global	Female	MDR-TB	Incidence	1.0	0.9	1.0
2010 to 2014	2012	Global	Female	MDR-TB	Incidence	0.8	0.8	0.8
2015 to 2019	2017	Global	Female	MDR-TB	Incidence	0.8	0.7	0.8
1990 to 1994	1992	High SDI	Female	MDR-TB	Incidence	0.8	0.8	0.9
1995 to 1999	1997	High SDI	Female	MDR-TB	Incidence	1.1	1.0	1.1
2000 to 2004	2002	High SDI	Female	MDR-TB	Incidence	1.0	1.0	1.0
2005 to 2009	2007	High SDI	Female	MDR-TB	Incidence	0.8	0.7	0.8
2010 to 2014	2012	High SDI	Female	MDR-TB	Incidence	0.5	0.5	0.6
2015 to 2019	2017	High SDI	Female	MDR-TB	Incidence	0.4	0.4	0.5
1990 to 1994	1992	High-middle SDI	Female	MDR-TB	Incidence	0.4	0.4	0.5
1995 to 1999	1997	High-middle SDI	Female	MDR-TB	Incidence	0.8	0.8	0.9
2000 to 2004	2002	High-middle SDI	Female	MDR-TB	Incidence	1.0	1.0	1.0
2005 to 2009	2007	High-middle SDI	Female	MDR-TB	Incidence	1.0	0.9	1.0
2010 to 2014	2012	High-middle SDI	Female	MDR-TB	Incidence	0.7	0.7	0.8
2015 to 2019	2017	High-middle SDI	Female	MDR-TB	Incidence	0.6	0.5	0.6
1990 to 1994	1992	Low SDI	Female	MDR-TB	Incidence	0.2	0.2	0.3
1995 to 1999	1997	Low SDI	Female	MDR-TB	Incidence	0.7	0.7	0.7
2000 to 2004	2002	Low SDI	Female	MDR-TB	Incidence	1.0	1.0	1.0

2005 to 2009	2007	Low SDI	Female	MDR-TB	Incidence	1.0	1.0	1.1
2010 to 2014	2012	Low SDI	Female	MDR-TB	Incidence	1.0	1.0	1.0
2015 to 2019	2017	Low SDI	Female	MDR-TB	Incidence	1.0	0.9	1.0
1990 to 1994	1992	Low-middle SDI	Female	MDR-TB	Incidence	0.2	0.2	0.2
1995 to 1999	1997	Low-middle SDI	Female	MDR-TB	Incidence	0.7	0.6	0.7
2000 to 2004	2002	Low-middle SDI	Female	MDR-TB	Incidence	1.0	1.0	1.0
2005 to 2009	2007	Low-middle SDI	Female	MDR-TB	Incidence	1.0	1.0	1.1
2010 to 2014	2012	Low-middle SDI	Female	MDR-TB	Incidence	1.0	1.0	1.0
2015 to 2019	2017	Low-middle SDI	Female	MDR-TB	Incidence	1.0	0.9	1.0
1990 to 1994	1992	Middle SDI	Female	MDR-TB	Incidence	0.6	0.6	0.6
1995 to 1999	1997	Middle SDI	Female	MDR-TB	Incidence	1.0	1.0	1.0
2000 to 2004	2002	Middle SDI	Female	MDR-TB	Incidence	1.0	1.0	1.0
2005 to 2009	2007	Middle SDI	Female	MDR-TB	Incidence	0.9	0.8	0.9
2010 to 2014	2012	Middle SDI	Female	MDR-TB	Incidence	0.6	0.6	0.7
2015 to 2019	2017	Middle SDI	Female	MDR-TB	Incidence	0.6	0.5	0.6
1990 to 1994	1992	Global	Both	MDR-TB	Incidence	0.4	0.4	0.4
1995 to 1999	1997	Global	Both	MDR-TB	Incidence	0.8	0.8	0.8
2000 to 2004	2002	Global	Both	MDR-TB	Incidence	1.0	1.0	1.0
2005 to 2009	2007	Global	Both	MDR-TB	Incidence	1.0	1.0	1.0
2010 to 2014	2012	Global	Both	MDR-TB	Incidence	0.8	0.8	0.9
2015 to 2019	2017	Global	Both	MDR-TB	Incidence	0.8	0.7	0.8
1990 to 1994	1992	High SDI	Both	MDR-TB	Incidence	0.8	0.8	0.8
1995 to 1999	1997	High SDI	Both	MDR-TB	Incidence	1.0	1.0	1.1
2000 to 2004	2002	High SDI	Both	MDR-TB	Incidence	1.0	1.0	1.0
2005 to 2009	2007	High SDI	Both	MDR-TB	Incidence	0.8	0.8	0.8
2010 to 2014	2012	High SDI	Both	MDR-TB	Incidence	0.5	0.5	0.5
2015 to 2019	2017	High SDI	Both	MDR-TB	Incidence	0.5	0.4	0.5
1990 to 1994	1992	High-middle SDI	Both	MDR-TB	Incidence	0.4	0.4	0.4
1995 to 1999	1997	High-middle SDI	Both	MDR-TB	Incidence	0.8	0.8	0.8
2000 to 2004	2002	High-middle SDI	Both	MDR-TB	Incidence	1.0	1.0	1.0
2005 to 2009	2007	High-middle SDI	Both	MDR-TB	Incidence	1.0	0.9	1.0
2010 to 2014	2012	High-middle SDI	Both	MDR-TB	Incidence	0.7	0.7	0.8
2015 to 2019	2017	High-middle SDI	Both	MDR-TB	Incidence	0.5	0.5	0.6
1990 to 1994	1992	Low SDI	Both	MDR-TB	Incidence	0.2	0.2	0.2
1995 to 1999	1997	Low SDI	Both	MDR-TB	Incidence	0.7	0.6	0.7
2000 to 2004	2002	Low SDI	Both	MDR-TB	Incidence	1.0	1.0	1.0
2005 to 2009	2007	Low SDI	Both	MDR-TB	Incidence	1.1	1.0	1.1
2010 to 2014	2012	Low SDI	Both	MDR-TB	Incidence	1.0	1.0	1.1
2015 to 2019	2017	Low SDI	Both	MDR-TB	Incidence	1.0	1.0	1.1
1990 to 1994	1992	Low-middle SDI	Both	MDR-TB	Incidence	0.2	0.2	0.2
1995 to 1999	1997	Low-middle SDI	Both	MDR-TB	Incidence	0.6	0.6	0.7
2000 to 2004	2002	Low-middle SDI	Both	MDR-TB	Incidence	1.0	1.0	1.0
2005 to 2009	2007	Low-middle SDI	Both	MDR-TB	Incidence	1.1	1.0	1.1
2010 to 2014	2012	Low-middle SDI	Both	MDR-TB	Incidence	1.0	1.0	1.0
2015 to 2019	2017	Low-middle SDI	Both	MDR-TB	Incidence	1.0	1.0	1.0
1990 to 1994	1992	Middle SDI	Both	MDR-TB	Incidence	0.6	0.6	0.6
1995 to 1999	1997	Middle SDI	Both	MDR-TB	Incidence	0.9	0.9	1.0
2000 to 2004	2002	Middle SDI	Both	MDR-TB	Incidence	1.0	1.0	1.0
2005 to 2009	2007	Middle SDI	Both	MDR-TB	Incidence	0.9	0.9	0.9
2010 to 2014	2012	Middle SDI	Both	MDR-TB	Incidence	0.7	0.7	0.7
2015 to 2019	2017	Middle SDI	Both	MDR-TB	Incidence	0.6	0.6	0.6

Figure 4C Cohort effect on the relative risk of MDR-TB among HIV-free individuals, grouped by SDI

label	Cohort	location	sex	cause	measure	Rate Ratio	95%CI (lower)	95%CI (upper)
1890 to 1899	1895	Global	Male	MDR-TB	Incidence	0.4	0.0	8.6
1895 to 1904	1900	Global	Male	MDR-TB	Incidence	0.4	0.2	1.3
1900 to 1909	1905	Global	Male	MDR-TB	Incidence	0.5	0.3	0.8
1905 to 1914	1910	Global	Male	MDR-TB	Incidence	0.5	0.4	0.7
1910 to 1919	1915	Global	Male	MDR-TB	Incidence	0.6	0.5	0.7
1915 to 1924	1920	Global	Male	MDR-TB	Incidence	0.6	0.5	0.7
1920 to 1929	1925	Global	Male	MDR-TB	Incidence	0.6	0.5	0.7
1925 to 1934	1930	Global	Male	MDR-TB	Incidence	0.6	0.5	0.7
1930 to 1939	1935	Global	Male	MDR-TB	Incidence	0.6	0.6	0.7
1935 to 1944	1940	Global	Male	MDR-TB	Incidence	0.6	0.6	0.7
1940 to 1949	1945	Global	Male	MDR-TB	Incidence	0.6	0.6	0.7
1945 to 1954	1950	Global	Male	MDR-TB	Incidence	0.7	0.7	0.8
1950 to 1959	1955	Global	Male	MDR-TB	Incidence	0.9	0.8	0.9
1955 to 1964	1960	Global	Male	MDR-TB	Incidence	1.0	1.0	1.0
1960 to 1969	1965	Global	Male	MDR-TB	Incidence	1.1	1.1	1.2
1965 to 1974	1970	Global	Male	MDR-TB	Incidence	1.3	1.3	1.4
1970 to 1979	1975	Global	Male	MDR-TB	Incidence	1.7	1.6	1.8
1975 to 1984	1980	Global	Male	MDR-TB	Incidence	2.0	1.9	2.1
1980 to 1989	1985	Global	Male	MDR-TB	Incidence	2.4	2.2	2.6
1985 to 1994	1990	Global	Male	MDR-TB	Incidence	2.8	2.6	3.0
1990 to 1999	1995	Global	Male	MDR-TB	Incidence	3.2	2.9	3.5
1995 to 2004	2000	Global	Male	MDR-TB	Incidence	3.6	3.2	3.9
2000 to 2009	2005	Global	Male	MDR-TB	Incidence	3.6	3.2	4.1
2005 to 2014	2010	Global	Male	MDR-TB	Incidence	4.0	3.4	4.6
2010 to 2019	2015	Global	Male	MDR-TB	Incidence	4.5	3.7	5.4
1890 to 1899	1895	High SDI	Male	MDR-TB	Incidence	9.5	2.2	41.0
1895 to 1904	1900	High SDI	Male	MDR-TB	Incidence	10.1	5.7	17.7
1900 to 1909	1905	High SDI	Male	MDR-TB	Incidence	7.8	5.6	10.7
1905 to 1914	1910	High SDI	Male	MDR-TB	Incidence	6.4	5.2	8.0
1910 to 1919	1915	High SDI	Male	MDR-TB	Incidence	5.5	4.6	6.5
1915 to 1924	1920	High SDI	Male	MDR-TB	Incidence	4.5	3.9	5.3
1920 to 1929	1925	High SDI	Male	MDR-TB	Incidence	3.7	3.3	4.3
1925 to 1934	1930	High SDI	Male	MDR-TB	Incidence	3.2	2.8	3.6
1930 to 1939	1935	High SDI	Male	MDR-TB	Incidence	2.6	2.3	2.9
1935 to 1944	1940	High SDI	Male	MDR-TB	Incidence	1.9	1.7	2.1
1940 to 1949	1945	High SDI	Male	MDR-TB	Incidence	1.4	1.3	1.6
1945 to 1954	1950	High SDI	Male	MDR-TB	Incidence	1.2	1.1	1.3
1950 to 1959	1955	High SDI	Male	MDR-TB	Incidence	1.1	1.0	1.2
1955 to 1964	1960	High SDI	Male	MDR-TB	Incidence	1.0	1.0	1.0
1960 to 1969	1965	High SDI	Male	MDR-TB	Incidence	0.9	0.8	1.0
1965 to 1974	1970	High SDI	Male	MDR-TB	Incidence	0.8	0.8	0.9
1970 to 1979	1975	High SDI	Male	MDR-TB	Incidence	0.8	0.7	0.9
1975 to 1984	1980	High SDI	Male	MDR-TB	Incidence	0.8	0.7	0.8
1980 to 1989	1985	High SDI	Male	MDR-TB	Incidence	0.7	0.6	0.8
1985 to 1994	1990	High SDI	Male	MDR-TB	Incidence	0.6	0.6	0.7
1990 to 1999	1995	High SDI	Male	MDR-TB	Incidence	0.5	0.4	0.6

1995 to 2004	2000	High SDI	Male	MDR-TB	Incidence	0.4	0.4	0.6
2000 to 2009	2005	High SDI	Male	MDR-TB	Incidence	0.4	0.3	0.5
2005 to 2014	2010	High SDI	Male	MDR-TB	Incidence	0.4	0.3	0.6
2010 to 2019	2015	High SDI	Male	MDR-TB	Incidence	0.3	0.2	0.6
1890 to 1899	1895	High-middle SDI	Male	MDR-TB	Incidence	1.1	0.0	89.4
1895 to 1904	1900	High-middle SDI	Male	MDR-TB	Incidence	1.0	0.2	4.3
1900 to 1909	1905	High-middle SDI	Male	MDR-TB	Incidence	1.0	0.5	1.9
1905 to 1914	1910	High-middle SDI	Male	MDR-TB	Incidence	1.0	0.7	1.4
1910 to 1919	1915	High-middle SDI	Male	MDR-TB	Incidence	1.0	0.8	1.2
1915 to 1924	1920	High-middle SDI	Male	MDR-TB	Incidence	0.9	0.8	1.1
1920 to 1929	1925	High-middle SDI	Male	MDR-TB	Incidence	0.8	0.7	1.0
1925 to 1934	1930	High-middle SDI	Male	MDR-TB	Incidence	0.8	0.7	0.9
1930 to 1939	1935	High-middle SDI	Male	MDR-TB	Incidence	0.7	0.6	0.8
1935 to 1944	1940	High-middle SDI	Male	MDR-TB	Incidence	0.7	0.6	0.8
1940 to 1949	1945	High-middle SDI	Male	MDR-TB	Incidence	0.6	0.6	0.7
1945 to 1954	1950	High-middle SDI	Male	MDR-TB	Incidence	0.8	0.7	0.8
1950 to 1959	1955	High-middle SDI	Male	MDR-TB	Incidence	0.9	0.8	0.9
1955 to 1964	1960	High-middle SDI	Male	MDR-TB	Incidence	1.0	1.0	1.0
1960 to 1969	1965	High-middle SDI	Male	MDR-TB	Incidence	1.0	0.9	1.1
1965 to 1974	1970	High-middle SDI	Male	MDR-TB	Incidence	1.1	1.1	1.2
1970 to 1979	1975	High-middle SDI	Male	MDR-TB	Incidence	1.4	1.3	1.5
1975 to 1984	1980	High-middle SDI	Male	MDR-TB	Incidence	1.6	1.5	1.7
1980 to 1989	1985	High-middle SDI	Male	MDR-TB	Incidence	1.7	1.6	1.9
1985 to 1994	1990	High-middle SDI	Male	MDR-TB	Incidence	1.8	1.6	2.0
1990 to 1999	1995	High-middle SDI	Male	MDR-TB	Incidence	1.8	1.6	2.0
1995 to 2004	2000	High-middle SDI	Male	MDR-TB	Incidence	1.7	1.5	2.0
2000 to 2009	2005	High-middle SDI	Male	MDR-TB	Incidence	1.5	1.2	1.9
2005 to 2014	2010	High-middle SDI	Male	MDR-TB	Incidence	1.5	1.1	2.0
2010 to 2019	2015	High-middle SDI	Male	MDR-TB	Incidence	1.4	0.9	2.1
1890 to 1899	1895	Low SDI	Male	MDR-TB	Incidence	0.0	0.0	1.2
1895 to 1904	1900	Low SDI	Male	MDR-TB	Incidence	0.0	0.0	0.2
1900 to 1909	1905	Low SDI	Male	MDR-TB	Incidence	0.1	0.0	0.1
1905 to 1914	1910	Low SDI	Male	MDR-TB	Incidence	0.1	0.1	0.1
1910 to 1919	1915	Low SDI	Male	MDR-TB	Incidence	0.1	0.1	0.1
1915 to 1924	1920	Low SDI	Male	MDR-TB	Incidence	0.1	0.1	0.1
1920 to 1929	1925	Low SDI	Male	MDR-TB	Incidence	0.2	0.1	0.2
1925 to 1934	1930	Low SDI	Male	MDR-TB	Incidence	0.2	0.2	0.2
1930 to 1939	1935	Low SDI	Male	MDR-TB	Incidence	0.3	0.3	0.3
1935 to 1944	1940	Low SDI	Male	MDR-TB	Incidence	0.4	0.3	0.4
1940 to 1949	1945	Low SDI	Male	MDR-TB	Incidence	0.5	0.4	0.5
1945 to 1954	1950	Low SDI	Male	MDR-TB	Incidence	0.6	0.6	0.6
1950 to 1959	1955	Low SDI	Male	MDR-TB	Incidence	0.8	0.7	0.8
1955 to 1964	1960	Low SDI	Male	MDR-TB	Incidence	1.0	1.0	1.0
1960 to 1969	1965	Low SDI	Male	MDR-TB	Incidence	1.3	1.2	1.4
1965 to 1974	1970	Low SDI	Male	MDR-TB	Incidence	1.8	1.7	1.9
1970 to 1979	1975	Low SDI	Male	MDR-TB	Incidence	2.5	2.3	2.6
1975 to 1984	1980	Low SDI	Male	MDR-TB	Incidence	3.4	3.2	3.6
1980 to 1989	1985	Low SDI	Male	MDR-TB	Incidence	4.5	4.2	4.9
1985 to 1994	1990	Low SDI	Male	MDR-TB	Incidence	6.0	5.5	6.4
1990 to 1999	1995	Low SDI	Male	MDR-TB	Incidence	7.6	7.0	8.3
1995 to 2004	2000	Low SDI	Male	MDR-TB	Incidence	9.4	8.6	10.3

2000 to 2009	2005	Low SDI	Male	MDR-TB	Incidence	10.8	9.8	12.0
2005 to 2014	2010	Low SDI	Male	MDR-TB	Incidence	13.4	11.9	15.0
2010 to 2019	2015	Low SDI	Male	MDR-TB	Incidence	16.9	14.9	19.3
1890 to 1899	1895	Low-middle SDI	Male	MDR-TB	Incidence	0.0	0.0	1.0
1895 to 1904	1900	Low-middle SDI	Male	MDR-TB	Incidence	0.1	0.0	0.1
1900 to 1909	1905	Low-middle SDI	Male	MDR-TB	Incidence	0.1	0.0	0.1
1905 to 1914	1910	Low-middle SDI	Male	MDR-TB	Incidence	0.1	0.1	0.1
1910 to 1919	1915	Low-middle SDI	Male	MDR-TB	Incidence	0.1	0.1	0.1
1915 to 1924	1920	Low-middle SDI	Male	MDR-TB	Incidence	0.2	0.1	0.2
1920 to 1929	1925	Low-middle SDI	Male	MDR-TB	Incidence	0.2	0.2	0.2
1925 to 1934	1930	Low-middle SDI	Male	MDR-TB	Incidence	0.2	0.2	0.3
1930 to 1939	1935	Low-middle SDI	Male	MDR-TB	Incidence	0.3	0.3	0.3
1935 to 1944	1940	Low-middle SDI	Male	MDR-TB	Incidence	0.3	0.3	0.4
1940 to 1949	1945	Low-middle SDI	Male	MDR-TB	Incidence	0.4	0.4	0.5
1945 to 1954	1950	Low-middle SDI	Male	MDR-TB	Incidence	0.6	0.5	0.6
1950 to 1959	1955	Low-middle SDI	Male	MDR-TB	Incidence	0.8	0.7	0.8
1955 to 1964	1960	Low-middle SDI	Male	MDR-TB	Incidence	1.0	1.0	1.0
1960 to 1969	1965	Low-middle SDI	Male	MDR-TB	Incidence	1.3	1.3	1.4
1965 to 1974	1970	Low-middle SDI	Male	MDR-TB	Incidence	1.9	1.8	1.9
1970 to 1979	1975	Low-middle SDI	Male	MDR-TB	Incidence	2.6	2.5	2.7
1975 to 1984	1980	Low-middle SDI	Male	MDR-TB	Incidence	3.5	3.4	3.7
1980 to 1989	1985	Low-middle SDI	Male	MDR-TB	Incidence	4.8	4.5	5.0
1985 to 1994	1990	Low-middle SDI	Male	MDR-TB	Incidence	6.3	6.0	6.7
1990 to 1999	1995	Low-middle SDI	Male	MDR-TB	Incidence	8.0	7.5	8.6
1995 to 2004	2000	Low-middle SDI	Male	MDR-TB	Incidence	10.0	9.3	10.8
2000 to 2009	2005	Low-middle SDI	Male	MDR-TB	Incidence	11.3	10.3	12.4
2005 to 2014	2010	Low-middle SDI	Male	MDR-TB	Incidence	13.5	12.0	15.1
2010 to 2019	2015	Low-middle SDI	Male	MDR-TB	Incidence	17.2	14.9	19.8
1890 to 1899	1895	Middle SDI	Male	MDR-TB	Incidence	1.8	0.1	29.1
1895 to 1904	1900	Middle SDI	Male	MDR-TB	Incidence	1.7	0.6	4.8
1900 to 1909	1905	Middle SDI	Male	MDR-TB	Incidence	1.9	1.2	2.9
1905 to 1914	1910	Middle SDI	Male	MDR-TB	Incidence	1.9	1.5	2.5
1910 to 1919	1915	Middle SDI	Male	MDR-TB	Incidence	1.9	1.6	2.2
1915 to 1924	1920	Middle SDI	Male	MDR-TB	Incidence	1.7	1.5	2.0
1920 to 1929	1925	Middle SDI	Male	MDR-TB	Incidence	1.6	1.4	1.7
1925 to 1934	1930	Middle SDI	Male	MDR-TB	Incidence	1.4	1.3	1.5
1930 to 1939	1935	Middle SDI	Male	MDR-TB	Incidence	1.2	1.1	1.3
1935 to 1944	1940	Middle SDI	Male	MDR-TB	Incidence	1.1	1.0	1.2
1940 to 1949	1945	Middle SDI	Male	MDR-TB	Incidence	1.0	0.9	1.1
1945 to 1954	1950	Middle SDI	Male	MDR-TB	Incidence	1.0	0.9	1.0
1950 to 1959	1955	Middle SDI	Male	MDR-TB	Incidence	1.0	0.9	1.0
1955 to 1964	1960	Middle SDI	Male	MDR-TB	Incidence	1.0	1.0	1.0
1960 to 1969	1965	Middle SDI	Male	MDR-TB	Incidence	1.0	1.0	1.1
1965 to 1974	1970	Middle SDI	Male	MDR-TB	Incidence	1.1	1.1	1.2
1970 to 1979	1975	Middle SDI	Male	MDR-TB	Incidence	1.2	1.2	1.3
1975 to 1984	1980	Middle SDI	Male	MDR-TB	Incidence	1.4	1.3	1.5
1980 to 1989	1985	Middle SDI	Male	MDR-TB	Incidence	1.5	1.4	1.6
1985 to 1994	1990	Middle SDI	Male	MDR-TB	Incidence	1.6	1.5	1.7
1990 to 1999	1995	Middle SDI	Male	MDR-TB	Incidence	1.6	1.5	1.8
1995 to 2004	2000	Middle SDI	Male	MDR-TB	Incidence	1.6	1.4	1.8
2000 to 2009	2005	Middle SDI	Male	MDR-TB	Incidence	1.5	1.3	1.7

2005 to 2014	2010	Middle SDI	Male	MDR-TB	Incidence	1.4	1.2	1.6
2010 to 2019	2015	Middle SDI	Male	MDR-TB	Incidence	1.2	1.0	1.5
1890 to 1899	1895	Global	Female	MDR-TB	Incidence	0.6	0.2	2.6
1895 to 1904	1900	Global	Female	MDR-TB	Incidence	0.6	0.3	1.0
1900 to 1909	1905	Global	Female	MDR-TB	Incidence	0.6	0.4	0.8
1905 to 1914	1910	Global	Female	MDR-TB	Incidence	0.6	0.5	0.7
1910 to 1919	1915	Global	Female	MDR-TB	Incidence	0.6	0.5	0.7
1915 to 1924	1920	Global	Female	MDR-TB	Incidence	0.6	0.5	0.7
1920 to 1929	1925	Global	Female	MDR-TB	Incidence	0.6	0.5	0.6
1925 to 1934	1930	Global	Female	MDR-TB	Incidence	0.6	0.6	0.7
1930 to 1939	1935	Global	Female	MDR-TB	Incidence	0.6	0.6	0.7
1935 to 1944	1940	Global	Female	MDR-TB	Incidence	0.7	0.6	0.7
1940 to 1949	1945	Global	Female	MDR-TB	Incidence	0.7	0.7	0.7
1945 to 1954	1950	Global	Female	MDR-TB	Incidence	0.8	0.7	0.8
1950 to 1959	1955	Global	Female	MDR-TB	Incidence	0.9	0.8	0.9
1955 to 1964	1960	Global	Female	MDR-TB	Incidence	1.0	1.0	1.0
1960 to 1969	1965	Global	Female	MDR-TB	Incidence	1.1	1.1	1.2
1965 to 1974	1970	Global	Female	MDR-TB	Incidence	1.4	1.3	1.4
1970 to 1979	1975	Global	Female	MDR-TB	Incidence	1.6	1.5	1.7
1975 to 1984	1980	Global	Female	MDR-TB	Incidence	1.8	1.7	1.9
1980 to 1989	1985	Global	Female	MDR-TB	Incidence	2.1	2.0	2.1
1985 to 1994	1990	Global	Female	MDR-TB	Incidence	2.3	2.2	2.4
1990 to 1999	1995	Global	Female	MDR-TB	Incidence	2.5	2.4	2.7
1995 to 2004	2000	Global	Female	MDR-TB	Incidence	2.8	2.7	3.0
2000 to 2009	2005	Global	Female	MDR-TB	Incidence	3.0	2.8	3.2
2005 to 2014	2010	Global	Female	MDR-TB	Incidence	3.1	2.9	3.4
2010 to 2019	2015	Global	Female	MDR-TB	Incidence	3.3	3.0	3.7
1890 to 1899	1895	High SDI	Female	MDR-TB	Incidence	10.9	3.2	37.0
1895 to 1904	1900	High SDI	Female	MDR-TB	Incidence	9.0	5.2	15.7
1900 to 1909	1905	High SDI	Female	MDR-TB	Incidence	6.9	4.9	9.8
1905 to 1914	1910	High SDI	Female	MDR-TB	Incidence	5.7	4.4	7.4
1910 to 1919	1915	High SDI	Female	MDR-TB	Incidence	4.9	3.9	6.1
1915 to 1924	1920	High SDI	Female	MDR-TB	Incidence	4.2	3.4	5.1
1920 to 1929	1925	High SDI	Female	MDR-TB	Incidence	3.6	3.0	4.3
1925 to 1934	1930	High SDI	Female	MDR-TB	Incidence	3.2	2.7	3.8
1930 to 1939	1935	High SDI	Female	MDR-TB	Incidence	2.7	2.3	3.2
1935 to 1944	1940	High SDI	Female	MDR-TB	Incidence	2.0	1.7	2.3
1940 to 1949	1945	High SDI	Female	MDR-TB	Incidence	1.4	1.2	1.7
1945 to 1954	1950	High SDI	Female	MDR-TB	Incidence	1.2	1.1	1.4
1950 to 1959	1955	High SDI	Female	MDR-TB	Incidence	1.1	1.0	1.3
1955 to 1964	1960	High SDI	Female	MDR-TB	Incidence	1.0	1.0	1.0
1960 to 1969	1965	High SDI	Female	MDR-TB	Incidence	0.9	0.8	1.0
1965 to 1974	1970	High SDI	Female	MDR-TB	Incidence	0.8	0.7	0.9
1970 to 1979	1975	High SDI	Female	MDR-TB	Incidence	0.7	0.6	0.8
1975 to 1984	1980	High SDI	Female	MDR-TB	Incidence	0.7	0.6	0.8
1980 to 1989	1985	High SDI	Female	MDR-TB	Incidence	0.6	0.5	0.7
1985 to 1994	1990	High SDI	Female	MDR-TB	Incidence	0.5	0.4	0.6
1990 to 1999	1995	High SDI	Female	MDR-TB	Incidence	0.4	0.3	0.5
1995 to 2004	2000	High SDI	Female	MDR-TB	Incidence	0.3	0.3	0.4
2000 to 2009	2005	High SDI	Female	MDR-TB	Incidence	0.3	0.2	0.4
2005 to 2014	2010	High SDI	Female	MDR-TB	Incidence	0.2	0.2	0.4

2010 to 2019	2015	High SDI	Female	MDR-TB	Incidence	0.2	0.1	0.4
1890 to 1899	1895	High-middle SDI	Female	MDR-TB	Incidence	1.4	0.2	12.2
1895 to 1904	1900	High-middle SDI	Female	MDR-TB	Incidence	1.3	0.6	2.8
1900 to 1909	1905	High-middle SDI	Female	MDR-TB	Incidence	1.2	0.8	1.8
1905 to 1914	1910	High-middle SDI	Female	MDR-TB	Incidence	1.1	0.9	1.5
1910 to 1919	1915	High-middle SDI	Female	MDR-TB	Incidence	1.1	0.9	1.4
1915 to 1924	1920	High-middle SDI	Female	MDR-TB	Incidence	1.1	0.9	1.3
1920 to 1929	1925	High-middle SDI	Female	MDR-TB	Incidence	1.0	0.9	1.1
1925 to 1934	1930	High-middle SDI	Female	MDR-TB	Incidence	0.9	0.8	1.0
1930 to 1939	1935	High-middle SDI	Female	MDR-TB	Incidence	0.8	0.8	0.9
1935 to 1944	1940	High-middle SDI	Female	MDR-TB	Incidence	0.8	0.7	0.9
1940 to 1949	1945	High-middle SDI	Female	MDR-TB	Incidence	0.7	0.7	0.8
1945 to 1954	1950	High-middle SDI	Female	MDR-TB	Incidence	0.8	0.8	0.9
1950 to 1959	1955	High-middle SDI	Female	MDR-TB	Incidence	0.9	0.8	1.0
1955 to 1964	1960	High-middle SDI	Female	MDR-TB	Incidence	1.0	1.0	1.0
1960 to 1969	1965	High-middle SDI	Female	MDR-TB	Incidence	1.0	1.0	1.1
1965 to 1974	1970	High-middle SDI	Female	MDR-TB	Incidence	1.1	1.1	1.2
1970 to 1979	1975	High-middle SDI	Female	MDR-TB	Incidence	1.3	1.2	1.4
1975 to 1984	1980	High-middle SDI	Female	MDR-TB	Incidence	1.4	1.4	1.5
1980 to 1989	1985	High-middle SDI	Female	MDR-TB	Incidence	1.5	1.4	1.6
1985 to 1994	1990	High-middle SDI	Female	MDR-TB	Incidence	1.5	1.4	1.6
1990 to 1999	1995	High-middle SDI	Female	MDR-TB	Incidence	1.4	1.3	1.6
1995 to 2004	2000	High-middle SDI	Female	MDR-TB	Incidence	1.4	1.2	1.5
2000 to 2009	2005	High-middle SDI	Female	MDR-TB	Incidence	1.2	1.0	1.4
2005 to 2014	2010	High-middle SDI	Female	MDR-TB	Incidence	1.2	1.0	1.4
2010 to 2019	2015	High-middle SDI	Female	MDR-TB	Incidence	1.1	0.8	1.4
1890 to 1899	1895	Low SDI	Female	MDR-TB	Incidence	0.0	0.0	0.8
1895 to 1904	1900	Low SDI	Female	MDR-TB	Incidence	0.1	0.0	0.2
1900 to 1909	1905	Low SDI	Female	MDR-TB	Incidence	0.1	0.0	0.1
1905 to 1914	1910	Low SDI	Female	MDR-TB	Incidence	0.1	0.1	0.1
1910 to 1919	1915	Low SDI	Female	MDR-TB	Incidence	0.1	0.1	0.1
1915 to 1924	1920	Low SDI	Female	MDR-TB	Incidence	0.1	0.1	0.2
1920 to 1929	1925	Low SDI	Female	MDR-TB	Incidence	0.2	0.2	0.2
1925 to 1934	1930	Low SDI	Female	MDR-TB	Incidence	0.2	0.2	0.2
1930 to 1939	1935	Low SDI	Female	MDR-TB	Incidence	0.3	0.3	0.3
1935 to 1944	1940	Low SDI	Female	MDR-TB	Incidence	0.4	0.3	0.4
1940 to 1949	1945	Low SDI	Female	MDR-TB	Incidence	0.5	0.5	0.5
1945 to 1954	1950	Low SDI	Female	MDR-TB	Incidence	0.6	0.6	0.7
1950 to 1959	1955	Low SDI	Female	MDR-TB	Incidence	0.8	0.8	0.8
1955 to 1964	1960	Low SDI	Female	MDR-TB	Incidence	1.0	1.0	1.0
1960 to 1969	1965	Low SDI	Female	MDR-TB	Incidence	1.2	1.2	1.3
1965 to 1974	1970	Low SDI	Female	MDR-TB	Incidence	1.6	1.5	1.7
1970 to 1979	1975	Low SDI	Female	MDR-TB	Incidence	2.0	1.9	2.1
1975 to 1984	1980	Low SDI	Female	MDR-TB	Incidence	2.6	2.4	2.7
1980 to 1989	1985	Low SDI	Female	MDR-TB	Incidence	3.2	3.0	3.4
1985 to 1994	1990	Low SDI	Female	MDR-TB	Incidence	4.0	3.7	4.3
1990 to 1999	1995	Low SDI	Female	MDR-TB	Incidence	5.0	4.6	5.3
1995 to 2004	2000	Low SDI	Female	MDR-TB	Incidence	6.0	5.6	6.5
2000 to 2009	2005	Low SDI	Female	MDR-TB	Incidence	7.1	6.6	7.8
2005 to 2014	2010	Low SDI	Female	MDR-TB	Incidence	8.7	7.9	9.5
2010 to 2019	2015	Low SDI	Female	MDR-TB	Incidence	10.4	9.4	11.5

1890 to 1899	1895	Low-middle SDI	Female	MDR-TB	Incidence	0.1	0.0	0.7
1895 to 1904	1900	Low-middle SDI	Female	MDR-TB	Incidence	0.1	0.0	0.2
1900 to 1909	1905	Low-middle SDI	Female	MDR-TB	Incidence	0.1	0.1	0.1
1905 to 1914	1910	Low-middle SDI	Female	MDR-TB	Incidence	0.1	0.1	0.1
1910 to 1919	1915	Low-middle SDI	Female	MDR-TB	Incidence	0.1	0.1	0.2
1915 to 1924	1920	Low-middle SDI	Female	MDR-TB	Incidence	0.2	0.1	0.2
1920 to 1929	1925	Low-middle SDI	Female	MDR-TB	Incidence	0.2	0.2	0.2
1925 to 1934	1930	Low-middle SDI	Female	MDR-TB	Incidence	0.2	0.2	0.3
1930 to 1939	1935	Low-middle SDI	Female	MDR-TB	Incidence	0.3	0.3	0.3
1935 to 1944	1940	Low-middle SDI	Female	MDR-TB	Incidence	0.4	0.3	0.4
1940 to 1949	1945	Low-middle SDI	Female	MDR-TB	Incidence	0.4	0.4	0.5
1945 to 1954	1950	Low-middle SDI	Female	MDR-TB	Incidence	0.6	0.6	0.6
1950 to 1959	1955	Low-middle SDI	Female	MDR-TB	Incidence	0.8	0.7	0.8
1955 to 1964	1960	Low-middle SDI	Female	MDR-TB	Incidence	1.0	1.0	1.0
1960 to 1969	1965	Low-middle SDI	Female	MDR-TB	Incidence	1.3	1.3	1.4
1965 to 1974	1970	Low-middle SDI	Female	MDR-TB	Incidence	1.8	1.7	1.9
1970 to 1979	1975	Low-middle SDI	Female	MDR-TB	Incidence	2.4	2.3	2.5
1975 to 1984	1980	Low-middle SDI	Female	MDR-TB	Incidence	3.1	3.0	3.2
1980 to 1989	1985	Low-middle SDI	Female	MDR-TB	Incidence	3.9	3.7	4.1
1985 to 1994	1990	Low-middle SDI	Female	MDR-TB	Incidence	4.8	4.6	5.0
1990 to 1999	1995	Low-middle SDI	Female	MDR-TB	Incidence	5.8	5.5	6.1
1995 to 2004	2000	Low-middle SDI	Female	MDR-TB	Incidence	7.1	6.7	7.5
2000 to 2009	2005	Low-middle SDI	Female	MDR-TB	Incidence	8.2	7.7	8.8
2005 to 2014	2010	Low-middle SDI	Female	MDR-TB	Incidence	9.7	9.0	10.5
2010 to 2019	2015	Low-middle SDI	Female	MDR-TB	Incidence	11.5	10.5	12.7
1890 to 1899	1895	Middle SDI	Female	MDR-TB	Incidence	3.8	1.0	14.1
1895 to 1904	1900	Middle SDI	Female	MDR-TB	Incidence	3.5	2.1	5.9
1900 to 1909	1905	Middle SDI	Female	MDR-TB	Incidence	3.2	2.5	4.1
1905 to 1914	1910	Middle SDI	Female	MDR-TB	Incidence	2.9	2.4	3.4
1910 to 1919	1915	Middle SDI	Female	MDR-TB	Incidence	2.6	2.3	2.9
1915 to 1924	1920	Middle SDI	Female	MDR-TB	Incidence	2.2	2.0	2.5
1920 to 1929	1925	Middle SDI	Female	MDR-TB	Incidence	1.9	1.8	2.1
1925 to 1934	1930	Middle SDI	Female	MDR-TB	Incidence	1.7	1.5	1.8
1930 to 1939	1935	Middle SDI	Female	MDR-TB	Incidence	1.5	1.4	1.6
1935 to 1944	1940	Middle SDI	Female	MDR-TB	Incidence	1.3	1.2	1.4
1940 to 1949	1945	Middle SDI	Female	MDR-TB	Incidence	1.2	1.1	1.2
1945 to 1954	1950	Middle SDI	Female	MDR-TB	Incidence	1.1	1.0	1.1
1950 to 1959	1955	Middle SDI	Female	MDR-TB	Incidence	1.0	1.0	1.1
1955 to 1964	1960	Middle SDI	Female	MDR-TB	Incidence	1.0	1.0	1.0
1960 to 1969	1965	Middle SDI	Female	MDR-TB	Incidence	1.0	1.0	1.0
1965 to 1974	1970	Middle SDI	Female	MDR-TB	Incidence	1.0	1.0	1.1
1970 to 1979	1975	Middle SDI	Female	MDR-TB	Incidence	1.0	1.0	1.1
1975 to 1984	1980	Middle SDI	Female	MDR-TB	Incidence	1.1	1.0	1.1
1980 to 1989	1985	Middle SDI	Female	MDR-TB	Incidence	1.1	1.0	1.1
1985 to 1994	1990	Middle SDI	Female	MDR-TB	Incidence	1.1	1.0	1.1
1990 to 1999	1995	Middle SDI	Female	MDR-TB	Incidence	1.1	1.0	1.1
1995 to 2004	2000	Middle SDI	Female	MDR-TB	Incidence	1.1	1.0	1.1
2000 to 2009	2005	Middle SDI	Female	MDR-TB	Incidence	1.0	0.9	1.1
2005 to 2014	2010	Middle SDI	Female	MDR-TB	Incidence	0.9	0.8	0.9
2010 to 2019	2015	Middle SDI	Female	MDR-TB	Incidence	0.7	0.6	0.8
1890 to 1899	1895	Global	Both	MDR-TB	Incidence	0.5	0.1	3.8

1895 to 1904	1900	Global	Both	MDR-TB	Incidence	0.5	0.2	1.0
1900 to 1909	1905	Global	Both	MDR-TB	Incidence	0.5	0.4	0.7
1905 to 1914	1910	Global	Both	MDR-TB	Incidence	0.5	0.4	0.7
1910 to 1919	1915	Global	Both	MDR-TB	Incidence	0.6	0.5	0.7
1915 to 1924	1920	Global	Both	MDR-TB	Incidence	0.6	0.5	0.7
1920 to 1929	1925	Global	Both	MDR-TB	Incidence	0.6	0.5	0.6
1925 to 1934	1930	Global	Both	MDR-TB	Incidence	0.6	0.6	0.7
1930 to 1939	1935	Global	Both	MDR-TB	Incidence	0.6	0.6	0.7
1935 to 1944	1940	Global	Both	MDR-TB	Incidence	0.6	0.6	0.7
1940 to 1949	1945	Global	Both	MDR-TB	Incidence	0.7	0.6	0.7
1945 to 1954	1950	Global	Both	MDR-TB	Incidence	0.7	0.7	0.8
1950 to 1959	1955	Global	Both	MDR-TB	Incidence	0.9	0.8	0.9
1955 to 1964	1960	Global	Both	MDR-TB	Incidence	1.0	1.0	1.0
1960 to 1969	1965	Global	Both	MDR-TB	Incidence	1.1	1.1	1.2
1965 to 1974	1970	Global	Both	MDR-TB	Incidence	1.3	1.3	1.4
1970 to 1979	1975	Global	Both	MDR-TB	Incidence	1.6	1.6	1.7
1975 to 1984	1980	Global	Both	MDR-TB	Incidence	1.9	1.8	2.0
1980 to 1989	1985	Global	Both	MDR-TB	Incidence	2.2	2.1	2.3
1985 to 1994	1990	Global	Both	MDR-TB	Incidence	2.5	2.4	2.7
1990 to 1999	1995	Global	Both	MDR-TB	Incidence	2.8	2.6	3.0
1995 to 2004	2000	Global	Both	MDR-TB	Incidence	3.1	2.9	3.4
2000 to 2009	2005	Global	Both	MDR-TB	Incidence	3.3	3.0	3.6
2005 to 2014	2010	Global	Both	MDR-TB	Incidence	3.5	3.2	3.9
2010 to 2019	2015	Global	Both	MDR-TB	Incidence	3.9	3.4	4.4
1890 to 1899	1895	High SDI	Both	MDR-TB	Incidence	11.6	4.9	27.4
1895 to 1904	1900	High SDI	Both	MDR-TB	Incidence	8.8	5.9	13.0
1900 to 1909	1905	High SDI	Both	MDR-TB	Incidence	6.8	5.4	8.6
1905 to 1914	1910	High SDI	Both	MDR-TB	Incidence	5.7	4.8	6.7
1910 to 1919	1915	High SDI	Both	MDR-TB	Incidence	5.0	4.3	5.7
1915 to 1924	1920	High SDI	Both	MDR-TB	Incidence	4.2	3.7	4.7
1920 to 1929	1925	High SDI	Both	MDR-TB	Incidence	3.6	3.2	4.0
1925 to 1934	1930	High SDI	Both	MDR-TB	Incidence	3.1	2.8	3.5
1930 to 1939	1935	High SDI	Both	MDR-TB	Incidence	2.6	2.4	2.8
1935 to 1944	1940	High SDI	Both	MDR-TB	Incidence	1.9	1.8	2.1
1940 to 1949	1945	High SDI	Both	MDR-TB	Incidence	1.4	1.3	1.5
1945 to 1954	1950	High SDI	Both	MDR-TB	Incidence	1.2	1.1	1.3
1950 to 1959	1955	High SDI	Both	MDR-TB	Incidence	1.1	1.0	1.2
1955 to 1964	1960	High SDI	Both	MDR-TB	Incidence	1.0	1.0	1.0
1960 to 1969	1965	High SDI	Both	MDR-TB	Incidence	0.9	0.8	0.9
1965 to 1974	1970	High SDI	Both	MDR-TB	Incidence	0.8	0.7	0.9
1970 to 1979	1975	High SDI	Both	MDR-TB	Incidence	0.8	0.7	0.8
1975 to 1984	1980	High SDI	Both	MDR-TB	Incidence	0.7	0.7	0.8
1980 to 1989	1985	High SDI	Both	MDR-TB	Incidence	0.7	0.6	0.7
1985 to 1994	1990	High SDI	Both	MDR-TB	Incidence	0.6	0.5	0.6
1990 to 1999	1995	High SDI	Both	MDR-TB	Incidence	0.5	0.4	0.5
1995 to 2004	2000	High SDI	Both	MDR-TB	Incidence	0.4	0.3	0.4
2000 to 2009	2005	High SDI	Both	MDR-TB	Incidence	0.3	0.3	0.4
2005 to 2014	2010	High SDI	Both	MDR-TB	Incidence	0.3	0.2	0.4
2010 to 2019	2015	High SDI	Both	MDR-TB	Incidence	0.3	0.2	0.4
1890 to 1899	1895	High-middle SDI	Both	MDR-TB	Incidence	1.2	0.1	28.1
1895 to 1904	1900	High-middle SDI	Both	MDR-TB	Incidence	1.1	0.3	3.3

1900 to 1909	1905	High-middle SDI	Both	MDR-TB	Incidence	1.0	0.6	1.7
1905 to 1914	1910	High-middle SDI	Both	MDR-TB	Incidence	1.0	0.7	1.3
1910 to 1919	1915	High-middle SDI	Both	MDR-TB	Incidence	1.0	0.8	1.2
1915 to 1924	1920	High-middle SDI	Both	MDR-TB	Incidence	0.9	0.8	1.1
1920 to 1929	1925	High-middle SDI	Both	MDR-TB	Incidence	0.8	0.7	1.0
1925 to 1934	1930	High-middle SDI	Both	MDR-TB	Incidence	0.8	0.7	0.9
1930 to 1939	1935	High-middle SDI	Both	MDR-TB	Incidence	0.7	0.7	0.8
1935 to 1944	1940	High-middle SDI	Both	MDR-TB	Incidence	0.7	0.7	0.8
1940 to 1949	1945	High-middle SDI	Both	MDR-TB	Incidence	0.7	0.6	0.7
1945 to 1954	1950	High-middle SDI	Both	MDR-TB	Incidence	0.8	0.7	0.8
1950 to 1959	1955	High-middle SDI	Both	MDR-TB	Incidence	0.9	0.8	0.9
1955 to 1964	1960	High-middle SDI	Both	MDR-TB	Incidence	1.0	1.0	1.0
1960 to 1969	1965	High-middle SDI	Both	MDR-TB	Incidence	1.0	0.9	1.1
1965 to 1974	1970	High-middle SDI	Both	MDR-TB	Incidence	1.1	1.1	1.2
1970 to 1979	1975	High-middle SDI	Both	MDR-TB	Incidence	1.4	1.3	1.4
1975 to 1984	1980	High-middle SDI	Both	MDR-TB	Incidence	1.5	1.4	1.6
1980 to 1989	1985	High-middle SDI	Both	MDR-TB	Incidence	1.6	1.5	1.8
1985 to 1994	1990	High-middle SDI	Both	MDR-TB	Incidence	1.6	1.5	1.8
1990 to 1999	1995	High-middle SDI	Both	MDR-TB	Incidence	1.6	1.4	1.8
1995 to 2004	2000	High-middle SDI	Both	MDR-TB	Incidence	1.5	1.3	1.8
2000 to 2009	2005	High-middle SDI	Both	MDR-TB	Incidence	1.3	1.1	1.6
2005 to 2014	2010	High-middle SDI	Both	MDR-TB	Incidence	1.4	1.1	1.8
2010 to 2019	2015	High-middle SDI	Both	MDR-TB	Incidence	1.2	0.9	1.8
1890 to 1899	1895	Low SDI	Both	MDR-TB	Incidence	0.0	0.0	0.8
1895 to 1904	1900	Low SDI	Both	MDR-TB	Incidence	0.1	0.0	0.1
1900 to 1909	1905	Low SDI	Both	MDR-TB	Incidence	0.1	0.0	0.1
1905 to 1914	1910	Low SDI	Both	MDR-TB	Incidence	0.1	0.1	0.1
1910 to 1919	1915	Low SDI	Both	MDR-TB	Incidence	0.1	0.1	0.1
1915 to 1924	1920	Low SDI	Both	MDR-TB	Incidence	0.1	0.1	0.2
1920 to 1929	1925	Low SDI	Both	MDR-TB	Incidence	0.2	0.2	0.2
1925 to 1934	1930	Low SDI	Both	MDR-TB	Incidence	0.2	0.2	0.2
1930 to 1939	1935	Low SDI	Both	MDR-TB	Incidence	0.3	0.3	0.3
1935 to 1944	1940	Low SDI	Both	MDR-TB	Incidence	0.4	0.3	0.4
1940 to 1949	1945	Low SDI	Both	MDR-TB	Incidence	0.5	0.4	0.5
1945 to 1954	1950	Low SDI	Both	MDR-TB	Incidence	0.6	0.6	0.7
1950 to 1959	1955	Low SDI	Both	MDR-TB	Incidence	0.8	0.8	0.8
1955 to 1964	1960	Low SDI	Both	MDR-TB	Incidence	1.0	1.0	1.0
1960 to 1969	1965	Low SDI	Both	MDR-TB	Incidence	1.3	1.2	1.3
1965 to 1974	1970	Low SDI	Both	MDR-TB	Incidence	1.7	1.6	1.8
1970 to 1979	1975	Low SDI	Both	MDR-TB	Incidence	2.3	2.1	2.4
1975 to 1984	1980	Low SDI	Both	MDR-TB	Incidence	3.0	2.8	3.2
1980 to 1989	1985	Low SDI	Both	MDR-TB	Incidence	3.8	3.6	4.1
1985 to 1994	1990	Low SDI	Both	MDR-TB	Incidence	4.9	4.6	5.2
1990 to 1999	1995	Low SDI	Both	MDR-TB	Incidence	6.2	5.7	6.6
1995 to 2004	2000	Low SDI	Both	MDR-TB	Incidence	7.5	7.0	8.1
2000 to 2009	2005	Low SDI	Both	MDR-TB	Incidence	8.8	8.1	9.6
2005 to 2014	2010	Low SDI	Both	MDR-TB	Incidence	10.7	9.8	11.8
2010 to 2019	2015	Low SDI	Both	MDR-TB	Incidence	13.1	11.8	14.6
1890 to 1899	1895	Low-middle SDI	Both	MDR-TB	Incidence	0.1	0.0	0.6
1895 to 1904	1900	Low-middle SDI	Both	MDR-TB	Incidence	0.1	0.0	0.1
1900 to 1909	1905	Low-middle SDI	Both	MDR-TB	Incidence	0.1	0.1	0.1

1905 to 1914	1910	Low-middle SDI	Both	MDR-TB	Incidence	0.1	0.1	0.1
1910 to 1919	1915	Low-middle SDI	Both	MDR-TB	Incidence	0.1	0.1	0.1
1915 to 1924	1920	Low-middle SDI	Both	MDR-TB	Incidence	0.2	0.1	0.2
1920 to 1929	1925	Low-middle SDI	Both	MDR-TB	Incidence	0.2	0.2	0.2
1925 to 1934	1930	Low-middle SDI	Both	MDR-TB	Incidence	0.2	0.2	0.3
1930 to 1939	1935	Low-middle SDI	Both	MDR-TB	Incidence	0.3	0.3	0.3
1935 to 1944	1940	Low-middle SDI	Both	MDR-TB	Incidence	0.4	0.3	0.4
1940 to 1949	1945	Low-middle SDI	Both	MDR-TB	Incidence	0.4	0.4	0.5
1945 to 1954	1950	Low-middle SDI	Both	MDR-TB	Incidence	0.6	0.6	0.6
1950 to 1959	1955	Low-middle SDI	Both	MDR-TB	Incidence	0.8	0.7	0.8
1955 to 1964	1960	Low-middle SDI	Both	MDR-TB	Incidence	1.0	1.0	1.0
1960 to 1969	1965	Low-middle SDI	Both	MDR-TB	Incidence	1.3	1.3	1.4
1965 to 1974	1970	Low-middle SDI	Both	MDR-TB	Incidence	1.8	1.8	1.9
1970 to 1979	1975	Low-middle SDI	Both	MDR-TB	Incidence	2.5	2.4	2.6
1975 to 1984	1980	Low-middle SDI	Both	MDR-TB	Incidence	3.3	3.2	3.4
1980 to 1989	1985	Low-middle SDI	Both	MDR-TB	Incidence	4.3	4.1	4.5
1985 to 1994	1990	Low-middle SDI	Both	MDR-TB	Incidence	5.5	5.2	5.7
1990 to 1999	1995	Low-middle SDI	Both	MDR-TB	Incidence	6.7	6.4	7.1
1995 to 2004	2000	Low-middle SDI	Both	MDR-TB	Incidence	8.3	7.9	8.8
2000 to 2009	2005	Low-middle SDI	Both	MDR-TB	Incidence	9.5	8.9	10.2
2005 to 2014	2010	Low-middle SDI	Both	MDR-TB	Incidence	11.3	10.4	12.3
2010 to 2019	2015	Low-middle SDI	Both	MDR-TB	Incidence	13.9	12.5	15.3
1890 to 1899	1895	Middle SDI	Both	MDR-TB	Incidence	2.6	0.5	14.4
1895 to 1904	1900	Middle SDI	Both	MDR-TB	Incidence	2.5	1.3	4.8
1900 to 1909	1905	Middle SDI	Both	MDR-TB	Incidence	2.4	1.7	3.2
1905 to 1914	1910	Middle SDI	Both	MDR-TB	Incidence	2.3	1.9	2.8
1910 to 1919	1915	Middle SDI	Both	MDR-TB	Incidence	2.1	1.8	2.4
1915 to 1924	1920	Middle SDI	Both	MDR-TB	Incidence	1.9	1.7	2.1
1920 to 1929	1925	Middle SDI	Both	MDR-TB	Incidence	1.7	1.6	1.9
1925 to 1934	1930	Middle SDI	Both	MDR-TB	Incidence	1.5	1.4	1.6
1930 to 1939	1935	Middle SDI	Both	MDR-TB	Incidence	1.3	1.2	1.4
1935 to 1944	1940	Middle SDI	Both	MDR-TB	Incidence	1.2	1.1	1.3
1940 to 1949	1945	Middle SDI	Both	MDR-TB	Incidence	1.1	1.0	1.1
1945 to 1954	1950	Middle SDI	Both	MDR-TB	Incidence	1.0	1.0	1.1
1950 to 1959	1955	Middle SDI	Both	MDR-TB	Incidence	1.0	0.9	1.0
1955 to 1964	1960	Middle SDI	Both	MDR-TB	Incidence	1.0	1.0	1.0
1960 to 1969	1965	Middle SDI	Both	MDR-TB	Incidence	1.0	1.0	1.1
1965 to 1974	1970	Middle SDI	Both	MDR-TB	Incidence	1.1	1.0	1.1
1970 to 1979	1975	Middle SDI	Both	MDR-TB	Incidence	1.2	1.1	1.2
1975 to 1984	1980	Middle SDI	Both	MDR-TB	Incidence	1.2	1.1	1.3
1980 to 1989	1985	Middle SDI	Both	MDR-TB	Incidence	1.3	1.2	1.3
1985 to 1994	1990	Middle SDI	Both	MDR-TB	Incidence	1.3	1.2	1.4
1990 to 1999	1995	Middle SDI	Both	MDR-TB	Incidence	1.3	1.2	1.4
1995 to 2004	2000	Middle SDI	Both	MDR-TB	Incidence	1.3	1.2	1.4
2000 to 2009	2005	Middle SDI	Both	MDR-TB	Incidence	1.2	1.1	1.3
2005 to 2014	2010	Middle SDI	Both	MDR-TB	Incidence	1.1	1.0	1.2
2010 to 2019	2015	Middle SDI	Both	MDR-TB	Incidence	0.9	0.8	1.1

Figure 5 Projected incidence of MDR-TB from 2020 to 2030 using the Nordpred model, by sex and SDI

measure	location	cause	sex	year	ASR	crude rate	case
Incidence	Global	MDR-TB	Male	1990	1.2	1.0	27782
Incidence	Global	MDR-TB	Male	1991	2.1	1.8	49760
Incidence	Global	MDR-TB	Male	1992	2.9	2.6	71832
Incidence	Global	MDR-TB	Male	1993	3.7	3.3	92842
Incidence	Global	MDR-TB	Male	1994	4.4	3.9	111579
Incidence	Global	MDR-TB	Male	1995	4.9	4.4	126743
Incidence	Global	MDR-TB	Male	1996	5.3	4.8	141350
Incidence	Global	MDR-TB	Male	1997	5.8	5.3	158271
Incidence	Global	MDR-TB	Male	1998	6.3	5.8	175988
Incidence	Global	MDR-TB	Male	1999	6.8	6.3	192879
Incidence	Global	MDR-TB	Male	2000	7.2	6.7	207118
Incidence	Global	MDR-TB	Male	2001	7.5	7.0	220285
Incidence	Global	MDR-TB	Male	2002	7.8	7.4	234084
Incidence	Global	MDR-TB	Male	2003	8.1	7.7	246988
Incidence	Global	MDR-TB	Male	2004	8.2	7.9	257441
Incidence	Global	MDR-TB	Male	2005	8.3	8.0	263631
Incidence	Global	MDR-TB	Male	2006	8.2	7.9	265247
Incidence	Global	MDR-TB	Male	2007	8.0	7.8	263754
Incidence	Global	MDR-TB	Male	2008	7.7	7.6	260453
Incidence	Global	MDR-TB	Male	2009	7.5	7.4	256803
Incidence	Global	MDR-TB	Male	2010	7.3	7.2	254429
Incidence	Global	MDR-TB	Male	2011	7.1	7.1	251368
Incidence	Global	MDR-TB	Male	2012	6.8	6.8	245733
Incidence	Global	MDR-TB	Male	2013	6.5	6.6	239610
Incidence	Global	MDR-TB	Male	2014	6.3	6.4	235171
Incidence	Global	MDR-TB	Male	2015	6.2	6.3	234732
Incidence	Global	MDR-TB	Male	2016	6.3	6.4	239764
Incidence	Global	MDR-TB	Male	2017	6.3	6.5	246055
Incidence	Global	MDR-TB	Male	2018	6.4	6.5	251105
Incidence	Global	MDR-TB	Male	2019	6.4	6.6	256219
Incidence	Global	MDR-TB	Male	2020	6.1	6.3	247349
Incidence	Global	MDR-TB	Male	2021	6.0	6.2	247117
Incidence	Global	MDR-TB	Male	2022	5.9	6.1	246723
Incidence	Global	MDR-TB	Male	2023	5.8	6.1	247810
Incidence	Global	MDR-TB	Male	2024	5.8	6.1	248810
Incidence	Global	MDR-TB	Male	2025	5.7	6.0	249710
Incidence	Global	MDR-TB	Male	2026	5.7	6.0	250507
Incidence	Global	MDR-TB	Male	2027	5.6	6.0	251204
Incidence	Global	MDR-TB	Male	2028	5.6	5.9	251805
Incidence	Global	MDR-TB	Male	2029	5.5	5.9	252318
Incidence	Global	MDR-TB	Male	2030	5.5	5.9	252733
Incidence	Global	MDR-TB	Female	1990	0.9	0.9	23951
Incidence	Global	MDR-TB	Female	1991	1.7	1.6	44084
Incidence	Global	MDR-TB	Female	1992	2.4	2.3	63882
Incidence	Global	MDR-TB	Female	1993	3.0	3.0	82325
Incidence	Global	MDR-TB	Female	1994	3.6	3.5	98337
Incidence	Global	MDR-TB	Female	1995	4.0	3.9	110772

Incidence	Global	MDR-TB	Female	1996	4.3	4.2	122359
Incidence	Global	MDR-TB	Female	1997	4.7	4.6	135786
Incidence	Global	MDR-TB	Female	1998	5.1	5.0	149721
Incidence	Global	MDR-TB	Female	1999	5.4	5.4	162669
Incidence	Global	MDR-TB	Female	2000	5.7	5.7	172975
Incidence	Global	MDR-TB	Female	2001	5.9	5.9	181131
Incidence	Global	MDR-TB	Female	2002	6.0	6.0	188535
Incidence	Global	MDR-TB	Female	2003	6.1	6.1	194820
Incidence	Global	MDR-TB	Female	2004	6.2	6.2	199574
Incidence	Global	MDR-TB	Female	2005	6.2	6.2	202240
Incidence	Global	MDR-TB	Female	2006	6.1	6.1	202417
Incidence	Global	MDR-TB	Female	2007	5.9	6.0	200584
Incidence	Global	MDR-TB	Female	2008	5.7	5.8	197684
Incidence	Global	MDR-TB	Female	2009	5.6	5.7	194802
Incidence	Global	MDR-TB	Female	2010	5.5	5.6	193143
Incidence	Global	MDR-TB	Female	2011	5.3	5.4	191130
Incidence	Global	MDR-TB	Female	2012	5.1	5.3	187249
Incidence	Global	MDR-TB	Female	2013	5.0	5.1	182983
Incidence	Global	MDR-TB	Female	2014	4.8	4.9	179856
Incidence	Global	MDR-TB	Female	2015	4.7	4.9	179459
Incidence	Global	MDR-TB	Female	2016	4.8	4.9	181959
Incidence	Global	MDR-TB	Female	2017	4.8	4.9	185254
Incidence	Global	MDR-TB	Female	2018	4.8	5.0	189202
Incidence	Global	MDR-TB	Female	2019	4.9	5.0	194377
Incidence	Global	MDR-TB	Female	2020	4.6	4.7	186637
Incidence	Global	MDR-TB	Female	2021	4.6	4.7	186241
Incidence	Global	MDR-TB	Female	2022	4.5	4.6	185769
Incidence	Global	MDR-TB	Female	2023	4.5	4.6	186116
Incidence	Global	MDR-TB	Female	2024	4.4	4.6	186402
Incidence	Global	MDR-TB	Female	2025	4.4	4.5	186628
Incidence	Global	MDR-TB	Female	2026	4.3	4.5	186798
Incidence	Global	MDR-TB	Female	2027	4.3	4.5	186920
Incidence	Global	MDR-TB	Female	2028	4.2	4.4	186991
Incidence	Global	MDR-TB	Female	2029	4.2	4.4	187013
Incidence	Global	MDR-TB	Female	2030	4.2	4.3	186979
Incidence	Global	MDR-TB	Both	1990	1.0	1.0	51734
Incidence	Global	MDR-TB	Both	1991	1.9	1.7	93843
Incidence	Global	MDR-TB	Both	1992	2.6	2.5	135713
Incidence	Global	MDR-TB	Both	1993	3.3	3.1	175167
Incidence	Global	MDR-TB	Both	1994	3.9	3.7	209916
Incidence	Global	MDR-TB	Both	1995	4.4	4.1	237515
Incidence	Global	MDR-TB	Both	1996	4.8	4.5	263708
Incidence	Global	MDR-TB	Both	1997	5.2	5.0	294057
Incidence	Global	MDR-TB	Both	1998	5.7	5.4	325709
Incidence	Global	MDR-TB	Both	1999	6.1	5.9	355547
Incidence	Global	MDR-TB	Both	2000	6.4	6.2	380093
Incidence	Global	MDR-TB	Both	2001	6.6	6.4	401417
Incidence	Global	MDR-TB	Both	2002	6.9	6.7	422619
Incidence	Global	MDR-TB	Both	2003	7.0	6.9	441808
Incidence	Global	MDR-TB	Both	2004	7.2	7.1	457015
Incidence	Global	MDR-TB	Both	2005	7.2	7.1	465872

Incidence	Global	MDR-TB	Both	2006	7.1	7.0	467664
Incidence	Global	MDR-TB	Both	2007	6.9	6.9	464338
Incidence	Global	MDR-TB	Both	2008	6.7	6.7	458138
Incidence	Global	MDR-TB	Both	2009	6.5	6.5	451604
Incidence	Global	MDR-TB	Both	2010	6.3	6.4	447572
Incidence	Global	MDR-TB	Both	2011	6.2	6.3	442499
Incidence	Global	MDR-TB	Both	2012	5.9	6.0	432982
Incidence	Global	MDR-TB	Both	2013	5.7	5.8	422593
Incidence	Global	MDR-TB	Both	2014	5.5	5.7	415028
Incidence	Global	MDR-TB	Both	2015	5.5	5.6	414191
Incidence	Global	MDR-TB	Both	2016	5.5	5.6	421723
Incidence	Global	MDR-TB	Both	2017	5.5	5.7	431310
Incidence	Global	MDR-TB	Both	2018	5.6	5.7	440306
Incidence	Global	MDR-TB	Both	2019	5.6	5.8	450597
Incidence	Global	MDR-TB	Both	2020	5.3	5.5	433986
Incidence	Global	MDR-TB	Both	2021	5.2	5.4	433358
Incidence	Global	MDR-TB	Both	2022	5.2	5.4	432491
Incidence	Global	MDR-TB	Both	2023	5.1	5.3	433926
Incidence	Global	MDR-TB	Both	2024	5.1	5.3	435212
Incidence	Global	MDR-TB	Both	2025	5.0	5.3	436339
Incidence	Global	MDR-TB	Both	2026	5.0	5.2	437305
Incidence	Global	MDR-TB	Both	2027	4.9	5.2	438123
Incidence	Global	MDR-TB	Both	2028	4.9	5.2	438795
Incidence	Global	MDR-TB	Both	2029	4.9	5.1	439331
Incidence	Global	MDR-TB	Both	2030	4.8	5.1	439712
Incidence	High SDI	MDR-TB	Male	1990	0.3	0.3	1327
Incidence	High SDI	MDR-TB	Male	1991	0.4	0.4	1730
Incidence	High SDI	MDR-TB	Male	1992	0.5	0.5	2104
Incidence	High SDI	MDR-TB	Male	1993	0.5	0.6	2425
Incidence	High SDI	MDR-TB	Male	1994	0.6	0.6	2668
Incidence	High SDI	MDR-TB	Male	1995	0.6	0.7	2805
Incidence	High SDI	MDR-TB	Male	1996	0.6	0.7	2859
Incidence	High SDI	MDR-TB	Male	1997	0.6	0.7	2882
Incidence	High SDI	MDR-TB	Male	1998	0.6	0.7	2886
Incidence	High SDI	MDR-TB	Male	1999	0.6	0.7	2886
Incidence	High SDI	MDR-TB	Male	2000	0.6	0.7	2895
Incidence	High SDI	MDR-TB	Male	2001	0.6	0.7	2915
Incidence	High SDI	MDR-TB	Male	2002	0.6	0.7	2932
Incidence	High SDI	MDR-TB	Male	2003	0.6	0.7	2940
Incidence	High SDI	MDR-TB	Male	2004	0.6	0.7	2938
Incidence	High SDI	MDR-TB	Male	2005	0.6	0.6	2921
Incidence	High SDI	MDR-TB	Male	2006	0.5	0.6	2817
Incidence	High SDI	MDR-TB	Male	2007	0.5	0.6	2597
Incidence	High SDI	MDR-TB	Male	2008	0.4	0.5	2323
Incidence	High SDI	MDR-TB	Male	2009	0.4	0.4	2070
Incidence	High SDI	MDR-TB	Male	2010	0.3	0.4	1916
Incidence	High SDI	MDR-TB	Male	2011	0.3	0.4	1843
Incidence	High SDI	MDR-TB	Male	2012	0.3	0.4	1777
Incidence	High SDI	MDR-TB	Male	2013	0.3	0.4	1720
Incidence	High SDI	MDR-TB	Male	2014	0.3	0.3	1679
Incidence	High SDI	MDR-TB	Male	2015	0.3	0.3	1657

Incidence	High SDI	MDR-TB	Male	2016	0.3	0.3	1652
Incidence	High SDI	MDR-TB	Male	2017	0.3	0.3	1652
Incidence	High SDI	MDR-TB	Male	2018	0.3	0.3	1643
Incidence	High SDI	MDR-TB	Male	2019	0.3	0.3	1627
Incidence	High SDI	MDR-TB	Male	2020	0.3	0.3	1450
Incidence	High SDI	MDR-TB	Male	2021	0.3	0.3	1420
Incidence	High SDI	MDR-TB	Male	2022	0.2	0.3	1389
Incidence	High SDI	MDR-TB	Male	2023	0.2	0.3	1376
Incidence	High SDI	MDR-TB	Male	2024	0.2	0.3	1362
Incidence	High SDI	MDR-TB	Male	2025	0.2	0.3	1348
Incidence	High SDI	MDR-TB	Male	2026	0.2	0.3	1332
Incidence	High SDI	MDR-TB	Male	2027	0.2	0.3	1316
Incidence	High SDI	MDR-TB	Male	2028	0.2	0.3	1299
Incidence	High SDI	MDR-TB	Male	2029	0.2	0.3	1281
Incidence	High SDI	MDR-TB	Male	2030	0.2	0.3	1262
Incidence	High SDI	MDR-TB	Female	1990	0.2	0.2	1006
Incidence	High SDI	MDR-TB	Female	1991	0.3	0.3	1287
Incidence	High SDI	MDR-TB	Female	1992	0.3	0.4	1543
Incidence	High SDI	MDR-TB	Female	1993	0.4	0.4	1759
Incidence	High SDI	MDR-TB	Female	1994	0.4	0.4	1921
Incidence	High SDI	MDR-TB	Female	1995	0.4	0.5	2012
Incidence	High SDI	MDR-TB	Female	1996	0.4	0.5	2044
Incidence	High SDI	MDR-TB	Female	1997	0.4	0.5	2048
Incidence	High SDI	MDR-TB	Female	1998	0.4	0.5	2036
Incidence	High SDI	MDR-TB	Female	1999	0.4	0.5	2022
Incidence	High SDI	MDR-TB	Female	2000	0.4	0.5	2020
Incidence	High SDI	MDR-TB	Female	2001	0.4	0.5	2026
Incidence	High SDI	MDR-TB	Female	2002	0.4	0.4	2023
Incidence	High SDI	MDR-TB	Female	2003	0.4	0.4	2011
Incidence	High SDI	MDR-TB	Female	2004	0.4	0.4	1987
Incidence	High SDI	MDR-TB	Female	2005	0.4	0.4	1953
Incidence	High SDI	MDR-TB	Female	2006	0.4	0.4	1868
Incidence	High SDI	MDR-TB	Female	2007	0.3	0.4	1712
Incidence	High SDI	MDR-TB	Female	2008	0.3	0.3	1527
Incidence	High SDI	MDR-TB	Female	2009	0.3	0.3	1358
Incidence	High SDI	MDR-TB	Female	2010	0.2	0.3	1255
Incidence	High SDI	MDR-TB	Female	2011	0.2	0.2	1202
Incidence	High SDI	MDR-TB	Female	2012	0.2	0.2	1147
Incidence	High SDI	MDR-TB	Female	2013	0.2	0.2	1097
Incidence	High SDI	MDR-TB	Female	2014	0.2	0.2	1057
Incidence	High SDI	MDR-TB	Female	2015	0.2	0.2	1036
Incidence	High SDI	MDR-TB	Female	2016	0.2	0.2	1034
Incidence	High SDI	MDR-TB	Female	2017	0.2	0.2	1038
Incidence	High SDI	MDR-TB	Female	2018	0.2	0.2	1032
Incidence	High SDI	MDR-TB	Female	2019	0.2	0.2	1018
Incidence	High SDI	MDR-TB	Female	2020	0.2	0.2	904
Incidence	High SDI	MDR-TB	Female	2021	0.2	0.2	881
Incidence	High SDI	MDR-TB	Female	2022	0.2	0.2	857
Incidence	High SDI	MDR-TB	Female	2023	0.2	0.2	844
Incidence	High SDI	MDR-TB	Female	2024	0.2	0.2	830
Incidence	High SDI	MDR-TB	Female	2025	0.2	0.2	816

Incidence	High SDI	MDR-TB	Female	2026	0.1	0.2	803
Incidence	High SDI	MDR-TB	Female	2027	0.1	0.2	788
Incidence	High SDI	MDR-TB	Female	2028	0.1	0.2	773
Incidence	High SDI	MDR-TB	Female	2029	0.1	0.2	758
Incidence	High SDI	MDR-TB	Female	2030	0.1	0.2	743
Incidence	High SDI	MDR-TB	Both	1990	0.3	0.3	2333
Incidence	High SDI	MDR-TB	Both	1991	0.3	0.4	3017
Incidence	High SDI	MDR-TB	Both	1992	0.4	0.4	3647
Incidence	High SDI	MDR-TB	Both	1993	0.4	0.5	4184
Incidence	High SDI	MDR-TB	Both	1994	0.5	0.5	4589
Incidence	High SDI	MDR-TB	Both	1995	0.5	0.6	4817
Incidence	High SDI	MDR-TB	Both	1996	0.5	0.6	4903
Incidence	High SDI	MDR-TB	Both	1997	0.5	0.6	4930
Incidence	High SDI	MDR-TB	Both	1998	0.5	0.6	4922
Incidence	High SDI	MDR-TB	Both	1999	0.5	0.6	4908
Incidence	High SDI	MDR-TB	Both	2000	0.5	0.6	4916
Incidence	High SDI	MDR-TB	Both	2001	0.5	0.6	4940
Incidence	High SDI	MDR-TB	Both	2002	0.5	0.6	4955
Incidence	High SDI	MDR-TB	Both	2003	0.5	0.5	4951
Incidence	High SDI	MDR-TB	Both	2004	0.5	0.5	4926
Incidence	High SDI	MDR-TB	Both	2005	0.5	0.5	4874
Incidence	High SDI	MDR-TB	Both	2006	0.4	0.5	4685
Incidence	High SDI	MDR-TB	Both	2007	0.4	0.5	4309
Incidence	High SDI	MDR-TB	Both	2008	0.4	0.4	3850
Incidence	High SDI	MDR-TB	Both	2009	0.3	0.4	3428
Incidence	High SDI	MDR-TB	Both	2010	0.3	0.3	3171
Incidence	High SDI	MDR-TB	Both	2011	0.3	0.3	3045
Incidence	High SDI	MDR-TB	Both	2012	0.3	0.3	2925
Incidence	High SDI	MDR-TB	Both	2013	0.3	0.3	2817
Incidence	High SDI	MDR-TB	Both	2014	0.2	0.3	2736
Incidence	High SDI	MDR-TB	Both	2015	0.2	0.3	2693
Incidence	High SDI	MDR-TB	Both	2016	0.2	0.3	2686
Incidence	High SDI	MDR-TB	Both	2017	0.2	0.3	2690
Incidence	High SDI	MDR-TB	Both	2018	0.2	0.3	2676
Incidence	High SDI	MDR-TB	Both	2019	0.2	0.3	2645
Incidence	High SDI	MDR-TB	Both	2020	0.2	0.2	2354
Incidence	High SDI	MDR-TB	Both	2021	0.2	0.2	2301
Incidence	High SDI	MDR-TB	Both	2022	0.2	0.2	2246
Incidence	High SDI	MDR-TB	Both	2023	0.2	0.2	2220
Incidence	High SDI	MDR-TB	Both	2024	0.2	0.2	2192
Incidence	High SDI	MDR-TB	Both	2025	0.2	0.2	2164
Incidence	High SDI	MDR-TB	Both	2026	0.2	0.2	2135
Incidence	High SDI	MDR-TB	Both	2027	0.2	0.2	2104
Incidence	High SDI	MDR-TB	Both	2028	0.2	0.2	2072
Incidence	High SDI	MDR-TB	Both	2029	0.2	0.2	2039
Incidence	High SDI	MDR-TB	Both	2030	0.2	0.2	2005
Incidence	High-middle SDI	MDR-TB	Male	1990	1.4	1.4	7957
Incidence	High-middle SDI	MDR-TB	Male	1991	2.7	2.7	15342
Incidence	High-middle SDI	MDR-TB	Male	1992	4.0	3.9	22705
Incidence	High-middle SDI	MDR-TB	Male	1993	5.1	5.0	29709
Incidence	High-middle SDI	MDR-TB	Male	1994	6.0	6.1	35996

Incidence	High-middle SDI	MDR-TB	Male	1995	6.8	6.9	41210
Incidence	High-middle SDI	MDR-TB	Male	1996	7.5	7.6	46046
Incidence	High-middle SDI	MDR-TB	Male	1997	8.1	8.4	51219
Incidence	High-middle SDI	MDR-TB	Male	1998	8.8	9.2	56439
Incidence	High-middle SDI	MDR-TB	Male	1999	9.4	9.9	61383
Incidence	High-middle SDI	MDR-TB	Male	2000	9.9	10.5	65715
Incidence	High-middle SDI	MDR-TB	Male	2001	10.4	11.1	70054
Incidence	High-middle SDI	MDR-TB	Male	2002	10.9	11.8	74703
Incidence	High-middle SDI	MDR-TB	Male	2003	11.3	12.3	78964
Incidence	High-middle SDI	MDR-TB	Male	2004	11.5	12.7	82134
Incidence	High-middle SDI	MDR-TB	Male	2005	11.5	12.8	83463
Incidence	High-middle SDI	MDR-TB	Male	2006	11.3	12.6	82875
Incidence	High-middle SDI	MDR-TB	Male	2007	10.9	12.3	81009
Incidence	High-middle SDI	MDR-TB	Male	2008	10.4	11.8	78409
Incidence	High-middle SDI	MDR-TB	Male	2009	9.9	11.3	75609
Incidence	High-middle SDI	MDR-TB	Male	2010	9.4	10.8	73207
Incidence	High-middle SDI	MDR-TB	Male	2011	8.9	10.3	70211
Incidence	High-middle SDI	MDR-TB	Male	2012	8.3	9.6	65797
Incidence	High-middle SDI	MDR-TB	Male	2013	7.6	8.8	61007
Incidence	High-middle SDI	MDR-TB	Male	2014	7.0	8.2	56859
Incidence	High-middle SDI	MDR-TB	Male	2015	6.7	7.8	54420
Incidence	High-middle SDI	MDR-TB	Male	2016	6.5	7.6	53510
Incidence	High-middle SDI	MDR-TB	Male	2017	6.4	7.5	53050
Incidence	High-middle SDI	MDR-TB	Male	2018	6.3	7.4	52722
Incidence	High-middle SDI	MDR-TB	Male	2019	6.2	7.3	52486
Incidence	High-middle SDI	MDR-TB	Male	2020	5.5	6.4	22895
Incidence	High-middle SDI	MDR-TB	Male	2021	5.2	6.0	21786
Incidence	High-middle SDI	MDR-TB	Male	2022	4.9	5.7	20660
Incidence	High-middle SDI	MDR-TB	Male	2023	4.8	5.5	20002
Incidence	High-middle SDI	MDR-TB	Male	2024	4.6	5.3	19331
Incidence	High-middle SDI	MDR-TB	Male	2025	4.4	5.1	18652
Incidence	High-middle SDI	MDR-TB	Male	2026	4.2	4.9	17968
Incidence	High-middle SDI	MDR-TB	Male	2027	4.1	4.7	17277
Incidence	High-middle SDI	MDR-TB	Male	2028	3.9	4.5	16579
Incidence	High-middle SDI	MDR-TB	Male	2029	3.7	4.3	15870
Incidence	High-middle SDI	MDR-TB	Male	2030	3.5	4.1	15152
Incidence	High-middle SDI	MDR-TB	Female	1990	1.0	1.0	5731
Incidence	High-middle SDI	MDR-TB	Female	1991	1.8	1.9	10867
Incidence	High-middle SDI	MDR-TB	Female	1992	2.6	2.7	15794
Incidence	High-middle SDI	MDR-TB	Female	1993	3.3	3.4	20277
Incidence	High-middle SDI	MDR-TB	Female	1994	3.9	4.0	24065
Incidence	High-middle SDI	MDR-TB	Female	1995	4.3	4.4	26892
Incidence	High-middle SDI	MDR-TB	Female	1996	4.6	4.8	29153
Incidence	High-middle SDI	MDR-TB	Female	1997	4.9	5.1	31393
Incidence	High-middle SDI	MDR-TB	Female	1998	5.1	5.4	33575
Incidence	High-middle SDI	MDR-TB	Female	1999	5.4	5.7	35630
Incidence	High-middle SDI	MDR-TB	Female	2000	5.6	5.9	37491
Incidence	High-middle SDI	MDR-TB	Female	2001	5.8	6.2	39321
Incidence	High-middle SDI	MDR-TB	Female	2002	6.0	6.4	41139
Incidence	High-middle SDI	MDR-TB	Female	2003	6.1	6.6	42683
Incidence	High-middle SDI	MDR-TB	Female	2004	6.2	6.7	43679

Incidence	High-middle SDI	MDR-TB	Female	2005	6.1	6.7	43839
Incidence	High-middle SDI	MDR-TB	Female	2006	6.0	6.5	43243
Incidence	High-middle SDI	MDR-TB	Female	2007	5.8	6.3	42234
Incidence	High-middle SDI	MDR-TB	Female	2008	5.6	6.1	41002
Incidence	High-middle SDI	MDR-TB	Female	2009	5.3	5.9	39753
Incidence	High-middle SDI	MDR-TB	Female	2010	5.2	5.7	38709
Incidence	High-middle SDI	MDR-TB	Female	2011	4.9	5.4	37142
Incidence	High-middle SDI	MDR-TB	Female	2012	4.5	5.0	34580
Incidence	High-middle SDI	MDR-TB	Female	2013	4.1	4.6	31702
Incidence	High-middle SDI	MDR-TB	Female	2014	3.8	4.2	29172
Incidence	High-middle SDI	MDR-TB	Female	2015	3.6	3.9	27658
Incidence	High-middle SDI	MDR-TB	Female	2016	3.5	3.8	26894
Incidence	High-middle SDI	MDR-TB	Female	2017	3.4	3.7	26419
Incidence	High-middle SDI	MDR-TB	Female	2018	3.4	3.7	26496
Incidence	High-middle SDI	MDR-TB	Female	2019	3.5	3.8	27061
Incidence	High-middle SDI	MDR-TB	Female	2020	3.0	3.2	11827
Incidence	High-middle SDI	MDR-TB	Female	2021	2.8	3.0	11181
Incidence	High-middle SDI	MDR-TB	Female	2022	2.6	2.8	10531
Incidence	High-middle SDI	MDR-TB	Female	2023	2.5	2.7	10145
Incidence	High-middle SDI	MDR-TB	Female	2024	2.4	2.6	9757
Incidence	High-middle SDI	MDR-TB	Female	2025	2.3	2.5	9368
Incidence	High-middle SDI	MDR-TB	Female	2026	2.2	2.4	8980
Incidence	High-middle SDI	MDR-TB	Female	2027	2.1	2.3	8590
Incidence	High-middle SDI	MDR-TB	Female	2028	2.0	2.1	8199
Incidence	High-middle SDI	MDR-TB	Female	2029	1.9	2.0	7805
Incidence	High-middle SDI	MDR-TB	Female	2030	1.8	1.9	7408
Incidence	High-middle SDI	MDR-TB	Both	1990	1.2	1.2	13688
Incidence	High-middle SDI	MDR-TB	Both	1991	2.2	2.3	26209
Incidence	High-middle SDI	MDR-TB	Both	1992	3.2	3.3	38500
Incidence	High-middle SDI	MDR-TB	Both	1993	4.1	4.2	49986
Incidence	High-middle SDI	MDR-TB	Both	1994	4.9	5.0	60061
Incidence	High-middle SDI	MDR-TB	Both	1995	5.5	5.7	68102
Incidence	High-middle SDI	MDR-TB	Both	1996	5.9	6.2	75200
Incidence	High-middle SDI	MDR-TB	Both	1997	6.4	6.7	82612
Incidence	High-middle SDI	MDR-TB	Both	1998	6.9	7.3	90013
Incidence	High-middle SDI	MDR-TB	Both	1999	7.3	7.8	97013
Incidence	High-middle SDI	MDR-TB	Both	2000	7.7	8.2	103206
Incidence	High-middle SDI	MDR-TB	Both	2001	8.0	8.6	109375
Incidence	High-middle SDI	MDR-TB	Both	2002	8.3	9.1	115842
Incidence	High-middle SDI	MDR-TB	Both	2003	8.6	9.4	121647
Incidence	High-middle SDI	MDR-TB	Both	2004	8.8	9.7	125813
Incidence	High-middle SDI	MDR-TB	Both	2005	8.8	9.7	127302
Incidence	High-middle SDI	MDR-TB	Both	2006	8.6	9.6	126117
Incidence	High-middle SDI	MDR-TB	Both	2007	8.3	9.3	123242
Incidence	High-middle SDI	MDR-TB	Both	2008	7.9	8.9	119411
Incidence	High-middle SDI	MDR-TB	Both	2009	7.6	8.6	115361
Incidence	High-middle SDI	MDR-TB	Both	2010	7.3	8.3	111916
Incidence	High-middle SDI	MDR-TB	Both	2011	6.9	7.9	107353
Incidence	High-middle SDI	MDR-TB	Both	2012	6.4	7.3	100377
Incidence	High-middle SDI	MDR-TB	Both	2013	5.8	6.7	92709
Incidence	High-middle SDI	MDR-TB	Both	2014	5.4	6.2	86031

Incidence	High-middle SDI	MDR-TB	Both	2015	5.1	5.9	82078
Incidence	High-middle SDI	MDR-TB	Both	2016	5.0	5.7	80404
Incidence	High-middle SDI	MDR-TB	Both	2017	4.9	5.6	79469
Incidence	High-middle SDI	MDR-TB	Both	2018	4.8	5.6	79218
Incidence	High-middle SDI	MDR-TB	Both	2019	4.8	5.6	79547
Incidence	High-middle SDI	MDR-TB	Both	2020	4.2	4.7	34722
Incidence	High-middle SDI	MDR-TB	Both	2021	4.0	4.5	32967
Incidence	High-middle SDI	MDR-TB	Both	2022	3.8	4.2	31191
Incidence	High-middle SDI	MDR-TB	Both	2023	3.6	4.1	30147
Incidence	High-middle SDI	MDR-TB	Both	2024	3.5	3.9	29088
Incidence	High-middle SDI	MDR-TB	Both	2025	3.4	3.8	28021
Incidence	High-middle SDI	MDR-TB	Both	2026	3.2	3.6	26948
Incidence	High-middle SDI	MDR-TB	Both	2027	3.1	3.5	25868
Incidence	High-middle SDI	MDR-TB	Both	2028	2.9	3.3	24777
Incidence	High-middle SDI	MDR-TB	Both	2029	2.8	3.2	23675
Incidence	High-middle SDI	MDR-TB	Both	2030	2.7	3.0	22560
Incidence	Middle SDI	MDR-TB	Male	1990	2.0	1.6	14156
Incidence	Middle SDI	MDR-TB	Male	1991	3.3	2.6	23399
Incidence	Middle SDI	MDR-TB	Male	1992	4.4	3.6	32209
Incidence	Middle SDI	MDR-TB	Male	1993	5.4	4.4	40020
Incidence	Middle SDI	MDR-TB	Male	1994	6.1	5.0	46259
Incidence	Middle SDI	MDR-TB	Male	1995	6.5	5.3	50309
Incidence	Middle SDI	MDR-TB	Male	1996	6.7	5.5	53074
Incidence	Middle SDI	MDR-TB	Male	1997	6.9	5.8	55835
Incidence	Middle SDI	MDR-TB	Male	1998	7.0	6.0	58559
Incidence	Middle SDI	MDR-TB	Male	1999	7.1	6.2	61202
Incidence	Middle SDI	MDR-TB	Male	2000	7.3	6.3	63662
Incidence	Middle SDI	MDR-TB	Male	2001	7.4	6.5	66196
Incidence	Middle SDI	MDR-TB	Male	2002	7.5	6.7	68872
Incidence	Middle SDI	MDR-TB	Male	2003	7.6	6.9	71359
Incidence	Middle SDI	MDR-TB	Male	2004	7.6	7.0	73307
Incidence	Middle SDI	MDR-TB	Male	2005	7.5	7.0	74251
Incidence	Middle SDI	MDR-TB	Male	2006	7.3	6.9	73922
Incidence	Middle SDI	MDR-TB	Male	2007	7.0	6.7	72542
Incidence	Middle SDI	MDR-TB	Male	2008	6.7	6.4	70520
Incidence	Middle SDI	MDR-TB	Male	2009	6.4	6.2	68371
Incidence	Middle SDI	MDR-TB	Male	2010	6.1	6.0	66674
Incidence	Middle SDI	MDR-TB	Male	2011	5.8	5.8	64831
Incidence	Middle SDI	MDR-TB	Male	2012	5.5	5.5	62249
Incidence	Middle SDI	MDR-TB	Male	2013	5.2	5.2	59610
Incidence	Middle SDI	MDR-TB	Male	2014	4.9	5.0	57654
Incidence	Middle SDI	MDR-TB	Male	2015	4.8	4.9	57158
Incidence	Middle SDI	MDR-TB	Male	2016	4.9	5.0	58533
Incidence	Middle SDI	MDR-TB	Male	2017	4.9	5.1	60328
Incidence	Middle SDI	MDR-TB	Male	2018	5.0	5.2	61847
Incidence	Middle SDI	MDR-TB	Male	2019	5.1	5.3	63525
Incidence	Middle SDI	MDR-TB	Male	2020	4.6	5.1	73625
Incidence	Middle SDI	MDR-TB	Male	2021	4.5	5.0	72940
Incidence	Middle SDI	MDR-TB	Male	2022	4.4	4.9	72154
Incidence	Middle SDI	MDR-TB	Male	2023	4.4	4.9	72074
Incidence	Middle SDI	MDR-TB	Male	2024	4.3	4.8	71939

Incidence	Middle SDI	MDR-TB	Male	2025	4.3	4.8	71747
Incidence	Middle SDI	MDR-TB	Male	2026	4.2	4.8	71501
Incidence	Middle SDI	MDR-TB	Male	2027	4.2	4.7	71211
Incidence	Middle SDI	MDR-TB	Male	2028	4.1	4.7	70877
Incidence	Middle SDI	MDR-TB	Male	2029	4.1	4.7	70500
Incidence	Middle SDI	MDR-TB	Male	2030	4.0	4.6	70077
Incidence	Middle SDI	MDR-TB	Female	1990	1.7	1.5	12772
Incidence	Middle SDI	MDR-TB	Female	1991	2.9	2.6	22178
Incidence	Middle SDI	MDR-TB	Female	1992	3.9	3.6	30991
Incidence	Middle SDI	MDR-TB	Female	1993	4.8	4.4	38671
Incidence	Middle SDI	MDR-TB	Female	1994	5.4	5.0	44671
Incidence	Middle SDI	MDR-TB	Female	1995	5.8	5.3	48419
Incidence	Middle SDI	MDR-TB	Female	1996	5.9	5.5	50832
Incidence	Middle SDI	MDR-TB	Female	1997	6.1	5.7	53181
Incidence	Middle SDI	MDR-TB	Female	1998	6.2	5.8	55399
Incidence	Middle SDI	MDR-TB	Female	1999	6.3	6.0	57395
Incidence	Middle SDI	MDR-TB	Female	2000	6.3	6.1	59037
Incidence	Middle SDI	MDR-TB	Female	2001	6.3	6.1	60153
Incidence	Middle SDI	MDR-TB	Female	2002	6.3	6.1	60745
Incidence	Middle SDI	MDR-TB	Female	2003	6.2	6.0	60937
Incidence	Middle SDI	MDR-TB	Female	2004	6.1	6.0	60813
Incidence	Middle SDI	MDR-TB	Female	2005	5.9	5.8	60397
Incidence	Middle SDI	MDR-TB	Female	2006	5.7	5.7	59434
Incidence	Middle SDI	MDR-TB	Female	2007	5.5	5.5	57823
Incidence	Middle SDI	MDR-TB	Female	2008	5.2	5.2	55857
Incidence	Middle SDI	MDR-TB	Female	2009	4.9	5.0	53895
Incidence	Middle SDI	MDR-TB	Female	2010	4.7	4.8	52348
Incidence	Middle SDI	MDR-TB	Female	2011	4.5	4.6	50622
Incidence	Middle SDI	MDR-TB	Female	2012	4.2	4.3	48215
Incidence	Middle SDI	MDR-TB	Female	2013	3.9	4.1	45736
Incidence	Middle SDI	MDR-TB	Female	2014	3.7	3.9	43832
Incidence	Middle SDI	MDR-TB	Female	2015	3.6	3.8	43188
Incidence	Middle SDI	MDR-TB	Female	2016	3.7	3.8	43871
Incidence	Middle SDI	MDR-TB	Female	2017	3.7	3.8	44908
Incidence	Middle SDI	MDR-TB	Female	2018	3.8	3.9	46166
Incidence	Middle SDI	MDR-TB	Female	2019	3.9	4.0	47876
Incidence	Middle SDI	MDR-TB	Female	2020	3.4	3.6	51172
Incidence	Middle SDI	MDR-TB	Female	2021	3.3	3.5	49821
Incidence	Middle SDI	MDR-TB	Female	2022	3.1	3.3	48410
Incidence	Middle SDI	MDR-TB	Female	2023	3.1	3.3	47706
Incidence	Middle SDI	MDR-TB	Female	2024	3.0	3.2	46970
Incidence	Middle SDI	MDR-TB	Female	2025	2.9	3.1	46201
Incidence	Middle SDI	MDR-TB	Female	2026	2.8	3.1	45405
Incidence	Middle SDI	MDR-TB	Female	2027	2.8	3.0	44591
Incidence	Middle SDI	MDR-TB	Female	2028	2.7	2.9	43754
Incidence	Middle SDI	MDR-TB	Female	2029	2.6	2.9	42895
Incidence	Middle SDI	MDR-TB	Female	2030	2.6	2.8	42014
Incidence	Middle SDI	MDR-TB	Both	1990	1.9	1.6	26927
Incidence	Middle SDI	MDR-TB	Both	1991	3.1	2.6	45577
Incidence	Middle SDI	MDR-TB	Both	1992	4.1	3.6	63200
Incidence	Middle SDI	MDR-TB	Both	1993	5.1	4.4	78691

Incidence	Middle SDI	MDR-TB	Both	1994	5.7	5.0	90930
Incidence	Middle SDI	MDR-TB	Both	1995	6.1	5.3	98728
Incidence	Middle SDI	MDR-TB	Both	1996	6.3	5.5	103906
Incidence	Middle SDI	MDR-TB	Both	1997	6.4	5.7	109016
Incidence	Middle SDI	MDR-TB	Both	1998	6.6	5.9	113958
Incidence	Middle SDI	MDR-TB	Both	1999	6.7	6.1	118597
Incidence	Middle SDI	MDR-TB	Both	2000	6.7	6.2	122699
Incidence	Middle SDI	MDR-TB	Both	2001	6.8	6.3	126349
Incidence	Middle SDI	MDR-TB	Both	2002	6.8	6.4	129617
Incidence	Middle SDI	MDR-TB	Both	2003	6.8	6.5	132296
Incidence	Middle SDI	MDR-TB	Both	2004	6.8	6.5	134120
Incidence	Middle SDI	MDR-TB	Both	2005	6.7	6.4	134647
Incidence	Middle SDI	MDR-TB	Both	2006	6.5	6.3	133356
Incidence	Middle SDI	MDR-TB	Both	2007	6.2	6.1	130365
Incidence	Middle SDI	MDR-TB	Both	2008	5.9	5.8	126377
Incidence	Middle SDI	MDR-TB	Both	2009	5.6	5.6	122266
Incidence	Middle SDI	MDR-TB	Both	2010	5.4	5.4	119022
Incidence	Middle SDI	MDR-TB	Both	2011	5.1	5.2	115453
Incidence	Middle SDI	MDR-TB	Both	2012	4.8	4.9	110464
Incidence	Middle SDI	MDR-TB	Both	2013	4.5	4.6	105346
Incidence	Middle SDI	MDR-TB	Both	2014	4.3	4.4	101486
Incidence	Middle SDI	MDR-TB	Both	2015	4.2	4.3	100345
Incidence	Middle SDI	MDR-TB	Both	2016	4.2	4.4	102404
Incidence	Middle SDI	MDR-TB	Both	2017	4.3	4.5	105236
Incidence	Middle SDI	MDR-TB	Both	2018	4.4	4.5	108013
Incidence	Middle SDI	MDR-TB	Both	2019	4.4	4.6	111401
Incidence	Middle SDI	MDR-TB	Both	2020	4.0	4.3	124797
Incidence	Middle SDI	MDR-TB	Both	2021	3.9	4.2	122760
Incidence	Middle SDI	MDR-TB	Both	2022	3.8	4.1	120565
Incidence	Middle SDI	MDR-TB	Both	2023	3.7	4.1	119779
Incidence	Middle SDI	MDR-TB	Both	2024	3.6	4.0	118908
Incidence	Middle SDI	MDR-TB	Both	2025	3.6	4.0	117948
Incidence	Middle SDI	MDR-TB	Both	2026	3.5	3.9	116905
Incidence	Middle SDI	MDR-TB	Both	2027	3.5	3.9	115803
Incidence	Middle SDI	MDR-TB	Both	2028	3.4	3.8	114630
Incidence	Middle SDI	MDR-TB	Both	2029	3.3	3.8	113395
Incidence	Middle SDI	MDR-TB	Both	2030	3.3	3.7	112091
Incidence	Low-middle SDI	MDR-TB	Male	1990	0.7	0.5	3118
Incidence	Low-middle SDI	MDR-TB	Male	1991	1.5	1.1	6672
Incidence	Low-middle SDI	MDR-TB	Male	1992	2.3	1.8	10622
Incidence	Low-middle SDI	MDR-TB	Male	1993	3.2	2.4	14788
Incidence	Low-middle SDI	MDR-TB	Male	1994	4.0	3.1	18977
Incidence	Low-middle SDI	MDR-TB	Male	1995	4.7	3.6	22976
Incidence	Low-middle SDI	MDR-TB	Male	1996	5.6	4.3	27769
Incidence	Low-middle SDI	MDR-TB	Male	1997	6.7	5.2	33965
Incidence	Low-middle SDI	MDR-TB	Male	1998	7.8	6.1	40752
Incidence	Low-middle SDI	MDR-TB	Male	1999	8.9	7.0	47276
Incidence	Low-middle SDI	MDR-TB	Male	2000	9.7	7.7	52594
Incidence	Low-middle SDI	MDR-TB	Male	2001	10.3	8.2	57228
Incidence	Low-middle SDI	MDR-TB	Male	2002	10.9	8.8	62072
Incidence	Low-middle SDI	MDR-TB	Male	2003	11.5	9.3	66724

Incidence	Low-middle SDI	MDR-TB	Male	2004	11.9	9.7	70750
Incidence	Low-middle SDI	MDR-TB	Male	2005	12.1	10.0	73625
Incidence	Low-middle SDI	MDR-TB	Male	2006	12.1	10.1	75406
Incidence	Low-middle SDI	MDR-TB	Male	2007	12.0	10.1	76605
Incidence	Low-middle SDI	MDR-TB	Male	2008	11.9	10.1	77449
Incidence	Low-middle SDI	MDR-TB	Male	2009	11.7	10.0	78217
Incidence	Low-middle SDI	MDR-TB	Male	2010	11.6	10.0	79207
Incidence	Low-middle SDI	MDR-TB	Male	2011	11.5	10.0	80089
Incidence	Low-middle SDI	MDR-TB	Male	2012	11.4	9.9	80539
Incidence	Low-middle SDI	MDR-TB	Male	2013	11.2	9.8	80896
Incidence	Low-middle SDI	MDR-TB	Male	2014	11.1	9.8	81514
Incidence	Low-middle SDI	MDR-TB	Male	2015	11.0	9.8	82797
Incidence	Low-middle SDI	MDR-TB	Male	2016	11.1	10.0	85650
Incidence	Low-middle SDI	MDR-TB	Male	2017	11.3	10.2	88770
Incidence	Low-middle SDI	MDR-TB	Male	2018	11.3	10.4	91038
Incidence	Low-middle SDI	MDR-TB	Male	2019	11.4	10.5	93113
Incidence	Low-middle SDI	MDR-TB	Male	2020	11.2	10.2	119068
Incidence	Low-middle SDI	MDR-TB	Male	2021	11.1	10.3	121348
Incidence	Low-middle SDI	MDR-TB	Male	2022	11.1	10.3	123642
Incidence	Low-middle SDI	MDR-TB	Male	2023	11.1	10.4	126059
Incidence	Low-middle SDI	MDR-TB	Male	2024	11.1	10.5	128520
Incidence	Low-middle SDI	MDR-TB	Male	2025	11.1	10.6	131006
Incidence	Low-middle SDI	MDR-TB	Male	2026	11.1	10.6	133502
Incidence	Low-middle SDI	MDR-TB	Male	2027	11.1	10.7	135996
Incidence	Low-middle SDI	MDR-TB	Male	2028	11.1	10.8	138508
Incidence	Low-middle SDI	MDR-TB	Male	2029	11.1	10.9	141052
Incidence	Low-middle SDI	MDR-TB	Male	2030	11.1	11.0	143602
Incidence	Low-middle SDI	MDR-TB	Female	1990	0.7	0.6	3132
Incidence	Low-middle SDI	MDR-TB	Female	1991	1.4	1.2	6941
Incidence	Low-middle SDI	MDR-TB	Female	1992	2.2	1.9	11081
Incidence	Low-middle SDI	MDR-TB	Female	1993	3.0	2.6	15367
Incidence	Low-middle SDI	MDR-TB	Female	1994	3.8	3.3	19603
Incidence	Low-middle SDI	MDR-TB	Female	1995	4.5	3.9	23578
Incidence	Low-middle SDI	MDR-TB	Female	1996	5.2	4.6	28297
Incidence	Low-middle SDI	MDR-TB	Female	1997	6.2	5.5	34378
Incidence	Low-middle SDI	MDR-TB	Female	1998	7.2	6.4	40972
Incidence	Low-middle SDI	MDR-TB	Female	1999	8.1	7.3	47160
Incidence	Low-middle SDI	MDR-TB	Female	2000	8.7	7.9	51936
Incidence	Low-middle SDI	MDR-TB	Female	2001	9.2	8.3	55657
Incidence	Low-middle SDI	MDR-TB	Female	2002	9.6	8.7	59259
Incidence	Low-middle SDI	MDR-TB	Female	2003	9.9	9.0	62563
Incidence	Low-middle SDI	MDR-TB	Female	2004	10.2	9.3	65367
Incidence	Low-middle SDI	MDR-TB	Female	2005	10.3	9.4	67404
Incidence	Low-middle SDI	MDR-TB	Female	2006	10.2	9.4	68533
Incidence	Low-middle SDI	MDR-TB	Female	2007	10.1	9.3	68966
Incidence	Low-middle SDI	MDR-TB	Female	2008	9.9	9.2	69034
Incidence	Low-middle SDI	MDR-TB	Female	2009	9.7	9.1	69120
Incidence	Low-middle SDI	MDR-TB	Female	2010	9.6	9.0	69627
Incidence	Low-middle SDI	MDR-TB	Female	2011	9.5	9.0	70417
Incidence	Low-middle SDI	MDR-TB	Female	2012	9.5	8.9	71133
Incidence	Low-middle SDI	MDR-TB	Female	2013	9.4	8.9	71880

Incidence	Low-middle SDI	MDR-TB	Female	2014	9.4	8.9	72751
Incidence	Low-middle SDI	MDR-TB	Female	2015	9.3	8.9	73837
Incidence	Low-middle SDI	MDR-TB	Female	2016	9.3	8.9	75323
Incidence	Low-middle SDI	MDR-TB	Female	2017	9.3	9.0	76836
Incidence	Low-middle SDI	MDR-TB	Female	2018	9.3	9.0	78310
Incidence	Low-middle SDI	MDR-TB	Female	2019	9.3	9.1	80030
Incidence	Low-middle SDI	MDR-TB	Female	2020	9.2	8.9	101948
Incidence	Low-middle SDI	MDR-TB	Female	2021	9.2	8.9	103458
Incidence	Low-middle SDI	MDR-TB	Female	2022	9.2	8.9	104975
Incidence	Low-middle SDI	MDR-TB	Female	2023	9.1	9.0	106567
Incidence	Low-middle SDI	MDR-TB	Female	2024	9.1	9.0	108173
Incidence	Low-middle SDI	MDR-TB	Female	2025	9.1	9.0	109784
Incidence	Low-middle SDI	MDR-TB	Female	2026	9.1	9.1	111393
Incidence	Low-middle SDI	MDR-TB	Female	2027	9.1	9.1	112996
Incidence	Low-middle SDI	MDR-TB	Female	2028	9.0	9.1	114610
Incidence	Low-middle SDI	MDR-TB	Female	2029	9.0	9.2	116240
Incidence	Low-middle SDI	MDR-TB	Female	2030	9.0	9.2	117871
Incidence	Low-middle SDI	MDR-TB	Both	1990	0.7	0.6	6250
Incidence	Low-middle SDI	MDR-TB	Both	1991	1.5	1.2	13614
Incidence	Low-middle SDI	MDR-TB	Both	1992	2.3	1.9	21703
Incidence	Low-middle SDI	MDR-TB	Both	1993	3.1	2.5	30155
Incidence	Low-middle SDI	MDR-TB	Both	1994	3.9	3.2	38579
Incidence	Low-middle SDI	MDR-TB	Both	1995	4.6	3.8	46554
Incidence	Low-middle SDI	MDR-TB	Both	1996	5.4	4.5	56066
Incidence	Low-middle SDI	MDR-TB	Both	1997	6.4	5.3	68344
Incidence	Low-middle SDI	MDR-TB	Both	1998	7.5	6.3	81725
Incidence	Low-middle SDI	MDR-TB	Both	1999	8.5	7.1	94436
Incidence	Low-middle SDI	MDR-TB	Both	2000	9.2	7.8	104530
Incidence	Low-middle SDI	MDR-TB	Both	2001	9.7	8.3	112884
Incidence	Low-middle SDI	MDR-TB	Both	2002	10.2	8.7	121331
Incidence	Low-middle SDI	MDR-TB	Both	2003	10.7	9.2	129287
Incidence	Low-middle SDI	MDR-TB	Both	2004	11.0	9.5	136116
Incidence	Low-middle SDI	MDR-TB	Both	2005	11.1	9.7	141029
Incidence	Low-middle SDI	MDR-TB	Both	2006	11.1	9.8	143940
Incidence	Low-middle SDI	MDR-TB	Both	2007	11.0	9.7	145572
Incidence	Low-middle SDI	MDR-TB	Both	2008	10.8	9.6	146483
Incidence	Low-middle SDI	MDR-TB	Both	2009	10.7	9.5	147336
Incidence	Low-middle SDI	MDR-TB	Both	2010	10.6	9.5	148834
Incidence	Low-middle SDI	MDR-TB	Both	2011	10.5	9.5	150506
Incidence	Low-middle SDI	MDR-TB	Both	2012	10.4	9.4	151673
Incidence	Low-middle SDI	MDR-TB	Both	2013	10.3	9.3	152775
Incidence	Low-middle SDI	MDR-TB	Both	2014	10.2	9.3	154265
Incidence	Low-middle SDI	MDR-TB	Both	2015	10.1	9.3	156635
Incidence	Low-middle SDI	MDR-TB	Both	2016	10.2	9.5	160974
Incidence	Low-middle SDI	MDR-TB	Both	2017	10.2	9.6	165606
Incidence	Low-middle SDI	MDR-TB	Both	2018	10.3	9.7	169348
Incidence	Low-middle SDI	MDR-TB	Both	2019	10.3	9.8	173142
Incidence	Low-middle SDI	MDR-TB	Both	2020	10.2	9.6	221016
Incidence	Low-middle SDI	MDR-TB	Both	2021	10.1	9.6	224806
Incidence	Low-middle SDI	MDR-TB	Both	2022	10.1	9.6	228617
Incidence	Low-middle SDI	MDR-TB	Both	2023	10.1	9.7	232626

Incidence	Low-middle SDI	MDR-TB	Both	2024	10.1	9.7	236693
Incidence	Low-middle SDI	MDR-TB	Both	2025	10.1	9.8	240790
Incidence	Low-middle SDI	MDR-TB	Both	2026	10.1	9.9	244895
Incidence	Low-middle SDI	MDR-TB	Both	2027	10.0	9.9	248992
Incidence	Low-middle SDI	MDR-TB	Both	2028	10.0	10.0	253118
Incidence	Low-middle SDI	MDR-TB	Both	2029	10.0	10.0	257291
Incidence	Low-middle SDI	MDR-TB	Both	2030	10.0	10.1	261473
Incidence	Low SDI	MDR-TB	Male	1990	0.6	0.5	1220
Incidence	Low SDI	MDR-TB	Male	1991	1.3	1.0	2610
Incidence	Low SDI	MDR-TB	Male	1992	2.0	1.5	4180
Incidence	Low SDI	MDR-TB	Male	1993	2.8	2.0	5886
Incidence	Low SDI	MDR-TB	Male	1994	3.6	2.6	7661
Incidence	Low SDI	MDR-TB	Male	1995	4.3	3.1	9424
Incidence	Low SDI	MDR-TB	Male	1996	5.2	3.7	11581
Incidence	Low SDI	MDR-TB	Male	1997	6.3	4.5	14346
Incidence	Low SDI	MDR-TB	Male	1998	7.4	5.3	17328
Incidence	Low SDI	MDR-TB	Male	1999	8.4	5.9	20105
Incidence	Low SDI	MDR-TB	Male	2000	9.1	6.4	22223
Incidence	Low SDI	MDR-TB	Male	2001	9.5	6.7	23865
Incidence	Low SDI	MDR-TB	Male	2002	9.9	7.0	25477
Incidence	Low SDI	MDR-TB	Male	2003	10.2	7.2	26971
Incidence	Low SDI	MDR-TB	Male	2004	10.5	7.3	28282
Incidence	Low SDI	MDR-TB	Male	2005	10.6	7.4	29342
Incidence	Low SDI	MDR-TB	Male	2006	10.6	7.4	30196
Incidence	Low SDI	MDR-TB	Male	2007	10.5	7.4	30969
Incidence	Low SDI	MDR-TB	Male	2008	10.5	7.4	31720
Incidence	Low SDI	MDR-TB	Male	2009	10.4	7.4	32502
Incidence	Low SDI	MDR-TB	Male	2010	10.4	7.4	33391
Incidence	Low SDI	MDR-TB	Male	2011	10.4	7.4	34359
Incidence	Low SDI	MDR-TB	Male	2012	10.4	7.4	35333
Incidence	Low SDI	MDR-TB	Male	2013	10.4	7.4	36338
Incidence	Low SDI	MDR-TB	Male	2014	10.4	7.5	37425
Incidence	Low SDI	MDR-TB	Male	2015	10.4	7.5	38658
Incidence	Low SDI	MDR-TB	Male	2016	10.6	7.7	40375
Incidence	Low SDI	MDR-TB	Male	2017	10.7	7.8	42210
Incidence	Low SDI	MDR-TB	Male	2018	10.8	7.9	43807
Incidence	Low SDI	MDR-TB	Male	2019	10.9	8.0	45421
Incidence	Low SDI	MDR-TB	Male	2020	10.9	8.0	39453
Incidence	Low SDI	MDR-TB	Male	2021	11.0	8.1	40944
Incidence	Low SDI	MDR-TB	Male	2022	11.1	8.2	42472
Incidence	Low SDI	MDR-TB	Male	2023	11.1	8.3	43980
Incidence	Low SDI	MDR-TB	Male	2024	11.2	8.4	45542
Incidence	Low SDI	MDR-TB	Male	2025	11.2	8.5	47155
Incidence	Low SDI	MDR-TB	Male	2026	11.3	8.6	48813
Incidence	Low SDI	MDR-TB	Male	2027	11.3	8.7	50507
Incidence	Low SDI	MDR-TB	Male	2028	11.4	8.8	52246
Incidence	Low SDI	MDR-TB	Male	2029	11.4	8.9	54039
Incidence	Low SDI	MDR-TB	Male	2030	11.5	9.0	55881
Incidence	Low SDI	MDR-TB	Female	1990	0.6	0.5	1308
Incidence	Low SDI	MDR-TB	Female	1991	1.2	1.0	2802
Incidence	Low SDI	MDR-TB	Female	1992	1.9	1.6	4461

Incidence	Low SDI	MDR-TB	Female	1993	2.6	2.2	6237
Incidence	Low SDI	MDR-TB	Female	1994	3.3	2.8	8061
Incidence	Low SDI	MDR-TB	Female	1995	4.0	3.3	9852
Incidence	Low SDI	MDR-TB	Female	1996	4.7	3.9	12011
Incidence	Low SDI	MDR-TB	Female	1997	5.7	4.7	14763
Incidence	Low SDI	MDR-TB	Female	1998	6.6	5.5	17713
Incidence	Low SDI	MDR-TB	Female	1999	7.5	6.2	20434
Incidence	Low SDI	MDR-TB	Female	2000	8.0	6.6	22462
Incidence	Low SDI	MDR-TB	Female	2001	8.3	6.8	23945
Incidence	Low SDI	MDR-TB	Female	2002	8.6	7.0	25339
Incidence	Low SDI	MDR-TB	Female	2003	8.8	7.2	26599
Incidence	Low SDI	MDR-TB	Female	2004	8.9	7.3	27700
Incidence	Low SDI	MDR-TB	Female	2005	9.0	7.3	28619
Incidence	Low SDI	MDR-TB	Female	2006	9.0	7.3	29312
Incidence	Low SDI	MDR-TB	Female	2007	8.9	7.2	29820
Incidence	Low SDI	MDR-TB	Female	2008	8.7	7.1	30236
Incidence	Low SDI	MDR-TB	Female	2009	8.6	7.0	30648
Incidence	Low SDI	MDR-TB	Female	2010	8.5	7.0	31175
Incidence	Low SDI	MDR-TB	Female	2011	8.4	6.9	31718
Incidence	Low SDI	MDR-TB	Female	2012	8.3	6.8	32143
Incidence	Low SDI	MDR-TB	Female	2013	8.2	6.7	32535
Incidence	Low SDI	MDR-TB	Female	2014	8.2	6.6	33009
Incidence	Low SDI	MDR-TB	Female	2015	8.1	6.6	33704
Incidence	Low SDI	MDR-TB	Female	2016	8.1	6.7	34799
Incidence	Low SDI	MDR-TB	Female	2017	8.1	6.7	36015
Incidence	Low SDI	MDR-TB	Female	2018	8.1	6.8	37159
Incidence	Low SDI	MDR-TB	Female	2019	8.2	6.8	38354
Incidence	Low SDI	MDR-TB	Female	2020	8.0	6.7	32800
Incidence	Low SDI	MDR-TB	Female	2021	8.0	6.7	33610
Incidence	Low SDI	MDR-TB	Female	2022	8.0	6.7	34425
Incidence	Low SDI	MDR-TB	Female	2023	7.9	6.7	35275
Incidence	Low SDI	MDR-TB	Female	2024	7.9	6.7	36140
Incidence	Low SDI	MDR-TB	Female	2025	7.9	6.7	37019
Incidence	Low SDI	MDR-TB	Female	2026	7.9	6.7	37908
Incidence	Low SDI	MDR-TB	Female	2027	7.8	6.7	38802
Incidence	Low SDI	MDR-TB	Female	2028	7.8	6.7	39704
Incidence	Low SDI	MDR-TB	Female	2029	7.8	6.7	40617
Incidence	Low SDI	MDR-TB	Female	2030	7.7	6.7	41538
Incidence	Low SDI	MDR-TB	Both	1990	0.6	0.5	2528
Incidence	Low SDI	MDR-TB	Both	1991	1.3	1.0	5412
Incidence	Low SDI	MDR-TB	Both	1992	2.0	1.6	8641
Incidence	Low SDI	MDR-TB	Both	1993	2.7	2.1	12123
Incidence	Low SDI	MDR-TB	Both	1994	3.4	2.7	15722
Incidence	Low SDI	MDR-TB	Both	1995	4.1	3.2	19276
Incidence	Low SDI	MDR-TB	Both	1996	4.9	3.8	23592
Incidence	Low SDI	MDR-TB	Both	1997	6.0	4.6	29108
Incidence	Low SDI	MDR-TB	Both	1998	7.0	5.4	35041
Incidence	Low SDI	MDR-TB	Both	1999	7.9	6.0	40539
Incidence	Low SDI	MDR-TB	Both	2000	8.5	6.5	44684
Incidence	Low SDI	MDR-TB	Both	2001	8.9	6.8	47810
Incidence	Low SDI	MDR-TB	Both	2002	9.2	7.0	50816

Incidence	Low SDI	MDR-TB	Both	2003	9.5	7.2	53570
Incidence	Low SDI	MDR-TB	Both	2004	9.7	7.3	55982
Incidence	Low SDI	MDR-TB	Both	2005	9.8	7.4	57961
Incidence	Low SDI	MDR-TB	Both	2006	9.8	7.4	59508
Incidence	Low SDI	MDR-TB	Both	2007	9.7	7.3	60789
Incidence	Low SDI	MDR-TB	Both	2008	9.6	7.3	61956
Incidence	Low SDI	MDR-TB	Both	2009	9.5	7.2	63151
Incidence	Low SDI	MDR-TB	Both	2010	9.4	7.2	64566
Incidence	Low SDI	MDR-TB	Both	2011	9.4	7.1	66077
Incidence	Low SDI	MDR-TB	Both	2012	9.4	7.1	67476
Incidence	Low SDI	MDR-TB	Both	2013	9.3	7.1	68873
Incidence	Low SDI	MDR-TB	Both	2014	9.3	7.0	70434
Incidence	Low SDI	MDR-TB	Both	2015	9.2	7.1	72361
Incidence	Low SDI	MDR-TB	Both	2016	9.3	7.2	75173
Incidence	Low SDI	MDR-TB	Both	2017	9.4	7.3	78225
Incidence	Low SDI	MDR-TB	Both	2018	9.4	7.4	80966
Incidence	Low SDI	MDR-TB	Both	2019	9.5	7.4	83775
Incidence	Low SDI	MDR-TB	Both	2020	9.4	7.4	72253
Incidence	Low SDI	MDR-TB	Both	2021	9.5	7.4	74553
Incidence	Low SDI	MDR-TB	Both	2022	9.5	7.5	76898
Incidence	Low SDI	MDR-TB	Both	2023	9.5	7.5	79255
Incidence	Low SDI	MDR-TB	Both	2024	9.5	7.6	81683
Incidence	Low SDI	MDR-TB	Both	2025	9.5	7.6	84175
Incidence	Low SDI	MDR-TB	Both	2026	9.5	7.7	86721
Incidence	Low SDI	MDR-TB	Both	2027	9.5	7.7	89309
Incidence	Low SDI	MDR-TB	Both	2028	9.5	7.7	91950
Incidence	Low SDI	MDR-TB	Both	2029	9.5	7.8	94655
Incidence	Low SDI	MDR-TB	Both	2030	9.5	7.8	97419

Table S1 The lexis diagram of incidence data for the age-period-cohort model

cause	measure	sex	location	age	1895 to 1899	1900 to 1904	1905 to 1909	1910 to 1915	1915 to 1919	1920 to 1924	1925 to 1929	1930 to 1934	1935 to 1939	1940 to 1944	1945 to 1949	1950 to 1954	1954 to 1959	1960 to 1964	1965 to 1969	1970 to 1974	1975 to 1979	1980 to 1984	1985 to 1989	1990 to 1994	1995 to 1999	2000 to 2004	2005 to 2009	2010 to 2014	2015 to 2019
MDR-TB	Incidence	Male	Global	0 to 4																				1.11 (0.64 to 1.89)	1.9 (1.23 to 2.97)	2.07 (1.34 to 3.14)	1.9 (1.26 to 2.68)	1.63 (1.05 to 2.38)	1.57 (0.98 to 2.39)
MDR-TB	Incidence	Male	Global	5 to 9																			0.61 (0.34 to 1.05)	1.14 (0.67 to 1.86)	1.33 (0.78 to 2.12)	1.24 (0.74 to 1.92)	1.08 (0.63 to 1.75)	1.06 (0.59 to 1.79)	
MDR-TB	Incidence	Male	Global	10 to 14																		0.74 (0.4 to 1.3)	1.52 (0.88 to 2.53)	1.95 (1.14 to 3.08)	1.91 (1.1 to 2.97)	1.68 (0.93 to 2.77)	1.62 (0.85 to 2.78)		
MDR-TB	Incidence	Male	Global	15 to 19																	1.91 (1.02 to 3.43)	3.92 (2.32 to 6.39)	5.45 (3.29 to 8.66)	5.49 (3.45 to 8.42)	4.96 (2.8 to 8.24)	4.97 (2.51 to 9.14)			
MDR-TB	Incidence	Male	Global	20 to 24																2.59 (1.32 to 4.75)	5.26 (3 to 8.44)	7.28 (4.2 to 11.36)	7.79 (4.59 to 12.12)	6.91 (3.79 to 11.71)	6.51 (3.22 to 12.14)				
MDR-TB	Incidence	Male	Global	25 to 29															2.29 (1.21 to 4.3)	5.32 (3 to 9.28)	8.07 (4.54 to 13.39)	8.98 (5.04 to 14.55)	8.09 (4.31 to 14.05)	7.38 (3.58 to 14.21)					
MDR-TB	Incidence	Male	Global	30 to 34														2.62 (1.38 to 4.68)	6.24 (3.43 to 10.82)	9.26 (5.09 to 15.04)	10.4 (5.8 to 16.33)	9.43 (4.83 to 16.8)	8.63 (4.05 to 16.95)						
MDR-TB	Incidence	Male	Global	35 to 39													3.02 (1.59 to 5.23)	6.8 (3.85 to 11.39)	9.48 (5.47 to 15.25)	10.25 (5.84 to 16.15)	9.44 (4.83 to 16.43)	8.89 (4.15 to 17.37)							
MDR-TB	Incidence	Male	Global	40 to 44												3.46 (1.86 to 6.05)	7.66 (4.51 to 12.71)	11.09 (6.58 to 17.15)	11.17 (6.56 to 17.07)	9.72 (5.15 to 16.62)	9.46 (4.5 to 18.27)								
MDR-TB	Incidence	Male	Global	45 to 49											3.46 (1.93 to 6.07)	7.78 (4.62 to 13.22)	11.06 (6.65 to 17.27)	11.74 (6.98 to 17.81)	9.35 (5.07 to 15.99)	8.68 (4.04 to 17.02)									
MDR-TB	Incidence	Male	Global	50 to 54										4.13 (2.25 to 7.53)	8.23 (4.79 to 14.08)	11.5 (6.9 to 18.24)	11.99 (7.15 to 18.51)	10.36 (5.49 to 17.75)	8.88 (4.12 to 17.22)										
MDR-TB	Incidence	Male	Global	55 to 59									5.57 (3.13 to 9.62)	10.81 (6.75 to 17.63)	13.06 (8.24 to 20.44)	13.51 (8.4 to 20.45)	11.58 (6.44 to 19.49)	10.86 (5.26 to 21.4)											
MDR-TB	Incidence	Male	Global	60 to 64								6.32 (3.4 to 11.2)	12.06 (7.33 to 19.49)	14.56 (8.94 to 22)	13.41 (8.36 to 20.2)	11.29 (6.16 to 20.03)	10.41 (4.92 to 21.19)												
MDR-TB	Incidence	Male	Global	65 to 69							7.35 (3.82 to 13.22)	13.33 (8.22 to 21.07)	15.84 (10.09 to 23.95)	14.81 (9.53 to 22.27)	11.11 (6.05 to 19.43)	9.9 (4.62 to 19.42)													
MDR-TB	Incidence	Male	Global	70 to 74						6.92 (3.71 to 12.38)	12 (7.72 to 18.66)	13.99 (9.28 to 20.83)	12.84 (8.43 to 18.26)	9.65 (5.35 to 15.94)	8.48 (3.94 to 16.34)														
MDR-TB	Incidence	Male	Global	75 to 79					6.42 (3.51 to 11.49)	11.4 (7.23 to 17.37)	12.96 (8.56 to 18.77)	11.89 (8.01 to 17)	8.97 (5.2 to 14.7)	8.18 (4.02 to 15.7)															
MDR-TB	Incidence	Male	Global	80 to 84				7.26 (4.37 to 12.17)	13.6 (9.5 to 19.71)	15.69 (11.27 to 21.67)	14.1 (10.56 to 18.77)	11.2 (7.21 to 17.2)	10.04 (5.37 to 18)																
MDR-TB	Incidence	Male	Global	85 to 89			7.24 (4.53 to 11.63)	13.25 (9.41 to 19.2)	16.01 (11.77 to 21.74)	14.5 (11.09 to 18.88)	11.82 (7.91 to 17.72)	11.17 (6.29 to 19.39)																	
MDR-TB	Incidence	Male	Global	90 to 94		5.74 (3.75 to 8.98)	11.16 (7.7 to 16.64)	13.37 (9.5 to 18.96)	12.7 (9.37 to 17.15)	10.86 (7.22 to 16.71)	11.27 (6.27 to 20.05)																		
MDR-TB	Incidence	Male	Global	95 plus	4.88 (3.04 to 7.49)	9.4 (5.99 to 14.44)	11.15 (7.32 to 16.46)	10.11 (6.81 to 14.47)	8.82 (5.69 to 13.41)	9.11 (5.17 to 15.48)																			
MDR-TB	Incidence	Female	Global	0 to 4																				1.47 (0.84 to 2.52)	2.69 (1.71 to 4.25)	3.11 (2.03 to 4.73)	2.95 (1.97 to 4.17)	2.47 (1.61 to 3.62)	2.25 (1.42 to 3.45)
MDR-TB	Incidence	Female	Global	5 to 9																			0.92 (0.49 to 1.63)	1.83 (1.03 to 3.13)	2.19 (1.27 to 3.55)	2.06 (1.23 to 3.2)	1.72 (1.01 to 2.79)	1.64 (0.91 to 2.83)	
MDR-TB	Incidence	Female	Global	10 to 14																		1.12 (0.59 to 2.03)	2.5 (1.38 to 4.31)	3.22 (1.84 to 5.22)	3.07 (1.78 to 4.85)	2.58 (1.43 to 4.24)	2.52 (1.28 to 4.42)		
MDR-TB	Incidence	Female	Global	15 to 19																	2.42 (1.25 to 4.4)	5.24 (2.94 to 9)	7.07 (4.06 to 11.77)	6.73 (4.03 to 10.54)	5.86 (3.27 to 9.72)	5.9 (2.95 to 10.65)			
MDR-TB	Incidence	Female	Global	20 to 24																3.1 (1.55 to 5.82)	6.37 (3.43 to 10.9)	8.31 (4.61 to 13.43)	8.38 (4.8 to 13.31)	7.21 (3.83 to 12.53)	6.71 (3.21 to 12.72)				
MDR-TB	Incidence	Female	Global	25 to 29															2.71 (1.38 to 5.13)	5.94 (3.24 to 10.5)	7.96 (4.36 to 13.4)	8.15 (4.41 to 13.42)	7.17 (3.65 to 12.71)	6.18 (2.78 to 12.26)					
MDR-TB	Incidence	Female	Global	30 to 34														2.53 (1.32 to 4.59)	5.8 (3.18 to 10.16)	7.67 (4.12 to 12.84)	7.9 (4.19 to 12.85)	7.18 (3.51 to 13.26)	6.31 (2.77 to 12.74)						
MDR-TB	Incidence	Female	Global	35 to 39													2.31 (1.17 to 4.15)	4.71 (2.59 to 8.2)	6.31 (3.49 to 10.56)	6.61 (3.65 to 10.7)	6.22 (3.02 to 11.24)	5.86 (2.53 to 12.13)							
MDR-TB	Incidence	Female	Global	40 to 44												2.33 (1.19 to 4.24)	4.73 (2.69 to 8.07)	6.29 (3.62 to 10.05)	6.33 (3.6 to 9.93)	5.78 (2.84 to 10.36)	5.73 (2.44 to 11.82)								
MDR-TB	Incidence	Female	Global	45 to 49											2.26 (1.19 to 4.15)	4.6 (2.61 to 8.03)	5.9 (3.42 to 9.54)	6.02 (3.42 to 9.38)	5.21 (2.58 to 9.48)	5.04 (2.06 to 10.62)									
MDR-TB	Incidence	Female	Global	50 to 54										2.43 (1.24 to 4.69)	4.75 (2.66 to 8.23)	5.92 (3.43 to 9.84)	5.91 (3.41 to 9.38)	5.44 (2.69 to 9.75)	4.94 (2 to 10.38)										
MDR-TB	Incidence	Female	Global	55 to 59									3.1 (1.65 to 5.6)	5.72 (3.41 to 9.78)	6.93 (4.16 to 11.27)	6.79 (4.12 to 10.56)	5.94 (3.16 to 10.66)	5.8 (2.58 to 12.14)											
MDR-TB	Incidence	Female	Global	60 to 64								3.54 (1.81 to 6.42)	6.54 (3.78 to 10.87)	7.87 (4.66 to 12.41)	7.35 (4.46 to 11.49)	6.21 (3.25 to 11.5)	5.94 (2.61 to 12.96)												
MDR-TB	Incidence	Female	Global	65 to 69							4.36 (2.2 to 8.07)	7.77 (4.64 to 12.6)	9.15 (5.55 to 14.3)	8.55 (5.26 to 13.39)	6.74 (3.56 to 12.17)	6.15 (2.72 to 12.69)													
MDR-TB	Incidence	Female	Global	70 to 74						4.48 (2.35 to 8.18)	7.44 (4.68 to 11.74)	8.5 (5.48 to 13)	7.92 (5.05 to 11.59)	6.39 (3.45 to 10.89)	5.72 (2.56 to 11.47)														
MDR-TB	Incidence	Female	Global	75 to 79					3.94 (2.1 to 7.24)	6.71 (4.18 to 10.3)	7.21 (4.71 to 10.61)	6.7 (4.4 to 9.76)	5.57 (3.15 to 9.42)	5.25 (2.52 to 10.26)															
MDR-TB	Incidence	Female	Global	80 to 84				3.82 (2.22 to 6.58)	6.93 (4.77 to 9.95)	7.54 (5.42 to 10.37)	6.65 (4.93 to 8.88)	5.65 (3.65 to 8.64)	5.41 (2.87 to 9.79)																
MDR-TB	Incidence	Female	Global	85 to 89			3.69 (2.26 to 6.17)	6.19 (4.39 to 8.71)	7.11 (5.23 to 9.45)	6.22 (4.74 to 8.04)	5.16 (3.53 to 7.61)	5.07 (2.88 to 8.77)																	
MDR-TB	Incidence	Female	Global	90 to 94		3.23 (1.97 to 5.26)	5.44 (3.77 to 7.78)	5.77 (4.15 to 7.9)	5.4 (4 to 7.19)	4.42 (3.04 to 6.58)	4.47 (2.59 to 7.59)																		
MDR-TB	Incidence	Female	Global	95 plus	2.71 (1.62 to 4.38)	4.4 (2.86 to 6.59)	4.59 (3.06 to 6.63)	3.99 (2.71 to 5.65)	3.4 (2.25 to 5.06)	3.4 (1.99 to 5.57)																			
MDR-TB	Incidence	Both	Global	0 to 4																				1.28 (0.74 to 2.18)	2.28 (1.46 to 3.58)	2.57 (1.68 to 3.9)	2.41 (1.61 to 3.4)	2.04 (1.32 to 2.98)	1.9 (1.19 to 2.9)
MDR-TB	Incidence	Both	Global	5 to 9																			0.76 (0.41 to 1.33)	1.48 (0.84 to 2.47)	1.75 (1.02 to 2.82)	1.63 (0.98 to 2.54)	1.39 (0.82 to 2.24)	1.34 (0.75 to 2.28)	
MDR-TB	Incidence	Both	Global	10 to 14																		0.93 (0.49 to 1.64)	1.99 (1.12 to 3.38)	2.56 (1.48 to 4.12)	2.47 (1.43 to 3.87)	2.12 (1.17 to 3.49)	2.05 (1.06 to 3.58)		
MDR-TB	Incidence	Both	Global	15 to 19																	2.16 (1.14 to 3.93)	4.57 (2.63 to 7.65)	6.24 (3.67 to 10.22)	6.1 (3.73 to 9.4)	5.4 (3.02 to 8.93)	5.42 (2.74 to 9.87)			
MDR-TB	Incidence	Both	Global	20 to 24																2.85 (1.44 to 5.28)	5.81 (3.22 to 9.67)	7.79 (4.41 to 12.37)	8.08 (4.68 to 12.71)	7.06 (3.83 to 12.17)	6.61 (3.24 to 12.4)				
MDR-TB	Incidence	Both	Global	25 to 29															2.5 (1.29 to 4.7)	5.63 (3.13 to 9.91)	8.02 (4.47 to 13.4)	8.56 (4.72 to 13.97)	7.63 (3.99 to 13.33)	6.78 (3.19 to 13.3)					
MDR-TB	Incidence	Both	Global	30 to 34														2.57 (1.36 to 4.65)	6.02 (3.31 to 10.51)	8.47 (4.62 to 13.91)	9.15 (5.02 to 14.57)	8.31 (4.16 to 15.03)	7.48 (3.4 to 14.87)						
MDR-TB	Incidence	Both	Global	35 to 39													2.67 (1.38 to 4.68)	5.77 (3.24 to 9.84)	7.91 (4.53 to 12.96)	8.45 (4.76 to 13.43)	7.84 (3.92 to 13.88)	7.39 (3.35 to 14.83)							
MDR-TB	Incidence	Both	Global	40 to 44												2.9 (1.54 to 5.15)	6.22 (3.62 to 10.4)	8.73 (5.12 to 13.68)	8.78 (5.1 to 13.49)	7.77 (4.02 to 13.46)	7.61 (3.47 to 15.09)								
MDR-TB	Incidence	Both	Global	45 to 49											2.87 (1.57 to 5.14)	6.22 (3.64 to 10.67)	8.52 (5.05 to 13.41)	8.91 (5.23 to 13.63)	7.29 (3.83 to 12.79)	6.87 (3.06 to 13.86)									
MDR-TB	Incidence	Both	Global	50 to 54										3.29 (1.75 to 6.09)	6.51 (3.75 to 11.13)	8.73 (5.17 to 14.07)	8.96 (5.29 to 13.97)	7.9 (4.1 to 13.71)	6.9 (3.08 to 13.77)										
MDR-TB	Incidence	Both	Global	55 to 59									4.34 (2.39 to 7.61)	8.26 (5.1 to 13.64)	10 (6.18 to 15.85)	10.13 (6.23 to 15.43)	8.73 (4.8 to 15)	8.3 (3.89 to 16.56)											
MDR-TB	Incidence	Both	Global	60 to 64								4.9 (2.59 to 8.8)	9.25 (5.53 to 15)	11.15 (6.77 to 17.08)	10.33 (6.4 to 15.79)	8.69 (4.69 to 15.62)	8.11 (3.75 to 16.95)												
MDR-TB	Incidence	Both	Global	65 to 69							5.76 (2.98 to 10.47)	10.42 (6.36 to 16.59)	12.35 (7.72 to 18.87)	11.54 (7.32 to 17.58)	8.85 (4.78 to 15.71)	7.95 (3.66 to 15.86)													
MDR-TB	Incidence	Both	Global	70 to 74						5.56 (2.95 to 10.04)	9.5 (6.05 to 14.85)	11.03 (7.26 to 16.65)	10.19 (6.67 to 14.62)	7.9 (4.35 to 13.2)	7.02 (3.21 to 13.89)														
MDR-TB	Incidence	Both	Global	75 to 79					4.97 (2.69 to 9)	8.68 (5.48 to 13.22)	9.67 (6.4 to 14.1)	8.98 (5.98 to 12.91)	7.08 (4.07 to 11.7)	6.56 (3.2 to 12.68)															
MDR-TB	Incidence	Both	Global	80 to 84				5.11 (3.03 to 8.64)	9.47 (6.58 to 13.6)	10.7 (7.7 to 14.74)	9.61 (7.18 to 12.8)	7.93 (5.11 to 12.1)	7.34 (3.93 to 13.23)																
MDR-TB	Incidence	Both	Global	85 to 89			4.87 (3.01 to 7.97)	8.55 (6.09 to 12.09)	10.14 (7.45 to 13.61)	9.09 (6.96 to 11.81)	7.53 (5.11 to 11.2)	7.33 (4.15 to 12.76)																	
MDR-TB	Incidence	Both	Global	90 to 94		3.94 (2.49 to 6.23)	7.09 (4.96 to 10.23)	7.97 (5.71 to 11.03)	7.56 (5.61 to 10.07)	6.34 (4.29 to 9.58)	6.56 (3.73 to 11.46)																		
MDR-TB	Incidence	Both	Global	95 plus	3.25 (1.99 to 5.13)	5.64 (3.66 to 8.48)	6.29 (4.2 to 9.12)	5.58 (3.8 to 7.94)	4.85 (3.18 to 7.27)	4.92 (2.87 to 8.24)																			

S.Figure 1A DALYs number of MDR-TB among HIV-negative individuals in 204 countries and territories, all ages, 2019

measure	location	sex	age	cause	metric	year	value	95% UI (lower)	95% UI (upper)
DALYs	India	Both	All ages	MDR-TB	Number	2019	1945258	315451	4920120
DALYs	Pakistan	Both	All ages	MDR-TB	Number	2019	321250	87661	819613
DALYs	Nigeria	Both	All ages	MDR-TB	Number	2019	162363	35619	462362
DALYs	Somalia	Both	All ages	MDR-TB	Number	2019	133605	30621	361783
DALYs	Democratic Republic of the Congo	Both	All ages	MDR-TB	Number	2019	112214	11085	403214
DALYs	Russian Federation	Both	All ages	MDR-TB	Number	2019	98537	49854	158056
DALYs	Mozambique	Both	All ages	MDR-TB	Number	2019	97602	25734	235851
DALYs	China	Both	All ages	MDR-TB	Number	2019	91492	16761	269144
DALYs	Ethiopia	Both	All ages	MDR-TB	Number	2019	83672	14745	266932
DALYs	Bangladesh	Both	All ages	MDR-TB	Number	2019	74667	16034	193613
DALYs	Philippines	Both	All ages	MDR-TB	Number	2019	59232	13482	154566
DALYs	Ukraine	Both	All ages	MDR-TB	Number	2019	58136	29632	89759
DALYs	Myanmar	Both	All ages	MDR-TB	Number	2019	56554	17134	138000
DALYs	United Republic of Tanzania	Both	All ages	MDR-TB	Number	2019	48699	6545	175441
DALYs	South Africa	Both	All ages	MDR-TB	Number	2019	46297	13668	113401
DALYs	Zimbabwe	Both	All ages	MDR-TB	Number	2019	45909	6033	140367
DALYs	Uganda	Both	All ages	MDR-TB	Number	2019	42957	7394	122871
DALYs	Viet Nam	Both	All ages	MDR-TB	Number	2019	39577	9751	98335
DALYs	Angola	Both	All ages	MDR-TB	Number	2019	35536	3496	119561
DALYs	Uzbekistan	Both	All ages	MDR-TB	Number	2019	34542	13823	58612
DALYs	Madagascar	Both	All ages	MDR-TB	Number	2019	31642	4889	105733
DALYs	Burundi	Both	All ages	MDR-TB	Number	2019	25664	3102	92054
DALYs	Nepal	Both	All ages	MDR-TB	Number	2019	23920	5251	62506
DALYs	Kenya	Both	All ages	MDR-TB	Number	2019	23557	5951	57921
DALYs	Zambia	Both	All ages	MDR-TB	Number	2019	23317	3560	77184
DALYs	Burkina Faso	Both	All ages	MDR-TB	Number	2019	22990	2954	71791
DALYs	Ghana	Both	All ages	MDR-TB	Number	2019	22581	2915	71404
DALYs	C么te d'Ivoire	Both	All ages	MDR-TB	Number	2019	22199	3063	75334
DALYs	Indonesia	Both	All ages	MDR-TB	Number	2019	21620	2062	89552
DALYs	Chad	Both	All ages	MDR-TB	Number	2019	20904	2597	69940
DALYs	Eritrea	Both	All ages	MDR-TB	Number	2019	16884	2121	59000
DALYs	South Sudan	Both	All ages	MDR-TB	Number	2019	16482	2052	46065
DALYs	Afghanistan	Both	All ages	MDR-TB	Number	2019	15555	2341	49967
DALYs	Niger	Both	All ages	MDR-TB	Number	2019	15491	1741	52392
DALYs	Mali	Both	All ages	MDR-TB	Number	2019	15156	1659	48959
DALYs	Cameroon	Both	All ages	MDR-TB	Number	2019	14492	1921	51693
DALYs	Malawi	Both	All ages	MDR-TB	Number	2019	14167	2179	43343
DALYs	Central African Republic	Both	All ages	MDR-TB	Number	2019	14157	1711	51222
DALYs	Brazil	Both	All ages	MDR-TB	Number	2019	11594	1847	32496
DALYs	Rwanda	Both	All ages	MDR-TB	Number	2019	10815	3174	26963

DALYs	Lesotho	Both	All ages	MDR-TB	Number	2019	10683	2842	25873
DALYs	Kazakhstan	Both	All ages	MDR-TB	Number	2019	10662	5292	17261
DALYs	Peru	Both	All ages	MDR-TB	Number	2019	10373	3757	21721
DALYs	Guinea	Both	All ages	MDR-TB	Number	2019	9237	1078	31647
DALYs	Democratic People's Republic of Korea	Both	All ages	MDR-TB	Number	2019	9159	2359	21967
DALYs	Thailand	Both	All ages	MDR-TB	Number	2019	8387	1853	23258
DALYs	Tajikistan	Both	All ages	MDR-TB	Number	2019	8179	3325	14733
DALYs	Azerbaijan	Both	All ages	MDR-TB	Number	2019	7418	2320	13836
DALYs	Senegal	Both	All ages	MDR-TB	Number	2019	7130	1675	18164
DALYs	Kyrgyzstan	Both	All ages	MDR-TB	Number	2019	6292	2427	10522
DALYs	Eswatini	Both	All ages	MDR-TB	Number	2019	5971	1287	13632
DALYs	Sierra Leone	Both	All ages	MDR-TB	Number	2019	5702	640	19846
DALYs	Morocco	Both	All ages	MDR-TB	Number	2019	5614	1196	15742
DALYs	Mexico	Both	All ages	MDR-TB	Number	2019	5606	968	17137
DALYs	Bolivia (Plurinational State of)	Both	All ages	MDR-TB	Number	2019	5469	800	17245
DALYs	Papua New Guinea	Both	All ages	MDR-TB	Number	2019	5262	1279	13251
DALYs	Belarus	Both	All ages	MDR-TB	Number	2019	5085	3145	7332
DALYs	Benin	Both	All ages	MDR-TB	Number	2019	5071	745	15841
DALYs	Turkmenistan	Both	All ages	MDR-TB	Number	2019	4905	1789	9448
DALYs	Togo	Both	All ages	MDR-TB	Number	2019	4689	603	14614
DALYs	Namibia	Both	All ages	MDR-TB	Number	2019	4310	1438	9310
DALYs	Egypt	Both	All ages	MDR-TB	Number	2019	3716	781	10381
DALYs	Congo	Both	All ages	MDR-TB	Number	2019	3690	377	14765
DALYs	Botswana	Both	All ages	MDR-TB	Number	2019	3641	544	11072
DALYs	Republic of Moldova	Both	All ages	MDR-TB	Number	2019	3471	2348	4721
DALYs	Cambodia	Both	All ages	MDR-TB	Number	2019	3344	366	12679
DALYs	Saudi Arabia	Both	All ages	MDR-TB	Number	2019	3118	535	10383
DALYs	Mongolia	Both	All ages	MDR-TB	Number	2019	2785	579	7131
DALYs	Ecuador	Both	All ages	MDR-TB	Number	2019	2735	440	8513
DALYs	Djibouti	Both	All ages	MDR-TB	Number	2019	2597	835	5866
DALYs	Colombia	Both	All ages	MDR-TB	Number	2019	2403	381	7219
DALYs	Iraq	Both	All ages	MDR-TB	Number	2019	2325	562	6196
DALYs	Turkey	Both	All ages	MDR-TB	Number	2019	2308	868	4958
DALYs	Romania	Both	All ages	MDR-TB	Number	2019	2045	648	4658
DALYs	Sudan	Both	All ages	MDR-TB	Number	2019	2045	200	8527
DALYs	Yemen	Both	All ages	MDR-TB	Number	2019	2031	319	6929
DALYs	Liberia	Both	All ages	MDR-TB	Number	2019	1873	204	7425
DALYs	Georgia	Both	All ages	MDR-TB	Number	2019	1830	947	3064
DALYs	Comoros	Both	All ages	MDR-TB	Number	2019	1702	209	5135
DALYs	Republic of Korea	Both	All ages	MDR-TB	Number	2019	1546	181	6148
DALYs	Gambia	Both	All ages	MDR-TB	Number	2019	1379	162	4887
DALYs	Lithuania	Both	All ages	MDR-TB	Number	2019	1311	645	2165
DALYs	Guinea-Bissau	Both	All ages	MDR-TB	Number	2019	1215	168	4303
DALYs	Gabon	Both	All ages	MDR-TB	Number	2019	1130	124	4183
DALYs	Malaysia	Both	All ages	MDR-TB	Number	2019	1075	226	3307

DALYs	Venezuela (Bolivarian Republic of)	Both	All ages	MDR-TB	Number	2019	1031	114	3684
DALYs	Honduras	Both	All ages	MDR-TB	Number	2019	955	125	3223
DALYs	Iran (Islamic Republic of)	Both	All ages	MDR-TB	Number	2019	949	229	2553
DALYs	Guatemala	Both	All ages	MDR-TB	Number	2019	941	115	3038
DALYs	Mauritania	Both	All ages	MDR-TB	Number	2019	855	85	2953
DALYs	Taiwan (Province of China)	Both	All ages	MDR-TB	Number	2019	821	87	3107
DALYs	Lao People's Democratic Republic	Both	All ages	MDR-TB	Number	2019	808	82	3308
DALYs	Japan	Both	All ages	MDR-TB	Number	2019	797	84	3287
DALYs	Argentina	Both	All ages	MDR-TB	Number	2019	711	82	2648
DALYs	Germany	Both	All ages	MDR-TB	Number	2019	678	240	1496
DALYs	Algeria	Both	All ages	MDR-TB	Number	2019	646	55	3009
DALYs	United States of America	Both	All ages	MDR-TB	Number	2019	640	205	1589
DALYs	Armenia	Both	All ages	MDR-TB	Number	2019	638	156	1361
DALYs	Paraguay	Both	All ages	MDR-TB	Number	2019	581	64	1991
DALYs	Haiti	Both	All ages	MDR-TB	Number	2019	551	48	2378
DALYs	France	Both	All ages	MDR-TB	Number	2019	533	153	1428
DALYs	Equatorial Guinea	Both	All ages	MDR-TB	Number	2019	466	46	1693
DALYs	Italy	Both	All ages	MDR-TB	Number	2019	389	131	889
DALYs	Dominican Republic	Both	All ages	MDR-TB	Number	2019	365	31	1793
DALYs	Panama	Both	All ages	MDR-TB	Number	2019	345	35	1350
DALYs	Bhutan	Both	All ages	MDR-TB	Number	2019	309	33	1026
DALYs	Latvia	Both	All ages	MDR-TB	Number	2019	277	112	543
DALYs	Chile	Both	All ages	MDR-TB	Number	2019	251	73	646
DALYs	United Arab Emirates	Both	All ages	MDR-TB	Number	2019	248	23	954
DALYs	Poland	Both	All ages	MDR-TB	Number	2019	246	75	631
DALYs	Sri Lanka	Both	All ages	MDR-TB	Number	2019	227	17	1000
DALYs	Estonia	Both	All ages	MDR-TB	Number	2019	213	102	364
DALYs	United Kingdom	Both	All ages	MDR-TB	Number	2019	213	67	491
DALYs	Nicaragua	Both	All ages	MDR-TB	Number	2019	204	30	649
DALYs	Syrian Arab Republic	Both	All ages	MDR-TB	Number	2019	184	23	712
DALYs	Spain	Both	All ages	MDR-TB	Number	2019	173	20	723
DALYs	Bulgaria	Both	All ages	MDR-TB	Number	2019	156	37	409
DALYs	Portugal	Both	All ages	MDR-TB	Number	2019	140	27	433
DALYs	Cabo Verde	Both	All ages	MDR-TB	Number	2019	137	15	439
DALYs	Greece	Both	All ages	MDR-TB	Number	2019	128	21	414
DALYs	Libya	Both	All ages	MDR-TB	Number	2019	120	11	471
DALYs	El Salvador	Both	All ages	MDR-TB	Number	2019	113	13	429
DALYs	Australia	Both	All ages	MDR-TB	Number	2019	111	35	253
DALYs	Timor-Leste	Both	All ages	MDR-TB	Number	2019	108	10	497
DALYs	Tunisia	Both	All ages	MDR-TB	Number	2019	102	25	301
DALYs	Jordan	Both	All ages	MDR-TB	Number	2019	79	13	256
DALYs	Canada	Both	All ages	MDR-TB	Number	2019	74	21	178
DALYs	Austria	Both	All ages	MDR-TB	Number	2019	68	21	158
DALYs	Sweden	Both	All ages	MDR-TB	Number	2019	67	20	164
DALYs	Netherlands	Both	All ages	MDR-TB	Number	2019	65	18	160

DALYs	Cuba	Both	All ages	MDR-TB	Number	2019	61	10	174
DALYs	Israel	Both	All ages	MDR-TB	Number	2019	59	17	146
DALYs	Costa Rica	Both	All ages	MDR-TB	Number	2019	59	7	216
DALYs	Finland	Both	All ages	MDR-TB	Number	2019	57	15	149
DALYs	Serbia	Both	All ages	MDR-TB	Number	2019	57	13	160
DALYs	Lebanon	Both	All ages	MDR-TB	Number	2019	55	6	230
DALYs	Sao Tome and Principe	Both	All ages	MDR-TB	Number	2019	54	6	180
DALYs	Belgium	Both	All ages	MDR-TB	Number	2019	54	15	152
DALYs	Switzerland	Both	All ages	MDR-TB	Number	2019	51	16	109
DALYs	Czechia	Both	All ages	MDR-TB	Number	2019	51	15	128
DALYs	Palestine	Both	All ages	MDR-TB	Number	2019	42	5	170
DALYs	Guyana	Both	All ages	MDR-TB	Number	2019	37	3	204
DALYs	Hungary	Both	All ages	MDR-TB	Number	2019	36	6	125
DALYs	Oman	Both	All ages	MDR-TB	Number	2019	33	6	102
DALYs	Kuwait	Both	All ages	MDR-TB	Number	2019	31	7	82
DALYs	Singapore	Both	All ages	MDR-TB	Number	2019	31	11	73
DALYs	Norway	Both	All ages	MDR-TB	Number	2019	31	7	79
DALYs	North Macedonia	Both	All ages	MDR-TB	Number	2019	28	7	71
DALYs	Bahrain	Both	All ages	MDR-TB	Number	2019	26	4	80
DALYs	Bosnia and Herzegovina	Both	All ages	MDR-TB	Number	2019	25	5	76
DALYs	Denmark	Both	All ages	MDR-TB	Number	2019	19	4	50
DALYs	Qatar	Both	All ages	MDR-TB	Number	2019	19	3	64
DALYs	Kiribati	Both	All ages	MDR-TB	Number	2019	17	1	72
DALYs	Ireland	Both	All ages	MDR-TB	Number	2019	17	4	51
DALYs	New Zealand	Both	All ages	MDR-TB	Number	2019	15	3	44
DALYs	Puerto Rico	Both	All ages	MDR-TB	Number	2019	14	2	48
DALYs	Solomon Islands	Both	All ages	MDR-TB	Number	2019	13	1	52
DALYs	Jamaica	Both	All ages	MDR-TB	Number	2019	12	2	44
DALYs	Slovakia	Both	All ages	MDR-TB	Number	2019	12	2	42
DALYs	Uruguay	Both	All ages	MDR-TB	Number	2019	11	2	34
DALYs	Bahamas	Both	All ages	MDR-TB	Number	2019	9	1	37
DALYs	Croatia	Both	All ages	MDR-TB	Number	2019	9	2	28
DALYs	Trinidad and Tobago	Both	All ages	MDR-TB	Number	2019	7	1	29
DALYs	Marshall Islands	Both	All ages	MDR-TB	Number	2019	7	1	23
DALYs	Belize	Both	All ages	MDR-TB	Number	2019	6	1	26
DALYs	Vanuatu	Both	All ages	MDR-TB	Number	2019	6	0	28
DALYs	Fiji	Both	All ages	MDR-TB	Number	2019	6	0	26
DALYs	Mauritius	Both	All ages	MDR-TB	Number	2019	6	1	18
DALYs	Albania	Both	All ages	MDR-TB	Number	2019	4	1	13
DALYs	Brunei Darussalam	Both	All ages	MDR-TB	Number	2019	4	1	14
DALYs	Maldives	Both	All ages	MDR-TB	Number	2019	4	0	17
DALYs	Cyprus	Both	All ages	MDR-TB	Number	2019	4	1	11
DALYs	Suriname	Both	All ages	MDR-TB	Number	2019	3	0	13
DALYs	Micronesia (Federated States of)	Both	All ages	MDR-TB	Number	2019	3	0	15
DALYs	Greenland	Both	All ages	MDR-TB	Number	2019	3	0	10

DALYs	Montenegro	Both	All ages	MDR-TB	Number	2019	3	0	9
DALYs	Luxembourg	Both	All ages	MDR-TB	Number	2019	2	0	7
DALYs	Saint Lucia	Both	All ages	MDR-TB	Number	2019	1	0	6
DALYs	Guam	Both	All ages	MDR-TB	Number	2019	1	0	5
DALYs	Dominica	Both	All ages	MDR-TB	Number	2019	1	0	6
DALYs	Saint Vincent and the Grenadines	Both	All ages	MDR-TB	Number	2019	1	0	4
DALYs	Tonga	Both	All ages	MDR-TB	Number	2019	1	0	4
DALYs	Northern Mariana Islands	Both	All ages	MDR-TB	Number	2019	1	0	4
DALYs	Samoa	Both	All ages	MDR-TB	Number	2019	1	0	3
DALYs	Slovenia	Both	All ages	MDR-TB	Number	2019	1	0	2
DALYs	Seychelles	Both	All ages	MDR-TB	Number	2019	1	0	3
DALYs	Iceland	Both	All ages	MDR-TB	Number	2019	0	0	2
DALYs	Malta	Both	All ages	MDR-TB	Number	2019	0	0	2
DALYs	Barbados	Both	All ages	MDR-TB	Number	2019	0	0	2
DALYs	Saint Kitts and Nevis	Both	All ages	MDR-TB	Number	2019	0	0	2
DALYs	Monaco	Both	All ages	MDR-TB	Number	2019	0	0	1
DALYs	Grenada	Both	All ages	MDR-TB	Number	2019	0	0	2
DALYs	United States Virgin Islands	Both	All ages	MDR-TB	Number	2019	0	0	1
DALYs	Tuvalu	Both	All ages	MDR-TB	Number	2019	0	0	1
DALYs	Antigua and Barbuda	Both	All ages	MDR-TB	Number	2019	0	0	1
DALYs	Palau	Both	All ages	MDR-TB	Number	2019	0	0	1
DALYs	Nauru	Both	All ages	MDR-TB	Number	2019	0	0	1
DALYs	American Samoa	Both	All ages	MDR-TB	Number	2019	0	0	1
DALYs	San Marino	Both	All ages	MDR-TB	Number	2019	0	0	0
DALYs	Cook Islands	Both	All ages	MDR-TB	Number	2019	0	0	0
DALYs	Bermuda	Both	All ages	MDR-TB	Number	2019	0	0	0
DALYs	Andorra	Both	All ages	MDR-TB	Number	2019	0	0	0
DALYs	Tokelau	Both	All ages	MDR-TB	Number	2019	0	0	0
DALYs	Niue	Both	All ages	MDR-TB	Number	2019	0	0	0

S.Figure 1B Age-standardised DALYs rate of MDR-TB among HIV-negative individuals in 204 countries and territories, 2019

measure	location	sex	age	cause	metric	year	value (per 100,000)	95% UI (lower)	95%UI (upper)
DALYs	Armenia	Both	Age-standardized	MDR-TB	Rate	2019	18.4	4.4	39.1
DALYs	Kiribati	Both	Age-standardized	MDR-TB	Rate	2019	17.9	1.5	75.6
DALYs	Azerbaijan	Both	Age-standardized	MDR-TB	Rate	2019	66.6	21.4	125.1
DALYs	Papua New Guinea	Both	Age-standardized	MDR-TB	Rate	2019	66.1	16.0	166.2
DALYs	Samoa	Both	Age-standardized	MDR-TB	Rate	2019	0.4	0.0	1.5
DALYs	Myanmar	Both	Age-standardized	MDR-TB	Rate	2019	110.1	33.3	266.3
DALYs	Solomon Islands	Both	Age-standardized	MDR-TB	Rate	2019	2.7	0.2	10.8
DALYs	Philippines	Both	Age-standardized	MDR-TB	Rate	2019	61.6	14.2	161.5
DALYs	Luxembourg	Both	Age-standardized	MDR-TB	Rate	2019	0.2	0.0	0.8
DALYs	Sri Lanka	Both	Age-standardized	MDR-TB	Rate	2019	0.9	0.1	4.1
DALYs	Argentina	Both	Age-standardized	MDR-TB	Rate	2019	1.5	0.2	5.5
DALYs	Republic of Moldova	Both	Age-standardized	MDR-TB	Rate	2019	76.0	51.5	103.8
DALYs	Slovenia	Both	Age-standardized	MDR-TB	Rate	2019	0.0	0.0	0.1
DALYs	Chile	Both	Age-standardized	MDR-TB	Rate	2019	1.1	0.3	2.9
DALYs	Russian Federation	Both	Age-standardized	MDR-TB	Rate	2019	55.5	28.1	89.0
DALYs	Japan	Both	Age-standardized	MDR-TB	Rate	2019	0.2	0.0	0.9
DALYs	Uruguay	Both	Age-standardized	MDR-TB	Rate	2019	0.3	0.0	0.8
DALYs	Republic of Korea	Both	Age-standardized	MDR-TB	Rate	2019	1.9	0.2	7.6
DALYs	Ukraine	Both	Age-standardized	MDR-TB	Rate	2019	106.9	54.6	166.0
DALYs	Cambodia	Both	Age-standardized	MDR-TB	Rate	2019	23.6	2.6	89.0
DALYs	Singapore	Both	Age-standardized	MDR-TB	Rate	2019	0.4	0.1	1.0
DALYs	Finland	Both	Age-standardized	MDR-TB	Rate	2019	0.5	0.1	1.3
DALYs	Indonesia	Both	Age-standardized	MDR-TB	Rate	2019	9.1	0.9	37.4
DALYs	Andorra	Both	Age-standardized	MDR-TB	Rate	2019	0.0	0.0	0.1
DALYs	Qatar	Both	Age-standardized	MDR-TB	Rate	2019	1.2	0.2	3.9
DALYs	France	Both	Age-standardized	MDR-TB	Rate	2019	0.4	0.1	1.1
DALYs	Austria	Both	Age-standardized	MDR-TB	Rate	2019	0.5	0.1	1.1
DALYs	Panama	Both	Age-standardized	MDR-TB	Rate	2019	8.3	0.8	32.6
DALYs	Israel	Both	Age-standardized	MDR-TB	Rate	2019	0.6	0.2	1.4
DALYs	Fiji	Both	Age-standardized	MDR-TB	Rate	2019	0.7	0.1	3.0
DALYs	Jordan	Both	Age-standardized	MDR-TB	Rate	2019	1.0	0.2	3.3
DALYs	Kuwait	Both	Age-standardized	MDR-TB	Rate	2019	0.9	0.2	2.5
DALYs	Italy	Both	Age-standardized	MDR-TB	Rate	2019	0.3	0.1	0.8
DALYs	Turkey	Both	Age-standardized	MDR-TB	Rate	2019	2.6	1.0	5.7
DALYs	Brunei Darussalam	Both	Age-standardized	MDR-TB	Rate	2019	1.3	0.2	4.5
DALYs	Belgium	Both	Age-standardized	MDR-TB	Rate	2019	0.3	0.1	0.8
DALYs	China	Both	Age-standardized	MDR-TB	Rate	2019	5.1	0.9	14.9
DALYs	United Arab Emirates	Both	Age-standardized	MDR-TB	Rate	2019	3.0	0.3	11.1
DALYs	Angola	Both	Age-standardized	MDR-TB	Rate	2019	165.3	16.4	558.3
DALYs	Portugal	Both	Age-standardized	MDR-TB	Rate	2019	0.8	0.2	2.4
DALYs	Antigua and Barbuda	Both	Age-standardized	MDR-TB	Rate	2019	0.2	0.0	1.0
DALYs	Cyprus	Both	Age-standardized	MDR-TB	Rate	2019	0.2	0.0	0.6
DALYs	Yemen	Both	Age-standardized	MDR-TB	Rate	2019	9.6	1.5	32.9
DALYs	Central African Republic	Both	Age-standardized	MDR-TB	Rate	2019	333.2	40.7	1205.9
DALYs	Spain	Both	Age-standardized	MDR-TB	Rate	2019	0.2	0.0	0.9
DALYs	Denmark	Both	Age-standardized	MDR-TB	Rate	2019	0.2	0.0	0.5
DALYs	Sweden	Both	Age-standardized	MDR-TB	Rate	2019	0.3	0.1	0.8
DALYs	Democratic People's Republic of Korea	Both	Age-standardized	MDR-TB	Rate	2019	29.8	7.8	71.3
DALYs	Lao People's Democratic Republic	Both	Age-standardized	MDR-TB	Rate	2019	13.9	1.4	55.7

DALYs	Algeria	Both	Age-standardized	MDR-TB	Rate	2019	1.7	0.1	8.3
DALYs	Thailand	Both	Age-standardized	MDR-TB	Rate	2019	8.8	1.9	24.4
DALYs	Taiwan (Province of China)	Both	Age-standardized	MDR-TB	Rate	2019	2.3	0.2	8.7
DALYs	Malta	Both	Age-standardized	MDR-TB	Rate	2019	0.1	0.0	0.2
DALYs	Netherlands	Both	Age-standardized	MDR-TB	Rate	2019	0.2	0.1	0.5
DALYs	Timor-Leste	Both	Age-standardized	MDR-TB	Rate	2019	10.7	1.0	50.1
DALYs	Canada	Both	Age-standardized	MDR-TB	Rate	2019	0.1	0.0	0.3
DALYs	Tonga	Both	Age-standardized	MDR-TB	Rate	2019	1.1	0.1	4.8
DALYs	Norway	Both	Age-standardized	MDR-TB	Rate	2019	0.3	0.1	0.8
DALYs	Viet Nam	Both	Age-standardized	MDR-TB	Rate	2019	41.4	10.4	102.3
DALYs	United States of America	Both	Age-standardized	MDR-TB	Rate	2019	0.1	0.0	0.3
DALYs	Romania	Both	Age-standardized	MDR-TB	Rate	2019	8.5	2.7	19.0
DALYs	Vanuatu	Both	Age-standardized	MDR-TB	Rate	2019	2.7	0.2	12.5
DALYs	Serbia	Both	Age-standardized	MDR-TB	Rate	2019	0.4	0.1	1.2
DALYs	Uzbekistan	Both	Age-standardized	MDR-TB	Rate	2019	106.3	42.1	180.5
DALYs	Belize	Both	Age-standardized	MDR-TB	Rate	2019	1.7	0.2	7.1
DALYs	Croatia	Both	Age-standardized	MDR-TB	Rate	2019	0.1	0.0	0.4
DALYs	Slovakia	Both	Age-standardized	MDR-TB	Rate	2019	0.1	0.0	0.5
DALYs	Czechia	Both	Age-standardized	MDR-TB	Rate	2019	0.3	0.1	0.7
DALYs	Hungary	Both	Age-standardized	MDR-TB	Rate	2019	0.2	0.0	0.7
DALYs	Lebanon	Both	Age-standardized	MDR-TB	Rate	2019	1.0	0.1	4.3
DALYs	Albania	Both	Age-standardized	MDR-TB	Rate	2019	0.1	0.0	0.4
DALYs	Libya	Both	Age-standardized	MDR-TB	Rate	2019	2.0	0.2	7.5
DALYs	Morocco	Both	Age-standardized	MDR-TB	Rate	2019	16.7	3.5	46.2
DALYs	Afghanistan	Both	Age-standardized	MDR-TB	Rate	2019	62.0	9.3	200.3
DALYs	Saudi Arabia	Both	Age-standardized	MDR-TB	Rate	2019	10.6	1.9	34.8
DALYs	Congo	Both	Age-standardized	MDR-TB	Rate	2019	93.3	9.5	381.8
DALYs	Syrian Arab Republic	Both	Age-standardized	MDR-TB	Rate	2019	1.4	0.2	5.3
DALYs	Estonia	Both	Age-standardized	MDR-TB	Rate	2019	12.2	5.8	20.9
DALYs	Democratic Republic of the Congo	Both	Age-standardized	MDR-TB	Rate	2019	182.4	18.4	659.9
DALYs	Nepal	Both	Age-standardized	MDR-TB	Rate	2019	94.7	20.7	247.8
DALYs	Pakistan	Both	Age-standardized	MDR-TB	Rate	2019	178.4	47.7	457.0
DALYs	Switzerland	Both	Age-standardized	MDR-TB	Rate	2019	0.3	0.1	0.7
DALYs	Cuba	Both	Age-standardized	MDR-TB	Rate	2019	0.4	0.1	1.1
DALYs	United Kingdom	Both	Age-standardized	MDR-TB	Rate	2019	0.2	0.1	0.5
DALYs	Greece	Both	Age-standardized	MDR-TB	Rate	2019	0.7	0.1	2.1
DALYs	Dominica	Both	Age-standardized	MDR-TB	Rate	2019	1.6	0.1	7.9
DALYs	Belarus	Both	Age-standardized	MDR-TB	Rate	2019	41.3	25.2	59.6
DALYs	Bahamas	Both	Age-standardized	MDR-TB	Rate	2019	2.3	0.3	9.0
DALYs	Iceland	Both	Age-standardized	MDR-TB	Rate	2019	0.1	0.0	0.3
DALYs	Barbados	Both	Age-standardized	MDR-TB	Rate	2019	0.1	0.0	0.6
DALYs	Ireland	Both	Age-standardized	MDR-TB	Rate	2019	0.2	0.1	0.7
DALYs	Mozambique	Both	Age-standardized	MDR-TB	Rate	2019	514.7	133.5	1240.8
DALYs	Germany	Both	Age-standardized	MDR-TB	Rate	2019	0.4	0.2	1.0
DALYs	Bolivia (Plurinational State of)	Both	Age-standardized	MDR-TB	Rate	2019	50.6	7.3	160.2
DALYs	Rwanda	Both	Age-standardized	MDR-TB	Rate	2019	117.5	34.1	293.7
DALYs	Zambia	Both	Age-standardized	MDR-TB	Rate	2019	194.0	28.7	638.1
DALYs	Seychelles	Both	Age-standardized	MDR-TB	Rate	2019	0.5	0.0	2.4
DALYs	Ecuador	Both	Age-standardized	MDR-TB	Rate	2019	16.2	2.6	50.4
DALYs	Eswatini	Both	Age-standardized	MDR-TB	Rate	2019	614.8	133.8	1402.0
DALYs	Bermuda	Both	Age-standardized	MDR-TB	Rate	2019	0.1	0.0	0.4
DALYs	Senegal	Both	Age-standardized	MDR-TB	Rate	2019	68.4	15.8	172.9
DALYs	Guinea	Both	Age-standardized	MDR-TB	Rate	2019	100.8	11.9	343.8
DALYs	Botswana	Both	Age-standardized	MDR-TB	Rate	2019	171.3	26.0	520.3
DALYs	Costa Rica	Both	Age-standardized	MDR-TB	Rate	2019	1.2	0.1	4.2

DALYs	Saint Kitts and Nevis	Both	Age-standardized	MDR-TB	Rate	2019	0.6	0.0	2.7
DALYs	El Salvador	Both	Age-standardized	MDR-TB	Rate	2019	1.9	0.2	7.1
DALYs	Mauritius	Both	Age-standardized	MDR-TB	Rate	2019	0.4	0.1	1.1
DALYs	Gambia	Both	Age-standardized	MDR-TB	Rate	2019	99.4	11.2	348.5
DALYs	Sierra Leone	Both	Age-standardized	MDR-TB	Rate	2019	89.5	10.4	309.3
DALYs	Cabo Verde	Both	Age-standardized	MDR-TB	Rate	2019	27.4	3.1	88.2
DALYs	Guam	Both	Age-standardized	MDR-TB	Rate	2019	0.7	0.1	2.8
DALYs	Dominican Republic	Both	Age-standardized	MDR-TB	Rate	2019	3.4	0.3	16.4
DALYs	Grenada	Both	Age-standardized	MDR-TB	Rate	2019	0.3	0.0	1.7
DALYs	Honduras	Both	Age-standardized	MDR-TB	Rate	2019	13.1	1.7	44.3
DALYs	Guyana	Both	Age-standardized	MDR-TB	Rate	2019	4.9	0.4	27.0
DALYs	Suriname	Both	Age-standardized	MDR-TB	Rate	2019	0.5	0.1	2.1
DALYs	Tokelau	Both	Age-standardized	MDR-TB	Rate	2019	1.1	0.1	5.9
DALYs	Tuvalu	Both	Age-standardized	MDR-TB	Rate	2019	2.2	0.2	9.2
DALYs	United States Virgin Islands	Both	Age-standardized	MDR-TB	Rate	2019	0.2	0.0	0.8
DALYs	Monaco	Both	Age-standardized	MDR-TB	Rate	2019	0.5	0.0	1.9
DALYs	South Sudan	Both	Age-standardized	MDR-TB	Rate	2019	242.9	30.5	684.8
DALYs	Nauru	Both	Age-standardized	MDR-TB	Rate	2019	2.3	0.2	11.0
DALYs	Comoros	Both	Age-standardized	MDR-TB	Rate	2019	284.8	34.6	851.6
DALYs	Niue	Both	Age-standardized	MDR-TB	Rate	2019	0.6	0.1	2.8
DALYs	Djibouti	Both	Age-standardized	MDR-TB	Rate	2019	273.2	87.6	605.3
DALYs	Latvia	Both	Age-standardized	MDR-TB	Rate	2019	10.1	4.2	20.0
DALYs	Eritrea	Both	Age-standardized	MDR-TB	Rate	2019	368.9	46.3	1271.1
DALYs	Lithuania	Both	Age-standardized	MDR-TB	Rate	2019	35.2	17.4	58.0
DALYs	Saint Vincent and the Grenadines	Both	Age-standardized	MDR-TB	Rate	2019	0.8	0.1	3.4
DALYs	Somalia	Both	Age-standardized	MDR-TB	Rate	2019	1010.9	230.5	2778.9
DALYs	Zimbabwe	Both	Age-standardized	MDR-TB	Rate	2019	399.3	51.4	1221.6
DALYs	United Republic of Tanzania	Both	Age-standardized	MDR-TB	Rate	2019	121.0	16.6	440.6
DALYs	Benin	Both	Age-standardized	MDR-TB	Rate	2019	57.0	8.3	183.9
DALYs	Cook Islands	Both	Age-standardized	MDR-TB	Rate	2019	0.3	0.0	1.3
DALYs	Uganda	Both	Age-standardized	MDR-TB	Rate	2019	168.5	28.5	481.9
DALYs	Togo	Both	Age-standardized	MDR-TB	Rate	2019	84.7	10.7	263.0
DALYs	Burkina Faso	Both	Age-standardized	MDR-TB	Rate	2019	129.1	17.1	414.7
DALYs	Chad	Both	Age-standardized	MDR-TB	Rate	2019	169.6	21.5	558.7
DALYs	Greenland	Both	Age-standardized	MDR-TB	Rate	2019	4.2	0.5	15.4
DALYs	Cameroon	Both	Age-standardized	MDR-TB	Rate	2019	72.7	9.6	262.6
DALYs	Mali	Both	Age-standardized	MDR-TB	Rate	2019	93.8	10.4	307.1
DALYs	Mongolia	Both	Age-standardized	MDR-TB	Rate	2019	77.8	16.2	200.3
DALYs	North Macedonia	Both	Age-standardized	MDR-TB	Rate	2019	1.0	0.2	2.4
DALYs	Trinidad and Tobago	Both	Age-standardized	MDR-TB	Rate	2019	0.4	0.0	1.7
DALYs	Mexico	Both	Age-standardized	MDR-TB	Rate	2019	4.4	0.8	13.6
DALYs	Montenegro	Both	Age-standardized	MDR-TB	Rate	2019	0.3	0.0	1.0
DALYs	Nicaragua	Both	Age-standardized	MDR-TB	Rate	2019	3.7	0.5	11.6
DALYs	Poland	Both	Age-standardized	MDR-TB	Rate	2019	0.4	0.1	1.1
DALYs	Bahrain	Both	Age-standardized	MDR-TB	Rate	2019	2.3	0.3	7.0
DALYs	Haiti	Both	Age-standardized	MDR-TB	Rate	2019	4.9	0.4	20.3
DALYs	Bangladesh	Both	Age-standardized	MDR-TB	Rate	2019	51.5	11.1	133.8
DALYs	Jamaica	Both	Age-standardized	MDR-TB	Rate	2019	0.4	0.1	1.5
DALYs	Guatemala	Both	Age-standardized	MDR-TB	Rate	2019	6.1	0.7	19.7
DALYs	Bhutan	Both	Age-standardized	MDR-TB	Rate	2019	46.5	4.9	154.9
DALYs	Saint Lucia	Both	Age-standardized	MDR-TB	Rate	2019	0.7	0.1	2.9
DALYs	Puerto Rico	Both	Age-standardized	MDR-TB	Rate	2019	0.3	0.0	0.9
DALYs	India	Both	Age-standardized	MDR-TB	Rate	2019	149.7	24.5	379.0
DALYs	Venezuela (Bolivarian Republic of)	Both	Age-standardized	MDR-TB	Rate	2019	3.5	0.4	12.6
DALYs	Northern Mariana Islands	Both	Age-standardized	MDR-TB	Rate	2019	1.7	0.2	7.2

DALYs	C么te d'Ivoire	Both	Age-standardized	MDR-TB	Rate	2019	119.0	16.6	407.8
DALYs	Palestine	Both	Age-standardized	MDR-TB	Rate	2019	1.3	0.1	5.4
DALYs	Palau	Both	Age-standardized	MDR-TB	Rate	2019	0.9	0.1	4.3
DALYs	Marshall Islands	Both	Age-standardized	MDR-TB	Rate	2019	13.9	1.9	49.1
DALYs	Malaysia	Both	Age-standardized	MDR-TB	Rate	2019	3.7	0.8	11.6
DALYs	Georgia	Both	Age-standardized	MDR-TB	Rate	2019	45.1	23.4	75.0
DALYs	Kazakhstan	Both	Age-standardized	MDR-TB	Rate	2019	54.2	26.9	87.5
DALYs	Bosnia and Herzegovina	Both	Age-standardized	MDR-TB	Rate	2019	0.5	0.1	1.4
DALYs	Kyrgyzstan	Both	Age-standardized	MDR-TB	Rate	2019	99.5	38.5	166.3
DALYs	Bulgaria	Both	Age-standardized	MDR-TB	Rate	2019	1.5	0.4	3.8
DALYs	San Marino	Both	Age-standardized	MDR-TB	Rate	2019	0.1	0.0	0.5
DALYs	Australia	Both	Age-standardized	MDR-TB	Rate	2019	0.3	0.1	0.7
DALYs	Egypt	Both	Age-standardized	MDR-TB	Rate	2019	4.4	0.9	12.4
DALYs	Tunisia	Both	Age-standardized	MDR-TB	Rate	2019	0.8	0.2	2.4
DALYs	Equatorial Guinea	Both	Age-standardized	MDR-TB	Rate	2019	53.8	5.4	198.4
DALYs	Tajikistan	Both	Age-standardized	MDR-TB	Rate	2019	91.7	36.8	165.9
DALYs	Brazil	Both	Age-standardized	MDR-TB	Rate	2019	4.9	0.8	13.8
DALYs	Paraguay	Both	Age-standardized	MDR-TB	Rate	2019	9.1	1.0	31.0
DALYs	Ethiopia	Both	Age-standardized	MDR-TB	Rate	2019	122.6	21.2	393.6
DALYs	Lesotho	Both	Age-standardized	MDR-TB	Rate	2019	577.5	152.4	1412.9
DALYs	Guinea-Bissau	Both	Age-standardized	MDR-TB	Rate	2019	100.2	13.5	349.8
DALYs	Namibia	Both	Age-standardized	MDR-TB	Rate	2019	214.2	71.9	461.0
DALYs	Liberia	Both	Age-standardized	MDR-TB	Rate	2019	58.0	6.3	230.7
DALYs	Mauritania	Both	Age-standardized	MDR-TB	Rate	2019	31.7	3.1	109.1
DALYs	Nigeria	Both	Age-standardized	MDR-TB	Rate	2019	107.2	23.5	306.6
DALYs	South Africa	Both	Age-standardized	MDR-TB	Rate	2019	84.5	25.1	208.3
DALYs	Sao Tome and Principe	Both	Age-standardized	MDR-TB	Rate	2019	38.3	4.2	125.7
DALYs	Gabon	Both	Age-standardized	MDR-TB	Rate	2019	81.4	9.0	293.5
DALYs	Sudan	Both	Age-standardized	MDR-TB	Rate	2019	6.8	0.7	29.2
DALYs	Turkmenistan	Both	Age-standardized	MDR-TB	Rate	2019	93.9	34.1	180.6
DALYs	Burundi	Both	Age-standardized	MDR-TB	Rate	2019	330.0	39.4	1194.8
DALYs	Kenya	Both	Age-standardized	MDR-TB	Rate	2019	68.3	17.1	170.9
DALYs	Madagascar	Both	Age-standardized	MDR-TB	Rate	2019	171.5	26.2	589.8
DALYs	Oman	Both	Age-standardized	MDR-TB	Rate	2019	1.2	0.2	3.9
DALYs	Maldives	Both	Age-standardized	MDR-TB	Rate	2019	1.1	0.1	4.7
DALYs	Micronesia (Federated States of)	Both	Age-standardized	MDR-TB	Rate	2019	3.4	0.2	16.9
DALYs	American Samoa	Both	Age-standardized	MDR-TB	Rate	2019	0.3	0.0	1.5
DALYs	Peru	Both	Age-standardized	MDR-TB	Rate	2019	30.5	11.0	63.8
DALYs	Ghana	Both	Age-standardized	MDR-TB	Rate	2019	98.6	12.7	316.2
DALYs	Iran (Islamic Republic of)	Both	Age-standardized	MDR-TB	Rate	2019	1.2	0.3	3.2
DALYs	New Zealand	Both	Age-standardized	MDR-TB	Rate	2019	0.2	0.0	0.7
DALYs	Iraq	Both	Age-standardized	MDR-TB	Rate	2019	7.3	1.8	19.2
DALYs	Malawi	Both	Age-standardized	MDR-TB	Rate	2019	119.4	18.1	363.3
DALYs	Niger	Both	Age-standardized	MDR-TB	Rate	2019	93.7	10.9	312.7
DALYs	Colombia	Both	Age-standardized	MDR-TB	Rate	2019	4.8	0.8	14.3

S.Figure 2A Age-specific distribution of deaths from level 2 GBD causes attributable to alcohol use in 1990 and 2019

measure	location	sex	age	cause	risk factor	metric	year	value	95% UI (lower)	95% UI (upper)
Deaths	Global	Male	15-19 years	Neoplasms	Alcohol use	Number	1990	130	100	167
Deaths	Global	Female	15-19 years	Neoplasms	Alcohol use	Number	1990	36	30	43
Deaths	Global	Male	15-19 years	Neurological disorders	Alcohol use	Number	1990	321	210	438
Deaths	Global	Female	15-19 years	Neurological disorders	Alcohol use	Number	1990	89	58	124
Deaths	Global	Male	15-19 years	Digestive diseases	Alcohol use	Number	1990	985	719	1316
Deaths	Global	Female	15-19 years	Digestive diseases	Alcohol use	Number	1990	329	234	449
Deaths	Global	Male	15-19 years	Unintentional injuries	Alcohol use	Number	1990	1471	665	2580
Deaths	Global	Female	15-19 years	Unintentional injuries	Alcohol use	Number	1990	150	62	279
Deaths	Global	Male	15-19 years	Self-harm and interpersonal violence	Alcohol use	Number	1990	4754	3034	6592
Deaths	Global	Female	15-19 years	Self-harm and interpersonal violence	Alcohol use	Number	1990	622	335	943
Deaths	Global	Male	15-19 years	Respiratory infections and tuberculosis	Alcohol use	Number	1990	1305	803	1839
Deaths	Global	Female	15-19 years	Respiratory infections and tuberculosis	Alcohol use	Number	1990	446	250	662
Deaths	Global	Male	15-19 years	Substance use disorders	Alcohol use	Number	1990	851	737	935
Deaths	Global	Female	15-19 years	Substance use disorders	Alcohol use	Number	1990	344	315	376
Deaths	Global	Male	15-19 years	Transport injuries	Alcohol use	Number	1990	4277	2302	6579
Deaths	Global	Female	15-19 years	Transport injuries	Alcohol use	Number	1990	575	299	899
Deaths	Global	Male	20-24 years	Neoplasms	Alcohol use	Number	1990	535	422	658
Deaths	Global	Female	20-24 years	Neoplasms	Alcohol use	Number	1990	123	106	145
Deaths	Global	Male	20-24 years	Neurological disorders	Alcohol use	Number	1990	770	554	1024
Deaths	Global	Female	20-24 years	Neurological disorders	Alcohol use	Number	1990	158	106	217
Deaths	Global	Male	20-24 years	Digestive diseases	Alcohol use	Number	1990	3491	2590	4562
Deaths	Global	Female	20-24 years	Digestive diseases	Alcohol use	Number	1990	718	530	964
Deaths	Global	Male	20-24 years	Unintentional injuries	Alcohol use	Number	1990	3167	1499	5435
Deaths	Global	Female	20-24 years	Unintentional injuries	Alcohol use	Number	1990	219	96	394
Deaths	Global	Male	20-24 years	Self-harm and interpersonal violence	Alcohol use	Number	1990	13251	8857	18126
Deaths	Global	Female	20-24 years	Self-harm and interpersonal violence	Alcohol use	Number	1990	1307	741	1958
Deaths	Global	Male	20-24 years	Respiratory infections and tuberculosis	Alcohol use	Number	1990	5638	3761	7422
Deaths	Global	Female	20-24 years	Respiratory infections and tuberculosis	Alcohol use	Number	1990	1278	792	1825
Deaths	Global	Male	20-24 years	Substance use disorders	Alcohol use	Number	1990	2107	1845	2290
Deaths	Global	Female	20-24 years	Substance use disorders	Alcohol use	Number	1990	450	412	496
Deaths	Global	Male	20-24 years	Transport injuries	Alcohol use	Number	1990	12370	6790	18097
Deaths	Global	Female	20-24 years	Transport injuries	Alcohol use	Number	1990	789	410	1216
Deaths	Global	Male	25-29 years	Neoplasms	Alcohol use	Number	1990	925	737	1133
Deaths	Global	Female	25-29 years	Neoplasms	Alcohol use	Number	1990	303	263	342
Deaths	Global	Male	25-29 years	Digestive diseases	Alcohol use	Number	1990	7622	5828	9504
Deaths	Global	Female	25-29 years	Digestive diseases	Alcohol use	Number	1990	1275	931	1687
Deaths	Global	Male	25-29 years	Unintentional injuries	Alcohol use	Number	1990	3383	1607	5713
Deaths	Global	Female	25-29 years	Unintentional injuries	Alcohol use	Number	1990	221	99	390
Deaths	Global	Male	25-29 years	Neurological disorders	Alcohol use	Number	1990	850	621	1101
Deaths	Global	Female	25-29 years	Neurological disorders	Alcohol use	Number	1990	174	118	234
Deaths	Global	Male	25-29 years	Self-harm and interpersonal violence	Alcohol use	Number	1990	15488	10528	20655
Deaths	Global	Female	25-29 years	Self-harm and interpersonal violence	Alcohol use	Number	1990	1477	861	2164
Deaths	Global	Male	25-29 years	Respiratory infections and tuberculosis	Alcohol use	Number	1990	9479	6569	12419
Deaths	Global	Female	25-29 years	Respiratory infections and tuberculosis	Alcohol use	Number	1990	1732	1077	2406
Deaths	Global	Male	25-29 years	Substance use disorders	Alcohol use	Number	1990	4270	3742	4552
Deaths	Global	Female	25-29 years	Substance use disorders	Alcohol use	Number	1990	740	687	796
Deaths	Global	Male	25-29 years	Transport injuries	Alcohol use	Number	1990	11200	6271	16393
Deaths	Global	Female	25-29 years	Transport injuries	Alcohol use	Number	1990	570	294	877
Deaths	Global	Male	30-34 years	Neoplasms	Alcohol use	Number	1990	2036	1643	2523
Deaths	Global	Female	30-34 years	Neoplasms	Alcohol use	Number	1990	807	698	911

Deaths	Global	Male	30-34 years	Digestive diseases	Alcohol use	Number	1990	14385	11366	17455
Deaths	Global	Female	30-34 years	Digestive diseases	Alcohol use	Number	1990	2197	1688	2786
Deaths	Global	Male	30-34 years	Unintentional injuries	Alcohol use	Number	1990	3452	1622	5875
Deaths	Global	Female	30-34 years	Unintentional injuries	Alcohol use	Number	1990	235	104	413
Deaths	Global	Male	30-34 years	Neurological disorders	Alcohol use	Number	1990	867	639	1123
Deaths	Global	Female	30-34 years	Neurological disorders	Alcohol use	Number	1990	165	111	225
Deaths	Global	Male	30-34 years	Self-harm and interpersonal violence	Alcohol use	Number	1990	14917	10138	19820
Deaths	Global	Female	30-34 years	Self-harm and interpersonal violence	Alcohol use	Number	1990	1489	837	2198
Deaths	Global	Male	30-34 years	Respiratory infections and tuberculosis	Alcohol use	Number	1990	12421	8472	16106
Deaths	Global	Female	30-34 years	Respiratory infections and tuberculosis	Alcohol use	Number	1990	1794	1105	2548
Deaths	Global	Male	30-34 years	Substance use disorders	Alcohol use	Number	1990	8018	7180	8467
Deaths	Global	Female	30-34 years	Substance use disorders	Alcohol use	Number	1990	1270	1194	1342
Deaths	Global	Male	30-34 years	Transport injuries	Alcohol use	Number	1990	7110	3917	10774
Deaths	Global	Female	30-34 years	Transport injuries	Alcohol use	Number	1990	306	160	473
Deaths	Global	Male	35-39 years	Neoplasms	Alcohol use	Number	1990	4778	3840	5893
Deaths	Global	Female	35-39 years	Neoplasms	Alcohol use	Number	1990	1734	1494	1985
Deaths	Global	Male	35-39 years	Digestive diseases	Alcohol use	Number	1990	24702	19399	29927
Deaths	Global	Female	35-39 years	Digestive diseases	Alcohol use	Number	1990	3537	2672	4525
Deaths	Global	Male	35-39 years	Unintentional injuries	Alcohol use	Number	1990	3726	1742	6399
Deaths	Global	Female	35-39 years	Unintentional injuries	Alcohol use	Number	1990	256	113	462
Deaths	Global	Male	35-39 years	Neurological disorders	Alcohol use	Number	1990	936	683	1192
Deaths	Global	Female	35-39 years	Neurological disorders	Alcohol use	Number	1990	158	107	217
Deaths	Global	Male	35-39 years	Self-harm and interpersonal violence	Alcohol use	Number	1990	14055	9407	19130
Deaths	Global	Female	35-39 years	Self-harm and interpersonal violence	Alcohol use	Number	1990	1516	815	2304
Deaths	Global	Male	35-39 years	Respiratory infections and tuberculosis	Alcohol use	Number	1990	15782	10876	20366
Deaths	Global	Female	35-39 years	Respiratory infections and tuberculosis	Alcohol use	Number	1990	1935	1182	2773
Deaths	Global	Male	35-39 years	Substance use disorders	Alcohol use	Number	1990	11079	10164	11688
Deaths	Global	Female	35-39 years	Substance use disorders	Alcohol use	Number	1990	1957	1836	2059
Deaths	Global	Male	35-39 years	Transport injuries	Alcohol use	Number	1990	5036	2772	7657
Deaths	Global	Female	35-39 years	Transport injuries	Alcohol use	Number	1990	268	140	411
Deaths	Global	Male	40-44 years	Neoplasms	Alcohol use	Number	1990	9345	7801	11161
Deaths	Global	Female	40-44 years	Neoplasms	Alcohol use	Number	1990	2960	2558	3384
Deaths	Global	Male	40-44 years	Digestive diseases	Alcohol use	Number	1990	33507	26666	40080
Deaths	Global	Female	40-44 years	Digestive diseases	Alcohol use	Number	1990	5137	3883	6380
Deaths	Global	Male	40-44 years	Unintentional injuries	Alcohol use	Number	1990	3474	1614	5863
Deaths	Global	Female	40-44 years	Unintentional injuries	Alcohol use	Number	1990	264	116	462
Deaths	Global	Male	40-44 years	Neurological disorders	Alcohol use	Number	1990	825	614	1050
Deaths	Global	Female	40-44 years	Neurological disorders	Alcohol use	Number	1990	147	98	200
Deaths	Global	Male	40-44 years	Self-harm and interpersonal violence	Alcohol use	Number	1990	11794	7905	15890
Deaths	Global	Female	40-44 years	Self-harm and interpersonal violence	Alcohol use	Number	1990	1438	774	2160
Deaths	Global	Male	40-44 years	Respiratory infections and tuberculosis	Alcohol use	Number	1990	17971	12528	23008
Deaths	Global	Female	40-44 years	Respiratory infections and tuberculosis	Alcohol use	Number	1990	1911	1189	2755
Deaths	Global	Male	40-44 years	Substance use disorders	Alcohol use	Number	1990	12184	11114	12886
Deaths	Global	Female	40-44 years	Substance use disorders	Alcohol use	Number	1990	2177	2069	2275
Deaths	Global	Male	40-44 years	Transport injuries	Alcohol use	Number	1990	3877	2116	5919
Deaths	Global	Female	40-44 years	Transport injuries	Alcohol use	Number	1990	395	203	620
Deaths	Global	Male	45-49 years	Neoplasms	Alcohol use	Number	1990	14272	12192	16542
Deaths	Global	Female	45-49 years	Neoplasms	Alcohol use	Number	1990	4142	3575	4720
Deaths	Global	Male	45-49 years	Digestive diseases	Alcohol use	Number	1990	38382	30200	46373
Deaths	Global	Female	45-49 years	Digestive diseases	Alcohol use	Number	1990	6533	4892	8250
Deaths	Global	Male	45-49 years	Unintentional injuries	Alcohol use	Number	1990	3058	1411	5182
Deaths	Global	Female	45-49 years	Unintentional injuries	Alcohol use	Number	1990	255	117	450
Deaths	Global	Male	45-49 years	Neurological disorders	Alcohol use	Number	1990	670	498	853
Deaths	Global	Female	45-49 years	Neurological disorders	Alcohol use	Number	1990	116	79	156
Deaths	Global	Male	45-49 years	Self-harm and interpersonal violence	Alcohol use	Number	1990	9550	6250	12870

Deaths	Global	Female	45-49 years	Self-harm and interpersonal violence	Alcohol use	Number	1990	1278	675	1915
Deaths	Global	Male	45-49 years	Respiratory infections and tuberculosis	Alcohol use	Number	1990	20568	13941	26884
Deaths	Global	Female	45-49 years	Respiratory infections and tuberculosis	Alcohol use	Number	1990	2024	1240	2848
Deaths	Global	Male	45-49 years	Substance use disorders	Alcohol use	Number	1990	12054	10987	12880
Deaths	Global	Female	45-49 years	Substance use disorders	Alcohol use	Number	1990	2179	2068	2267
Deaths	Global	Male	45-49 years	Transport injuries	Alcohol use	Number	1990	3650	2005	5562
Deaths	Global	Female	45-49 years	Transport injuries	Alcohol use	Number	1990	246	128	378
Deaths	Global	Male	50-54 years	Neoplasms	Alcohol use	Number	1990	23265	20036	26533
Deaths	Global	Female	50-54 years	Neoplasms	Alcohol use	Number	1990	5998	5181	6845
Deaths	Global	Male	50-54 years	Self-harm and interpersonal violence	Alcohol use	Number	1990	9393	6070	12655
Deaths	Global	Female	50-54 years	Self-harm and interpersonal violence	Alcohol use	Number	1990	1318	656	2020
Deaths	Global	Male	50-54 years	Digestive diseases	Alcohol use	Number	1990	46899	37027	56071
Deaths	Global	Female	50-54 years	Digestive diseases	Alcohol use	Number	1990	9153	6766	11685
Deaths	Global	Male	50-54 years	Unintentional injuries	Alcohol use	Number	1990	3382	1577	5740
Deaths	Global	Female	50-54 years	Unintentional injuries	Alcohol use	Number	1990	321	139	585
Deaths	Global	Male	50-54 years	Neurological disorders	Alcohol use	Number	1990	575	427	736
Deaths	Global	Female	50-54 years	Neurological disorders	Alcohol use	Number	1990	110	73	151
Deaths	Global	Male	50-54 years	Respiratory infections and tuberculosis	Alcohol use	Number	1990	24031	16652	30936
Deaths	Global	Female	50-54 years	Respiratory infections and tuberculosis	Alcohol use	Number	1990	2679	1629	3846
Deaths	Global	Male	50-54 years	Substance use disorders	Alcohol use	Number	1990	13488	12329	14172
Deaths	Global	Female	50-54 years	Substance use disorders	Alcohol use	Number	1990	2947	2804	3060
Deaths	Global	Male	50-54 years	Transport injuries	Alcohol use	Number	1990	2892	1597	4420
Deaths	Global	Female	50-54 years	Transport injuries	Alcohol use	Number	1990	274	142	426
Deaths	Global	Male	55-59 years	Neoplasms	Alcohol use	Number	1990	30425	26539	34850
Deaths	Global	Female	55-59 years	Neoplasms	Alcohol use	Number	1990	7423	6482	8451
Deaths	Global	Male	55-59 years	Self-harm and interpersonal violence	Alcohol use	Number	1990	7528	4704	10350
Deaths	Global	Female	55-59 years	Self-harm and interpersonal violence	Alcohol use	Number	1990	1144	568	1782
Deaths	Global	Male	55-59 years	Digestive diseases	Alcohol use	Number	1990	50284	39663	60929
Deaths	Global	Female	55-59 years	Digestive diseases	Alcohol use	Number	1990	11614	8750	14950
Deaths	Global	Male	55-59 years	Unintentional injuries	Alcohol use	Number	1990	2924	1369	4925
Deaths	Global	Female	55-59 years	Unintentional injuries	Alcohol use	Number	1990	311	136	550
Deaths	Global	Male	55-59 years	Neurological disorders	Alcohol use	Number	1990	496	364	635
Deaths	Global	Female	55-59 years	Neurological disorders	Alcohol use	Number	1990	90	61	120
Deaths	Global	Male	55-59 years	Respiratory infections and tuberculosis	Alcohol use	Number	1990	25530	17590	33065
Deaths	Global	Female	55-59 years	Respiratory infections and tuberculosis	Alcohol use	Number	1990	3006	1881	4308
Deaths	Global	Male	55-59 years	Substance use disorders	Alcohol use	Number	1990	11461	10429	12110
Deaths	Global	Female	55-59 years	Substance use disorders	Alcohol use	Number	1990	2492	2386	2587
Deaths	Global	Male	55-59 years	Transport injuries	Alcohol use	Number	1990	2727	1490	4156
Deaths	Global	Female	55-59 years	Transport injuries	Alcohol use	Number	1990	319	166	496
Deaths	Global	Male	60-64 years	Neoplasms	Alcohol use	Number	1990	34702	30432	39209
Deaths	Global	Female	60-64 years	Neoplasms	Alcohol use	Number	1990	8906	7765	10111
Deaths	Global	Male	60-64 years	Self-harm and interpersonal violence	Alcohol use	Number	1990	6179	3830	8631
Deaths	Global	Female	60-64 years	Self-harm and interpersonal violence	Alcohol use	Number	1990	1068	525	1650
Deaths	Global	Male	60-64 years	Digestive diseases	Alcohol use	Number	1990	49967	39625	60437
Deaths	Global	Female	60-64 years	Digestive diseases	Alcohol use	Number	1990	13557	10226	17527
Deaths	Global	Male	60-64 years	Unintentional injuries	Alcohol use	Number	1990	2676	1260	4513
Deaths	Global	Female	60-64 years	Unintentional injuries	Alcohol use	Number	1990	366	165	669
Deaths	Global	Male	60-64 years	Neurological disorders	Alcohol use	Number	1990	437	318	563
Deaths	Global	Female	60-64 years	Neurological disorders	Alcohol use	Number	1990	85	57	113
Deaths	Global	Male	60-64 years	Respiratory infections and tuberculosis	Alcohol use	Number	1990	26583	18499	34387
Deaths	Global	Female	60-64 years	Respiratory infections and tuberculosis	Alcohol use	Number	1990	3207	2011	4529
Deaths	Global	Male	60-64 years	Substance use disorders	Alcohol use	Number	1990	9049	8225	9536
Deaths	Global	Female	60-64 years	Substance use disorders	Alcohol use	Number	1990	2176	2098	2249
Deaths	Global	Male	60-64 years	Transport injuries	Alcohol use	Number	1990	1965	1074	2998
Deaths	Global	Female	60-64 years	Transport injuries	Alcohol use	Number	1990	204	105	319

Deaths	Global	Male	65-69 years	Neoplasms	Alcohol use	Number	1990	31852	28014	36101
Deaths	Global	Female	65-69 years	Neoplasms	Alcohol use	Number	1990	9571	8248	10933
Deaths	Global	Male	65-69 years	Self-harm and interpersonal violence	Alcohol use	Number	1990	4391	2602	6258
Deaths	Global	Female	65-69 years	Self-harm and interpersonal violence	Alcohol use	Number	1990	924	429	1457
Deaths	Global	Male	65-69 years	Digestive diseases	Alcohol use	Number	1990	40608	32401	48635
Deaths	Global	Female	65-69 years	Digestive diseases	Alcohol use	Number	1990	13386	10067	16947
Deaths	Global	Male	65-69 years	Unintentional injuries	Alcohol use	Number	1990	2001	930	3366
Deaths	Global	Female	65-69 years	Unintentional injuries	Alcohol use	Number	1990	391	174	702
Deaths	Global	Male	65-69 years	Neurological disorders	Alcohol use	Number	1990	336	245	441
Deaths	Global	Female	65-69 years	Neurological disorders	Alcohol use	Number	1990	81	55	111
Deaths	Global	Male	65-69 years	Respiratory infections and tuberculosis	Alcohol use	Number	1990	23702	16586	30382
Deaths	Global	Female	65-69 years	Respiratory infections and tuberculosis	Alcohol use	Number	1990	3073	1929	4399
Deaths	Global	Male	65-69 years	Substance use disorders	Alcohol use	Number	1990	5174	4759	5447
Deaths	Global	Female	65-69 years	Substance use disorders	Alcohol use	Number	1990	1543	1476	1601
Deaths	Global	Male	65-69 years	Transport injuries	Alcohol use	Number	1990	1481	803	2248
Deaths	Global	Female	65-69 years	Transport injuries	Alcohol use	Number	1990	164	85	254
Deaths	Global	Male	70-74 years	Neoplasms	Alcohol use	Number	1990	24145	20868	27660
Deaths	Global	Female	70-74 years	Neoplasms	Alcohol use	Number	1990	8338	7135	9638
Deaths	Global	Male	70-74 years	Self-harm and interpersonal violence	Alcohol use	Number	1990	3112	1803	4498
Deaths	Global	Female	70-74 years	Self-harm and interpersonal violence	Alcohol use	Number	1990	727	320	1175
Deaths	Global	Male	70-74 years	Digestive diseases	Alcohol use	Number	1990	27249	21344	33242
Deaths	Global	Female	70-74 years	Digestive diseases	Alcohol use	Number	1990	10972	8343	13998
Deaths	Global	Male	70-74 years	Unintentional injuries	Alcohol use	Number	1990	1577	743	2693
Deaths	Global	Female	70-74 years	Unintentional injuries	Alcohol use	Number	1990	439	198	775
Deaths	Global	Male	70-74 years	Neurological disorders	Alcohol use	Number	1990	241	175	312
Deaths	Global	Female	70-74 years	Neurological disorders	Alcohol use	Number	1990	71	46	100
Deaths	Global	Male	70-74 years	Respiratory infections and tuberculosis	Alcohol use	Number	1990	20730	14459	27257
Deaths	Global	Female	70-74 years	Respiratory infections and tuberculosis	Alcohol use	Number	1990	3364	2178	4821
Deaths	Global	Male	70-74 years	Substance use disorders	Alcohol use	Number	1990	2785	2488	2965
Deaths	Global	Female	70-74 years	Substance use disorders	Alcohol use	Number	1990	783	746	816
Deaths	Global	Male	70-74 years	Transport injuries	Alcohol use	Number	1990	1196	653	1831
Deaths	Global	Female	70-74 years	Transport injuries	Alcohol use	Number	1990	339	175	537
Deaths	Global	Male	75-79 years	Neoplasms	Alcohol use	Number	1990	18423	16169	20797
Deaths	Global	Female	75-79 years	Neoplasms	Alcohol use	Number	1990	8066	6916	9163
Deaths	Global	Male	75-79 years	Self-harm and interpersonal violence	Alcohol use	Number	1990	2566	1427	3751
Deaths	Global	Female	75-79 years	Self-harm and interpersonal violence	Alcohol use	Number	1990	647	285	1037
Deaths	Global	Male	75-79 years	Digestive diseases	Alcohol use	Number	1990	20035	16109	24105
Deaths	Global	Female	75-79 years	Digestive diseases	Alcohol use	Number	1990	9772	7440	12388
Deaths	Global	Male	75-79 years	Unintentional injuries	Alcohol use	Number	1990	1549	717	2640
Deaths	Global	Female	75-79 years	Unintentional injuries	Alcohol use	Number	1990	674	299	1178
Deaths	Global	Male	75-79 years	Neurological disorders	Alcohol use	Number	1990	198	144	256
Deaths	Global	Female	75-79 years	Neurological disorders	Alcohol use	Number	1990	68	45	99
Deaths	Global	Male	75-79 years	Respiratory infections and tuberculosis	Alcohol use	Number	1990	15648	11102	20258
Deaths	Global	Female	75-79 years	Respiratory infections and tuberculosis	Alcohol use	Number	1990	3060	1765	4470
Deaths	Global	Male	75-79 years	Substance use disorders	Alcohol use	Number	1990	2016	1786	2147
Deaths	Global	Female	75-79 years	Substance use disorders	Alcohol use	Number	1990	765	714	812
Deaths	Global	Male	75-79 years	Transport injuries	Alcohol use	Number	1990	1468	805	2188
Deaths	Global	Female	75-79 years	Transport injuries	Alcohol use	Number	1990	327	170	516
Deaths	Global	Male	80-84 years	Neoplasms	Alcohol use	Number	1990	9827	8501	11179
Deaths	Global	Female	80-84 years	Neoplasms	Alcohol use	Number	1990	5826	4905	6752
Deaths	Global	Male	80-84 years	Self-harm and interpersonal violence	Alcohol use	Number	1990	1491	781	2236
Deaths	Global	Female	80-84 years	Self-harm and interpersonal violence	Alcohol use	Number	1990	406	168	661
Deaths	Global	Male	80-84 years	Digestive diseases	Alcohol use	Number	1990	10827	8513	13196
Deaths	Global	Female	80-84 years	Digestive diseases	Alcohol use	Number	1990	6912	5151	8849
Deaths	Global	Male	80-84 years	Unintentional injuries	Alcohol use	Number	1990	1264	590	2167

Deaths	Global	Female	80-84 years	Unintentional injuries	Alcohol use	Number	1990	886	396	1574
Deaths	Global	Male	80-84 years	Neurological disorders	Alcohol use	Number	1990	115	82	149
Deaths	Global	Female	80-84 years	Neurological disorders	Alcohol use	Number	1990	57	38	87
Deaths	Global	Male	80-84 years	Substance use disorders	Alcohol use	Number	1990	847	771	898
Deaths	Global	Female	80-84 years	Substance use disorders	Alcohol use	Number	1990	426	389	458
Deaths	Global	Male	80-84 years	Respiratory infections and tuberculosis	Alcohol use	Number	1990	10022	6816	13204
Deaths	Global	Female	80-84 years	Respiratory infections and tuberculosis	Alcohol use	Number	1990	2866	1535	4202
Deaths	Global	Male	80-84 years	Transport injuries	Alcohol use	Number	1990	1230	693	1758
Deaths	Global	Female	80-84 years	Transport injuries	Alcohol use	Number	1990	432	223	665
Deaths	Global	Male	85-89 years	Neoplasms	Alcohol use	Number	1990	4353	3707	4960
Deaths	Global	Female	85-89 years	Neoplasms	Alcohol use	Number	1990	3671	3000	4313
Deaths	Global	Male	85-89 years	Self-harm and interpersonal violence	Alcohol use	Number	1990	715	369	1061
Deaths	Global	Female	85-89 years	Self-harm and interpersonal violence	Alcohol use	Number	1990	215	90	353
Deaths	Global	Male	85-89 years	Digestive diseases	Alcohol use	Number	1990	4774	3774	5820
Deaths	Global	Female	85-89 years	Digestive diseases	Alcohol use	Number	1990	3867	2840	5000
Deaths	Global	Male	85-89 years	Unintentional injuries	Alcohol use	Number	1990	897	422	1519
Deaths	Global	Female	85-89 years	Unintentional injuries	Alcohol use	Number	1990	915	424	1605
Deaths	Global	Male	85-89 years	Neurological disorders	Alcohol use	Number	1990	58	42	75
Deaths	Global	Female	85-89 years	Neurological disorders	Alcohol use	Number	1990	40	26	60
Deaths	Global	Male	85-89 years	Substance use disorders	Alcohol use	Number	1990	352	312	378
Deaths	Global	Female	85-89 years	Substance use disorders	Alcohol use	Number	1990	250	217	278
Deaths	Global	Male	85-89 years	Respiratory infections and tuberculosis	Alcohol use	Number	1990	5875	3714	7813
Deaths	Global	Female	85-89 years	Respiratory infections and tuberculosis	Alcohol use	Number	1990	2414	1139	3738
Deaths	Global	Male	85-89 years	Transport injuries	Alcohol use	Number	1990	530	302	761
Deaths	Global	Female	85-89 years	Transport injuries	Alcohol use	Number	1990	215	112	334
Deaths	Global	Male	90-94 years	Neoplasms	Alcohol use	Number	1990	1100	911	1276
Deaths	Global	Female	90-94 years	Neoplasms	Alcohol use	Number	1990	1459	1118	1764
Deaths	Global	Male	90-94 years	Self-harm and interpersonal violence	Alcohol use	Number	1990	165	86	248
Deaths	Global	Female	90-94 years	Self-harm and interpersonal violence	Alcohol use	Number	1990	58	25	95
Deaths	Global	Male	90-94 years	Digestive diseases	Alcohol use	Number	1990	1347	1036	1666
Deaths	Global	Female	90-94 years	Digestive diseases	Alcohol use	Number	1990	1507	1039	2008
Deaths	Global	Male	90-94 years	Unintentional injuries	Alcohol use	Number	1990	363	171	614
Deaths	Global	Female	90-94 years	Unintentional injuries	Alcohol use	Number	1990	524	249	925
Deaths	Global	Male	90-94 years	Neurological disorders	Alcohol use	Number	1990	19	14	24
Deaths	Global	Female	90-94 years	Neurological disorders	Alcohol use	Number	1990	18	12	26
Deaths	Global	Male	90-94 years	Substance use disorders	Alcohol use	Number	1990	82	69	89
Deaths	Global	Female	90-94 years	Substance use disorders	Alcohol use	Number	1990	73	59	81
Deaths	Global	Male	90-94 years	Respiratory infections and tuberculosis	Alcohol use	Number	1990	2026	1219	2792
Deaths	Global	Female	90-94 years	Respiratory infections and tuberculosis	Alcohol use	Number	1990	1218	500	1969
Deaths	Global	Male	90-94 years	Transport injuries	Alcohol use	Number	1990	127	72	183
Deaths	Global	Female	90-94 years	Transport injuries	Alcohol use	Number	1990	63	33	98
Deaths	Global	Male	95+ years	Neoplasms	Alcohol use	Number	1990	225	179	266
Deaths	Global	Female	95+ years	Neoplasms	Alcohol use	Number	1990	439	330	531
Deaths	Global	Male	95+ years	Digestive diseases	Alcohol use	Number	1990	349	247	449
Deaths	Global	Female	95+ years	Digestive diseases	Alcohol use	Number	1990	507	319	710
Deaths	Global	Male	95+ years	Self-harm and interpersonal violence	Alcohol use	Number	1990	28	15	41
Deaths	Global	Female	95+ years	Self-harm and interpersonal violence	Alcohol use	Number	1990	11	5	17
Deaths	Global	Male	95+ years	Unintentional injuries	Alcohol use	Number	1990	103	50	177
Deaths	Global	Female	95+ years	Unintentional injuries	Alcohol use	Number	1990	203	91	352
Deaths	Global	Male	95+ years	Neurological disorders	Alcohol use	Number	1990	5	3	6
Deaths	Global	Female	95+ years	Neurological disorders	Alcohol use	Number	1990	6	4	9
Deaths	Global	Male	95+ years	Respiratory infections and tuberculosis	Alcohol use	Number	1990	557	302	814
Deaths	Global	Female	95+ years	Respiratory infections and tuberculosis	Alcohol use	Number	1990	498	190	832
Deaths	Global	Male	95+ years	Substance use disorders	Alcohol use	Number	1990	23	18	26
Deaths	Global	Female	95+ years	Substance use disorders	Alcohol use	Number	1990	24	19	27

Deaths	Global	Male	95+ years	Transport injuries	Alcohol use	Number	1990	30	17	43
Deaths	Global	Female	95+ years	Transport injuries	Alcohol use	Number	1990	18	9	28
Deaths	Global	Male	15-19 years	Transport injuries	Alcohol use	Number	2019	3484	1909	5421
Deaths	Global	Female	15-19 years	Transport injuries	Alcohol use	Number	2019	404	205	643
Deaths	Global	Male	15-19 years	Neurological disorders	Alcohol use	Number	2019	301	200	422
Deaths	Global	Female	15-19 years	Neurological disorders	Alcohol use	Number	2019	78	51	111
Deaths	Global	Male	15-19 years	Digestive diseases	Alcohol use	Number	2019	1213	818	1692
Deaths	Global	Female	15-19 years	Digestive diseases	Alcohol use	Number	2019	349	223	516
Deaths	Global	Male	15-19 years	Unintentional injuries	Alcohol use	Number	2019	837	378	1468
Deaths	Global	Female	15-19 years	Unintentional injuries	Alcohol use	Number	2019	84	34	153
Deaths	Global	Male	15-19 years	Respiratory infections and tuberculosis	Alcohol use	Number	2019	944	563	1350
Deaths	Global	Female	15-19 years	Respiratory infections and tuberculosis	Alcohol use	Number	2019	305	165	455
Deaths	Global	Male	15-19 years	Self-harm and interpersonal violence	Alcohol use	Number	2019	4261	2740	5913
Deaths	Global	Female	15-19 years	Self-harm and interpersonal violence	Alcohol use	Number	2019	537	290	808
Deaths	Global	Male	15-19 years	Substance use disorders	Alcohol use	Number	2019	592	433	673
Deaths	Global	Female	15-19 years	Substance use disorders	Alcohol use	Number	2019	129	120	139
Deaths	Global	Male	15-19 years	Neoplasms	Alcohol use	Number	2019	109	88	132
Deaths	Global	Female	15-19 years	Neoplasms	Alcohol use	Number	2019	34	28	40
Deaths	Global	Male	20-24 years	Transport injuries	Alcohol use	Number	2019	11584	6557	16741
Deaths	Global	Female	20-24 years	Transport injuries	Alcohol use	Number	2019	597	305	948
Deaths	Global	Male	20-24 years	Neurological disorders	Alcohol use	Number	2019	816	586	1094
Deaths	Global	Female	20-24 years	Neurological disorders	Alcohol use	Number	2019	142	94	195
Deaths	Global	Male	20-24 years	Digestive diseases	Alcohol use	Number	2019	4755	3402	6332
Deaths	Global	Female	20-24 years	Digestive diseases	Alcohol use	Number	2019	840	571	1194
Deaths	Global	Male	20-24 years	Unintentional injuries	Alcohol use	Number	2019	2219	1031	3773
Deaths	Global	Female	20-24 years	Unintentional injuries	Alcohol use	Number	2019	142	63	251
Deaths	Global	Male	20-24 years	Respiratory infections and tuberculosis	Alcohol use	Number	2019	4749	3095	6296
Deaths	Global	Female	20-24 years	Respiratory infections and tuberculosis	Alcohol use	Number	2019	897	530	1282
Deaths	Global	Male	20-24 years	Self-harm and interpersonal violence	Alcohol use	Number	2019	13270	8944	17839
Deaths	Global	Female	20-24 years	Self-harm and interpersonal violence	Alcohol use	Number	2019	1044	613	1508
Deaths	Global	Male	20-24 years	Substance use disorders	Alcohol use	Number	2019	1961	1477	2209
Deaths	Global	Female	20-24 years	Substance use disorders	Alcohol use	Number	2019	237	217	259
Deaths	Global	Male	20-24 years	Neoplasms	Alcohol use	Number	2019	546	456	657
Deaths	Global	Female	20-24 years	Neoplasms	Alcohol use	Number	2019	130	109	153
Deaths	Global	Male	25-29 years	Neurological disorders	Alcohol use	Number	2019	1075	785	1410
Deaths	Global	Female	25-29 years	Neurological disorders	Alcohol use	Number	2019	163	109	225
Deaths	Global	Male	25-29 years	Transport injuries	Alcohol use	Number	2019	12161	6887	17597
Deaths	Global	Female	25-29 years	Transport injuries	Alcohol use	Number	2019	441	224	694
Deaths	Global	Male	25-29 years	Digestive diseases	Alcohol use	Number	2019	11306	8502	14254
Deaths	Global	Female	25-29 years	Digestive diseases	Alcohol use	Number	2019	1588	1143	2128
Deaths	Global	Male	25-29 years	Unintentional injuries	Alcohol use	Number	2019	2820	1345	4760
Deaths	Global	Female	25-29 years	Unintentional injuries	Alcohol use	Number	2019	164	70	285
Deaths	Global	Male	25-29 years	Respiratory infections and tuberculosis	Alcohol use	Number	2019	8939	6074	11575
Deaths	Global	Female	25-29 years	Respiratory infections and tuberculosis	Alcohol use	Number	2019	1265	772	1797
Deaths	Global	Male	25-29 years	Self-harm and interpersonal violence	Alcohol use	Number	2019	16600	11352	21972
Deaths	Global	Female	25-29 years	Self-harm and interpersonal violence	Alcohol use	Number	2019	1198	709	1740
Deaths	Global	Male	25-29 years	Substance use disorders	Alcohol use	Number	2019	4487	3653	4908
Deaths	Global	Female	25-29 years	Substance use disorders	Alcohol use	Number	2019	574	513	633
Deaths	Global	Male	25-29 years	Neoplasms	Alcohol use	Number	2019	1205	1012	1423
Deaths	Global	Female	25-29 years	Neoplasms	Alcohol use	Number	2019	322	274	373
Deaths	Global	Male	30-34 years	Neurological disorders	Alcohol use	Number	2019	1183	864	1569
Deaths	Global	Female	30-34 years	Neurological disorders	Alcohol use	Number	2019	172	114	238
Deaths	Global	Male	30-34 years	Transport injuries	Alcohol use	Number	2019	8463	4761	12983
Deaths	Global	Female	30-34 years	Transport injuries	Alcohol use	Number	2019	269	136	420
Deaths	Global	Male	30-34 years	Digestive diseases	Alcohol use	Number	2019	21160	16289	26055

Deaths	Global	Female	30-34 years	Digestive diseases	Alcohol use	Number	2019	3054	2306	3887
Deaths	Global	Male	30-34 years	Unintentional injuries	Alcohol use	Number	2019	3323	1581	5645
Deaths	Global	Female	30-34 years	Unintentional injuries	Alcohol use	Number	2019	200	87	350
Deaths	Global	Male	30-34 years	Respiratory infections and tuberculosis	Alcohol use	Number	2019	12521	8569	16196
Deaths	Global	Female	30-34 years	Respiratory infections and tuberculosis	Alcohol use	Number	2019	1466	873	2055
Deaths	Global	Male	30-34 years	Self-harm and interpersonal violence	Alcohol use	Number	2019	17178	11544	22688
Deaths	Global	Female	30-34 years	Self-harm and interpersonal violence	Alcohol use	Number	2019	1276	740	1838
Deaths	Global	Male	30-34 years	Substance use disorders	Alcohol use	Number	2019	9428	7665	10255
Deaths	Global	Female	30-34 years	Substance use disorders	Alcohol use	Number	2019	1213	1076	1354
Deaths	Global	Male	30-34 years	Neoplasms	Alcohol use	Number	2019	2980	2500	3504
Deaths	Global	Female	30-34 years	Neoplasms	Alcohol use	Number	2019	840	714	970
Deaths	Global	Male	35-39 years	Neurological disorders	Alcohol use	Number	2019	1244	905	1633
Deaths	Global	Female	35-39 years	Neurological disorders	Alcohol use	Number	2019	169	113	235
Deaths	Global	Male	35-39 years	Transport injuries	Alcohol use	Number	2019	6024	3435	9310
Deaths	Global	Female	35-39 years	Transport injuries	Alcohol use	Number	2019	234	118	369
Deaths	Global	Male	35-39 years	Digestive diseases	Alcohol use	Number	2019	34137	26270	41994
Deaths	Global	Female	35-39 years	Digestive diseases	Alcohol use	Number	2019	4627	3372	5904
Deaths	Global	Male	35-39 years	Unintentional injuries	Alcohol use	Number	2019	3596	1695	6113
Deaths	Global	Female	35-39 years	Unintentional injuries	Alcohol use	Number	2019	225	98	403
Deaths	Global	Male	35-39 years	Respiratory infections and tuberculosis	Alcohol use	Number	2019	15749	10814	20160
Deaths	Global	Female	35-39 years	Respiratory infections and tuberculosis	Alcohol use	Number	2019	1651	1002	2323
Deaths	Global	Male	35-39 years	Self-harm and interpersonal violence	Alcohol use	Number	2019	16055	10951	21387
Deaths	Global	Female	35-39 years	Self-harm and interpersonal violence	Alcohol use	Number	2019	1295	724	1891
Deaths	Global	Male	35-39 years	Substance use disorders	Alcohol use	Number	2019	12842	10643	13949
Deaths	Global	Female	35-39 years	Substance use disorders	Alcohol use	Number	2019	1921	1710	2131
Deaths	Global	Male	35-39 years	Neoplasms	Alcohol use	Number	2019	5938	5038	6932
Deaths	Global	Female	35-39 years	Neoplasms	Alcohol use	Number	2019	1694	1452	1956
Deaths	Global	Male	40-44 years	Neurological disorders	Alcohol use	Number	2019	1183	875	1566
Deaths	Global	Female	40-44 years	Neurological disorders	Alcohol use	Number	2019	192	130	262
Deaths	Global	Male	40-44 years	Transport injuries	Alcohol use	Number	2019	4882	2781	7538
Deaths	Global	Female	40-44 years	Transport injuries	Alcohol use	Number	2019	319	162	506
Deaths	Global	Male	40-44 years	Digestive diseases	Alcohol use	Number	2019	46979	36341	57495
Deaths	Global	Female	40-44 years	Digestive diseases	Alcohol use	Number	2019	7062	5206	8957
Deaths	Global	Male	40-44 years	Unintentional injuries	Alcohol use	Number	2019	3860	1851	6361
Deaths	Global	Female	40-44 years	Unintentional injuries	Alcohol use	Number	2019	267	119	475
Deaths	Global	Male	40-44 years	Respiratory infections and tuberculosis	Alcohol use	Number	2019	17623	12152	22391
Deaths	Global	Female	40-44 years	Respiratory infections and tuberculosis	Alcohol use	Number	2019	1875	1150	2725
Deaths	Global	Male	40-44 years	Self-harm and interpersonal violence	Alcohol use	Number	2019	14123	9599	19083
Deaths	Global	Female	40-44 years	Self-harm and interpersonal violence	Alcohol use	Number	2019	1375	788	1991
Deaths	Global	Male	40-44 years	Substance use disorders	Alcohol use	Number	2019	15740	13306	17142
Deaths	Global	Female	40-44 years	Substance use disorders	Alcohol use	Number	2019	2384	2151	2622
Deaths	Global	Male	40-44 years	Neoplasms	Alcohol use	Number	2019	11858	10176	13646
Deaths	Global	Female	40-44 years	Neoplasms	Alcohol use	Number	2019	3080	2619	3561
Deaths	Global	Male	45-49 years	Neurological disorders	Alcohol use	Number	2019	1120	835	1447
Deaths	Global	Female	45-49 years	Neurological disorders	Alcohol use	Number	2019	178	122	240
Deaths	Global	Male	45-49 years	Transport injuries	Alcohol use	Number	2019	4629	2598	7159
Deaths	Global	Female	45-49 years	Transport injuries	Alcohol use	Number	2019	223	113	354
Deaths	Global	Male	45-49 years	Digestive diseases	Alcohol use	Number	2019	60189	46878	74028
Deaths	Global	Female	45-49 years	Digestive diseases	Alcohol use	Number	2019	9535	7064	12265
Deaths	Global	Male	45-49 years	Unintentional injuries	Alcohol use	Number	2019	4200	1949	7041
Deaths	Global	Female	45-49 years	Unintentional injuries	Alcohol use	Number	2019	299	133	513
Deaths	Global	Male	45-49 years	Respiratory infections and tuberculosis	Alcohol use	Number	2019	20834	14555	26315
Deaths	Global	Female	45-49 years	Respiratory infections and tuberculosis	Alcohol use	Number	2019	2104	1301	2978
Deaths	Global	Male	45-49 years	Self-harm and interpersonal violence	Alcohol use	Number	2019	13119	8738	17677
Deaths	Global	Female	45-49 years	Self-harm and interpersonal violence	Alcohol use	Number	2019	1447	814	2121

Deaths	Global	Male	45-49 years	Substance use disorders	Alcohol use	Number	2019	19094	15983	20878
Deaths	Global	Female	45-49 years	Substance use disorders	Alcohol use	Number	2019	2878	2609	3141
Deaths	Global	Male	45-49 years	Neoplasms	Alcohol use	Number	2019	21850	18609	25216
Deaths	Global	Female	45-49 years	Neoplasms	Alcohol use	Number	2019	5004	4275	5743
Deaths	Global	Male	50-54 years	Neurological disorders	Alcohol use	Number	2019	961	714	1242
Deaths	Global	Female	50-54 years	Neurological disorders	Alcohol use	Number	2019	190	132	258
Deaths	Global	Male	50-54 years	Transport injuries	Alcohol use	Number	2019	3781	2137	5858
Deaths	Global	Female	50-54 years	Transport injuries	Alcohol use	Number	2019	270	136	426
Deaths	Global	Male	50-54 years	Digestive diseases	Alcohol use	Number	2019	71022	54082	87313
Deaths	Global	Female	50-54 years	Digestive diseases	Alcohol use	Number	2019	13078	9480	17134
Deaths	Global	Male	50-54 years	Self-harm and interpersonal violence	Alcohol use	Number	2019	11787	7805	15979
Deaths	Global	Female	50-54 years	Self-harm and interpersonal violence	Alcohol use	Number	2019	1438	759	2166
Deaths	Global	Male	50-54 years	Unintentional injuries	Alcohol use	Number	2019	4332	2027	7230
Deaths	Global	Female	50-54 years	Unintentional injuries	Alcohol use	Number	2019	374	169	662
Deaths	Global	Male	50-54 years	Respiratory infections and tuberculosis	Alcohol use	Number	2019	22535	15992	28880
Deaths	Global	Female	50-54 years	Respiratory infections and tuberculosis	Alcohol use	Number	2019	2791	1718	4046
Deaths	Global	Male	50-54 years	Substance use disorders	Alcohol use	Number	2019	18386	15604	20054
Deaths	Global	Female	50-54 years	Substance use disorders	Alcohol use	Number	2019	3154	2860	3433
Deaths	Global	Male	50-54 years	Neoplasms	Alcohol use	Number	2019	36335	30911	41577
Deaths	Global	Female	50-54 years	Neoplasms	Alcohol use	Number	2019	7692	6529	8845
Deaths	Global	Male	55-59 years	Neurological disorders	Alcohol use	Number	2019	905	658	1156
Deaths	Global	Female	55-59 years	Neurological disorders	Alcohol use	Number	2019	166	113	226
Deaths	Global	Male	55-59 years	Transport injuries	Alcohol use	Number	2019	3470	1953	5399
Deaths	Global	Female	55-59 years	Transport injuries	Alcohol use	Number	2019	324	162	513
Deaths	Global	Male	55-59 years	Digestive diseases	Alcohol use	Number	2019	77100	59124	94495
Deaths	Global	Female	55-59 years	Digestive diseases	Alcohol use	Number	2019	17723	12834	23293
Deaths	Global	Male	55-59 years	Self-harm and interpersonal violence	Alcohol use	Number	2019	10547	6709	14446
Deaths	Global	Female	55-59 years	Self-harm and interpersonal violence	Alcohol use	Number	2019	1307	690	1945
Deaths	Global	Male	55-59 years	Unintentional injuries	Alcohol use	Number	2019	4209	1997	7015
Deaths	Global	Female	55-59 years	Unintentional injuries	Alcohol use	Number	2019	413	190	711
Deaths	Global	Male	55-59 years	Respiratory infections and tuberculosis	Alcohol use	Number	2019	24664	17164	31242
Deaths	Global	Female	55-59 years	Respiratory infections and tuberculosis	Alcohol use	Number	2019	3128	1968	4373
Deaths	Global	Male	55-59 years	Substance use disorders	Alcohol use	Number	2019	19025	16477	20556
Deaths	Global	Female	55-59 years	Substance use disorders	Alcohol use	Number	2019	3395	3076	3725
Deaths	Global	Male	55-59 years	Neoplasms	Alcohol use	Number	2019	49000	42414	56119
Deaths	Global	Female	55-59 years	Neoplasms	Alcohol use	Number	2019	10017	8642	11448
Deaths	Global	Male	60-64 years	Neurological disorders	Alcohol use	Number	2019	791	584	1022
Deaths	Global	Female	60-64 years	Neurological disorders	Alcohol use	Number	2019	155	106	212
Deaths	Global	Male	60-64 years	Transport injuries	Alcohol use	Number	2019	2640	1486	4133
Deaths	Global	Female	60-64 years	Transport injuries	Alcohol use	Number	2019	185	93	295
Deaths	Global	Male	60-64 years	Digestive diseases	Alcohol use	Number	2019	75951	58406	92918
Deaths	Global	Female	60-64 years	Digestive diseases	Alcohol use	Number	2019	19924	14703	26054
Deaths	Global	Male	60-64 years	Self-harm and interpersonal violence	Alcohol use	Number	2019	8300	5189	11433
Deaths	Global	Female	60-64 years	Self-harm and interpersonal violence	Alcohol use	Number	2019	1077	541	1645
Deaths	Global	Male	60-64 years	Unintentional injuries	Alcohol use	Number	2019	4068	1892	6681
Deaths	Global	Female	60-64 years	Unintentional injuries	Alcohol use	Number	2019	491	220	866
Deaths	Global	Male	60-64 years	Respiratory infections and tuberculosis	Alcohol use	Number	2019	24180	17220	30860
Deaths	Global	Female	60-64 years	Respiratory infections and tuberculosis	Alcohol use	Number	2019	3267	2027	4613
Deaths	Global	Male	60-64 years	Substance use disorders	Alcohol use	Number	2019	15804	13763	17105
Deaths	Global	Female	60-64 years	Substance use disorders	Alcohol use	Number	2019	2913	2647	3180
Deaths	Global	Male	60-64 years	Neoplasms	Alcohol use	Number	2019	58150	50309	66383
Deaths	Global	Female	60-64 years	Neoplasms	Alcohol use	Number	2019	11690	10090	13325
Deaths	Global	Male	65-69 years	Neurological disorders	Alcohol use	Number	2019	703	512	915
Deaths	Global	Female	65-69 years	Neurological disorders	Alcohol use	Number	2019	158	106	216
Deaths	Global	Male	65-69 years	Transport injuries	Alcohol use	Number	2019	2029	1135	3159

Deaths	Global	Female	65-69 years	Transport injuries	Alcohol use	Number	2019	152	75	243
Deaths	Global	Male	65-69 years	Digestive diseases	Alcohol use	Number	2019	65338	50529	79725
Deaths	Global	Female	65-69 years	Digestive diseases	Alcohol use	Number	2019	19010	13823	25008
Deaths	Global	Male	65-69 years	Self-harm and interpersonal violence	Alcohol use	Number	2019	6578	3964	9315
Deaths	Global	Female	65-69 years	Self-harm and interpersonal violence	Alcohol use	Number	2019	885	432	1366
Deaths	Global	Male	65-69 years	Unintentional injuries	Alcohol use	Number	2019	3700	1728	6111
Deaths	Global	Female	65-69 years	Unintentional injuries	Alcohol use	Number	2019	549	240	959
Deaths	Global	Male	65-69 years	Respiratory infections and tuberculosis	Alcohol use	Number	2019	23472	16903	30295
Deaths	Global	Female	65-69 years	Respiratory infections and tuberculosis	Alcohol use	Number	2019	3045	1875	4403
Deaths	Global	Male	65-69 years	Substance use disorders	Alcohol use	Number	2019	10921	9425	11755
Deaths	Global	Female	65-69 years	Substance use disorders	Alcohol use	Number	2019	2258	2060	2437
Deaths	Global	Male	65-69 years	Neoplasms	Alcohol use	Number	2019	61311	53244	70201
Deaths	Global	Female	65-69 years	Neoplasms	Alcohol use	Number	2019	12709	10942	14780
Deaths	Global	Male	70-74 years	Neurological disorders	Alcohol use	Number	2019	581	429	751
Deaths	Global	Female	70-74 years	Neurological disorders	Alcohol use	Number	2019	168	109	234
Deaths	Global	Male	70-74 years	Transport injuries	Alcohol use	Number	2019	1576	876	2438
Deaths	Global	Female	70-74 years	Transport injuries	Alcohol use	Number	2019	353	177	562
Deaths	Global	Male	70-74 years	Digestive diseases	Alcohol use	Number	2019	48717	37244	60239
Deaths	Global	Female	70-74 years	Digestive diseases	Alcohol use	Number	2019	16641	11930	21713
Deaths	Global	Male	70-74 years	Self-harm and interpersonal violence	Alcohol use	Number	2019	5153	3005	7480
Deaths	Global	Female	70-74 years	Self-harm and interpersonal violence	Alcohol use	Number	2019	760	357	1187
Deaths	Global	Male	70-74 years	Unintentional injuries	Alcohol use	Number	2019	3514	1697	5772
Deaths	Global	Female	70-74 years	Unintentional injuries	Alcohol use	Number	2019	690	304	1227
Deaths	Global	Male	70-74 years	Respiratory infections and tuberculosis	Alcohol use	Number	2019	22569	16241	28808
Deaths	Global	Female	70-74 years	Respiratory infections and tuberculosis	Alcohol use	Number	2019	3339	2036	4793
Deaths	Global	Male	70-74 years	Substance use disorders	Alcohol use	Number	2019	6635	5624	7194
Deaths	Global	Female	70-74 years	Substance use disorders	Alcohol use	Number	2019	1276	1170	1367
Deaths	Global	Female	70-74 years	Neoplasms	Alcohol use	Number	2019	12681	10798	14815
Deaths	Global	Male	70-74 years	Neoplasms	Alcohol use	Number	2019	54572	46791	62884
Deaths	Global	Male	75-79 years	Neurological disorders	Alcohol use	Number	2019	503	371	654
Deaths	Global	Female	75-79 years	Neurological disorders	Alcohol use	Number	2019	159	94	220
Deaths	Global	Male	75-79 years	Transport injuries	Alcohol use	Number	2019	1982	1104	3056
Deaths	Global	Female	75-79 years	Transport injuries	Alcohol use	Number	2019	319	160	508
Deaths	Global	Male	75-79 years	Digestive diseases	Alcohol use	Number	2019	34073	26388	42511
Deaths	Global	Female	75-79 years	Digestive diseases	Alcohol use	Number	2019	13496	9811	17675
Deaths	Global	Male	75-79 years	Self-harm and interpersonal violence	Alcohol use	Number	2019	3721	2041	5496
Deaths	Global	Female	75-79 years	Self-harm and interpersonal violence	Alcohol use	Number	2019	553	261	882
Deaths	Global	Male	75-79 years	Unintentional injuries	Alcohol use	Number	2019	3220	1495	5393
Deaths	Global	Female	75-79 years	Unintentional injuries	Alcohol use	Number	2019	837	352	1486
Deaths	Global	Male	75-79 years	Respiratory infections and tuberculosis	Alcohol use	Number	2019	18012	12461	23855
Deaths	Global	Female	75-79 years	Respiratory infections and tuberculosis	Alcohol use	Number	2019	2914	1642	4447
Deaths	Global	Male	75-79 years	Substance use disorders	Alcohol use	Number	2019	4380	3635	4795
Deaths	Global	Female	75-79 years	Substance use disorders	Alcohol use	Number	2019	1138	1027	1231
Deaths	Global	Male	75-79 years	Neoplasms	Alcohol use	Number	2019	40208	34674	46280
Deaths	Global	Female	75-79 years	Neoplasms	Alcohol use	Number	2019	10791	9091	12655
Deaths	Global	Male	80-84 years	Neurological disorders	Alcohol use	Number	2019	411	296	541
Deaths	Global	Female	80-84 years	Neurological disorders	Alcohol use	Number	2019	191	95	276
Deaths	Global	Male	80-84 years	Transport injuries	Alcohol use	Number	2019	1783	980	2717
Deaths	Global	Female	80-84 years	Transport injuries	Alcohol use	Number	2019	452	227	716
Deaths	Global	Male	80-84 years	Digestive diseases	Alcohol use	Number	2019	22703	17112	29178
Deaths	Global	Female	80-84 years	Digestive diseases	Alcohol use	Number	2019	11436	8051	15468
Deaths	Global	Male	80-84 years	Self-harm and interpersonal violence	Alcohol use	Number	2019	2519	1280	3797
Deaths	Global	Female	80-84 years	Self-harm and interpersonal violence	Alcohol use	Number	2019	408	172	662
Deaths	Global	Male	80-84 years	Unintentional injuries	Alcohol use	Number	2019	3176	1503	5372
Deaths	Global	Female	80-84 years	Unintentional injuries	Alcohol use	Number	2019	1207	552	2160

Deaths	Global	Male	80-84 years	Respiratory infections and tuberculosis	Alcohol use	Number	2019	15200	9937	20428
Deaths	Global	Female	80-84 years	Respiratory infections and tuberculosis	Alcohol use	Number	2019	3214	1642	4962
Deaths	Global	Male	80-84 years	Substance use disorders	Alcohol use	Number	2019	2200	1855	2385
Deaths	Global	Female	80-84 years	Substance use disorders	Alcohol use	Number	2019	713	614	788
Deaths	Global	Male	80-84 years	Neoplasms	Alcohol use	Number	2019	27130	22815	31282
Deaths	Global	Female	80-84 years	Neoplasms	Alcohol use	Number	2019	9534	7666	11235
Deaths	Global	Male	85-89 years	Neurological disorders	Alcohol use	Number	2019	297	214	387
Deaths	Global	Female	85-89 years	Neurological disorders	Alcohol use	Number	2019	183	80	270
Deaths	Global	Male	85-89 years	Transport injuries	Alcohol use	Number	2019	965	530	1490
Deaths	Global	Female	85-89 years	Transport injuries	Alcohol use	Number	2019	284	142	455
Deaths	Global	Male	85-89 years	Digestive diseases	Alcohol use	Number	2019	13341	10233	16975
Deaths	Global	Female	85-89 years	Digestive diseases	Alcohol use	Number	2019	7829	5536	10489
Deaths	Global	Male	85-89 years	Self-harm and interpersonal violence	Alcohol use	Number	2019	1571	809	2378
Deaths	Global	Female	85-89 years	Self-harm and interpersonal violence	Alcohol use	Number	2019	283	118	469
Deaths	Global	Male	85-89 years	Unintentional injuries	Alcohol use	Number	2019	2937	1382	4910
Deaths	Global	Female	85-89 years	Unintentional injuries	Alcohol use	Number	2019	1500	701	2663
Deaths	Global	Male	85-89 years	Respiratory infections and tuberculosis	Alcohol use	Number	2019	12046	7315	16794
Deaths	Global	Female	85-89 years	Respiratory infections and tuberculosis	Alcohol use	Number	2019	3405	1459	5593
Deaths	Global	Male	85-89 years	Substance use disorders	Alcohol use	Number	2019	1067	872	1164
Deaths	Global	Female	85-89 years	Substance use disorders	Alcohol use	Number	2019	503	413	578
Deaths	Global	Male	85-89 years	Neoplasms	Alcohol use	Number	2019	15957	13212	18552
Deaths	Global	Female	85-89 years	Neoplasms	Alcohol use	Number	2019	7628	5824	9107
Deaths	Global	Male	90-94 years	Neurological disorders	Alcohol use	Number	2019	134	95	176
Deaths	Global	Female	90-94 years	Neurological disorders	Alcohol use	Number	2019	123	52	181
Deaths	Global	Male	90-94 years	Transport injuries	Alcohol use	Number	2019	292	159	446
Deaths	Global	Female	90-94 years	Transport injuries	Alcohol use	Number	2019	124	62	195
Deaths	Global	Male	90-94 years	Digestive diseases	Alcohol use	Number	2019	5002	3673	6432
Deaths	Global	Female	90-94 years	Digestive diseases	Alcohol use	Number	2019	4439	2954	6153
Deaths	Global	Male	90-94 years	Self-harm and interpersonal violence	Alcohol use	Number	2019	500	254	762
Deaths	Global	Female	90-94 years	Self-harm and interpersonal violence	Alcohol use	Number	2019	120	49	200
Deaths	Global	Male	90-94 years	Unintentional injuries	Alcohol use	Number	2019	1630	787	2780
Deaths	Global	Female	90-94 years	Unintentional injuries	Alcohol use	Number	2019	1349	606	2376
Deaths	Global	Male	90-94 years	Respiratory infections and tuberculosis	Alcohol use	Number	2019	6345	3474	9232
Deaths	Global	Female	90-94 years	Respiratory infections and tuberculosis	Alcohol use	Number	2019	2891	1035	4974
Deaths	Global	Male	90-94 years	Substance use disorders	Alcohol use	Number	2019	323	257	359
Deaths	Global	Female	90-94 years	Substance use disorders	Alcohol use	Number	2019	237	185	274
Deaths	Global	Male	90-94 years	Neoplasms	Alcohol use	Number	2019	5356	4277	6265
Deaths	Global	Female	90-94 years	Neoplasms	Alcohol use	Number	2019	4750	3474	5785
Deaths	Global	Male	95+ years	Neurological disorders	Alcohol use	Number	2019	41	28	54
Deaths	Global	Female	95+ years	Neurological disorders	Alcohol use	Number	2019	57	24	84
Deaths	Global	Male	95+ years	Transport injuries	Alcohol use	Number	2019	82	44	125
Deaths	Global	Female	95+ years	Transport injuries	Alcohol use	Number	2019	48	24	76
Deaths	Global	Male	95+ years	Self-harm and interpersonal violence	Alcohol use	Number	2019	121	65	182
Deaths	Global	Female	95+ years	Self-harm and interpersonal violence	Alcohol use	Number	2019	37	15	61
Deaths	Global	Male	95+ years	Digestive diseases	Alcohol use	Number	2019	1654	1130	2205
Deaths	Global	Female	95+ years	Digestive diseases	Alcohol use	Number	2019	2301	1363	3414
Deaths	Global	Male	95+ years	Unintentional injuries	Alcohol use	Number	2019	628	309	1058
Deaths	Global	Female	95+ years	Unintentional injuries	Alcohol use	Number	2019	819	378	1432
Deaths	Global	Male	95+ years	Respiratory infections and tuberculosis	Alcohol use	Number	2019	2521	1243	3827
Deaths	Global	Female	95+ years	Respiratory infections and tuberculosis	Alcohol use	Number	2019	1969	654	3483
Deaths	Global	Male	95+ years	Substance use disorders	Alcohol use	Number	2019	104	77	118
Deaths	Global	Female	95+ years	Substance use disorders	Alcohol use	Number	2019	104	76	121
Deaths	Global	Male	95+ years	Neoplasms	Alcohol use	Number	2019	1412	1059	1701
Deaths	Global	Female	95+ years	Neoplasms	Alcohol use	Number	2019	2220	1578	2757

S.Figure 2B Age-specific mortality rate from level 2 GBD causes attributable to alcohol use in 1990 and 2019

measure	location	sex	age	cause	risk factor	metric	year	value (per 100000)	95% UI (lower)	95% UI (upper)
Deaths	Global	Male	15-19 years	Digestive diseases	Alcohol use	Rate	1990	0.37	0.27	0.50
Deaths	Global	Female	15-19 years	Digestive diseases	Alcohol use	Rate	1990	0.13	0.09	0.18
Deaths	Global	Male	15-19 years	Neoplasms	Alcohol use	Rate	1990	0.05	0.04	0.06
Deaths	Global	Female	15-19 years	Neoplasms	Alcohol use	Rate	1990	0.01	0.01	0.02
Deaths	Global	Male	15-19 years	Neurological disorders	Alcohol use	Rate	1990	0.12	0.08	0.17
Deaths	Global	Female	15-19 years	Neurological disorders	Alcohol use	Rate	1990	0.03	0.02	0.05
Deaths	Global	Male	15-19 years	Respiratory infections and tuberculosis	Alcohol use	Rate	1990	0.49	0.30	0.70
Deaths	Global	Female	15-19 years	Respiratory infections and tuberculosis	Alcohol use	Rate	1990	0.17	0.10	0.26
Deaths	Global	Male	15-19 years	Self-harm and interpersonal violence	Alcohol use	Rate	1990	1.80	1.15	2.50
Deaths	Global	Female	15-19 years	Self-harm and interpersonal violence	Alcohol use	Rate	1990	0.24	0.13	0.37
Deaths	Global	Male	15-19 years	Substance use disorders	Alcohol use	Rate	1990	0.32	0.28	0.35
Deaths	Global	Female	15-19 years	Substance use disorders	Alcohol use	Rate	1990	0.13	0.12	0.15
Deaths	Global	Male	15-19 years	Transport injuries	Alcohol use	Rate	1990	1.62	0.87	2.49
Deaths	Global	Female	15-19 years	Transport injuries	Alcohol use	Rate	1990	0.23	0.12	0.35
Deaths	Global	Male	15-19 years	Unintentional injuries	Alcohol use	Rate	1990	0.56	0.25	0.98
Deaths	Global	Female	15-19 years	Unintentional injuries	Alcohol use	Rate	1990	0.06	0.02	0.11
Deaths	Global	Male	20-24 years	Digestive diseases	Alcohol use	Rate	1990	1.41	1.04	1.84
Deaths	Global	Female	20-24 years	Digestive diseases	Alcohol use	Rate	1990	0.29	0.22	0.39
Deaths	Global	Male	20-24 years	Neoplasms	Alcohol use	Rate	1990	0.22	0.17	0.26
Deaths	Global	Female	20-24 years	Neoplasms	Alcohol use	Rate	1990	0.05	0.04	0.06
Deaths	Global	Male	20-24 years	Neurological disorders	Alcohol use	Rate	1990	0.31	0.22	0.41
Deaths	Global	Female	20-24 years	Neurological disorders	Alcohol use	Rate	1990	0.06	0.04	0.09
Deaths	Global	Male	20-24 years	Respiratory infections and tuberculosis	Alcohol use	Rate	1990	2.27	1.51	2.99
Deaths	Global	Female	20-24 years	Respiratory infections and tuberculosis	Alcohol use	Rate	1990	0.52	0.32	0.75
Deaths	Global	Male	20-24 years	Self-harm and interpersonal violence	Alcohol use	Rate	1990	5.34	3.57	7.30
Deaths	Global	Female	20-24 years	Self-harm and interpersonal violence	Alcohol use	Rate	1990	0.53	0.30	0.80
Deaths	Global	Male	20-24 years	Substance use disorders	Alcohol use	Rate	1990	0.85	0.74	0.92
Deaths	Global	Female	20-24 years	Substance use disorders	Alcohol use	Rate	1990	0.18	0.17	0.20
Deaths	Global	Male	20-24 years	Transport injuries	Alcohol use	Rate	1990	4.98	2.73	7.29
Deaths	Global	Female	20-24 years	Transport injuries	Alcohol use	Rate	1990	0.32	0.17	0.50
Deaths	Global	Male	20-24 years	Unintentional injuries	Alcohol use	Rate	1990	1.28	0.60	2.19
Deaths	Global	Female	20-24 years	Unintentional injuries	Alcohol use	Rate	1990	0.09	0.04	0.16
Deaths	Global	Male	25-29 years	Digestive diseases	Alcohol use	Rate	1990	3.42	2.62	4.27
Deaths	Global	Female	25-29 years	Digestive diseases	Alcohol use	Rate	1990	0.58	0.42	0.77
Deaths	Global	Male	25-29 years	Neoplasms	Alcohol use	Rate	1990	0.42	0.33	0.51
Deaths	Global	Female	25-29 years	Neoplasms	Alcohol use	Rate	1990	0.14	0.12	0.16
Deaths	Global	Male	25-29 years	Neurological disorders	Alcohol use	Rate	1990	0.38	0.28	0.49
Deaths	Global	Female	25-29 years	Neurological disorders	Alcohol use	Rate	1990	0.08	0.05	0.11
Deaths	Global	Male	25-29 years	Respiratory infections and tuberculosis	Alcohol use	Rate	1990	4.26	2.95	5.58
Deaths	Global	Female	25-29 years	Respiratory infections and tuberculosis	Alcohol use	Rate	1990	0.79	0.49	1.09
Deaths	Global	Male	25-29 years	Self-harm and interpersonal violence	Alcohol use	Rate	1990	6.95	4.73	9.27
Deaths	Global	Female	25-29 years	Self-harm and interpersonal violence	Alcohol use	Rate	1990	0.67	0.39	0.98
Deaths	Global	Male	25-29 years	Substance use disorders	Alcohol use	Rate	1990	1.92	1.68	2.04
Deaths	Global	Female	25-29 years	Substance use disorders	Alcohol use	Rate	1990	0.34	0.31	0.36
Deaths	Global	Male	25-29 years	Transport injuries	Alcohol use	Rate	1990	5.03	2.82	7.36
Deaths	Global	Female	25-29 years	Transport injuries	Alcohol use	Rate	1990	0.26	0.13	0.40
Deaths	Global	Male	25-29 years	Unintentional injuries	Alcohol use	Rate	1990	1.52	0.72	2.56
Deaths	Global	Female	25-29 years	Unintentional injuries	Alcohol use	Rate	1990	0.10	0.04	0.18
Deaths	Global	Male	30-34 years	Digestive diseases	Alcohol use	Rate	1990	7.36	5.81	8.93
Deaths	Global	Female	30-34 years	Digestive diseases	Alcohol use	Rate	1990	1.16	0.89	1.47
Deaths	Global	Male	30-34 years	Neoplasms	Alcohol use	Rate	1990	1.04	0.84	1.29
Deaths	Global	Female	30-34 years	Neoplasms	Alcohol use	Rate	1990	0.42	0.37	0.48
Deaths	Global	Male	30-34 years	Neurological disorders	Alcohol use	Rate	1990	0.44	0.33	0.57
Deaths	Global	Female	30-34 years	Neurological disorders	Alcohol use	Rate	1990	0.09	0.06	0.12

Deaths	Global	Male	30-34 years	Respiratory infections and tuberculosis	Alcohol use	Rate	1990	6.35	4.33	8.24
Deaths	Global	Female	30-34 years	Respiratory infections and tuberculosis	Alcohol use	Rate	1990	0.94	0.58	1.34
Deaths	Global	Male	30-34 years	Self-harm and interpersonal violence	Alcohol use	Rate	1990	7.63	5.19	10.14
Deaths	Global	Female	30-34 years	Self-harm and interpersonal violence	Alcohol use	Rate	1990	0.78	0.44	1.16
Deaths	Global	Male	30-34 years	Substance use disorders	Alcohol use	Rate	1990	4.10	3.67	4.33
Deaths	Global	Female	30-34 years	Substance use disorders	Alcohol use	Rate	1990	0.67	0.63	0.71
Deaths	Global	Male	30-34 years	Transport injuries	Alcohol use	Rate	1990	3.64	2.00	5.51
Deaths	Global	Female	30-34 years	Transport injuries	Alcohol use	Rate	1990	0.16	0.08	0.25
Deaths	Global	Male	30-34 years	Unintentional injuries	Alcohol use	Rate	1990	1.77	0.83	3.01
Deaths	Global	Female	30-34 years	Unintentional injuries	Alcohol use	Rate	1990	0.12	0.05	0.22
Deaths	Global	Male	35-39 years	Digestive diseases	Alcohol use	Rate	1990	13.80	10.84	16.72
Deaths	Global	Female	35-39 years	Digestive diseases	Alcohol use	Rate	1990	2.04	1.54	2.60
Deaths	Global	Male	35-39 years	Neoplasms	Alcohol use	Rate	1990	2.67	2.15	3.29
Deaths	Global	Female	35-39 years	Neoplasms	Alcohol use	Rate	1990	1.00	0.86	1.14
Deaths	Global	Male	35-39 years	Neurological disorders	Alcohol use	Rate	1990	0.52	0.38	0.67
Deaths	Global	Female	35-39 years	Neurological disorders	Alcohol use	Rate	1990	0.09	0.06	0.12
Deaths	Global	Male	35-39 years	Respiratory infections and tuberculosis	Alcohol use	Rate	1990	8.82	6.08	11.38
Deaths	Global	Female	35-39 years	Respiratory infections and tuberculosis	Alcohol use	Rate	1990	1.11	0.68	1.60
Deaths	Global	Male	35-39 years	Self-harm and interpersonal violence	Alcohol use	Rate	1990	7.85	5.26	10.69
Deaths	Global	Female	35-39 years	Self-harm and interpersonal violence	Alcohol use	Rate	1990	0.87	0.47	1.33
Deaths	Global	Male	35-39 years	Substance use disorders	Alcohol use	Rate	1990	6.19	5.68	6.53
Deaths	Global	Female	35-39 years	Substance use disorders	Alcohol use	Rate	1990	1.13	1.06	1.19
Deaths	Global	Male	35-39 years	Transport injuries	Alcohol use	Rate	1990	2.81	1.55	4.28
Deaths	Global	Female	35-39 years	Transport injuries	Alcohol use	Rate	1990	0.15	0.08	0.24
Deaths	Global	Male	35-39 years	Unintentional injuries	Alcohol use	Rate	1990	2.08	0.97	3.57
Deaths	Global	Female	35-39 years	Unintentional injuries	Alcohol use	Rate	1990	0.15	0.07	0.27
Deaths	Global	Male	40-44 years	Digestive diseases	Alcohol use	Rate	1990	22.92	18.24	27.42
Deaths	Global	Female	40-44 years	Digestive diseases	Alcohol use	Rate	1990	3.67	2.77	4.55
Deaths	Global	Male	40-44 years	Neoplasms	Alcohol use	Rate	1990	6.39	5.34	7.64
Deaths	Global	Female	40-44 years	Neoplasms	Alcohol use	Rate	1990	2.11	1.83	2.42
Deaths	Global	Male	40-44 years	Neurological disorders	Alcohol use	Rate	1990	0.56	0.42	0.72
Deaths	Global	Female	40-44 years	Neurological disorders	Alcohol use	Rate	1990	0.10	0.07	0.14
Deaths	Global	Male	40-44 years	Respiratory infections and tuberculosis	Alcohol use	Rate	1990	12.29	8.57	15.74
Deaths	Global	Female	40-44 years	Respiratory infections and tuberculosis	Alcohol use	Rate	1990	1.36	0.85	1.97
Deaths	Global	Male	40-44 years	Self-harm and interpersonal violence	Alcohol use	Rate	1990	8.07	5.41	10.87
Deaths	Global	Female	40-44 years	Self-harm and interpersonal violence	Alcohol use	Rate	1990	1.03	0.55	1.54
Deaths	Global	Male	40-44 years	Substance use disorders	Alcohol use	Rate	1990	8.33	7.60	8.81
Deaths	Global	Female	40-44 years	Substance use disorders	Alcohol use	Rate	1990	1.55	1.48	1.62
Deaths	Global	Male	40-44 years	Transport injuries	Alcohol use	Rate	1990	2.65	1.45	4.05
Deaths	Global	Female	40-44 years	Transport injuries	Alcohol use	Rate	1990	0.28	0.14	0.44
Deaths	Global	Male	40-44 years	Unintentional injuries	Alcohol use	Rate	1990	2.38	1.10	4.01
Deaths	Global	Female	40-44 years	Unintentional injuries	Alcohol use	Rate	1990	0.19	0.08	0.33
Deaths	Global	Male	45-49 years	Digestive diseases	Alcohol use	Rate	1990	32.38	25.48	39.13
Deaths	Global	Female	45-49 years	Digestive diseases	Alcohol use	Rate	1990	5.74	4.30	7.24
Deaths	Global	Male	45-49 years	Neoplasms	Alcohol use	Rate	1990	12.04	10.29	13.96
Deaths	Global	Female	45-49 years	Neoplasms	Alcohol use	Rate	1990	3.64	3.14	4.14
Deaths	Global	Male	45-49 years	Neurological disorders	Alcohol use	Rate	1990	0.57	0.42	0.72
Deaths	Global	Female	45-49 years	Neurological disorders	Alcohol use	Rate	1990	0.10	0.07	0.14
Deaths	Global	Male	45-49 years	Respiratory infections and tuberculosis	Alcohol use	Rate	1990	17.35	11.76	22.68
Deaths	Global	Female	45-49 years	Respiratory infections and tuberculosis	Alcohol use	Rate	1990	1.78	1.09	2.50
Deaths	Global	Male	45-49 years	Self-harm and interpersonal violence	Alcohol use	Rate	1990	8.06	5.27	10.86
Deaths	Global	Female	45-49 years	Self-harm and interpersonal violence	Alcohol use	Rate	1990	1.12	0.59	1.68
Deaths	Global	Male	45-49 years	Substance use disorders	Alcohol use	Rate	1990	10.17	9.27	10.87
Deaths	Global	Female	45-49 years	Substance use disorders	Alcohol use	Rate	1990	1.91	1.82	1.99
Deaths	Global	Male	45-49 years	Transport injuries	Alcohol use	Rate	1990	3.08	1.69	4.69
Deaths	Global	Female	45-49 years	Transport injuries	Alcohol use	Rate	1990	0.22	0.11	0.33
Deaths	Global	Male	45-49 years	Unintentional injuries	Alcohol use	Rate	1990	2.58	1.19	4.37
Deaths	Global	Female	45-49 years	Unintentional injuries	Alcohol use	Rate	1990	0.22	0.10	0.40
Deaths	Global	Male	50-54 years	Digestive diseases	Alcohol use	Rate	1990	43.57	34.39	52.08
Deaths	Global	Female	50-54 years	Digestive diseases	Alcohol use	Rate	1990	8.72	6.45	11.13

Deaths	Global	Male	50-54 years	Neoplasms	Alcohol use	Rate	1990	21.61	18.61	24.65
Deaths	Global	Female	50-54 years	Neoplasms	Alcohol use	Rate	1990	5.72	4.94	6.52
Deaths	Global	Male	50-54 years	Neurological disorders	Alcohol use	Rate	1990	0.53	0.40	0.68
Deaths	Global	Female	50-54 years	Neurological disorders	Alcohol use	Rate	1990	0.10	0.07	0.14
Deaths	Global	Male	50-54 years	Respiratory infections and tuberculosis	Alcohol use	Rate	1990	22.32	15.47	28.74
Deaths	Global	Female	50-54 years	Respiratory infections and tuberculosis	Alcohol use	Rate	1990	2.55	1.55	3.67
Deaths	Global	Male	50-54 years	Self-harm and interpersonal violence	Alcohol use	Rate	1990	8.73	5.64	11.75
Deaths	Global	Female	50-54 years	Self-harm and interpersonal violence	Alcohol use	Rate	1990	1.26	0.63	1.92
Deaths	Global	Male	50-54 years	Substance use disorders	Alcohol use	Rate	1990	12.53	11.45	13.16
Deaths	Global	Female	50-54 years	Substance use disorders	Alcohol use	Rate	1990	2.81	2.67	2.92
Deaths	Global	Male	50-54 years	Transport injuries	Alcohol use	Rate	1990	2.69	1.48	4.11
Deaths	Global	Female	50-54 years	Transport injuries	Alcohol use	Rate	1990	0.26	0.14	0.41
Deaths	Global	Male	50-54 years	Unintentional injuries	Alcohol use	Rate	1990	3.14	1.47	5.33
Deaths	Global	Female	50-54 years	Unintentional injuries	Alcohol use	Rate	1990	0.31	0.13	0.56
Deaths	Global	Male	55-59 years	Digestive diseases	Alcohol use	Rate	1990	54.04	42.63	65.48
Deaths	Global	Female	55-59 years	Digestive diseases	Alcohol use	Rate	1990	12.57	9.47	16.18
Deaths	Global	Male	55-59 years	Neoplasms	Alcohol use	Rate	1990	32.70	28.52	37.46
Deaths	Global	Female	55-59 years	Neoplasms	Alcohol use	Rate	1990	8.04	7.02	9.15
Deaths	Global	Male	55-59 years	Neurological disorders	Alcohol use	Rate	1990	0.53	0.39	0.68
Deaths	Global	Female	55-59 years	Neurological disorders	Alcohol use	Rate	1990	0.10	0.07	0.13
Deaths	Global	Male	55-59 years	Respiratory infections and tuberculosis	Alcohol use	Rate	1990	27.44	18.90	35.54
Deaths	Global	Female	55-59 years	Respiratory infections and tuberculosis	Alcohol use	Rate	1990	3.25	2.04	4.66
Deaths	Global	Male	55-59 years	Self-harm and interpersonal violence	Alcohol use	Rate	1990	8.09	5.06	11.12
Deaths	Global	Female	55-59 years	Self-harm and interpersonal violence	Alcohol use	Rate	1990	1.24	0.61	1.93
Deaths	Global	Male	55-59 years	Substance use disorders	Alcohol use	Rate	1990	12.32	11.21	13.02
Deaths	Global	Female	55-59 years	Substance use disorders	Alcohol use	Rate	1990	2.70	2.58	2.80
Deaths	Global	Male	55-59 years	Transport injuries	Alcohol use	Rate	1990	2.93	1.60	4.47
Deaths	Global	Female	55-59 years	Transport injuries	Alcohol use	Rate	1990	0.35	0.18	0.54
Deaths	Global	Male	55-59 years	Unintentional injuries	Alcohol use	Rate	1990	3.14	1.47	5.29
Deaths	Global	Female	55-59 years	Unintentional injuries	Alcohol use	Rate	1990	0.34	0.15	0.60
Deaths	Global	Male	60-64 years	Digestive diseases	Alcohol use	Rate	1990	63.59	50.42	76.91
Deaths	Global	Female	60-64 years	Digestive diseases	Alcohol use	Rate	1990	16.52	12.46	21.36
Deaths	Global	Male	60-64 years	Neoplasms	Alcohol use	Rate	1990	44.16	38.73	49.90
Deaths	Global	Female	60-64 years	Neoplasms	Alcohol use	Rate	1990	10.85	9.46	12.32
Deaths	Global	Male	60-64 years	Neurological disorders	Alcohol use	Rate	1990	0.56	0.40	0.72
Deaths	Global	Female	60-64 years	Neurological disorders	Alcohol use	Rate	1990	0.10	0.07	0.14
Deaths	Global	Male	60-64 years	Respiratory infections and tuberculosis	Alcohol use	Rate	1990	33.83	23.54	43.76
Deaths	Global	Female	60-64 years	Respiratory infections and tuberculosis	Alcohol use	Rate	1990	3.91	2.45	5.52
Deaths	Global	Male	60-64 years	Self-harm and interpersonal violence	Alcohol use	Rate	1990	7.86	4.87	10.98
Deaths	Global	Female	60-64 years	Self-harm and interpersonal violence	Alcohol use	Rate	1990	1.30	0.64	2.01
Deaths	Global	Male	60-64 years	Substance use disorders	Alcohol use	Rate	1990	11.52	10.47	12.13
Deaths	Global	Female	60-64 years	Substance use disorders	Alcohol use	Rate	1990	2.65	2.56	2.74
Deaths	Global	Male	60-64 years	Transport injuries	Alcohol use	Rate	1990	2.50	1.37	3.81
Deaths	Global	Female	60-64 years	Transport injuries	Alcohol use	Rate	1990	0.25	0.13	0.39
Deaths	Global	Male	60-64 years	Unintentional injuries	Alcohol use	Rate	1990	3.41	1.60	5.74
Deaths	Global	Female	60-64 years	Unintentional injuries	Alcohol use	Rate	1990	0.45	0.20	0.81
Deaths	Global	Male	65-69 years	Digestive diseases	Alcohol use	Rate	1990	70.92	56.59	84.94
Deaths	Global	Female	65-69 years	Digestive diseases	Alcohol use	Rate	1990	20.21	15.20	25.59
Deaths	Global	Male	65-69 years	Neoplasms	Alcohol use	Rate	1990	55.63	48.93	63.05
Deaths	Global	Female	65-69 years	Neoplasms	Alcohol use	Rate	1990	14.45	12.45	16.51
Deaths	Global	Male	65-69 years	Neurological disorders	Alcohol use	Rate	1990	0.59	0.43	0.77
Deaths	Global	Female	65-69 years	Neurological disorders	Alcohol use	Rate	1990	0.12	0.08	0.17
Deaths	Global	Male	65-69 years	Respiratory infections and tuberculosis	Alcohol use	Rate	1990	41.40	28.97	53.06
Deaths	Global	Female	65-69 years	Respiratory infections and tuberculosis	Alcohol use	Rate	1990	4.64	2.91	6.64
Deaths	Global	Male	65-69 years	Self-harm and interpersonal violence	Alcohol use	Rate	1990	7.67	4.54	10.93
Deaths	Global	Female	65-69 years	Self-harm and interpersonal violence	Alcohol use	Rate	1990	1.40	0.65	2.20
Deaths	Global	Male	65-69 years	Substance use disorders	Alcohol use	Rate	1990	9.04	8.31	9.51
Deaths	Global	Female	65-69 years	Substance use disorders	Alcohol use	Rate	1990	2.33	2.23	2.42
Deaths	Global	Male	65-69 years	Transport injuries	Alcohol use	Rate	1990	2.59	1.40	3.93
Deaths	Global	Female	65-69 years	Transport injuries	Alcohol use	Rate	1990	0.25	0.13	0.38

Deaths	Global	Male	65-69 years	Unintentional injuries	Alcohol use	Rate	1990	3.49	1.62	5.88
Deaths	Global	Female	65-69 years	Unintentional injuries	Alcohol use	Rate	1990	0.59	0.26	1.06
Deaths	Global	Male	70-74 years	Digestive diseases	Alcohol use	Rate	1990	72.62	56.88	88.59
Deaths	Global	Female	70-74 years	Digestive diseases	Alcohol use	Rate	1990	23.35	17.75	29.79
Deaths	Global	Male	70-74 years	Neoplasms	Alcohol use	Rate	1990	64.35	55.62	73.72
Deaths	Global	Female	70-74 years	Neoplasms	Alcohol use	Rate	1990	17.74	15.18	20.51
Deaths	Global	Male	70-74 years	Neurological disorders	Alcohol use	Rate	1990	0.64	0.47	0.83
Deaths	Global	Female	70-74 years	Neurological disorders	Alcohol use	Rate	1990	0.15	0.10	0.21
Deaths	Global	Male	70-74 years	Respiratory infections and tuberculosis	Alcohol use	Rate	1990	55.25	38.53	72.64
Deaths	Global	Female	70-74 years	Respiratory infections and tuberculosis	Alcohol use	Rate	1990	7.16	4.64	10.26
Deaths	Global	Male	70-74 years	Self-harm and interpersonal violence	Alcohol use	Rate	1990	8.29	4.80	11.99
Deaths	Global	Female	70-74 years	Self-harm and interpersonal violence	Alcohol use	Rate	1990	1.55	0.68	2.50
Deaths	Global	Male	70-74 years	Substance use disorders	Alcohol use	Rate	1990	7.42	6.63	7.90
Deaths	Global	Female	70-74 years	Substance use disorders	Alcohol use	Rate	1990	1.67	1.59	1.74
Deaths	Global	Male	70-74 years	Transport injuries	Alcohol use	Rate	1990	3.19	1.74	4.88
Deaths	Global	Female	70-74 years	Transport injuries	Alcohol use	Rate	1990	0.72	0.37	1.14
Deaths	Global	Male	70-74 years	Unintentional injuries	Alcohol use	Rate	1990	4.20	1.98	7.18
Deaths	Global	Female	70-74 years	Unintentional injuries	Alcohol use	Rate	1990	0.93	0.42	1.65
Deaths	Global	Male	75-79 years	Digestive diseases	Alcohol use	Rate	1990	79.95	64.29	96.20
Deaths	Global	Female	75-79 years	Digestive diseases	Alcohol use	Rate	1990	26.96	20.52	34.17
Deaths	Global	Male	75-79 years	Neoplasms	Alcohol use	Rate	1990	73.52	64.53	82.99
Deaths	Global	Female	75-79 years	Neoplasms	Alcohol use	Rate	1990	22.25	19.08	25.28
Deaths	Global	Male	75-79 years	Neurological disorders	Alcohol use	Rate	1990	0.79	0.57	1.02
Deaths	Global	Female	75-79 years	Neurological disorders	Alcohol use	Rate	1990	0.19	0.12	0.27
Deaths	Global	Male	75-79 years	Respiratory infections and tuberculosis	Alcohol use	Rate	1990	62.45	44.31	80.85
Deaths	Global	Female	75-79 years	Respiratory infections and tuberculosis	Alcohol use	Rate	1990	8.44	4.87	12.33
Deaths	Global	Male	75-79 years	Self-harm and interpersonal violence	Alcohol use	Rate	1990	10.24	5.70	14.97
Deaths	Global	Female	75-79 years	Self-harm and interpersonal violence	Alcohol use	Rate	1990	1.78	0.79	2.86
Deaths	Global	Male	75-79 years	Substance use disorders	Alcohol use	Rate	1990	8.05	7.13	8.57
Deaths	Global	Female	75-79 years	Substance use disorders	Alcohol use	Rate	1990	2.11	1.97	2.24
Deaths	Global	Male	75-79 years	Transport injuries	Alcohol use	Rate	1990	5.86	3.21	8.73
Deaths	Global	Female	75-79 years	Transport injuries	Alcohol use	Rate	1990	0.90	0.47	1.42
Deaths	Global	Male	75-79 years	Unintentional injuries	Alcohol use	Rate	1990	6.18	2.86	10.54
Deaths	Global	Female	75-79 years	Unintentional injuries	Alcohol use	Rate	1990	1.86	0.82	3.25
Deaths	Global	Male	80-84 years	Digestive diseases	Alcohol use	Rate	1990	82.14	64.58	100.11
Deaths	Global	Female	80-84 years	Digestive diseases	Alcohol use	Rate	1990	31.36	23.37	40.15
Deaths	Global	Male	80-84 years	Neoplasms	Alcohol use	Rate	1990	74.55	64.49	84.81
Deaths	Global	Female	80-84 years	Neoplasms	Alcohol use	Rate	1990	26.44	22.26	30.64
Deaths	Global	Male	80-84 years	Neurological disorders	Alcohol use	Rate	1990	0.87	0.62	1.13
Deaths	Global	Female	80-84 years	Neurological disorders	Alcohol use	Rate	1990	0.26	0.17	0.40
Deaths	Global	Male	80-84 years	Respiratory infections and tuberculosis	Alcohol use	Rate	1990	76.03	51.71	100.17
Deaths	Global	Female	80-84 years	Respiratory infections and tuberculosis	Alcohol use	Rate	1990	13.01	6.96	19.07
Deaths	Global	Male	80-84 years	Self-harm and interpersonal violence	Alcohol use	Rate	1990	11.31	5.92	16.96
Deaths	Global	Female	80-84 years	Self-harm and interpersonal violence	Alcohol use	Rate	1990	1.84	0.76	3.00
Deaths	Global	Male	80-84 years	Substance use disorders	Alcohol use	Rate	1990	6.43	5.85	6.81
Deaths	Global	Female	80-84 years	Substance use disorders	Alcohol use	Rate	1990	1.93	1.76	2.08
Deaths	Global	Male	80-84 years	Transport injuries	Alcohol use	Rate	1990	9.33	5.26	13.34
Deaths	Global	Female	80-84 years	Transport injuries	Alcohol use	Rate	1990	1.96	1.01	3.02
Deaths	Global	Male	80-84 years	Unintentional injuries	Alcohol use	Rate	1990	9.59	4.48	16.44
Deaths	Global	Female	80-84 years	Unintentional injuries	Alcohol use	Rate	1990	4.02	1.80	7.14
Deaths	Global	Male	85-89 years	Digestive diseases	Alcohol use	Rate	1990	96.00	75.90	117.03
Deaths	Global	Female	85-89 years	Digestive diseases	Alcohol use	Rate	1990	38.31	28.13	49.52
Deaths	Global	Male	85-89 years	Neoplasms	Alcohol use	Rate	1990	87.53	74.55	99.74
Deaths	Global	Female	85-89 years	Neoplasms	Alcohol use	Rate	1990	36.36	29.72	42.72
Deaths	Global	Male	85-89 years	Neurological disorders	Alcohol use	Rate	1990	1.17	0.85	1.51
Deaths	Global	Female	85-89 years	Neurological disorders	Alcohol use	Rate	1990	0.39	0.26	0.60
Deaths	Global	Male	85-89 years	Respiratory infections and tuberculosis	Alcohol use	Rate	1990	118.15	74.67	157.11
Deaths	Global	Female	85-89 years	Respiratory infections and tuberculosis	Alcohol use	Rate	1990	23.92	11.28	37.03
Deaths	Global	Male	85-89 years	Self-harm and interpersonal violence	Alcohol use	Rate	1990	14.38	7.41	21.34
Deaths	Global	Female	85-89 years	Self-harm and interpersonal violence	Alcohol use	Rate	1990	2.13	0.89	3.50

Deaths	Global	Male	85-89 years	Substance use disorders	Alcohol use	Rate	1990	7.08	6.27	7.60
Deaths	Global	Female	85-89 years	Substance use disorders	Alcohol use	Rate	1990	2.48	2.15	2.75
Deaths	Global	Male	85-89 years	Transport injuries	Alcohol use	Rate	1990	10.67	6.07	15.30
Deaths	Global	Female	85-89 years	Transport injuries	Alcohol use	Rate	1990	2.13	1.11	3.31
Deaths	Global	Male	85-89 years	Unintentional injuries	Alcohol use	Rate	1990	18.03	8.48	30.55
Deaths	Global	Female	85-89 years	Unintentional injuries	Alcohol use	Rate	1990	9.06	4.20	15.90
Deaths	Global	Male	90-94 years	Digestive diseases	Alcohol use	Rate	1990	108.39	83.34	134.03
Deaths	Global	Female	90-94 years	Digestive diseases	Alcohol use	Rate	1990	47.63	32.85	63.47
Deaths	Global	Male	90-94 years	Neoplasms	Alcohol use	Rate	1990	88.50	73.28	102.65
Deaths	Global	Female	90-94 years	Neoplasms	Alcohol use	Rate	1990	46.13	35.35	55.77
Deaths	Global	Male	90-94 years	Neurological disorders	Alcohol use	Rate	1990	1.50	1.09	1.94
Deaths	Global	Female	90-94 years	Neurological disorders	Alcohol use	Rate	1990	0.56	0.37	0.84
Deaths	Global	Male	90-94 years	Respiratory infections and tuberculosis	Alcohol use	Rate	1990	163.04	98.10	224.72
Deaths	Global	Female	90-94 years	Respiratory infections and tuberculosis	Alcohol use	Rate	1990	38.49	15.81	62.25
Deaths	Global	Male	90-94 years	Self-harm and interpersonal violence	Alcohol use	Rate	1990	13.24	6.96	19.98
Deaths	Global	Female	90-94 years	Self-harm and interpersonal violence	Alcohol use	Rate	1990	1.83	0.78	2.99
Deaths	Global	Male	90-94 years	Substance use disorders	Alcohol use	Rate	1990	6.59	5.55	7.18
Deaths	Global	Female	90-94 years	Substance use disorders	Alcohol use	Rate	1990	2.29	1.86	2.57
Deaths	Global	Male	90-94 years	Transport injuries	Alcohol use	Rate	1990	10.20	5.79	14.69
Deaths	Global	Female	90-94 years	Transport injuries	Alcohol use	Rate	1990	2.00	1.04	3.11
Deaths	Global	Male	90-94 years	Unintentional injuries	Alcohol use	Rate	1990	29.20	13.77	49.42
Deaths	Global	Female	90-94 years	Unintentional injuries	Alcohol use	Rate	1990	16.57	7.86	29.26
Deaths	Global	Male	95+ years	Digestive diseases	Alcohol use	Rate	1990	136.95	97.01	176.29
Deaths	Global	Female	95+ years	Digestive diseases	Alcohol use	Rate	1990	65.52	41.23	91.66
Deaths	Global	Male	95+ years	Neoplasms	Alcohol use	Rate	1990	88.31	70.27	104.27
Deaths	Global	Female	95+ years	Neoplasms	Alcohol use	Rate	1990	56.62	42.64	68.62
Deaths	Global	Male	95+ years	Neurological disorders	Alcohol use	Rate	1990	1.84	1.30	2.45
Deaths	Global	Female	95+ years	Neurological disorders	Alcohol use	Rate	1990	0.79	0.51	1.15
Deaths	Global	Male	95+ years	Respiratory infections and tuberculosis	Alcohol use	Rate	1990	218.57	118.28	319.14
Deaths	Global	Female	95+ years	Respiratory infections and tuberculosis	Alcohol use	Rate	1990	64.33	24.48	107.40
Deaths	Global	Male	95+ years	Self-harm and interpersonal violence	Alcohol use	Rate	1990	11.09	6.06	16.25
Deaths	Global	Female	95+ years	Self-harm and interpersonal violence	Alcohol use	Rate	1990	1.36	0.59	2.23
Deaths	Global	Male	95+ years	Substance use disorders	Alcohol use	Rate	1990	9.07	7.10	10.19
Deaths	Global	Female	95+ years	Substance use disorders	Alcohol use	Rate	1990	3.08	2.40	3.52
Deaths	Global	Male	95+ years	Transport injuries	Alcohol use	Rate	1990	11.81	6.65	16.68
Deaths	Global	Female	95+ years	Transport injuries	Alcohol use	Rate	1990	2.34	1.19	3.63
Deaths	Global	Male	95+ years	Unintentional injuries	Alcohol use	Rate	1990	40.36	19.54	69.45
Deaths	Global	Female	95+ years	Unintentional injuries	Alcohol use	Rate	1990	26.17	11.78	45.50
Deaths	Global	Male	15-19 years	Digestive diseases	Alcohol use	Rate	2019	0.38	0.26	0.53
Deaths	Global	Female	15-19 years	Digestive diseases	Alcohol use	Rate	2019	0.12	0.07	0.17
Deaths	Global	Male	15-19 years	Neoplasms	Alcohol use	Rate	2019	0.03	0.03	0.04
Deaths	Global	Female	15-19 years	Neoplasms	Alcohol use	Rate	2019	0.01	0.01	0.01
Deaths	Global	Male	15-19 years	Neurological disorders	Alcohol use	Rate	2019	0.09	0.06	0.13
Deaths	Global	Female	15-19 years	Neurological disorders	Alcohol use	Rate	2019	0.03	0.02	0.04
Deaths	Global	Male	15-19 years	Respiratory infections and tuberculosis	Alcohol use	Rate	2019	0.30	0.18	0.42
Deaths	Global	Female	15-19 years	Respiratory infections and tuberculosis	Alcohol use	Rate	2019	0.10	0.05	0.15
Deaths	Global	Male	15-19 years	Self-harm and interpersonal violence	Alcohol use	Rate	2019	1.34	0.86	1.86
Deaths	Global	Female	15-19 years	Self-harm and interpersonal violence	Alcohol use	Rate	2019	0.18	0.10	0.27
Deaths	Global	Male	15-19 years	Substance use disorders	Alcohol use	Rate	2019	0.19	0.14	0.21
Deaths	Global	Female	15-19 years	Substance use disorders	Alcohol use	Rate	2019	0.04	0.04	0.05
Deaths	Global	Male	15-19 years	Transport injuries	Alcohol use	Rate	2019	1.10	0.60	1.71
Deaths	Global	Female	15-19 years	Transport injuries	Alcohol use	Rate	2019	0.13	0.07	0.21
Deaths	Global	Male	15-19 years	Unintentional injuries	Alcohol use	Rate	2019	0.26	0.12	0.46
Deaths	Global	Female	15-19 years	Unintentional injuries	Alcohol use	Rate	2019	0.03	0.01	0.05
Deaths	Global	Male	20-24 years	Digestive diseases	Alcohol use	Rate	2019	1.56	1.12	2.08
Deaths	Global	Female	20-24 years	Digestive diseases	Alcohol use	Rate	2019	0.28	0.19	0.40
Deaths	Global	Male	20-24 years	Neoplasms	Alcohol use	Rate	2019	0.18	0.15	0.22
Deaths	Global	Female	20-24 years	Neoplasms	Alcohol use	Rate	2019	0.04	0.04	0.05
Deaths	Global	Male	20-24 years	Neurological disorders	Alcohol use	Rate	2019	0.27	0.19	0.36
Deaths	Global	Female	20-24 years	Neurological disorders	Alcohol use	Rate	2019	0.05	0.03	0.07

Deaths	Global	Male	20-24 years	Respiratory infections and tuberculosis	Alcohol use	Rate	2019	1.56	1.02	2.07
Deaths	Global	Female	20-24 years	Respiratory infections and tuberculosis	Alcohol use	Rate	2019	0.30	0.18	0.43
Deaths	Global	Male	20-24 years	Self-harm and interpersonal violence	Alcohol use	Rate	2019	4.36	2.94	5.86
Deaths	Global	Female	20-24 years	Self-harm and interpersonal violence	Alcohol use	Rate	2019	0.35	0.21	0.51
Deaths	Global	Male	20-24 years	Substance use disorders	Alcohol use	Rate	2019	0.64	0.49	0.73
Deaths	Global	Female	20-24 years	Substance use disorders	Alcohol use	Rate	2019	0.08	0.07	0.09
Deaths	Global	Male	20-24 years	Transport injuries	Alcohol use	Rate	2019	3.81	2.15	5.50
Deaths	Global	Female	20-24 years	Transport injuries	Alcohol use	Rate	2019	0.20	0.10	0.32
Deaths	Global	Male	20-24 years	Unintentional injuries	Alcohol use	Rate	2019	0.73	0.34	1.24
Deaths	Global	Female	20-24 years	Unintentional injuries	Alcohol use	Rate	2019	0.05	0.02	0.08
Deaths	Global	Male	25-29 years	Digestive diseases	Alcohol use	Rate	2019	3.71	2.79	4.68
Deaths	Global	Female	25-29 years	Digestive diseases	Alcohol use	Rate	2019	0.53	0.38	0.71
Deaths	Global	Male	25-29 years	Neoplasms	Alcohol use	Rate	2019	0.40	0.33	0.47
Deaths	Global	Female	25-29 years	Neoplasms	Alcohol use	Rate	2019	0.11	0.09	0.12
Deaths	Global	Male	25-29 years	Neurological disorders	Alcohol use	Rate	2019	0.35	0.26	0.46
Deaths	Global	Female	25-29 years	Neurological disorders	Alcohol use	Rate	2019	0.05	0.04	0.07
Deaths	Global	Male	25-29 years	Respiratory infections and tuberculosis	Alcohol use	Rate	2019	2.93	1.99	3.80
Deaths	Global	Female	25-29 years	Respiratory infections and tuberculosis	Alcohol use	Rate	2019	0.42	0.26	0.60
Deaths	Global	Male	25-29 years	Self-harm and interpersonal violence	Alcohol use	Rate	2019	5.45	3.72	7.21
Deaths	Global	Female	25-29 years	Self-harm and interpersonal violence	Alcohol use	Rate	2019	0.40	0.24	0.58
Deaths	Global	Male	25-29 years	Substance use disorders	Alcohol use	Rate	2019	1.47	1.20	1.61
Deaths	Global	Female	25-29 years	Substance use disorders	Alcohol use	Rate	2019	0.19	0.17	0.21
Deaths	Global	Male	25-29 years	Transport injuries	Alcohol use	Rate	2019	3.99	2.26	5.77
Deaths	Global	Female	25-29 years	Transport injuries	Alcohol use	Rate	2019	0.15	0.07	0.23
Deaths	Global	Male	25-29 years	Unintentional injuries	Alcohol use	Rate	2019	0.93	0.44	1.56
Deaths	Global	Female	25-29 years	Unintentional injuries	Alcohol use	Rate	2019	0.05	0.02	0.09
Deaths	Global	Male	30-34 years	Digestive diseases	Alcohol use	Rate	2019	6.98	5.37	8.59
Deaths	Global	Female	30-34 years	Digestive diseases	Alcohol use	Rate	2019	1.02	0.77	1.30
Deaths	Global	Male	30-34 years	Neoplasms	Alcohol use	Rate	2019	0.98	0.82	1.16
Deaths	Global	Female	30-34 years	Neoplasms	Alcohol use	Rate	2019	0.28	0.24	0.32
Deaths	Global	Male	30-34 years	Neurological disorders	Alcohol use	Rate	2019	0.39	0.28	0.52
Deaths	Global	Female	30-34 years	Neurological disorders	Alcohol use	Rate	2019	0.06	0.04	0.08
Deaths	Global	Male	30-34 years	Respiratory infections and tuberculosis	Alcohol use	Rate	2019	4.13	2.83	5.34
Deaths	Global	Female	30-34 years	Respiratory infections and tuberculosis	Alcohol use	Rate	2019	0.49	0.29	0.69
Deaths	Global	Male	30-34 years	Self-harm and interpersonal violence	Alcohol use	Rate	2019	5.67	3.81	7.48
Deaths	Global	Female	30-34 years	Self-harm and interpersonal violence	Alcohol use	Rate	2019	0.43	0.25	0.62
Deaths	Global	Male	30-34 years	Substance use disorders	Alcohol use	Rate	2019	3.11	2.53	3.38
Deaths	Global	Female	30-34 years	Substance use disorders	Alcohol use	Rate	2019	0.41	0.36	0.45
Deaths	Global	Male	30-34 years	Transport injuries	Alcohol use	Rate	2019	2.79	1.57	4.28
Deaths	Global	Female	30-34 years	Transport injuries	Alcohol use	Rate	2019	0.09	0.05	0.14
Deaths	Global	Male	30-34 years	Unintentional injuries	Alcohol use	Rate	2019	1.10	0.52	1.86
Deaths	Global	Female	30-34 years	Unintentional injuries	Alcohol use	Rate	2019	0.07	0.03	0.12
Deaths	Global	Male	35-39 years	Digestive diseases	Alcohol use	Rate	2019	12.53	9.64	15.41
Deaths	Global	Female	35-39 years	Digestive diseases	Alcohol use	Rate	2019	1.72	1.26	2.20
Deaths	Global	Male	35-39 years	Neoplasms	Alcohol use	Rate	2019	2.18	1.85	2.54
Deaths	Global	Female	35-39 years	Neoplasms	Alcohol use	Rate	2019	0.63	0.54	0.73
Deaths	Global	Male	35-39 years	Neurological disorders	Alcohol use	Rate	2019	0.46	0.33	0.60
Deaths	Global	Female	35-39 years	Neurological disorders	Alcohol use	Rate	2019	0.06	0.04	0.09
Deaths	Global	Male	35-39 years	Respiratory infections and tuberculosis	Alcohol use	Rate	2019	5.78	3.97	7.40
Deaths	Global	Female	35-39 years	Respiratory infections and tuberculosis	Alcohol use	Rate	2019	0.61	0.37	0.87
Deaths	Global	Male	35-39 years	Self-harm and interpersonal violence	Alcohol use	Rate	2019	5.89	4.02	7.85
Deaths	Global	Female	35-39 years	Self-harm and interpersonal violence	Alcohol use	Rate	2019	0.48	0.27	0.70
Deaths	Global	Male	35-39 years	Substance use disorders	Alcohol use	Rate	2019	4.71	3.91	5.12
Deaths	Global	Female	35-39 years	Substance use disorders	Alcohol use	Rate	2019	0.72	0.64	0.79
Deaths	Global	Male	35-39 years	Transport injuries	Alcohol use	Rate	2019	2.21	1.26	3.42
Deaths	Global	Female	35-39 years	Transport injuries	Alcohol use	Rate	2019	0.09	0.04	0.14
Deaths	Global	Male	35-39 years	Unintentional injuries	Alcohol use	Rate	2019	1.32	0.62	2.24
Deaths	Global	Female	35-39 years	Unintentional injuries	Alcohol use	Rate	2019	0.08	0.04	0.15
Deaths	Global	Male	40-44 years	Digestive diseases	Alcohol use	Rate	2019	18.89	14.61	23.11
Deaths	Global	Female	40-44 years	Digestive diseases	Alcohol use	Rate	2019	2.89	2.13	3.66

Deaths	Global	Male	40-44 years	Neoplasms	Alcohol use	Rate	2019	4.77	4.09	5.49
Deaths	Global	Female	40-44 years	Neoplasms	Alcohol use	Rate	2019	1.26	1.07	1.46
Deaths	Global	Male	40-44 years	Neurological disorders	Alcohol use	Rate	2019	0.48	0.35	0.63
Deaths	Global	Female	40-44 years	Neurological disorders	Alcohol use	Rate	2019	0.08	0.05	0.11
Deaths	Global	Male	40-44 years	Respiratory infections and tuberculosis	Alcohol use	Rate	2019	7.08	4.89	9.00
Deaths	Global	Female	40-44 years	Respiratory infections and tuberculosis	Alcohol use	Rate	2019	0.77	0.47	1.11
Deaths	Global	Male	40-44 years	Self-harm and interpersonal violence	Alcohol use	Rate	2019	5.68	3.86	7.67
Deaths	Global	Female	40-44 years	Self-harm and interpersonal violence	Alcohol use	Rate	2019	0.56	0.32	0.81
Deaths	Global	Male	40-44 years	Substance use disorders	Alcohol use	Rate	2019	6.33	5.35	6.89
Deaths	Global	Female	40-44 years	Substance use disorders	Alcohol use	Rate	2019	0.97	0.88	1.07
Deaths	Global	Male	40-44 years	Transport injuries	Alcohol use	Rate	2019	1.96	1.12	3.03
Deaths	Global	Female	40-44 years	Transport injuries	Alcohol use	Rate	2019	0.13	0.07	0.21
Deaths	Global	Male	40-44 years	Unintentional injuries	Alcohol use	Rate	2019	1.55	0.74	2.56
Deaths	Global	Female	40-44 years	Unintentional injuries	Alcohol use	Rate	2019	0.11	0.05	0.19
Deaths	Global	Male	45-49 years	Digestive diseases	Alcohol use	Rate	2019	25.25	19.67	31.06
Deaths	Global	Female	45-49 years	Digestive diseases	Alcohol use	Rate	2019	4.05	3.00	5.21
Deaths	Global	Male	45-49 years	Neoplasms	Alcohol use	Rate	2019	9.17	7.81	10.58
Deaths	Global	Female	45-49 years	Neoplasms	Alcohol use	Rate	2019	2.13	1.82	2.44
Deaths	Global	Male	45-49 years	Neurological disorders	Alcohol use	Rate	2019	0.47	0.35	0.61
Deaths	Global	Female	45-49 years	Neurological disorders	Alcohol use	Rate	2019	0.08	0.05	0.10
Deaths	Global	Male	45-49 years	Respiratory infections and tuberculosis	Alcohol use	Rate	2019	8.74	6.11	11.04
Deaths	Global	Female	45-49 years	Respiratory infections and tuberculosis	Alcohol use	Rate	2019	0.89	0.55	1.26
Deaths	Global	Male	45-49 years	Self-harm and interpersonal violence	Alcohol use	Rate	2019	5.50	3.67	7.42
Deaths	Global	Female	45-49 years	Self-harm and interpersonal violence	Alcohol use	Rate	2019	0.61	0.35	0.90
Deaths	Global	Male	45-49 years	Substance use disorders	Alcohol use	Rate	2019	8.01	6.70	8.76
Deaths	Global	Female	45-49 years	Substance use disorders	Alcohol use	Rate	2019	1.22	1.11	1.33
Deaths	Global	Male	45-49 years	Transport injuries	Alcohol use	Rate	2019	1.94	1.09	3.00
Deaths	Global	Female	45-49 years	Transport injuries	Alcohol use	Rate	2019	0.09	0.05	0.15
Deaths	Global	Male	45-49 years	Unintentional injuries	Alcohol use	Rate	2019	1.76	0.82	2.95
Deaths	Global	Female	45-49 years	Unintentional injuries	Alcohol use	Rate	2019	0.13	0.06	0.22
Deaths	Global	Male	50-54 years	Digestive diseases	Alcohol use	Rate	2019	32.64	24.86	40.13
Deaths	Global	Female	50-54 years	Digestive diseases	Alcohol use	Rate	2019	5.97	4.32	7.82
Deaths	Global	Male	50-54 years	Neoplasms	Alcohol use	Rate	2019	16.70	14.21	19.11
Deaths	Global	Female	50-54 years	Neoplasms	Alcohol use	Rate	2019	3.51	2.98	4.03
Deaths	Global	Male	50-54 years	Neurological disorders	Alcohol use	Rate	2019	0.44	0.33	0.57
Deaths	Global	Female	50-54 years	Neurological disorders	Alcohol use	Rate	2019	0.09	0.06	0.12
Deaths	Global	Male	50-54 years	Respiratory infections and tuberculosis	Alcohol use	Rate	2019	10.36	7.35	13.27
Deaths	Global	Female	50-54 years	Respiratory infections and tuberculosis	Alcohol use	Rate	2019	1.27	0.78	1.85
Deaths	Global	Male	50-54 years	Self-harm and interpersonal violence	Alcohol use	Rate	2019	5.42	3.59	7.34
Deaths	Global	Female	50-54 years	Self-harm and interpersonal violence	Alcohol use	Rate	2019	0.66	0.35	0.99
Deaths	Global	Male	50-54 years	Substance use disorders	Alcohol use	Rate	2019	8.45	7.17	9.22
Deaths	Global	Female	50-54 years	Substance use disorders	Alcohol use	Rate	2019	1.44	1.30	1.57
Deaths	Global	Male	50-54 years	Transport injuries	Alcohol use	Rate	2019	1.74	0.98	2.69
Deaths	Global	Female	50-54 years	Transport injuries	Alcohol use	Rate	2019	0.12	0.06	0.19
Deaths	Global	Male	50-54 years	Unintentional injuries	Alcohol use	Rate	2019	1.99	0.93	3.32
Deaths	Global	Female	50-54 years	Unintentional injuries	Alcohol use	Rate	2019	0.17	0.08	0.30
Deaths	Global	Male	55-59 years	Digestive diseases	Alcohol use	Rate	2019	42.19	32.35	51.70
Deaths	Global	Female	55-59 years	Digestive diseases	Alcohol use	Rate	2019	9.41	6.82	12.37
Deaths	Global	Male	55-59 years	Neoplasms	Alcohol use	Rate	2019	26.81	23.21	30.71
Deaths	Global	Female	55-59 years	Neoplasms	Alcohol use	Rate	2019	5.32	4.59	6.08
Deaths	Global	Male	55-59 years	Neurological disorders	Alcohol use	Rate	2019	0.50	0.36	0.63
Deaths	Global	Female	55-59 years	Neurological disorders	Alcohol use	Rate	2019	0.09	0.06	0.12
Deaths	Global	Male	55-59 years	Respiratory infections and tuberculosis	Alcohol use	Rate	2019	13.50	9.39	17.09
Deaths	Global	Female	55-59 years	Respiratory infections and tuberculosis	Alcohol use	Rate	2019	1.66	1.05	2.32
Deaths	Global	Male	55-59 years	Self-harm and interpersonal violence	Alcohol use	Rate	2019	5.77	3.67	7.90
Deaths	Global	Female	55-59 years	Self-harm and interpersonal violence	Alcohol use	Rate	2019	0.69	0.37	1.03
Deaths	Global	Male	55-59 years	Substance use disorders	Alcohol use	Rate	2019	10.41	9.02	11.25
Deaths	Global	Female	55-59 years	Substance use disorders	Alcohol use	Rate	2019	1.80	1.63	1.98
Deaths	Global	Male	55-59 years	Transport injuries	Alcohol use	Rate	2019	1.90	1.07	2.95
Deaths	Global	Female	55-59 years	Transport injuries	Alcohol use	Rate	2019	0.17	0.09	0.27

Deaths	Global	Male	55-59 years	Unintentional injuries	Alcohol use	Rate	2019	2.30	1.09	3.84
Deaths	Global	Female	55-59 years	Unintentional injuries	Alcohol use	Rate	2019	0.22	0.10	0.38
Deaths	Global	Male	60-64 years	Digestive diseases	Alcohol use	Rate	2019	49.92	38.39	61.07
Deaths	Global	Female	60-64 years	Digestive diseases	Alcohol use	Rate	2019	12.42	9.17	16.25
Deaths	Global	Male	60-64 years	Neoplasms	Alcohol use	Rate	2019	38.22	33.06	43.63
Deaths	Global	Female	60-64 years	Neoplasms	Alcohol use	Rate	2019	7.29	6.29	8.31
Deaths	Global	Male	60-64 years	Neurological disorders	Alcohol use	Rate	2019	0.52	0.38	0.67
Deaths	Global	Female	60-64 years	Neurological disorders	Alcohol use	Rate	2019	0.10	0.07	0.13
Deaths	Global	Male	60-64 years	Respiratory infections and tuberculosis	Alcohol use	Rate	2019	15.89	11.32	20.28
Deaths	Global	Female	60-64 years	Respiratory infections and tuberculosis	Alcohol use	Rate	2019	2.04	1.26	2.88
Deaths	Global	Male	60-64 years	Self-harm and interpersonal violence	Alcohol use	Rate	2019	5.46	3.41	7.51
Deaths	Global	Female	60-64 years	Self-harm and interpersonal violence	Alcohol use	Rate	2019	0.67	0.34	1.03
Deaths	Global	Male	60-64 years	Substance use disorders	Alcohol use	Rate	2019	10.39	9.05	11.24
Deaths	Global	Female	60-64 years	Substance use disorders	Alcohol use	Rate	2019	1.82	1.65	1.98
Deaths	Global	Male	60-64 years	Transport injuries	Alcohol use	Rate	2019	1.74	0.98	2.72
Deaths	Global	Female	60-64 years	Transport injuries	Alcohol use	Rate	2019	0.12	0.06	0.18
Deaths	Global	Male	60-64 years	Unintentional injuries	Alcohol use	Rate	2019	2.67	1.24	4.39
Deaths	Global	Female	60-64 years	Unintentional injuries	Alcohol use	Rate	2019	0.31	0.14	0.54
Deaths	Global	Male	65-69 years	Digestive diseases	Alcohol use	Rate	2019	52.86	40.88	64.49
Deaths	Global	Female	65-69 years	Digestive diseases	Alcohol use	Rate	2019	14.09	10.24	18.53
Deaths	Global	Male	65-69 years	Neoplasms	Alcohol use	Rate	2019	49.60	43.07	56.79
Deaths	Global	Female	65-69 years	Neoplasms	Alcohol use	Rate	2019	9.42	8.11	10.95
Deaths	Global	Male	65-69 years	Neurological disorders	Alcohol use	Rate	2019	0.57	0.41	0.74
Deaths	Global	Female	65-69 years	Neurological disorders	Alcohol use	Rate	2019	0.12	0.08	0.16
Deaths	Global	Male	65-69 years	Respiratory infections and tuberculosis	Alcohol use	Rate	2019	18.99	13.67	24.51
Deaths	Global	Female	65-69 years	Respiratory infections and tuberculosis	Alcohol use	Rate	2019	2.26	1.39	3.26
Deaths	Global	Male	65-69 years	Self-harm and interpersonal violence	Alcohol use	Rate	2019	5.32	3.21	7.54
Deaths	Global	Female	65-69 years	Self-harm and interpersonal violence	Alcohol use	Rate	2019	0.66	0.32	1.01
Deaths	Global	Male	65-69 years	Substance use disorders	Alcohol use	Rate	2019	8.83	7.62	9.51
Deaths	Global	Female	65-69 years	Substance use disorders	Alcohol use	Rate	2019	1.67	1.53	1.81
Deaths	Global	Male	65-69 years	Transport injuries	Alcohol use	Rate	2019	1.64	0.92	2.56
Deaths	Global	Female	65-69 years	Transport injuries	Alcohol use	Rate	2019	0.11	0.06	0.18
Deaths	Global	Male	65-69 years	Unintentional injuries	Alcohol use	Rate	2019	2.99	1.40	4.94
Deaths	Global	Female	65-69 years	Unintentional injuries	Alcohol use	Rate	2019	0.41	0.18	0.71
Deaths	Global	Male	70-74 years	Digestive diseases	Alcohol use	Rate	2019	55.30	42.27	68.37
Deaths	Global	Female	70-74 years	Digestive diseases	Alcohol use	Rate	2019	16.81	12.05	21.94
Deaths	Global	Male	70-74 years	Neoplasms	Alcohol use	Rate	2019	61.94	53.11	71.37
Deaths	Global	Female	70-74 years	Neoplasms	Alcohol use	Rate	2019	12.81	10.91	14.97
Deaths	Global	Male	70-74 years	Neurological disorders	Alcohol use	Rate	2019	0.66	0.49	0.85
Deaths	Global	Female	70-74 years	Neurological disorders	Alcohol use	Rate	2019	0.17	0.11	0.24
Deaths	Global	Male	70-74 years	Respiratory infections and tuberculosis	Alcohol use	Rate	2019	25.62	18.43	32.70
Deaths	Global	Female	70-74 years	Respiratory infections and tuberculosis	Alcohol use	Rate	2019	3.37	2.06	4.84
Deaths	Global	Male	70-74 years	Self-harm and interpersonal violence	Alcohol use	Rate	2019	5.85	3.41	8.49
Deaths	Global	Female	70-74 years	Self-harm and interpersonal violence	Alcohol use	Rate	2019	0.77	0.36	1.20
Deaths	Global	Male	70-74 years	Substance use disorders	Alcohol use	Rate	2019	7.53	6.38	8.17
Deaths	Global	Female	70-74 years	Substance use disorders	Alcohol use	Rate	2019	1.29	1.18	1.38
Deaths	Global	Male	70-74 years	Transport injuries	Alcohol use	Rate	2019	1.79	0.99	2.77
Deaths	Global	Female	70-74 years	Transport injuries	Alcohol use	Rate	2019	0.36	0.18	0.57
Deaths	Global	Male	70-74 years	Unintentional injuries	Alcohol use	Rate	2019	3.99	1.93	6.55
Deaths	Global	Female	70-74 years	Unintentional injuries	Alcohol use	Rate	2019	0.70	0.31	1.24
Deaths	Global	Male	75-79 years	Digestive diseases	Alcohol use	Rate	2019	59.56	46.13	74.31
Deaths	Global	Female	75-79 years	Digestive diseases	Alcohol use	Rate	2019	19.32	14.05	25.30
Deaths	Global	Male	75-79 years	Neoplasms	Alcohol use	Rate	2019	70.29	60.61	80.90
Deaths	Global	Female	75-79 years	Neoplasms	Alcohol use	Rate	2019	15.45	13.02	18.12
Deaths	Global	Male	75-79 years	Neurological disorders	Alcohol use	Rate	2019	0.88	0.65	1.14
Deaths	Global	Female	75-79 years	Neurological disorders	Alcohol use	Rate	2019	0.23	0.14	0.31
Deaths	Global	Male	75-79 years	Respiratory infections and tuberculosis	Alcohol use	Rate	2019	31.49	21.78	41.70
Deaths	Global	Female	75-79 years	Respiratory infections and tuberculosis	Alcohol use	Rate	2019	4.17	2.35	6.37
Deaths	Global	Male	75-79 years	Self-harm and interpersonal violence	Alcohol use	Rate	2019	6.51	3.57	9.61
Deaths	Global	Female	75-79 years	Self-harm and interpersonal violence	Alcohol use	Rate	2019	0.79	0.37	1.26

Deaths	Global	Male	75-79 years	Substance use disorders	Alcohol use	Rate	2019	7.66	6.35	8.38
Deaths	Global	Female	75-79 years	Substance use disorders	Alcohol use	Rate	2019	1.63	1.47	1.76
Deaths	Global	Male	75-79 years	Transport injuries	Alcohol use	Rate	2019	3.46	1.93	5.34
Deaths	Global	Female	75-79 years	Transport injuries	Alcohol use	Rate	2019	0.46	0.23	0.73
Deaths	Global	Male	75-79 years	Unintentional injuries	Alcohol use	Rate	2019	5.63	2.61	9.43
Deaths	Global	Female	75-79 years	Unintentional injuries	Alcohol use	Rate	2019	1.20	0.50	2.13
Deaths	Global	Male	80-84 years	Digestive diseases	Alcohol use	Rate	2019	64.43	48.56	82.80
Deaths	Global	Female	80-84 years	Digestive diseases	Alcohol use	Rate	2019	23.25	16.37	31.45
Deaths	Global	Male	80-84 years	Neoplasms	Alcohol use	Rate	2019	76.99	64.75	88.78
Deaths	Global	Female	80-84 years	Neoplasms	Alcohol use	Rate	2019	19.38	15.59	22.84
Deaths	Global	Male	80-84 years	Neurological disorders	Alcohol use	Rate	2019	1.17	0.84	1.54
Deaths	Global	Female	80-84 years	Neurological disorders	Alcohol use	Rate	2019	0.39	0.19	0.56
Deaths	Global	Male	80-84 years	Respiratory infections and tuberculosis	Alcohol use	Rate	2019	43.13	28.20	57.97
Deaths	Global	Female	80-84 years	Respiratory infections and tuberculosis	Alcohol use	Rate	2019	6.53	3.34	10.09
Deaths	Global	Male	80-84 years	Self-harm and interpersonal violence	Alcohol use	Rate	2019	7.15	3.63	10.78
Deaths	Global	Female	80-84 years	Self-harm and interpersonal violence	Alcohol use	Rate	2019	0.83	0.35	1.35
Deaths	Global	Male	80-84 years	Substance use disorders	Alcohol use	Rate	2019	6.24	5.26	6.77
Deaths	Global	Female	80-84 years	Substance use disorders	Alcohol use	Rate	2019	1.45	1.25	1.60
Deaths	Global	Male	80-84 years	Transport injuries	Alcohol use	Rate	2019	5.06	2.78	7.71
Deaths	Global	Female	80-84 years	Transport injuries	Alcohol use	Rate	2019	0.92	0.46	1.46
Deaths	Global	Male	80-84 years	Unintentional injuries	Alcohol use	Rate	2019	9.01	4.27	15.24
Deaths	Global	Female	80-84 years	Unintentional injuries	Alcohol use	Rate	2019	2.45	1.12	4.39
Deaths	Global	Male	85-89 years	Digestive diseases	Alcohol use	Rate	2019	81.93	62.85	104.25
Deaths	Global	Female	85-89 years	Digestive diseases	Alcohol use	Rate	2019	28.79	20.35	38.57
Deaths	Global	Male	85-89 years	Neoplasms	Alcohol use	Rate	2019	98.00	81.14	113.93
Deaths	Global	Female	85-89 years	Neoplasms	Alcohol use	Rate	2019	28.05	21.41	33.48
Deaths	Global	Male	85-89 years	Neurological disorders	Alcohol use	Rate	2019	1.83	1.31	2.37
Deaths	Global	Female	85-89 years	Neurological disorders	Alcohol use	Rate	2019	0.67	0.30	0.99
Deaths	Global	Male	85-89 years	Respiratory infections and tuberculosis	Alcohol use	Rate	2019	73.97	44.93	103.14
Deaths	Global	Female	85-89 years	Respiratory infections and tuberculosis	Alcohol use	Rate	2019	12.52	5.37	20.56
Deaths	Global	Male	85-89 years	Self-harm and interpersonal violence	Alcohol use	Rate	2019	9.65	4.97	14.60
Deaths	Global	Female	85-89 years	Self-harm and interpersonal violence	Alcohol use	Rate	2019	1.04	0.43	1.72
Deaths	Global	Male	85-89 years	Substance use disorders	Alcohol use	Rate	2019	6.55	5.35	7.15
Deaths	Global	Female	85-89 years	Substance use disorders	Alcohol use	Rate	2019	1.85	1.52	2.13
Deaths	Global	Male	85-89 years	Transport injuries	Alcohol use	Rate	2019	5.93	3.25	9.15
Deaths	Global	Female	85-89 years	Transport injuries	Alcohol use	Rate	2019	1.04	0.52	1.67
Deaths	Global	Male	85-89 years	Unintentional injuries	Alcohol use	Rate	2019	18.03	8.49	30.15
Deaths	Global	Female	85-89 years	Unintentional injuries	Alcohol use	Rate	2019	5.52	2.58	9.79
Deaths	Global	Male	90-94 years	Digestive diseases	Alcohol use	Rate	2019	94.24	69.20	121.18
Deaths	Global	Female	90-94 years	Digestive diseases	Alcohol use	Rate	2019	38.43	25.58	53.28
Deaths	Global	Male	90-94 years	Neoplasms	Alcohol use	Rate	2019	100.91	80.58	118.03
Deaths	Global	Female	90-94 years	Neoplasms	Alcohol use	Rate	2019	41.13	30.08	50.09
Deaths	Global	Male	90-94 years	Neurological disorders	Alcohol use	Rate	2019	2.52	1.78	3.31
Deaths	Global	Female	90-94 years	Neurological disorders	Alcohol use	Rate	2019	1.06	0.45	1.57
Deaths	Global	Male	90-94 years	Respiratory infections and tuberculosis	Alcohol use	Rate	2019	119.55	65.45	173.94
Deaths	Global	Female	90-94 years	Respiratory infections and tuberculosis	Alcohol use	Rate	2019	25.03	8.96	43.07
Deaths	Global	Male	90-94 years	Self-harm and interpersonal violence	Alcohol use	Rate	2019	9.42	4.78	14.36
Deaths	Global	Female	90-94 years	Self-harm and interpersonal violence	Alcohol use	Rate	2019	1.04	0.42	1.73
Deaths	Global	Male	90-94 years	Substance use disorders	Alcohol use	Rate	2019	6.09	4.84	6.77
Deaths	Global	Female	90-94 years	Substance use disorders	Alcohol use	Rate	2019	2.05	1.60	2.37
Deaths	Global	Male	90-94 years	Transport injuries	Alcohol use	Rate	2019	5.50	2.99	8.40
Deaths	Global	Female	90-94 years	Transport injuries	Alcohol use	Rate	2019	1.08	0.53	1.69
Deaths	Global	Male	90-94 years	Unintentional injuries	Alcohol use	Rate	2019	30.70	14.83	52.37
Deaths	Global	Female	90-94 years	Unintentional injuries	Alcohol use	Rate	2019	11.68	5.25	20.57
Deaths	Global	Male	95+ years	Digestive diseases	Alcohol use	Rate	2019	129.56	88.55	172.72
Deaths	Global	Female	95+ years	Digestive diseases	Alcohol use	Rate	2019	65.79	38.97	97.64
Deaths	Global	Male	95+ years	Neoplasms	Alcohol use	Rate	2019	110.67	82.95	133.28
Deaths	Global	Female	95+ years	Neoplasms	Alcohol use	Rate	2019	63.48	45.13	78.85
Deaths	Global	Male	95+ years	Neurological disorders	Alcohol use	Rate	2019	3.19	2.21	4.21
Deaths	Global	Female	95+ years	Neurological disorders	Alcohol use	Rate	2019	1.63	0.68	2.40

Deaths	Global	Male	95+ years	Respiratory infections and tuberculosis	Alcohol use	Rate	2019	197.51	97.35	299.81
Deaths	Global	Female	95+ years	Respiratory infections and tuberculosis	Alcohol use	Rate	2019	56.30	18.70	99.61
Deaths	Global	Male	95+ years	Self-harm and interpersonal violence	Alcohol use	Rate	2019	9.47	5.07	14.24
Deaths	Global	Female	95+ years	Self-harm and interpersonal violence	Alcohol use	Rate	2019	1.05	0.44	1.75
Deaths	Global	Male	95+ years	Substance use disorders	Alcohol use	Rate	2019	8.15	6.03	9.28
Deaths	Global	Female	95+ years	Substance use disorders	Alcohol use	Rate	2019	2.96	2.18	3.46
Deaths	Global	Male	95+ years	Transport injuries	Alcohol use	Rate	2019	6.46	3.44	9.81
Deaths	Global	Female	95+ years	Transport injuries	Alcohol use	Rate	2019	1.38	0.68	2.16
Deaths	Global	Male	95+ years	Unintentional injuries	Alcohol use	Rate	2019	49.19	24.19	82.88
Deaths	Global	Female	95+ years	Unintentional injuries	Alcohol use	Rate	2019	23.43	10.80	40.94

S.Figure 3A Trends in ASMR for MDR-TB among HIV-negative individuals caused by alcohol use, stratified by sex, 1990-2019

measure	location	sex	age	cause	risk factor	metric	year	value (per 100000)	95% UI (lower)	95% UI (upper)
Deaths	Global	Male	Age-standardized	MDR-TB	Alcohol use	Rate	1990	0.15	0.05	0.36
Deaths	Global	Female	Age-standardized	MDR-TB	Alcohol use	Rate	1990	0.02	0.01	0.04
Deaths	Global	Both	Age-standardized	MDR-TB	Alcohol use	Rate	1990	0.08	0.02	0.19
Deaths	Global	Male	Age-standardized	MDR-TB	Alcohol use	Rate	1991	0.22	0.08	0.50
Deaths	Global	Female	Age-standardized	MDR-TB	Alcohol use	Rate	1991	0.02	0.01	0.05
Deaths	Global	Both	Age-standardized	MDR-TB	Alcohol use	Rate	1991	0.12	0.04	0.26
Deaths	Global	Male	Age-standardized	MDR-TB	Alcohol use	Rate	1992	0.31	0.12	0.62
Deaths	Global	Female	Age-standardized	MDR-TB	Alcohol use	Rate	1992	0.03	0.01	0.06
Deaths	Global	Both	Age-standardized	MDR-TB	Alcohol use	Rate	1992	0.16	0.06	0.32
Deaths	Global	Male	Age-standardized	MDR-TB	Alcohol use	Rate	1993	0.39	0.17	0.74
Deaths	Global	Female	Age-standardized	MDR-TB	Alcohol use	Rate	1993	0.04	0.02	0.08
Deaths	Global	Both	Age-standardized	MDR-TB	Alcohol use	Rate	1993	0.20	0.09	0.39
Deaths	Global	Male	Age-standardized	MDR-TB	Alcohol use	Rate	1994	0.48	0.22	0.89
Deaths	Global	Female	Age-standardized	MDR-TB	Alcohol use	Rate	1994	0.05	0.02	0.09
Deaths	Global	Both	Age-standardized	MDR-TB	Alcohol use	Rate	1994	0.25	0.11	0.47
Deaths	Global	Male	Age-standardized	MDR-TB	Alcohol use	Rate	1995	0.55	0.25	1.01
Deaths	Global	Female	Age-standardized	MDR-TB	Alcohol use	Rate	1995	0.05	0.02	0.10
Deaths	Global	Both	Age-standardized	MDR-TB	Alcohol use	Rate	1995	0.29	0.13	0.53
Deaths	Global	Male	Age-standardized	MDR-TB	Alcohol use	Rate	1996	0.60	0.28	1.12
Deaths	Global	Female	Age-standardized	MDR-TB	Alcohol use	Rate	1996	0.06	0.02	0.11
Deaths	Global	Both	Age-standardized	MDR-TB	Alcohol use	Rate	1996	0.31	0.15	0.58
Deaths	Global	Male	Age-standardized	MDR-TB	Alcohol use	Rate	1997	0.62	0.29	1.15
Deaths	Global	Female	Age-standardized	MDR-TB	Alcohol use	Rate	1997	0.06	0.03	0.12
Deaths	Global	Both	Age-standardized	MDR-TB	Alcohol use	Rate	1997	0.33	0.15	0.61
Deaths	Global	Male	Age-standardized	MDR-TB	Alcohol use	Rate	1998	0.65	0.30	1.20
Deaths	Global	Female	Age-standardized	MDR-TB	Alcohol use	Rate	1998	0.06	0.03	0.12
Deaths	Global	Both	Age-standardized	MDR-TB	Alcohol use	Rate	1998	0.34	0.16	0.63
Deaths	Global	Male	Age-standardized	MDR-TB	Alcohol use	Rate	1999	0.69	0.32	1.24
Deaths	Global	Female	Age-standardized	MDR-TB	Alcohol use	Rate	1999	0.07	0.03	0.13
Deaths	Global	Both	Age-standardized	MDR-TB	Alcohol use	Rate	1999	0.36	0.17	0.66
Deaths	Global	Male	Age-standardized	MDR-TB	Alcohol use	Rate	2000	0.72	0.34	1.29
Deaths	Global	Female	Age-standardized	MDR-TB	Alcohol use	Rate	2000	0.07	0.03	0.13
Deaths	Global	Both	Age-standardized	MDR-TB	Alcohol use	Rate	2000	0.38	0.18	0.68
Deaths	Global	Male	Age-standardized	MDR-TB	Alcohol use	Rate	2001	0.73	0.35	1.29
Deaths	Global	Female	Age-standardized	MDR-TB	Alcohol use	Rate	2001	0.07	0.03	0.13
Deaths	Global	Both	Age-standardized	MDR-TB	Alcohol use	Rate	2001	0.39	0.19	0.68
Deaths	Global	Male	Age-standardized	MDR-TB	Alcohol use	Rate	2002	0.74	0.36	1.30
Deaths	Global	Female	Age-standardized	MDR-TB	Alcohol use	Rate	2002	0.07	0.03	0.14
Deaths	Global	Both	Age-standardized	MDR-TB	Alcohol use	Rate	2002	0.39	0.19	0.69
Deaths	Global	Male	Age-standardized	MDR-TB	Alcohol use	Rate	2003	0.74	0.36	1.29
Deaths	Global	Female	Age-standardized	MDR-TB	Alcohol use	Rate	2003	0.07	0.03	0.14
Deaths	Global	Both	Age-standardized	MDR-TB	Alcohol use	Rate	2003	0.39	0.19	0.69
Deaths	Global	Male	Age-standardized	MDR-TB	Alcohol use	Rate	2004	0.72	0.35	1.25
Deaths	Global	Female	Age-standardized	MDR-TB	Alcohol use	Rate	2004	0.07	0.03	0.13
Deaths	Global	Both	Age-standardized	MDR-TB	Alcohol use	Rate	2004	0.38	0.19	0.67
Deaths	Global	Male	Age-standardized	MDR-TB	Alcohol use	Rate	2005	0.72	0.35	1.25

Deaths	Global	Female	Age-standardized	MDR-TB	Alcohol use	Rate	2005	0.07	0.03	0.13
Deaths	Global	Both	Age-standardized	MDR-TB	Alcohol use	Rate	2005	0.38	0.18	0.66
Deaths	Global	Male	Age-standardized	MDR-TB	Alcohol use	Rate	2006	0.68	0.33	1.18
Deaths	Global	Female	Age-standardized	MDR-TB	Alcohol use	Rate	2006	0.07	0.03	0.13
Deaths	Global	Both	Age-standardized	MDR-TB	Alcohol use	Rate	2006	0.36	0.18	0.63
Deaths	Global	Male	Age-standardized	MDR-TB	Alcohol use	Rate	2007	0.65	0.31	1.14
Deaths	Global	Female	Age-standardized	MDR-TB	Alcohol use	Rate	2007	0.07	0.03	0.12
Deaths	Global	Both	Age-standardized	MDR-TB	Alcohol use	Rate	2007	0.35	0.17	0.61
Deaths	Global	Male	Age-standardized	MDR-TB	Alcohol use	Rate	2008	0.63	0.30	1.10
Deaths	Global	Female	Age-standardized	MDR-TB	Alcohol use	Rate	2008	0.06	0.03	0.12
Deaths	Global	Both	Age-standardized	MDR-TB	Alcohol use	Rate	2008	0.34	0.16	0.59
Deaths	Global	Male	Age-standardized	MDR-TB	Alcohol use	Rate	2009	0.60	0.28	1.06
Deaths	Global	Female	Age-standardized	MDR-TB	Alcohol use	Rate	2009	0.06	0.03	0.11
Deaths	Global	Both	Age-standardized	MDR-TB	Alcohol use	Rate	2009	0.32	0.15	0.56
Deaths	Global	Male	Age-standardized	MDR-TB	Alcohol use	Rate	2010	0.58	0.26	1.01
Deaths	Global	Female	Age-standardized	MDR-TB	Alcohol use	Rate	2010	0.06	0.02	0.10
Deaths	Global	Both	Age-standardized	MDR-TB	Alcohol use	Rate	2010	0.31	0.14	0.54
Deaths	Global	Male	Age-standardized	MDR-TB	Alcohol use	Rate	2011	0.55	0.25	1.00
Deaths	Global	Female	Age-standardized	MDR-TB	Alcohol use	Rate	2011	0.05	0.02	0.10
Deaths	Global	Both	Age-standardized	MDR-TB	Alcohol use	Rate	2011	0.29	0.13	0.54
Deaths	Global	Male	Age-standardized	MDR-TB	Alcohol use	Rate	2012	0.55	0.24	1.02
Deaths	Global	Female	Age-standardized	MDR-TB	Alcohol use	Rate	2012	0.05	0.02	0.10
Deaths	Global	Both	Age-standardized	MDR-TB	Alcohol use	Rate	2012	0.29	0.13	0.54
Deaths	Global	Male	Age-standardized	MDR-TB	Alcohol use	Rate	2013	0.53	0.23	1.01
Deaths	Global	Female	Age-standardized	MDR-TB	Alcohol use	Rate	2013	0.05	0.02	0.09
Deaths	Global	Both	Age-standardized	MDR-TB	Alcohol use	Rate	2013	0.28	0.12	0.53
Deaths	Global	Male	Age-standardized	MDR-TB	Alcohol use	Rate	2014	0.52	0.22	1.00
Deaths	Global	Female	Age-standardized	MDR-TB	Alcohol use	Rate	2014	0.05	0.02	0.09
Deaths	Global	Both	Age-standardized	MDR-TB	Alcohol use	Rate	2014	0.27	0.12	0.52
Deaths	Global	Male	Age-standardized	MDR-TB	Alcohol use	Rate	2015	0.50	0.21	0.96
Deaths	Global	Female	Age-standardized	MDR-TB	Alcohol use	Rate	2015	0.05	0.02	0.09
Deaths	Global	Both	Age-standardized	MDR-TB	Alcohol use	Rate	2015	0.27	0.11	0.50
Deaths	Global	Male	Age-standardized	MDR-TB	Alcohol use	Rate	2016	0.49	0.20	0.97
Deaths	Global	Female	Age-standardized	MDR-TB	Alcohol use	Rate	2016	0.05	0.02	0.09
Deaths	Global	Both	Age-standardized	MDR-TB	Alcohol use	Rate	2016	0.26	0.11	0.51
Deaths	Global	Male	Age-standardized	MDR-TB	Alcohol use	Rate	2017	0.48	0.19	0.96
Deaths	Global	Female	Age-standardized	MDR-TB	Alcohol use	Rate	2017	0.05	0.02	0.09
Deaths	Global	Both	Age-standardized	MDR-TB	Alcohol use	Rate	2017	0.26	0.11	0.51
Deaths	Global	Male	Age-standardized	MDR-TB	Alcohol use	Rate	2018	0.47	0.19	0.94
Deaths	Global	Female	Age-standardized	MDR-TB	Alcohol use	Rate	2018	0.05	0.02	0.09
Deaths	Global	Both	Age-standardized	MDR-TB	Alcohol use	Rate	2018	0.25	0.10	0.49
Deaths	Global	Male	Age-standardized	MDR-TB	Alcohol use	Rate	2019	0.45	0.18	0.93
Deaths	Global	Female	Age-standardized	MDR-TB	Alcohol use	Rate	2019	0.04	0.02	0.09
Deaths	Global	Both	Age-standardized	MDR-TB	Alcohol use	Rate	2019	0.24	0.10	0.49

ASMR: age-standardized mortality rate.

S.Figure 3B Trends in ASMR for MDR-TB among HIV-negative individuals caused by alcohol use, stratified by SDI, 1990-2019

measure	location	sex	age	cause	risk factor	metric	year	value (per 100000)	95% UI (lower)	95% UI (upper)
Deaths	Low SDI	Both	Age-standardized	MDR-TB	Alcohol use	Rate	1990	0.1	0.2	0.0
Deaths	High-middle SDI	Both	Age-standardized	MDR-TB	Alcohol use	Rate	1990	0.1	0.2	0.0
Deaths	Low-middle SDI	Both	Age-standardized	MDR-TB	Alcohol use	Rate	1990	0.1	0.2	0.0
Deaths	Middle SDI	Both	Age-standardized	MDR-TB	Alcohol use	Rate	1990	0.1	0.4	0.0
Deaths	High SDI	Both	Age-standardized	MDR-TB	Alcohol use	Rate	1990	0.0	0.0	0.0
Deaths	High-middle SDI	Both	Age-standardized	MDR-TB	Alcohol use	Rate	1991	0.1	0.3	0.0
Deaths	Middle SDI	Both	Age-standardized	MDR-TB	Alcohol use	Rate	1991	0.2	0.5	0.1
Deaths	Low-middle SDI	Both	Age-standardized	MDR-TB	Alcohol use	Rate	1991	0.1	0.3	0.0
Deaths	High SDI	Both	Age-standardized	MDR-TB	Alcohol use	Rate	1991	0.0	0.1	0.0
Deaths	Low SDI	Both	Age-standardized	MDR-TB	Alcohol use	Rate	1991	0.1	0.2	0.0
Deaths	High SDI	Both	Age-standardized	MDR-TB	Alcohol use	Rate	1992	0.0	0.1	0.0
Deaths	Middle SDI	Both	Age-standardized	MDR-TB	Alcohol use	Rate	1992	0.3	0.6	0.1
Deaths	Low SDI	Both	Age-standardized	MDR-TB	Alcohol use	Rate	1992	0.2	0.4	0.1
Deaths	Low-middle SDI	Both	Age-standardized	MDR-TB	Alcohol use	Rate	1992	0.2	0.4	0.0
Deaths	High-middle SDI	Both	Age-standardized	MDR-TB	Alcohol use	Rate	1992	0.2	0.4	0.1
Deaths	Low SDI	Both	Age-standardized	MDR-TB	Alcohol use	Rate	1993	0.3	0.6	0.1
Deaths	High-middle SDI	Both	Age-standardized	MDR-TB	Alcohol use	Rate	1993	0.2	0.5	0.1
Deaths	Low-middle SDI	Both	Age-standardized	MDR-TB	Alcohol use	Rate	1993	0.2	0.5	0.1
Deaths	Middle SDI	Both	Age-standardized	MDR-TB	Alcohol use	Rate	1993	0.3	0.6	0.1
Deaths	High SDI	Both	Age-standardized	MDR-TB	Alcohol use	Rate	1993	0.0	0.1	0.0
Deaths	Low-middle SDI	Both	Age-standardized	MDR-TB	Alcohol use	Rate	1994	0.3	0.7	0.1
Deaths	Middle SDI	Both	Age-standardized	MDR-TB	Alcohol use	Rate	1994	0.3	0.6	0.1
Deaths	High-middle SDI	Both	Age-standardized	MDR-TB	Alcohol use	Rate	1994	0.3	0.6	0.2
Deaths	Low SDI	Both	Age-standardized	MDR-TB	Alcohol use	Rate	1994	0.4	0.9	0.1
Deaths	High SDI	Both	Age-standardized	MDR-TB	Alcohol use	Rate	1994	0.0	0.1	0.0
Deaths	High SDI	Both	Age-standardized	MDR-TB	Alcohol use	Rate	1995	0.0	0.1	0.0
Deaths	High-middle SDI	Both	Age-standardized	MDR-TB	Alcohol use	Rate	1995	0.4	0.7	0.2
Deaths	Low SDI	Both	Age-standardized	MDR-TB	Alcohol use	Rate	1995	0.5	1.1	0.2
Deaths	Middle SDI	Both	Age-standardized	MDR-TB	Alcohol use	Rate	1995	0.4	0.6	0.2
Deaths	Low-middle SDI	Both	Age-standardized	MDR-TB	Alcohol use	Rate	1995	0.3	0.9	0.1
Deaths	High SDI	Both	Age-standardized	MDR-TB	Alcohol use	Rate	1996	0.0	0.1	0.0
Deaths	Low SDI	Both	Age-standardized	MDR-TB	Alcohol use	Rate	1996	0.6	1.4	0.2
Deaths	Middle SDI	Both	Age-standardized	MDR-TB	Alcohol use	Rate	1996	0.4	0.7	0.2
Deaths	High-middle SDI	Both	Age-standardized	MDR-TB	Alcohol use	Rate	1996	0.4	0.7	0.2
Deaths	Low-middle SDI	Both	Age-standardized	MDR-TB	Alcohol use	Rate	1996	0.4	1.0	0.1
Deaths	High-middle SDI	Both	Age-standardized	MDR-TB	Alcohol use	Rate	1997	0.4	0.7	0.2
Deaths	Middle SDI	Both	Age-standardized	MDR-TB	Alcohol use	Rate	1997	0.4	0.6	0.2
Deaths	Low-middle SDI	Both	Age-standardized	MDR-TB	Alcohol use	Rate	1997	0.4	1.1	0.1
Deaths	High SDI	Both	Age-standardized	MDR-TB	Alcohol use	Rate	1997	0.0	0.1	0.0
Deaths	Low SDI	Both	Age-standardized	MDR-TB	Alcohol use	Rate	1997	0.7	1.6	0.2
Deaths	Low-middle SDI	Both	Age-standardized	MDR-TB	Alcohol use	Rate	1998	0.5	1.2	0.2
Deaths	Low SDI	Both	Age-standardized	MDR-TB	Alcohol use	Rate	1998	0.8	1.8	0.3
Deaths	High SDI	Both	Age-standardized	MDR-TB	Alcohol use	Rate	1998	0.0	0.1	0.0
Deaths	High-middle SDI	Both	Age-standardized	MDR-TB	Alcohol use	Rate	1998	0.4	0.7	0.2
Deaths	Middle SDI	Both	Age-standardized	MDR-TB	Alcohol use	Rate	1998	0.4	0.6	0.2
Deaths	Low SDI	Both	Age-standardized	MDR-TB	Alcohol use	Rate	1999	0.9	1.9	0.3

Deaths	High SDI	Both	Age-standardized	MDR-TB	Alcohol use	Rate	1999	0.0	0.1	0.0
Deaths	High-middle SDI	Both	Age-standardized	MDR-TB	Alcohol use	Rate	1999	0.5	0.8	0.2
Deaths	Middle SDI	Both	Age-standardized	MDR-TB	Alcohol use	Rate	1999	0.4	0.7	0.2
Deaths	Low-middle SDI	Both	Age-standardized	MDR-TB	Alcohol use	Rate	1999	0.5	1.3	0.2
Deaths	Low SDI	Both	Age-standardized	MDR-TB	Alcohol use	Rate	2000	0.9	1.8	0.3
Deaths	Middle SDI	Both	Age-standardized	MDR-TB	Alcohol use	Rate	2000	0.4	0.7	0.2
Deaths	Low-middle SDI	Both	Age-standardized	MDR-TB	Alcohol use	Rate	2000	0.6	1.3	0.2
Deaths	High-middle SDI	Both	Age-standardized	MDR-TB	Alcohol use	Rate	2000	0.5	0.8	0.3
Deaths	High SDI	Both	Age-standardized	MDR-TB	Alcohol use	Rate	2000	0.0	0.1	0.0
Deaths	High SDI	Both	Age-standardized	MDR-TB	Alcohol use	Rate	2001	0.0	0.1	0.0
Deaths	Low-middle SDI	Both	Age-standardized	MDR-TB	Alcohol use	Rate	2001	0.6	1.2	0.2
Deaths	High-middle SDI	Both	Age-standardized	MDR-TB	Alcohol use	Rate	2001	0.5	0.8	0.3
Deaths	Middle SDI	Both	Age-standardized	MDR-TB	Alcohol use	Rate	2001	0.4	0.6	0.2
Deaths	Low SDI	Both	Age-standardized	MDR-TB	Alcohol use	Rate	2001	0.9	1.8	0.3
Deaths	High SDI	Both	Age-standardized	MDR-TB	Alcohol use	Rate	2002	0.0	0.1	0.0
Deaths	Low SDI	Both	Age-standardized	MDR-TB	Alcohol use	Rate	2002	0.9	1.7	0.3
Deaths	Low-middle SDI	Both	Age-standardized	MDR-TB	Alcohol use	Rate	2002	0.6	1.2	0.2
Deaths	High-middle SDI	Both	Age-standardized	MDR-TB	Alcohol use	Rate	2002	0.5	0.8	0.3
Deaths	Middle SDI	Both	Age-standardized	MDR-TB	Alcohol use	Rate	2002	0.4	0.7	0.2
Deaths	Low-middle SDI	Both	Age-standardized	MDR-TB	Alcohol use	Rate	2003	0.6	1.2	0.2
Deaths	Low SDI	Both	Age-standardized	MDR-TB	Alcohol use	Rate	2003	0.8	1.7	0.3
Deaths	Middle SDI	Both	Age-standardized	MDR-TB	Alcohol use	Rate	2003	0.4	0.7	0.2
Deaths	High-middle SDI	Both	Age-standardized	MDR-TB	Alcohol use	Rate	2003	0.5	0.9	0.3
Deaths	High SDI	Both	Age-standardized	MDR-TB	Alcohol use	Rate	2003	0.0	0.1	0.0
Deaths	High SDI	Both	Age-standardized	MDR-TB	Alcohol use	Rate	2004	0.0	0.1	0.0
Deaths	Low-middle SDI	Both	Age-standardized	MDR-TB	Alcohol use	Rate	2004	0.6	1.1	0.2
Deaths	Middle SDI	Both	Age-standardized	MDR-TB	Alcohol use	Rate	2004	0.3	0.6	0.2
Deaths	Low SDI	Both	Age-standardized	MDR-TB	Alcohol use	Rate	2004	0.8	1.7	0.3
Deaths	High-middle SDI	Both	Age-standardized	MDR-TB	Alcohol use	Rate	2004	0.5	0.8	0.3
Deaths	High SDI	Both	Age-standardized	MDR-TB	Alcohol use	Rate	2005	0.0	0.0	0.0
Deaths	Middle SDI	Both	Age-standardized	MDR-TB	Alcohol use	Rate	2005	0.3	0.6	0.1
Deaths	High-middle SDI	Both	Age-standardized	MDR-TB	Alcohol use	Rate	2005	0.5	0.9	0.3
Deaths	Low SDI	Both	Age-standardized	MDR-TB	Alcohol use	Rate	2005	0.8	1.6	0.3
Deaths	Low-middle SDI	Both	Age-standardized	MDR-TB	Alcohol use	Rate	2005	0.6	1.1	0.3
Deaths	Low SDI	Both	Age-standardized	MDR-TB	Alcohol use	Rate	2006	0.8	1.5	0.3
Deaths	Low-middle SDI	Both	Age-standardized	MDR-TB	Alcohol use	Rate	2006	0.6	1.1	0.3
Deaths	Middle SDI	Both	Age-standardized	MDR-TB	Alcohol use	Rate	2006	0.3	0.6	0.1
Deaths	High-middle SDI	Both	Age-standardized	MDR-TB	Alcohol use	Rate	2006	0.5	0.8	0.2
Deaths	High SDI	Both	Age-standardized	MDR-TB	Alcohol use	Rate	2006	0.0	0.0	0.0
Deaths	Low-middle SDI	Both	Age-standardized	MDR-TB	Alcohol use	Rate	2007	0.6	1.1	0.3
Deaths	Middle SDI	Both	Age-standardized	MDR-TB	Alcohol use	Rate	2007	0.3	0.5	0.1
Deaths	Low SDI	Both	Age-standardized	MDR-TB	Alcohol use	Rate	2007	0.8	1.5	0.3
Deaths	High SDI	Both	Age-standardized	MDR-TB	Alcohol use	Rate	2007	0.0	0.0	0.0
Deaths	High-middle SDI	Both	Age-standardized	MDR-TB	Alcohol use	Rate	2007	0.4	0.7	0.2
Deaths	High SDI	Both	Age-standardized	MDR-TB	Alcohol use	Rate	2008	0.0	0.0	0.0
Deaths	High-middle SDI	Both	Age-standardized	MDR-TB	Alcohol use	Rate	2008	0.4	0.6	0.2
Deaths	Middle SDI	Both	Age-standardized	MDR-TB	Alcohol use	Rate	2008	0.3	0.5	0.1
Deaths	Low SDI	Both	Age-standardized	MDR-TB	Alcohol use	Rate	2008	0.8	1.5	0.3
Deaths	Low-middle SDI	Both	Age-standardized	MDR-TB	Alcohol use	Rate	2008	0.6	1.2	0.2
Deaths	High-middle SDI	Both	Age-standardized	MDR-TB	Alcohol use	Rate	2009	0.3	0.5	0.2
Deaths	Middle SDI	Both	Age-standardized	MDR-TB	Alcohol use	Rate	2009	0.3	0.5	0.1

Deaths	Low SDI	Both	Age-standardized	MDR-TB	Alcohol use	Rate	2009	0.7	1.5	0.3
Deaths	High SDI	Both	Age-standardized	MDR-TB	Alcohol use	Rate	2009	0.0	0.0	0.0
Deaths	Low-middle SDI	Both	Age-standardized	MDR-TB	Alcohol use	Rate	2009	0.6	1.2	0.2
Deaths	Middle SDI	Both	Age-standardized	MDR-TB	Alcohol use	Rate	2010	0.3	0.5	0.1
Deaths	Low-middle SDI	Both	Age-standardized	MDR-TB	Alcohol use	Rate	2010	0.6	1.2	0.2
Deaths	High-middle SDI	Both	Age-standardized	MDR-TB	Alcohol use	Rate	2010	0.3	0.5	0.2
Deaths	High SDI	Both	Age-standardized	MDR-TB	Alcohol use	Rate	2010	0.0	0.0	0.0
Deaths	Low SDI	Both	Age-standardized	MDR-TB	Alcohol use	Rate	2010	0.7	1.4	0.3
Deaths	Low SDI	Both	Age-standardized	MDR-TB	Alcohol use	Rate	2011	0.7	1.4	0.3
Deaths	Low-middle SDI	Both	Age-standardized	MDR-TB	Alcohol use	Rate	2011	0.6	1.3	0.2
Deaths	High SDI	Both	Age-standardized	MDR-TB	Alcohol use	Rate	2011	0.0	0.0	0.0
Deaths	High-middle SDI	Both	Age-standardized	MDR-TB	Alcohol use	Rate	2011	0.3	0.4	0.1
Deaths	Middle SDI	Both	Age-standardized	MDR-TB	Alcohol use	Rate	2011	0.2	0.4	0.1
Deaths	High SDI	Both	Age-standardized	MDR-TB	Alcohol use	Rate	2012	0.0	0.0	0.0
Deaths	High-middle SDI	Both	Age-standardized	MDR-TB	Alcohol use	Rate	2012	0.3	0.4	0.1
Deaths	Middle SDI	Both	Age-standardized	MDR-TB	Alcohol use	Rate	2012	0.2	0.4	0.1
Deaths	Low SDI	Both	Age-standardized	MDR-TB	Alcohol use	Rate	2012	0.7	1.5	0.3
Deaths	Low-middle SDI	Both	Age-standardized	MDR-TB	Alcohol use	Rate	2012	0.6	1.3	0.2
Deaths	Middle SDI	Both	Age-standardized	MDR-TB	Alcohol use	Rate	2013	0.2	0.4	0.1
Deaths	High-middle SDI	Both	Age-standardized	MDR-TB	Alcohol use	Rate	2013	0.2	0.4	0.1
Deaths	Low-middle SDI	Both	Age-standardized	MDR-TB	Alcohol use	Rate	2013	0.6	1.4	0.2
Deaths	High SDI	Both	Age-standardized	MDR-TB	Alcohol use	Rate	2013	0.0	0.0	0.0
Deaths	Low SDI	Both	Age-standardized	MDR-TB	Alcohol use	Rate	2013	0.8	1.5	0.3
Deaths	Low SDI	Both	Age-standardized	MDR-TB	Alcohol use	Rate	2014	0.8	1.6	0.3
Deaths	Low-middle SDI	Both	Age-standardized	MDR-TB	Alcohol use	Rate	2014	0.6	1.5	0.2
Deaths	Middle SDI	Both	Age-standardized	MDR-TB	Alcohol use	Rate	2014	0.2	0.4	0.1
Deaths	High SDI	Both	Age-standardized	MDR-TB	Alcohol use	Rate	2014	0.0	0.0	0.0
Deaths	High-middle SDI	Both	Age-standardized	MDR-TB	Alcohol use	Rate	2014	0.2	0.3	0.1
Deaths	Low SDI	Both	Age-standardized	MDR-TB	Alcohol use	Rate	2015	0.8	1.6	0.3
Deaths	Low-middle SDI	Both	Age-standardized	MDR-TB	Alcohol use	Rate	2015	0.6	1.4	0.2
Deaths	High SDI	Both	Age-standardized	MDR-TB	Alcohol use	Rate	2015	0.0	0.0	0.0
Deaths	High-middle SDI	Both	Age-standardized	MDR-TB	Alcohol use	Rate	2015	0.2	0.3	0.1
Deaths	Middle SDI	Both	Age-standardized	MDR-TB	Alcohol use	Rate	2015	0.2	0.4	0.1
Deaths	High-middle SDI	Both	Age-standardized	MDR-TB	Alcohol use	Rate	2016	0.2	0.3	0.1
Deaths	High SDI	Both	Age-standardized	MDR-TB	Alcohol use	Rate	2016	0.0	0.0	0.0
Deaths	Middle SDI	Both	Age-standardized	MDR-TB	Alcohol use	Rate	2016	0.2	0.4	0.1
Deaths	Low-middle SDI	Both	Age-standardized	MDR-TB	Alcohol use	Rate	2016	0.6	1.4	0.2
Deaths	Low SDI	Both	Age-standardized	MDR-TB	Alcohol use	Rate	2016	0.8	1.5	0.3
Deaths	Middle SDI	Both	Age-standardized	MDR-TB	Alcohol use	Rate	2017	0.2	0.3	0.1
Deaths	High SDI	Both	Age-standardized	MDR-TB	Alcohol use	Rate	2017	0.0	0.0	0.0
Deaths	Low-middle SDI	Both	Age-standardized	MDR-TB	Alcohol use	Rate	2017	0.6	1.5	0.2
Deaths	High-middle SDI	Both	Age-standardized	MDR-TB	Alcohol use	Rate	2017	0.2	0.3	0.1
Deaths	Low SDI	Both	Age-standardized	MDR-TB	Alcohol use	Rate	2017	0.7	1.5	0.3
Deaths	Low SDI	Both	Age-standardized	MDR-TB	Alcohol use	Rate	2018	0.7	1.5	0.3
Deaths	High SDI	Both	Age-standardized	MDR-TB	Alcohol use	Rate	2018	0.0	0.0	0.0
Deaths	Low-middle SDI	Both	Age-standardized	MDR-TB	Alcohol use	Rate	2018	0.6	1.4	0.2
Deaths	Middle SDI	Both	Age-standardized	MDR-TB	Alcohol use	Rate	2018	0.2	0.3	0.1
Deaths	High-middle SDI	Both	Age-standardized	MDR-TB	Alcohol use	Rate	2018	0.2	0.3	0.1
Deaths	Low-middle SDI	Both	Age-standardized	MDR-TB	Alcohol use	Rate	2019	0.6	1.3	0.2
Deaths	Low SDI	Both	Age-standardized	MDR-TB	Alcohol use	Rate	2019	0.7	1.5	0.3
Deaths	High-middle SDI	Both	Age-standardized	MDR-TB	Alcohol use	Rate	2019	0.2	0.3	0.1

Deaths	High SDI	Both	Age-standardized	MDR-TB	Alcohol use	Rate	2019	0.0	0.0	0.0
Deaths	Middle SDI	Both	Age-standardized	MDR-TB	Alcohol use	Rate	2019	0.2	0.3	0.1

ASMR: age-standardized mortality rate.

S.Figure 4A Age-related variation in the mortality risk from MDR-TB among HIV-negative individuals, differentiated by SDI

label	Age (years)	location	sex	cause	measure	Rate	95%CI (lower)	95%CI (upper)
0 to 4	2.5	Global	Male	MDR-TB	Deaths	0.4	0.4	0.5
5 to 9	7.5	Global	Male	MDR-TB	Deaths	0.1	0.1	0.1
10 to 14	12.5	Global	Male	MDR-TB	Deaths	0.1	0.0	0.1
15 to 19	17.5	Global	Male	MDR-TB	Deaths	0.1	0.1	0.2
20 to 24	22.5	Global	Male	MDR-TB	Deaths	0.3	0.3	0.4
25 to 29	27.5	Global	Male	MDR-TB	Deaths	0.6	0.6	0.7
30 to 34	32.5	Global	Male	MDR-TB	Deaths	1.0	0.9	1.0
35 to 39	37.5	Global	Male	MDR-TB	Deaths	1.4	1.3	1.5
40 to 44	42.5	Global	Male	MDR-TB	Deaths	2.0	1.9	2.1
45 to 49	47.5	Global	Male	MDR-TB	Deaths	3.1	2.9	3.2
50 to 54	52.5	Global	Male	MDR-TB	Deaths	4.1	4.0	4.3
55 to 59	57.5	Global	Male	MDR-TB	Deaths	5.6	5.3	5.8
60 to 64	62.5	Global	Male	MDR-TB	Deaths	7.1	6.8	7.5
65 to 69	67.5	Global	Male	MDR-TB	Deaths	9.4	8.9	10.0
70 to 74	72.5	Global	Male	MDR-TB	Deaths	12.5	11.8	13.3
75 to 79	77.5	Global	Male	MDR-TB	Deaths	14.9	13.9	15.9
80 to 84	82.5	Global	Male	MDR-TB	Deaths	19.1	17.7	20.5
85 to 89	87.5	Global	Male	MDR-TB	Deaths	21.8	20.0	23.9
90 to 94	92.5	Global	Male	MDR-TB	Deaths	22.5	19.6	25.8
95 plus	97.5	Global	Male	MDR-TB	Deaths	20.1	15.3	26.2
0 to 4	2.5	High SDI	Male	MDR-TB	Deaths	0.0	0.0	0.1
5 to 9	7.5	High SDI	Male	MDR-TB	Deaths	0.0	0.0	0.0
10 to 14	12.5	High SDI	Male	MDR-TB	Deaths	0.0	0.0	0.0
15 to 19	17.5	High SDI	Male	MDR-TB	Deaths	0.0	0.0	0.0
20 to 24	22.5	High SDI	Male	MDR-TB	Deaths	0.0	0.0	0.1
25 to 29	27.5	High SDI	Male	MDR-TB	Deaths	0.1	0.0	0.1
30 to 34	32.5	High SDI	Male	MDR-TB	Deaths	0.1	0.0	0.1
35 to 39	37.5	High SDI	Male	MDR-TB	Deaths	0.1	0.1	0.1
40 to 44	42.5	High SDI	Male	MDR-TB	Deaths	0.1	0.1	0.1
45 to 49	47.5	High SDI	Male	MDR-TB	Deaths	0.1	0.1	0.1
50 to 54	52.5	High SDI	Male	MDR-TB	Deaths	0.1	0.1	0.1
55 to 59	57.5	High SDI	Male	MDR-TB	Deaths	0.1	0.1	0.1
60 to 64	62.5	High SDI	Male	MDR-TB	Deaths	0.1	0.1	0.1
65 to 69	67.5	High SDI	Male	MDR-TB	Deaths	0.1	0.1	0.1
70 to 74	72.5	High SDI	Male	MDR-TB	Deaths	0.1	0.1	0.1
75 to 79	77.5	High SDI	Male	MDR-TB	Deaths	0.1	0.1	0.1
80 to 84	82.5	High SDI	Male	MDR-TB	Deaths	0.1	0.1	0.1
85 to 89	87.5	High SDI	Male	MDR-TB	Deaths	0.1	0.1	0.1
90 to 94	92.5	High SDI	Male	MDR-TB	Deaths	0.1	0.1	0.1
95 plus	97.5	High SDI	Male	MDR-TB	Deaths	0.1	0.1	0.1
0 to 4	2.5	High-middle SDI	Male	MDR-TB	Deaths	0.7	0.5	0.9
5 to 9	7.5	High-middle SDI	Male	MDR-TB	Deaths	0.1	0.0	0.1
10 to 14	12.5	High-middle SDI	Male	MDR-TB	Deaths	0.1	0.0	0.1
15 to 19	17.5	High-middle SDI	Male	MDR-TB	Deaths	0.1	0.1	0.2
20 to 24	22.5	High-middle SDI	Male	MDR-TB	Deaths	0.5	0.4	0.6
25 to 29	27.5	High-middle SDI	Male	MDR-TB	Deaths	0.9	0.8	1.0

30 to 34	32.5	High-middle SDI	Male	MDR-TB	Deaths	1.2	1.1	1.4
35 to 39	37.5	High-middle SDI	Male	MDR-TB	Deaths	1.3	1.2	1.4
40 to 44	42.5	High-middle SDI	Male	MDR-TB	Deaths	1.7	1.5	1.8
45 to 49	47.5	High-middle SDI	Male	MDR-TB	Deaths	3.0	2.8	3.2
50 to 54	52.5	High-middle SDI	Male	MDR-TB	Deaths	3.2	3.0	3.5
55 to 59	57.5	High-middle SDI	Male	MDR-TB	Deaths	3.3	3.0	3.5
60 to 64	62.5	High-middle SDI	Male	MDR-TB	Deaths	2.9	2.7	3.2
65 to 69	67.5	High-middle SDI	Male	MDR-TB	Deaths	2.5	2.3	2.8
70 to 74	72.5	High-middle SDI	Male	MDR-TB	Deaths	2.4	2.1	2.7
75 to 79	77.5	High-middle SDI	Male	MDR-TB	Deaths	2.1	1.8	2.4
80 to 84	82.5	High-middle SDI	Male	MDR-TB	Deaths	1.9	1.6	2.2
85 to 89	87.5	High-middle SDI	Male	MDR-TB	Deaths	1.7	1.4	2.1
90 to 94	92.5	High-middle SDI	Male	MDR-TB	Deaths	1.4	0.9	1.9
95 plus	97.5	High-middle SDI	Male	MDR-TB	Deaths	0.9	0.4	2.0
0 to 4	2.5	China	Male	MDR-TB	Deaths	41.6	35.5	48.7
5 to 9	7.5	China	Male	MDR-TB	Deaths	2.7	2.3	3.2
10 to 14	12.5	China	Male	MDR-TB	Deaths	1.2	1.0	1.5
15 to 19	17.5	China	Male	MDR-TB	Deaths	1.3	1.2	1.5
20 to 24	22.5	China	Male	MDR-TB	Deaths	1.4	1.3	1.6
25 to 29	27.5	China	Male	MDR-TB	Deaths	1.1	1.0	1.2
30 to 34	32.5	China	Male	MDR-TB	Deaths	1.1	1.0	1.2
35 to 39	37.5	China	Male	MDR-TB	Deaths	0.9	0.9	1.0
40 to 44	42.5	China	Male	MDR-TB	Deaths	0.8	0.7	0.8
45 to 49	47.5	China	Male	MDR-TB	Deaths	0.6	0.6	0.7
50 to 54	52.5	China	Male	MDR-TB	Deaths	0.5	0.5	0.5
55 to 59	57.5	China	Male	MDR-TB	Deaths	0.4	0.4	0.5
60 to 64	62.5	China	Male	MDR-TB	Deaths	0.4	0.4	0.4
65 to 69	67.5	China	Male	MDR-TB	Deaths	0.3	0.3	0.4
70 to 74	72.5	China	Male	MDR-TB	Deaths	0.3	0.3	0.3
75 to 79	77.5	China	Male	MDR-TB	Deaths	0.3	0.2	0.3
80 to 84	82.5	China	Male	MDR-TB	Deaths	0.2	0.2	0.2
85 to 89	87.5	China	Male	MDR-TB	Deaths	0.2	0.2	0.2
90 to 94	92.5	China	Male	MDR-TB	Deaths	0.2	0.2	0.2
95 plus	97.5	China	Male	MDR-TB	Deaths	0.1	0.1	0.2
0 to 4	2.5	Low SDI	Male	MDR-TB	Deaths	0.5	0.5	0.6
5 to 9	7.5	Low SDI	Male	MDR-TB	Deaths	0.1	0.1	0.1
10 to 14	12.5	Low SDI	Male	MDR-TB	Deaths	0.1	0.1	0.1
15 to 19	17.5	Low SDI	Male	MDR-TB	Deaths	0.2	0.2	0.2
20 to 24	22.5	Low SDI	Male	MDR-TB	Deaths	0.6	0.6	0.6
25 to 29	27.5	Low SDI	Male	MDR-TB	Deaths	1.2	1.1	1.2
30 to 34	32.5	Low SDI	Male	MDR-TB	Deaths	2.0	1.9	2.1
35 to 39	37.5	Low SDI	Male	MDR-TB	Deaths	3.2	3.0	3.3
40 to 44	42.5	Low SDI	Male	MDR-TB	Deaths	4.9	4.7	5.1
45 to 49	47.5	Low SDI	Male	MDR-TB	Deaths	7.8	7.6	8.1
50 to 54	52.5	Low SDI	Male	MDR-TB	Deaths	12.8	12.4	13.3
55 to 59	57.5	Low SDI	Male	MDR-TB	Deaths	19.6	18.9	20.2
60 to 64	62.5	Low SDI	Male	MDR-TB	Deaths	26.8	25.7	27.9
65 to 69	67.5	Low SDI	Male	MDR-TB	Deaths	39.8	38.1	41.7
70 to 74	72.5	Low SDI	Male	MDR-TB	Deaths	68.4	65.1	71.8
75 to 79	77.5	Low SDI	Male	MDR-TB	Deaths	100.6	95.2	106.3
80 to 84	82.5	Low SDI	Male	MDR-TB	Deaths	186.3	175.3	198.1

85 to 89	87.5	Low SDI	Male	MDR-TB	Deaths	294.3	273.1	317.2
90 to 94	92.5	Low SDI	Male	MDR-TB	Deaths	426.1	382.3	475.0
95 plus	97.5	Low SDI	Male	MDR-TB	Deaths	565.1	454.3	702.9
0 to 4	2.5	Low-middle SDI	Male	MDR-TB	Deaths	0.3	0.2	0.3
5 to 9	7.5	Low-middle SDI	Male	MDR-TB	Deaths	0.0	0.0	0.1
10 to 14	12.5	Low-middle SDI	Male	MDR-TB	Deaths	0.0	0.0	0.1
15 to 19	17.5	Low-middle SDI	Male	MDR-TB	Deaths	0.1	0.1	0.1
20 to 24	22.5	Low-middle SDI	Male	MDR-TB	Deaths	0.3	0.3	0.3
25 to 29	27.5	Low-middle SDI	Male	MDR-TB	Deaths	0.6	0.6	0.7
30 to 34	32.5	Low-middle SDI	Male	MDR-TB	Deaths	1.2	1.1	1.2
35 to 39	37.5	Low-middle SDI	Male	MDR-TB	Deaths	2.1	2.0	2.2
40 to 44	42.5	Low-middle SDI	Male	MDR-TB	Deaths	3.5	3.3	3.6
45 to 49	47.5	Low-middle SDI	Male	MDR-TB	Deaths	5.9	5.7	6.1
50 to 54	52.5	Low-middle SDI	Male	MDR-TB	Deaths	9.4	9.1	9.7
55 to 59	57.5	Low-middle SDI	Male	MDR-TB	Deaths	15.7	15.2	16.2
60 to 64	62.5	Low-middle SDI	Male	MDR-TB	Deaths	24.2	23.3	25.2
65 to 69	67.5	Low-middle SDI	Male	MDR-TB	Deaths	38.3	36.7	39.9
70 to 74	72.5	Low-middle SDI	Male	MDR-TB	Deaths	58.1	55.5	60.9
75 to 79	77.5	Low-middle SDI	Male	MDR-TB	Deaths	84.1	79.8	88.5
80 to 84	82.5	Low-middle SDI	Male	MDR-TB	Deaths	132.4	124.9	140.3
85 to 89	87.5	Low-middle SDI	Male	MDR-TB	Deaths	179.4	166.9	192.8
90 to 94	92.5	Low-middle SDI	Male	MDR-TB	Deaths	219.1	196.5	244.3
95 plus	97.5	Low-middle SDI	Male	MDR-TB	Deaths	240.8	193.5	299.8
0 to 4	2.5	Middle SDI	Male	MDR-TB	Deaths	1.0	0.9	1.1
5 to 9	7.5	Middle SDI	Male	MDR-TB	Deaths	0.1	0.1	0.2
10 to 14	12.5	Middle SDI	Male	MDR-TB	Deaths	0.1	0.1	0.1
15 to 19	17.5	Middle SDI	Male	MDR-TB	Deaths	0.2	0.2	0.2
20 to 24	22.5	Middle SDI	Male	MDR-TB	Deaths	0.4	0.4	0.5
25 to 29	27.5	Middle SDI	Male	MDR-TB	Deaths	0.7	0.6	0.7
30 to 34	32.5	Middle SDI	Male	MDR-TB	Deaths	0.9	0.9	1.0
35 to 39	37.5	Middle SDI	Male	MDR-TB	Deaths	1.2	1.1	1.3
40 to 44	42.5	Middle SDI	Male	MDR-TB	Deaths	1.5	1.4	1.5
45 to 49	47.5	Middle SDI	Male	MDR-TB	Deaths	1.8	1.7	1.9
50 to 54	52.5	Middle SDI	Male	MDR-TB	Deaths	2.3	2.2	2.5
55 to 59	57.5	Middle SDI	Male	MDR-TB	Deaths	2.9	2.7	3.1
60 to 64	62.5	Middle SDI	Male	MDR-TB	Deaths	3.6	3.3	3.8
65 to 69	67.5	Middle SDI	Male	MDR-TB	Deaths	4.4	4.1	4.7
70 to 74	72.5	Middle SDI	Male	MDR-TB	Deaths	5.4	5.0	5.8
75 to 79	77.5	Middle SDI	Male	MDR-TB	Deaths	6.1	5.7	6.6
80 to 84	82.5	Middle SDI	Male	MDR-TB	Deaths	7.0	6.5	7.6
85 to 89	87.5	Middle SDI	Male	MDR-TB	Deaths	8.1	7.3	8.9
90 to 94	92.5	Middle SDI	Male	MDR-TB	Deaths	8.6	7.5	10.0
95 plus	97.5	Middle SDI	Male	MDR-TB	Deaths	8.1	6.2	10.5
0 to 4	2.5	Global	Female	MDR-TB	Deaths	0.6	0.5	0.6
5 to 9	7.5	Global	Female	MDR-TB	Deaths	0.1	0.1	0.1
10 to 14	12.5	Global	Female	MDR-TB	Deaths	0.1	0.1	0.1
15 to 19	17.5	Global	Female	MDR-TB	Deaths	0.2	0.2	0.2
20 to 24	22.5	Global	Female	MDR-TB	Deaths	0.3	0.3	0.3
25 to 29	27.5	Global	Female	MDR-TB	Deaths	0.4	0.4	0.4
30 to 34	32.5	Global	Female	MDR-TB	Deaths	0.5	0.5	0.6
35 to 39	37.5	Global	Female	MDR-TB	Deaths	0.7	0.6	0.7

40 to 44	42.5	Global	Female	MDR-TB	Deaths	0.8	0.8	0.9
45 to 49	47.5	Global	Female	MDR-TB	Deaths	1.1	1.0	1.2
50 to 54	52.5	Global	Female	MDR-TB	Deaths	1.7	1.6	1.8
55 to 59	57.5	Global	Female	MDR-TB	Deaths	2.3	2.2	2.4
60 to 64	62.5	Global	Female	MDR-TB	Deaths	3.1	2.9	3.3
65 to 69	67.5	Global	Female	MDR-TB	Deaths	4.2	3.9	4.5
70 to 74	72.5	Global	Female	MDR-TB	Deaths	6.1	5.8	6.6
75 to 79	77.5	Global	Female	MDR-TB	Deaths	7.8	7.2	8.3
80 to 84	82.5	Global	Female	MDR-TB	Deaths	9.9	9.2	10.7
85 to 89	87.5	Global	Female	MDR-TB	Deaths	9.5	8.7	10.4
90 to 94	92.5	Global	Female	MDR-TB	Deaths	8.0	7.0	9.1
95 plus	97.5	Global	Female	MDR-TB	Deaths	9.2	7.6	11.3
0 to 4	2.5	High SDI	Female	MDR-TB	Deaths	0.0	0.0	0.1
5 to 9	7.5	High SDI	Female	MDR-TB	Deaths	0.0	0.0	0.0
10 to 14	12.5	High SDI	Female	MDR-TB	Deaths	0.0	0.0	0.0
15 to 19	17.5	High SDI	Female	MDR-TB	Deaths	0.0	0.0	0.0
20 to 24	22.5	High SDI	Female	MDR-TB	Deaths	0.0	0.0	0.0
25 to 29	27.5	High SDI	Female	MDR-TB	Deaths	0.0	0.0	0.0
30 to 34	32.5	High SDI	Female	MDR-TB	Deaths	0.0	0.0	0.0
35 to 39	37.5	High SDI	Female	MDR-TB	Deaths	0.0	0.0	0.0
40 to 44	42.5	High SDI	Female	MDR-TB	Deaths	0.0	0.0	0.0
45 to 49	47.5	High SDI	Female	MDR-TB	Deaths	0.0	0.0	0.0
50 to 54	52.5	High SDI	Female	MDR-TB	Deaths	0.0	0.0	0.0
55 to 59	57.5	High SDI	Female	MDR-TB	Deaths	0.0	0.0	0.0
60 to 64	62.5	High SDI	Female	MDR-TB	Deaths	0.0	0.0	0.0
65 to 69	67.5	High SDI	Female	MDR-TB	Deaths	0.0	0.0	0.0
70 to 74	72.5	High SDI	Female	MDR-TB	Deaths	0.0	0.0	0.0
75 to 79	77.5	High SDI	Female	MDR-TB	Deaths	0.0	0.0	0.1
80 to 84	82.5	High SDI	Female	MDR-TB	Deaths	0.1	0.0	0.1
85 to 89	87.5	High SDI	Female	MDR-TB	Deaths	0.1	0.0	0.1
90 to 94	92.5	High SDI	Female	MDR-TB	Deaths	0.1	0.0	0.1
95 plus	97.5	High SDI	Female	MDR-TB	Deaths	0.1	0.0	0.1
0 to 4	2.5	High-middle SDI	Female	MDR-TB	Deaths	0.4	0.3	0.6
5 to 9	7.5	High-middle SDI	Female	MDR-TB	Deaths	0.1	0.0	0.1
10 to 14	12.5	High-middle SDI	Female	MDR-TB	Deaths	0.0	0.0	0.1
15 to 19	17.5	High-middle SDI	Female	MDR-TB	Deaths	0.1	0.1	0.1
20 to 24	22.5	High-middle SDI	Female	MDR-TB	Deaths	0.2	0.2	0.3
25 to 29	27.5	High-middle SDI	Female	MDR-TB	Deaths	0.3	0.3	0.3
30 to 34	32.5	High-middle SDI	Female	MDR-TB	Deaths	0.3	0.3	0.4
35 to 39	37.5	High-middle SDI	Female	MDR-TB	Deaths	0.4	0.3	0.4
40 to 44	42.5	High-middle SDI	Female	MDR-TB	Deaths	0.4	0.4	0.5
45 to 49	47.5	High-middle SDI	Female	MDR-TB	Deaths	0.6	0.5	0.6
50 to 54	52.5	High-middle SDI	Female	MDR-TB	Deaths	0.6	0.6	0.7
55 to 59	57.5	High-middle SDI	Female	MDR-TB	Deaths	0.7	0.6	0.8
60 to 64	62.5	High-middle SDI	Female	MDR-TB	Deaths	0.7	0.6	0.8
65 to 69	67.5	High-middle SDI	Female	MDR-TB	Deaths	0.7	0.6	0.8
70 to 74	72.5	High-middle SDI	Female	MDR-TB	Deaths	0.8	0.7	1.0
75 to 79	77.5	High-middle SDI	Female	MDR-TB	Deaths	0.9	0.8	1.0
80 to 84	82.5	High-middle SDI	Female	MDR-TB	Deaths	1.0	0.8	1.2
85 to 89	87.5	High-middle SDI	Female	MDR-TB	Deaths	0.9	0.7	1.0
90 to 94	92.5	High-middle SDI	Female	MDR-TB	Deaths	0.6	0.5	0.8

95 plus	97.5	High-middle SDI	Female	MDR-TB	Deaths	0.5	0.3	0.9
0 to 4	2.5	China	Female	MDR-TB	Deaths	76.7	57.8	101.7
5 to 9	7.5	China	Female	MDR-TB	Deaths	4.1	3.0	5.6
10 to 14	12.5	China	Female	MDR-TB	Deaths	2.3	1.7	3.0
15 to 19	17.5	China	Female	MDR-TB	Deaths	1.8	1.5	2.3
20 to 24	22.5	China	Female	MDR-TB	Deaths	1.8	1.5	2.1
25 to 29	27.5	China	Female	MDR-TB	Deaths	1.2	1.0	1.4
30 to 34	32.5	China	Female	MDR-TB	Deaths	0.9	0.8	1.0
35 to 39	37.5	China	Female	MDR-TB	Deaths	0.5	0.5	0.6
40 to 44	42.5	China	Female	MDR-TB	Deaths	0.3	0.3	0.4
45 to 49	47.5	China	Female	MDR-TB	Deaths	0.2	0.2	0.3
50 to 54	52.5	China	Female	MDR-TB	Deaths	0.2	0.1	0.2
55 to 59	57.5	China	Female	MDR-TB	Deaths	0.1	0.1	0.1
60 to 64	62.5	China	Female	MDR-TB	Deaths	0.1	0.1	0.1
65 to 69	67.5	China	Female	MDR-TB	Deaths	0.1	0.1	0.1
70 to 74	72.5	China	Female	MDR-TB	Deaths	0.1	0.1	0.1
75 to 79	77.5	China	Female	MDR-TB	Deaths	0.1	0.0	0.1
80 to 84	82.5	China	Female	MDR-TB	Deaths	0.0	0.0	0.0
85 to 89	87.5	China	Female	MDR-TB	Deaths	0.0	0.0	0.0
90 to 94	92.5	China	Female	MDR-TB	Deaths	0.0	0.0	0.0
95 plus	97.5	China	Female	MDR-TB	Deaths	0.0	0.0	0.0
0 to 4	2.5	Low SDI	Female	MDR-TB	Deaths	0.8	0.7	0.9
5 to 9	7.5	Low SDI	Female	MDR-TB	Deaths	0.1	0.1	0.1
10 to 14	12.5	Low SDI	Female	MDR-TB	Deaths	0.1	0.1	0.1
15 to 19	17.5	Low SDI	Female	MDR-TB	Deaths	0.2	0.2	0.3
20 to 24	22.5	Low SDI	Female	MDR-TB	Deaths	0.5	0.4	0.5
25 to 29	27.5	Low SDI	Female	MDR-TB	Deaths	0.7	0.7	0.8
30 to 34	32.5	Low SDI	Female	MDR-TB	Deaths	1.1	1.0	1.2
35 to 39	37.5	Low SDI	Female	MDR-TB	Deaths	1.5	1.4	1.6
40 to 44	42.5	Low SDI	Female	MDR-TB	Deaths	2.2	2.1	2.3
45 to 49	47.5	Low SDI	Female	MDR-TB	Deaths	3.4	3.3	3.6
50 to 54	52.5	Low SDI	Female	MDR-TB	Deaths	6.6	6.3	6.9
55 to 59	57.5	Low SDI	Female	MDR-TB	Deaths	10.3	9.8	10.8
60 to 64	62.5	Low SDI	Female	MDR-TB	Deaths	15.1	14.3	16.0
65 to 69	67.5	Low SDI	Female	MDR-TB	Deaths	21.3	20.1	22.7
70 to 74	72.5	Low SDI	Female	MDR-TB	Deaths	40.3	37.7	43.0
75 to 79	77.5	Low SDI	Female	MDR-TB	Deaths	61.9	57.6	66.6
80 to 84	82.5	Low SDI	Female	MDR-TB	Deaths	103.7	95.6	112.4
85 to 89	87.5	Low SDI	Female	MDR-TB	Deaths	139.8	126.8	154.1
90 to 94	92.5	Low SDI	Female	MDR-TB	Deaths	183.7	160.6	210.2
95 plus	97.5	Low SDI	Female	MDR-TB	Deaths	430.3	357.0	518.6
0 to 4	2.5	Low-middle SDI	Female	MDR-TB	Deaths	0.5	0.4	0.5
5 to 9	7.5	Low-middle SDI	Female	MDR-TB	Deaths	0.1	0.1	0.1
10 to 14	12.5	Low-middle SDI	Female	MDR-TB	Deaths	0.1	0.1	0.1
15 to 19	17.5	Low-middle SDI	Female	MDR-TB	Deaths	0.2	0.2	0.2
20 to 24	22.5	Low-middle SDI	Female	MDR-TB	Deaths	0.4	0.3	0.4
25 to 29	27.5	Low-middle SDI	Female	MDR-TB	Deaths	0.6	0.5	0.6
30 to 34	32.5	Low-middle SDI	Female	MDR-TB	Deaths	0.8	0.8	0.9
35 to 39	37.5	Low-middle SDI	Female	MDR-TB	Deaths	1.2	1.1	1.3
40 to 44	42.5	Low-middle SDI	Female	MDR-TB	Deaths	1.8	1.7	1.9
45 to 49	47.5	Low-middle SDI	Female	MDR-TB	Deaths	2.5	2.3	2.6

50 to 54	52.5	Low-middle SDI	Female	MDR-TB	Deaths	4.6	4.3	4.8
55 to 59	57.5	Low-middle SDI	Female	MDR-TB	Deaths	7.2	6.8	7.6
60 to 64	62.5	Low-middle SDI	Female	MDR-TB	Deaths	10.8	10.1	11.5
65 to 69	67.5	Low-middle SDI	Female	MDR-TB	Deaths	16.9	15.8	18.2
70 to 74	72.5	Low-middle SDI	Female	MDR-TB	Deaths	28.3	26.3	30.5
75 to 79	77.5	Low-middle SDI	Female	MDR-TB	Deaths	42.6	39.2	46.2
80 to 84	82.5	Low-middle SDI	Female	MDR-TB	Deaths	66.6	60.8	72.9
85 to 89	87.5	Low-middle SDI	Female	MDR-TB	Deaths	75.4	67.6	84.2
90 to 94	92.5	Low-middle SDI	Female	MDR-TB	Deaths	75.5	64.2	88.9
95 plus	97.5	Low-middle SDI	Female	MDR-TB	Deaths	86.2	64.4	115.3
0 to 4	2.5	Middle SDI	Female	MDR-TB	Deaths	1.5	1.3	1.8
5 to 9	7.5	Middle SDI	Female	MDR-TB	Deaths	0.2	0.2	0.3
10 to 14	12.5	Middle SDI	Female	MDR-TB	Deaths	0.2	0.2	0.2
15 to 19	17.5	Middle SDI	Female	MDR-TB	Deaths	0.3	0.3	0.4
20 to 24	22.5	Middle SDI	Female	MDR-TB	Deaths	0.5	0.5	0.6
25 to 29	27.5	Middle SDI	Female	MDR-TB	Deaths	0.6	0.5	0.7
30 to 34	32.5	Middle SDI	Female	MDR-TB	Deaths	0.6	0.6	0.7
35 to 39	37.5	Middle SDI	Female	MDR-TB	Deaths	0.6	0.6	0.7
40 to 44	42.5	Middle SDI	Female	MDR-TB	Deaths	0.7	0.6	0.7
45 to 49	47.5	Middle SDI	Female	MDR-TB	Deaths	0.7	0.7	0.8
50 to 54	52.5	Middle SDI	Female	MDR-TB	Deaths	0.9	0.8	1.0
55 to 59	57.5	Middle SDI	Female	MDR-TB	Deaths	1.0	1.0	1.1
60 to 64	62.5	Middle SDI	Female	MDR-TB	Deaths	1.3	1.1	1.4
65 to 69	67.5	Middle SDI	Female	MDR-TB	Deaths	1.6	1.5	1.8
70 to 74	72.5	Middle SDI	Female	MDR-TB	Deaths	2.1	1.9	2.3
75 to 79	77.5	Middle SDI	Female	MDR-TB	Deaths	2.5	2.2	2.7
80 to 84	82.5	Middle SDI	Female	MDR-TB	Deaths	3.1	2.8	3.5
85 to 89	87.5	Middle SDI	Female	MDR-TB	Deaths	3.2	2.8	3.6
90 to 94	92.5	Middle SDI	Female	MDR-TB	Deaths	2.7	2.3	3.2
95 plus	97.5	Middle SDI	Female	MDR-TB	Deaths	3.5	2.7	4.5
0 to 4	2.5	Global	Both	MDR-TB	Deaths	0.5	0.4	0.5
5 to 9	7.5	Global	Both	MDR-TB	Deaths	0.1	0.1	0.1
10 to 14	12.5	Global	Both	MDR-TB	Deaths	0.1	0.1	0.1
15 to 19	17.5	Global	Both	MDR-TB	Deaths	0.2	0.1	0.2
20 to 24	22.5	Global	Both	MDR-TB	Deaths	0.3	0.3	0.3
25 to 29	27.5	Global	Both	MDR-TB	Deaths	0.5	0.5	0.6
30 to 34	32.5	Global	Both	MDR-TB	Deaths	0.8	0.7	0.8
35 to 39	37.5	Global	Both	MDR-TB	Deaths	1.0	1.0	1.1
40 to 44	42.5	Global	Both	MDR-TB	Deaths	1.4	1.4	1.5
45 to 49	47.5	Global	Both	MDR-TB	Deaths	2.1	2.0	2.2
50 to 54	52.5	Global	Both	MDR-TB	Deaths	2.9	2.8	3.0
55 to 59	57.5	Global	Both	MDR-TB	Deaths	3.9	3.7	4.1
60 to 64	62.5	Global	Both	MDR-TB	Deaths	5.0	4.8	5.3
65 to 69	67.5	Global	Both	MDR-TB	Deaths	6.7	6.3	7.0
70 to 74	72.5	Global	Both	MDR-TB	Deaths	9.1	8.6	9.6
75 to 79	77.5	Global	Both	MDR-TB	Deaths	10.8	10.2	11.5
80 to 84	82.5	Global	Both	MDR-TB	Deaths	13.6	12.7	14.5
85 to 89	87.5	Global	Both	MDR-TB	Deaths	13.9	12.8	15.1
90 to 94	92.5	Global	Both	MDR-TB	Deaths	12.4	11.0	14.0
95 plus	97.5	Global	Both	MDR-TB	Deaths	12.1	9.8	14.9
0 to 4	2.5	High SDI	Both	MDR-TB	Deaths	0.0	0.0	0.1

5 to 9	7.5	High SDI	Both	MDR-TB	Deaths	0.0	0.0	0.0
10 to 14	12.5	High SDI	Both	MDR-TB	Deaths	0.0	0.0	0.0
15 to 19	17.5	High SDI	Both	MDR-TB	Deaths	0.0	0.0	0.0
20 to 24	22.5	High SDI	Both	MDR-TB	Deaths	0.0	0.0	0.0
25 to 29	27.5	High SDI	Both	MDR-TB	Deaths	0.0	0.0	0.1
30 to 34	32.5	High SDI	Both	MDR-TB	Deaths	0.0	0.0	0.1
35 to 39	37.5	High SDI	Both	MDR-TB	Deaths	0.1	0.0	0.1
40 to 44	42.5	High SDI	Both	MDR-TB	Deaths	0.1	0.0	0.1
45 to 49	47.5	High SDI	Both	MDR-TB	Deaths	0.1	0.1	0.1
50 to 54	52.5	High SDI	Both	MDR-TB	Deaths	0.1	0.0	0.1
55 to 59	57.5	High SDI	Both	MDR-TB	Deaths	0.1	0.0	0.1
60 to 64	62.5	High SDI	Both	MDR-TB	Deaths	0.0	0.0	0.1
65 to 69	67.5	High SDI	Both	MDR-TB	Deaths	0.0	0.0	0.1
70 to 74	72.5	High SDI	Both	MDR-TB	Deaths	0.0	0.0	0.1
75 to 79	77.5	High SDI	Both	MDR-TB	Deaths	0.1	0.0	0.1
80 to 84	82.5	High SDI	Both	MDR-TB	Deaths	0.1	0.1	0.1
85 to 89	87.5	High SDI	Both	MDR-TB	Deaths	0.1	0.1	0.1
90 to 94	92.5	High SDI	Both	MDR-TB	Deaths	0.1	0.1	0.1
95 plus	97.5	High SDI	Both	MDR-TB	Deaths	0.1	0.0	0.1
0 to 4	2.5	High-middle SDI	Both	MDR-TB	Deaths	0.6	0.4	0.8
5 to 9	7.5	High-middle SDI	Both	MDR-TB	Deaths	0.1	0.0	0.1
10 to 14	12.5	High-middle SDI	Both	MDR-TB	Deaths	0.1	0.0	0.1
15 to 19	17.5	High-middle SDI	Both	MDR-TB	Deaths	0.1	0.1	0.2
20 to 24	22.5	High-middle SDI	Both	MDR-TB	Deaths	0.4	0.3	0.4
25 to 29	27.5	High-middle SDI	Both	MDR-TB	Deaths	0.6	0.5	0.7
30 to 34	32.5	High-middle SDI	Both	MDR-TB	Deaths	0.8	0.7	0.9
35 to 39	37.5	High-middle SDI	Both	MDR-TB	Deaths	0.9	0.8	0.9
40 to 44	42.5	High-middle SDI	Both	MDR-TB	Deaths	1.1	1.0	1.2
45 to 49	47.5	High-middle SDI	Both	MDR-TB	Deaths	1.8	1.7	2.0
50 to 54	52.5	High-middle SDI	Both	MDR-TB	Deaths	1.9	1.8	2.1
55 to 59	57.5	High-middle SDI	Both	MDR-TB	Deaths	1.9	1.8	2.1
60 to 64	62.5	High-middle SDI	Both	MDR-TB	Deaths	1.7	1.6	1.9
65 to 69	67.5	High-middle SDI	Both	MDR-TB	Deaths	1.6	1.4	1.8
70 to 74	72.5	High-middle SDI	Both	MDR-TB	Deaths	1.5	1.3	1.7
75 to 79	77.5	High-middle SDI	Both	MDR-TB	Deaths	1.4	1.2	1.6
80 to 84	82.5	High-middle SDI	Both	MDR-TB	Deaths	1.3	1.1	1.5
85 to 89	87.5	High-middle SDI	Both	MDR-TB	Deaths	1.1	0.9	1.4
90 to 94	92.5	High-middle SDI	Both	MDR-TB	Deaths	0.8	0.6	1.1
95 plus	97.5	High-middle SDI	Both	MDR-TB	Deaths	0.6	0.3	1.2
0 to 4	2.5	China	Both	MDR-TB	Deaths	54.1	46.1	63.6
5 to 9	7.5	China	Both	MDR-TB	Deaths	3.2	2.7	3.9
10 to 14	12.5	China	Both	MDR-TB	Deaths	1.6	1.4	1.9
15 to 19	17.5	China	Both	MDR-TB	Deaths	1.5	1.3	1.7
20 to 24	22.5	China	Both	MDR-TB	Deaths	1.5	1.4	1.7
25 to 29	27.5	China	Both	MDR-TB	Deaths	1.1	1.0	1.2
30 to 34	32.5	China	Both	MDR-TB	Deaths	1.0	0.9	1.1
35 to 39	37.5	China	Both	MDR-TB	Deaths	0.7	0.7	0.8
40 to 44	42.5	China	Both	MDR-TB	Deaths	0.6	0.5	0.6
45 to 49	47.5	China	Both	MDR-TB	Deaths	0.4	0.4	0.5
50 to 54	52.5	China	Both	MDR-TB	Deaths	0.3	0.3	0.4
55 to 59	57.5	China	Both	MDR-TB	Deaths	0.3	0.3	0.3

60 to 64	62.5	China	Both	MDR-TB	Deaths	0.2	0.2	0.3
65 to 69	67.5	China	Both	MDR-TB	Deaths	0.2	0.2	0.2
70 to 74	72.5	China	Both	MDR-TB	Deaths	0.2	0.2	0.2
75 to 79	77.5	China	Both	MDR-TB	Deaths	0.1	0.1	0.2
80 to 84	82.5	China	Both	MDR-TB	Deaths	0.1	0.1	0.1
85 to 89	87.5	China	Both	MDR-TB	Deaths	0.1	0.1	0.1
90 to 94	92.5	China	Both	MDR-TB	Deaths	0.0	0.0	0.1
95 plus	97.5	China	Both	MDR-TB	Deaths	0.0	0.0	0.0
0 to 4	2.5	Low SDI	Both	MDR-TB	Deaths	0.6	0.6	0.6
5 to 9	7.5	Low SDI	Both	MDR-TB	Deaths	0.1	0.1	0.1
10 to 14	12.5	Low SDI	Both	MDR-TB	Deaths	0.1	0.1	0.1
15 to 19	17.5	Low SDI	Both	MDR-TB	Deaths	0.2	0.2	0.2
20 to 24	22.5	Low SDI	Both	MDR-TB	Deaths	0.5	0.5	0.5
25 to 29	27.5	Low SDI	Both	MDR-TB	Deaths	0.9	0.9	1.0
30 to 34	32.5	Low SDI	Both	MDR-TB	Deaths	1.5	1.5	1.6
35 to 39	37.5	Low SDI	Both	MDR-TB	Deaths	2.4	2.3	2.4
40 to 44	42.5	Low SDI	Both	MDR-TB	Deaths	3.6	3.5	3.7
45 to 49	47.5	Low SDI	Both	MDR-TB	Deaths	5.7	5.5	5.8
50 to 54	52.5	Low SDI	Both	MDR-TB	Deaths	9.8	9.5	10.0
55 to 59	57.5	Low SDI	Both	MDR-TB	Deaths	14.9	14.5	15.4
60 to 64	62.5	Low SDI	Both	MDR-TB	Deaths	20.9	20.3	21.6
65 to 69	67.5	Low SDI	Both	MDR-TB	Deaths	30.5	29.4	31.6
70 to 74	72.5	Low SDI	Both	MDR-TB	Deaths	53.9	51.8	56.0
75 to 79	77.5	Low SDI	Both	MDR-TB	Deaths	80.0	76.6	83.6
80 to 84	82.5	Low SDI	Both	MDR-TB	Deaths	140.9	134.2	147.9
85 to 89	87.5	Low SDI	Both	MDR-TB	Deaths	205.3	193.5	217.8
90 to 94	92.5	Low SDI	Both	MDR-TB	Deaths	275.6	253.3	299.8
95 plus	97.5	Low SDI	Both	MDR-TB	Deaths	471.9	410.7	542.3
0 to 4	2.5	Low-middle SDI	Both	MDR-TB	Deaths	0.3	0.3	0.4
5 to 9	7.5	Low-middle SDI	Both	MDR-TB	Deaths	0.1	0.1	0.1
10 to 14	12.5	Low-middle SDI	Both	MDR-TB	Deaths	0.1	0.1	0.1
15 to 19	17.5	Low-middle SDI	Both	MDR-TB	Deaths	0.2	0.2	0.2
20 to 24	22.5	Low-middle SDI	Both	MDR-TB	Deaths	0.3	0.3	0.4
25 to 29	27.5	Low-middle SDI	Both	MDR-TB	Deaths	0.6	0.6	0.6
30 to 34	32.5	Low-middle SDI	Both	MDR-TB	Deaths	1.0	1.0	1.0
35 to 39	37.5	Low-middle SDI	Both	MDR-TB	Deaths	1.6	1.6	1.7
40 to 44	42.5	Low-middle SDI	Both	MDR-TB	Deaths	2.6	2.5	2.7
45 to 49	47.5	Low-middle SDI	Both	MDR-TB	Deaths	4.2	4.1	4.4
50 to 54	52.5	Low-middle SDI	Both	MDR-TB	Deaths	7.0	6.8	7.2
55 to 59	57.5	Low-middle SDI	Both	MDR-TB	Deaths	11.4	11.0	11.7
60 to 64	62.5	Low-middle SDI	Both	MDR-TB	Deaths	17.3	16.6	18.0
65 to 69	67.5	Low-middle SDI	Both	MDR-TB	Deaths	27.1	26.0	28.3
70 to 74	72.5	Low-middle SDI	Both	MDR-TB	Deaths	42.3	40.4	44.3
75 to 79	77.5	Low-middle SDI	Both	MDR-TB	Deaths	61.5	58.5	64.7
80 to 84	82.5	Low-middle SDI	Both	MDR-TB	Deaths	95.7	90.4	101.2
85 to 89	87.5	Low-middle SDI	Both	MDR-TB	Deaths	118.9	110.9	127.4
90 to 94	92.5	Low-middle SDI	Both	MDR-TB	Deaths	131.0	118.0	145.3
95 plus	97.5	Low-middle SDI	Both	MDR-TB	Deaths	140.6	115.1	171.8
0 to 4	2.5	Middle SDI	Both	MDR-TB	Deaths	1.2	1.0	1.3
5 to 9	7.5	Middle SDI	Both	MDR-TB	Deaths	0.2	0.1	0.2
10 to 14	12.5	Middle SDI	Both	MDR-TB	Deaths	0.1	0.1	0.2

15 to 19	17.5	Middle SDI	Both	MDR-TB	Deaths	0.2	0.2	0.3
20 to 24	22.5	Middle SDI	Both	MDR-TB	Deaths	0.5	0.4	0.5
25 to 29	27.5	Middle SDI	Both	MDR-TB	Deaths	0.6	0.6	0.7
30 to 34	32.5	Middle SDI	Both	MDR-TB	Deaths	0.8	0.7	0.9
35 to 39	37.5	Middle SDI	Both	MDR-TB	Deaths	0.9	0.9	1.0
40 to 44	42.5	Middle SDI	Both	MDR-TB	Deaths	1.1	1.0	1.1
45 to 49	47.5	Middle SDI	Both	MDR-TB	Deaths	1.3	1.2	1.4
50 to 54	52.5	Middle SDI	Both	MDR-TB	Deaths	1.6	1.5	1.7
55 to 59	57.5	Middle SDI	Both	MDR-TB	Deaths	2.0	1.8	2.1
60 to 64	62.5	Middle SDI	Both	MDR-TB	Deaths	2.4	2.2	2.5
65 to 69	67.5	Middle SDI	Both	MDR-TB	Deaths	2.9	2.8	3.1
70 to 74	72.5	Middle SDI	Both	MDR-TB	Deaths	3.6	3.4	3.9
75 to 79	77.5	Middle SDI	Both	MDR-TB	Deaths	4.1	3.8	4.5
80 to 84	82.5	Middle SDI	Both	MDR-TB	Deaths	4.9	4.5	5.3
85 to 89	87.5	Middle SDI	Both	MDR-TB	Deaths	5.2	4.7	5.7
90 to 94	92.5	Middle SDI	Both	MDR-TB	Deaths	4.8	4.2	5.5
95 plus	97.5	Middle SDI	Both	MDR-TB	Deaths	5.2	4.1	6.6

S.Figure 4B Period influence on the mortality risk from MDR-TB in HIV-negative Individuals, segmented by SDI

label	period	location	sex	cause	measure	Rate Ratio	95%CI (lower)	95%CI (upper)
1990 to 1994	1992	Global	Male	MDR-TB	Deaths	0.3	0.3	0.4
1995 to 1999	1997	Global	Male	MDR-TB	Deaths	0.8	0.8	0.8
2000 to 2004	2002	Global	Male	MDR-TB	Deaths	1.0	1.0	1.0
2005 to 2009	2007	Global	Male	MDR-TB	Deaths	0.9	0.9	0.9
2010 to 2014	2012	Global	Male	MDR-TB	Deaths	0.7	0.7	0.7
2015 to 2019	2017	Global	Male	MDR-TB	Deaths	0.6	0.6	0.6
1990 to 1994	1992	High SDI	Male	MDR-TB	Deaths	1.0	0.9	1.1
1995 to 1999	1997	High SDI	Male	MDR-TB	Deaths	1.3	1.1	1.4
2000 to 2004	2002	High SDI	Male	MDR-TB	Deaths	1.0	1.0	1.0
2005 to 2009	2007	High SDI	Male	MDR-TB	Deaths	0.6	0.5	0.7
2010 to 2014	2012	High SDI	Male	MDR-TB	Deaths	0.4	0.3	0.4
2015 to 2019	2017	High SDI	Male	MDR-TB	Deaths	0.3	0.2	0.4
1990 to 1994	1992	High-middle SDI	Male	MDR-TB	Deaths	0.4	0.4	0.5
1995 to 1999	1997	High-middle SDI	Male	MDR-TB	Deaths	0.9	0.8	0.9
2000 to 2004	2002	High-middle SDI	Male	MDR-TB	Deaths	1.0	1.0	1.0
2005 to 2009	2007	High-middle SDI	Male	MDR-TB	Deaths	0.8	0.7	0.8
2010 to 2014	2012	High-middle SDI	Male	MDR-TB	Deaths	0.4	0.4	0.5
2015 to 2019	2017	High-middle SDI	Male	MDR-TB	Deaths	0.3	0.3	0.3
1990 to 1994	1992	Low SDI	Male	MDR-TB	Deaths	0.2	0.2	0.2
1995 to 1999	1997	Low SDI	Male	MDR-TB	Deaths	0.8	0.8	0.8
2000 to 2004	2002	Low SDI	Male	MDR-TB	Deaths	1.0	1.0	1.0
2005 to 2009	2007	Low SDI	Male	MDR-TB	Deaths	0.9	0.9	0.9
2010 to 2014	2012	Low SDI	Male	MDR-TB	Deaths	0.8	0.8	0.8
2015 to 2019	2017	Low SDI	Male	MDR-TB	Deaths	0.8	0.7	0.8
1990 to 1994	1992	Low-middle SDI	Male	MDR-TB	Deaths	0.2	0.2	0.2
1995 to 1999	1997	Low-middle SDI	Male	MDR-TB	Deaths	0.7	0.7	0.7
2000 to 2004	2002	Low-middle SDI	Male	MDR-TB	Deaths	1.0	1.0	1.0
2005 to 2009	2007	Low-middle SDI	Male	MDR-TB	Deaths	0.9	0.9	1.0
2010 to 2014	2012	Low-middle SDI	Male	MDR-TB	Deaths	0.8	0.8	0.9
2015 to 2019	2017	Low-middle SDI	Male	MDR-TB	Deaths	0.8	0.7	0.8
1990 to 1994	1992	Middle SDI	Male	MDR-TB	Deaths	0.6	0.6	0.6
1995 to 1999	1997	Middle SDI	Male	MDR-TB	Deaths	0.9	0.9	1.0
2000 to 2004	2002	Middle SDI	Male	MDR-TB	Deaths	1.0	1.0	1.0
2005 to 2009	2007	Middle SDI	Male	MDR-TB	Deaths	0.8	0.8	0.8
2010 to 2014	2012	Middle SDI	Male	MDR-TB	Deaths	0.6	0.6	0.6
2015 to 2019	2017	Middle SDI	Male	MDR-TB	Deaths	0.4	0.4	0.5
1990 to 1994	1992	Global	Female	MDR-TB	Deaths	0.4	0.4	0.4
1995 to 1999	1997	Global	Female	MDR-TB	Deaths	0.8	0.8	0.9
2000 to 2004	2002	Global	Female	MDR-TB	Deaths	1.0	1.0	1.0
2005 to 2009	2007	Global	Female	MDR-TB	Deaths	0.9	0.8	0.9
2010 to 2014	2012	Global	Female	MDR-TB	Deaths	0.7	0.7	0.8
2015 to 2019	2017	Global	Female	MDR-TB	Deaths	0.7	0.6	0.7
1990 to 1994	1992	High SDI	Female	MDR-TB	Deaths	0.9	0.8	1.1
1995 to 1999	1997	High SDI	Female	MDR-TB	Deaths	1.1	1.0	1.3
2000 to 2004	2002	High SDI	Female	MDR-TB	Deaths	1.0	1.0	1.0
2005 to 2009	2007	High SDI	Female	MDR-TB	Deaths	0.6	0.5	0.7

2010 to 2014	2012	High SDI	Female	MDR-TB	Deaths	0.4	0.3	0.5
2015 to 2019	2017	High SDI	Female	MDR-TB	Deaths	0.4	0.3	0.5
1990 to 1994	1992	High-middle SDI	Female	MDR-TB	Deaths	0.5	0.5	0.6
1995 to 1999	1997	High-middle SDI	Female	MDR-TB	Deaths	0.9	0.8	1.0
2000 to 2004	2002	High-middle SDI	Female	MDR-TB	Deaths	1.0	1.0	1.0
2005 to 2009	2007	High-middle SDI	Female	MDR-TB	Deaths	0.8	0.8	0.9
2010 to 2014	2012	High-middle SDI	Female	MDR-TB	Deaths	0.5	0.5	0.6
2015 to 2019	2017	High-middle SDI	Female	MDR-TB	Deaths	0.4	0.4	0.5
1990 to 1994	1992	Low SDI	Female	MDR-TB	Deaths	0.2	0.2	0.3
1995 to 1999	1997	Low SDI	Female	MDR-TB	Deaths	0.8	0.8	0.8
2000 to 2004	2002	Low SDI	Female	MDR-TB	Deaths	1.0	1.0	1.0
2005 to 2009	2007	Low SDI	Female	MDR-TB	Deaths	0.9	0.9	0.9
2010 to 2014	2012	Low SDI	Female	MDR-TB	Deaths	0.8	0.8	0.8
2015 to 2019	2017	Low SDI	Female	MDR-TB	Deaths	0.7	0.7	0.7
1990 to 1994	1992	Low-middle SDI	Female	MDR-TB	Deaths	0.2	0.2	0.3
1995 to 1999	1997	Low-middle SDI	Female	MDR-TB	Deaths	0.7	0.7	0.8
2000 to 2004	2002	Low-middle SDI	Female	MDR-TB	Deaths	1.0	1.0	1.0
2005 to 2009	2007	Low-middle SDI	Female	MDR-TB	Deaths	0.9	0.9	0.9
2010 to 2014	2012	Low-middle SDI	Female	MDR-TB	Deaths	0.8	0.7	0.8
2015 to 2019	2017	Low-middle SDI	Female	MDR-TB	Deaths	0.7	0.7	0.7
1990 to 1994	1992	Middle SDI	Female	MDR-TB	Deaths	0.7	0.7	0.7
1995 to 1999	1997	Middle SDI	Female	MDR-TB	Deaths	1.0	1.0	1.1
2000 to 2004	2002	Middle SDI	Female	MDR-TB	Deaths	1.0	1.0	1.0
2005 to 2009	2007	Middle SDI	Female	MDR-TB	Deaths	0.8	0.8	0.8
2010 to 2014	2012	Middle SDI	Female	MDR-TB	Deaths	0.6	0.5	0.6
2015 to 2019	2017	Middle SDI	Female	MDR-TB	Deaths	0.4	0.4	0.4
1990 to 1994	1992	Global	Both	MDR-TB	Deaths	0.4	0.3	0.4
1995 to 1999	1997	Global	Both	MDR-TB	Deaths	0.8	0.8	0.8
2000 to 2004	2002	Global	Both	MDR-TB	Deaths	1.0	1.0	1.0
2005 to 2009	2007	Global	Both	MDR-TB	Deaths	0.9	0.9	0.9
2010 to 2014	2012	Global	Both	MDR-TB	Deaths	0.7	0.7	0.7
2015 to 2019	2017	Global	Both	MDR-TB	Deaths	0.6	0.6	0.7
1990 to 1994	1992	High SDI	Both	MDR-TB	Deaths	1.0	0.9	1.1
1995 to 1999	1997	High SDI	Both	MDR-TB	Deaths	1.2	1.1	1.3
2000 to 2004	2002	High SDI	Both	MDR-TB	Deaths	1.0	1.0	1.0
2005 to 2009	2007	High SDI	Both	MDR-TB	Deaths	0.6	0.6	0.7
2010 to 2014	2012	High SDI	Both	MDR-TB	Deaths	0.4	0.3	0.4
2015 to 2019	2017	High SDI	Both	MDR-TB	Deaths	0.3	0.3	0.4
1990 to 1994	1992	High-middle SDI	Both	MDR-TB	Deaths	0.4	0.4	0.5
1995 to 1999	1997	High-middle SDI	Both	MDR-TB	Deaths	0.9	0.8	0.9
2000 to 2004	2002	High-middle SDI	Both	MDR-TB	Deaths	1.0	1.0	1.0
2005 to 2009	2007	High-middle SDI	Both	MDR-TB	Deaths	0.8	0.7	0.8
2010 to 2014	2012	High-middle SDI	Both	MDR-TB	Deaths	0.5	0.4	0.5
2015 to 2019	2017	High-middle SDI	Both	MDR-TB	Deaths	0.3	0.3	0.3
1990 to 1994	1992	Low SDI	Both	MDR-TB	Deaths	0.2	0.2	0.2
1995 to 1999	1997	Low SDI	Both	MDR-TB	Deaths	0.8	0.8	0.8
2000 to 2004	2002	Low SDI	Both	MDR-TB	Deaths	1.0	1.0	1.0
2005 to 2009	2007	Low SDI	Both	MDR-TB	Deaths	0.9	0.9	0.9
2010 to 2014	2012	Low SDI	Both	MDR-TB	Deaths	0.8	0.8	0.8
2015 to 2019	2017	Low SDI	Both	MDR-TB	Deaths	0.7	0.7	0.8
1990 to 1994	1992	Low-middle SDI	Both	MDR-TB	Deaths	0.2	0.2	0.2

1995 to 1999	1997	Low-middle SDI	Both	MDR-TB	Deaths	0.7	0.7	0.8
2000 to 2004	2002	Low-middle SDI	Both	MDR-TB	Deaths	1.0	1.0	1.0
2005 to 2009	2007	Low-middle SDI	Both	MDR-TB	Deaths	0.9	0.9	0.9
2010 to 2014	2012	Low-middle SDI	Both	MDR-TB	Deaths	0.8	0.8	0.8
2015 to 2019	2017	Low-middle SDI	Both	MDR-TB	Deaths	0.7	0.7	0.8
1990 to 1994	1992	Middle SDI	Both	MDR-TB	Deaths	0.6	0.6	0.6
1995 to 1999	1997	Middle SDI	Both	MDR-TB	Deaths	1.0	0.9	1.0
2000 to 2004	2002	Middle SDI	Both	MDR-TB	Deaths	1.0	1.0	1.0
2005 to 2009	2007	Middle SDI	Both	MDR-TB	Deaths	0.8	0.8	0.8
2010 to 2014	2012	Middle SDI	Both	MDR-TB	Deaths	0.6	0.6	0.6
2015 to 2019	2017	Middle SDI	Both	MDR-TB	Deaths	0.4	0.4	0.4

S.Figure 4C Cohort effect on the mortality risk from MDR-TB among HIV-negative individuals, stratified by SDI

label	Cohort	location	sex	cause	measure	Rate Ratio	95%CI (lower)	95%CI (upper)
1890 to 1899	1895	Global	Male	MDR-TB	Deaths	0.7	0.2	2.2
1895 to 1904	1900	Global	Male	MDR-TB	Deaths	0.7	0.4	1.0
1900 to 1909	1905	Global	Male	MDR-TB	Deaths	0.7	0.5	0.8
1905 to 1914	1910	Global	Male	MDR-TB	Deaths	0.6	0.6	0.7
1910 to 1919	1915	Global	Male	MDR-TB	Deaths	0.7	0.6	0.8
1915 to 1924	1920	Global	Male	MDR-TB	Deaths	0.7	0.6	0.8
1920 to 1929	1925	Global	Male	MDR-TB	Deaths	0.7	0.6	0.7
1925 to 1934	1930	Global	Male	MDR-TB	Deaths	0.7	0.7	0.8
1930 to 1939	1935	Global	Male	MDR-TB	Deaths	0.7	0.7	0.8
1935 to 1944	1940	Global	Male	MDR-TB	Deaths	0.7	0.7	0.8
1940 to 1949	1945	Global	Male	MDR-TB	Deaths	0.8	0.7	0.8
1945 to 1954	1950	Global	Male	MDR-TB	Deaths	0.8	0.8	0.8
1950 to 1959	1955	Global	Male	MDR-TB	Deaths	0.9	0.9	0.9
1955 to 1964	1960	Global	Male	MDR-TB	Deaths	1.0	1.0	1.0
1960 to 1969	1965	Global	Male	MDR-TB	Deaths	1.1	1.0	1.1
1965 to 1974	1970	Global	Male	MDR-TB	Deaths	1.2	1.1	1.3
1970 to 1979	1975	Global	Male	MDR-TB	Deaths	1.5	1.4	1.6
1975 to 1984	1980	Global	Male	MDR-TB	Deaths	1.7	1.6	1.8
1980 to 1989	1985	Global	Male	MDR-TB	Deaths	1.8	1.7	2.0
1985 to 1994	1990	Global	Male	MDR-TB	Deaths	2.1	1.9	2.3
1990 to 1999	1995	Global	Male	MDR-TB	Deaths	2.2	2.0	2.5
1995 to 2004	2000	Global	Male	MDR-TB	Deaths	2.2	1.9	2.5
2000 to 2009	2005	Global	Male	MDR-TB	Deaths	2.2	1.9	2.5
2005 to 2014	2010	Global	Male	MDR-TB	Deaths	2.2	1.9	2.6
2010 to 2019	2015	Global	Male	MDR-TB	Deaths	2.2	1.8	2.6
1890 to 1899	1895	High SDI	Male	MDR-TB	Deaths	37.0	10.9	125.6
1895 to 1904	1900	High SDI	Male	MDR-TB	Deaths	28.9	16.3	51.3
1900 to 1909	1905	High SDI	Male	MDR-TB	Deaths	24.5	16.9	35.6
1905 to 1914	1910	High SDI	Male	MDR-TB	Deaths	22.2	16.4	30.0
1910 to 1919	1915	High SDI	Male	MDR-TB	Deaths	19.1	14.5	25.1
1915 to 1924	1920	High SDI	Male	MDR-TB	Deaths	14.7	11.3	19.1
1920 to 1929	1925	High SDI	Male	MDR-TB	Deaths	10.8	8.4	13.9
1925 to 1934	1930	High SDI	Male	MDR-TB	Deaths	7.6	6.0	9.8
1930 to 1939	1935	High SDI	Male	MDR-TB	Deaths	5.1	4.0	6.5
1935 to 1944	1940	High SDI	Male	MDR-TB	Deaths	3.4	2.7	4.3
1940 to 1949	1945	High SDI	Male	MDR-TB	Deaths	2.2	1.7	2.7
1945 to 1954	1950	High SDI	Male	MDR-TB	Deaths	1.5	1.3	1.9
1950 to 1959	1955	High SDI	Male	MDR-TB	Deaths	1.3	1.0	1.6
1955 to 1964	1960	High SDI	Male	MDR-TB	Deaths	1.0	1.0	1.0
1960 to 1969	1965	High SDI	Male	MDR-TB	Deaths	0.7	0.6	0.9
1965 to 1974	1970	High SDI	Male	MDR-TB	Deaths	0.6	0.4	0.7
1970 to 1979	1975	High SDI	Male	MDR-TB	Deaths	0.5	0.3	0.7
1975 to 1984	1980	High SDI	Male	MDR-TB	Deaths	0.4	0.3	0.6
1980 to 1989	1985	High SDI	Male	MDR-TB	Deaths	0.3	0.2	0.6
1985 to 1994	1990	High SDI	Male	MDR-TB	Deaths	0.3	0.2	0.6
1990 to 1999	1995	High SDI	Male	MDR-TB	Deaths	0.2	0.1	0.6
1995 to 2004	2000	High SDI	Male	MDR-TB	Deaths	0.2	0.1	0.6
2000 to 2009	2005	High SDI	Male	MDR-TB	Deaths	0.1	0.0	0.5
2005 to 2014	2010	High SDI	Male	MDR-TB	Deaths	0.0	0.0	1.6
2010 to 2019	2015	High SDI	Male	MDR-TB	Deaths	0.0	0.0	5.3
1890 to 1899	1895	High-middle SDI	Male	MDR-TB	Deaths	11.2	0.8	153.7
1895 to 1904	1900	High-middle SDI	Male	MDR-TB	Deaths	8.9	3.7	21.9
1900 to 1909	1905	High-middle SDI	Male	MDR-TB	Deaths	6.7	4.3	10.2
1905 to 1914	1910	High-middle SDI	Male	MDR-TB	Deaths	5.0	3.8	6.5
1910 to 1919	1915	High-middle SDI	Male	MDR-TB	Deaths	4.2	3.5	5.1
1915 to 1924	1920	High-middle SDI	Male	MDR-TB	Deaths	3.2	2.7	3.8
1920 to 1929	1925	High-middle SDI	Male	MDR-TB	Deaths	2.4	2.0	2.7

1925 to 1934	1930	High-middle SDI	Male	MDR-TB	Deaths	1.8	1.6	2.0
1930 to 1939	1935	High-middle SDI	Male	MDR-TB	Deaths	1.4	1.3	1.6
1935 to 1944	1940	High-middle SDI	Male	MDR-TB	Deaths	1.2	1.1	1.4
1940 to 1949	1945	High-middle SDI	Male	MDR-TB	Deaths	0.9	0.8	1.0
1945 to 1954	1950	High-middle SDI	Male	MDR-TB	Deaths	1.0	0.9	1.1
1950 to 1959	1955	High-middle SDI	Male	MDR-TB	Deaths	1.0	0.9	1.1
1955 to 1964	1960	High-middle SDI	Male	MDR-TB	Deaths	1.0	1.0	1.0
1960 to 1969	1965	High-middle SDI	Male	MDR-TB	Deaths	0.8	0.8	0.9
1965 to 1974	1970	High-middle SDI	Male	MDR-TB	Deaths	0.8	0.7	0.9
1970 to 1979	1975	High-middle SDI	Male	MDR-TB	Deaths	0.9	0.8	1.0
1975 to 1984	1980	High-middle SDI	Male	MDR-TB	Deaths	0.9	0.8	1.1
1980 to 1989	1985	High-middle SDI	Male	MDR-TB	Deaths	0.8	0.7	1.0
1985 to 1994	1990	High-middle SDI	Male	MDR-TB	Deaths	0.7	0.6	0.9
1990 to 1999	1995	High-middle SDI	Male	MDR-TB	Deaths	0.6	0.4	0.8
1995 to 2004	2000	High-middle SDI	Male	MDR-TB	Deaths	0.4	0.2	0.5
2000 to 2009	2005	High-middle SDI	Male	MDR-TB	Deaths	0.3	0.2	0.4
2005 to 2014	2010	High-middle SDI	Male	MDR-TB	Deaths	0.2	0.1	0.4
2010 to 2019	2015	High-middle SDI	Male	MDR-TB	Deaths	0.2	0.1	0.4
1890 to 1899	1895	Low SDI	Male	MDR-TB	Deaths	0.1	0.0	0.3
1895 to 1904	1900	Low SDI	Male	MDR-TB	Deaths	0.1	0.1	0.2
1900 to 1909	1905	Low SDI	Male	MDR-TB	Deaths	0.1	0.1	0.2
1905 to 1914	1910	Low SDI	Male	MDR-TB	Deaths	0.2	0.1	0.2
1910 to 1919	1915	Low SDI	Male	MDR-TB	Deaths	0.2	0.2	0.2
1915 to 1924	1920	Low SDI	Male	MDR-TB	Deaths	0.2	0.2	0.3
1920 to 1929	1925	Low SDI	Male	MDR-TB	Deaths	0.3	0.3	0.3
1925 to 1934	1930	Low SDI	Male	MDR-TB	Deaths	0.4	0.3	0.4
1930 to 1939	1935	Low SDI	Male	MDR-TB	Deaths	0.4	0.4	0.5
1935 to 1944	1940	Low SDI	Male	MDR-TB	Deaths	0.5	0.5	0.5
1940 to 1949	1945	Low SDI	Male	MDR-TB	Deaths	0.6	0.6	0.6
1945 to 1954	1950	Low SDI	Male	MDR-TB	Deaths	0.7	0.7	0.7
1950 to 1959	1955	Low SDI	Male	MDR-TB	Deaths	0.8	0.8	0.9
1955 to 1964	1960	Low SDI	Male	MDR-TB	Deaths	1.0	1.0	1.0
1960 to 1969	1965	Low SDI	Male	MDR-TB	Deaths	1.2	1.1	1.2
1965 to 1974	1970	Low SDI	Male	MDR-TB	Deaths	1.4	1.3	1.5
1970 to 1979	1975	Low SDI	Male	MDR-TB	Deaths	1.7	1.6	1.8
1975 to 1984	1980	Low SDI	Male	MDR-TB	Deaths	2.1	2.0	2.2
1980 to 1989	1985	Low SDI	Male	MDR-TB	Deaths	2.5	2.3	2.6
1985 to 1994	1990	Low SDI	Male	MDR-TB	Deaths	3.0	2.8	3.2
1990 to 1999	1995	Low SDI	Male	MDR-TB	Deaths	3.3	3.1	3.6
1995 to 2004	2000	Low SDI	Male	MDR-TB	Deaths	3.4	3.2	3.7
2000 to 2009	2005	Low SDI	Male	MDR-TB	Deaths	3.8	3.4	4.1
2005 to 2014	2010	Low SDI	Male	MDR-TB	Deaths	4.1	3.8	4.6
2010 to 2019	2015	Low SDI	Male	MDR-TB	Deaths	4.4	4.0	4.9
1890 to 1899	1895	Low-middle SDI	Male	MDR-TB	Deaths	0.1	0.0	0.4
1895 to 1904	1900	Low-middle SDI	Male	MDR-TB	Deaths	0.1	0.1	0.2
1900 to 1909	1905	Low-middle SDI	Male	MDR-TB	Deaths	0.1	0.1	0.2
1905 to 1914	1910	Low-middle SDI	Male	MDR-TB	Deaths	0.2	0.2	0.2
1910 to 1919	1915	Low-middle SDI	Male	MDR-TB	Deaths	0.2	0.2	0.2
1915 to 1924	1920	Low-middle SDI	Male	MDR-TB	Deaths	0.2	0.2	0.3
1920 to 1929	1925	Low-middle SDI	Male	MDR-TB	Deaths	0.3	0.3	0.3
1925 to 1934	1930	Low-middle SDI	Male	MDR-TB	Deaths	0.3	0.3	0.4
1930 to 1939	1935	Low-middle SDI	Male	MDR-TB	Deaths	0.4	0.4	0.4
1935 to 1944	1940	Low-middle SDI	Male	MDR-TB	Deaths	0.4	0.4	0.5
1940 to 1949	1945	Low-middle SDI	Male	MDR-TB	Deaths	0.5	0.5	0.6
1945 to 1954	1950	Low-middle SDI	Male	MDR-TB	Deaths	0.6	0.6	0.7
1950 to 1959	1955	Low-middle SDI	Male	MDR-TB	Deaths	0.8	0.8	0.8
1955 to 1964	1960	Low-middle SDI	Male	MDR-TB	Deaths	1.0	1.0	1.0
1960 to 1969	1965	Low-middle SDI	Male	MDR-TB	Deaths	1.2	1.2	1.3
1965 to 1974	1970	Low-middle SDI	Male	MDR-TB	Deaths	1.6	1.5	1.6
1970 to 1979	1975	Low-middle SDI	Male	MDR-TB	Deaths	2.0	1.9	2.1
1975 to 1984	1980	Low-middle SDI	Male	MDR-TB	Deaths	2.4	2.3	2.6
1980 to 1989	1985	Low-middle SDI	Male	MDR-TB	Deaths	3.0	2.8	3.2
1985 to 1994	1990	Low-middle SDI	Male	MDR-TB	Deaths	3.6	3.4	3.9

1990 to 1999	1995	Low-middle SDI	Male	MDR-TB	Deaths	3.9	3.6	4.3
1995 to 2004	2000	Low-middle SDI	Male	MDR-TB	Deaths	4.0	3.6	4.4
2000 to 2009	2005	Low-middle SDI	Male	MDR-TB	Deaths	4.2	3.7	4.7
2005 to 2014	2010	Low-middle SDI	Male	MDR-TB	Deaths	4.3	3.8	4.8
2010 to 2019	2015	Low-middle SDI	Male	MDR-TB	Deaths	4.2	3.6	4.8
1890 to 1899	1895	Middle SDI	Male	MDR-TB	Deaths	3.6	1.3	10.1
1895 to 1904	1900	Middle SDI	Male	MDR-TB	Deaths	3.7	2.5	5.4
1900 to 1909	1905	Middle SDI	Male	MDR-TB	Deaths	3.4	2.8	4.1
1905 to 1914	1910	Middle SDI	Male	MDR-TB	Deaths	3.2	2.8	3.6
1910 to 1919	1915	Middle SDI	Male	MDR-TB	Deaths	2.9	2.6	3.2
1915 to 1924	1920	Middle SDI	Male	MDR-TB	Deaths	2.5	2.3	2.8
1920 to 1929	1925	Middle SDI	Male	MDR-TB	Deaths	2.2	2.0	2.3
1925 to 1934	1930	Middle SDI	Male	MDR-TB	Deaths	1.8	1.7	2.0
1930 to 1939	1935	Middle SDI	Male	MDR-TB	Deaths	1.6	1.5	1.7
1935 to 1944	1940	Middle SDI	Male	MDR-TB	Deaths	1.3	1.2	1.4
1940 to 1949	1945	Middle SDI	Male	MDR-TB	Deaths	1.2	1.1	1.2
1945 to 1954	1950	Middle SDI	Male	MDR-TB	Deaths	1.1	1.0	1.1
1950 to 1959	1955	Middle SDI	Male	MDR-TB	Deaths	1.0	0.9	1.1
1955 to 1964	1960	Middle SDI	Male	MDR-TB	Deaths	1.0	1.0	1.0
1960 to 1969	1965	Middle SDI	Male	MDR-TB	Deaths	0.9	0.9	1.0
1965 to 1974	1970	Middle SDI	Male	MDR-TB	Deaths	0.9	0.9	1.0
1970 to 1979	1975	Middle SDI	Male	MDR-TB	Deaths	0.9	0.9	1.0
1975 to 1984	1980	Middle SDI	Male	MDR-TB	Deaths	0.9	0.9	1.0
1980 to 1989	1985	Middle SDI	Male	MDR-TB	Deaths	0.9	0.8	1.0
1985 to 1994	1990	Middle SDI	Male	MDR-TB	Deaths	0.9	0.8	1.0
1990 to 1999	1995	Middle SDI	Male	MDR-TB	Deaths	0.8	0.7	0.9
1995 to 2004	2000	Middle SDI	Male	MDR-TB	Deaths	0.6	0.5	0.7
2000 to 2009	2005	Middle SDI	Male	MDR-TB	Deaths	0.4	0.4	0.5
2005 to 2014	2010	Middle SDI	Male	MDR-TB	Deaths	0.4	0.3	0.4
2010 to 2019	2015	Middle SDI	Male	MDR-TB	Deaths	0.3	0.2	0.4
1890 to 1899	1895	Global	Female	MDR-TB	Deaths	0.6	0.3	1.3
1895 to 1904	1900	Global	Female	MDR-TB	Deaths	0.6	0.4	0.8
1900 to 1909	1905	Global	Female	MDR-TB	Deaths	0.6	0.5	0.7
1905 to 1914	1910	Global	Female	MDR-TB	Deaths	0.6	0.5	0.6
1910 to 1919	1915	Global	Female	MDR-TB	Deaths	0.6	0.5	0.7
1915 to 1924	1920	Global	Female	MDR-TB	Deaths	0.6	0.6	0.7
1920 to 1929	1925	Global	Female	MDR-TB	Deaths	0.6	0.6	0.7
1925 to 1934	1930	Global	Female	MDR-TB	Deaths	0.7	0.6	0.7
1930 to 1939	1935	Global	Female	MDR-TB	Deaths	0.7	0.7	0.8
1935 to 1944	1940	Global	Female	MDR-TB	Deaths	0.7	0.7	0.8
1940 to 1949	1945	Global	Female	MDR-TB	Deaths	0.8	0.8	0.8
1945 to 1954	1950	Global	Female	MDR-TB	Deaths	0.8	0.8	0.9
1950 to 1959	1955	Global	Female	MDR-TB	Deaths	0.9	0.8	0.9
1955 to 1964	1960	Global	Female	MDR-TB	Deaths	1.0	1.0	1.0
1960 to 1969	1965	Global	Female	MDR-TB	Deaths	1.1	1.0	1.2
1965 to 1974	1970	Global	Female	MDR-TB	Deaths	1.2	1.1	1.3
1970 to 1979	1975	Global	Female	MDR-TB	Deaths	1.4	1.3	1.5
1975 to 1984	1980	Global	Female	MDR-TB	Deaths	1.5	1.4	1.6
1980 to 1989	1985	Global	Female	MDR-TB	Deaths	1.5	1.4	1.7
1985 to 1994	1990	Global	Female	MDR-TB	Deaths	1.7	1.5	1.8
1990 to 1999	1995	Global	Female	MDR-TB	Deaths	1.8	1.6	2.0
1995 to 2004	2000	Global	Female	MDR-TB	Deaths	1.8	1.6	2.0
2000 to 2009	2005	Global	Female	MDR-TB	Deaths	1.9	1.7	2.1
2005 to 2014	2010	Global	Female	MDR-TB	Deaths	1.9	1.7	2.1
2010 to 2019	2015	Global	Female	MDR-TB	Deaths	1.7	1.5	1.9
1890 to 1899	1895	High SDI	Female	MDR-TB	Deaths	15.3	5.0	47.0
1895 to 1904	1900	High SDI	Female	MDR-TB	Deaths	13.6	7.1	25.9
1900 to 1909	1905	High SDI	Female	MDR-TB	Deaths	12.9	7.7	21.5
1905 to 1914	1910	High SDI	Female	MDR-TB	Deaths	12.1	7.6	19.4
1910 to 1919	1915	High SDI	Female	MDR-TB	Deaths	11.2	7.1	17.6
1915 to 1924	1920	High SDI	Female	MDR-TB	Deaths	9.3	6.0	14.5
1920 to 1929	1925	High SDI	Female	MDR-TB	Deaths	7.6	4.9	11.7
1925 to 1934	1930	High SDI	Female	MDR-TB	Deaths	5.9	3.8	9.1

1930 to 1939	1935	High SDI	Female	MDR-TB	Deaths	4.1	2.7	6.3
1935 to 1944	1940	High SDI	Female	MDR-TB	Deaths	2.7	1.8	4.2
1940 to 1949	1945	High SDI	Female	MDR-TB	Deaths	1.8	1.2	2.8
1945 to 1954	1950	High SDI	Female	MDR-TB	Deaths	1.4	1.0	2.1
1950 to 1959	1955	High SDI	Female	MDR-TB	Deaths	1.2	0.8	1.8
1955 to 1964	1960	High SDI	Female	MDR-TB	Deaths	1.0	1.0	1.0
1960 to 1969	1965	High SDI	Female	MDR-TB	Deaths	0.8	0.6	1.2
1965 to 1974	1970	High SDI	Female	MDR-TB	Deaths	0.7	0.5	1.1
1970 to 1979	1975	High SDI	Female	MDR-TB	Deaths	0.7	0.4	1.1
1975 to 1984	1980	High SDI	Female	MDR-TB	Deaths	0.6	0.3	1.1
1980 to 1989	1985	High SDI	Female	MDR-TB	Deaths	0.5	0.3	1.0
1985 to 1994	1990	High SDI	Female	MDR-TB	Deaths	0.4	0.2	0.9
1990 to 1999	1995	High SDI	Female	MDR-TB	Deaths	0.3	0.1	0.8
1995 to 2004	2000	High SDI	Female	MDR-TB	Deaths	0.2	0.1	0.8
2000 to 2009	2005	High SDI	Female	MDR-TB	Deaths	0.1	0.0	0.9
2005 to 2014	2010	High SDI	Female	MDR-TB	Deaths	0.0	0.0	2.5
2010 to 2019	2015	High SDI	Female	MDR-TB	Deaths	0.0	0.0	7.6
1890 to 1899	1895	High-middle SDI	Female	MDR-TB	Deaths	4.8	0.8	28.8
1895 to 1904	1900	High-middle SDI	Female	MDR-TB	Deaths	4.3	2.1	8.6
1900 to 1909	1905	High-middle SDI	Female	MDR-TB	Deaths	4.0	2.8	5.6
1905 to 1914	1910	High-middle SDI	Female	MDR-TB	Deaths	3.3	2.6	4.2
1910 to 1919	1915	High-middle SDI	Female	MDR-TB	Deaths	2.8	2.3	3.5
1915 to 1924	1920	High-middle SDI	Female	MDR-TB	Deaths	2.3	2.0	2.8
1920 to 1929	1925	High-middle SDI	Female	MDR-TB	Deaths	1.8	1.6	2.2
1925 to 1934	1930	High-middle SDI	Female	MDR-TB	Deaths	1.6	1.3	1.8
1930 to 1939	1935	High-middle SDI	Female	MDR-TB	Deaths	1.4	1.2	1.6
1935 to 1944	1940	High-middle SDI	Female	MDR-TB	Deaths	1.1	1.0	1.3
1940 to 1949	1945	High-middle SDI	Female	MDR-TB	Deaths	1.0	0.8	1.1
1945 to 1954	1950	High-middle SDI	Female	MDR-TB	Deaths	1.0	0.9	1.1
1950 to 1959	1955	High-middle SDI	Female	MDR-TB	Deaths	1.0	0.9	1.1
1955 to 1964	1960	High-middle SDI	Female	MDR-TB	Deaths	1.0	1.0	1.0
1960 to 1969	1965	High-middle SDI	Female	MDR-TB	Deaths	0.9	0.8	1.0
1965 to 1974	1970	High-middle SDI	Female	MDR-TB	Deaths	0.9	0.8	1.1
1970 to 1979	1975	High-middle SDI	Female	MDR-TB	Deaths	1.1	0.9	1.2
1975 to 1984	1980	High-middle SDI	Female	MDR-TB	Deaths	1.1	0.9	1.3
1980 to 1989	1985	High-middle SDI	Female	MDR-TB	Deaths	1.0	0.9	1.3
1985 to 1994	1990	High-middle SDI	Female	MDR-TB	Deaths	0.9	0.7	1.2
1990 to 1999	1995	High-middle SDI	Female	MDR-TB	Deaths	0.8	0.6	1.0
1995 to 2004	2000	High-middle SDI	Female	MDR-TB	Deaths	0.6	0.4	0.8
2000 to 2009	2005	High-middle SDI	Female	MDR-TB	Deaths	0.4	0.3	0.6
2005 to 2014	2010	High-middle SDI	Female	MDR-TB	Deaths	0.4	0.2	0.6
2010 to 2019	2015	High-middle SDI	Female	MDR-TB	Deaths	0.2	0.1	0.4
1890 to 1899	1895	Low SDI	Female	MDR-TB	Deaths	0.1	0.0	0.3
1895 to 1904	1900	Low SDI	Female	MDR-TB	Deaths	0.1	0.1	0.2
1900 to 1909	1905	Low SDI	Female	MDR-TB	Deaths	0.1	0.1	0.2
1905 to 1914	1910	Low SDI	Female	MDR-TB	Deaths	0.2	0.1	0.2
1910 to 1919	1915	Low SDI	Female	MDR-TB	Deaths	0.2	0.2	0.2
1915 to 1924	1920	Low SDI	Female	MDR-TB	Deaths	0.3	0.2	0.3
1920 to 1929	1925	Low SDI	Female	MDR-TB	Deaths	0.3	0.3	0.3
1925 to 1934	1930	Low SDI	Female	MDR-TB	Deaths	0.4	0.3	0.4
1930 to 1939	1935	Low SDI	Female	MDR-TB	Deaths	0.4	0.4	0.5
1935 to 1944	1940	Low SDI	Female	MDR-TB	Deaths	0.5	0.5	0.5
1940 to 1949	1945	Low SDI	Female	MDR-TB	Deaths	0.6	0.6	0.7
1945 to 1954	1950	Low SDI	Female	MDR-TB	Deaths	0.7	0.7	0.8
1950 to 1959	1955	Low SDI	Female	MDR-TB	Deaths	0.9	0.8	0.9
1955 to 1964	1960	Low SDI	Female	MDR-TB	Deaths	1.0	1.0	1.0
1960 to 1969	1965	Low SDI	Female	MDR-TB	Deaths	1.2	1.1	1.2
1965 to 1974	1970	Low SDI	Female	MDR-TB	Deaths	1.3	1.2	1.4
1970 to 1979	1975	Low SDI	Female	MDR-TB	Deaths	1.5	1.4	1.6
1975 to 1984	1980	Low SDI	Female	MDR-TB	Deaths	1.7	1.6	1.8
1980 to 1989	1985	Low SDI	Female	MDR-TB	Deaths	1.9	1.7	2.1
1985 to 1994	1990	Low SDI	Female	MDR-TB	Deaths	2.2	2.0	2.4
1990 to 1999	1995	Low SDI	Female	MDR-TB	Deaths	2.4	2.2	2.6

1995 to 2004	2000	Low SDI	Female	MDR-TB	Deaths	2.5	2.3	2.8
2000 to 2009	2005	Low SDI	Female	MDR-TB	Deaths	2.8	2.5	3.1
2005 to 2014	2010	Low SDI	Female	MDR-TB	Deaths	3.0	2.7	3.4
2010 to 2019	2015	Low SDI	Female	MDR-TB	Deaths	3.0	2.7	3.4
1890 to 1899	1895	Low-middle SDI	Female	MDR-TB	Deaths	0.1	0.0	0.8
1895 to 1904	1900	Low-middle SDI	Female	MDR-TB	Deaths	0.2	0.1	0.3
1900 to 1909	1905	Low-middle SDI	Female	MDR-TB	Deaths	0.2	0.1	0.3
1905 to 1914	1910	Low-middle SDI	Female	MDR-TB	Deaths	0.2	0.2	0.3
1910 to 1919	1915	Low-middle SDI	Female	MDR-TB	Deaths	0.3	0.2	0.3
1915 to 1924	1920	Low-middle SDI	Female	MDR-TB	Deaths	0.3	0.3	0.3
1920 to 1929	1925	Low-middle SDI	Female	MDR-TB	Deaths	0.3	0.3	0.4
1925 to 1934	1930	Low-middle SDI	Female	MDR-TB	Deaths	0.4	0.4	0.4
1930 to 1939	1935	Low-middle SDI	Female	MDR-TB	Deaths	0.4	0.4	0.5
1935 to 1944	1940	Low-middle SDI	Female	MDR-TB	Deaths	0.5	0.5	0.5
1940 to 1949	1945	Low-middle SDI	Female	MDR-TB	Deaths	0.6	0.6	0.6
1945 to 1954	1950	Low-middle SDI	Female	MDR-TB	Deaths	0.7	0.7	0.7
1950 to 1959	1955	Low-middle SDI	Female	MDR-TB	Deaths	0.8	0.8	0.9
1955 to 1964	1960	Low-middle SDI	Female	MDR-TB	Deaths	1.0	1.0	1.0
1960 to 1969	1965	Low-middle SDI	Female	MDR-TB	Deaths	1.2	1.2	1.3
1965 to 1974	1970	Low-middle SDI	Female	MDR-TB	Deaths	1.4	1.3	1.5
1970 to 1979	1975	Low-middle SDI	Female	MDR-TB	Deaths	1.7	1.6	1.9
1975 to 1984	1980	Low-middle SDI	Female	MDR-TB	Deaths	2.0	1.8	2.1
1980 to 1989	1985	Low-middle SDI	Female	MDR-TB	Deaths	2.2	2.0	2.4
1985 to 1994	1990	Low-middle SDI	Female	MDR-TB	Deaths	2.6	2.3	2.9
1990 to 1999	1995	Low-middle SDI	Female	MDR-TB	Deaths	2.7	2.4	3.0
1995 to 2004	2000	Low-middle SDI	Female	MDR-TB	Deaths	2.8	2.5	3.2
2000 to 2009	2005	Low-middle SDI	Female	MDR-TB	Deaths	3.1	2.7	3.6
2005 to 2014	2010	Low-middle SDI	Female	MDR-TB	Deaths	3.2	2.8	3.7
2010 to 2019	2015	Low-middle SDI	Female	MDR-TB	Deaths	2.8	2.4	3.4
1890 to 1899	1895	Middle SDI	Female	MDR-TB	Deaths	5.4	2.4	12.4
1895 to 1904	1900	Middle SDI	Female	MDR-TB	Deaths	5.1	3.5	7.4
1900 to 1909	1905	Middle SDI	Female	MDR-TB	Deaths	4.5	3.7	5.6
1905 to 1914	1910	Middle SDI	Female	MDR-TB	Deaths	4.1	3.6	4.8
1910 to 1919	1915	Middle SDI	Female	MDR-TB	Deaths	3.6	3.2	4.1
1915 to 1924	1920	Middle SDI	Female	MDR-TB	Deaths	3.2	2.9	3.6
1920 to 1929	1925	Middle SDI	Female	MDR-TB	Deaths	2.8	2.5	3.1
1925 to 1934	1930	Middle SDI	Female	MDR-TB	Deaths	2.4	2.1	2.6
1930 to 1939	1935	Middle SDI	Female	MDR-TB	Deaths	2.0	1.9	2.3
1935 to 1944	1940	Middle SDI	Female	MDR-TB	Deaths	1.7	1.6	1.9
1940 to 1949	1945	Middle SDI	Female	MDR-TB	Deaths	1.4	1.3	1.6
1945 to 1954	1950	Middle SDI	Female	MDR-TB	Deaths	1.2	1.1	1.3
1950 to 1959	1955	Middle SDI	Female	MDR-TB	Deaths	1.1	1.0	1.2
1955 to 1964	1960	Middle SDI	Female	MDR-TB	Deaths	1.0	1.0	1.0
1960 to 1969	1965	Middle SDI	Female	MDR-TB	Deaths	0.9	0.8	1.0
1965 to 1974	1970	Middle SDI	Female	MDR-TB	Deaths	0.8	0.7	0.9
1970 to 1979	1975	Middle SDI	Female	MDR-TB	Deaths	0.8	0.7	0.9
1975 to 1984	1980	Middle SDI	Female	MDR-TB	Deaths	0.7	0.6	0.8
1980 to 1989	1985	Middle SDI	Female	MDR-TB	Deaths	0.6	0.5	0.7
1985 to 1994	1990	Middle SDI	Female	MDR-TB	Deaths	0.6	0.5	0.7
1990 to 1999	1995	Middle SDI	Female	MDR-TB	Deaths	0.5	0.4	0.6
1995 to 2004	2000	Middle SDI	Female	MDR-TB	Deaths	0.4	0.3	0.5
2000 to 2009	2005	Middle SDI	Female	MDR-TB	Deaths	0.3	0.2	0.4
2005 to 2014	2010	Middle SDI	Female	MDR-TB	Deaths	0.2	0.2	0.3
2010 to 2019	2015	Middle SDI	Female	MDR-TB	Deaths	0.2	0.1	0.2
1890 to 1899	1895	Global	Both	MDR-TB	Deaths	0.6	0.2	1.5
1895 to 1904	1900	Global	Both	MDR-TB	Deaths	0.6	0.4	0.9
1900 to 1909	1905	Global	Both	MDR-TB	Deaths	0.6	0.5	0.7
1905 to 1914	1910	Global	Both	MDR-TB	Deaths	0.6	0.5	0.7
1910 to 1919	1915	Global	Both	MDR-TB	Deaths	0.6	0.6	0.7
1915 to 1924	1920	Global	Both	MDR-TB	Deaths	0.7	0.6	0.7
1920 to 1929	1925	Global	Both	MDR-TB	Deaths	0.7	0.6	0.7
1925 to 1934	1930	Global	Both	MDR-TB	Deaths	0.7	0.6	0.7
1930 to 1939	1935	Global	Both	MDR-TB	Deaths	0.7	0.7	0.8

1935 to 1944	1940	Global	Both	MDR-TB	Deaths	0.7	0.7	0.8
1940 to 1949	1945	Global	Both	MDR-TB	Deaths	0.8	0.7	0.8
1945 to 1954	1950	Global	Both	MDR-TB	Deaths	0.8	0.8	0.8
1950 to 1959	1955	Global	Both	MDR-TB	Deaths	0.9	0.9	0.9
1955 to 1964	1960	Global	Both	MDR-TB	Deaths	1.0	1.0	1.0
1960 to 1969	1965	Global	Both	MDR-TB	Deaths	1.1	1.0	1.1
1965 to 1974	1970	Global	Both	MDR-TB	Deaths	1.2	1.1	1.3
1970 to 1979	1975	Global	Both	MDR-TB	Deaths	1.4	1.4	1.5
1975 to 1984	1980	Global	Both	MDR-TB	Deaths	1.6	1.5	1.7
1980 to 1989	1985	Global	Both	MDR-TB	Deaths	1.7	1.6	1.8
1985 to 1994	1990	Global	Both	MDR-TB	Deaths	1.9	1.7	2.1
1990 to 1999	1995	Global	Both	MDR-TB	Deaths	2.0	1.8	2.2
1995 to 2004	2000	Global	Both	MDR-TB	Deaths	2.0	1.8	2.2
2000 to 2009	2005	Global	Both	MDR-TB	Deaths	2.0	1.8	2.3
2005 to 2014	2010	Global	Both	MDR-TB	Deaths	2.1	1.8	2.4
2010 to 2019	2015	Global	Both	MDR-TB	Deaths	1.9	1.7	2.2
1890 to 1899	1895	High SDI	Both	MDR-TB	Deaths	23.9	10.6	53.7
1895 to 1904	1900	High SDI	Both	MDR-TB	Deaths	19.8	13.1	29.9
1900 to 1909	1905	High SDI	Both	MDR-TB	Deaths	17.9	13.4	23.9
1905 to 1914	1910	High SDI	Both	MDR-TB	Deaths	16.6	13.0	21.3
1910 to 1919	1915	High SDI	Both	MDR-TB	Deaths	14.9	11.8	18.8
1915 to 1924	1920	High SDI	Both	MDR-TB	Deaths	12.1	9.7	15.2
1920 to 1929	1925	High SDI	Both	MDR-TB	Deaths	9.4	7.6	11.7
1925 to 1934	1930	High SDI	Both	MDR-TB	Deaths	7.0	5.7	8.7
1930 to 1939	1935	High SDI	Both	MDR-TB	Deaths	4.8	3.9	5.9
1935 to 1944	1940	High SDI	Both	MDR-TB	Deaths	3.2	2.6	3.9
1940 to 1949	1945	High SDI	Both	MDR-TB	Deaths	2.1	1.7	2.5
1945 to 1954	1950	High SDI	Both	MDR-TB	Deaths	1.5	1.3	1.8
1950 to 1959	1955	High SDI	Both	MDR-TB	Deaths	1.3	1.1	1.5
1955 to 1964	1960	High SDI	Both	MDR-TB	Deaths	1.0	1.0	1.0
1960 to 1969	1965	High SDI	Both	MDR-TB	Deaths	0.7	0.6	0.9
1965 to 1974	1970	High SDI	Both	MDR-TB	Deaths	0.6	0.5	0.8
1970 to 1979	1975	High SDI	Both	MDR-TB	Deaths	0.5	0.4	0.7
1975 to 1984	1980	High SDI	Both	MDR-TB	Deaths	0.5	0.3	0.6
1980 to 1989	1985	High SDI	Both	MDR-TB	Deaths	0.4	0.3	0.6
1985 to 1994	1990	High SDI	Both	MDR-TB	Deaths	0.3	0.2	0.5
1990 to 1999	1995	High SDI	Both	MDR-TB	Deaths	0.2	0.1	0.4
1995 to 2004	2000	High SDI	Both	MDR-TB	Deaths	0.1	0.1	0.4
2000 to 2009	2005	High SDI	Both	MDR-TB	Deaths	0.1	0.0	0.3
2005 to 2014	2010	High SDI	Both	MDR-TB	Deaths	0.0	0.0	0.3
2010 to 2019	2015	High SDI	Both	MDR-TB	Deaths	0.0	0.0	0.4
1890 to 1899	1895	High-middle SDI	Both	MDR-TB	Deaths	7.7	0.7	82.9
1895 to 1904	1900	High-middle SDI	Both	MDR-TB	Deaths	6.5	2.8	15.4
1900 to 1909	1905	High-middle SDI	Both	MDR-TB	Deaths	5.4	3.6	8.1
1905 to 1914	1910	High-middle SDI	Both	MDR-TB	Deaths	4.1	3.2	5.4
1910 to 1919	1915	High-middle SDI	Both	MDR-TB	Deaths	3.5	2.9	4.3
1915 to 1924	1920	High-middle SDI	Both	MDR-TB	Deaths	2.8	2.4	3.3
1920 to 1929	1925	High-middle SDI	Both	MDR-TB	Deaths	2.1	1.8	2.5
1925 to 1934	1930	High-middle SDI	Both	MDR-TB	Deaths	1.7	1.5	2.0
1930 to 1939	1935	High-middle SDI	Both	MDR-TB	Deaths	1.4	1.2	1.6
1935 to 1944	1940	High-middle SDI	Both	MDR-TB	Deaths	1.2	1.1	1.4
1940 to 1949	1945	High-middle SDI	Both	MDR-TB	Deaths	0.9	0.8	1.0
1945 to 1954	1950	High-middle SDI	Both	MDR-TB	Deaths	1.0	0.9	1.1
1950 to 1959	1955	High-middle SDI	Both	MDR-TB	Deaths	1.0	0.9	1.1
1955 to 1964	1960	High-middle SDI	Both	MDR-TB	Deaths	1.0	1.0	1.0
1960 to 1969	1965	High-middle SDI	Both	MDR-TB	Deaths	0.8	0.8	0.9
1965 to 1974	1970	High-middle SDI	Both	MDR-TB	Deaths	0.8	0.7	0.9
1970 to 1979	1975	High-middle SDI	Both	MDR-TB	Deaths	0.9	0.8	1.1
1975 to 1984	1980	High-middle SDI	Both	MDR-TB	Deaths	1.0	0.8	1.1
1980 to 1989	1985	High-middle SDI	Both	MDR-TB	Deaths	0.9	0.7	1.0
1985 to 1994	1990	High-middle SDI	Both	MDR-TB	Deaths	0.8	0.6	1.0
1990 to 1999	1995	High-middle SDI	Both	MDR-TB	Deaths	0.6	0.4	0.8
1995 to 2004	2000	High-middle SDI	Both	MDR-TB	Deaths	0.4	0.3	0.6

2000 to 2009	2005	High-middle SDI	Both	MDR-TB	Deaths	0.3	0.2	0.5
2005 to 2014	2010	High-middle SDI	Both	MDR-TB	Deaths	0.3	0.2	0.5
2010 to 2019	2015	High-middle SDI	Both	MDR-TB	Deaths	0.2	0.1	0.4
1890 to 1899	1895	Low SDI	Both	MDR-TB	Deaths	0.1	0.0	0.2
1895 to 1904	1900	Low SDI	Both	MDR-TB	Deaths	0.1	0.1	0.2
1900 to 1909	1905	Low SDI	Both	MDR-TB	Deaths	0.1	0.1	0.2
1905 to 1914	1910	Low SDI	Both	MDR-TB	Deaths	0.2	0.2	0.2
1910 to 1919	1915	Low SDI	Both	MDR-TB	Deaths	0.2	0.2	0.2
1915 to 1924	1920	Low SDI	Both	MDR-TB	Deaths	0.2	0.2	0.3
1920 to 1929	1925	Low SDI	Both	MDR-TB	Deaths	0.3	0.3	0.3
1925 to 1934	1930	Low SDI	Both	MDR-TB	Deaths	0.4	0.3	0.4
1930 to 1939	1935	Low SDI	Both	MDR-TB	Deaths	0.4	0.4	0.5
1935 to 1944	1940	Low SDI	Both	MDR-TB	Deaths	0.5	0.5	0.5
1940 to 1949	1945	Low SDI	Both	MDR-TB	Deaths	0.6	0.6	0.6
1945 to 1954	1950	Low SDI	Both	MDR-TB	Deaths	0.7	0.7	0.7
1950 to 1959	1955	Low SDI	Both	MDR-TB	Deaths	0.9	0.8	0.9
1955 to 1964	1960	Low SDI	Both	MDR-TB	Deaths	1.0	1.0	1.0
1960 to 1969	1965	Low SDI	Both	MDR-TB	Deaths	1.2	1.1	1.2
1965 to 1974	1970	Low SDI	Both	MDR-TB	Deaths	1.4	1.3	1.4
1970 to 1979	1975	Low SDI	Both	MDR-TB	Deaths	1.7	1.6	1.7
1975 to 1984	1980	Low SDI	Both	MDR-TB	Deaths	1.9	1.9	2.0
1980 to 1989	1985	Low SDI	Both	MDR-TB	Deaths	2.2	2.1	2.4
1985 to 1994	1990	Low SDI	Both	MDR-TB	Deaths	2.7	2.5	2.8
1990 to 1999	1995	Low SDI	Both	MDR-TB	Deaths	2.9	2.8	3.1
1995 to 2004	2000	Low SDI	Both	MDR-TB	Deaths	3.1	2.9	3.3
2000 to 2009	2005	Low SDI	Both	MDR-TB	Deaths	3.4	3.2	3.6
2005 to 2014	2010	Low SDI	Both	MDR-TB	Deaths	3.7	3.4	4.0
2010 to 2019	2015	Low SDI	Both	MDR-TB	Deaths	3.8	3.5	4.1
1890 to 1899	1895	Low-middle SDI	Both	MDR-TB	Deaths	0.1	0.0	0.4
1895 to 1904	1900	Low-middle SDI	Both	MDR-TB	Deaths	0.1	0.1	0.2
1900 to 1909	1905	Low-middle SDI	Both	MDR-TB	Deaths	0.2	0.1	0.2
1905 to 1914	1910	Low-middle SDI	Both	MDR-TB	Deaths	0.2	0.2	0.2
1910 to 1919	1915	Low-middle SDI	Both	MDR-TB	Deaths	0.2	0.2	0.2
1915 to 1924	1920	Low-middle SDI	Both	MDR-TB	Deaths	0.3	0.3	0.3
1920 to 1929	1925	Low-middle SDI	Both	MDR-TB	Deaths	0.3	0.3	0.3
1925 to 1934	1930	Low-middle SDI	Both	MDR-TB	Deaths	0.4	0.3	0.4
1930 to 1939	1935	Low-middle SDI	Both	MDR-TB	Deaths	0.4	0.4	0.4
1935 to 1944	1940	Low-middle SDI	Both	MDR-TB	Deaths	0.5	0.4	0.5
1940 to 1949	1945	Low-middle SDI	Both	MDR-TB	Deaths	0.6	0.5	0.6
1945 to 1954	1950	Low-middle SDI	Both	MDR-TB	Deaths	0.7	0.6	0.7
1950 to 1959	1955	Low-middle SDI	Both	MDR-TB	Deaths	0.8	0.8	0.8
1955 to 1964	1960	Low-middle SDI	Both	MDR-TB	Deaths	1.0	1.0	1.0
1960 to 1969	1965	Low-middle SDI	Both	MDR-TB	Deaths	1.2	1.2	1.3
1965 to 1974	1970	Low-middle SDI	Both	MDR-TB	Deaths	1.5	1.4	1.6
1970 to 1979	1975	Low-middle SDI	Both	MDR-TB	Deaths	1.9	1.8	2.0
1975 to 1984	1980	Low-middle SDI	Both	MDR-TB	Deaths	2.2	2.1	2.4
1980 to 1989	1985	Low-middle SDI	Both	MDR-TB	Deaths	2.6	2.5	2.8
1985 to 1994	1990	Low-middle SDI	Both	MDR-TB	Deaths	3.1	2.9	3.4
1990 to 1999	1995	Low-middle SDI	Both	MDR-TB	Deaths	3.3	3.1	3.6
1995 to 2004	2000	Low-middle SDI	Both	MDR-TB	Deaths	3.4	3.1	3.7
2000 to 2009	2005	Low-middle SDI	Both	MDR-TB	Deaths	3.7	3.4	4.1
2005 to 2014	2010	Low-middle SDI	Both	MDR-TB	Deaths	3.8	3.4	4.2
2010 to 2019	2015	Low-middle SDI	Both	MDR-TB	Deaths	3.6	3.2	4.0
1890 to 1899	1895	Middle SDI	Both	MDR-TB	Deaths	4.2	1.9	9.6
1895 to 1904	1900	Middle SDI	Both	MDR-TB	Deaths	4.2	3.0	5.8
1900 to 1909	1905	Middle SDI	Both	MDR-TB	Deaths	3.8	3.1	4.5
1905 to 1914	1910	Middle SDI	Both	MDR-TB	Deaths	3.5	3.1	3.9
1910 to 1919	1915	Middle SDI	Both	MDR-TB	Deaths	3.1	2.8	3.4
1915 to 1924	1920	Middle SDI	Both	MDR-TB	Deaths	2.7	2.5	3.0
1920 to 1929	1925	Middle SDI	Both	MDR-TB	Deaths	2.3	2.2	2.5
1925 to 1934	1930	Middle SDI	Both	MDR-TB	Deaths	2.0	1.9	2.2
1930 to 1939	1935	Middle SDI	Both	MDR-TB	Deaths	1.7	1.6	1.8
1935 to 1944	1940	Middle SDI	Both	MDR-TB	Deaths	1.5	1.4	1.6

1940 to 1949	1945	Middle SDI	Both	MDR-TB	Deaths	1.3	1.2	1.3
1945 to 1954	1950	Middle SDI	Both	MDR-TB	Deaths	1.1	1.1	1.2
1950 to 1959	1955	Middle SDI	Both	MDR-TB	Deaths	1.0	1.0	1.1
1955 to 1964	1960	Middle SDI	Both	MDR-TB	Deaths	1.0	1.0	1.0
1960 to 1969	1965	Middle SDI	Both	MDR-TB	Deaths	0.9	0.9	1.0
1965 to 1974	1970	Middle SDI	Both	MDR-TB	Deaths	0.9	0.8	0.9
1970 to 1979	1975	Middle SDI	Both	MDR-TB	Deaths	0.9	0.8	1.0
1975 to 1984	1980	Middle SDI	Both	MDR-TB	Deaths	0.8	0.8	0.9
1980 to 1989	1985	Middle SDI	Both	MDR-TB	Deaths	0.8	0.7	0.9
1985 to 1994	1990	Middle SDI	Both	MDR-TB	Deaths	0.8	0.7	0.9
1990 to 1999	1995	Middle SDI	Both	MDR-TB	Deaths	0.6	0.6	0.7
1995 to 2004	2000	Middle SDI	Both	MDR-TB	Deaths	0.5	0.4	0.6
2000 to 2009	2005	Middle SDI	Both	MDR-TB	Deaths	0.4	0.3	0.4
2005 to 2014	2010	Middle SDI	Both	MDR-TB	Deaths	0.3	0.2	0.4
2010 to 2019	2015	Middle SDI	Both	MDR-TB	Deaths	0.2	0.2	0.3

Figure S5 Projected mortality outcomes from MDR-TB: 2020-2030 forecast based on the Nordpred model, segmented by sex and SDI

measure	location	cause	sex	year	ASR	crude rate	case
Deaths	Global	MDR-TB	Male	1990	0.5	0.4	10430
Deaths	Global	MDR-TB	Male	1991	0.8	0.6	16151
Deaths	Global	MDR-TB	Male	1992	1.1	0.8	23123
Deaths	Global	MDR-TB	Male	1993	1.4	1.1	30790
Deaths	Global	MDR-TB	Male	1994	1.8	1.4	39597
Deaths	Global	MDR-TB	Male	1995	2.2	1.7	48219
Deaths	Global	MDR-TB	Male	1996	2.4	1.9	55542
Deaths	Global	MDR-TB	Male	1997	2.6	2.1	61291
Deaths	Global	MDR-TB	Male	1998	2.8	2.2	67036
Deaths	Global	MDR-TB	Male	1999	3.0	2.4	73455
Deaths	Global	MDR-TB	Male	2000	3.1	2.5	78641
Deaths	Global	MDR-TB	Male	2001	3.2	2.6	81764
Deaths	Global	MDR-TB	Male	2002	3.2	2.6	84198
Deaths	Global	MDR-TB	Male	2003	3.2	2.7	85469
Deaths	Global	MDR-TB	Male	2004	3.0	2.6	84250
Deaths	Global	MDR-TB	Male	2005	3.0	2.6	84707
Deaths	Global	MDR-TB	Male	2006	2.9	2.5	82893
Deaths	Global	MDR-TB	Male	2007	2.7	2.4	81547
Deaths	Global	MDR-TB	Male	2008	2.6	2.3	80537
Deaths	Global	MDR-TB	Male	2009	2.5	2.3	78162
Deaths	Global	MDR-TB	Male	2010	2.4	2.2	76033
Deaths	Global	MDR-TB	Male	2011	2.3	2.1	74480
Deaths	Global	MDR-TB	Male	2012	2.2	2.1	74940
Deaths	Global	MDR-TB	Male	2013	2.2	2.1	75061
Deaths	Global	MDR-TB	Male	2014	2.1	2.0	74139
Deaths	Global	MDR-TB	Male	2015	2.0	2.0	72600
Deaths	Global	MDR-TB	Male	2016	2.0	1.9	72652
Deaths	Global	MDR-TB	Male	2017	2.0	1.9	72834
Deaths	Global	MDR-TB	Male	2018	1.9	1.9	72208
Deaths	Global	MDR-TB	Male	2019	1.8	1.8	71216
Deaths	Global	MDR-TB	Male	2020	1.8	1.8	71354
Deaths	Global	MDR-TB	Male	2021	1.7	1.8	70725
Deaths	Global	MDR-TB	Male	2022	1.7	1.7	69978
Deaths	Global	MDR-TB	Male	2023	1.6	1.7	70162
Deaths	Global	MDR-TB	Male	2024	1.6	1.7	70323
Deaths	Global	MDR-TB	Male	2025	1.6	1.7	70440
Deaths	Global	MDR-TB	Male	2026	1.6	1.7	70498
Deaths	Global	MDR-TB	Male	2027	1.5	1.7	70491
Deaths	Global	MDR-TB	Male	2028	1.5	1.7	70441
Deaths	Global	MDR-TB	Male	2029	1.5	1.6	70361
Deaths	Global	MDR-TB	Male	2030	1.4	1.6	70235
Deaths	Global	MDR-TB	Female	1990	0.3	0.2	6326
Deaths	Global	MDR-TB	Female	1991	0.4	0.4	9703
Deaths	Global	MDR-TB	Female	1992	0.6	0.5	13707
Deaths	Global	MDR-TB	Female	1993	0.7	0.6	17739
Deaths	Global	MDR-TB	Female	1994	0.9	0.8	22361
Deaths	Global	MDR-TB	Female	1995	1.1	0.9	26914

Deaths	Global	MDR-TB	Female	1996	1.2	1.1	30801
Deaths	Global	MDR-TB	Female	1997	1.3	1.2	33793
Deaths	Global	MDR-TB	Female	1998	1.4	1.2	36881
Deaths	Global	MDR-TB	Female	1999	1.5	1.3	40191
Deaths	Global	MDR-TB	Female	2000	1.5	1.4	42426
Deaths	Global	MDR-TB	Female	2001	1.5	1.4	44149
Deaths	Global	MDR-TB	Female	2002	1.5	1.4	45087
Deaths	Global	MDR-TB	Female	2003	1.5	1.4	45211
Deaths	Global	MDR-TB	Female	2004	1.5	1.4	44340
Deaths	Global	MDR-TB	Female	2005	1.4	1.4	44399
Deaths	Global	MDR-TB	Female	2006	1.4	1.3	43751
Deaths	Global	MDR-TB	Female	2007	1.3	1.3	42698
Deaths	Global	MDR-TB	Female	2008	1.3	1.2	41951
Deaths	Global	MDR-TB	Female	2009	1.2	1.2	40638
Deaths	Global	MDR-TB	Female	2010	1.2	1.2	40039
Deaths	Global	MDR-TB	Female	2011	1.1	1.1	39807
Deaths	Global	MDR-TB	Female	2012	1.1	1.1	39548
Deaths	Global	MDR-TB	Female	2013	1.1	1.1	39765
Deaths	Global	MDR-TB	Female	2014	1.0	1.1	39283
Deaths	Global	MDR-TB	Female	2015	1.0	1.1	39672
Deaths	Global	MDR-TB	Female	2016	1.0	1.1	39722
Deaths	Global	MDR-TB	Female	2017	1.0	1.0	39510
Deaths	Global	MDR-TB	Female	2018	1.0	1.0	39302
Deaths	Global	MDR-TB	Female	2019	0.9	1.0	38768
Deaths	Global	MDR-TB	Female	2020	0.9	1.0	39597
Deaths	Global	MDR-TB	Female	2021	0.9	1.0	39553
Deaths	Global	MDR-TB	Female	2022	0.9	1.0	39473
Deaths	Global	MDR-TB	Female	2023	0.9	1.0	39739
Deaths	Global	MDR-TB	Female	2024	0.9	1.0	40004
Deaths	Global	MDR-TB	Female	2025	0.9	1.0	40258
Deaths	Global	MDR-TB	Female	2026	0.8	1.0	40494
Deaths	Global	MDR-TB	Female	2027	0.8	1.0	40707
Deaths	Global	MDR-TB	Female	2028	0.8	1.0	40910
Deaths	Global	MDR-TB	Female	2029	0.8	1.0	41109
Deaths	Global	MDR-TB	Female	2030	0.8	1.0	41296
Deaths	Global	MDR-TB	Both	1990	0.4	0.3	16755
Deaths	Global	MDR-TB	Both	1991	0.6	0.5	25854
Deaths	Global	MDR-TB	Both	1992	0.8	0.7	36830
Deaths	Global	MDR-TB	Both	1993	1.1	0.9	48528
Deaths	Global	MDR-TB	Both	1994	1.3	1.1	61958
Deaths	Global	MDR-TB	Both	1995	1.6	1.3	75132
Deaths	Global	MDR-TB	Both	1996	1.8	1.5	86342
Deaths	Global	MDR-TB	Both	1997	1.9	1.6	95084
Deaths	Global	MDR-TB	Both	1998	2.0	1.7	103917
Deaths	Global	MDR-TB	Both	1999	2.2	1.9	113645
Deaths	Global	MDR-TB	Both	2000	2.3	2.0	121067
Deaths	Global	MDR-TB	Both	2001	2.3	2.0	125913
Deaths	Global	MDR-TB	Both	2002	2.3	2.0	129286
Deaths	Global	MDR-TB	Both	2003	2.3	2.0	130680
Deaths	Global	MDR-TB	Both	2004	2.2	2.0	128590
Deaths	Global	MDR-TB	Both	2005	2.2	2.0	129106

Deaths	Global	MDR-TB	Both	2006	2.1	1.9	126643
Deaths	Global	MDR-TB	Both	2007	2.0	1.8	124245
Deaths	Global	MDR-TB	Both	2008	1.9	1.8	122487
Deaths	Global	MDR-TB	Both	2009	1.8	1.7	118799
Deaths	Global	MDR-TB	Both	2010	1.7	1.7	116072
Deaths	Global	MDR-TB	Both	2011	1.7	1.6	114287
Deaths	Global	MDR-TB	Both	2012	1.6	1.6	114487
Deaths	Global	MDR-TB	Both	2013	1.6	1.6	114826
Deaths	Global	MDR-TB	Both	2014	1.6	1.5	113421
Deaths	Global	MDR-TB	Both	2015	1.5	1.5	112272
Deaths	Global	MDR-TB	Both	2016	1.5	1.5	112374
Deaths	Global	MDR-TB	Both	2017	1.4	1.5	112344
Deaths	Global	MDR-TB	Both	2018	1.4	1.5	111510
Deaths	Global	MDR-TB	Both	2019	1.4	1.4	109984
Deaths	Global	MDR-TB	Both	2020	1.3	1.4	110952
Deaths	Global	MDR-TB	Both	2021	1.3	1.4	110278
Deaths	Global	MDR-TB	Both	2022	1.3	1.4	109452
Deaths	Global	MDR-TB	Both	2023	1.2	1.4	109901
Deaths	Global	MDR-TB	Both	2024	1.2	1.3	110327
Deaths	Global	MDR-TB	Both	2025	1.2	1.3	110697
Deaths	Global	MDR-TB	Both	2026	1.2	1.3	110993
Deaths	Global	MDR-TB	Both	2027	1.2	1.3	111198
Deaths	Global	MDR-TB	Both	2028	1.1	1.3	111351
Deaths	Global	MDR-TB	Both	2029	1.1	1.3	111470
Deaths	Global	MDR-TB	Both	2030	1.1	1.3	111531
Deaths	High SDI	MDR-TB	Male	1990	0.1	0.1	414
Deaths	High SDI	MDR-TB	Male	1991	0.1	0.1	539
Deaths	High SDI	MDR-TB	Male	1992	0.1	0.2	665
Deaths	High SDI	MDR-TB	Male	1993	0.2	0.2	784
Deaths	High SDI	MDR-TB	Male	1994	0.2	0.2	885
Deaths	High SDI	MDR-TB	Male	1995	0.2	0.2	937
Deaths	High SDI	MDR-TB	Male	1996	0.2	0.2	949
Deaths	High SDI	MDR-TB	Male	1997	0.2	0.2	932
Deaths	High SDI	MDR-TB	Male	1998	0.2	0.2	928
Deaths	High SDI	MDR-TB	Male	1999	0.2	0.2	918
Deaths	High SDI	MDR-TB	Male	2000	0.2	0.2	883
Deaths	High SDI	MDR-TB	Male	2001	0.2	0.2	857
Deaths	High SDI	MDR-TB	Male	2002	0.2	0.2	824
Deaths	High SDI	MDR-TB	Male	2003	0.1	0.2	787
Deaths	High SDI	MDR-TB	Male	2004	0.1	0.2	737
Deaths	High SDI	MDR-TB	Male	2005	0.1	0.1	672
Deaths	High SDI	MDR-TB	Male	2006	0.1	0.1	605
Deaths	High SDI	MDR-TB	Male	2007	0.1	0.1	554
Deaths	High SDI	MDR-TB	Male	2008	0.1	0.1	481
Deaths	High SDI	MDR-TB	Male	2009	0.1	0.1	419
Deaths	High SDI	MDR-TB	Male	2010	0.1	0.1	387
Deaths	High SDI	MDR-TB	Male	2011	0.1	0.1	362
Deaths	High SDI	MDR-TB	Male	2012	0.1	0.1	349
Deaths	High SDI	MDR-TB	Male	2013	0.0	0.1	341
Deaths	High SDI	MDR-TB	Male	2014	0.0	0.1	331
Deaths	High SDI	MDR-TB	Male	2015	0.0	0.1	321

Deaths	High SDI	MDR-TB	Male	2016	0.0	0.1	319
Deaths	High SDI	MDR-TB	Male	2017	0.0	0.1	316
Deaths	High SDI	MDR-TB	Male	2018	0.0	0.1	317
Deaths	High SDI	MDR-TB	Male	2019	0.0	0.1	320
Deaths	High SDI	MDR-TB	Male	2020	0.0	0.1	281
Deaths	High SDI	MDR-TB	Male	2021	0.0	0.1	272
Deaths	High SDI	MDR-TB	Male	2022	0.0	0.1	262
Deaths	High SDI	MDR-TB	Male	2023	0.0	0.1	259
Deaths	High SDI	MDR-TB	Male	2024	0.0	0.1	256
Deaths	High SDI	MDR-TB	Male	2025	0.0	0.1	252
Deaths	High SDI	MDR-TB	Male	2026	0.0	0.1	248
Deaths	High SDI	MDR-TB	Male	2027	0.0	0.1	244
Deaths	High SDI	MDR-TB	Male	2028	0.0	0.0	239
Deaths	High SDI	MDR-TB	Male	2029	0.0	0.0	233
Deaths	High SDI	MDR-TB	Male	2030	0.0	0.0	227
Deaths	High SDI	MDR-TB	Female	1990	0.0	0.0	201
Deaths	High SDI	MDR-TB	Female	1991	0.0	0.1	250
Deaths	High SDI	MDR-TB	Female	1992	0.0	0.1	295
Deaths	High SDI	MDR-TB	Female	1993	0.1	0.1	337
Deaths	High SDI	MDR-TB	Female	1994	0.1	0.1	363
Deaths	High SDI	MDR-TB	Female	1995	0.1	0.1	385
Deaths	High SDI	MDR-TB	Female	1996	0.1	0.1	398
Deaths	High SDI	MDR-TB	Female	1997	0.1	0.1	406
Deaths	High SDI	MDR-TB	Female	1998	0.1	0.1	416
Deaths	High SDI	MDR-TB	Female	1999	0.1	0.1	427
Deaths	High SDI	MDR-TB	Female	2000	0.1	0.1	416
Deaths	High SDI	MDR-TB	Female	2001	0.1	0.1	409
Deaths	High SDI	MDR-TB	Female	2002	0.1	0.1	400
Deaths	High SDI	MDR-TB	Female	2003	0.1	0.1	391
Deaths	High SDI	MDR-TB	Female	2004	0.0	0.1	366
Deaths	High SDI	MDR-TB	Female	2005	0.0	0.1	337
Deaths	High SDI	MDR-TB	Female	2006	0.0	0.1	305
Deaths	High SDI	MDR-TB	Female	2007	0.0	0.1	280
Deaths	High SDI	MDR-TB	Female	2008	0.0	0.1	252
Deaths	High SDI	MDR-TB	Female	2009	0.0	0.0	225
Deaths	High SDI	MDR-TB	Female	2010	0.0	0.0	210
Deaths	High SDI	MDR-TB	Female	2011	0.0	0.0	200
Deaths	High SDI	MDR-TB	Female	2012	0.0	0.0	194
Deaths	High SDI	MDR-TB	Female	2013	0.0	0.0	193
Deaths	High SDI	MDR-TB	Female	2014	0.0	0.0	190
Deaths	High SDI	MDR-TB	Female	2015	0.0	0.0	186
Deaths	High SDI	MDR-TB	Female	2016	0.0	0.0	187
Deaths	High SDI	MDR-TB	Female	2017	0.0	0.0	187
Deaths	High SDI	MDR-TB	Female	2018	0.0	0.0	189
Deaths	High SDI	MDR-TB	Female	2019	0.0	0.0	190
Deaths	High SDI	MDR-TB	Female	2020	0.0	0.0	171
Deaths	High SDI	MDR-TB	Female	2021	0.0	0.0	168
Deaths	High SDI	MDR-TB	Female	2022	0.0	0.0	164
Deaths	High SDI	MDR-TB	Female	2023	0.0	0.0	163
Deaths	High SDI	MDR-TB	Female	2024	0.0	0.0	161
Deaths	High SDI	MDR-TB	Female	2025	0.0	0.0	160

Deaths	High SDI	MDR-TB	Female	2026	0.0	0.0	159
Deaths	High SDI	MDR-TB	Female	2027	0.0	0.0	157
Deaths	High SDI	MDR-TB	Female	2028	0.0	0.0	155
Deaths	High SDI	MDR-TB	Female	2029	0.0	0.0	153
Deaths	High SDI	MDR-TB	Female	2030	0.0	0.0	151
Deaths	High SDI	MDR-TB	Both	1990	0.1	0.1	615
Deaths	High SDI	MDR-TB	Both	1991	0.1	0.1	789
Deaths	High SDI	MDR-TB	Both	1992	0.1	0.1	960
Deaths	High SDI	MDR-TB	Both	1993	0.1	0.1	1121
Deaths	High SDI	MDR-TB	Both	1994	0.1	0.1	1249
Deaths	High SDI	MDR-TB	Both	1995	0.1	0.2	1322
Deaths	High SDI	MDR-TB	Both	1996	0.1	0.2	1347
Deaths	High SDI	MDR-TB	Both	1997	0.1	0.2	1338
Deaths	High SDI	MDR-TB	Both	1998	0.1	0.2	1345
Deaths	High SDI	MDR-TB	Both	1999	0.1	0.2	1345
Deaths	High SDI	MDR-TB	Both	2000	0.1	0.1	1299
Deaths	High SDI	MDR-TB	Both	2001	0.1	0.1	1266
Deaths	High SDI	MDR-TB	Both	2002	0.1	0.1	1224
Deaths	High SDI	MDR-TB	Both	2003	0.1	0.1	1178
Deaths	High SDI	MDR-TB	Both	2004	0.1	0.1	1103
Deaths	High SDI	MDR-TB	Both	2005	0.1	0.1	1009
Deaths	High SDI	MDR-TB	Both	2006	0.1	0.1	911
Deaths	High SDI	MDR-TB	Both	2007	0.1	0.1	834
Deaths	High SDI	MDR-TB	Both	2008	0.1	0.1	732
Deaths	High SDI	MDR-TB	Both	2009	0.0	0.1	644
Deaths	High SDI	MDR-TB	Both	2010	0.0	0.1	597
Deaths	High SDI	MDR-TB	Both	2011	0.0	0.1	561
Deaths	High SDI	MDR-TB	Both	2012	0.0	0.1	543
Deaths	High SDI	MDR-TB	Both	2013	0.0	0.1	534
Deaths	High SDI	MDR-TB	Both	2014	0.0	0.1	522
Deaths	High SDI	MDR-TB	Both	2015	0.0	0.1	507
Deaths	High SDI	MDR-TB	Both	2016	0.0	0.1	505
Deaths	High SDI	MDR-TB	Both	2017	0.0	0.1	502
Deaths	High SDI	MDR-TB	Both	2018	0.0	0.1	506
Deaths	High SDI	MDR-TB	Both	2019	0.0	0.1	511
Deaths	High SDI	MDR-TB	Both	2020	0.0	0.0	452
Deaths	High SDI	MDR-TB	Both	2021	0.0	0.0	440
Deaths	High SDI	MDR-TB	Both	2022	0.0	0.0	426
Deaths	High SDI	MDR-TB	Both	2023	0.0	0.0	421
Deaths	High SDI	MDR-TB	Both	2024	0.0	0.0	417
Deaths	High SDI	MDR-TB	Both	2025	0.0	0.0	412
Deaths	High SDI	MDR-TB	Both	2026	0.0	0.0	407
Deaths	High SDI	MDR-TB	Both	2027	0.0	0.0	401
Deaths	High SDI	MDR-TB	Both	2028	0.0	0.0	394
Deaths	High SDI	MDR-TB	Both	2029	0.0	0.0	386
Deaths	High SDI	MDR-TB	Both	2030	0.0	0.0	378
Deaths	High-middle SDI	MDR-TB	Male	1990	0.5	0.4	2240
Deaths	High-middle SDI	MDR-TB	Male	1991	0.7	0.6	3475
Deaths	High-middle SDI	MDR-TB	Male	1992	1.0	0.9	4979
Deaths	High-middle SDI	MDR-TB	Male	1993	1.3	1.1	6739
Deaths	High-middle SDI	MDR-TB	Male	1994	1.6	1.5	8827

Deaths	High-middle SDI	MDR-TB	Male	1995	1.9	1.7	10432
Deaths	High-middle SDI	MDR-TB	Male	1996	2.0	1.9	11241
Deaths	High-middle SDI	MDR-TB	Male	1997	2.0	1.9	11685
Deaths	High-middle SDI	MDR-TB	Male	1998	2.0	2.0	12132
Deaths	High-middle SDI	MDR-TB	Male	1999	2.2	2.2	13414
Deaths	High-middle SDI	MDR-TB	Male	2000	2.3	2.3	14514
Deaths	High-middle SDI	MDR-TB	Male	2001	2.3	2.4	14835
Deaths	High-middle SDI	MDR-TB	Male	2002	2.3	2.4	15220
Deaths	High-middle SDI	MDR-TB	Male	2003	2.3	2.4	15469
Deaths	High-middle SDI	MDR-TB	Male	2004	2.2	2.4	15234
Deaths	High-middle SDI	MDR-TB	Male	2005	2.2	2.4	15506
Deaths	High-middle SDI	MDR-TB	Male	2006	1.9	2.1	14025
Deaths	High-middle SDI	MDR-TB	Male	2007	1.8	2.0	12980
Deaths	High-middle SDI	MDR-TB	Male	2008	1.6	1.8	12185
Deaths	High-middle SDI	MDR-TB	Male	2009	1.4	1.6	10779
Deaths	High-middle SDI	MDR-TB	Male	2010	1.3	1.5	10050
Deaths	High-middle SDI	MDR-TB	Male	2011	1.1	1.3	9154
Deaths	High-middle SDI	MDR-TB	Male	2012	1.1	1.2	8544
Deaths	High-middle SDI	MDR-TB	Male	2013	1.0	1.2	7957
Deaths	High-middle SDI	MDR-TB	Male	2014	0.9	1.1	7429
Deaths	High-middle SDI	MDR-TB	Male	2015	0.8	1.0	7205
Deaths	High-middle SDI	MDR-TB	Male	2016	0.8	1.0	6825
Deaths	High-middle SDI	MDR-TB	Male	2017	0.7	0.9	6391
Deaths	High-middle SDI	MDR-TB	Male	2018	0.7	0.9	6231
Deaths	High-middle SDI	MDR-TB	Male	2019	0.7	0.8	6072
Deaths	High-middle SDI	MDR-TB	Male	2020	0.6	0.8	2709
Deaths	High-middle SDI	MDR-TB	Male	2021	0.6	0.7	2530
Deaths	High-middle SDI	MDR-TB	Male	2022	0.5	0.6	2346
Deaths	High-middle SDI	MDR-TB	Male	2023	0.5	0.6	2264
Deaths	High-middle SDI	MDR-TB	Male	2024	0.5	0.6	2180
Deaths	High-middle SDI	MDR-TB	Male	2025	0.4	0.6	2093
Deaths	High-middle SDI	MDR-TB	Male	2026	0.4	0.5	2004
Deaths	High-middle SDI	MDR-TB	Male	2027	0.4	0.5	1913
Deaths	High-middle SDI	MDR-TB	Male	2028	0.4	0.5	1818
Deaths	High-middle SDI	MDR-TB	Male	2029	0.4	0.5	1721
Deaths	High-middle SDI	MDR-TB	Male	2030	0.3	0.4	1621
Deaths	High-middle SDI	MDR-TB	Female	1990	0.2	0.2	977
Deaths	High-middle SDI	MDR-TB	Female	1991	0.2	0.3	1469
Deaths	High-middle SDI	MDR-TB	Female	1992	0.3	0.3	1972
Deaths	High-middle SDI	MDR-TB	Female	1993	0.4	0.4	2416
Deaths	High-middle SDI	MDR-TB	Female	1994	0.5	0.5	2870
Deaths	High-middle SDI	MDR-TB	Female	1995	0.5	0.5	3208
Deaths	High-middle SDI	MDR-TB	Female	1996	0.5	0.6	3392
Deaths	High-middle SDI	MDR-TB	Female	1997	0.5	0.6	3469
Deaths	High-middle SDI	MDR-TB	Female	1998	0.5	0.6	3584
Deaths	High-middle SDI	MDR-TB	Female	1999	0.6	0.6	3847
Deaths	High-middle SDI	MDR-TB	Female	2000	0.6	0.6	4057
Deaths	High-middle SDI	MDR-TB	Female	2001	0.6	0.7	4192
Deaths	High-middle SDI	MDR-TB	Female	2002	0.6	0.7	4245
Deaths	High-middle SDI	MDR-TB	Female	2003	0.6	0.7	4254
Deaths	High-middle SDI	MDR-TB	Female	2004	0.6	0.6	4167

Deaths	High-middle SDI	MDR-TB	Female	2005	0.6	0.6	4201
Deaths	High-middle SDI	MDR-TB	Female	2006	0.5	0.6	3900
Deaths	High-middle SDI	MDR-TB	Female	2007	0.5	0.6	3746
Deaths	High-middle SDI	MDR-TB	Female	2008	0.4	0.5	3558
Deaths	High-middle SDI	MDR-TB	Female	2009	0.4	0.5	3222
Deaths	High-middle SDI	MDR-TB	Female	2010	0.4	0.4	3050
Deaths	High-middle SDI	MDR-TB	Female	2011	0.3	0.4	2842
Deaths	High-middle SDI	MDR-TB	Female	2012	0.3	0.4	2676
Deaths	High-middle SDI	MDR-TB	Female	2013	0.3	0.4	2613
Deaths	High-middle SDI	MDR-TB	Female	2014	0.3	0.4	2506
Deaths	High-middle SDI	MDR-TB	Female	2015	0.3	0.4	2466
Deaths	High-middle SDI	MDR-TB	Female	2016	0.3	0.3	2382
Deaths	High-middle SDI	MDR-TB	Female	2017	0.2	0.3	2275
Deaths	High-middle SDI	MDR-TB	Female	2018	0.2	0.3	2243
Deaths	High-middle SDI	MDR-TB	Female	2019	0.2	0.3	2195
Deaths	High-middle SDI	MDR-TB	Female	2020	0.2	0.3	1097
Deaths	High-middle SDI	MDR-TB	Female	2021	0.2	0.3	1048
Deaths	High-middle SDI	MDR-TB	Female	2022	0.2	0.3	998
Deaths	High-middle SDI	MDR-TB	Female	2023	0.2	0.3	974
Deaths	High-middle SDI	MDR-TB	Female	2024	0.2	0.3	949
Deaths	High-middle SDI	MDR-TB	Female	2025	0.2	0.2	924
Deaths	High-middle SDI	MDR-TB	Female	2026	0.2	0.2	899
Deaths	High-middle SDI	MDR-TB	Female	2027	0.2	0.2	874
Deaths	High-middle SDI	MDR-TB	Female	2028	0.2	0.2	848
Deaths	High-middle SDI	MDR-TB	Female	2029	0.2	0.2	822
Deaths	High-middle SDI	MDR-TB	Female	2030	0.1	0.2	795
Deaths	High-middle SDI	MDR-TB	Both	1990	0.3	0.3	3218
Deaths	High-middle SDI	MDR-TB	Both	1991	0.4	0.4	4944
Deaths	High-middle SDI	MDR-TB	Both	1992	0.6	0.6	6952
Deaths	High-middle SDI	MDR-TB	Both	1993	0.8	0.8	9156
Deaths	High-middle SDI	MDR-TB	Both	1994	1.0	1.0	11698
Deaths	High-middle SDI	MDR-TB	Both	1995	1.1	1.1	13640
Deaths	High-middle SDI	MDR-TB	Both	1996	1.2	1.2	14633
Deaths	High-middle SDI	MDR-TB	Both	1997	1.2	1.2	15154
Deaths	High-middle SDI	MDR-TB	Both	1998	1.2	1.3	15716
Deaths	High-middle SDI	MDR-TB	Both	1999	1.3	1.4	17261
Deaths	High-middle SDI	MDR-TB	Both	2000	1.4	1.5	18571
Deaths	High-middle SDI	MDR-TB	Both	2001	1.4	1.5	19028
Deaths	High-middle SDI	MDR-TB	Both	2002	1.4	1.5	19465
Deaths	High-middle SDI	MDR-TB	Both	2003	1.4	1.5	19723
Deaths	High-middle SDI	MDR-TB	Both	2004	1.3	1.5	19401
Deaths	High-middle SDI	MDR-TB	Both	2005	1.3	1.5	19707
Deaths	High-middle SDI	MDR-TB	Both	2006	1.2	1.4	17925
Deaths	High-middle SDI	MDR-TB	Both	2007	1.1	1.3	16727
Deaths	High-middle SDI	MDR-TB	Both	2008	1.0	1.2	15742
Deaths	High-middle SDI	MDR-TB	Both	2009	0.9	1.0	14001
Deaths	High-middle SDI	MDR-TB	Both	2010	0.8	1.0	13100
Deaths	High-middle SDI	MDR-TB	Both	2011	0.7	0.9	11995
Deaths	High-middle SDI	MDR-TB	Both	2012	0.7	0.8	11221
Deaths	High-middle SDI	MDR-TB	Both	2013	0.6	0.8	10571
Deaths	High-middle SDI	MDR-TB	Both	2014	0.6	0.7	9935

Deaths	High-middle SDI	MDR-TB	Both	2015	0.5	0.7	9671
Deaths	High-middle SDI	MDR-TB	Both	2016	0.5	0.7	9207
Deaths	High-middle SDI	MDR-TB	Both	2017	0.5	0.6	8666
Deaths	High-middle SDI	MDR-TB	Both	2018	0.5	0.6	8474
Deaths	High-middle SDI	MDR-TB	Both	2019	0.4	0.6	8267
Deaths	High-middle SDI	MDR-TB	Both	2020	0.4	0.5	3806
Deaths	High-middle SDI	MDR-TB	Both	2021	0.4	0.5	3579
Deaths	High-middle SDI	MDR-TB	Both	2022	0.3	0.5	3344
Deaths	High-middle SDI	MDR-TB	Both	2023	0.3	0.4	3238
Deaths	High-middle SDI	MDR-TB	Both	2024	0.3	0.4	3129
Deaths	High-middle SDI	MDR-TB	Both	2025	0.3	0.4	3017
Deaths	High-middle SDI	MDR-TB	Both	2026	0.3	0.4	2904
Deaths	High-middle SDI	MDR-TB	Both	2027	0.3	0.4	2787
Deaths	High-middle SDI	MDR-TB	Both	2028	0.3	0.4	2667
Deaths	High-middle SDI	MDR-TB	Both	2029	0.2	0.3	2543
Deaths	High-middle SDI	MDR-TB	Both	2030	0.2	0.3	2416
Deaths	Middle SDI	MDR-TB	Male	1990	0.9	0.5	4749
Deaths	Middle SDI	MDR-TB	Male	1991	1.3	0.8	7197
Deaths	Middle SDI	MDR-TB	Male	1992	1.8	1.1	9748
Deaths	Middle SDI	MDR-TB	Male	1993	2.1	1.3	11852
Deaths	Middle SDI	MDR-TB	Male	1994	2.4	1.5	13740
Deaths	Middle SDI	MDR-TB	Male	1995	2.6	1.6	15339
Deaths	Middle SDI	MDR-TB	Male	1996	2.7	1.7	16526
Deaths	Middle SDI	MDR-TB	Male	1997	2.7	1.8	17141
Deaths	Middle SDI	MDR-TB	Male	1998	2.7	1.8	17638
Deaths	Middle SDI	MDR-TB	Male	1999	2.8	1.8	18388
Deaths	Middle SDI	MDR-TB	Male	2000	2.8	1.9	19389
Deaths	Middle SDI	MDR-TB	Male	2001	2.8	2.0	19954
Deaths	Middle SDI	MDR-TB	Male	2002	2.8	2.0	20457
Deaths	Middle SDI	MDR-TB	Male	2003	2.8	2.0	20655
Deaths	Middle SDI	MDR-TB	Male	2004	2.6	1.9	20142
Deaths	Middle SDI	MDR-TB	Male	2005	2.5	1.9	19752
Deaths	Middle SDI	MDR-TB	Male	2006	2.3	1.8	19033
Deaths	Middle SDI	MDR-TB	Male	2007	2.2	1.7	18535
Deaths	Middle SDI	MDR-TB	Male	2008	2.1	1.7	18079
Deaths	Middle SDI	MDR-TB	Male	2009	1.9	1.6	17351
Deaths	Middle SDI	MDR-TB	Male	2010	1.8	1.5	16419
Deaths	Middle SDI	MDR-TB	Male	2011	1.7	1.4	15631
Deaths	Middle SDI	MDR-TB	Male	2012	1.6	1.4	15340
Deaths	Middle SDI	MDR-TB	Male	2013	1.5	1.3	14572
Deaths	Middle SDI	MDR-TB	Male	2014	1.4	1.2	13902
Deaths	Middle SDI	MDR-TB	Male	2015	1.3	1.1	13124
Deaths	Middle SDI	MDR-TB	Male	2016	1.2	1.1	13071
Deaths	Middle SDI	MDR-TB	Male	2017	1.2	1.1	12948
Deaths	Middle SDI	MDR-TB	Male	2018	1.1	1.1	12753
Deaths	Middle SDI	MDR-TB	Male	2019	1.1	1.0	12562
Deaths	Middle SDI	MDR-TB	Male	2020	1.0	1.1	15496
Deaths	Middle SDI	MDR-TB	Male	2021	0.9	1.0	14919
Deaths	Middle SDI	MDR-TB	Male	2022	0.9	1.0	14280
Deaths	Middle SDI	MDR-TB	Male	2023	0.8	1.0	14096
Deaths	Middle SDI	MDR-TB	Male	2024	0.8	0.9	13898

Deaths	Middle SDI	MDR-TB	Male	2025	0.8	0.9	13676
Deaths	Middle SDI	MDR-TB	Male	2026	0.7	0.9	13424
Deaths	Middle SDI	MDR-TB	Male	2027	0.7	0.9	13138
Deaths	Middle SDI	MDR-TB	Male	2028	0.7	0.9	12828
Deaths	Middle SDI	MDR-TB	Male	2029	0.6	0.8	12500
Deaths	Middle SDI	MDR-TB	Male	2030	0.6	0.8	12147
Deaths	Middle SDI	MDR-TB	Female	1990	0.5	0.4	3125
Deaths	Middle SDI	MDR-TB	Female	1991	0.8	0.5	4709
Deaths	Middle SDI	MDR-TB	Female	1992	1.0	0.7	6339
Deaths	Middle SDI	MDR-TB	Female	1993	1.2	0.9	7546
Deaths	Middle SDI	MDR-TB	Female	1994	1.4	1.0	8704
Deaths	Middle SDI	MDR-TB	Female	1995	1.4	1.0	9541
Deaths	Middle SDI	MDR-TB	Female	1996	1.5	1.1	9989
Deaths	Middle SDI	MDR-TB	Female	1997	1.5	1.1	10119
Deaths	Middle SDI	MDR-TB	Female	1998	1.4	1.1	10234
Deaths	Middle SDI	MDR-TB	Female	1999	1.4	1.1	10483
Deaths	Middle SDI	MDR-TB	Female	2000	1.4	1.1	10802
Deaths	Middle SDI	MDR-TB	Female	2001	1.4	1.1	10933
Deaths	Middle SDI	MDR-TB	Female	2002	1.4	1.1	10940
Deaths	Middle SDI	MDR-TB	Female	2003	1.4	1.1	10888
Deaths	Middle SDI	MDR-TB	Female	2004	1.3	1.0	10598
Deaths	Middle SDI	MDR-TB	Female	2005	1.2	1.0	10370
Deaths	Middle SDI	MDR-TB	Female	2006	1.2	1.0	10047
Deaths	Middle SDI	MDR-TB	Female	2007	1.1	0.9	9640
Deaths	Middle SDI	MDR-TB	Female	2008	1.0	0.9	9347
Deaths	Middle SDI	MDR-TB	Female	2009	0.9	0.8	8933
Deaths	Middle SDI	MDR-TB	Female	2010	0.9	0.8	8589
Deaths	Middle SDI	MDR-TB	Female	2011	0.8	0.8	8336
Deaths	Middle SDI	MDR-TB	Female	2012	0.8	0.7	8081
Deaths	Middle SDI	MDR-TB	Female	2013	0.7	0.7	7631
Deaths	Middle SDI	MDR-TB	Female	2014	0.7	0.6	7063
Deaths	Middle SDI	MDR-TB	Female	2015	0.6	0.6	6719
Deaths	Middle SDI	MDR-TB	Female	2016	0.6	0.6	6657
Deaths	Middle SDI	MDR-TB	Female	2017	0.6	0.6	6592
Deaths	Middle SDI	MDR-TB	Female	2018	0.5	0.6	6603
Deaths	Middle SDI	MDR-TB	Female	2019	0.5	0.5	6522
Deaths	Middle SDI	MDR-TB	Female	2020	0.5	0.5	7848
Deaths	Middle SDI	MDR-TB	Female	2021	0.4	0.5	7540
Deaths	Middle SDI	MDR-TB	Female	2022	0.4	0.5	7202
Deaths	Middle SDI	MDR-TB	Female	2023	0.4	0.5	7100
Deaths	Middle SDI	MDR-TB	Female	2024	0.4	0.5	6991
Deaths	Middle SDI	MDR-TB	Female	2025	0.4	0.5	6872
Deaths	Middle SDI	MDR-TB	Female	2026	0.3	0.5	6737
Deaths	Middle SDI	MDR-TB	Female	2027	0.3	0.4	6586
Deaths	Middle SDI	MDR-TB	Female	2028	0.3	0.4	6423
Deaths	Middle SDI	MDR-TB	Female	2029	0.3	0.4	6250
Deaths	Middle SDI	MDR-TB	Female	2030	0.3	0.4	6064
Deaths	Middle SDI	MDR-TB	Both	1990	0.7	0.5	7874
Deaths	Middle SDI	MDR-TB	Both	1991	1.0	0.7	11906
Deaths	Middle SDI	MDR-TB	Both	1992	1.4	0.9	16087
Deaths	Middle SDI	MDR-TB	Both	1993	1.6	1.1	19398

Deaths	Middle SDI	MDR-TB	Both	1994	1.8	1.2	22444
Deaths	Middle SDI	MDR-TB	Both	1995	2.0	1.3	24880
Deaths	Middle SDI	MDR-TB	Both	1996	2.1	1.4	26515
Deaths	Middle SDI	MDR-TB	Both	1997	2.1	1.4	27260
Deaths	Middle SDI	MDR-TB	Both	1998	2.0	1.4	27872
Deaths	Middle SDI	MDR-TB	Both	1999	2.1	1.5	28872
Deaths	Middle SDI	MDR-TB	Both	2000	2.1	1.5	30191
Deaths	Middle SDI	MDR-TB	Both	2001	2.1	1.5	30887
Deaths	Middle SDI	MDR-TB	Both	2002	2.1	1.5	31397
Deaths	Middle SDI	MDR-TB	Both	2003	2.0	1.5	31543
Deaths	Middle SDI	MDR-TB	Both	2004	1.9	1.5	30740
Deaths	Middle SDI	MDR-TB	Both	2005	1.8	1.4	30122
Deaths	Middle SDI	MDR-TB	Both	2006	1.7	1.4	29081
Deaths	Middle SDI	MDR-TB	Both	2007	1.6	1.3	28175
Deaths	Middle SDI	MDR-TB	Both	2008	1.5	1.3	27426
Deaths	Middle SDI	MDR-TB	Both	2009	1.4	1.2	26284
Deaths	Middle SDI	MDR-TB	Both	2010	1.3	1.1	25008
Deaths	Middle SDI	MDR-TB	Both	2011	1.2	1.1	23967
Deaths	Middle SDI	MDR-TB	Both	2012	1.2	1.0	23421
Deaths	Middle SDI	MDR-TB	Both	2013	1.1	1.0	22204
Deaths	Middle SDI	MDR-TB	Both	2014	1.0	0.9	20964
Deaths	Middle SDI	MDR-TB	Both	2015	0.9	0.9	19843
Deaths	Middle SDI	MDR-TB	Both	2016	0.9	0.8	19728
Deaths	Middle SDI	MDR-TB	Both	2017	0.9	0.8	19540
Deaths	Middle SDI	MDR-TB	Both	2018	0.8	0.8	19356
Deaths	Middle SDI	MDR-TB	Both	2019	0.8	0.8	19084
Deaths	Middle SDI	MDR-TB	Both	2020	0.7	0.8	23344
Deaths	Middle SDI	MDR-TB	Both	2021	0.7	0.8	22458
Deaths	Middle SDI	MDR-TB	Both	2022	0.6	0.7	21482
Deaths	Middle SDI	MDR-TB	Both	2023	0.6	0.7	21195
Deaths	Middle SDI	MDR-TB	Both	2024	0.6	0.7	20889
Deaths	Middle SDI	MDR-TB	Both	2025	0.6	0.7	20548
Deaths	Middle SDI	MDR-TB	Both	2026	0.5	0.7	20161
Deaths	Middle SDI	MDR-TB	Both	2027	0.5	0.7	19724
Deaths	Middle SDI	MDR-TB	Both	2028	0.5	0.6	19251
Deaths	Middle SDI	MDR-TB	Both	2029	0.5	0.6	18751
Deaths	Middle SDI	MDR-TB	Both	2030	0.4	0.6	18211
Deaths	Low-middle SDI	MDR-TB	Male	1990	0.6	0.3	1987
Deaths	Low-middle SDI	MDR-TB	Male	1991	0.9	0.6	3240
Deaths	Low-middle SDI	MDR-TB	Male	1992	1.4	0.8	5033
Deaths	Low-middle SDI	MDR-TB	Male	1993	2.0	1.2	7295
Deaths	Low-middle SDI	MDR-TB	Male	1994	2.7	1.6	10161
Deaths	Low-middle SDI	MDR-TB	Male	1995	3.5	2.1	13321
Deaths	Low-middle SDI	MDR-TB	Male	1996	4.2	2.6	16397
Deaths	Low-middle SDI	MDR-TB	Male	1997	4.9	3.0	19321
Deaths	Low-middle SDI	MDR-TB	Male	1998	5.5	3.3	22227
Deaths	Low-middle SDI	MDR-TB	Male	1999	6.1	3.7	25119
Deaths	Low-middle SDI	MDR-TB	Male	2000	6.5	4.0	27400
Deaths	Low-middle SDI	MDR-TB	Male	2001	6.8	4.2	29166
Deaths	Low-middle SDI	MDR-TB	Male	2002	6.9	4.3	30386
Deaths	Low-middle SDI	MDR-TB	Male	2003	6.9	4.3	30917

Deaths	Low-middle SDI	MDR-TB	Male	2004	6.6	4.2	30498
Deaths	Low-middle SDI	MDR-TB	Male	2005	6.5	4.2	31108
Deaths	Low-middle SDI	MDR-TB	Male	2006	6.4	4.2	31534
Deaths	Low-middle SDI	MDR-TB	Male	2007	6.3	4.2	31547
Deaths	Low-middle SDI	MDR-TB	Male	2008	6.2	4.2	32006
Deaths	Low-middle SDI	MDR-TB	Male	2009	5.9	4.1	31674
Deaths	Low-middle SDI	MDR-TB	Male	2010	5.7	4.0	31297
Deaths	Low-middle SDI	MDR-TB	Male	2011	5.5	3.9	31319
Deaths	Low-middle SDI	MDR-TB	Male	2012	5.5	4.0	32323
Deaths	Low-middle SDI	MDR-TB	Male	2013	5.6	4.0	33319
Deaths	Low-middle SDI	MDR-TB	Male	2014	5.6	4.0	33478
Deaths	Low-middle SDI	MDR-TB	Male	2015	5.3	3.9	32679
Deaths	Low-middle SDI	MDR-TB	Male	2016	5.2	3.9	33091
Deaths	Low-middle SDI	MDR-TB	Male	2017	5.1	3.9	33635
Deaths	Low-middle SDI	MDR-TB	Male	2018	5.0	3.8	33276
Deaths	Low-middle SDI	MDR-TB	Male	2019	4.7	3.7	32594
Deaths	Low-middle SDI	MDR-TB	Male	2020	4.7	3.6	41959
Deaths	Low-middle SDI	MDR-TB	Male	2021	4.6	3.6	42220
Deaths	Low-middle SDI	MDR-TB	Male	2022	4.5	3.5	42448
Deaths	Low-middle SDI	MDR-TB	Male	2023	4.5	3.6	43079
Deaths	Low-middle SDI	MDR-TB	Male	2024	4.4	3.6	43730
Deaths	Low-middle SDI	MDR-TB	Male	2025	4.4	3.6	44387
Deaths	Low-middle SDI	MDR-TB	Male	2026	4.3	3.6	45038
Deaths	Low-middle SDI	MDR-TB	Male	2027	4.3	3.6	45676
Deaths	Low-middle SDI	MDR-TB	Male	2028	4.2	3.6	46315
Deaths	Low-middle SDI	MDR-TB	Male	2029	4.2	3.6	46967
Deaths	Low-middle SDI	MDR-TB	Male	2030	4.1	3.6	47621
Deaths	Low-middle SDI	MDR-TB	Female	1990	0.4	0.2	1351
Deaths	Low-middle SDI	MDR-TB	Female	1991	0.6	0.4	2180
Deaths	Low-middle SDI	MDR-TB	Female	1992	0.9	0.6	3365
Deaths	Low-middle SDI	MDR-TB	Female	1993	1.2	0.8	4823
Deaths	Low-middle SDI	MDR-TB	Female	1994	1.7	1.1	6659
Deaths	Low-middle SDI	MDR-TB	Female	1995	2.1	1.4	8681
Deaths	Low-middle SDI	MDR-TB	Female	1996	2.5	1.7	10583
Deaths	Low-middle SDI	MDR-TB	Female	1997	2.9	2.0	12306
Deaths	Low-middle SDI	MDR-TB	Female	1998	3.2	2.2	13996
Deaths	Low-middle SDI	MDR-TB	Female	1999	3.6	2.4	15836
Deaths	Low-middle SDI	MDR-TB	Female	2000	3.8	2.6	17073
Deaths	Low-middle SDI	MDR-TB	Female	2001	3.9	2.7	18198
Deaths	Low-middle SDI	MDR-TB	Female	2002	4.0	2.8	18825
Deaths	Low-middle SDI	MDR-TB	Female	2003	3.9	2.7	18834
Deaths	Low-middle SDI	MDR-TB	Female	2004	3.7	2.6	18388
Deaths	Low-middle SDI	MDR-TB	Female	2005	3.7	2.6	18612
Deaths	Low-middle SDI	MDR-TB	Female	2006	3.6	2.6	18638
Deaths	Low-middle SDI	MDR-TB	Female	2007	3.4	2.5	18369
Deaths	Low-middle SDI	MDR-TB	Female	2008	3.3	2.4	18184
Deaths	Low-middle SDI	MDR-TB	Female	2009	3.1	2.3	17579
Deaths	Low-middle SDI	MDR-TB	Female	2010	3.0	2.3	17441
Deaths	Low-middle SDI	MDR-TB	Female	2011	2.9	2.2	17634
Deaths	Low-middle SDI	MDR-TB	Female	2012	2.8	2.2	17733
Deaths	Low-middle SDI	MDR-TB	Female	2013	2.9	2.3	18389

Deaths	Low-middle SDI	MDR-TB	Female	2014	2.8	2.2	18447
Deaths	Low-middle SDI	MDR-TB	Female	2015	2.9	2.3	19115
Deaths	Low-middle SDI	MDR-TB	Female	2016	2.8	2.3	19356
Deaths	Low-middle SDI	MDR-TB	Female	2017	2.7	2.3	19341
Deaths	Low-middle SDI	MDR-TB	Female	2018	2.6	2.2	19120
Deaths	Low-middle SDI	MDR-TB	Female	2019	2.5	2.1	18719
Deaths	Low-middle SDI	MDR-TB	Female	2020	2.6	2.1	24566
Deaths	Low-middle SDI	MDR-TB	Female	2021	2.6	2.1	24893
Deaths	Low-middle SDI	MDR-TB	Female	2022	2.5	2.1	25217
Deaths	Low-middle SDI	MDR-TB	Female	2023	2.5	2.2	25726
Deaths	Low-middle SDI	MDR-TB	Female	2024	2.5	2.2	26253
Deaths	Low-middle SDI	MDR-TB	Female	2025	2.5	2.2	26793
Deaths	Low-middle SDI	MDR-TB	Female	2026	2.5	2.2	27342
Deaths	Low-middle SDI	MDR-TB	Female	2027	2.5	2.2	27897
Deaths	Low-middle SDI	MDR-TB	Female	2028	2.5	2.3	28468
Deaths	Low-middle SDI	MDR-TB	Female	2029	2.4	2.3	29060
Deaths	Low-middle SDI	MDR-TB	Female	2030	2.4	2.3	29667
Deaths	Low-middle SDI	MDR-TB	Both	1990	0.5	0.3	3338
Deaths	Low-middle SDI	MDR-TB	Both	1991	0.7	0.5	5420
Deaths	Low-middle SDI	MDR-TB	Both	1992	1.1	0.7	8399
Deaths	Low-middle SDI	MDR-TB	Both	1993	1.6	1.0	12119
Deaths	Low-middle SDI	MDR-TB	Both	1994	2.2	1.4	16820
Deaths	Low-middle SDI	MDR-TB	Both	1995	2.8	1.8	22002
Deaths	Low-middle SDI	MDR-TB	Both	1996	3.4	2.1	26980
Deaths	Low-middle SDI	MDR-TB	Both	1997	3.9	2.5	31628
Deaths	Low-middle SDI	MDR-TB	Both	1998	4.4	2.8	36223
Deaths	Low-middle SDI	MDR-TB	Both	1999	4.8	3.1	40955
Deaths	Low-middle SDI	MDR-TB	Both	2000	5.1	3.3	44473
Deaths	Low-middle SDI	MDR-TB	Both	2001	5.3	3.5	47364
Deaths	Low-middle SDI	MDR-TB	Both	2002	5.4	3.5	49210
Deaths	Low-middle SDI	MDR-TB	Both	2003	5.4	3.5	49751
Deaths	Low-middle SDI	MDR-TB	Both	2004	5.1	3.4	48886
Deaths	Low-middle SDI	MDR-TB	Both	2005	5.1	3.4	49719
Deaths	Low-middle SDI	MDR-TB	Both	2006	5.0	3.4	50171
Deaths	Low-middle SDI	MDR-TB	Both	2007	4.8	3.3	49917
Deaths	Low-middle SDI	MDR-TB	Both	2008	4.7	3.3	50190
Deaths	Low-middle SDI	MDR-TB	Both	2009	4.5	3.2	49253
Deaths	Low-middle SDI	MDR-TB	Both	2010	4.3	3.1	48738
Deaths	Low-middle SDI	MDR-TB	Both	2011	4.2	3.1	48953
Deaths	Low-middle SDI	MDR-TB	Both	2012	4.2	3.1	50056
Deaths	Low-middle SDI	MDR-TB	Both	2013	4.2	3.2	51708
Deaths	Low-middle SDI	MDR-TB	Both	2014	4.1	3.1	51925
Deaths	Low-middle SDI	MDR-TB	Both	2015	4.0	3.1	51794
Deaths	Low-middle SDI	MDR-TB	Both	2016	3.9	3.1	52447
Deaths	Low-middle SDI	MDR-TB	Both	2017	3.9	3.1	52976
Deaths	Low-middle SDI	MDR-TB	Both	2018	3.7	3.0	52396
Deaths	Low-middle SDI	MDR-TB	Both	2019	3.6	2.9	51313
Deaths	Low-middle SDI	MDR-TB	Both	2020	3.6	2.9	66525
Deaths	Low-middle SDI	MDR-TB	Both	2021	3.6	2.9	67112
Deaths	Low-middle SDI	MDR-TB	Both	2022	3.5	2.9	67665
Deaths	Low-middle SDI	MDR-TB	Both	2023	3.5	2.9	68805

Deaths	Low-middle SDI	MDR-TB	Both	2024	3.4	2.9	69983
Deaths	Low-middle SDI	MDR-TB	Both	2025	3.4	2.9	71180
Deaths	Low-middle SDI	MDR-TB	Both	2026	3.4	2.9	72380
Deaths	Low-middle SDI	MDR-TB	Both	2027	3.3	2.9	73573
Deaths	Low-middle SDI	MDR-TB	Both	2028	3.3	2.9	74783
Deaths	Low-middle SDI	MDR-TB	Both	2029	3.3	3.0	76027
Deaths	Low-middle SDI	MDR-TB	Both	2030	3.2	3.0	77288
Deaths	Low SDI	MDR-TB	Male	1990	0.7	0.4	1037
Deaths	Low SDI	MDR-TB	Male	1991	1.1	0.6	1696
Deaths	Low SDI	MDR-TB	Male	1992	1.8	1.0	2693
Deaths	Low SDI	MDR-TB	Male	1993	2.6	1.4	4112
Deaths	Low SDI	MDR-TB	Male	1994	3.7	2.0	5975
Deaths	Low SDI	MDR-TB	Male	1995	5.0	2.7	8178
Deaths	Low SDI	MDR-TB	Male	1996	6.3	3.3	10415
Deaths	Low SDI	MDR-TB	Male	1997	7.2	3.8	12197
Deaths	Low SDI	MDR-TB	Male	1998	8.2	4.3	14095
Deaths	Low SDI	MDR-TB	Male	1999	8.9	4.6	15599
Deaths	Low SDI	MDR-TB	Male	2000	9.2	4.7	16437
Deaths	Low SDI	MDR-TB	Male	2001	9.2	4.8	16935
Deaths	Low SDI	MDR-TB	Male	2002	9.2	4.7	17295
Deaths	Low SDI	MDR-TB	Male	2003	9.2	4.7	17624
Deaths	Low SDI	MDR-TB	Male	2004	8.9	4.6	17623
Deaths	Low SDI	MDR-TB	Male	2005	8.8	4.4	17653
Deaths	Low SDI	MDR-TB	Male	2006	8.6	4.3	17680
Deaths	Low SDI	MDR-TB	Male	2007	8.4	4.3	17914
Deaths	Low SDI	MDR-TB	Male	2008	8.1	4.1	17771
Deaths	Low SDI	MDR-TB	Male	2009	8.0	4.1	17923
Deaths	Low SDI	MDR-TB	Male	2010	7.7	3.9	17865
Deaths	Low SDI	MDR-TB	Male	2011	7.6	3.9	17998
Deaths	Low SDI	MDR-TB	Male	2012	7.5	3.8	18366
Deaths	Low SDI	MDR-TB	Male	2013	7.6	3.8	18854
Deaths	Low SDI	MDR-TB	Male	2014	7.4	3.8	18981
Deaths	Low SDI	MDR-TB	Male	2015	7.3	3.7	19254
Deaths	Low SDI	MDR-TB	Male	2016	7.1	3.7	19329
Deaths	Low SDI	MDR-TB	Male	2017	7.0	3.6	19527
Deaths	Low SDI	MDR-TB	Male	2018	6.8	3.5	19613
Deaths	Low SDI	MDR-TB	Male	2019	6.6	3.5	19651
Deaths	Low SDI	MDR-TB	Male	2020	6.6	3.4	16626
Deaths	Low SDI	MDR-TB	Male	2021	6.5	3.4	16845
Deaths	Low SDI	MDR-TB	Male	2022	6.4	3.3	17051
Deaths	Low SDI	MDR-TB	Male	2023	6.3	3.3	17381
Deaths	Low SDI	MDR-TB	Male	2024	6.2	3.3	17729
Deaths	Low SDI	MDR-TB	Male	2025	6.1	3.3	18088
Deaths	Low SDI	MDR-TB	Male	2026	6.0	3.2	18449
Deaths	Low SDI	MDR-TB	Male	2027	6.0	3.2	18806
Deaths	Low SDI	MDR-TB	Male	2028	5.9	3.2	19170
Deaths	Low SDI	MDR-TB	Male	2029	5.8	3.2	19548
Deaths	Low SDI	MDR-TB	Male	2030	5.7	3.2	19935
Deaths	Low SDI	MDR-TB	Female	1990	0.4	0.3	671
Deaths	Low SDI	MDR-TB	Female	1991	0.7	0.4	1094
Deaths	Low SDI	MDR-TB	Female	1992	1.0	0.6	1732

Deaths	Low SDI	MDR-TB	Female	1993	1.5	0.9	2612
Deaths	Low SDI	MDR-TB	Female	1994	2.2	1.3	3758
Deaths	Low SDI	MDR-TB	Female	1995	2.9	1.7	5090
Deaths	Low SDI	MDR-TB	Female	1996	3.6	2.1	6430
Deaths	Low SDI	MDR-TB	Female	1997	4.1	2.4	7482
Deaths	Low SDI	MDR-TB	Female	1998	4.7	2.7	8639
Deaths	Low SDI	MDR-TB	Female	1999	5.1	2.9	9585
Deaths	Low SDI	MDR-TB	Female	2000	5.2	3.0	10066
Deaths	Low SDI	MDR-TB	Female	2001	5.3	3.0	10406
Deaths	Low SDI	MDR-TB	Female	2002	5.3	3.0	10666
Deaths	Low SDI	MDR-TB	Female	2003	5.2	2.9	10833
Deaths	Low SDI	MDR-TB	Female	2004	5.1	2.8	10811
Deaths	Low SDI	MDR-TB	Female	2005	5.0	2.8	10870
Deaths	Low SDI	MDR-TB	Female	2006	4.9	2.7	10851
Deaths	Low SDI	MDR-TB	Female	2007	4.7	2.6	10653
Deaths	Low SDI	MDR-TB	Female	2008	4.5	2.5	10601
Deaths	Low SDI	MDR-TB	Female	2009	4.4	2.5	10670
Deaths	Low SDI	MDR-TB	Female	2010	4.3	2.4	10739
Deaths	Low SDI	MDR-TB	Female	2011	4.2	2.3	10787
Deaths	Low SDI	MDR-TB	Female	2012	4.2	2.3	10853
Deaths	Low SDI	MDR-TB	Female	2013	4.1	2.3	10929
Deaths	Low SDI	MDR-TB	Female	2014	4.1	2.2	11067
Deaths	Low SDI	MDR-TB	Female	2015	4.0	2.2	11175
Deaths	Low SDI	MDR-TB	Female	2016	3.9	2.1	11130
Deaths	Low SDI	MDR-TB	Female	2017	3.8	2.1	11106
Deaths	Low SDI	MDR-TB	Female	2018	3.7	2.0	11138
Deaths	Low SDI	MDR-TB	Female	2019	3.6	2.0	11131
Deaths	Low SDI	MDR-TB	Female	2020	3.6	1.9	9396
Deaths	Low SDI	MDR-TB	Female	2021	3.5	1.9	9500
Deaths	Low SDI	MDR-TB	Female	2022	3.4	1.9	9596
Deaths	Low SDI	MDR-TB	Female	2023	3.4	1.9	9765
Deaths	Low SDI	MDR-TB	Female	2024	3.3	1.8	9943
Deaths	Low SDI	MDR-TB	Female	2025	3.3	1.8	10125
Deaths	Low SDI	MDR-TB	Female	2026	3.2	1.8	10308
Deaths	Low SDI	MDR-TB	Female	2027	3.2	1.8	10487
Deaths	Low SDI	MDR-TB	Female	2028	3.2	1.8	10668
Deaths	Low SDI	MDR-TB	Female	2029	3.1	1.8	10855
Deaths	Low SDI	MDR-TB	Female	2030	3.1	1.8	11046
Deaths	Low SDI	MDR-TB	Both	1990	0.6	0.3	1708
Deaths	Low SDI	MDR-TB	Both	1991	0.9	0.5	2790
Deaths	Low SDI	MDR-TB	Both	1992	1.4	0.8	4425
Deaths	Low SDI	MDR-TB	Both	1993	2.1	1.2	6723
Deaths	Low SDI	MDR-TB	Both	1994	3.0	1.7	9733
Deaths	Low SDI	MDR-TB	Both	1995	4.0	2.2	13268
Deaths	Low SDI	MDR-TB	Both	1996	4.9	2.7	16845
Deaths	Low SDI	MDR-TB	Both	1997	5.7	3.1	19679
Deaths	Low SDI	MDR-TB	Both	1998	6.4	3.5	22733
Deaths	Low SDI	MDR-TB	Both	1999	6.9	3.8	25184
Deaths	Low SDI	MDR-TB	Both	2000	7.2	3.9	26504
Deaths	Low SDI	MDR-TB	Both	2001	7.2	3.9	27341
Deaths	Low SDI	MDR-TB	Both	2002	7.2	3.9	27961

Deaths	Low SDI	MDR-TB	Both	2003	7.2	3.8	28457
Deaths	Low SDI	MDR-TB	Both	2004	7.0	3.7	28434
Deaths	Low SDI	MDR-TB	Both	2005	6.8	3.6	28523
Deaths	Low SDI	MDR-TB	Both	2006	6.7	3.5	28531
Deaths	Low SDI	MDR-TB	Both	2007	6.5	3.4	28567
Deaths	Low SDI	MDR-TB	Both	2008	6.3	3.3	28372
Deaths	Low SDI	MDR-TB	Both	2009	6.2	3.3	28593
Deaths	Low SDI	MDR-TB	Both	2010	6.0	3.2	28604
Deaths	Low SDI	MDR-TB	Both	2011	5.9	3.1	28785
Deaths	Low SDI	MDR-TB	Both	2012	5.8	3.1	29220
Deaths	Low SDI	MDR-TB	Both	2013	5.8	3.1	29783
Deaths	Low SDI	MDR-TB	Both	2014	5.7	3.0	30047
Deaths	Low SDI	MDR-TB	Both	2015	5.6	3.0	30429
Deaths	Low SDI	MDR-TB	Both	2016	5.4	2.9	30459
Deaths	Low SDI	MDR-TB	Both	2017	5.3	2.8	30633
Deaths	Low SDI	MDR-TB	Both	2018	5.2	2.8	30751
Deaths	Low SDI	MDR-TB	Both	2019	5.1	2.7	30783
Deaths	Low SDI	MDR-TB	Both	2020	5.0	2.7	26022
Deaths	Low SDI	MDR-TB	Both	2021	4.9	2.6	26345
Deaths	Low SDI	MDR-TB	Both	2022	4.8	2.6	26647
Deaths	Low SDI	MDR-TB	Both	2023	4.8	2.6	27146
Deaths	Low SDI	MDR-TB	Both	2024	4.7	2.6	27672
Deaths	Low SDI	MDR-TB	Both	2025	4.7	2.5	28213
Deaths	Low SDI	MDR-TB	Both	2026	4.6	2.5	28757
Deaths	Low SDI	MDR-TB	Both	2027	4.5	2.5	29293
Deaths	Low SDI	MDR-TB	Both	2028	4.5	2.5	29838
Deaths	Low SDI	MDR-TB	Both	2029	4.4	2.5	30404
Deaths	Low SDI	MDR-TB	Both	2030	4.3	2.5	30980
